# *closo*‐Carboranyl Analogs of β‐Arylethylamines: Direct Synthesis from Alkenes via EnT‐Catalysis

**DOI:** 10.1002/anie.202504793

**Published:** 2025-05-19

**Authors:** Fritz Paulus, Corinna Heusel, Marc Jaspers, Lilli M. Amrehn, Florian Schreiner, Debanjan Rana, Constantin G. Daniliuc, Michael Ryan Hansen, Frank Glorius

**Affiliations:** ^1^ Organisch‐Chemisches Institut University of Münster Corrensstraße 36 48149 Münster Germany; ^2^ Institut für Physikalische Chemie University of Münster Corrensstraße 28/30 48149 Münster Germany

**Keywords:** Bioisosteres, Carboranes, Carboranyl radicals, Energy transfer catalysis, β‐Arylethylamines

## Abstract

*closo*‐Carboranes are icosahedral carbon–boron clusters with unique properties and broad applicability. They particularly stand out in the context of drug development as privileged structural motifs for boron neutron capture therapy (BNCT) and as highly hydrophobic bioisosteres for the rotational volume of phenyl rings. Herein, we unveil the synthesis of N‐protected carboranyl analogs of β‐arylethylamines—widely found structural motifs in biologically active molecules—via a one‐step alkene difunctionalization approach. Key for our success were the enabling mechanistic characteristics of energy transfer catalysis which we have used for the first time to generate *closo*‐carboranyl radicals. Downstream modifications gave a series of analogs of amino acids and known *N*‐methyl‐d‐aspartate receptor (NMDAR) antagonists.

## Graphical abstract

A strategy to directly synthesize *closo*‐carboranyl analogs of medicinal chemically important β‐arylethylamines from alkenes is reported. This reaction is enabled by the first formation of *closo*‐carboranyl radicals by energy transfer (EnT) catalysis and its unique mechanistic features. Several analogs of known bioactive compounds were obtained after downstream modifications.

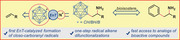

## Introduction

**Figure 1:**
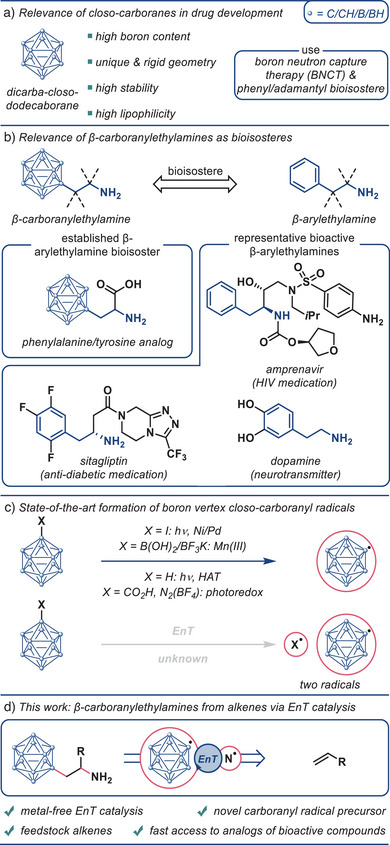
a) Relevance of *closo*‐carboranes in drug development. b) Relevance of β‐carboranylethylamines as bioisosteres. c) State‐of‐the‐art formation of boron vertex closo‐carboranyl radicals. d) This work: β‐carboranylethylamines from alkenes via EnT catalysis. BNCT = boron neutron capture therapy. HAT = hydrogen atom transfer. EnT = energy transfer.

Carboranes are carbon‐ and boron‐containing, three‐dimensional clusters with extraordinary chemical and physical properties.^[^ ^1^, ^2^, ^3^, ^4^ ^]^ While there is a large variety of structurally diverse carboranes, icosahedral dicarba‐*closo*‐dodecaboranes are arguably the most relevant subclass. Existing as *ortho*‐, *meta*‐, or *para*‐isomers—determined by the relative positions of the two carbon atoms in the cage skeleton—they possess a rigid 3D aromatic framework, leading to highly stable structures.^[^ ^1^, ^2^, ^3^, ^4^ ^]^ Due to this characteristic as well as their high lipophilicity and distinct electronical properties, dicarba‐*closo*‐dodecaboranes have attracted significant attention in the context of various research fields such as polymers,^[^ ^5^, ^6^, ^7^ ^]^ luminescent materials,^[^ ^8^, ^9^, ^10^ ^]^ ligand design,^[^ ^11^, ^12^, ^13^, ^14^, ^15^, ^16^, ^17^ ^]^ and others.^[^ ^1^, ^2^, ^18^, ^19^, ^20^, ^21^, ^22^, ^23^, ^24^, ^25^ ^]^ Furthermore, dicarba‐*closo*‐dodecaboranes are of high interest to medicinal chemistry because of 1) their high boron content which makes them a perfectly suitable moiety for cancer treatment with boron neutron capture therapy (BNCT), and 2) their bioisosterism of, e.g., adamantyl groups and a phenyl group's rotational volume (Figure 1a).^[^ ^2^, ^4^, ^26^, ^27^, ^28^, ^29^, ^30^, ^31^, ^32^ ^]^

An important class of carboranyl‐containing compounds are β‐carboranylethylamines, as they are bioisosteres of β‐arylethylamines—common motifs in bioactive molecules (Figure 1b).^[^ ^33^, ^34^, ^35^ ^]^ One particularly relevant representative of this class is *β*‐carboranyl‐*α*‐alanine which is a well‐studied, highly hydrophobic 3D bioisostere of phenylalanine and tyrosine (Figure 1b).^[^ ^36^, ^37^, ^38^, ^39^, ^40^, ^41^, ^42^, ^43^ ^]^ However, despite their great potential, other β‐carboranylethylamines are rare and their use as bioisosteres of β‐arylethylamines remains mostly unexplored, possibly due to limited available synthetic methods toward these compounds. Filling this synthetic gap by a straightforward and mild preparation approach—ideally by introducing the carboranyl group and the nitrogen moiety together in one reaction step—consequently is an important goal.

The selective late‐stage introduction of carboranyl groups to easily access complex carboranyl‐containing molecules is highly desirable. However, the carboranes’ high stability and arising vertex selectivity issues inherently render these processes challenging.^[^ ^1^, ^4^, ^44^ ^]^ A straightforward strategy to incorporate these moieties is the generation of carboranyl radicals and their subsequent radical addition to unsaturated functional groups. While carboranyl radicals have been known for more than 30 years,^[^ ^45^, ^46^, ^47^, ^48^ ^]^ their synthetic use has only recently attracted increased interest with important contributions on boron‐centered carboranyl radicals from Xie,^[^ ^49^, ^50^, ^51^ ^]^ Spokoyny,^[^ ^52^ ^]^ and Yan^[^ ^44^, ^53^ ^]^ (Figure 1c). In addition, several transformations have been developed utilizing carbon‐centered carboranyl radicals.^[^ ^54^, ^55^, ^56^, ^57^, ^58^ ^]^ Notably, carboranyl radicals have never been generated by energy transfer catalysis.^[^ ^59^, ^60^, ^61^ ^]^ This strategy, however, would be particularly mild, would allow for the use of a metal‐free photosensitizer, and would create a straight‐forward mechanistic design platform to achieve radical difunctionalizations of alkenes with a carboranyl radical and a second radical, rapidly constructing sought‐after scaffolds from feedstock alkenes.

Herein, we report the successful development of a novel bifunctional carboranyl radical precursor allowing for the unprecedented generation of carboranyl radicals via metal‐free energy transfer catalysis. These boron‐centered radicals were synthetically leveraged to achieve the first radical carboranylamination of alkenes (Figure 1d). Downstream modifications of the obtained products led to numerous carboranyl analogs of known bioactive compounds.

## Results and Discussion

### Reaction Development

**Figure 2:**
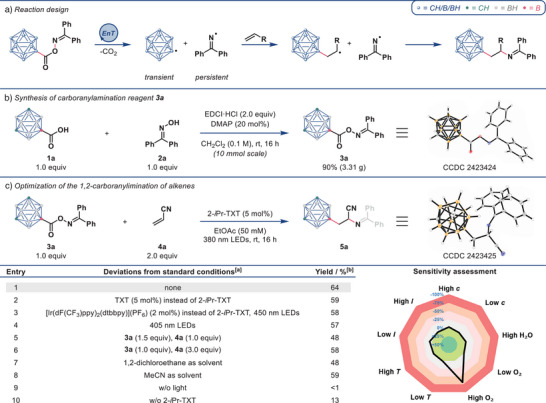
a) Reaction design. b) Synthesis of carboranylamination reagent **3a**. c) Optimization of the 1,2‐carboranylimination of alkenes. [a] Standard reaction conditions: **3a** (0.05 mmol, 1.0 equiv), **4a** (0.1 mmol, 2.0 equiv), 2‐*i*Pr‐TXT (5 mol%), EtOAc (1.0 mL, 50 mM), 380 nm LEDs (18 W), rt, 16 h. [b] Yields were determined by ^1^H NMR spectroscopy with mesitylene as internal standard. 2‐*i*Pr‐TXT = 2‐isopropylthioxanthone. TXT = thioxanthone.

To realize the radical difunctionalization of alkenes with both a carboranyl and a primary amine group, the combination of energy transfer catalysis with a novel benzophenone imine‐based bifunctional reagent—a type of reagent which was mainly pioneered by our group—would be a promising approach (Figure 2a).^[^ ^33^, ^62^, ^63^, ^64^, ^65^, ^66^, ^67^, ^68^, ^69^, ^70^, ^71^, ^72^, ^73^, ^74^, ^75^, ^76^ ^]^ The benzophenone imine moiety is employed to ensure high regioselectivity of the difunctionalization process and at the same time serves as a conveniently masked and readily deprotected primary amine, circumventing isolation issues and detrimental side reactions associated with free primary amines. In the envisioned mechanism (Figure 2a), energy transfer from an excited photocatalyst would lead to homolytic N─O bond cleavage in the carboranyl reagent and subsequent decarboxylation. Addition of the formed transient carboranyl radical to the alkene and terminal radical recombination with the persistent iminyl radical would then allow for the selective 1,2‐difunctionalization of alkenes.

We chose to commence our investigation with 9‐*meta*‐carborane carboxylic acid **1a**,^[^ ^44^ ^]^ and coupled it with oxime **2a** using EDCI. Solid carboranylamination reagent **3a** was smoothly obtained in 90% yield on a 10 mmol scale (Figure 2b). The reagent's structure was further confirmed by X‐ray analysis.^[^ ^77^ ^]^

With an effective synthesis of reagent **3a** in hand, we next started testing its reaction with alkenes. We were pleased to obtain the desired 1,2‐difunctionalization product **5a** in 64% NMR yield from the reaction of **3a** (1.0 equiv) and **4a** (2.0 equiv) in ethyl acetate (50 mM) using 2‐isopropylthioxanthone (2‐*i*Pr‐TXT, 5 mol%) under irradiation with 380 nm LEDs (Figure 2c, entry 1). Notably, the used catalyst is a cheap, commercially available substance. Insights into occurring side products are presented in the Supporting Information. Furthermore, we investigated the effect of modifying reagent **3a** with additional electron‐withdrawing, electron‐donating, or sterically hindering substituents at its benzophenone imine moiety (see the Supporting Information for experimental details). Amongst the tested reagents **3**, reagent **3a**—derived from simple benzophenone—proved most effective in carboranyliminating alkene **4a** under the abovementioned conditions.

Replacing 2‐*i*Pr‐TXT with thioxanthone (TXT, 5 mol%, 380 nm) or [Ir(dF(CF_3_)ppy)_2_(dtbbpy)](PF_6_) (2 mol%, 450 nm) led to slightly lower NMR yields of 59% and 58%, respectively (entries 2 and 3). Irradiation at 405 nm gave the desired product in 57% NMR yield (entry 4). Employing alkene **4a** as limiting starting material with 1.5 equiv of **3a** caused an NMR yield of 48% (entry 5), and a larger excess of alkene **4a** (3.0 equiv) led to a slightly decreased NMR yield of 58% (entry 6). The reaction is relatively robust regarding the choice of solvent, as illustrated by running the reaction in 1,2‐dichloroethane or acetonitrile (entries 7 and 8). No product formation was observed in the dark (entry 9), while in the absence of a photocatalyst, a 13% NMR yield was obtained (entry 10). This finding could be explained by an occurring minor direct excitation background reaction.

To identify critical reaction parameters, we performed a reaction condition‐based sensitivity assessment (Figure 2c).^[^ ^78^, ^79^ ^]^ Slight changes in concentration, light intensity, and temperature had no major effect on the detected yield. The same held true when performing the reaction with added water or with low oxygen (achieved by three freeze‐pump‐thaw cycles of the reaction mixture). A large decrease in yield was only observed, when performing the reaction under air.

### Substrate Scope

Utilizing the optimized reaction conditions, we next set out to investigate the reaction's substrate scope (Table 1). Most compounds were isolated as the imine, however, in case of poor separation during silica gel column chromatography, we isolated the product as the amine hydrochloride after mild acidic hydrolysis. At first, we questioned the ability of different reagents **3** to form the desired products **5**. Product **5a** containing a *m*‐carboranyl unit connected via a boron vertex was isolated in 59% yield using reagent **3a**. A boron vertex‐attached *o*‐carboranyl moiety was smoothly incorporated into the product as well, delivering **5b** in 57% yield. Unfortunately, reagents containing a *m*‐ or *o*‐carboranyl group attached via a carbon vertex did not give the respective products in substantial yields (see the Supporting Information for further details).

**Table 1:** Substrate scope.^[^ ^a^ ^]^

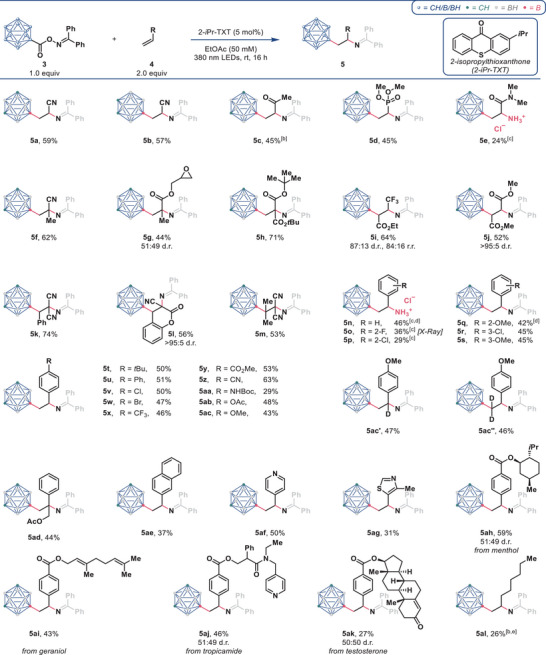

^[a]^ Isolated yields, d.r.s, and r.r.s are given. Standard reaction conditions: **3** (0.1 mmol, 1.0 equiv), **4** (0.2 mmol, 2.0 equiv), 2‐*i*Pr‐TXT (5 mol%), EtOAc (2.0 mL, 50 mM), 380 nm LEDs (18 W), rt, 16 h.

^[b]^ Reaction performed at 0.2 mmol scale.

^[c]^ Isolated yield after deprotection of the impure product with 1 M aq. HCl (1.2 equiv) in MeOH (0.15 M).

^[d]^ Reaction performed at 0.3 mmol scale.

^[e]^ Alkene **4ak** (10 equiv) used.

Examining the scope of alkenes using reagent **3a** revealed the suitability of a broad set of substrates. Other monosubstituted alkenes with different electron‐withdrawing groups, such as ketone (**5c**) and phosphonate ester (**5d**) delivered the desired products in 45% yield. Using *N*,*N*‐dimethylacrylamide also gave the product which was further hydrolyzed to give a 24% yield of amine hydrochloride **5e**. 1,1‐Disubstituted methacrylonitrile gave the corresponding product **5f** in 62% yield. Various 1,1‐ and 1,2‐substituted acrylates delivered products **5g**–**5j** in 44%–71% yield, whereby **5g** demonstrated the tolerance of a sensitive epoxide group. Due to the large steric bulk of the carboranyl group, products **5i** and **5j**, resulting from acyclic 1,2‐disubstituted alkenes, were obtained in high diastereomeric ratios.^[^ ^80^, ^81^ ^]^ Interestingly, **5i** showed inverted regioselectivity—with respect to the ester group—compared to other acrylates. Trisubstituted benzylidenemalononitrile gave product **5k** in 74% yield. A coumarin scaffold could also be addressed, delivering a 56% yield of **5l** with > 95:5 diastereomeric ratio. Difunctionalized product **5m** was formed in 53% yield from tetrasubstituted isopropylidenemalononitrile.

Styrenes also proved to be viable substrates. Styrene, *o*‐fluorostyrene, and *o*‐chlorostyrene gave amine hydrochlorides **5n**–**5p** in 29%–46% yield. *o*‐Methoxystyrene, *m*‐chlorostyrene, and *m*‐methoxystyrene delivered the corresponding products **5q**–**5s** in 42%–45% yield. Finally, a set of *para*‐substituted styrenes was examined to get a good overview about the functional group tolerance of the present protocol. Alkyl, aryl, chloride, and bromide groups were well tolerated, giving **5t**–**5w** in 47%–51% yield. Electron‐withdrawing trifluoromethyl, methyl ester, and nitrile groups led to **5x**–**5z** in 46%–63% yield—electron‐donating Boc‐protected amine, acetoxy, and methoxy groups led to **5aa**–**5ac** in 29%–48% yield. Differently deuterated products **5ac‘** and **5ac‘‘** were isolated in similar yields as the non‐deuterated compound, demonstrating the convenient synthesis of isotope labeled β‐carboranylethylamines. Compound **5ad** was formed from a 1,1‐disubstituted styrene in 44% yield. Styrenes containing other aromatic core structures such as naphthalene, pyridine, and thiazole formed the corresponding products **5ae**–**5ag** in 31%–50% yield. Lastly, we sought to demonstrate the applicability of our reaction to the modification of alkenes containing natural product and drug scaffolds. Products derived from menthol (**5ah**), geraniol (**5ai**), tropicamide (**5aj**), and testosterone (**5ak**) were obtained in 27%–59% yield and further showed the tolerance of unactivated alkenes and an amide. Notably, in the reaction toward **5ak**, the styrene moiety was selectively difunctionalized in the presence of the sterically hindered α,β‐unsaturated ketone contained in testosterone.

In addition, we were pleased to find that an unactivated alkene delivered the desired product **5al**, albeit with a reduced yield even when using 10 equivalents of the alkene.

### Mechanistic Study

**Figure 3:**
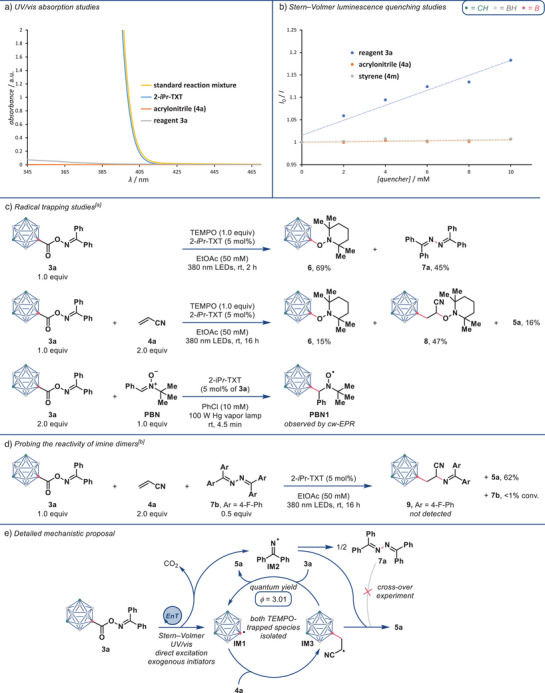
Selected mechanistic studies. a) UV–vis absorption studies at half the standard concentrations. b) Stern–Volmer luminescence quenching studies. c) Radical trapping studies. d) Cross‐over experiment with fluorinated imine dimer **7b**. e) Detailed mechanistic proposal based on the conducted mechanistic experiments. ^[a]^ Isolated yields given. ^[b]^ Yields and conversions were determined by ^1^H NMR spectroscopy with mesitylene as internal standard. TEMPO = (2,2,6,6‐tetramethylpiperidin‐1‐yl)oxyl. PBN = *N*‐*tert*‐butyl‐*α*‐phenylnitrone.

We next sought to obtain a more detailed mechanistic understanding of the occurring processes. For that purpose, we first wanted to get insights into the initial reaction step involving the energy transfer‐catalyzed cleavage of bifunctional reagent **3a** into two radicals **IM1** and **IM2**. UV–vis absorption studies of the reaction mixture and the individual components revealed that the reaction mixture's absorption at the reaction wavelength almost completely results from the photocatalyst (Figure 3a). Reagent **3a** shows very minor absorption at that wavelength which could enable the observed minor background reaction without photocatalyst. Stern–Volmer luminescence quenching studies highlighted quenching of 2‐*i*Pr‐TXT by reagent **3a** and negligible quenching by acrylonitrile (**4a**) and styrene (**4m**) (Figure 3b). Notably, the carboranyl moiety—compared to an alkyl group—has no major effect on both the absorption properties of reagent **3a** and its triplet energy (see the Supporting Information for comparative UV–vis spectra and computational triplet energy calculations). Running the standard reaction at direct excitation conditions (without photocatalyst under irradiation at 365 nm for 48 h) gave product **5a** in 48% NMR yield (see the Supporting Information for experimental details). Replacing the photocatalyst with the common radical starters azobisisobutyronitrile (AIBN) or di‐*tert*‐butyl peroxide (DTBP) and carrying out this reaction in the dark at 75 °C, led to NMR yields below 1% (see the Supporting Information for experimental details). Taken together, these results support that the first reaction step involves 2‐*i*Pr‐TXT‐catalyzed energy transfer to **3a**.

We then turned our attention to the detection of reaction intermediates (Figure 3c). For this purpose, we first conducted the standard reaction without an alkene **4** but with added (2,2,6,6‐tetramethylpiperidin‐1‐yl)oxyl (TEMPO). The TEMPO adduct with carboranyl radical **IM1** was isolated from this reaction mixture in 69% yield (**6**), and imine dimer **7a** was isolated in 45% yield. This experiment demonstrates that carboranyl radical **IM1** and iminyl radical **IM2** are formed under the applied conditions and that iminyl radical **IM2** readily dimerizes. Next, we added TEMPO to the standard reaction with alkene **4a**. In addition to **6** (15% isolated yield), we were able to isolate TEMPO‐trapped **IM3** in 47% yield (**8**) thus having isolated all mentioned radical key intermediates **IM1**–**IM3** as their TEMPO adducts or dimer. Desired product **5a** was obtained in 16% isolated yield from this reaction which is significantly lower than for the reaction without TEMPO and therefore further supports the involvement of radicals. We additionally tried trapping of radical intermediates with butylated hydroxytoluene (BHT) and observed the masses of adducts of **IM2** and **IM3** with BHT as well as a substantially lowered NMR yield of **5a** (6%, see the Supporting Information for experimental details). An additional investigation of the fate of radicals **IM1** and **IM2** in the absence of an alkene and radical scavenger can be found in the Supporting Information. Furthermore, we studied the photochemical cleavage of **3a** by continuous‐wave electron paramagnetic resonance (cw‐EPR). Spin trapping experiments with *N*‐*tert*‐butyl‐*α*‐phenylnitrone (PBN) under in situ UV–vis irradiation suggested the formation of the PBN adduct of radical **IM1** (**PBN1**, see the Supporting Information for the recorded cw‐EPR spectra, and the fitted EPR parameters).^[^ ^82^ ^]^

Subsequently, we questioned, whether the commonly observed imine dimer **7a** can be attacked by **IM3** to form **5a** and **IM2** (see the Supporting Information for a detailed mechanistic explanation), thus serving as an iminyl reservoir. To probe this, we conceived a cross‐over experiment with added fluorinated imine dimer **7b** (Figure 3d). The conversion of imine dimer **7b** was below 1%, **5a** was formed in 62% NMR yield, and we did not detect cross‐over product **9**, therefore concluding that imine dimer side products do not participate in the reaction. Lastly, we determined the reaction quantum yield for the formation of **5a**. The obtained value of *Φ* = 3.01 is significantly larger than 1 and consequently suggests an operating radical chain pathway in addition to simple radical recombination.^[^ ^62^, ^63^ ^]^

Based on these insights, we propose a detailed mechanistic picture (Figure 3e). The reaction is initiated by energy transfer to bifunctional reagent **3a**. N─O bond cleavage and CO_2_ extrusion delivers persistent iminyl radical **IM2** and transient carboranyl radical **IM1**. Carboranyl radical **IM1** then adds to alkene **4a** generating **IM3**. This intermediate can form **5a** either via recombination with iminyl radical **IM2** or via reaction with another molecule of **3a** which releases another carboranyl radical **IM1**. Imine dimer **7a** forms by recombination of two iminyl radicals **IM2** and does not further engage in the reaction.

We believe that these combined mechanistic insights and the resulting detailed mechanistic picture will significantly influence further developments within the field of energy transfer‐cleavable bifunctional reagents.

### Product Diversification

**Figure 4:**
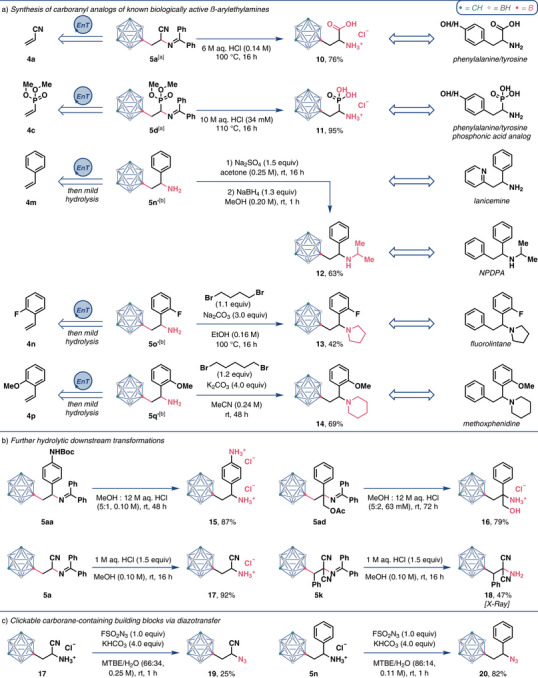
Product diversifications. See the Supporting Information for experimental details. a) Synthesis of carboranyl analogs of known biologically active β‐arylethylamines. b) Further hydrolytic downstream transformations. c) Clickable carborane‐containing building blocks via diazotransfer. [a] See Table 1 for the synthesis. [b] See the Supporting Information for the synthesis.

Having developed a straight‐forward one‐step method toward protected β‐carboranylethylamines from alkenes, we sought to unleash the full potential of this strategy by preparing concrete carboranyl analogs of known bioactive β‐arylethylamines (Figure 4a).

Two phenylalanine/tyrosine analogs were chosen as our first targets. Simple acidic hydrolysis of **5a** delivered β‐carboranyl‐α‐alanine **10**, in 76% yield. While the synthesis and application of β‐carboranyl‐α‐alanine is well established,^[^ ^36^ ^]^ to the best of our knowledge, this structure has only been prepared with the carboranyl moiety connected via a carbon atom. The boron vertex‐attached isomer **10** therefore offers the valuable opportunity to fine‐tune the properties of this amino acid and of peptides containing this amino acid. Amino phosphonic acid **11** was obtained by acidic hydrolysis of **5d** with a yield of 95% and is an analog of known bioactive amino phosphonic acids found for example in naturally occurring tripeptide K‐26.^[^ ^83^, ^84^ ^]^

Next, we aimed at preparing a representative set of analogs of 1,2‐diarylethylamines—an established class of *N*‐methyl‐d‐aspartate receptor (NMDAR) antagonists.^[^ ^85^ ^]^ Lanicemine analog **5n** was obtained in 46% yield from styrene (**4m**) via difunctionalization and subsequent hydrolysis of the imine moiety. A sequence of acidic deprotection of the imine‐protected products **5** and alkylation of the formed primary amines gave compounds **12** (NPDPA analog), **13** (fluorolintane analog), and **14** (methoxphenidine analog).

Further downstream acidic hydrolyses delivered diamine hydrochloride **15** in 87% yield and 1,2‐amino alcohol hydrochloride **16** in 79% yield (Figure 4b). Both compounds **15** and **16** offer many opportunities for additional modifications. Subsequently, we prepared amine hydrochloride **17** from **5a** in 92% yield and amine **18** from **5k** in 47% yield via mild hydrolysis.

Lastly, we prepared two representative azides (**19** and **20**) from amine hydrochlorides **17** and **5n** via diazotransfer (Figure 4c).^[^ ^86^ ^]^ These building blocks contain a carborane connected to an azide via a short and easily tunable linker, making them highly suitable for the introduction of carboranyl groups—e.g., for BNCT applications—via CuAAC click reactions.^[^ ^87^, ^88^ ^]^

## Conclusion

In summary, we have developed a novel carborane‐containing bifunctional reagent and its application to the one‐step synthesis of N‐protected β‐carboranylethylamines. This metal‐free difunctionalization approach is enabled by the first energy transfer‐catalyzed formation of *closo*‐carboranyl radicals and offers efficient access to the desired products from simple alkenes. Downstream modifications gave rise to multiple carboranyl analogs of biologically active compounds, demonstrating this method's value. Ongoing work will evaluate the biological activity of these compounds. We believe that the simplicity and effectiveness of the presented protocol will stimulate the more widespread medicinal chemical use of β‐carboranylethylamines and of carboranyl groups in general.

## Conflict of Interests

The authors declare no conflict of interest.

## Supporting information



Supporting Information

Supporting Information

## Data Availability

The data that support the findings of this study are available in the Supporting Information of this article.
