# Supplementary material for: closo‐Carboranyl Analogs of β‐Arylethylamines: Direct Synthesis from Alkenes via EnT‐Catalysis
Source: Angew Chem Int Ed Engl. 2025 May 19;64(27):e202504793. doi: 10.1002/anie.202504793 (PMC12207362; doi:10.1002/anie.202504793)
Supplement: Supplementary file 1 — Supporting Information [file ANIE-64-e202504793-s002.pdf]

closo-Carboranyl Analogs of β-Arylethylamines: Direct Synthesis from Alkenes via EnT-Catalysis

Fritz Paulus,[a] Corinna Heusel,[a] Marc Jaspers,[a] Lilli M. Amrehn,[a] Florian Schreiner,[b] Debanjan Rana,[a] Constantin G. Daniliuc,[a] Michael Ryan Hansen,[b] and Frank Glorius*[a]

[a]Organisch-Chemisches Institut, University of Münster, Corrensstraße 36, 48149 Münster, Germany.

[b]Institut für Physikalische Chemie, University of Münster, Corrensstraße 28/30, 48149 Münster, Germany.

*glorius@uni-muenster.de

SUPPORTING INFORMATION

Contents

1. General information 3

1.1 Experimental conditions, reagents, and solvent 3

1.2 Analytical techniques 3

1.3 Purification techniques 4

1.4 Photochemical set-up 4

2. Starting materials 6

2.1 Carborane carboxylic acids 1 6

2.2 Carboranylamination reagents 3 10

2.3 Alkenes 4 17

3. Reaction development 19

3.1 Crude analysis 19

3.2 Sensitivity assessment 20

3.3 Optimization of the benzophenone imine moiety of 3 22

4. Substrate scope 23

4.1 Characterization data 23

4.2 Limitations 52

5. Mechanistic experiments 53

5.1 UV/vis absorption spectroscopy 53

5.2 Stern–Volmer luminescence quenching studies 53

5.3 Direct excitation experiment 54

5.4 Radical trapping studies 55

5.5 Reaction in the absence of an alkene 59

5.6 EPR studies 60

5.7 Evaluation of common radical starters AIBN and DTBP 62

5.8 Quantum yield measurement 63

5.9 Cross-over experiment with a different imine dimer 65
5.10 The carborane's effect on the photophysical properties of reagents 3 69
5.10.1 UV/vis analysis 69
5.10.2 Computational studies 69
6. Product diversifications 79
6.1 Synthesis of carboranyl analogs of known biologically active β-arylethylamines 79
6.2 Further hydrolytic downstream transformations 84
6.3 Clickable carborane-containing building blocks via diazotransfer 86
7. X-Ray analysis 88
8. NMR spectra 93
8.1 Starting materials 93
8.2 Substrate scope 116
8.3 Mechanistic investigations 204
8.4 Product diversifications 213
9. References 239

1. General information

1.1 Experimental conditions, reagents, and solvent

Reactions were carried out in oven-dried glassware under argon, using standard Schlenk techniques, unless otherwise stated. Given reaction temperatures are the ones of the heating/cooling media and the reactions were stirred using PTFE-coated magnetic stirring bars. Removal of low boiling solvents was achieved by rotary evaporation under reduced pressure, using a water bath at 40 °C.

Thioxanthone and 2-isopropylthioxanthone were purchased from BLD Pharm and used as received. [Ir(dF(CF$_3$)ppy)$_2$(dtbbpy)](PF$_6$) (**[Ir-F]**) was synthesized according to a literature procedure.[1] Other commercially available chemicals were purchased from commercial suppliers and were used without further purification, unless otherwise noted.

The following solvents were purchased from ACROS Organics, Fischer Scientific, and Sigma-Aldrich (HPLC grade), dried using a solvent purification system (SPS) with activated alumina columns – custom-built by the "Feinmechanische Werkstatt des Organisch-Chemischen Instituts, Universität Münster" – and collected under positive argon pressure: dichloromethane (CH_2Cl_2), acetonitrile (MeCN), tetrahydrofuran (THF), diethylether (Et_2O), *N*,*N*-dimethylformamide (DMF), toluene, and methanol (MeOH). Other dry solvents were bought from ACROS Organics (ACROS ExtraDry solvents with ACROSeal® cap), stored over 3 or 4 Å molecular sieves, and collected under positive argon pressure.

n-Pentane, CH_2Cl_2, and ethyl acetate (EtOAc) used for column chromatography, extractions, etc., were purchased of technical grade and purified by atmospheric pressure distillation.

1.2 Analytical techniques

GC samples were filtered over a short plug of silica eluting with ethyl acetate prior to analysis, if not stated otherwise. GC-MS spectra were recorded on an Agilent Technologies 7890A GC-system (Agilent 5975C VL MSD or an Agilent 5975 MSD) with a HP-5MS column (0.25 mm · 30 m, film: 0.25 μm).

NMR spectra were recorded at room temperature in deuterated solvents using a Bruker Avance II 400, Bruker Avance Neo 400, Agilent DD2 500 or an Agilent DD2 600 spectrometer. Chemical shifts (δ) for ^{1}H and ^{13}C NMR spectra are reported in parts per million (ppm) relative to tetramethylsilane (TMS) using the residual solvent signals as references for ^{1}H and ^{13}C NMR spectra ($CDCl_3$: δ_H = 7.26 ppm, δ_C = 77.16 ppm; CD_2Cl_2: δ_H = 5.32 ppm, δ_C = 53.84 ppm; D_2O: δ_H = 4.79 ppm, ^{13}C NMR spectra not referenced; acetone-d$_6$: δ_H = 2.05 ppm, δ_C = 29.84 ppm; MeOH-

d$_4$: δ_H = 3.31 ppm, δ_C = 49.00 ppm). ^{11}B, ^{19}F, and ^{31}P NMR spectra are not referenced. NMR-signal multiplicities are reported using the following abbreviations (or combination thereof): s = singlet, d = doublet, t = triplet, q = quartet, p = quintet, h = sextet; hept = heptet; m = multiplet, br = broad signal. Coupling constants (*J*) are quoted in Hz. The spectra were processed using MestReNova 15, typically applying standard phase and baseline correction.

ESI high-resolution mass spectra (HRMS) were recorded by the mass spectrometry department of the Organisch-Chemisches Institut, University of Münster, on a MicroToF spectrometer (Bruker Daltronics) or on an Orbitap LTQ XL (Thermo-Fisher Scientific).

1.3 Purification techniques

Thin layer chromatography (TLC) was performed using Merck silica gel 60 F254 aluminum plates. Visualization was achieved with UV light (254 nm) and/or staining with basic $KMnO_4$ solution (4 g of $KMnO_4$, 10 g K_2CO_3, 1 g NaOH in 200 mL of distilled water) or acidic $PdCl_2$ solution (1 wt.% $PdCl_2$ in 6 M aq. HCl). Flash column chromatography was carried out manually using standard techniques with silica gel (40-63 mesh) by Merck and the specified solvent system under 0.3-0.5 bar overpressure.

Imine-containing products are acid-sensitive and were typically isolated using deactivated silica for column chromatography. Deactivated silica was prepared by adding 250 mL of silica to a 500 mL round-bottom flask, adding *n*-pentane to make a slurry, and then adding 7 mL of triethylamine. After mixing well, the volatiles were removed under reduced pressure to obtain the basified silica.

1.4 Photochemical set-up

Photochemical reactions were performed in a Hepatochem EvoluChem™ PhotoRedOx Box Duo device and irradiated with two EvoluChem™ HCK1012-01-008 LEDs (30 W, λ_{max} = 450 nm), two EvoluChem™ HCK1012-01-010 LEDs (18 W, λ_{max} = 405 nm), two EvoluChem™ HCK1012-01-006 LEDs (30 W, λ_{max} = 365 nm), or two EvoluChem™ HCK1012-01-013 LEDs (18 W, λ_{max} = 380 nm). The reaction temperature in this setup was measured to be between 30 °C and 35 °C.

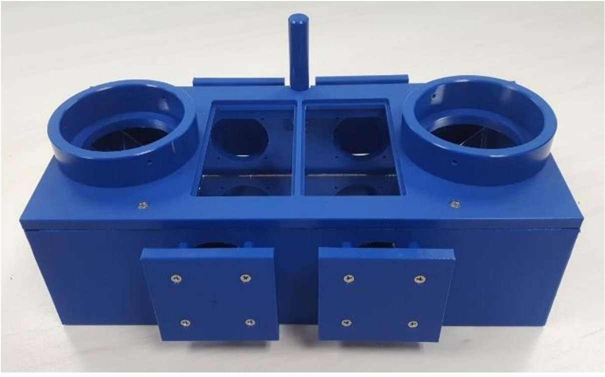

Supplementary Figure 1. Hepatochem EvoluChem™ PhotoRedOx Box Duo (without light sources and vial holders). Courtesy of Hepatochem.

2. Starting materials

2.1 Carborane carboxylic acids 1

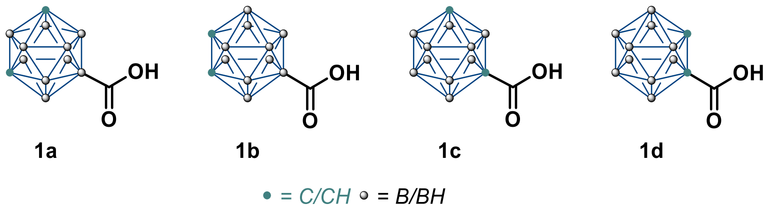

Supplementary Figure 2. Used carboxylic acids **1**.

9-*meta*-Carborane carboxylic acid (1a)

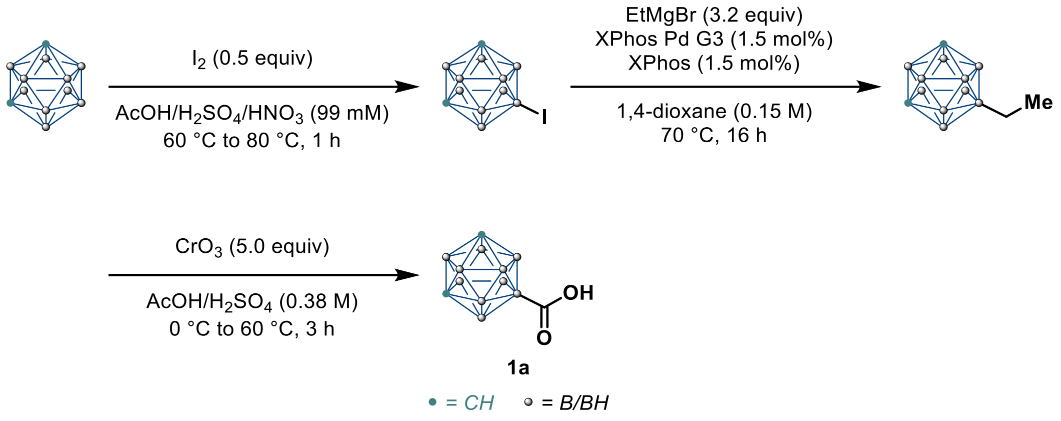

The title compound was prepared according to an adapted literature procedure.[2] A Schlenk tube was charged with a magnetic stirring bar, *m*-carborane (2.70 g, 18.7 mmol, 1.0 equiv), glacial acetic acid (135 mL), and iodine (2.38 g, 9.38 mmol, 0.50 equiv) under air. A mixture of concentrated sulfuric acid and concentrated nitric acid (1:1, 54 mL) was added dropwise at 60 °C, and the reaction mixture was then stirred at 80 °C for 1 h. Subsequently, the reaction mixture was cooled down, diluted with distilled water (500 mL), and the formed white precipitate was collected by filtration. The solid was washed with distilled water (50 mL) and was then dissolved in diethyl ether (150 mL). After washing the organic layer with aqueous Na_2SO_3 (0.1 M, 50 mL), the organic layer was dried over $MgSO_4$ and the solvent was evaporated. Drying in vacuo delivered 9-iodo-*meta*-carborane as a white solid which was taken to the next step without further purification.

A Schlenk tube was charged with a magnetic stirring bar, the obtained 9-iodo-*meta*-carborane (4.41 g, 16.3 mmol, 1.0 equiv), XPhos Pd G3 (207 mg, 0.245 mmol, 1.5 mol%), and XPhos (117 mg, 0.245 mmol, 1.5 mol%). The tube was evacuated and backfilled with argon three times and dry 1,4-dioxane (110 mL, 0.15 M) was added, followed by the dropwise addition of ethylmagnesium bromide (3 M in diethyl ether, 17.4 mL, 52.2 mmol, 3.2 equiv). After stirring at 70 °C for 16 h,

diethyl ether (50 mL) was added, and the reaction was quenched by the addition of distilled water (50 mL). The layers were separated and the aqueous layer was further extracted with diethyl ether (3 x 50 mL). All organic layers were combined, dried over Na_2SO_4, and evaporated. Column chromatography on silica (100% *n*-pentane) gave 9-ethyl-*meta*-carborane as a colorless oil which was immediately taken to the next reaction step.

A 250 mL round-bottom flask was charged with a magnetic stirring bar, the obtained 9-ethyl-*meta*-carborane (2.61 g, 15.2 mmol, 1.0 equiv), glacial acetic acid (36 mL), and concentrated sulfuric acid (3.6 mL). The mixture was cooled to 0 °C and CrO_3 (7.62 g, 76.2 mmol, 5.0 equiv) was added portionwise. Subsequently, the mixture was stirred at room temperature for 30 mins and at 60 °C for 2 h. After cooling to room temperature, distilled water (200 mL) was added, and the formed precipitate was filtered off and washed with distilled water (5 x 10 mL). Drying in vacuo gave the title compound as a pale green solid (2.11 g, 11.2 mmol, 60% over 3 steps).

^{1}H NMR (400 MHz, acetone-d_6) δ 10.16 (s, 1H), 3.77 (s, 2H) 3.50 – 1.30 (m, 9H).

^{13}C{^{1}H} NMR (101 MHz, acetone-d_6) δ 56.4. *The carbon NMR signal of the carboxylic acid group is not visible due to quadrupolar broadening.*

^{11}B{^{1}H} NMR (128 MHz, acetone-d_6) δ -6.8, -10.2, -13.0, -13.6, -16.9, -17.7.

HRMS (ESI): *m/z* calculated for $[C_3H_{12}O_2B_{10}Na]^+$ $[M+Na]^+$: 211.1734; found: 211.1732.

Note 1: 9-Ethyl-meta-carborane is volatile and requires careful drying.

Note 2: Heating up the last reaction step needs to be done carefully.

9-*ortho*-Carborane carboxylic acid (1b)

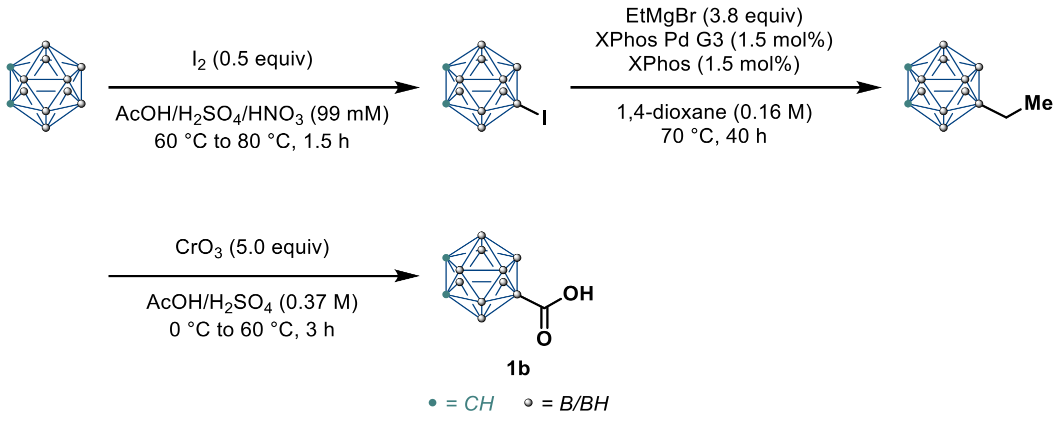

The title compound was prepared according to an adapted literature procedure.[2] A Schlenk tube was charged with a magnetic stirring bar, *o*-carborane (300 mg, 2.08 mmol, 1.0 equiv), glacial acetic acid (15 mL), and iodine (264 mg, 1.04 mmol, 0.50 equiv) under air. A mixture of

concentrated sulfuric acid and concentrated nitric acid (1:1, 6 mL) was added dropwise at 60 °C, and the reaction mixture was then stirred at 80 °C for 1.5 h. Subsequently, the reaction mixture was cooled down, diluted with distilled water (100 mL), and the formed white precipitate was collected by filtration. The solid was washed with distilled water (10 mL) and was then dissolved in diethyl ether (30 mL). After washing the organic layer with aqueous Na_2SO_3 (0.1 M, 10 mL), the organic layer was dried over $MgSO_4$ and the solvent was evaporated. Drying in vacuo delivered 9-iodo-*ortho*-carborane as a white solid which was taken to the next step without further purification.

A Schlenk tube was charged with a magnetic stirring bar, the obtained 9-iodo-*ortho*-carborane (507 mg, 1.88 mmol, 1.0 equiv), XPhos Pd G3 (24.5 mg, 0.0289 mmol, 1.5 mol%), and XPhos (13.8 mg, 0.0289 mmol, 1.5 mol%). The tube was evacuated and backfilled with argon three times and dry 1,4-dioxane (12 mL, 0.16 M) was added, followed by the dropwise addition of ethylmagnesium bromide (3 M in diethyl ether, 1.9 mL, 5.7 mmol, 3.0 equiv). After stirring at 70 °C for 24 h, more ethylmagnesium bromide (3 M in diethyl ether, 0.5 mL, 1.5 mmol, 0.80 equiv) was added and stirring at 70 °C was continued for 16 more hours. Subsequently, diethyl ether (10 mL) was added, and the reaction was quenched by the addition of distilled water (10 mL). The layers were separated and the aqueous layer was further extracted with diethyl ether (3 x 20 mL). All organic layers were combined, dried over Na_2SO_4, and evaporated. Column chromatography on silica (100% *n*-pentane) gave 9-ethyl-*ortho*-carborane as a colorless oil which was immediately taken to the next reaction step.

A 250 mL round-bottom flask was charged with a magnetic stirring bar, the obtained 9-ethyl-*ortho*-carborane (205 mg, 1.19 mmol, 1.0 equiv), glacial acetic acid (2.9 mL), and concentrated sulfuric acid (0.29 mL). The mixture was cooled to 0 °C and CrO_3 (595 mg, 5.95 mmol, 5.0 equiv) was added portionwise. Subsequently, the mixture was stirred at room temperature for 30 mins and at 60 °C for 2 h. After cooling to room temperature, distilled water (50 mL) was added, and the formed precipitate was filtered off and washed with distilled water (3 x 10 mL). Drying in vacuo gave the title compound as a pale green solid (120 mg, 0.638 mmol, 31% over 3 steps).

^{1}H NMR (400 MHz, $CDCl_3$) δ 3.81 – 3.43 (m, 2H), 3.25 – 1.14 (m, 9H).

^{13}C{^{1}H} NMR (101 MHz, $CDCl_3$) δ 53.6, 52.9. *The carbon NMR signal of the carboxylic acid group is not visible due to quadrupolar broadening.*

^{11}B{^{1}H} NMR (128 MHz, $CDCl_3$) δ -0.8, -2.3, -9.0, -13.9, -15.2.

HRMS (ESI): *m/z* calculated for $[C_3H_{12}O_2B_{10}Na]^+$ $[M+Na]^+$: 211.1734; found: 211.1731.

Note 1: 9-Ethyl-ortho-carborane is volatile and requires careful drying.

Note 2: Heating up the last reaction step needs to be done carefully.

1-***meta***-Carborane carboxylic acid (1c)

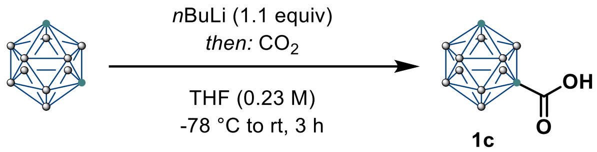

1c

• = C/CH ∘ = BH

The title compound was prepared according to a modified literature procedure.[3] A 10 mL Schlenk tube was charged with a magnetic stirring bar and *m*-carborane (100 mg, 0.693 mmol, 1.0 equiv). The tube was evacuated and backfilled with argon three times, cooled to -78 °C, and dry THF (3.0 mL, 0.23 M) was added. At this temperature, *n*BuLi (1.6 M in hexanes, 0.48 mL, 0.768 mmol, 1.1 equiv) was added dropwise and the mixture was stirred at room temperature for 30 mins. Then, the solution was purged with CO_2 (formed from dry ice and passed through concentrated sulfuric acid for drying) for 20 mins and then, a balloon filled with CO_2 was attached to the reaction mixture. Stirring at room temperature was continued for 2.5 h. The reaction was stopped by the addition of distilled water (5 mL), and the mixture was extracted with *n*-pentane (3 x 5 mL). All organic layers were disposed of. The aqueous layer was acidified with aq. HCl (1 M, 1.0 mL) during which white precipitate formed. Extraction with *n*-pentane (3 x 5 mL), drying of the combined organic layers with Na_2SO_4, and evaporation of the solvent gave the title compound as a white solid (75.6 mg, 0.402 mmol, 58%).

^{1}H NMR (400 MHz, $CDCl_3$) δ 5.00 (s, 1H), 3.03 (s, 1H), 3.72 – 1.48 (m, 10H).

^{13}C{^{1}H} NMR (101 MHz, $CDCl_3$) δ 166.1, 71.4, 55.0.

^{11}B{^{1}H} NMR (128 MHz, $CDCl_3$) δ -4.9, -6.6, -10.6, -11.3, -13.2, -15.7.

HRMS (ESI): *m/z* calculated for $[C_3H_{11}O_2B_{10}]^-$ $[M\text{-}H]^-$: 187.1769; found: 187.1768.

1-***ortho***-Carborane carboxylic acid (1d)

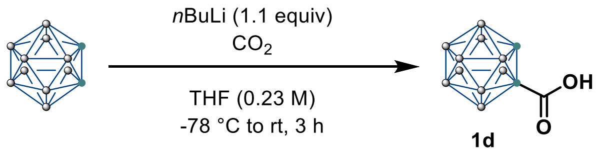

1d

• = C/CH ∘ = BH

The title compound was prepared according to a modified literature procedure.[3] A 10 mL Schlenk tube was charged with a magnetic stirring bar and *o*-carborane (100 mg, 0.693 mmol, 1.0 equiv).

The tube was evacuated and backfilled with argon three times, cooled to -78 °C, and dry THF (3.0 mL, 0.23 M) was added. At this temperature, *n*BuLi (1.6 M in hexanes, 0.48 mL, 0.768 mmol, 1.1 equiv) was added dropwise and the mixture was stirred at room temperature for 30 mins. Then, the solution was purged with CO_2 (formed from dry ice and passed through concentrated sulfuric acid for drying) for 20 mins and then, a balloon filled with CO_2 was attached to the reaction mixture. Stirring at room temperature was continued for 2.5 h. The reaction was stopped by the addition of distilled water (5 mL), and the mixture was extracted with *n*-pentane (3 x 5 mL). All organic layers were disposed of. The aqueous layer was acidified with aq. HCl (1 M, 1.0 mL) during which white precipitate formed. Extraction with *n*-pentane (3 x 5 mL), drying of the combined organic layers with Na_2SO_4, and evaporation of the solvent gave the title compound as a white solid (88.9 mg, 0.472 mmol, 68%).

^{1}H NMR (400 MHz, $CDCl_3$) δ 7.29 (s, 1H), 4.05 (s, 1H), 3.24 – 1.39 (m, 10H).

^{13}C{^{1}H} NMR (101 MHz, $CDCl_3$) δ 164.9, 68.0, 57.0.

^{11}B{^{1}H} NMR (128 MHz, $CDCl_3$) δ -2.3, -8.7, -11.9, -13.5.

HRMS (ESI): *m/z* calculated for $[C_3H_{11}O_2B_{10}]^-$ $[M-H]^-$: 187.1769; found: 187.1767.

2.2 Carboranylamination reagents 3

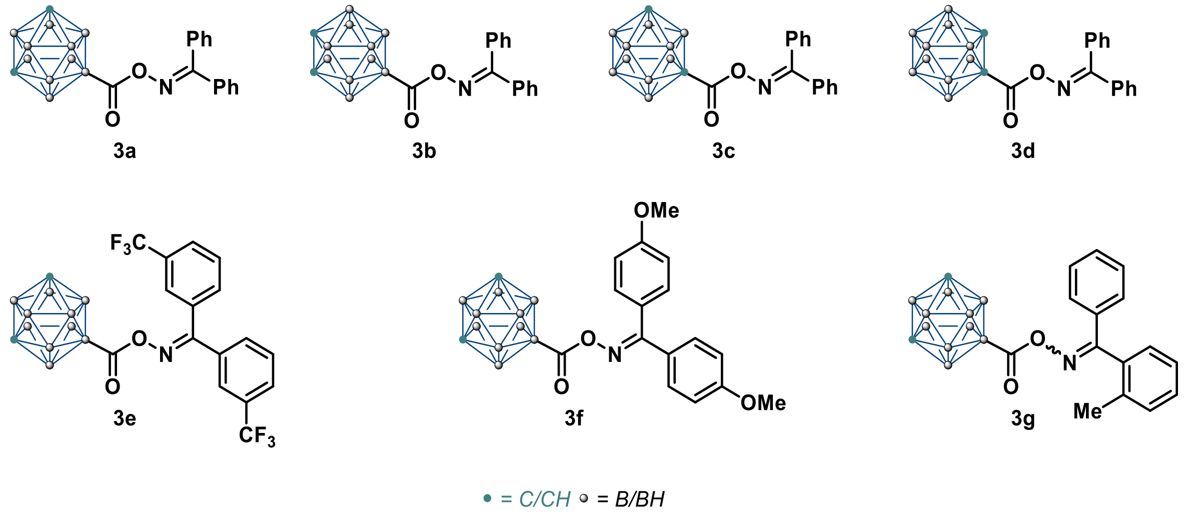

Supplementary Figure 3. Used carboranylamination reagents **3**.

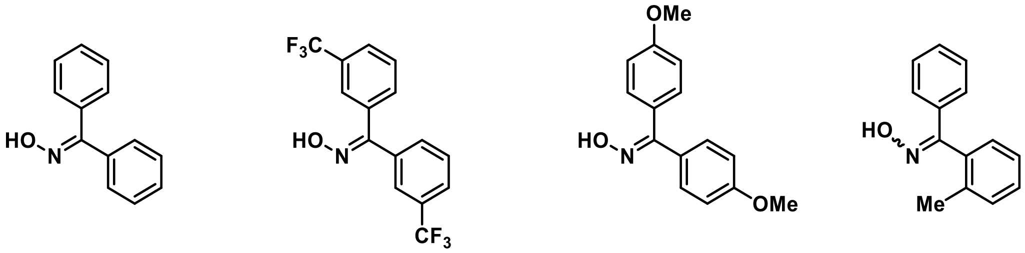

2a **2b** **2c** **2d**

Supplementary Figure 4. Used oximes **2**.

Oximes **2** were prepared according to an established procedure.[4]

General procedure GP1 for the synthesis of reagents 3 from acids 1 and oximes 2

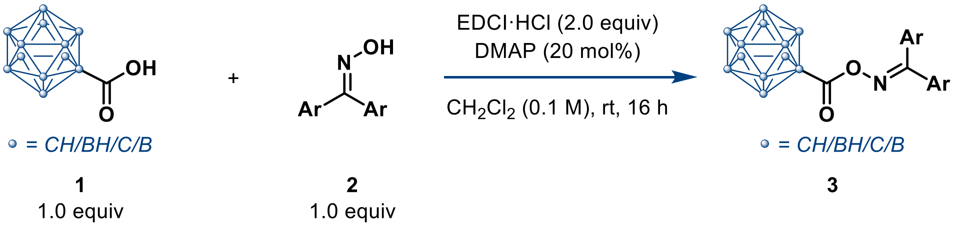

A Schlenk tube was charged with carborane carboxylic acid **1** (1.0 equiv), oxime **2** (1.0 equiv), EDCI·HCl (2.0 equiv), and a magnetic stirring bar. The tube was evacuated and backfilled with argon three times. Dry CH_2Cl_2 (0.1 M) followed by DMAP (20 mol%) was added and the resulting mixture was stirred at room temperature for 16 h. Water was added, and the mixture was transferred to a separatory funnel. After mixing well, the layers were separated and the aqueous layer was further extracted with CH_2Cl_2 three times. After combining the organic layers, drying over Na_2SO_4, and evaporation of the solvent, the residue was purified by column chromatography with the indicated solvent system.

Note 1: The product TLC spots were easily identified by the combination of using a UV lamp and $PdCl_2$ staining.

Carboranylamination reagent 3a

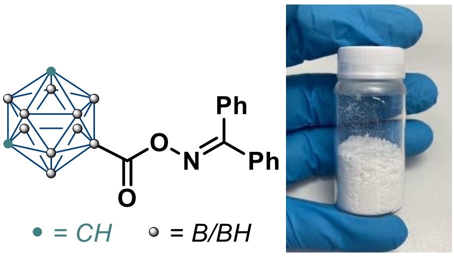

According to general procedure **GP1**, the title compound was prepared from carborane carboxylic acid **1a** (1.89 g, 10.0 mmol, 1.0 equiv), oxime **2a** (1.98 g, 10.0 mmol, 1.0 equiv), EDCI·HCl (3.83 g, 20.0 mmol, 2.0 equiv), and DMAP (244 mg, 2.00 mmol, 20 mol%) in dry CH_2Cl_2 (100 mL, 0.1 M) and was obtained as a white solid (3.31 g, 9.01 mmol, 90%) after purification by column chromatography (*n*-pentane/ethyl acetate = 8:1 to 1:1).

^{1}H NMR (400 MHz, $CDCl_3$) δ 7.64 – 7.59 (m, 2H), 7.46 – 7.40 (m, 4H), 7.38 – 7.30 (m, 4H), 2.93 (s, 2H), 3.52 – 1.41 (m, 9H).

^{13}C{^{1}H} NMR (101 MHz, $CDCl_3$) δ 165.2, 135.2, 133.2, 130.8, 129.3, 129.2, 129.1, 128.4, 128.0, 54.6. *The carbon NMR signal of the carboxylic acid group is not visible due to quadrupolar broadening.*

^{11}B{^{1}H} NMR (128 MHz, $CDCl_3$) δ -6.5, -7.9, -9.9, -13.1, -13.5, -17.2.

HRMS (ESI): *m/z* calculated for $[C_{16}H_{21}NO_2B_{10}Na]^+$ $[M+Na]^+$: 390.2476; found: 390.2460.

Note 1: The structure of this compound was confirmed by X-Ray crystal structure analysis.

*Note 2: Based on TLC analysis of the crude mixture (after extraction), the most relevant impurity for column chromatography is unconsumed oxime **2a** which elutes above the desired product. Running the column with n-pentane/ethyl acetate = 8:1 smoothly separates off oxime **2a**, after which the desired product can be quickly collected by significantly increasing the polarity. Below are shown the pictures of the TLCs with 1) UV light and 2) $PdCl_2$ staining.*

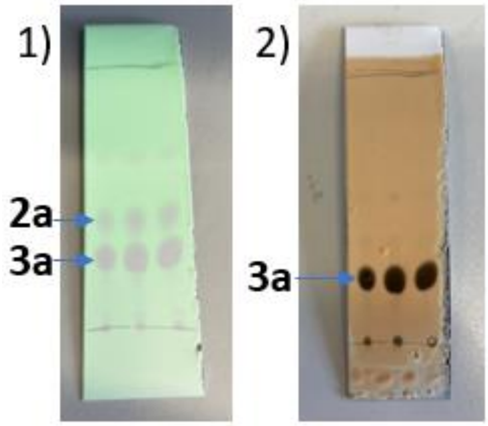

Supplementary Figure 5. TLC analysis of the reaction mixture of **3a** (after extraction) 1) under UV light and 2) after $PdCl_2$ staining.

Carboranylamination reagent 3b

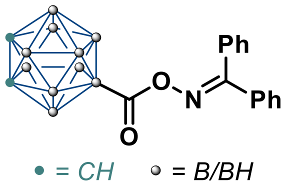

According to general procedure **GP1**, the title compound was prepared from carborane carboxylic acid **1b** (119 mg, 0.632 mmol, 1.0 equiv), oxime **2a** (125 mg, 0.634 mmol, 1.0 equiv), EDCI·HCl (242 mg, 1.26 mmol, 2.0 equiv), and DMAP (15.4 mg, 0.126 mmol, 20 mol%) in dry CH_2Cl_2 (6.3 mL, 0.1 M) and was obtained as a white solid (69.2 mg, 0.188 mmol, 30%) after purification by column chromatography (*n*-pentane/ethyl acetate = 4:1).

^{1}H NMR (400 MHz, $CDCl_3$) δ 7.62 – 7.57 (m, 2H), 7.47 – 7.39 (m, 4H), 7.38 – 7.29 (m, 4H), 3.55 (s, 2H), 3.20 – 0.73 (m, 9H).

^{13}C{^{1}H} NMR (101 MHz, $CDCl_3$) δ 165.1, 135.3, 133.2, 130.7, 129.3, 129.2, 129.2, 128.4, 128.0, 53.4, 52.9. *The carbon NMR signal of the carboxylic acid group is not visible due to quadrupolar broadening.*

^{11}B{^{1}H} NMR (128 MHz, $CDCl_3$) δ -0.9, -2.3, -9.1, -14.1, -15.4.

HRMS (ESI): *m/z* calculated for $[C_{16}H_{21}NO_2B_{10}Na]^+$ $[M+Na]^+$: 390.2476; found: 390.2464.

Carboranylamination reagent 3c

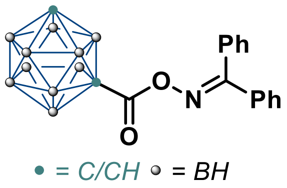

According to general procedure **GP1**, the title compound was prepared from carborane carboxylic acid **1c** (70.9 mg, 0.377 mmol, 1.0 equiv), oxime **2a** (74.4 mg, 0.377 mmol, 1.0 equiv), EDCI·HCl (145 mg, 0.756 mmol, 2.0 equiv), and DMAP (9.21 mg, 0.0754 mmol, 20 mol%) in dry CH_2Cl_2 (3.8 mL, 0.1 M) and was obtained as a white solid (74.6 mg, 0.203 mmol, 54%) after purification by column chromatography (*n*-pentane/ethyl acetate = 25:1 to 20:1).

^{1}H NMR (400 MHz, $CDCl_3$) δ 7.62 – 7.56 (m, 2H), 7.54 – 7.44 (m, 4H), 7.43 – 7.33 (m, 2H), 7.32 – 7.22 (m, 2H), 2.92 (s, 1H), 3.59 – 1.35 (m, 10H).

^{13}C{^{1}H} NMR (101 MHz, $CDCl_3$) δ 167.8, 159.0, 133.7, 132.0, 131.7, 130.1, 129.3, 128.7, 128.6, 128.5, 71.0, 54.8.

$^{11}B\{^1H\}$ **NMR** (128 MHz, $CDCl_3$) δ -5.0, -6.6, -10.7, -11.4, -13.4, -15.7.

HRMS (ESI): *m/z* calculated for $[C_{16}H_{21}NO_2B_{10}Na]^+$ $[M+Na]^+$: 390.2476; found: 390.2457.

Carboranylamination reagent 3d

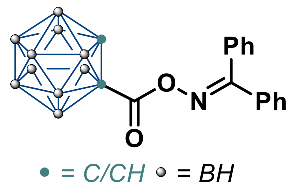

According to general procedure **GP1**, the title compound was prepared from carborane carboxylic acid **1d** (81.1 mg, 0.431 mmol, 1.0 equiv), oxime **2a** (85.0 mg, 0.431 mmol, 1.0 equiv), EDCI·HCl (165 mg, 0.861 mmol, 2.0 equiv), and DMAP (10.5 mg, 0.0859 mmol, 20 mol%) in dry CH_2Cl_2 (4.3 mL, 0.1 M) and was obtained as a white solid (49.0 mg, 0.133 mmol, 31%) after purification by column chromatography (*n*-pentane/ethyl acetate = 20:1).

^{1}**H NMR** (400 MHz, $CDCl_3$) δ 7.63 – 7.57 (m, 2H), 7.56 – 7.47 (m, 4H), 7.43 – 7.36 (m, 2H), 7.31 – 7.25 (m, 2H), 3.98 (s, 1H), 2.95 – 1.36 (m, 10H).

$^{13}C\{^1H\}$ **NMR** (101 MHz, $CDCl_3$) δ 168.7, 158.7, 133.3, 132.0, 131.6, 130.5, 129.4, 128.8, 128.6, 128.5, 67.9, 57.1.

$^{11}B\{^1H\}$ **NMR** (128 MHz, $CDCl_3$) δ -2.6, -8.8, -12.1, -13.6.

HRMS (ESI): *m/z* calculated for $[C_{16}H_{21}NO_2B_{10}Na]^+$ $[M+Na]^+$: 390.2476; found: 390.2462.

Carboranylamination reagent 3e

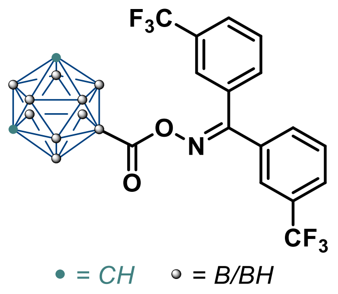

According to general procedure **GP1**, the title compound was prepared from carborane carboxylic acid **1a** (94.1 mg, 0.500 mmol, 1.0 equiv), oxime **2b** (167 mg, 0.501 mmol, 1.0 equiv), EDCI·HCl (192 mg, 1.00 mmol, 2.0 equiv), and DMAP (12.2 mg, 0.0999 mmol, 20 mol%) in dry CH_2Cl_2 (5.0 mL, 0.1 M) and was obtained as a white solid (237 mg, 0.471 mmol, 94%) after purification

by column chromatography (n-pentane/ethyl acetate = 9:1 to 4:1).

^{1}H NMR (500 MHz, $CDCl_3$) δ 7.89 – 7.87 (m, 1H), 7.80 – 7.69 (m, 4H), 7.64 – 7.59 (m, 1H), 7.55 – 7.50 (m, 1H), 7.48 – 7.44 (m, 1H), 2.96 (s, 2H), 3.37 – 1.50 (m, 9H).

^{13}C{^{1}H} NMR (126 MHz, $CDCl_3$) δ 162.0, 135.5, 133.0, 132.3 (q, J = 1.2 Hz), 132.2 (q, J = 1.0 Hz), 131.4 (q, J = 32.8 Hz), 131.0 (q, J = 32.9 Hz), 129.3, 129.1, 127.8 (q, J = 3.6 Hz), 126.7 (q, J = 3.7 Hz), 126.3 (q, J = 3.9 Hz), 125.7 (q, J = 3.8 Hz), 123.9 (q, J = 272.6 Hz), 123.8 (q, J = 272.6 Hz), 54.8. *The carbon NMR signal of the carboxylic acid group is not visible due to quadrupolar broadening.*

^{11}B{^{1}H} NMR (160 MHz, $CDCl_3$) δ -6.6, -8.3, -10.0, -13.1, -13.4, -17.1.

^{19}F{^{1}H} NMR (470 MHz, $CDCl_3$) δ -62.7, -62.7.

HRMS (ESI): m/z calculated for $[C_{18}H_{19}B_{10}F_6NO_2Na]^+$ $[M+Na]^+$: 526.2224; found: 526.2196.

Carboranylamination reagent 3f

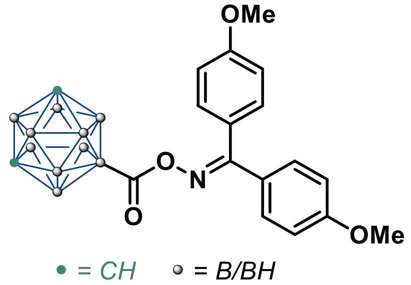

According to general procedure **GP1**, the title compound was prepared from carborane carboxylic acid **1a** (94.1 mg, 0.500 mmol, 1.0 equiv), oxime **2c** (129 mg, 0.501 mmol, 1.0 equiv), EDCI·HCl (192 mg, 1.00 mmol, 2.0 equiv), and DMAP (12.2 mg, 0.0999 mmol, 20 mol%) in dry CH_2Cl_2 (5.0 mL, 0.1 M) and was obtained as a white solid (206 mg, 0.482 mmol, 96%) after purification by column chromatography (n-pentane/ethyl acetate = 4:1 to 3:2).

^{1}H NMR (400 MHz, $CDCl_3$) δ 7.57 – 7.51 (m, 2H), 7.34 – 7.29 (m, 2H), 6.95 – 6.90 (m, 2H), 6.89 – 6.83 (m, 2H), 3.86 (s, 3H), 3.82 (s, 3H), 2.95 (s, 2H), 3.43 – 1.38 (m, 9H).

^{13}C{^{1}H} NMR (101 MHz, $CDCl_3$) δ 164.4, 161.7, 160.4, 131.3, 131.0, 128.1, 125.5, 113.7, 113.2, 55.5, 54.6. *The carbon NMR signal of the carboxylic acid group is not visible due to quadrupolar broadening.*

^{11}B{^{1}H} NMR (128 MHz, $CDCl_3$) δ -6.5, -7.8, -9.9, -13.0, -13.5, -17.2.

HRMS (ESI): m/z calculated for $[C_{18}H_{25}B_{10}NO_4Na]^+$ $[M+Na]^+$: 450.2688; found: 450.2660.

Carboranylamination reagent 3g

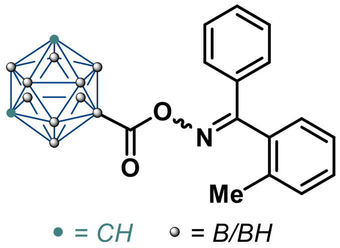

According to general procedure **GP1**, the title compound was prepared from carborane carboxylic acid **1a** (94.1 mg, 0.500 mmol, 1.0 equiv), oxime **2d** (106 mg, 0.502 mmol, 1.0 equiv), EDCI·HCl (192 mg, 1.00 mmol, 2.0 equiv), and DMAP (12.2 mg, 0.0999 mmol, 20 mol%) in dry CH_2Cl_2 (5.0 mL, 0.1 M) and was obtained as a white solid (179 mg, 0.469 mmol, 94%) after purification by column chromatography (*n*-pentane/ethyl acetate = 9:1 to 4:1).

^{1}H NMR (400 MHz, $CDCl_3$) δ 7.68 – 7.03 (m, 9H), 3.01 – 2.82 (m, 2H), 2.22 – 2.11 (m, 3H), 3.44 – 1.37 (m, 9H).

^{13}C{^{1}H} NMR (101 MHz, $CDCl_3$) δ 165.2, 165.1, 137.6, 135.5, 135.5, 134.2, 133.8, 133.5, 131.0, 130.9, 130.8, 130.0, 129.9, 129.9, 129.7, 128.8, 128.6, 128.4, 128.0, 127.3, 125.8, 125.5, 54.7, 54.6, 20.6, 19.6. *The carbon NMR signal of the carboxylic acid group is not visible due to quadrupolar broadening.*

^{11}B{^{1}H} NMR (128 MHz, $CDCl_3$) δ -6.6, -7.9, -10.0, -13.1, -13.5, -17.2.

HRMS (ESI): *m/z* calculated for $[C_{17}H_{23}B_{10}NO_2Na]^+$ $[M+Na]^+$: 404.2633; found: 404.2604.

Note: The product forms a 3:1 mixture of isomers (E/Z isomers of the imine).

2.3 Alkenes 4

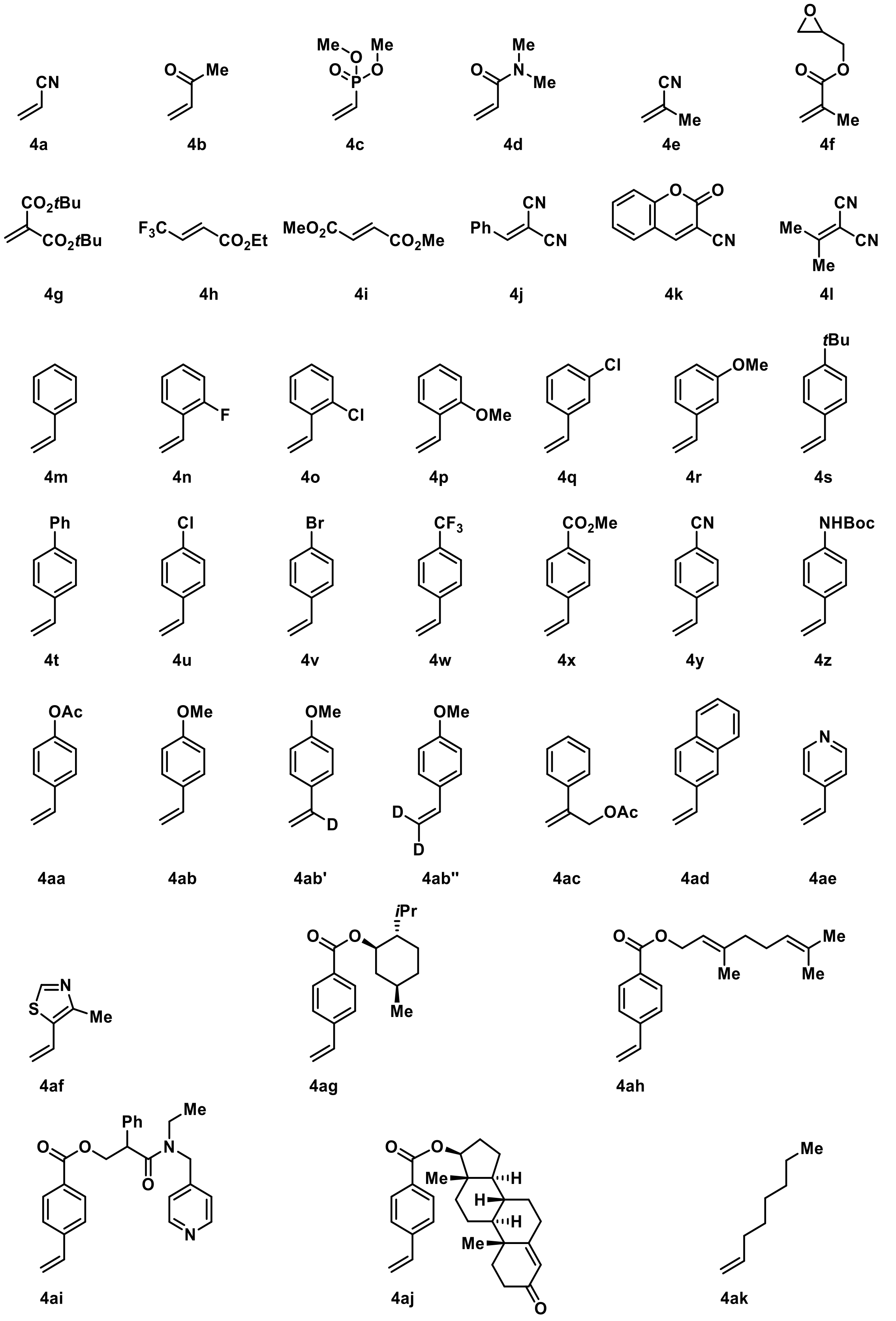

Supplementary Figure 6. Used alkenes **4**.

Commercially available alkenes were bought from commercial suppliers and used as received. Alkenes **4g**,[5] **4z**,[6] **4ab'**,[7] **4ab''**,[8] **4ac**,[9] **4ag**,[6] **4ah**,[10] and **4aj**[11] are literature known and were prepared according to literature procedures.

3-(Ethyl(pyridin-4-ylmethyl)amino)-3-oxo-2-phenylpropyl 4-vinylbenzoate (4ai)

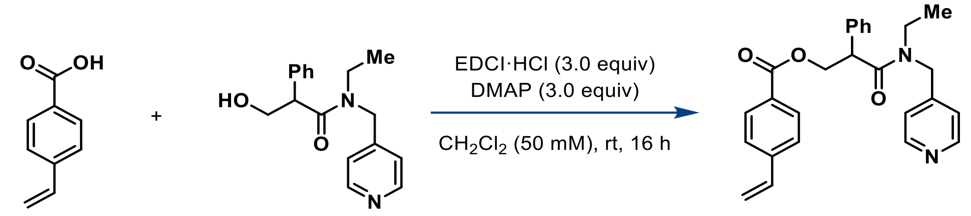

A Schlenk tube was charged with 4-vinylbenzoic acid (445 mg, 3.00 mmol, 3.0 equiv), tropicamide (284 mg, 0.999 mmol, 1.0 equiv), EDCI·HCl (575 mg, 3.00 mmol, 3.0 equiv), DMAP (367 mg, 3.00 mmol, 3.0 equiv), and a magnetic stirring bar. The Schlenk tube was evacuated and backfilled with argon three times. Dry CH_2Cl_2 (20 mL) was added, and the reaction mixture was stirred at room temperature for 16 h. Distilled water (20 mL) was added, the layers were separated, and the aqueous layer was extracted with CH_2Cl_2 (3x 10 mL). The combined organic layers were dried over Na_2SO_4 and concentrated under reduced pressure. Column chromatography (n-pentane/ethyl acetate = 1:2) gave the title compound as a sticky colorless oil (278 mg, 0.671 mmol, 67%).

^{1}H NMR (400 MHz, $CDCl_3$) δ 8.46 – 8.29 (m, 2H), 7.97 – 7.89 (m, 2H), 7.52 – 7.28 (m, 7H), 7.06 – 6.92 (m, 2H), 6.81 – 6.69 (m, 1H), 5.92 – 5.83 (m, 1H), 5.40 (d, J = 10.9 Hz, 1H), 4.98 – 4.86 (m, 1H), 4.72 – 3.99 (m, 4H), 3.79 – 3.33 (m, 1H), 3.29 – 3.10 (m, 1H), 1.14 – 0.93 (m, 3H).

^{13}C{^{1}H} NMR (101 MHz, $CDCl_3$) δ 171.1, 170.7, 166.3, 166.1, 150.2, 149.6, 147.6, 146.4, 142.3, 142.3, 136.1, 136.0, 135.5, 135.2, 130.1, 130.0, 129.4, 129.3, 129.2, 129.2, 128.4, 128.3, 128.2, 128.2, 126.3, 122.5, 121.2, 116.9, 116.8, 67.2, 66.9, 49.5, 48.7, 48.0, 42.8, 41.9, 14.3, 12.6.

HRMS (ESI): *m/z* calculated for $[C_{26}H_{26}N_2O_3Na]^+$ $[M+Na]^+$: 437.1836; found: 437.1835.

Note: The product forms a 2:1 mixture of rotamers.

3. Reaction development

3.1 Crude analysis

Crude ^{1}H and GC-MS analysis of the standard reaction towards 5a

To obtain detailed information about occurring side products and a better understanding of the reaction, we carried out ^{1}H NMR and GC-MS analysis of the crude reaction mixture of **5a**:

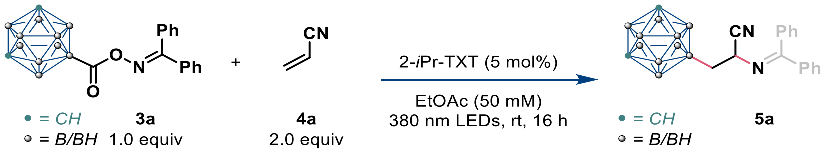

An oven-dried 10 mL Schlenk tube was charged with a Teflon-coated stirring bar, 2-isopropylthioxanthone (0.6 mg, 2 µmol, 5 mol%), and bifunctional reagent **3a** (18.4 mg, 0.0501 mmol, 1.0 equiv). The tube was evacuated and backfilled with argon three times. Dry ethyl acetate (1.0 mL, 50 mM) followed by alkene **4a** (5.3 mg, 0.10 mmol, 2.0 equiv) was added under argon counter flow. The mixture was then irradiated at 380 nm for 16 h. A sample of this mixture was examined by GC-MS. After that, the GC-MS sample was added back to the reaction mixture, the solvent was removed in vacuo, and the residue was examined by ^{1}H NMR spectroscopy with mesitylene as internal standard.

The crude ^{1}H NMR spectrum (Supplementary Figure 7) allows for easy identification and quantification of product **5a** (64% NMR yield).

The crude GC-MS data (Supplementary Figure 8) shows **5a** as the largest peak alongside imine dimer **7a**.

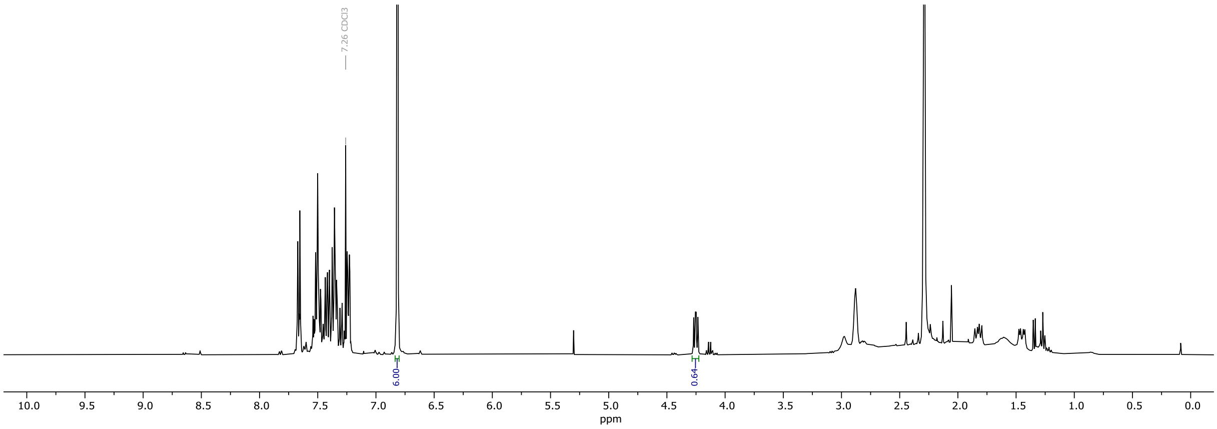

Supplementary Figure 7. Crude ^{1}H NMR spectrum of the reaction mixture of **5a**.

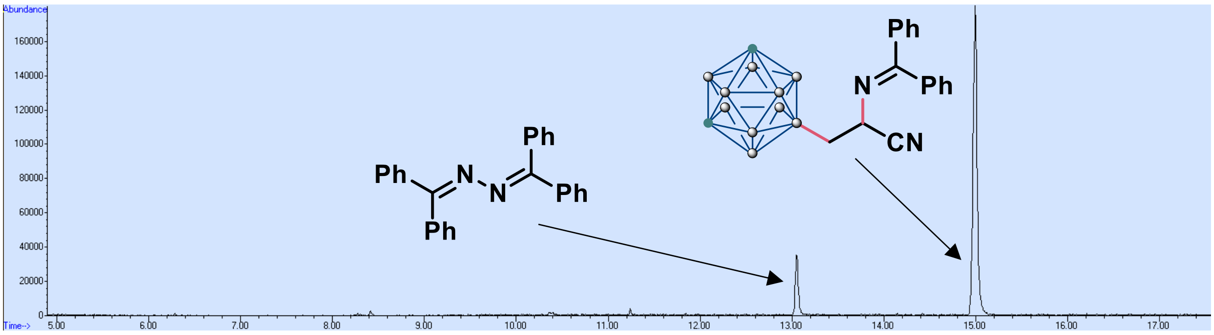

Supplementary Figure 8. GC-MS analysis of the crude reaction mixture of **5a**.

Crude HRMS analysis of the reaction towards 5a with 3.0 equiv of 4a

We were wondering why an increase in equivalents of alkene **4a** from 2.0 equiv to 3.0 equiv led to a slightly decreased yield of **5a** (see the manuscript: Figure 2, entry 6). Therefore, we analyzed this crude reaction mixture by HRMS:

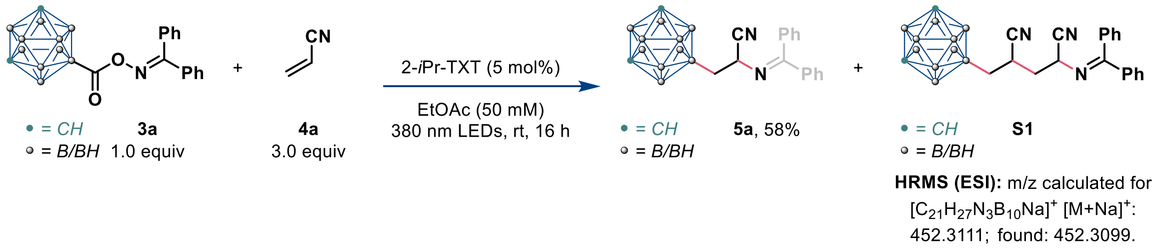

An oven-dried 10 mL Schlenk tube was charged with a Teflon-coated stirring bar, 2-isopropylthioxanthone (0.6 mg, 2 µmol, 5 mol%), and bifunctional reagent **3a** (18.4 mg, 0.0501 mmol, 1.0 equiv). The tube was evacuated and backfilled with argon three times. Dry ethyl acetate (1.0 mL, 50 mM) followed by alkene **4a** (8.0 mg, 0.15 mmol, 3.0 equiv) was added under argon counter flow. The mixture was then irradiated at 380 nm for 16 h. A sample of this mixture was examined by HRMS.

The crude HRMS analysis suggested the presence of compound **S1** formed by radical addition across two alkenes **4a** (attempts to isolate **S1** remained unsuccessful). Increasing the amount of **4a** could lead to increased formation of this side product and consequently a reduced yield of **5a**.

3.2 Sensitivity assessment

The sensitivity assessment was conducted in a similar manner as reported by Glorius and coworkers.[12]

General procedure and results

An oven-dried 10 mL Schlenk tube was charged with a Teflon-coated stirring bar, 2-isopropylthioxanthone (0.6 mg, 2 µmol, 5 mol%), and bifunctional reagent **3a** (18.4 mg, 50.1 µmol, 1.0 equiv). The tube was evacuated and backfilled with argon three times. Dry ethyl acetate (1.0 mL, 50 mM) followed by alkene **4a** (5.3 mg, 0.10 mmol, 2.0 equiv) was added under argon counter flow. The mixture was then irradiated at 380 nm for 16 h. After that, the solvent was removed in vacuo and the residue was examined by ^{1}H NMR spectroscopy with mesitylene as internal standard. The tested deviations from these conditions and the respective results are shown below.

Supplementary Table 1. Deviations from the standard reaction conditions and corresponding relative deviations in yield from the control standard reaction.

Entry	Reaction	Deviation from the standard conditions	Relative deviation in yield from the standard reaction / %[a]
1	High *c*	0.9 mL EtOAc	-3
2	Low *c*	1.1 mL EtOAc	3
3	High H_2O	+5 µL H_2O	-2
4	Low O_2	Degassing by 3x freeze-pump-thaw	-2
5	High O_2	Reaction prepared under air	-70
6	Low *T*	Water-cooled Schlenk tube	-2[b]
7	High *T*	Standard setup w/o fans turned on	-3
8	Low *I*	One lamp 32 cm away from reaction vessel	-8
9	High *I*	One lamp 2 cm away from reaction vessel	0

[a] Determined by ^{1}H NMR spectroscopy with mesitylene as internal standard.

[b] Deviation from a control reaction in a water-cooled Schlenk tube w/o water flow.

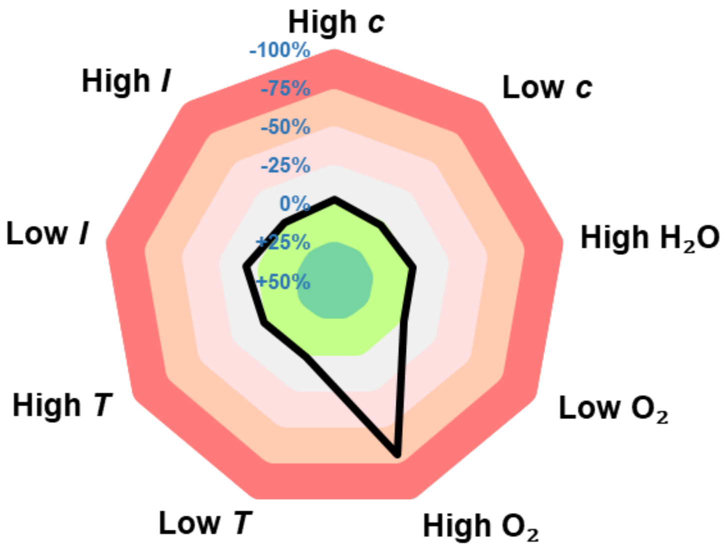

Supplementary Figure 9. Sensitivity assessment radar diagram.

3.3 Optimization of the benzophenone imine moiety of 3

We investigated the effect of electron-withdrawing, electron-donating, and sterically hindering substituents at the imine moiety of **3** trying to improve the reagent's performance in the 1,2-carboranylimination reaction (Supplementary Figure 10).

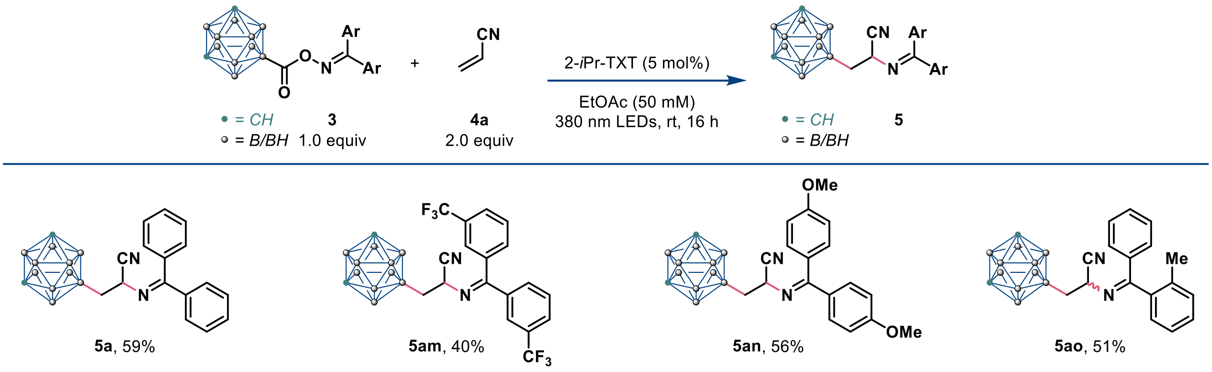

Supplementary Figure 10. Optimization of the reagent's benzophenone imine moiety. Reactions were performed on 0.1 mmol scale. Isolated yields are given. For the experimental procedures and characterization data, see chapter 4.

While the overall tolerance of the introduced functional groups was good, no improvement over **3a** was achieved.

4. Substrate scope

General procedure GP2 for the synthesis of carboranylaminated products 5

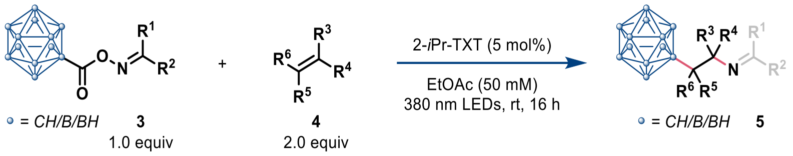

An oven-dried 10 mL Schlenk tube was charged with a Teflon-coated stirring bar, 2-isopropylthioxanthone (1.3 mg, 5.1 µmol, 5 mol%), and bifunctional reagent **3** (0.10 mmol, 1.0 equiv). The tube was evacuated and backfilled with argon three times. Dry ethyl acetate (2.0 mL, 50 mM) followed by alkene **4** (0.20 mmol, 2.0 equiv) was added under argon counter flow. The mixture was then irradiated at 380 nm for 16 h. After that, the solvent was removed and the product was isolated by column chromatography on deactivated silica using the specified solvent system.

Note 1: Solid alkenes were added to the tube before replacing the atmosphere with argon.

Note 2: Products from Michael acceptors typically showed signs of decomposition (hydrolysis of the imine moiety) after a few hours. Products from styrenes were typically more stable.

4.1 Characterization data

Carboranylaminated product 5a

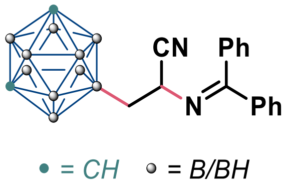

The title compound was prepared according to general procedure **GP2** from bifunctional reagent **3a** (36.7 mg, 99.9 µmol, 1.0 equiv) and alkene **4a** (10.6 mg, 0.200 mmol, 2.0 equiv). Column chromatography (*n*-pentane/ethyl acetate = 50:1 to 25:1) gave the title compound as a white solid (22.3 mg, 59.2 µmol, 59%).

^{1}H NMR (400 MHz, $CDCl_3$) δ 7.67 – 7.62 (m, 2H), 7.54 – 7.47 (m, 3H), 7.46 – 7.39 (m, 1H), 7.39 – 7.31 (m, 2H), 7.25 – 7.19 (m, 2H), 4.24 (dd, J = 8.7, 5.7 Hz, 1H), 2.88 (s, 2H), 1.81 (dd, J = 15.0, 8.8 Hz, 1H), 1.44 (dd, J = 14.9, 5.8 Hz, 1H), 3.42 – 1.33 (m, 9H).

^{13}C{^{1}H} NMR (101 MHz, $CDCl_3$) δ 171.6, 138.9, 135.4, 131.0, 129.3, 129.2, 129.0, 128.3, 127.6, 121.1, 54.7, 53.6, 22.9.

$^{11}B\{^1H\}$ NMR (128 MHz, $CDCl_3$) δ -2.1, -6.4, -10.0, -13.2, -13.8, -17.5, -19.5.

HRMS (ESI): *m/z* calculated for $[C_{18}H_{24}N_2B_{10}Na]^+$ $[M+Na]^+$: 399.2844; found: 399.2831.

Carboranylaminated product 5b

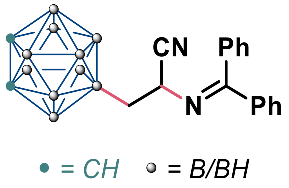

The title compound was prepared according to general procedure **GP2** from bifunctional reagent **3b** (36.7 mg, 99.9 µmol, 1.0 equiv) and alkene **4a** (10.6 mg, 0.200 mmol, 2.0 equiv). Column chromatography (*n*-pentane/ethyl acetate = 19:1 to 10:1) gave the title compound as a white solid (21.5 mg, 57.1 µmol, 57%).

^1H NMR (400 MHz, $CDCl_3$) δ 7.68 – 7.60 (m, 2H), 7.54 – 7.47 (m, 3H), 7.44 – 7.39 (m, 1H), 7.38 – 7.30 (m, 2H), 7.24 – 7.17 (m, 2H), 4.14 (dd, *J* = 8.9, 5.6 Hz, 1H), 3.53 (s, 1H), 3.46 (s, 1H), 1.68 (dd, *J* = 14.9, 9.0 Hz, 1H), 1.27 (dd, *J* = 14.9, 4.6 Hz, 1H), 3.04 – 1.07 (m, 9H).

$^{13}C\{^1H\}$ NMR (101 MHz, $CDCl_3$) δ 171.4, 138.9, 135.4, 131.0, 129.3, 129.2, 129.0, 128.3, 127.6, 121.2, 53.8, 53.3, 49.9, 24.9.

$^{11}B\{^1H\}$ NMR (128 MHz, $CDCl_3$) δ 5.5, -2.0, -8.9, -14.0, -15.3.

HRMS (ESI): *m/z* calculated for $[C_{18}H_{24}N_2B_{10}H]^+$ $[M+H]^+$: 377.3024; found: 377.3008.

Carboranylaminated product 5c

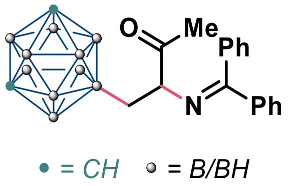

The title compound was prepared according to general procedure **GP2** on double the normal scale from bifunctional reagent **3a** (73.4 mg, 0.200 mmol, 1.0 equiv), alkene **4b** (28.0 mg, 0.399 mmol, 2.0 equiv), and 2-isopropylthioxanthone (2.6 mg, 10 µmol, 5 mol%) in dry ethyl acetate (4.0 mL, 50 mM). Column chromatography (*n*-pentane/ethyl acetate = 15:1) gave the title compound as a sticky colorless oil (35.6 mg, 90.5 µmol, 45%).

^1H NMR (400 MHz, $CDCl_3$) δ 7.70 – 7.64 (m, 2H), 7.48 – 7.30 (m, 6H), 7.16 – 7.11 (m, 2H), 4.04 (t,

J = 6.7 Hz, 1H), 2.82 (s, 2H), 2.20 (s, 3H), 1.62 (dd, *J* = 14.9, 6.6 Hz, 1H), 1.51 – 1.37 (m, 1H), 3.32 – 1.19 (m, 9H).

^{13}C{^{1}H} NMR (101 MHz, $CDCl_3$) *δ* 209.4, 168.9, 139.8, 136.6, 130.3, 129.0, 128.7, 128.6, 128.1, 127.9, 73.3, 54.4, 26.8, 20.8.

^{11}B{^{1}H} NMR (128 MHz, $CDCl_3$) *δ* -0.9, -6.4, -9.9, -13.2, -14.0, -17.7, -20.0.

HRMS (ESI): *m/z* calculated for $[C_{19}H_{27}NOB_{10}H]^+$ $[M+H]^+$: 394.3178; found: 394.3165.

Carboranylaminated product 5d

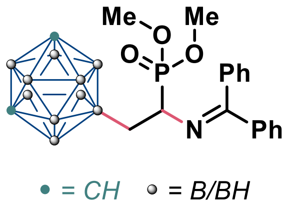

The title compound was prepared according to general procedure **GP2** from bifunctional reagent **3a** (36.7 mg, 99.9 µmol, 1.0 equiv) and alkene **4c** (27.2 mg, 0.200 mmol, 2.0 equiv). Column chromatography (*n*-pentane/ethyl acetate = 2:1 to 1:1) gave the title compound as a white sticky solid (20.6 mg, 44.8 µmol, 45%).

^{1}H NMR (400 MHz, $CDCl_3$) *δ* 7.65 (d, *J* = 7.5 Hz, 2H), 7.55 – 7.48 (m, 2H), 7.46 – 7.41 (m, 3H), 7.41 – 7.29 (m, 3H), 4.26 – 4.16 (m, 1H), 3.83 – 3.71 (m, 6H), 2.77 (s, 2H), 1.90 – 1.62 (m, 2H), 3.28 – 1.06 (m, 9H).

^{13}C{^{1}H} NMR (101 MHz, $CDCl_3$) *δ* 169.7, 140.3, 135.6, 130.2, 129.6, 129.5, 129.0, 128.1, 128.0, 61.1 (d, *J* = 153.6 Hz), 54.5, 54.3, 53.7 (d, *J* = 5.1 Hz), 53.3 (d, *J* = 7.1 Hz), 18.4.

^{11}B{^{1}H} NMR (128 MHz, $CDCl_3$) *δ* -0.6, -6.4, -10.1, -13.3, -14.0, -17.7, -20.1.

^{31}P NMR (162 MHz, $CDCl_3$) *δ* 28.4.

HRMS (ESI): *m/z* calculated for $[C_{19}H_{30}NO_3PB_{10}Na]^+$ $[M+Na]^+$: 482.2868; found: 482.2855.

Amine hydrochloride 5e

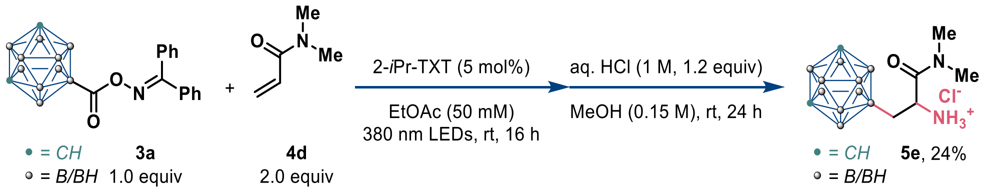

The title compound was prepared according to general procedure **GP2** from bifunctional reagent **3a** (36.7 mg, 99.9 µmol, 1.0 equiv) and alkene **4d** (19.8 mg, 0.200 mmol, 2.0 equiv). Preliminary purification was done with column chromatography (*n*-pentane/ethyl acetate = 8:1 to 3/2). Due to insufficient purity, we decided to isolate the product as the deprotected amine hydrochloride:

To a 20 mL vial was added a magnetic stirring bar, the obtained crude product, methanol (0.67 mL, 0.15 M), and aq. HCl (1.0 M; 120 µL, 0.120 mmol, 1.2 equiv). The mixture was stirred at room temperature for 24 h, and then, the solvent was removed under reduced pressure. Aq. HCl (1.0 M, 2 mL) was added, and the aqueous layer was washed with *n*-pentane (3 x 5 mL). The aqueous layer was subsequently evaporated, and drying under vacuum gave the desired product as a light-orange solid (7.0 mg, 23.7 µmol, 24%).

^{1}H NMR (400 MHz, D_2O) δ 4.47 (dd, J = 7.9, 5.8 Hz, 1H), 3.49 (s, 2H), 3.17 (s, 3H), 3.00 (s, 3H), 1.67 (dd, J = 15.7, 5.7 Hz, 1H), 1.47 (dd, J = 15.7, 7.9 Hz, 1H), 3.23 – 1.43 (m, 9H).

^{13}C{^{1}H} NMR (101 MHz, D_2O) δ 170.2, 55.8, 50.9, 37.3, 35.8, 17.5.

^{11}B{^{1}H} NMR (128 MHz, D_2O) δ -3.2, -6.8, -10.6, -13.1, -13.6, -16.9, -19.1.

HRMS (ESI): *m/z* calculated for $[C_7H_{22}B_{10}N_2ONa]^+$ $[M\text{-}HCl+Na]^+$: 281.2631; found: 281.2627.

Carboranylaminated product 5f

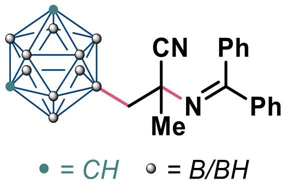

The title compound was prepared according to general procedure **GP2** from bifunctional reagent **3a** (36.7 mg, 99.9 µmol, 1.0 equiv) and alkene **4e** (13.4 mg, 0.200 mmol, 2.0 equiv). Column chromatography (*n*-pentane/ethyl acetate = 40:1 to 25:1) gave the title compound as a white solid (24.4 mg, 62.5 µmol, 62%).

^{1}H NMR (400 MHz, $CDCl_3$) δ 7.67 – 7.60 (m, 2H), 7.56 – 7.45 (m, 3H), 7.42 – 7.37 (m, 1H), 7.36 – 7.26 (m, 4H), 2.89 (s, 2H), 1.91 (d, J = 14.9 Hz, 1H), 1.80 – 1.69 (m, 4H), 3.44 – 0.97 (m, 9H).

^{13}C{^{1}H} NMR (101 MHz, $CDCl_3$) δ 167.1, 140.1, 135.5, 130.5, 129.5, 128.9, 128.6, 128.2, 128.1, 121.5, 57.1, 54.6, 32.8, 31.9.

^{11}B{^{1}H} NMR (128 MHz, $CDCl_3$) δ -2.2, -6.1, -9.5, -13.0, -13.9, -17.6, -19.4.

HRMS (ESI): *m/z* calculated for $[C_{19}H_{26}B_{10}N_2H]^+$ $[M+H]^+$: 391.3181; found: 391.3165.

Carboranylaminated product 5g

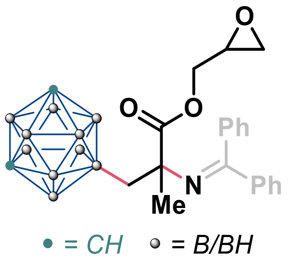

The title compound was prepared according to general procedure **GP2** from bifunctional reagent **3a** (36.7 mg, 99.9 µmol, 1.0 equiv) and alkene **4f** (28.4 mg, 0.200 mmol, 2.0 equiv). Column chromatography (*n*-pentane/ethyl acetate = 15:1 to 8:1) gave the title compound as a white solid (20.5 mg, 44.0 µmol, 44%) in 51:49 d.r.

^{1}H NMR (400 MHz, $CDCl_3$) δ 7.62 – 7.56 (m, 2H), 7.41 – 7.26 (m, 6H), 7.22 – 7.16 (m, 2H), 3.90 – 3.80 (m, 1H), 3.59 – 3.43 (m, 1H), 3.11 – 3.03 (m, 1H), 2.87 (s, 2H), 2.80 – 2.74 (m, 1H), 2.59 – 2.52 (m, 1H), 1.99 – 1.89 (m, 1H), 1.76 – 1.67 (m, 1H), 1.62 – 1.55 (m, 3H), 3.40 – 1.41 (m, 9H).

^{13}C{^{1}H} NMR (101 MHz, $CDCl_3$) δ 175.5, 175.5, 165.4, 165.4, 141.2, 137.4, 129.8, 128.9, 128.8, 128.7, 128.4, 127.9, 127.8, 66.8, 66.7, 65.3, 65.0, 54.4, 49.2, 49.2, 44.9, 44.8, 30.4, 26.6, 26.3.

^{11}B{^{1}H} NMR (128 MHz, $CDCl_3$) δ -1.5, -6.1, -9.5, -12.9, -14.0, -17.6, -19.6.

HRMS (ESI): *m/z* calculated for $[C_{22}H_{31}B_{10}NO_3Na]^+$ $[M+Na]^+$: 488.3210; found: 488.3199.

Carboranylaminated product 5h

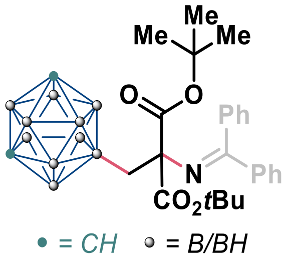

The title compound was prepared according to general procedure **GP2** from bifunctional reagent **3a** (36.7 mg, 99.9 µmol, 1.0 equiv) and alkene **4g** (45.7 mg, 0.200 mmol, 2.0 equiv). Column chromatography (*n*-pentane/ethyl acetate = 50:1 to 30:1) gave the title compound as a white solid (39.4 mg, 71.4 µmol, 71%).

^{1}H NMR (400 MHz, $CDCl_3$) δ 7.69 – 7.61 (m, 2H), 7.40 – 7.23 (m, 8H), 2.79 (s, 2H), 1.96 (s, 2H), 1.38 (s, 18H), 3.29 – 1.05 (m, 9H).

$^{13}C\{^1H\}$ **NMR** (101 MHz, $CDCl_3$) δ 170.1, 165.3, 141.7, 137.1, 129.7, 129.2, 129.0, 128.5, 127.9, 127.7, 81.7, 75.6, 54.3, 28.1.

$^{11}B\{^1H\}$ **NMR** (128 MHz, $CDCl_3$) δ -1.6, -6.1, -9.5, -13.0, -14.2, -17.7, -19.7.

HRMS (ESI): *m/z* calculated for $[C_{27}H_{41}NO_4B_{10}Na]^+$ $[M+Na]^+$: 574.3944; found: 574.3933.

Carboranylaminated product 5i

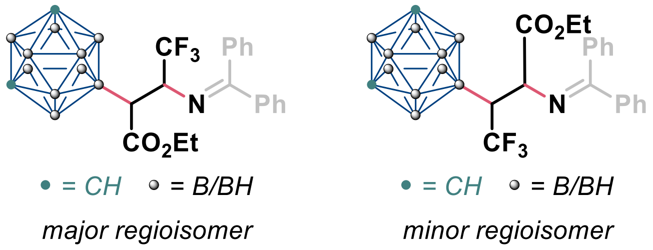

The title compound was prepared according to general procedure **GP2** from bifunctional reagent **3a** (36.7 mg, 99.9 µmol, 1.0 equiv) and alkene **4h** (33.6 mg, 0.200 mmol, 2.0 equiv). Column chromatography (*n*-pentane/ethyl acetate = 50:1 to 20:1) gave the title compound as two separable regioisomers:

Major regioisomer: white sticky solid (26.7 mg, 54.3 µmol, 54%), 87:13 d.r., NMR signals of the major diastereomer are reported:

^1H **NMR** (400 MHz, $CDCl_3$) δ 7.64 – 7.59 (m, 2H), 7.49 – 7.42 (m, 3H), 7.42 – 7.35 (m, 3H), 7.34 – 7.27 (m, 2H), 4.33 (dq, *J* = 8.2, 6.2 Hz, 1H), 4.14 – 3.95 (m, 2H), 3.19 (d, *J* = 8.6 Hz, 1H), 2.85 (s, 2H), 1.18 (t, *J* = 7.2 Hz, 3H), 3.37 – 0.92 (m, 9H).

$^{13}C\{^1H\}$ **NMR** (101 MHz, $CDCl_3$) δ 173.4, 172.4, 139.5, 135.9, 130.6, 129.2, 128.7, 128.4, 128.3, 128.1, 125.61 (q, *J* = 281.2 Hz), 65.2 (q, *J* = 27.0 Hz), 60.3, 54.5, 54.3, 37.3, 14.4.

$^{11}B\{^1H\}$ **NMR** (128 MHz, $CDCl_3$) δ -1.9, -6.4, -9.9, -13.2, -13.9, -17.6, -19.0.

$^{19}F\{^1H\}$ **NMR** (376 MHz, $CDCl_3$) δ -70.8.

HRMS (ESI): *m/z* calculated for $[C_{21}H_{28}NO_2B_{10}F_3Na]^+$ $[M+Na]^+$: 514.2978; found: 514.2966.

Minor regioisomer: white sticky solid (5.0 mg, 10 µmol, 10%), >95:5 d.r.

^1H **NMR** (400 MHz, $CDCl_3$) δ 7.70 – 7.64 (m, 2H), 7.44 – 7.36 (m, 4H), 7.35 – 7.30 (m, 2H), 7.29 – 7.23 (m, 2H), 4.35 (d, *J* = 2.7 Hz, 1H), 4.31 – 4.13 (m, 2H), 3.09 (q, *J* = 10.9 Hz, 1H), 2.86 (s, 2H), 1.28 (t, *J* = 7.1 Hz, 3H), 3.44 – 0.98 (m, 9H).

$^{13}C\{^1H\}$ **NMR** (101 MHz, $CDCl_3$) δ 171.9, 171.2, 140.3, 136.1, 130.4, 129.6, 129.0, 128.6 (q, J = 281.0 Hz), 128.5, 128.2, 128.1, 65.7 (q, J = 2.0 Hz), 61.8, 54.7, 54.5, 36.8, 14.2.

$^{11}B\{^1H\}$ **NMR** (128 MHz, $CDCl_3$) δ -2.9, -6.4, -10.1, -13.1, -13.8, -17.4, -18.7.

$^{19}F\{^1H\}$ **NMR** (376 MHz, $CDCl_3$) δ -57.0.

HRMS (ESI): *m/z* calculated for $[C_{21}H_{28}NO_2B_{10}F_3Na]^+$ $[M+Na]^+$: 514.2978; found: 514.2966.

Carboranylaminated product 5j

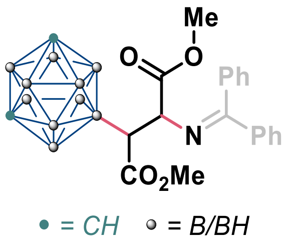

The title compound was prepared according to general procedure **GP2** from bifunctional reagent **3a** (36.7 mg, 99.9 µmol, 1.0 equiv) and alkene **4i** (28.8 mg, 0.200 mmol, 2.0 equiv). Column chromatography (*n*-pentane/ethyl acetate = 15:1 to 8:1) gave the title compound as a white solid (24.4 mg, 52.2 µmol, 52%) in >95:5 d.r.

^1H **NMR** (400 MHz, $CDCl_3$) δ 7.55 (d, J = 7.6 Hz, 2H), 7.52 – 7.34 (m, 6H), 7.30 (t, J = 7.5 Hz, 2H), 4.56 (d, J = 10.7 Hz, 1H), 3.70 (s, 3H), 3.63 – 3.53 (m, 4H), 2.85 (s, 2H), 3.41 – 1.16 (m, 9H).

$^{13}C\{^1H\}$ **NMR** (101 MHz, $CDCl_3$) δ 174.6, 172.0, 170.6, 139.9, 135.9, 130.6, 129.4, 129.1, 128.9, 128.1, 128.0, 68.0, 54.5, 52.2, 51.2, 39.2.

$^{11}B\{^1H\}$ **NMR** (128 MHz, $CDCl_3$) δ -2.3, -6.7, -10.2, -13.3, -13.8, -17.6, -19.1.

HRMS (ESI): *m/z* calculated for $[C_{21}H_{29}NO_4B_{10}Na]^+$ $[M+Na]^+$: 490.3013; found: 490.2992.

Note: The relative configuration of the major diastereomer could not be confirmed by experimental means. However, based on literature precedent about selectivity in radical difunctionalizations of acyclic acrylates,[13,14] the major diastereomer can most likely be described by the following structure:

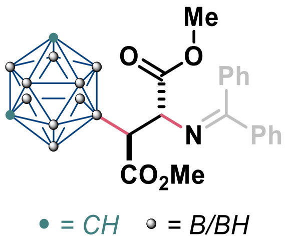

Carboranylaminated product 5k

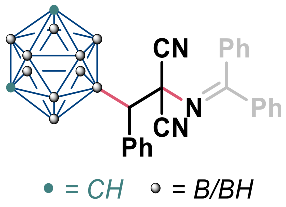

The title compound was prepared according to general procedure **GP2** from bifunctional reagent **3a** (36.7 mg, 99.9 µmol, 1.0 equiv) and alkene **4j** (30.8 mg, 0.200 mmol, 2.0 equiv). Column chromatography (*n*-pentane/ethyl acetate = 30:1 to 20:1) gave the title compound as a white solid (35.5 mg, 74.3 µmol, 74%).

^{1}H NMR (400 MHz, $CDCl_3$) δ 7.81 (d, *J* = 7.8 Hz, 1H), 7.66 (d, *J* = 7.7 Hz, 2H), 7.60 – 7.45 (m, 4H), 7.42 – 7.30 (m, 6H), 7.28 – 7.20 (m, 2H), 3.58 (s, 1H), 2.92 (s, 2H), 3.63 – 1.33 (m, 9H).

^{13}C{^{1}H} NMR (101 MHz, $CDCl_3$) δ 176.0, 140.8, 139.0, 134.1, 132.0, 131.4, 130.5, 129.8, 128.7, 128.7, 128.6, 128.5, 128.4, 128.4, 127.3, 115.0, 114.6, 57.4, 54.6, 54.6, 48.0.

^{11}B{^{1}H} NMR (128 MHz, $CDCl_3$) δ -1.4, -6.1, -9.5, -12.9, -13.6, -17.6, -18.3.

HRMS (ESI): *m/z* calculated for $[C_{25}H_{27}N_3B_{10}Na]^+$ $[M+Na]^+$: 500.3112; found: 500.3100.

Carboranylaminated product 5l

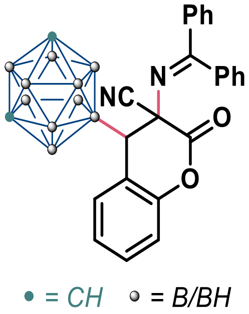

The title compound was prepared according to general procedure **GP2** from bifunctional reagent **3a** (36.7 mg, 99.9 µmol, 1.0 equiv) and alkene **4k** (34.2 mg, 0.200 mmol, 2.0 equiv). Column chromatography (*n*-pentane/ethyl acetate = 20:1 to 8:1) gave the title compound as a white solid (27.7 mg, 56.0 µmol, 56%) in >95:5 d.r.

^{1}H NMR (400 MHz, $CDCl_3$) δ 7.60 – 7.46 (m, 3H), 7.38 – 7.28 (m, 3H), 7.27 – 7.09 (m, 7H), 7.00 – 6.90 (m, 1H), 3.68 (s, 1H), 2.91 (s, 1H), 2.87 (s, 1H), 3.29 – 1.30 (m, 9H).

^{13}C{^{1}H} NMR (101 MHz, $CDCl_3$) δ 174.6, 161.4, 149.7, 139.5, 134.4, 131.5, 130.4, 129.4, 129.0, 128.5, 128.3, 128.1, 127.7, 127.5, 124.7, 116.6, 116.1, 64.1, 55.0, 54.9, 39.7.

^{11}**B{^{1}H} NMR** (128 MHz, $CDCl_3$) δ -2.7, -6.4, -9.8, -13.5, -17.2, -18.4.

HRMS (ESI): *m/z* calculated for $[C_{25}H_{26}N_2O_2B_{10}Na]^+$ $[M+Na]^+$: 517.2902; found: 517.2891.

Note: The relative configuration of the major diastereomer could not be confirmed by experimental means. However, based on literature precedent about the radical 1,2-difunctionalization of cyclic alkenes using benzophenone imine-based bifunctional reagents,[15,16] the major diastereomer can most likely be described by the following structure:

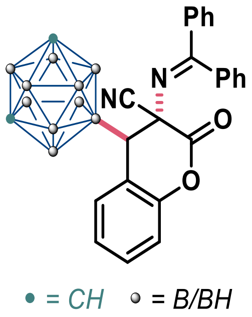

Carboranylaminated product 5m

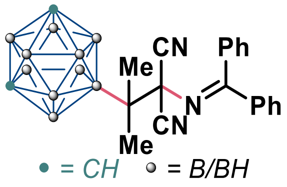

The title compound was prepared according to general procedure **GP2** from bifunctional reagent **3a** (36.7 mg, 99.9 µmol, 1.0 equiv) and alkene **4l** (21.2 mg, 0.200 mmol, 2.0 equiv). Column chromatography (*n*-pentane/ethyl acetate = 50:1 to 20:1) gave the title compound as a sticky white solid (22.7 mg, 52.8 µmol, 53%).

^{1}**H NMR** (400 MHz, $CDCl_3$) δ 7.73 (d, *J* = 7.9 Hz, 2H), 7.59 – 7.53 (m, 3H), 7.52 – 7.43 (m, 3H), 7.42 – 7.35 (m, 2H), 2.91 (s, 2H), 1.62 – 1.42 (m, 6H), 3.38 – 0.78 (m, 9H).

^{13}**C{^{1}H} NMR** (101 MHz, $CDCl_3$) δ 175.7, 139.0, 134.1, 131.9, 130.3, 129.9, 128.6, 128.5, 128.3, 114.3, 63.4, 54.4, 28.0, 27.3.

^{11}**B{^{1}H} NMR** (128 MHz, $CDCl_3$) δ 1.0, -6.6, -10.1, -13.4, -13.9, -17.6, -18.6.

HRMS (ESI): *m/z* calculated for $[C_{21}H_{27}N_3B_{10}Na]^+$ $[M+Na]^+$: 452.3111; found: 452.3099.

Amine hydrochloride 5n

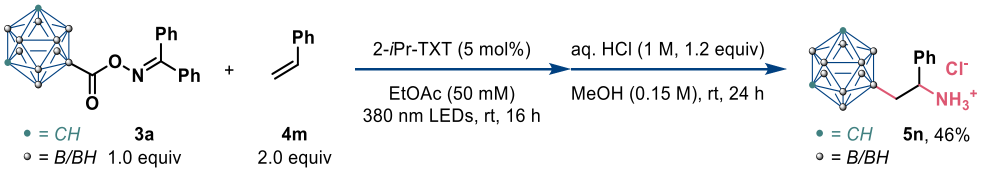

An oven-dried 10 mL Schlenk tube was charged with a Teflon-coated stirring bar, 2-*i*Pr-TXT (3.9 mg, 15 µmol, 5 mol%), and bifunctional reagent **3a** (110 mg, 0.299 mmol, 1.0 equiv). The tube was evacuated and backfilled with argon three times. Dry ethyl acetate (6.0 mL, 50 mM) followed by alkene **4m** (62.6 mg, 0.601 mmol, 2.0 equiv) was added under argon counter flow. The mixture was then irradiated at 380 nm for 16 h. After that, the solvent was removed, and the product was purified by column chromatography (*n*-pentane/ethyl acetate = 100:1 to 50:1). Since imine dimer **7a** could not be removed, we decided to isolate the product as the deprotected amine hydrochloride:

To a 20 mL vial was added a magnetic stirring bar, the obtained crude product, methanol (2.0 mL, 0.15 M), and aq. HCl (1.0 M; 350 µL, 0.350 mmol, 1.2 equiv). The mixture was stirred at room temperature for 24 h, and then, the solvent was removed under reduced pressure. Aq. HCl (1.0 M, 2 mL) was added, and the aqueous layer was washed with *n*-pentane (3 x 5 mL). The aqueous layer was subsequently evaporated, redissolved in CH_2Cl_2 (5 mL), filtered, evaporated, and dried under vacuum. The desired product was obtained as a white solid (41.4 mg, 0.138 mmol, 46%).

^{1}H NMR (400 MHz, $CDCl_3$) δ 8.43 (s, 3H), 7.52 – 7.42 (m, 2H), 7.33 – 7.25 (m, 3H), 4.39 – 3.81 (m, 1H), 2.73 (s, 2H), 1.89 – 1.78 (m, 1H), 1.68 (dd, J = 14.6, 3.2 Hz, 1H), 3.23 – 0.99 (m, 9H).

^{13}C{^{1}H} NMR (101 MHz, $CDCl_3$) δ 136.5, 129.1, 129.0, 128.7, 57.0, 54.4, 54.3, 20.2.

^{11}B{^{1}H} NMR (128 MHz, $CDCl_3$) δ -1.7, -6.5, -10.1, -13.3, -14.0, -17.7, -19.9.

HRMS (ESI): *m/z* calculated for $[C_{10}H_{22}NB_{10}]^+$ $[M\text{-}Cl^-]^+$: 264.2755; found: 264.2749.

Amine hydrochloride 5o

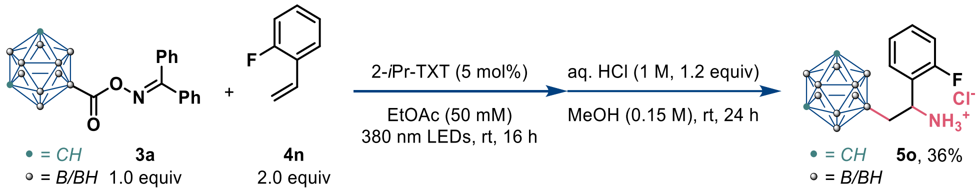

The title compound was prepared according to general procedure **GP2** from bifunctional reagent **3a** (36.7 mg, 99.9 µmol, 1.0 equiv) and alkene **4n** (24.4 mg, 0.200 mmol, 2.0 equiv). Preliminary

purification was done with column chromatography (*n*-pentane/ethyl acetate = 400:1 to 300:1). Since imine dimer **7a** could not be removed, we decided to isolate the product as the deprotected amine hydrochloride:

To a 20 mL vial was added a magnetic stirring bar, the obtained crude product, methanol (0.67 mL, 0.15 M), and aq. HCl (1.0 M; 120 µL, 0.120 mmol, 1.2 equiv). The mixture was stirred at room temperature for 24 h, and then, the solvent was removed under reduced pressure. Aq. HCl (1.0 M, 2 mL) was added, and the aqueous layer was washed with *n*-pentane (3 x 5 mL). The aqueous layer was subsequently filtered and the filter material was rinsed with aq. HCl (1.0 M, 2 mL). Evaporation and drying under vacuum gave the desired product as a white solid (11.3 mg, 35.6 µmol, 36%).

^{1}H NMR (400 MHz, D_2O) δ 7.60 (td, *J* = 7.6, 1.8 Hz, 1H), 7.48 (dddd, *J* = 8.4, 7.3, 5.4, 1.7 Hz, 1H), 7.29 (td, *J* = 7.6, 1.2 Hz, 1H), 7.22 (ddd, *J* = 11.0, 8.4, 1.2 Hz, 1H), 4.79 – 4.71 (m, 1H), 3.26 (s, 2H), 1.99 (t, *J* = 13.7 Hz, 1H), 1.68 (dd, *J* = 14.5, 3.5 Hz, 1H), 3.04 – 1.04 (m, 9H).

^{13}C{^{1}H} NMR (101 MHz, D_2O) δ 160.9 (d, *J* = 246.0 Hz), 131.5 (d, *J* = 8.9 Hz), 129.4 (d, *J* = 3.2 Hz), 124.9 (d, *J* = 3.5 Hz), 123.3 (d, *J* = 13.2 Hz), 115.9 (d, *J* = 22.0 Hz), 55.3, 55.3, 49.2 (d, *J* = 4.1 Hz), 18.4.

^{11}B{^{1}H} NMR (128 MHz, D_2O) δ -2.9, -6.9, -10.7, -13.4, -14.0, -17.4, -19.4.

^{19}F NMR (376 MHz, D_2O) δ -117.7 (dt, *J* = 12.0, 6.2 Hz).

HRMS (ESI): *m/z* calculated for $[C_{10}H_{21}NB_{10}F]^{+}$ $[M\text{-}Cl^{-}]^{+}$: 282.2661; found: 282.2655.

Note: This compound's structure was further confirmed by X-Ray crystal analysis.

Amine hydrochloride 5p

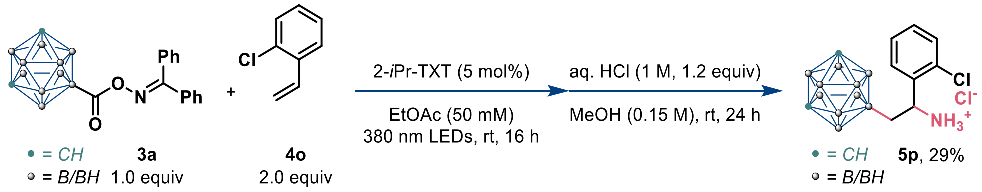

The title compound was prepared according to general procedure **GP2** from bifunctional reagent **3a** (36.7 mg, 99.9 µmol, 1.0 equiv) and alkene **4o** (27.7 mg, 0.200 mmol, 2.0 equiv). Preliminary purification was done with column chromatography (*n*-pentane/ethyl acetate = 300:1). Since imine dimer **7a** could not be removed, we decided to isolate the product as the deprotected amine hydrochloride:

To a 20 mL vial was added a magnetic stirring bar, the obtained crude product, methanol (0.67 mL, 0.15 M), and aq. HCl (1.0 M; 120 µL, 0.120 mmol, 1.2 equiv). The mixture was stirred at room temperature for 24 h, and then, the solvent was removed under reduced pressure. Aq. HCl (1.0 M, 2 mL) was added, and the aqueous layer was washed with *n*-pentane (3 x 5 mL). The aqueous layer was subsequently filtered and the filter material was rinsed with aq. HCl (1.0 M, 2 mL). Evaporation of the combined aqueous layers and drying under vacuum gave the desired product as a white solid (9.7 mg, 29 µmol, 29%).

^{1}H NMR (400 MHz, D_2O) δ 7.73 – 7.64 (m, 1H), 7.59 – 7.50 (m, 1H), 7.48 – 7.40 (m, 2H), 4.99 (dd, *J* = 12.7, 3.6 Hz, 1H), 3.26 (s, 2H), 2.04 (t, *J* = 13.7 Hz, 1H), 1.70 (dd, *J* = 14.6, 3.6 Hz, 1H), 2.95 – 1.13 (m, 9H).

^{13}C{^{1}H} NMR (101 MHz, D_2O) δ 134.5, 133.4, 130.9, 130.0, 128.9, 127.7, 55.4, 55.3, 51.7, 18.5.

^{11}B{^{1}H} NMR (128 MHz, D_2O) δ -2.9, -6.9, -10.6, -13.3, -14.0, -17.3, -19.4.

HRMS (ESI): *m/z* calculated for $[C_{10}H_{21}NB_{10}Cl]^+$ $[M-Cl^-]^+$: 297.2402; found: 297.2395.

Carboranylaminated product 5q

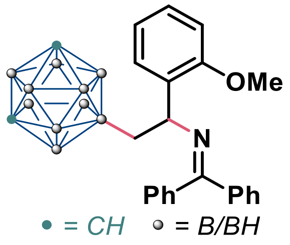

The title compound was prepared according to general procedure **GP2** on three times the normal scale from bifunctional reagent **3a** (110 mg, 0.299 mmol, 1.0 equiv) and alkene **4p** (80.5 mg, 0.600 mmol, 2.0 equiv). Column chromatography (*n*-pentane/CH_2Cl_2/ethyl acetate = 8:2:0.05) gave the title compound as a white sticky solid (58.0 mg, 0.127 mmol, 42%).

^{1}H NMR (400 MHz, $CDCl_3$) δ 7.69 – 7.63 (m, 2H), 7.56 (d, *J* = 7.4 Hz, 1H), 7.41 – 7.28 (m, 6H), 7.21 – 7.12 (m, 1H), 7.09 – 7.02 (m, 2H), 6.93 (t, *J* = 7.5 Hz, 1H), 6.76 (dd, *J* = 8.2, 1.2 Hz, 1H), 4.95 (t, *J* = 6.8 Hz, 1H), 3.61 (s, 3H), 2.75 (s, 2H), 1.82 – 1.73 (m, 1H), 1.59 (dd, *J* = 14.1, 6.5 Hz, 1H), 3.40 – 1.13 (m, 9H).

^{13}C{^{1}H} NMR (101 MHz, $CDCl_3$) δ 165.9, 156.4, 140.8, 137.7, 135.7, 129.5, 128.8, 128.2, 128.0, 128.0, 127.8, 127.0, 120.6, 110.4, 59.5, 55.2, 54.2, 25.7.

^{11}B{^{1}H} NMR (128 MHz, $CDCl_3$) δ -0.2, -6.4, -9.9, -13.3, -14.2, -17.8, -20.2.

HRMS (ESI): *m/z* calculated for $[C_{24}H_{31}NOB_{10}Na]^+$ $[M+Na]^+$: 480.3313; found: 480.3302.

Carboranylaminated product 5r

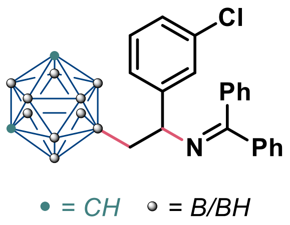

The title compound was prepared according to general procedure **GP2** from bifunctional reagent **3a** (36.7 mg, 99.9 µmol, 1.0 equiv) and alkene **4q** (27.7 mg, 0.200 mmol, 2.0 equiv). Column chromatography (*n*-pentane/ethyl acetate = 300:1) gave the title compound as a sticky white solid (20.6 mg, 44.6 µmol, 45%).

^{1}H NMR (400 MHz, $CDCl_3$) δ 7.67 – 7.62 (m, 2H), 7.46 – 7.26 (m, 7H), 7.22 – 7.11 (m, 3H), 7.07 (dd, *J* = 6.3, 3.0 Hz, 2H), 4.46 (t, *J* = 6.9 Hz, 1H), 2.76 (s, 2H), 1.72 (dd, *J* = 15.2, 7.2 Hz, 1H), 1.65 – 1.55 (m, 1H), 3.32 – 0.98 (m, 9H).

^{13}C{^{1}H} NMR (101 MHz, $CDCl_3$) δ 165.9, 148.6, 140.2, 137.2, 133.9, 129.9, 129.4, 128.8, 128.4, 128.4, 128.1, 128.0, 128.0, 126.6, 126.1, 66.5, 54.3, 27.1.

^{11}B{^{1}H} NMR (128 MHz, $CDCl_3$) δ -0.8, -6.4, -10.0, -13.3, -14.1, -17.7, -20.0.

HRMS (ESI): *m/z* calculated for $[C_{23}H_{28}B_{10}ClNNa]^+$ $[M+Na]^+$: 484.2811; found: 484.2809.

Carboranylaminated product 5s

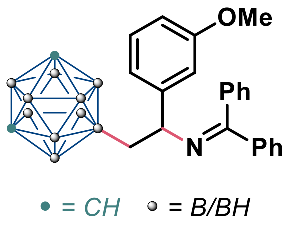

The title compound was prepared according to general procedure **GP2** from bifunctional reagent **3a** (36.7 mg, 99.9 µmol, 1.0 equiv) and alkene **4r** (26.8 mg, 0.200 mmol, 2.0 equiv). Column chromatography (*n*-pentane/ethyl acetate = 75:1 to 50:1) gave the title compound as a white solid (20.7 mg, 45.2 µmol, 45%).

^{1}H NMR (400 MHz, $CDCl_3$) δ 7.67 – 7.62 (m, 2H), 7.44 – 7.39 (m, 3H), 7.38 – 7.27 (m, 3H), 7.17 (t, *J* = 7.9 Hz, 1H), 7.12 – 7.07 (m, 2H), 6.90 – 6.81 (m, 2H), 6.77 – 6.70 (m, 1H), 4.47 (t, *J* = 6.8 Hz, 1H), 3.79 (s, 3H), 2.75 (s, 2H), 1.76 (dd, *J* = 15.1, 7.3 Hz, 1H), 1.61 (dd, *J* = 14.9, 6.1 Hz, 1H), 3.39 – 1.08 (m, 9H).

$^{13}C\{^1H\}$ **NMR** (101 MHz, $CDCl_3$) δ 165.3, 159.5, 148.2, 140.5, 137.4, 129.7, 129.0, 128.8, 128.3, 128.2, 128.1, 128.0, 120.4, 113.4, 112.0, 67.0, 55.4, 54.3, 27.1.

$^{11}B\{^1H\}$ **NMR** (128 MHz, $CDCl_3$) δ -0.6, -6.4, -9.9, -13.3, -14.1, -17.7, -20.1.

HRMS (ESI): *m/z* calculated for $[C_{24}H_{31}NOB_{10}H]^+$ $[M+H]^+$: 458.3493; found: 458.3481.

Carboranylaminated product 5t

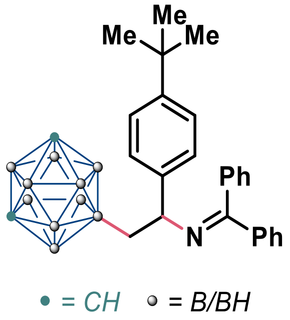

The title compound was prepared according to general procedure **GP2** from bifunctional reagent **3a** (36.7 mg, 99.9 µmol, 1.0 equiv) and alkene **4s** (32.1 mg, 0.200 mmol, 2.0 equiv). Column chromatography (*n*-pentane/ethyl acetate = 500:1 to 300:1) gave the title compound as a white solid (24.0 mg, 49.6 µmol, 50%).

^1H **NMR** (400 MHz, $CDCl_3$) δ 7.67 – 7.61 (m, 2H), 7.45 – 7.38 (m, 3H), 7.37 – 7.23 (m, 5H), 7.22 – 7.16 (m, 2H), 7.13 – 7.06 (m, 2H), 4.48 (t, *J* = 6.8 Hz, 1H), 2.74 (s, 2H), 1.74 (dd, *J* = 14.8, 6.9 Hz, 1H), 1.63 (dd, *J* = 14.7, 5.7 Hz, 1H), 1.30 (s, 9H), 3.29 – 1.04 (m, 9H).

$^{13}C\{^1H\}$ **NMR** (101 MHz, $CDCl_3$) δ 164.9, 149.1, 143.5, 140.6, 137.5, 129.6, 128.8, 128.2, 128.2, 128.0, 127.3, 125.0, 66.6, 54.2, 34.5, 31.6, 27.1.

$^{11}B\{^1H\}$ **NMR** (128 MHz, $CDCl_3$) δ -0.4, -6.4, -9.9, -13.3, -14.2, -17.8, -20.1.

HRMS (ESI): *m/z* calculated for $[C_{27}H_{37}NB_{10}Na]^+$ $[M+Na]^+$: 506.3834; found: 506.3822.

Carboranylaminated product 5u

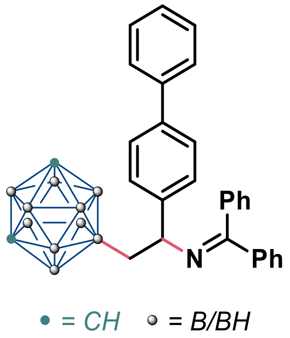

The title compound was prepared according to general procedure **GP2** from bifunctional reagent **3a** (36.7 mg, 99.9 µmol, 1.0 equiv) and alkene **4t** (36.1 mg, 0.200 mmol, 2.0 equiv). Column chromatography (*n*-pentane/ethyl acetate = 100:1 to 50:1) gave the title compound as a white solid (25.9 mg, 51.4 µmol, 51%).

^{1}H NMR (400 MHz, $CDCl_3$) δ 7.71 – 7.64 (m, 2H), 7.64 – 7.59 (m, 2H), 7.54 – 7.48 (m, 2H), 7.47 – 7.39 (m, 5H), 7.38 – 7.28 (m, 6H), 7.16 – 7.05 (m, 2H), 4.55 (t, *J* = 6.9 Hz, 1H), 2.75 (s, 2H), 1.81 (dd, *J* = 15.0, 7.3 Hz, 1H), 1.66 (dd, *J* = 15.0, 6.3 Hz, 1H), 3.26 – 1.12 (m, 9H).

^{13}C{^{1}H} NMR (101 MHz, $CDCl_3$) δ 165.3, 145.7, 141.4, 140.5, 139.2, 137.5, 129.7, 128.8, 128.8, 128.3, 128.3, 128.3, 128.1, 128.0, 127.1, 127.1, 126.9, 66.7, 54.3, 27.3.

^{11}B{^{1}H} NMR (128 MHz, $CDCl_3$) δ -0.6, -6.4, -9.9, -13.3, -14.1, -17.8, -20.1.

HRMS (ESI): *m/z* calculated for $[C_{29}H_{33}B_{10}NNa]^+$ $[M+Na]^+$: 526.3522; found: 526.3509.

Carboranylaminated product 5v

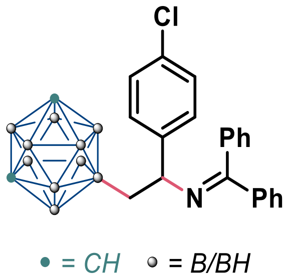

The title compound was prepared according to general procedure **GP2** from bifunctional reagent **3a** (36.7 mg, 99.9 µmol, 1.0 equiv) and alkene **4u** (27.7 mg, 0.200 mmol, 2.0 equiv). Column chromatography (*n*-pentane/ethyl acetate = 300:1 to 200:1) gave the title compound as a white solid (23.2 mg, 50.2 µmol, 50%).

^{1}H NMR (400 MHz, $CDCl_3$) δ 7.65 – 7.59 (m, 2H), 7.45 – 7.40 (m, 3H), 7.38 – 7.27 (m, 3H), 7.25 –

7.16 (m, 4H), 7.09 – 7.04 (m, 2H), 4.47 (dd, *J* = 7.7, 6.1 Hz, 1H), 2.75 (s, 2H), 1.73 (dd, *J* = 15.0, 7.7 Hz, 1H), 1.62 – 1.52 (m, 1H), 3.16 – 1.14 (m, 9H).

^{13}C{^{1}H} NMR (101 MHz, $CDCl_3$) δ 165.6, 144.8, 140.3, 137.3, 132.0, 129.8, 129.3, 128.8, 128.4, 128.4, 128.3, 128.1, 128.0, 66.4, 54.3, 26.8.

^{11}B{^{1}H} NMR (128 MHz, $CDCl_3$) δ -0.8, -6.5, -10.0, -13.3, -14.1, -17.8, -20.1.

HRMS (ESI): *m/z* calculated for $[C_{23}H_{28}NB_{10}ClH]^+$ $[M+H]^+$: 462.3000; found: 462.2989.

Carboranylaminated product 5w

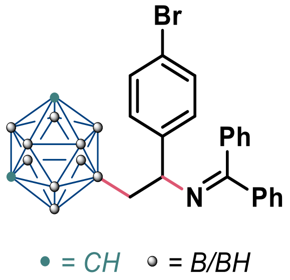

The title compound was prepared according to general procedure **GP2** from bifunctional reagent **3a** (36.7 mg, 99.9 µmol, 1.0 equiv) and alkene **4v** (36.2 mg, 0.198 mmol, 2.0 equiv). Column chromatography (*n*-pentane/ethyl acetate = 400:1) gave the title compound as a white solid (23.9 mg, 47.2 µmol, 47%).

^{1}H NMR (400 MHz, $CDCl_3$) δ 7.68 – 7.57 (m, 2H), 7.48 – 7.27 (m, 8H), 7.20 – 7.11 (m, 2H), 7.11 – 7.02 (m, 2H), 4.46 (t, *J* = 6.3 Hz, 1H), 2.75 (s, 2H), 1.73 (dd, *J* = 14.4, 5.5 Hz, 1H), 1.65 – 1.53 (m, 1H), 3.27 – 1.12 (m, 9H).

^{13}C{^{1}H} NMR (101 MHz, $CDCl_3$) δ 165.6, 145.4, 140.3, 137.3, 131.3, 129.9, 129.7, 128.8, 128.4, 128.4, 128.1, 128.0, 120.2, 66.4, 54.3, 26.9.

^{11}B{^{1}H} NMR (128 MHz, $CDCl_3$) δ -0.8, -6.5, -10.0, -13.3, -14.1, -17.8, -20.1.

HRMS (ESI): *m/z* calculated for $[C_{23}H_{28}NB_{10}BrNa]^+$ $[M+Na]^+$: 529.2298; found: 529.2289.

Carboranylaminated product 5x

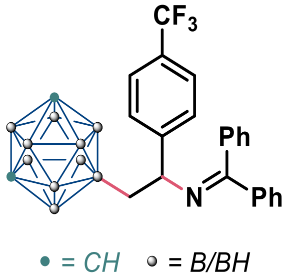

The title compound was prepared according to general procedure **GP2** from bifunctional reagent **3a** (36.7 mg, 99.9 µmol, 1.0 equiv) and alkene **4w** (34.4 mg, 0.200 mmol, 2.0 equiv). Column chromatography (*n*-pentane/ethyl acetate = 400:1) gave the title compound as a white solid (22.7 mg, 45.8 µmol, 46%).

^{1}H NMR (400 MHz, $CDCl_3$) δ 7.70 – 7.60 (m, 2H), 7.54 – 7.49 (m, 2H), 7.48 – 7.28 (m, 8H), 7.13 – 6.99 (m, 2H), 4.55 (t, J = 6.4 Hz, 1H), 2.76 (s, 2H), 1.74 (dd, J = 15.1, 5.0 Hz, 1H), 1.67 – 1.54 (m, 1H), 3.33 – 1.06 (m, 9H).

^{13}C{^{1}H} NMR (101 MHz, $CDCl_3$) δ 166.0, 150.6, 140.2, 137.2, 130.0, 128.8, 128.5, 128.4, 128.2, 128.1, 127.9, 125.2 (q, J = 3.8 Hz), 124.6 (q, J = 272.0 Hz), 66.7, 54.4, 26.9. *Due to signal broadening, not all expected C–F couplings were clearly visible.*

^{11}B{^{1}H} NMR (128 MHz, $CDCl_3$) δ -0.9, -6.5, -10.0, -13.3, -14.1, -17.7, -20.0.

^{19}F{^{1}H} NMR (377 MHz, $CDCl_3$) δ -62.2.

HRMS (ESI): *m/z* calculated for $[C_{24}H_{28}NB_{10}F_3H]^+$ $[M+H]^+$: 496.3261; found: 496.3252.

Carboranylaminated product 5y

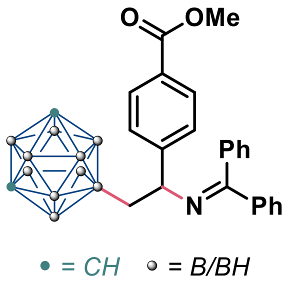

The title compound was prepared according to general procedure **GP2** from bifunctional reagent **3a** (36.7 mg, 99.9 µmol, 1.0 equiv) and alkene **4x** (32.0 mg, 0.197 mmol, 2.0 equiv). Column chromatography (*n*-pentane/CH_2Cl_2/ethyl acetate = 8:2:0.1 to 8:2:0.2) gave the title compound as a white solid (25.8 mg, 53.1 µmol, 53%).

^{1}**H NMR** (400 MHz, $CDCl_3$) δ 7.96 – 7.89 (m, 2H), 7.67 – 7.60 (m, 2H), 7.45 – 7.39 (m, 3H), 7.38 – 7.28 (m, 5H), 7.08 – 7.01 (m, 2H), 4.53 (t, J = 6.9 Hz, 1H), 3.89 (s, 3H), 2.75 (s, 2H), 1.74 (dd, J = 15.1, 7.2 Hz, 1H), 1.67 – 1.56 (m, 1H), 3.30 – 1.09 (m, 9H).

^{13}**C{^{1}H} NMR** (101 MHz, $CDCl_3$) δ 167.4, 166.0, 151.9, 140.2, 137.3, 129.9, 129.6, 128.8, 128.4, 128.4, 128.3, 128.1, 127.9, 127.9, 66.8, 54.3, 52.1, 26.6.

^{11}**B{^{1}H} NMR** (128 MHz, $CDCl_3$) δ -0.8, -6.5, -10.0, -13.3, -14.1, -17.7, -20.0.

HRMS (ESI): *m/z* calculated for $[C_{25}H_{31}NO_2B_{10}H]^+$ $[M+H]^+$: 486.3443; found: 486.3430.

Carboranylaminated product 5z

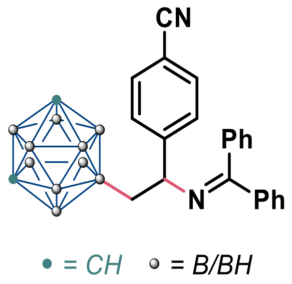

The title compound was prepared according to general procedure **GP2** from bifunctional reagent **3a** (36.7 mg, 99.9 µmol, 1.0 equiv) and alkene **4y** (25.8 mg, 0.200 mmol, 2.0 equiv). Column chromatography (*n*-pentane/ethyl acetate = 40:1 to 15:1) gave the title compound as a white solid (28.7 mg, 63.4 µmol, 63%).

^{1}**H NMR** (400 MHz, $CDCl_3$) δ 7.66 – 7.61 (m, 2H), 7.58 – 7.52 (m, 2H), 7.46 – 7.41 (m, 3H), 7.40 – 7.29 (m, 5H), 7.08 – 6.98 (m, 2H), 4.52 (t, J = 6.9 Hz, 1H), 2.77 (s, 2H), 1.71 (dd, J = 15.4, 7.2 Hz, 1H), 1.65 – 1.56 (m, 1H), 3.29 – 1.03 (m, 9H).

^{13}**C{^{1}H} NMR** (101 MHz, $CDCl_3$) δ 166.4, 152.0, 140.0, 137.1, 132.1, 130.1, 128.8, 128.7, 128.6, 128.5, 128.1, 127.8, 119.4, 110.2, 66.7, 54.4, 26.7.

^{11}**B{^{1}H} NMR** (128 MHz, $CDCl_3$) δ -1.1, -6.5, -10.1, -13.3, -14.1, -17.7, -20.0.

HRMS (ESI): *m/z* calculated for $[C_{24}H_{28}N_2B_{10}Na]^+$ $[M+Na]^+$: 475.3159; found: 475.3144.

Carboranylaminated product 5aa

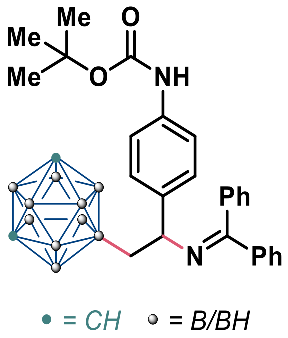

The title compound was prepared according to general procedure **GP2** from bifunctional reagent **3a** (36.7 mg, 99.9 μmol, 1.0 equiv) and alkene **4z** (43.9 mg, 0.200 mmol, 2.0 equiv). Column chromatography (*n*-pentane/ethyl acetate = 30:1 to 15:1) gave the title compound as a white solid (15.9 mg, 29.3 μmol, 29%).

^{1}H NMR (400 MHz, $CDCl_3$) *δ* 7.66 – 7.59 (m, 2H), 7.48 – 7.38 (m, 3H), 7.35 – 7.17 (m, 7H), 7.12 – 7.06 (m, 2H), 6.47 – 6.34 (m, 1H), 4.52 – 4.37 (m, 1H), 2.74 (s, 2H), 1.81 – 1.70 (m, 1H), 1.64 – 1.54 (m, 1H), 1.53 – 1.49 (m, 9H), 3.19 – 0.98 (m, 9H).

^{13}C{^{1}H} NMR (101 MHz, $CDCl_3$) *δ* 165.0, 152.9, 141.1, 140.5, 137.5, 136.7, 129.6, 128.8, 128.6, 128.3, 128.1, 128.1, 128.1, 118.3, 80.4, 66.6, 54.3, 28.5, 26.9.

^{11}B{^{1}H} NMR (128 MHz, $CDCl_3$) *δ* -0.4, -6.4, -9.9, -13.3, -14.1, -17.8, -20.1.

HRMS (ESI): *m/z* calculated for $[C_{28}H_{38}N_2O_2B_{10}H]^+$ $[M+H]^+$: 543.4023; found: 543.4045.

Carboranylaminated product 5ab

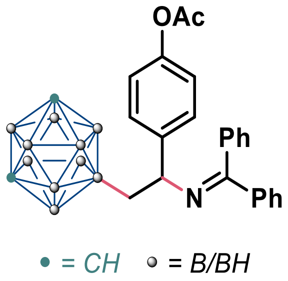

The title compound was prepared according to general procedure **GP2** from bifunctional reagent **3a** (36.7 mg, 99.9 μmol, 1.0 equiv) and alkene **4aa** (32.0 mg, 0.197 mmol, 2.0 equiv). Column chromatography (*n*-pentane/ethyl acetate = 50:1 to 20:1) gave the title compound as a white solid (23.1 mg, 47.6 μmol, 48%).

^{1}H NMR (400 MHz, $CDCl_3$) *δ* 7.67 – 7.60 (m, 2H), 7.46 – 7.39 (m, 3H), 7.37 – 7.24 (m, 5H), 7.11 –

7.05 (m, 2H), 7.01 – 6.95 (m, 2H), 4.50 (t, *J* = 6.9 Hz, 1H), 2.75 (s, 2H), 2.27 (s, 3H), 1.75 (dd, *J* = 15.0, 7.5 Hz, 1H), 1.61 (dd, *J* = 14.3, 5.8 Hz, 1H), 3.29 – 1.08 (m, 9H).

^{13}C{^{1}H} NMR (101 MHz, $CDCl_3$) δ 169.6, 165.4, 149.3, 143.9, 140.2, 137.3, 129.8, 128.9, 128.8, 128.4, 128.3, 128.1, 128.1, 121.1, 66.4, 54.3, 26.8, 21.3.

^{11}B{^{1}H} NMR (128 MHz, $CDCl_3$) δ -0.7, -6.5, -10.0, -13.3, -14.1, -17.8, -20.1.

HRMS (ESI): *m/z* calculated for $[C_{25}H_{31}NO_2B_{10}Na]^+$ $[M+Na]^+$: 508.3262; found: 508.3248.

Carboranylaminated product 5ac

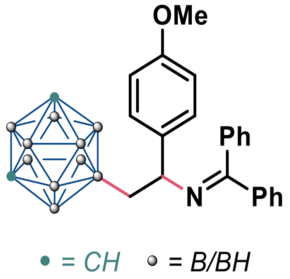

The title compound was prepared according to general procedure **GP2** from bifunctional reagent **3a** (36.7 mg, 99.9 µmol, 1.0 equiv) and alkene **4ab** (26.7 mg, 0.199 mmol, 2.0 equiv). Column chromatography (*n*-pentane/ethyl acetate = 50:1 to 30:1) gave the title compound as a white solid (19.7 mg, 43.0 µmol, 43%).

^{1}H NMR (400 MHz, $CDCl_3$) δ 7.66 – 7.60 (m, 2H), 7.45 – 7.39 (m, 3H), 7.36 – 7.26 (m, 3H), 7.22 – 7.14 (m, 2H), 7.13 – 7.06 (m, 2H), 6.84 – 6.76 (m, 2H), 4.46 (dd, *J* = 7.9, 5.9 Hz, 1H), 3.79 (s, 3H), 2.73 (s, 2H), 1.77 (dd, *J* = 14.9, 8.0 Hz, 1H), 1.56 (dd, *J* = 15.0, 5.8 Hz, 1H), 3.24 – 1.16 (m, 9H).

^{13}C{^{1}H} NMR (101 MHz, $CDCl_3$) δ 164.7, 158.3, 140.5, 138.5, 137.5, 129.6, 128.9, 128.8, 128.3, 128.2, 128.1, 128.0, 113.5, 66.4, 55.3, 54.2, 26.8.

^{11}B{^{1}H} NMR (128 MHz, $CDCl_3$) δ -0.7, -6.5, -10.0, -13.4, -14.2, -17.8, -20.2.

HRMS (ESI): *m/z* calculated for $[C_{24}H_{31}NOB_{10}H]^+$ $[M+H]^+$: 458.3493; found: 458.3481.

Carboranylaminated product 5ac'

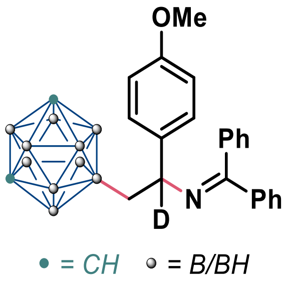

The title compound was prepared according to general procedure **GP2** from bifunctional reagent **3a** (36.7 mg, 99.9 µmol, 1.0 equiv) and alkene **4ab'** (27.0 mg, 0.200 mmol, 2.0 equiv). Column chromatography (*n*-pentane/CH_2Cl_2/ethyl acetate = 8:2:0.05 to 8:2:0.1) gave the title compound as a white solid (21.7 mg, 47.3 µmol, 47%).

^{1}H NMR (400 MHz, $CDCl_3$) δ 7.67 – 7.60 (m, 2H), 7.46 – 7.40 (m, 3H), 7.37 – 7.26 (m, 3H), 7.23 – 7.16 (m, 2H), 7.14 – 7.07 (m, 2H), 6.84 – 6.77 (m, 2H), 3.85 – 3.71 (m, 3H), 2.74 (s, 2H), 1.77 (d, *J* = 15.1 Hz, 1H), 1.55 (d, *J* = 15.0 Hz, 1H), 3.21 – 1.15 (m, 9H).

^{13}C{^{1}H} NMR (101 MHz, $CDCl_3$) δ 164.7, 158.3, 140.5, 138.4, 137.5, 129.6, 128.9, 128.8, 128.3, 128.2, 128.1, 128.0, 113.5, 66.0 (t, *J* = 19.6 Hz), 55.3, 54.2, 26.5.

^{11}B{^{1}H} NMR (128 MHz, $CDCl_3$) δ -0.6, -6.4, -10.0, -13.3, -14.2, -17.8, -20.1.

HRMS (ESI): *m/z* calculated for $[C_{24}H_{30}NOB_{10}DH]^+$ $[M+H]^+$: 459.3556; found: 459.3546.

*The benzylic proton from undeuterated **5ac** was not observed in the ^{1}H NMR spectrum of **5ac'**.*

Carboranylaminated product 5ac''

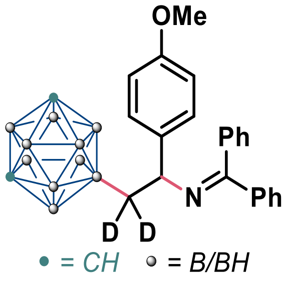

The title compound was prepared according to general procedure **GP2** from bifunctional reagent **3a** (36.7 mg, 99.9 µmol, 1.0 equiv) and alkene **4ab''** (27.2 mg, 0.200 mmol, 2.0 equiv). Column chromatography (*n*-pentane/CH_2Cl_2/ethyl acetate = 8:2:0.05 to 8:2:0.1) gave the title compound as a white solid (21.1 mg, 45.9 µmol, 46%).

^{1}H NMR (400 MHz, $CDCl_3$) δ 7.66 – 7.60 (m, 2H), 7.47 – 7.39 (m, 3H), 7.37 – 7.26 (m, 3H), 7.23 –

7.15 (m, 2H), 7.14 – 7.06 (m, 2H), 6.85 – 6.77 (m, 2H), 4.46 (s, 1H), 3.82 – 3.73 (m, 3H), 2.74 (s, 2H), 3.24 – 1.10 (m, 9H).

^{13}C{^{1}H} NMR (101 MHz, $CDCl_3$) δ 164.7, 158.2, 140.5, 138.4, 137.5, 129.6, 128.9, 128.8, 128.3, 128.2, 128.1, 128.0, 113.5, 66.3, 55.3, 54.2, 25.5.

^{11}B{^{1}H} NMR (128 MHz, $CDCl_3$) δ -0.7, -6.5, -10.0, -13.3, -14.2, -17.8, -20.1.

HRMS (ESI): *m/z* calculated for $[C_{24}H_{29}NOB_{10}D_2Na]^+$ $[M+Na]^+$: 482.3438; found: 482.3439.

*The protons next to the carboranyl group from undeuterated **5ac** were not observed in the ^{1}H NMR spectrum of **5ac''**.*

Carboranylaminated product 5ad

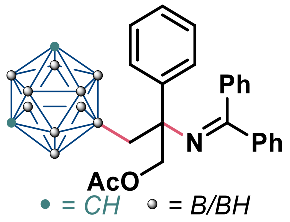

The title compound was prepared according to general procedure **GP2** from bifunctional reagent **3a** (36.7 mg, 99.9 µmol, 1.0 equiv) and alkene **4ac** (35.2 mg, 0.200 mmol, 2.0 equiv). Column chromatography (*n*-pentane/ethyl acetate = 30:1) gave the title compound as a white solid (22.0 mg, 44.0 µmol, 44%).

^{1}H NMR (400 MHz, $CDCl_3$) δ 7.65 – 7.58 (m, 2H), 7.38 – 7.15 (m, 6H), 7.13 – 7.05 (m, 5H), 6.80 – 6.70 (m, 2H), 4.68 (d, *J* = 11.0 Hz, 1H), 4.43 (d, *J* = 11.0 Hz, 1H), 2.81 (s, 2H), 1.89 (s, 2H), 1.86 (s, 3H), 3.39 – 1.11 (m, 9H).

^{13}C{^{1}H} NMR (101 MHz, $CDCl_3$) δ 171.1, 165.8, 147.4, 141.5, 138.9, 129.7, 128.5, 127.9, 127.5, 127.5, 127.5, 127.3, 127.3, 126.1, 71.0, 65.3, 54.4, 54.4, 24.6, 21.1.

^{11}B{^{1}H} NMR (128 MHz, $CDCl_3$) δ -1.4, -6.1, -9.4, -13.0, -14.1, -17.7, -19.7.

HRMS (ESI): *m/z* calculated for $[C_{26}H_{33}NO_2B_{10}Na]^+$ $[M+Na]^+$: 522.3419; found: 522.3410.

Carboranylaminated product 5ae

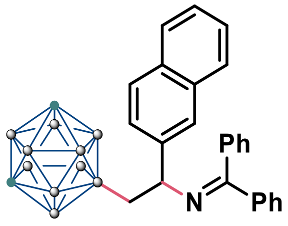

The title compound was prepared according to general procedure **GP2** from bifunctional reagent **3a** (36.7 mg, 99.9 µmol, 1.0 equiv) and alkene **4ad** (30.8 mg, 0.200 mmol, 2.0 equiv). Column chromatography (*n*-pentane/CH_2Cl_2/ethyl acetate = 8:2:0.05) gave the title compound as a white solid (17.5 mg, 36.6 µmol, 37%).

^{1}H NMR (400 MHz, $CDCl_3$) δ 7.84 – 7.73 (m, 3H), 7.70 – 7.59 (m, 3H), 7.55 – 7.49 (m, 1H), 7.47 – 7.39 (m, 5H), 7.37 – 7.28 (m, 3H), 7.13 – 7.06 (m, 2H), 4.67 (t, *J* = 6.8 Hz, 1H), 2.74 (s, 2H), 1.87 (dd, *J* = 15.2, 7.0 Hz, 1H), 1.72 (dd, *J* = 15.1, 6.5 Hz, 1H), 3.27 – 1.11 (m, 9H).

^{13}C{^{1}H} NMR (101 MHz, $CDCl_3$) δ 165.6, 144.1, 140.5, 137.5, 133.6, 132.7, 129.7, 128.9, 128.9, 128.4, 128.3, 128.2, 128.1, 127.8, 127.7, 126.5, 126.2, 125.7, 125.3, 67.2, 54.3, 26.5.

^{11}B{^{1}H} NMR (128 MHz, $CDCl_3$) δ -0.6, -6.4, -10.0, -13.3, -14.1, -17.7, -20.1.

HRMS (ESI): *m/z* calculated for $[C_{27}H_{31}NB_{10}H]^+$ $[M+H]^+$: 478.3545; found: 478.3531.

Carboranylaminated product 5af

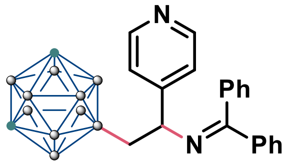

The title compound was prepared according to general procedure **GP2** from bifunctional reagent **3a** (36.7 mg, 99.9 µmol, 1.0 equiv) and alkene **4ae** (21.0 mg, 0.200 mmol, 2.0 equiv). Column chromatography (*n*-pentane/ethyl acetate = 10:1 to 4:1) gave the title compound as a white solid (21.6 mg, 50.4 µmol, 50%).

^{1}H NMR (400 MHz, $CDCl_3$) δ 8.52 – 8.42 (m, 2H), 7.68 – 7.61 (m, 2H), 7.46 – 7.24 (m, 8H), 7.08 – 6.99 (m, 2H), 4.48 (t, *J* = 6.8 Hz, 1H), 2.78 (s, 2H), 1.74 – 1.59 (m, 2H), 3.32 – 1.00 (m, 9H).

$^{13}C\{^1H\}$ **NMR** (101 MHz, $CDCl_3$) δ 167.1, 156.6, 148.7, 139.8, 136.9, 130.2, 128.9, 128.6, 128.5, 128.1, 127.8, 123.4, 66.0, 54.4, 26.7.

$^{11}B\{^1H\}$ **NMR** (128 MHz, $CDCl_3$) δ -1.0, -6.4, -10.0, -13.3, -14.0, -17.7, -19.9.

HRMS (ESI): *m/z* calculated for $[C_{22}H_{28}N_2B_{10}Na]^+$ $[M+Na]^+$: 451.3159; found: 451.3147.

Carboranylaminated product 5ag

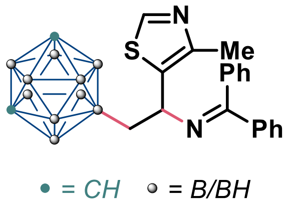

The title compound was prepared according to general procedure **GP2** from bifunctional reagent **3a** (36.7 mg, 99.9 µmol, 1.0 equiv) and alkene **4af** (25.0 mg, 0.200 mmol, 2.0 equiv). Column chromatography (*n*-pentane/ethyl acetate = 15:1 to 5:1) gave the title compound as a white solid (13.9 mg, 31.0 µmol, 31%).

^1H **NMR** (400 MHz, $CDCl_3$) δ 8.64 (s, 1H), 7.68 – 7.60 (m, 2H), 7.52 – 7.44 (m, 3H), 7.42 – 7.29 (m, 3H), 7.18 – 7.09 (m, 2H), 4.86 (s, 1H), 2.78 (s, 2H), 2.01 (s, 3H), 1.85 – 1.75 (m, 1H), 1.68 (dd, *J* = 14.3, 4.2 Hz, 1H), 3.32 – 1.02 (m, 9H).

$^{13}C\{^1H\}$ **NMR** (101 MHz, $CDCl_3$) δ 166.8, 151.1, 147.6, 139.5, 137.5, 136.4, 130.6, 129.2, 129.0, 128.7, 128.2, 128.0, 59.6, 54.4, 28.0, 15.5.

$^{11}B\{^1H\}$ **NMR** (192 MHz, $CDCl_3$) δ -1.3, -6.6, -10.1, -13.4, -14.0, -17.7, -19.9.

HRMS (ESI): *m/z* calculated for $[C_{21}H_{28}N_2SB_{10}Na]^+$ $[M+Na]^+$: 471.2879; found: 471.2870.

Carboranylaminated product 5ah

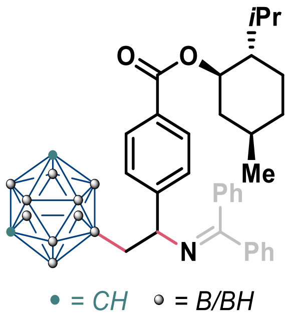

The title compound was prepared according to general procedure **GP2** from bifunctional reagent **3a** (36.7 mg, 99.9 µmol, 1.0 equiv) and alkene **4ag** (57.3 mg, 0.200 mmol, 2.0 equiv). Column chromatography (*n*-pentane/ethyl acetate = 100:1 to 75:1) gave the title compound as a white solid (36.2 mg, 59.4 µmol, 59%) in 51:49 d.r.

^{1}H NMR (400 MHz, $CDCl_3$) δ 8.00 – 7.87 (m, 2H), 7.68 – 7.60 (m, 2H), 7.45 – 7.40 (m, 3H), 7.38 – 7.27 (m, 5H), 7.09 – 7.02 (m, 2H), 4.97 – 4.86 (m, 1H), 4.63 – 4.47 (m, 1H), 2.76 (s, 2H), 2.19 – 2.08 (m, 1H), 2.04 – 1.92 (m, 1H), 1.81 – 1.48 (m, 6H), 1.21 – 1.02 (m, 2H), 1.00 – 0.84 (m, 7H), 0.82 – 0.74 (m, 3H), 3.07 – 0.56 (m, 9H).

^{13}C{^{1}H} NMR (101 MHz, $CDCl_3$) δ 166.4, 165.9, 165.8, 151.8, 151.7, 140.2, 137.3, 129.9, 129.6, 129.1, 128.8, 128.4, 128.4, 128.1, 128.0, 127.8, 127.7, 74.7, 66.9, 66.9, 54.3, 54.3, 47.4, 47.4, 41.1, 34.5, 31.6, 26.8, 26.6, 23.7, 22.2, 21.0, 20.9, 16.6.

^{11}B{^{1}H} NMR (128 MHz, $CDCl_3$) δ -0.8, -6.5, -10.0, -13.3, -14.1, -17.7, -20.1.

HRMS (ESI): *m/z* calculated for $[C_{34}H_{47}NO_2B_{10}H]^+$ $[M+H]^+$: 609.4730; found: 609.4724.

Carboranylaminated product 5ai

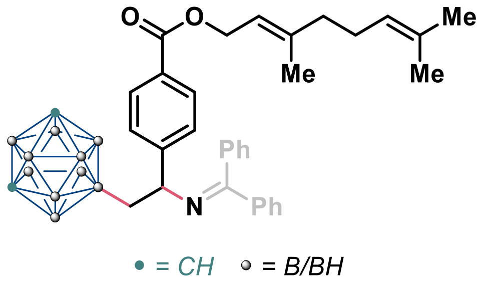

The title compound was prepared according to general procedure **GP2** from bifunctional reagent **3a** (36.7 mg, 99.9 µmol, 1.0 equiv) and alkene **4ah** (56.9 mg, 0.200 mmol, 2.0 equiv). Column chromatography (*n*-pentane/ethyl acetate = 60:1) gave the title compound as a colorless oil (26.2 mg, 43.1 µmol, 43%).

^{1}H NMR (400 MHz, $CDCl_3$) δ 7.95 (d, *J* = 8.0 Hz, 2H), 7.63 (d, *J* = 7.4 Hz, 2H), 7.46 – 7.39 (m, 3H), 7.38 – 7.28 (m, 5H), 7.10 – 7.02 (m, 2H), 5.51 – 5.42 (m, 1H), 5.14 – 5.05 (m, 1H), 4.82 (d, *J* = 7.0 Hz, 2H), 4.54 (t, *J* = 6.9 Hz, 1H), 2.75 (s, 2H), 2.09 (tq, *J* = 9.2, 5.1 Hz, 4H), 1.78 – 1.70 (m, 4H), 1.69 – 1.58 (m, 7H), 3.17 – 1.08 (m, 9H).

^{13}C{^{1}H} NMR (101 MHz, $CDCl_3$) δ 166.9, 165.9, 151.8, 142.3, 140.3, 137.3, 132.0, 129.9, 129.7, 128.8, 128.5, 128.4, 128.4, 128.1, 128.0, 127.9, 123.9, 118.7, 66.9, 61.9, 54.3, 39.7, 26.7, 26.5, 25.8, 17.8, 16.7.

^{11}**B{^{1}H} NMR** (128 MHz, $CDCl_3$) δ -0.8, -6.5, -10.0, -13.3, -14.1, -17.8, -20.1.

HRMS (ESI): *m/z* calculated for $[C_{34}H_{45}NO_2B_{10}Na]^+$ $[M+Na]^+$: 629.4393; found: 629.4384.

Carboranylaminated product 5aj

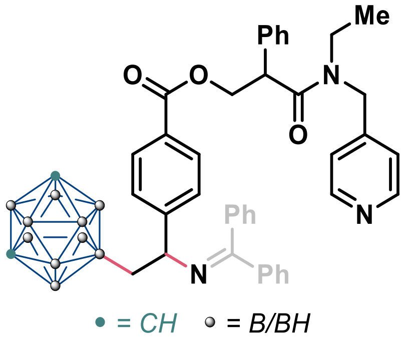

The title compound was prepared according to general procedure **GP2** from bifunctional reagent **3a** (36.7 mg, 99.9 µmol, 1.0 equiv) and alkene **4ai** (82.9 mg, 0.200 mmol, 2.0 equiv). Column chromatography (*n*-pentane/ethyl acetate = 2:1 to 1:1) gave the title compound as a white solid (33.8 mg, 45.8 µmol, 46%) in 51:49 d.r.

^{1}**H NMR** (400 MHz, $CDCl_3$) δ 8.56 – 8.29 (m, 2H), 7.94 – 7.84 (m, 2H), 7.71 – 7.63 (m, 2H), 7.50 – 7.27 (m, 13H), 7.11 – 6.91 (m, 4H), 5.02 – 4.82 (m, 1H), 4.75 – 3.99 (m, 5H), 3.76 – 3.36 (m, 1H), 3.28 – 3.12 (m, 1H), 2.78 (s, 2H), 1.81 (dd, *J* = 15.1, 7.5 Hz, 1H), 1.69 (dd, *J* = 15.1, 6.4 Hz, 1H), 1.16 – 0.94 (m, 3H), 3.23 – 1.10 (m, 9H).

^{13}**C{^{1}H} NMR** (101 MHz, $CDCl_3$) δ 171.1, 170.8, 170.8, 167.5, 167.4, 166.6, 166.6, 166.4, 166.4, 151.3, 150.2, 149.6, 147.5, 147.4, 146.4, 146.4, 139.2, 136.6, 136.6, 136.5, 135.7, 135.3, 135.3, 130.6, 130.5, 129.7, 129.3, 129.3, 129.0, 128.9, 128.5, 128.4, 128.4, 128.3, 128.2, 128.2, 128.2, 128.2, 128.0, 128.0, 128.0, 122.4, 121.2, 67.1, 66.9, 66.8, 54.4, 49.5, 48.6, 48.1, 48.1, 48.0, 48.0, 42.7, 41.8, 26.1, 14.2, 12.6.

^{11}**B{^{1}H} NMR** (128 MHz, $CDCl_3$) δ -0.9, -6.5, -10.0, -13.2, -14.1, -17.7, -19.8.

HRMS (ESI): *m/z* calculated for $[C_{41}H_{47}N_3O_3B_{10}H]^+$ $[M+H]^+$: 739.4687; found: 739.4679.

Note: The product forms a 2:1 mixture of rotamers.

Carboranylaminated product 5ak

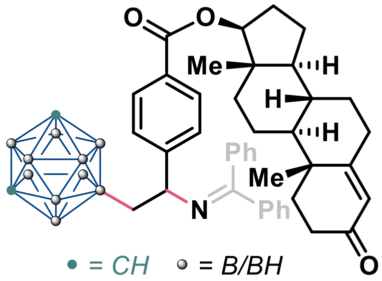

The title compound was prepared according to general procedure **GP2** from bifunctional reagent **3a** (36.7 mg, 99.9 µmol, 1.0 equiv) and alkene **4aj** (83.7 mg, 0.200 mmol, 2.0 equiv). Column chromatography (*n*-pentane/ethyl acetate = 12:1 to 8:1) gave the title compound as a white sticky solid (20.2 mg, 27.2 µmol, 27%) in 50:50 d.r.

^{1}H NMR (400 MHz, $CDCl_3$) δ 7.94 (d, J = 7.9 Hz, 2H), 7.63 (s, 2H), 7.48 – 7.28 (m, 8H), 7.05 (s, 2H), 5.74 (s, 1H), 4.87 – 4.78 (m, 1H), 4.54 (s, 1H), 2.76 (s, 2H), 2.50 – 2.22 (m, 5H), 2.03 (d, J = 13.3 Hz, 1H), 1.93 – 1.82 (m, 2H), 1.67 (dtd, J = 32.0, 15.4, 8.0 Hz, 7H), 1.51 – 1.34 (m, 2H), 1.31 – 0.92 (m, 10H), 3.30 – 0.69 (m, 9H).

^{13}C{^{1}H} NMR (101 MHz, $CDCl_3$) δ 199.6, 171.1, 166.7, 165.9, 151.9, 140.2, 137.1, 129.8, 129.6, 128.8, 128.5, 128.4, 128.2, 128.1, 128.0, 127.9, 124.1, 82.9, 82.9, 66.9, 66.9, 54.4, 53.9, 50.4, 43.0, 43.0, 38.8, 36.9, 35.8, 35.6, 34.1, 32.9, 31.7, 27.8, 27.8, 26.7, 23.8, 20.7, 17.6, 12.4.

^{11}B{^{1}H} NMR (128 MHz, $CDCl_3$) δ -0.9, -6.5, -10.0, -13.3, -14.1, -17.8, -20.0.

HRMS (ESI): *m/z* calculated for $[C_{43}H_{55}NO_3B_{10}Na]^+$ $[M+Na]^+$: 765.5072; found: 765.5066.

Carboranylaminated product 5al

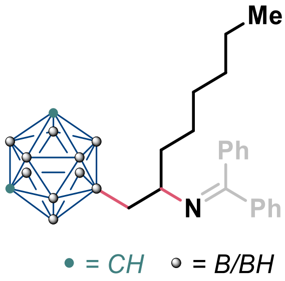

The title compound was prepared according to general procedure **GP2** on double the normal scale from bifunctional reagent **3a** (73.4 mg, 0.200 mmol, 1.0 equiv), alkene **4ak** (224 mg, 2.00 mmol, 10 equiv), and 2-isopropylthioxanthone (2.6 mg, 10 µmol, 5 mol%) in dry ethyl acetate (4.0 mL, 50 mM). Column chromatography (*n*-pentane/ethyl acetate = 500:1 to 300:1) gave the title compound as a colorless oil (22.5 mg, 51.6 µmol, 26%).

^{1}**H NMR** (400 MHz, $CDCl_3$) δ 7.62 – 7.57 (m, 2H), 7.46 – 7.36 (m, 3H), 7.35 – 7.27 (m, 3H), 7.19 – 7.12 (m, 2H), 3.37 (tt, J = 9.0, 3.2 Hz, 1H), 2.81 (s, 2H), 1.76 (dddd, J = 13.2, 9.6, 5.9, 3.2 Hz, 1H), 1.63 – 1.51 (m, 1H), 1.40 (dd, J = 14.9, 9.7 Hz, 1H), 1.35 – 1.00 (m, 9H), 0.85 (t, J = 6.8 Hz, 3H), 3.48 – 0.82 (m, 9H).

^{13}**C{^{1}H} NMR** (101 MHz, $CDCl_3$) δ 164.9, 140.9, 137.9, 129.5, 128.6, 128.3, 128.2, 128.1, 128.0, 62.5, 54.3, 36.8, 32.1, 29.5, 27.1, 24.3, 22.8, 14.3.

^{11}**B{^{1}H} NMR** (128 MHz, $CDCl_3$) δ -0.3, -6.4, -9.9, -13.2, -14.1, -17.8, -20.2.

HRMS (ESI): *m/z* calculated for $[C_{23}H_{37}NB_{10}Na]^+$ $[M+Na]^+$: 458.3833; found: 458.3819.

Carboranylaminated product 5am

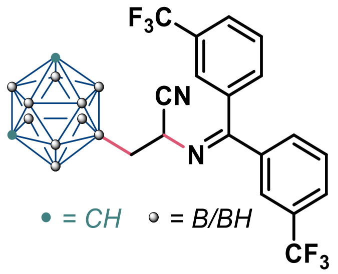

The title compound was prepared according to general procedure **GP2** from bifunctional reagent **3e** (50.3 mg, 99.9 µmol, 1.0 equiv) and alkene **4a** (10.6 mg, 0.200 mmol, 2.0 equiv). Column chromatography (*n*-pentane/ethyl acetate = 49:1 to 19:1) gave the title compound as a white solid (20.5 mg, 40.0 µmol, 40%).

^{1}**H NMR** (400 MHz, $CDCl_3$) δ 8.01 (s, 1H), 7.83 – 7.79 (m, 1H), 7.76 – 7.66 (m, 3H), 7.53 – 7.45 (m, 3H), 4.17 – 4.10 (m, 1H), 2.90 (s, 2H), 1.83 (dd, J = 15.0, 8.6 Hz, 1H), 1.44 (dd, J = 15.0, 5.8 Hz, 1H), 3.41 – 0.91 (m, 9H).

^{13}**C{^{1}H} NMR** (126 MHz, $CDCl_3$) δ 168.5, 138.9, 135.4, 132.4 (q, J = 0.9 Hz), 132.0 (q, J = 33.0 Hz), 131.2 (q, J = 32.6 Hz), 131.0 (q, J = 1.2 Hz), 130.1, 129.1, 128.0 (q, J = 3.7 Hz), 126.7 (q, J = 3.6 Hz), 125.5 (q, J = 3.9 Hz), 124.2 (q, J = 3.8 Hz), 123.9 (q, J = 272.5 Hz), 123.7 (q, J = 273.0 Hz), 120.3, 54.8, 53.7, 22.4.

^{11}**B{^{1}H} NMR** (128 MHz, $CDCl_3$) δ -2.4, -6.5, -10.0, -13.2, -13.7, -17.4, -19.4.

^{19}**F{^{1}H} NMR** (376 MHz, $CDCl_3$) δ -62.8, -62.8.

HRMS (ESI): *m/z* calculated for $[C_{20}H_{22}B_{10}N_2F_6Na]^+$ $[M+Na]^+$: 535.2592; found: 535.2585.

Carboranylaminated product 5an

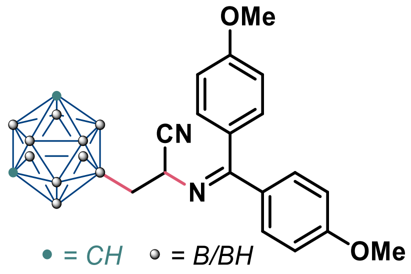

The title compound was prepared according to general procedure **GP2** from bifunctional reagent **3f** (42.8 mg, 0.100 mmol, 1.0 equiv) and alkene **4a** (10.6 mg, 0.200 mmol, 2.0 equiv). Column chromatography (*n*-pentane/ethyl acetate = 10:1) gave the title compound as a white solid (24.4 mg, 55.9 µmol, 56%).

^{1}H NMR (400 MHz, $CDCl_3$) δ 7.64 – 7.55 (m, 2H), 7.20 – 7.12 (m, 2H), 7.03 – 6.97 (m, 2H), 6.88 – 6.81 (m, 2H), 4.26 (dd, *J* = 8.2, 6.3 Hz, 1H), 3.88 (s, 3H), 3.82 (s, 3H), 2.88 (s, 2H), 1.79 (dd, *J* = 15.0, 8.2 Hz, 1H), 1.50 (dd, *J* = 14.9, 6.3 Hz, 1H), 3.40 – 1.01 (m, 9H).

^{13}C{^{1}H} NMR (101 MHz, $CDCl_3$) δ 171.1, 162.0, 160.2, 132.1, 131.0, 129.4, 127.7, 121.4, 114.2, 113.6, 55.5, 54.7, 53.3, 22.8.

^{11}B{^{1}H} NMR (128 MHz, $CDCl_3$) δ -1.9, -6.4, -9.9, -13.2, -13.8, -17.5, -19.5.

HRMS (ESI): *m/z* calculated for $[C_{20}H_{28}N_2O_2B_{10}Na]^+$ $[M+Na]^+$: 459.3056; found: 459.3038.

Carboranylaminated product 5ao

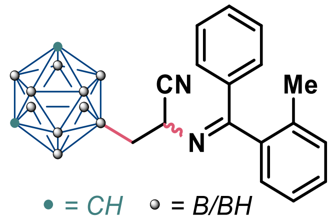

The title compound was prepared according to general procedure **GP2** from bifunctional reagent **3g** (38.1 mg, 99.9 µmol, 1.0 equiv) and alkene **4a** (10.6 mg, 0.200 mmol, 2.0 equiv). Column chromatography (*n*-pentane/ethyl acetate = 50:1 to 15:1) gave the title compound as a white solid (20.0 mg, 51.2 µmol, 51%).

^{1}H NMR (400 MHz, $CDCl_3$) δ 7.72 – 7.65 (m, 2H), 7.47 – 7.27 (m, 6H), 7.22 – 6.95 (m, 1H), 4.20 – 4.10 (m, 1H), 2.88 (s, 2H), 2.19 – 2.01 (m, 3H), 1.93 – 1.81 (m, 1H), 1.31 – 1.21 (m, 1H), 3.36 – 1.06 (m, 9H).

^{13}C{^{1}H} **NMR** (101 MHz, $CDCl_3$) δ 172.2, 171.6, 137.7, 135.5, 134.9, 134.9, 134.7, 131.4, 131.3, 130.9, 130.8, 129.5, 129.4, 128.8, 128.7, 128.6, 128.5, 127.6, 126.7, 126.6, 126.3, 120.5, 120.5, 54.7, 53.9, 53.5, 22.2, 19.7, 19.6.

^{11}B{^{1}H} **NMR** (128 MHz, $CDCl_3$) δ -2.2, -6.4, -9.9, -13.2, -13.8, -17.4, -19.5.

HRMS (ESI): *m/z* calculated for $[C_{19}H_{26}N_2B_{10}Na]^+$ $[M+Na]^+$: 413.3001; found: 413.2977.

Note: The product forms a 3:2 mixture of isomers (E/Z isomers of the imine).

4.2 Limitations

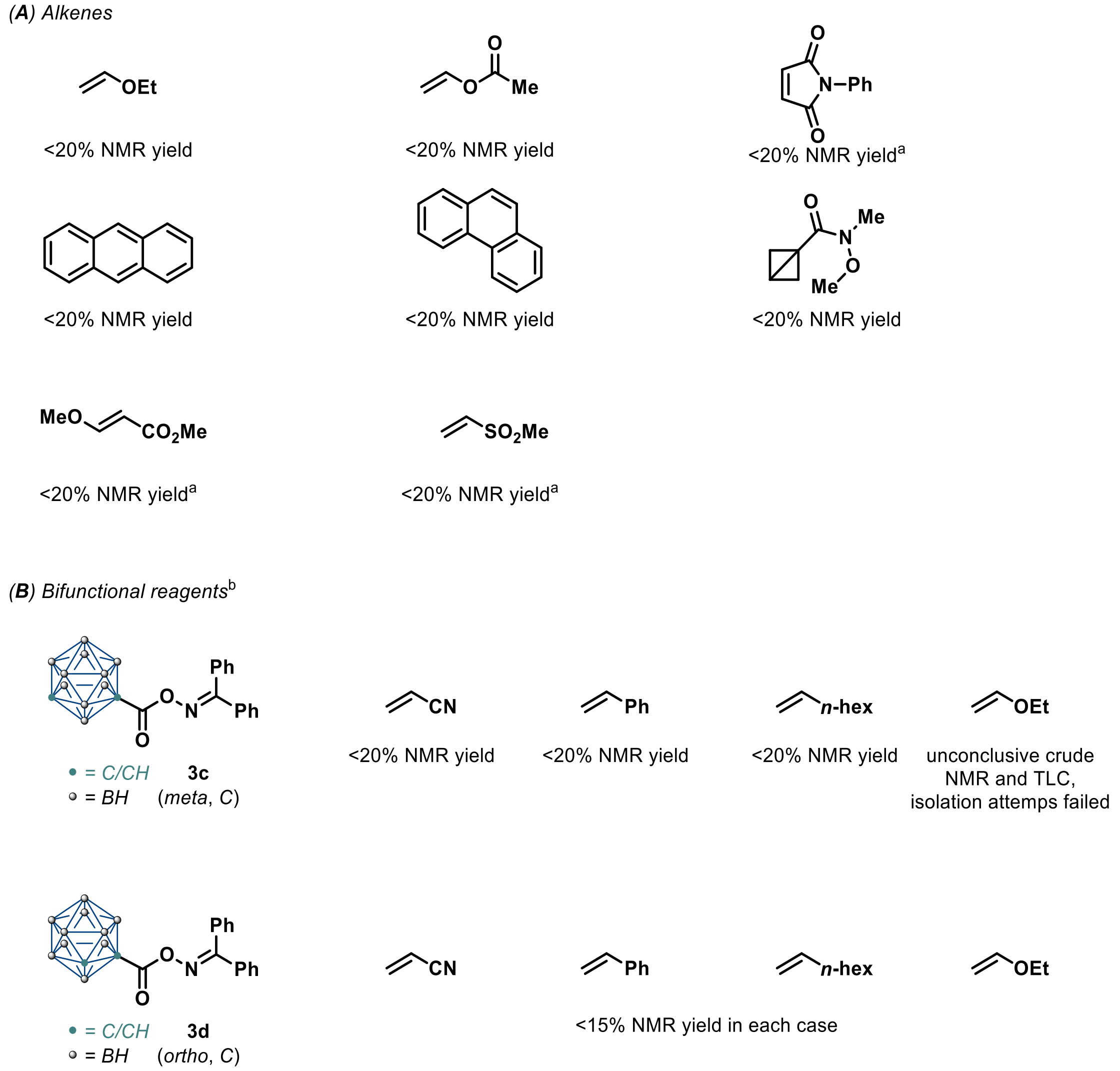

Supplementary Figure 11. Unsuccessful substrates. Reactions were performed on 0.05 mmol scale following the standard conditions with **3a** as bifunctional reagent. NMR yields were determined with mesitylene as internal standard. [a]Carried out on 0.1 mmol scale. [b]**3c** or **3d** used instead of **3a** and reactions carried out on 0.025 mmol scale.

5. Mechanistic experiments

5.1 UV/vis absorption spectroscopy

UV/vis absorption spectroscopy measurements were performed on a Jasco V-730 spectrophotometer, equipped with a temperature control unit at 25 °C. The samples were measured in Starna® fluorescence quartz cuvettes (type: 29-F, chamber volume = 1.400 mL, H × W × D = 48 mm × 12.5 mm × 12.5 mm, path length = 10 mm). The spectra were recorded using the following parameters: response time = 0.06 sec, data interval = 0.2 nm, scan speed = 400 nm/min. The reaction mixture and all individual components (in EtOAc) were measured at half the standard concentrations.

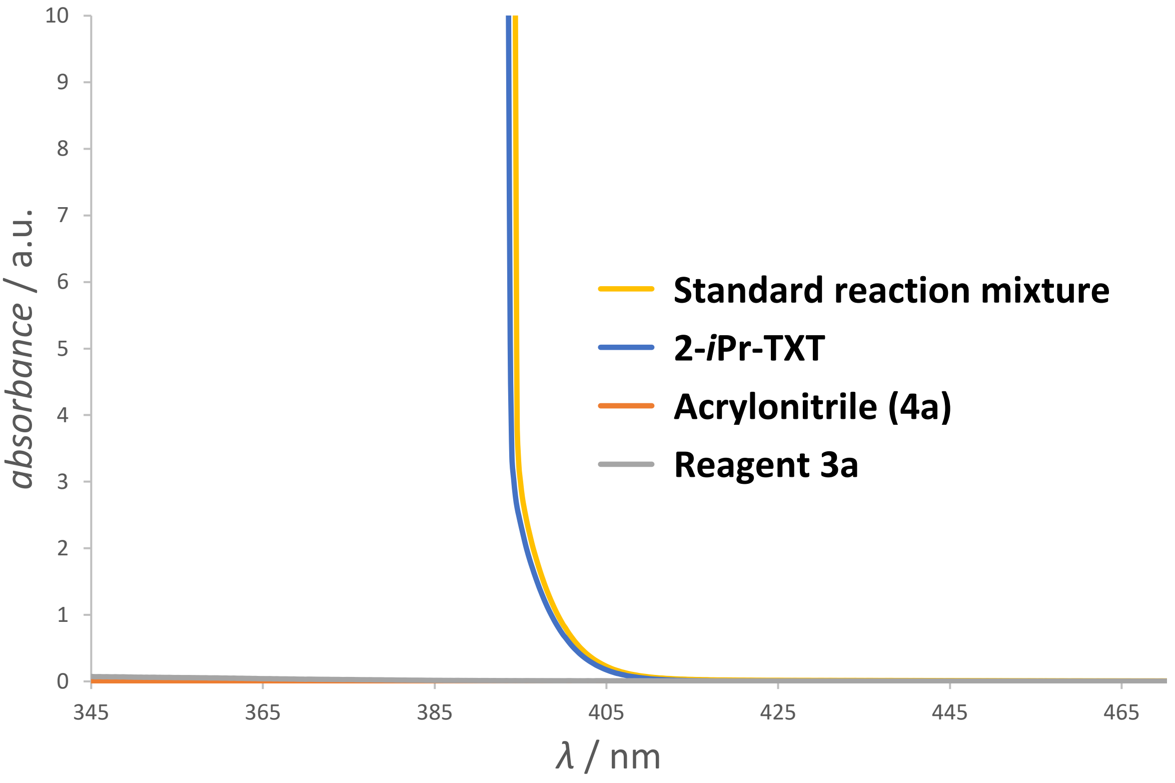

Supplementary Figure 12. UV/vis absorption spectra of the standard reaction mixture of **3a** and **4a** with half the standard concentration (yellow), 2-isopropylthioxanthone (c = 1.25 mM, blue), alkene **4a** (*c* = 50 mM, orange), and reagent **3a** (*c* = 25 mM, grey).

5.2 Stern–Volmer luminescence quenching studies

To identify species capable of quenching the excited 2-isopropylthioxanthone photocatalyst, Stern–Volmer luminescence quenching studies were performed. For this purpose, the luminescence of the excited photocatalyst was measured in the presence of different concentrations of the potential quenchers.

A JASCO FP-8300 spectrofluorometer was used for recording the emission spectra. Samples were introduced into the device in Starna® fluorescence quartz cuvettes (type: 29-F, chamber volume = 1.400 mL, H × W × D = 48 mm × 12.5 mm × 12.5 mm, path length = 10 mm). The spectra were recorded using the following parameters: excitation bandwidth = 5 nm, scan speed = 500 nm/min, data interval = 0.5 nm, response time = 0.1 sec, excitation wavelength λ_{ex} = 370 nm. Emission intensities of the samples were measured at λ_{em} = 402 nm. The samples were prepared in a glovebag filled with argon using degassed, dry EtOAc. The employed concentration of 2-isopropylthioxanthone was *c*(2-isopropylthioxanthone) = 50 μM. Cuvettes were capped with a PTFE stopper, shaken, and removed from the glovebag for measurement.

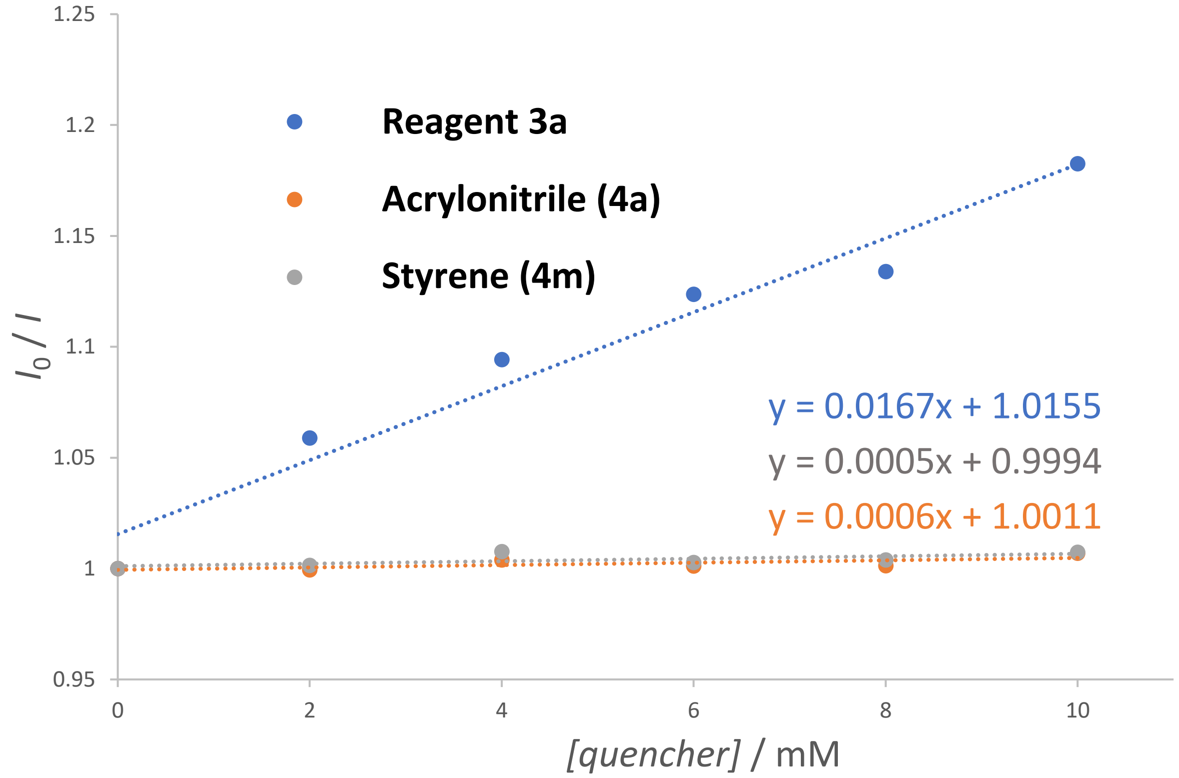

Supplementary Figure 13. Stern–Volmer luminescence quenching results.

5.3 Direct excitation experiment

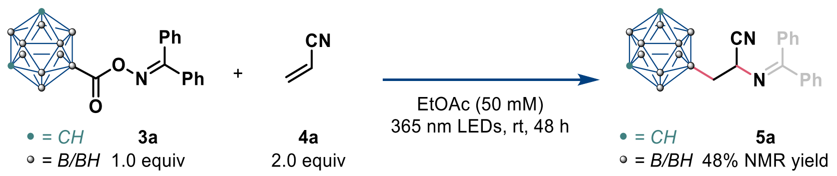

An oven-dried 10 mL Schlenk tube was charged with a Teflon-coated stirring bar and bifunctional reagent **3a** (18.4 mg, 0.0501 mmol, 1.0 equiv). The tube was evacuated and backfilled with argon three times. Dry ethyl acetate (1.0 mL, 50 mM) followed by alkene **4a** (5.3 mg, 0.10 mmol,

2.0 equiv) was added under argon counter flow. The mixture was then irradiated at 365 nm for 48 h. After that, the solvent was removed in vacuo and the residue was examined by ^{1}H NMR spectroscopy with mesitylene as internal standard. The NMR yield was determined to be 48%.

5.4 Radical trapping studies

TEMPO trapping experiment w/o alkene 4

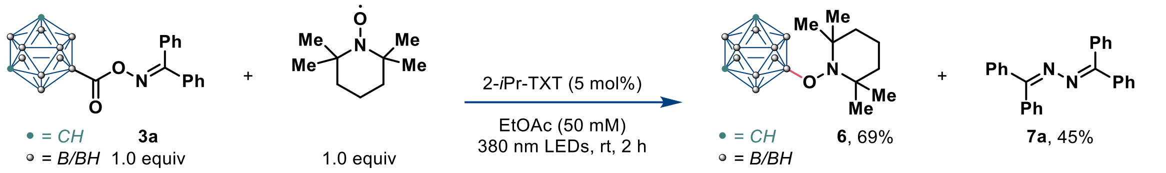

An oven-dried 10 mL Schlenk tube was charged with a Teflon-coated stirring bar, 2-isopropylthioxanthone (0.6 mg, 2 µmol, 5 mol%), bifunctional reagent **3a** (18.4 mg, 50.1 µmol, 1.0 equiv), and TEMPO (7.8 mg, 50 µmol, 1.0 equiv). The tube was evacuated and backfilled with argon three times. Dry ethyl acetate (1.0 mL, 50 mM) was added under argon counter flow and the mixture was then irradiated at 380 nm for 2 h. After that, the mixture was examined by GC-MS analysis. Furthermore, products **6** and **7a** were both isolated from the reaction mixture by column chromatography on silica (*n*-pentane/ethyl acetate = 60:1 to 25:1). The characterization data for both products is given below.

GC-MS analysis (Supplementary Figure 14) indicated *m*-carborane, TEMPO trapped species **6**, and imine dimer **7a** as major reaction products.

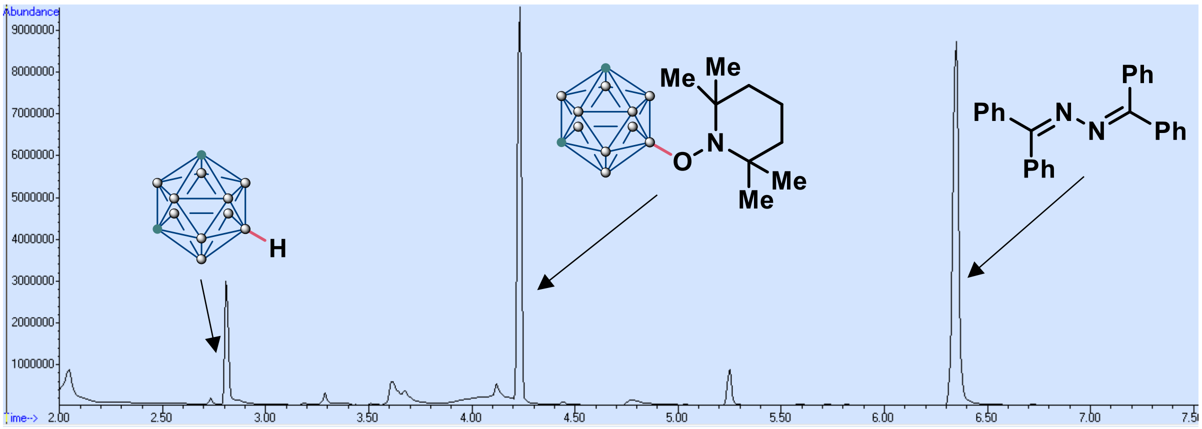

Supplementary Figure 14. GC-MS analysis of the crude reaction mixture.

TEMPO adduct 6

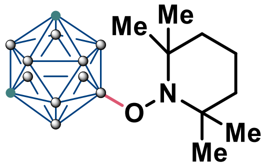

The product was obtained as a white solid (10.3 mg, 0.0344 mmol, 69%).

^{1}H NMR (400 MHz, $CDCl_3$) δ 2.65 (s, 2H), 1.54 – 1.08 (m, 18H), 3.25 – 0.98 (m, 9H).

^{13}C{^{1}H} NMR (101 MHz, $CDCl_3$) δ 60.9, 49.0, 40.3, 35.2, 20.1, 17.3.

^{11}B{^{1}H} NMR (128 MHz, $CDCl_3$) δ 10.8, -8.4, -11.8, -15.1, -17.2, -20.7, -26.2.

HRMS (ESI): *m/z* calculated for $[C_{11}H_{29}NOB_{10}H]^+$ $[M+H]^+$: 300.3331; found: 300.3323.

1,2-Bis(diphenylmethylene)hydrazine (7a)

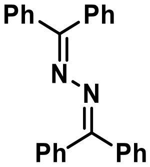

The product was obtained as a white solid (4.1 mg, 0.011 mmol, 45%).

^{1}H NMR (400 MHz, $CDCl_3$) δ 7.49 – 7.45 (m, 4H), 7.44 – 7.25 (m, 16H).

^{13}C{^{1}H} NMR (101 MHz, $CDCl_3$) δ 159.1, 138.3, 135.7, 129.7, 129.5, 128.8, 128.8, 128.2, 128.0.

HRMS (ESI): *m/z* calculated for $[C_{26}H_{20}N_2Na]^+$ $[M+Na]^+$: 383.1519; found: 383.1520.

Discussion: Upon triplet sensitization of reagent **3a** and formation of a carboranyl and iminyl radical, the carboranyl radical is TEMPO trapped (**6**). The iminyl radical dimerizes towards imine dimer **7a**. *m*-Carborane can likely result from formation of a carboranyl radical that abstracts a hydrogen atom from e.g. the solvent.

TEMPO trapping experiment w/ alkene 4a

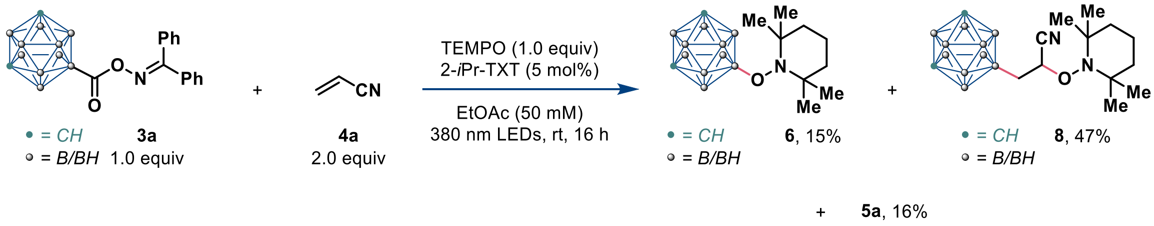

An oven-dried 10 mL Schlenk tube was charged with a Teflon-coated stirring bar, 2-isopropylthioxanthone (1.3 mg, 5.1 µmol, 5 mol%), bifunctional reagent **3a** (36.7 mg, 0.0999 mmol, 1.0 equiv), and TEMPO (15.6 mg, 99.8 µmol, 1.0 equiv). The tube was evacuated and backfilled with argon three times. Dry ethyl acetate (2.0 mL, 50 mM) followed by alkene **4a** (10.6 mg, 0.200 mmol, 2.0 equiv) was added under argon counter flow. The mixture was then irradiated at 380 nm for 16 h. After that, the solvent was removed in vacuo and the residue was purified by column chromatography (*n*-pentane/ethyl acetate = 49:1 to 19:1), isolating three products:

Carboranylaminated product 5a

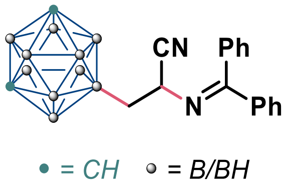

The title compound was obtained as a white solid (5.9 mg, 16 µmol, 16%). The analytical data is in accordance with the data of our previously herein described isolation of this compound.

TEMPO adduct 6

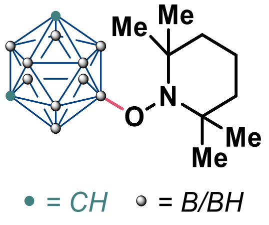

The title compound was obtained as a white solid (4.6 mg, 15 µmol, 15%). The analytical data is in accordance with the data of our previously herein described isolation of this compound.

TEMPO adduct 8

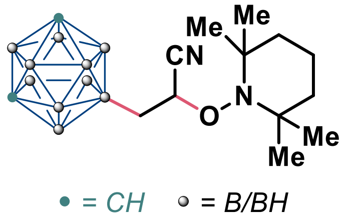

The product was obtained as a white solid (16.4 mg, 0.0465 mmol, 47%).

^{1}H NMR (599 MHz, $CDCl_3$) δ 4.62 (s, 1H), 2.99 – 2.88 (m, 2H), 1.68 – 1.32 (m, 11H), 1.21 (s, 3H), 1.14 (s, 3H), 1.10 (s, 3H), 3.13 – 1.64 (m, 9H).

^{13}C{^{1}H} NMR (151 MHz, $CDCl_3$) δ 120.5, 75.1, 61.1, 59.8, 54.8, 39.9, 34.1, 33.4, 20.7, 20.7, 20.6, 17.1.

^{11}B{^{1}H} NMR (128 MHz, $CDCl_3$) δ -2.5, -6.3, -9.8, -13.1, -13.8, -17.4, -19.6.

HRMS (ESI): *m/z* calculated for $[C_{14}H_{32}B_{10}N_2ONa]^+$ $[M+Na]^+$: 375.3417; found: 375.3407.

BHT trapping experiment w/ alkene 4a

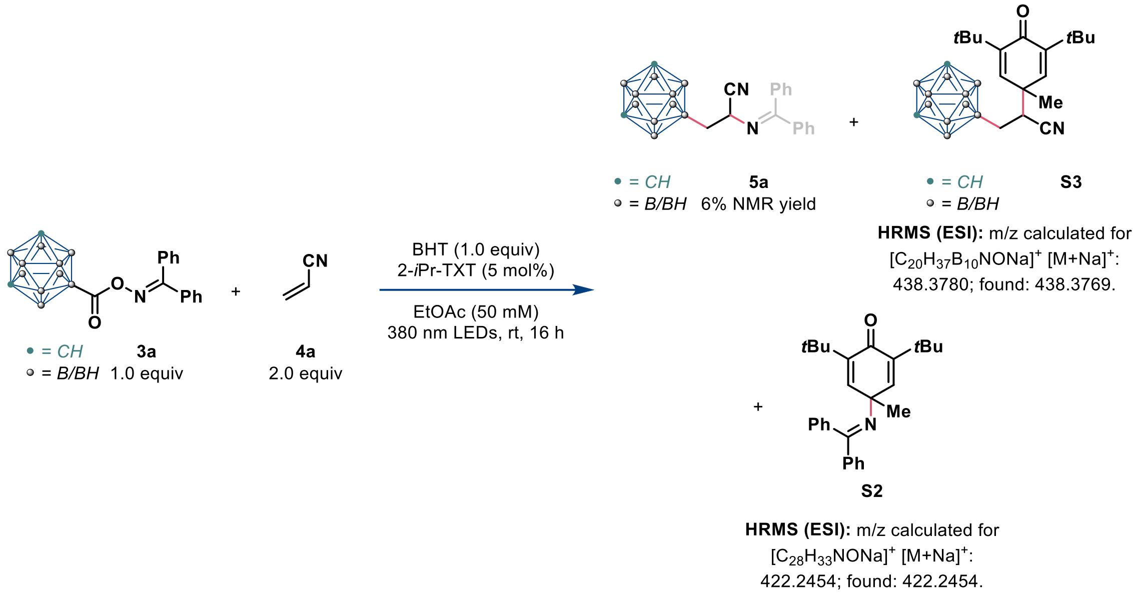

An oven-dried 10 mL Schlenk tube was charged with a Teflon-coated stirring bar, 2-isopropylthioxanthone (0.6 mg, 2 µmol, 5 mol%), bifunctional reagent **3a** (18.4 mg, 50.1 µmol, 1.0 equiv), and butylated hydroxytoluene (11.0 mg, 49.9 µmol, 1.0 equiv). The tube was evacuated and backfilled with argon three times. Dry ethyl acetate (1.0 mL, 50 mM) followed by alkene **4a** (5.3 mg, 0.10 mmol, 2.0 equiv) was added under argon counter flow. The mixture was then

irradiated at 380 nm for 16 h. After that, the solvent was removed in vacuo and the residue was examined by HRMS and by ^{1}H NMR spectroscopy with mesitylene as internal standard.

The NMR yield of **5a** was determined to be 6%. Furthermore, BHT-trapped **IM2** (**S2**) and BHT-trapped **IM3** (**S3**) were detected by HRMS analysis (structures are reasonable structures based on HRMS).

5.5 Reaction in the absence of an alkene

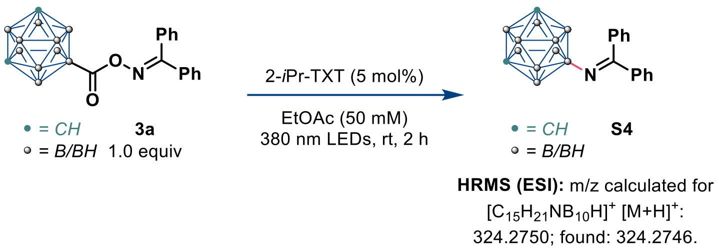

An oven-dried 10 mL Schlenk tube was charged with a Teflon-coated stirring bar, 2-isopropylthioxanthone (0.6 mg, 2 µmol, 5 mol%), and bifunctional reagent **3a** (18.4 mg, 0.0501 mmol, 1.0 equiv). The tube was evacuated and backfilled with argon three times. Dry ethyl acetate (1.0 mL, 50 mM) was added under argon counter flow. The mixture was then irradiated at 380 nm for 2 h. A sample of this mixture was examined by GC-MS and HRMS.

Crude GC-MS analysis (Supplementary Figure 15) showed imine dimer **7a**, *m*-carborane, benzophenone imine, and radical recombination product **S4**.

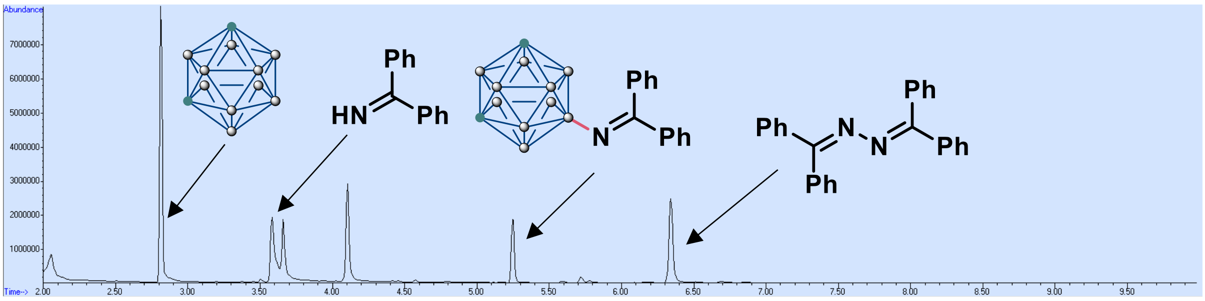

Supplementary Figure 15. GC-MS analysis of the crude reaction mixture of **S4**.

Compound **S4** was additionally detected by crude HRMS analysis. However, we were not able to isolate this compound.

Radical quenching of **IM1** and **IM2** by radical recombination seems to occur while being accompanied by quenching via HAT from e.g. the solvent and by quenching via dimerization of **IM2**.

5.6 EPR studies

Continuous-wave electron paramagnetic resonance (cw-EPR) experiments were performed on a Bruker EMXNano X-band spectrometer (9.63 GHz) at ambient temperature. All samples were prepared as dilute solutions in 4 mm o.d. clear fused quartz tubes, and EPR measurements were performed at a center field of 343 mT with a sweep range of 20 mT and sweep time of 20 s. A modulation amplitude of 0.04 mT at a modulation frequency of 100 kHz was employed. A microwave power of 1 mW was used in all experiments. The number of scans acquired as well as the receiver gain was optimized for each sample.

In situ UV/vis irradiation of the samples was achieved using a LOT LSH102 100 W Hg vapor lamp (wavelength range from 280 – 2500 nm) controlled by a LOT LSN161 power supply.

Spin trapping experiments were conducted using a degassed solution (PhCl or EtOAc) of **3a** (20 mM), 2-*i*Pr-TXT (5 mol% of **3a**), and *N*-*tert*-Bu-*α*-phenylnitrone (PBN) (10 mM) under an argon atmosphere. To ensure optimal EPR signal intensity, *in situ* UV/vis irradiation was performed while monitoring the intensity level by acquiring cw-EPR spectra in rapid succession (5 s per experiment). Irradiation was stopped as soon as no change in signal intensity could be observed for 5 consecutive scans. The durations of UV/vis irradiation as well as the number of scans are given in Supplementary Table 2.

Data acquisition was performed using the Bruker Xenon software. Iterative fitting of the cw-EPR spectra was performed using the EasySpin 6.0.6[17] MATLAB[18] package. These simulations utilized the built-in *esfit* and *garlic* functions to allow for fitting of isotropic liquid state cw-EPR spectra. The resulting EPR spectra were plotted using Matplotlib.[19]

For the reaction in PhCl, an EPR signal could be detected and by iterative fitting identified as **PBN1** (Supplementary Figure 16) through its coupling pattern**.** The spin system assumed for this fitting procedure consists of the ^{14}N and ^{1}H nuclei of PBN as well as the $^{10/11}B$ nucleus (in isotopic ratio) of the carborane. The resulting isotropic g values as well as hyperfine coupling constants are summarized in Supplementary Table 2. The reaction in EtOAc showed an additional, distinct EPR signal, which could be fitted by simultaneously using a spin system consisting of a ^{14}N and ^{1}H nucleus (Supplementary Figure 17). The two signals show an integral ratio of 1:1. No additional hyperfine coupling could be observed, and it is not possible to definitively assign this EPR signal to either **PBN2** or **PBN3**.

Because of the presence of a second species for the reaction in EtOAc, iterative fitting of two spin systems was performed on the cw-EPR spectrum of the reaction in PhCl. Convergence could only be achieved by inclusion of a second hyperfine coupling to a ^{14}N nucleus. It should be noted however, that despite a 1:1 integral ratio of these signals, the combination of hyperfine coupling

constants ($A_2(^{14}N) = 3A_2(^{14}N')$) and an increased line broadening lead to a near zero contribution in the observed first derivative spectrum. Because of this, a second spin system was not included for the reaction in PhCl, although its presence cannot be ruled out.

Supplementary Table 2. Fit parameters for the spin trapping experiments. Subscripts indicate separate spin systems used for iterative fitting of the spectra.

Solvent	EtOAc	PhCl
Number of scans	10	100
Duration of irradiation / min	2.5	4.5
g_1	2.006583(8)	2.006740(5)
$A_1(^{14}N)$ / mT	1.484(1)	1.492(1)
$A_1(^{1}H)$ / mT	0.1673(1)	0.1620(1)
$A_1(^{10/11}B)$ / mT	0.6087(1)	0.6108(1)
g_2	2.006861(7)	/
$A_2(^{14}N)$ / mT	1.371(1)	/
$A_2(^{1}H)$ / mT	0.2036(1)	/

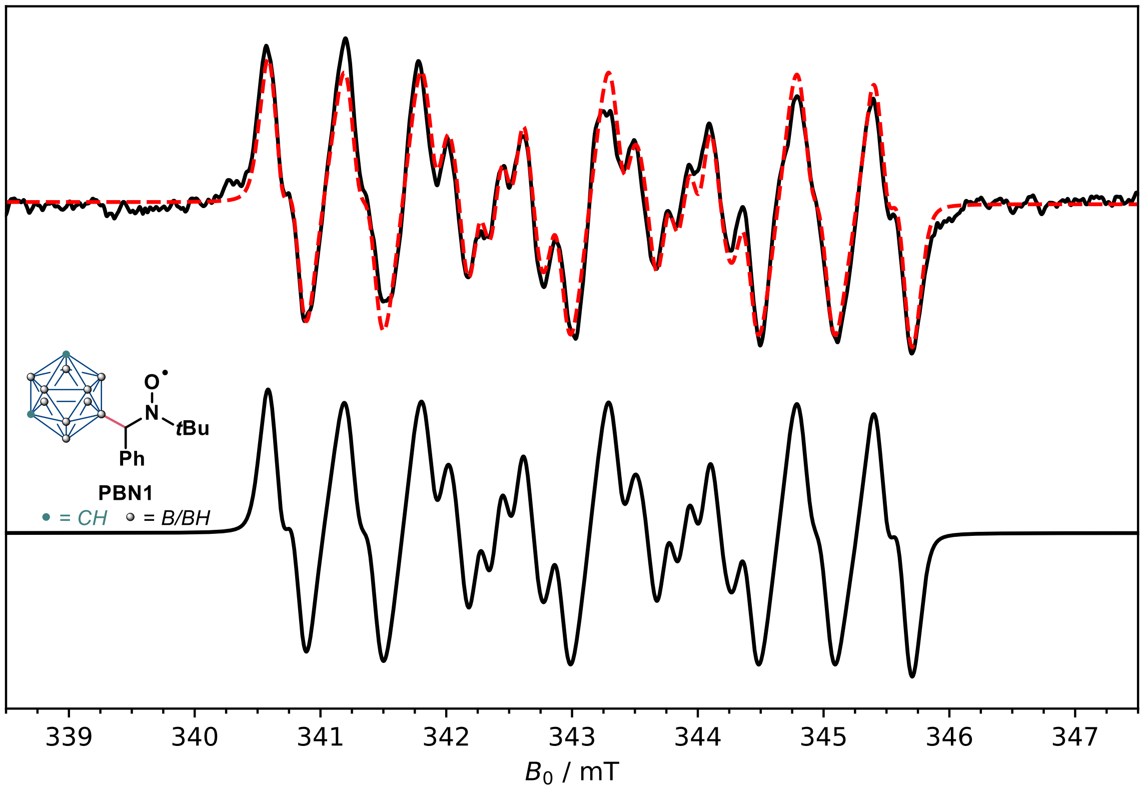

Supplementary Figure 16. cw-EPR spectrum (topmost solid black line) of the reaction of **3a** (20 mM) with PBN (10 mM) using 2-*i*Pr-TXT (5 mol% of **3a**) in PhCl after 4.5 min of UV/vis irradiation recorded using 100 scans and corresponding fit (red dashed line) as well as the fit (solid black line). The parameters of the deconvolution are given in Supplementary Table 2.

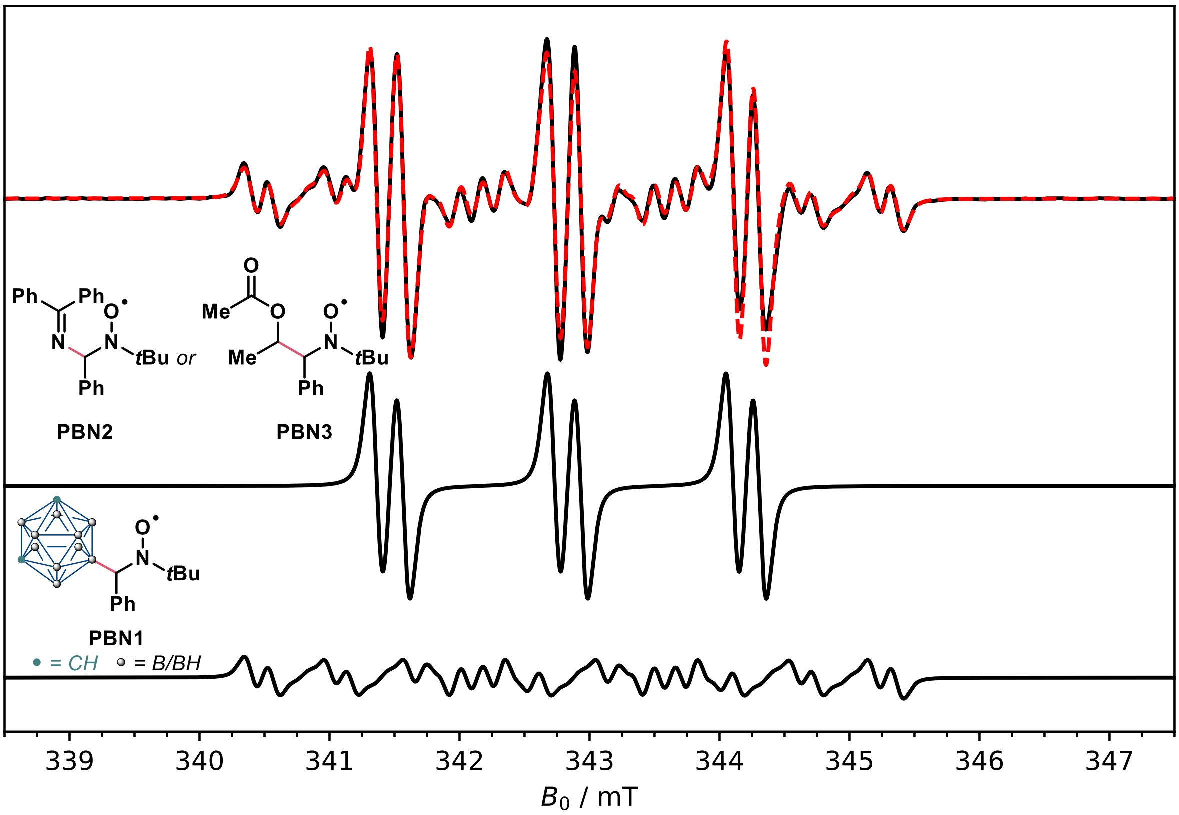

Supplementary Figure 17. cw-EPR spectrum (topmost solid black line) of the reaction of **3a** (20 mM) with PBN (10 mM) using 2-*i*Pr-TXT (5 mol% of **3a**) in EtOAc after 2.5 min of UV/vis irradiation recorded using 10 scans and corresponding sum of fits (red dashed line) as well as the components of the fit (solid black lines in the middle and on the bottom). The parameters of the deconvolution are given in Supplementary Table 2.

5.7 Evaluation of common radical starters AIBN and DTBP

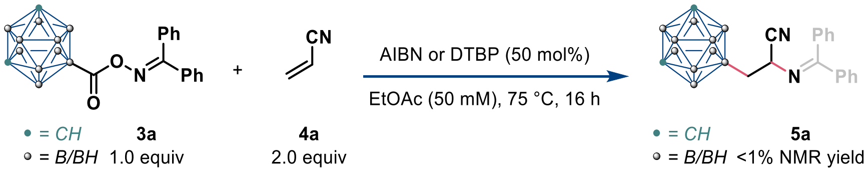

An oven-dried 10 mL Schlenk tube was charged with a Teflon-coated stirring bar, bifunctional reagent **3a** (18.4 mg, 0.0501 mmol, 1.0 equiv), and – in case of azobisisobutyronitrile (AIBN) – AIBN (4.1 mg, 0.025 mmol, 50 mol%). The tube was evacuated and backfilled with argon three times. Dry ethyl acetate (1.0 mL, 50 mM) followed by alkene **4a** (5.3 mg, 0.101 mmol, 2.0 equiv) and – in case of di-*tert*-butyl peroxide (DTBP) – DTBP (3.7 mg, 0.025 mmol, 50 mol%) was added under argon counter flow. The reaction mixture was then placed in a pre-heated heating block at 75°C and stirred for 16 h. After cooling to rt, the solvent was removed under reduced pressure and the residue was investigated by crude ^{1}H NMR spectroscopy with mesitylene as internal standard. The NMR yield of **5a** was determined to be <1% in both cases.

5.8 Quantum yield measurement

Measurement of the photon flux

The photon flux was determined by ferrioxalate actinometry similar to a procedure by Yoon.[20] A 3 W LED (λ_{max} = 395 nm) was used for the quantum yield measurement.

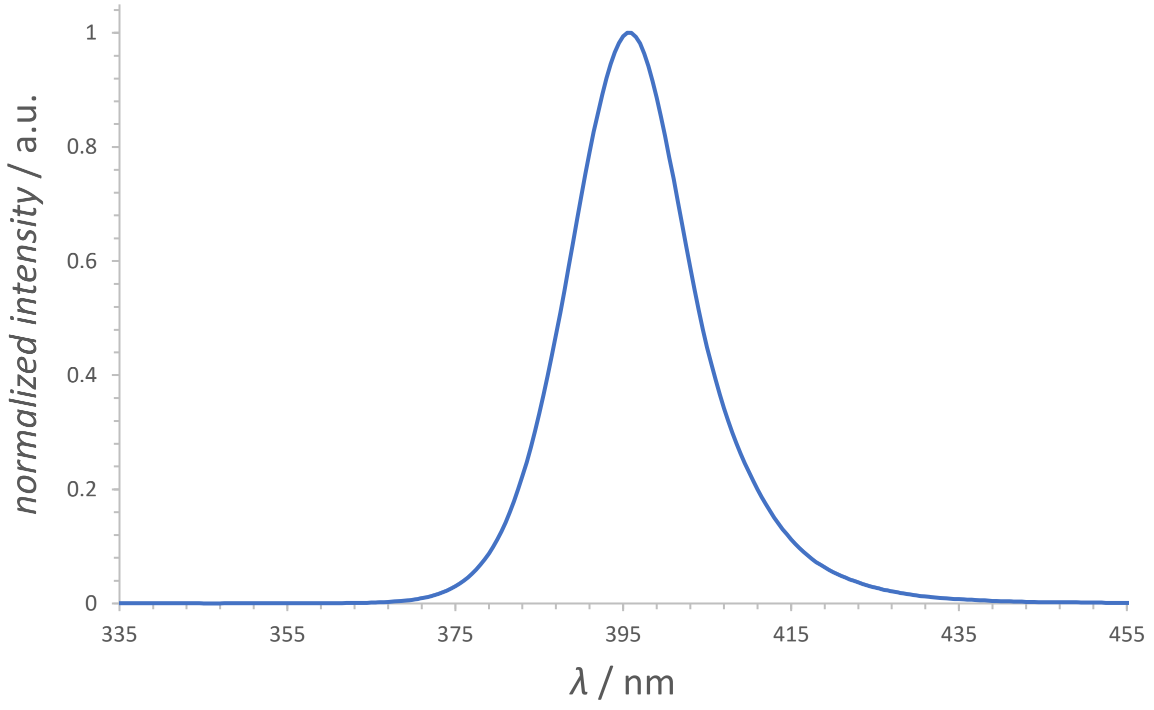

Supplementary Figure 18. Emission spectrum of the used 3 W LED (λ_{max} = 395 nm).

A solution (10 mL, 0.15 M) of potassium ferrioxalate hydrate (737 mg, 1.50 mmol) in aq. H_2SO_4 (50 mM) and a solution (20 mL, 5.0 mM) of 1,10-phenanthroline monohydrate (20 mg, 0.10 mmol) and sodium acetate (4.50 g) in aq. H_2SO_4 (500 mM) were prepared and kept in the dark. All following steps were conducted in the dark as well.

Six Schlenk tubes were charged with the prepared ferrioxalate solution (1.0 mL) and three of these tubes were successively irradiated with the described LED for 60 s at a distance of 5 cm, while three tubes were left in the dark. Then, to all six Schlenk tubes was added the prepared phenanthroline solution (175 µL each) and the reaction mixtures were stirred for 60 mins. For all six solutions, the absorbance at 510 nm was measured and the difference between the average absorbance of the three irradiated samples and the average absorbance of the three control samples was determined ($\Delta A_{510\ nm}$ = 2.023).

The formed amount of Fe(II) was calculated based on the Lambert–Beer law (equation 1) with V = 1.175 mL, l = 1.0 cm, and ε = 11100 L·mol^{-1}·cm^{-1}.[21,22]

$$n_{Fe(II)} = \frac{V \cdot \Delta A_{510\,\mathrm{nm}}}{l \cdot \varepsilon} \tag{1}$$

The fraction of light which was absorbed by the actinometer at λ = 395 nm (f) was determined with equation 2 with the absorbance of the ferrioxalate stock solution at λ = 395 nm being $A_{395\,\mathrm{nm}} > 3$ (f > 0.999).

$$f = 1 - 10^{-A_{395\,\mathrm{nm}}} \tag{2}$$

The photonflux ϕ_q was finally determined using equation 3 with ϕ_F = 1.13 (at λ = 392 nm) and t = 60 s, giving $\phi_\mathrm{q} = 3.159 \cdot 10^{-9}$ mol·s^{-1}.[23,24]

$$\phi_\mathrm{q} = \frac{n_{Fe(II)}}{\phi_\mathrm{F} \cdot t \cdot f} \tag{3}$$

Reaction quantum yield for the formation of 5a

An oven-dried 10 mL Schlenk tube was charged with a Teflon-coated stirring bar, 2-isopropylthioxanthone (1.3 mg, 5.1 µmol, 5 mol%), and bifunctional reagent **3a** (36.7 mg, 99.9 µmol, 1.0 equiv). The tube was evacuated and backfilled with argon three times. Dry ethyl acetate (1.0 mL, 0.10 M) followed by alkene **4a** (10.6 mg, 0.200 mmol, 2.0 equiv) was added under argon counter flow. The mixture was then irradiated at 395 nm for 420 s, using the described setup. After that, the solvent was removed in vacuo and the residue was examined by ^{1}H NMR spectroscopy with mesitylene as internal standard. The NMR yield was determined to be 4% (average of three reactions).

The reaction's quantum yield was determined using equation 4 with the determined photon flux ϕ_q, the irradiation time t = 420 s, and the fraction of light absorbed (f_R > 0.999; determined according to equation 2 with $A_{395\,\mathrm{nm}} > 3$) by the reaction mixture.

$$\phi = \frac{n_\mathrm{product}}{\phi_q \cdot t \cdot f_\mathrm{R}} \tag{4}$$

The determined quantum yield was ϕ = 3.01.

5.9 Cross-over experiment with a different imine dimer

Hypothesis and conclusion

We questioned, whether the commonly observed imine dimer **7a** side product (resulting from homo-recombination of two iminyl radicals) could reengage in the reaction and serve as an additional source of iminyl groups (Supplementary Figure 19).

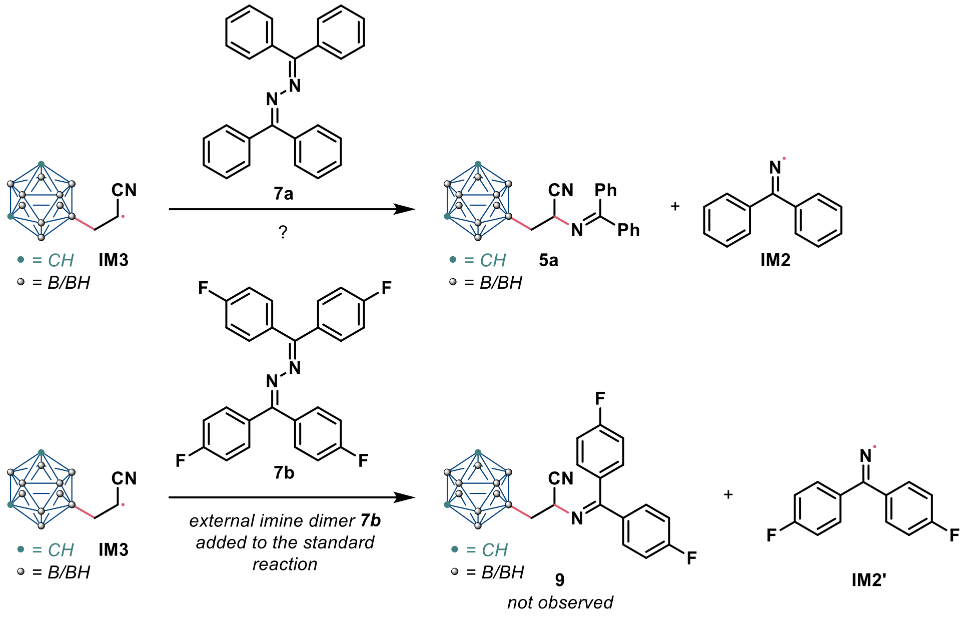

Supplementary Figure 19. Investigation whether imine dimer **7a** can reengage in the reaction as an iminyl group source.

To probe that question, we added a different imine dimer (**7b**) to the standard reaction mixture and searched for a cross-over product containing the fluorinated imine moiety (**9**). This compound was not observed and furthermore, imine dimer **7b** remained mostly untouched during the reaction. Consequently, we conclude that imine dimer side products do not participate in the reaction.

Synthesis of imine dimer 7b

Both procedures were carried out according to an adapted literature procedure.[25]

Bis(4-fluorophenyl)methanimine (S5)

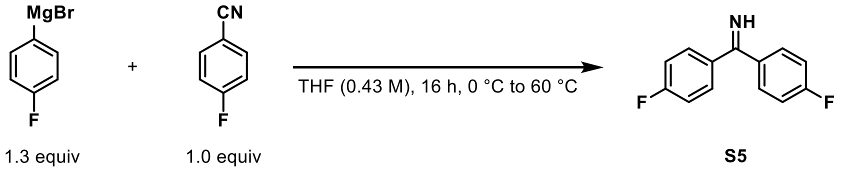

A Schlenk tube was charged with a magnetic stirring bar, and was then evacuated and backfilled with argon three times. A solution of 4-fluorophenylmagnesium bromide (1.0 M in THF, 6.5 mL, 6.5 mmol, 1.3 equiv) was added and the tube was cooled to 0 °C. Then, a solution of 4-fluorobenzonitrile (605 mg, 5.00 mmol, 1.0 equiv) in THF (5.0 mL, in total 0.43 M) was added dropwise and the mixture was stirred at 60 °C for 16 h. The tube was then cooled to 0 °C, and the reaction was quenched by addition of MeOH (1.0 mL) and diluted with *n*-pentane (10 mL). Filtration through Celite, rinsing the Celite pad with *n*-pentane (10 mL), and concentrating gave a residue which was further purified by column chromatography on deactivated silica (*n*-pentane + 5% NEt_3 to *n*-pentane/ethyl acetate = 10:1 + 5% NEt_3) to give a yellow oil (969 mg, 4.46 mmol, 89%).

^{1}H NMR (400 MHz, $CDCl_3$) δ 7.61 – 7.50 (m, 4H), 7.15 – 7.07 (m, 4H).

^{13}C{^{1}H} NMR (101 MHz, $CDCl_3$) δ 176.2, 164.3 (d, *J* = 251.0 Hz), 135.4, 130.6 (d, *J* = 8.6 Hz), 115.6 (d, *J* = 21.8 Hz).

^{19}F NMR (376 MHz, $CDCl_3$) δ -109.8.

HRMS (ESI): *m/z* calculated for $[C_{13}H_9NF_2H]^+$ $[M+H]^+$: 218.0776; found: 218.0774.

1,2-Bis(bis(4-fluorophenyl)methylene)hydrazine (7b)

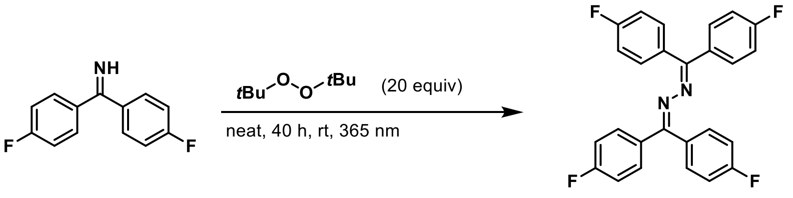

A Schlenk tube was charged with a magnetic stirring bar and imine **S5** (130 mg, 0.598 mmol, 1.0 equiv). The tube was evacuated and backfilled with argon three times and was then charged with di-*tert*-butyl peroxide (1.75 g, 12.0 mmol, 20 equiv). After irradiating at 365 nm for 40 h, the peroxide was removed in vacuo and the residue was washed with *n*-pentane (3 x 4 mL). Drying in vacuo gave the desired product as a bright yellow solid (83.5 mg, 0.193 mmol, 65%).

^{1}H NMR (400 MHz, $CDCl_3$) δ 7.50 – 7.43 (m, 4H), 7.35 – 7.27 (m, 4H), 7.16 – 7.07 (m, 4H), 7.04 – 6.96 (m, 4H).

^{13}C{^{1}H} NMR (101 MHz, $CDCl_3$) δ 164.1 (d, *J* = 250.9 Hz), 163.0 (d, *J* = 249.3 Hz), 159.0, 134.2 (d, *J* = 3.2 Hz), 131.6 (d, *J* = 8.3 Hz), 131.2 (d, *J* = 3.6 Hz), 130.8 (d, *J* = 8.5 Hz), 115.4 (d, *J* = 21.7 Hz), 115.3 (d, *J* = 21.4 Hz).

$^{19}F\{^1H\}$ **NMR** (376 MHz, $CDCl_3$) δ -110.5, -111.3.

HRMS (ESI): *m/z* calculated for $[C_{26}H_{16}N_2F_4Na]^+$ $[M+Na]^+$: 455.1142; found: 455.1141.

Cross-over experiment

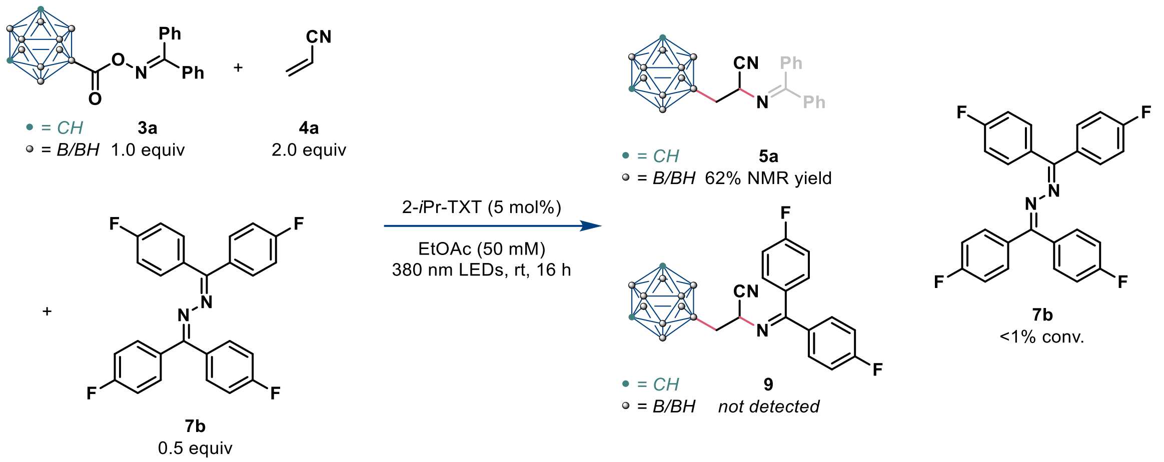

An oven-dried 10 mL Schlenk tube was charged with a Teflon-coated stirring bar, 2-isopropylthioxanthone (1.3 mg, 5.1 µmol, 5 mol%), bifunctional reagent **3a** (36.7 mg, 0.0999 mmol, 1.0 equiv), and imine dimer **7b** (21.6 mg, 0.0500 mmol, 0.50 equiv). The tube was evacuated and backfilled with argon three times. Dry ethyl acetate (2.0 mL, 50 mM) followed by alkene **4a** (10.6 mg, 0.200 mmol, 2.0 equiv) was added under argon counter flow. The mixture was then irradiated at 380 nm for 16 h. After that, the solvent was removed in vacuo and the residue was examined by HRMS and ^1H and ^{19}F NMR spectroscopy with mesitylene as internal standard.

Crude HRMS analysis did not detect the cross-over product **9**.

^{19}F NMR spectroscopy (Supplementary Figure 20) showed no significant new species and ^1H NMR spectroscopy (Supplementary Figure 21) indicated 62% NMR yield of product **5a**, as well as <1% conversion of imine dimer **7b**.

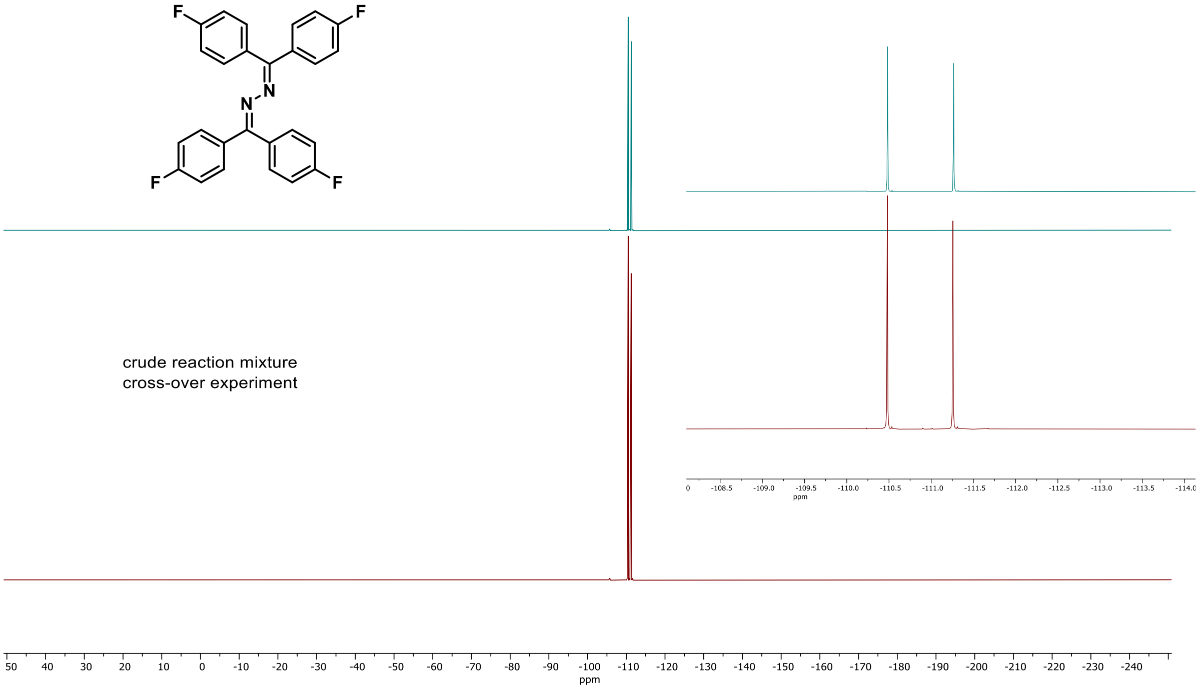

Supplementary Figure 20. ^{19}F NMR spectrum of the isolated imine dimer **7b** (upper spectrum) and the crude reaction mixture (lower spectrum).

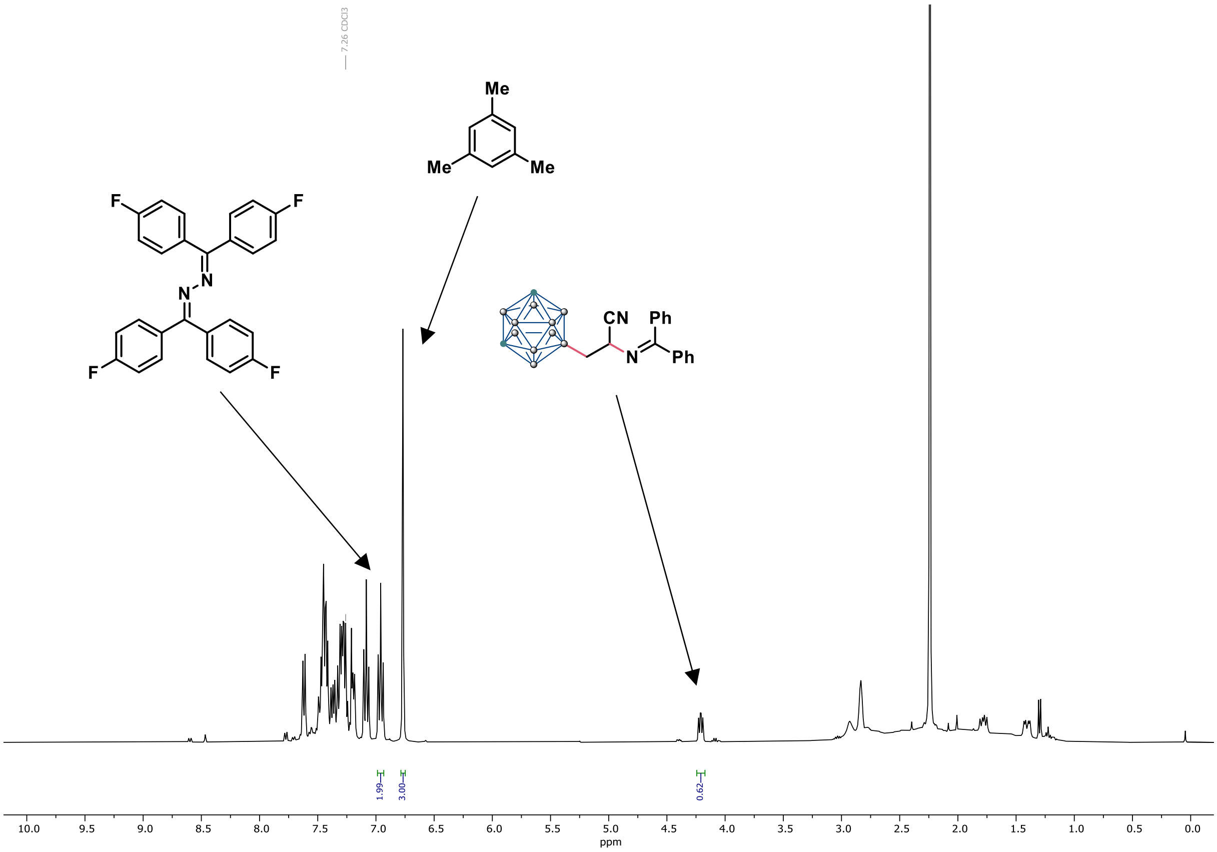

Supplementary Figure 21. ^{1}H NMR spectrum of the crude reaction mixture of the cross-over experiment.

5.10 The carborane's effect on the photophysical properties of reagents 3

5.10.1 UV/vis analysis

UV/vis absorption spectroscopy measurements were performed on a Jasco V-730 spectrophotometer, equipped with a temperature control unit at 25 °C. The samples were measured in Starna® fluorescence quartz cuvettes (type: 29-F, chamber volume = 1.400 mL, H × W × D = 48 mm × 12.5 mm × 12.5 mm, path length = 10 mm). The spectra were recorded using the following parameters: response time = 0.06 sec, data interval = 0.2 nm, scan speed = 400 nm/min. Solutions of **3a** (5 mM in EtOAc) and of **S6** (5 mM in EtOAc) were measured. **S6** was used as previously prepared by us.[24]

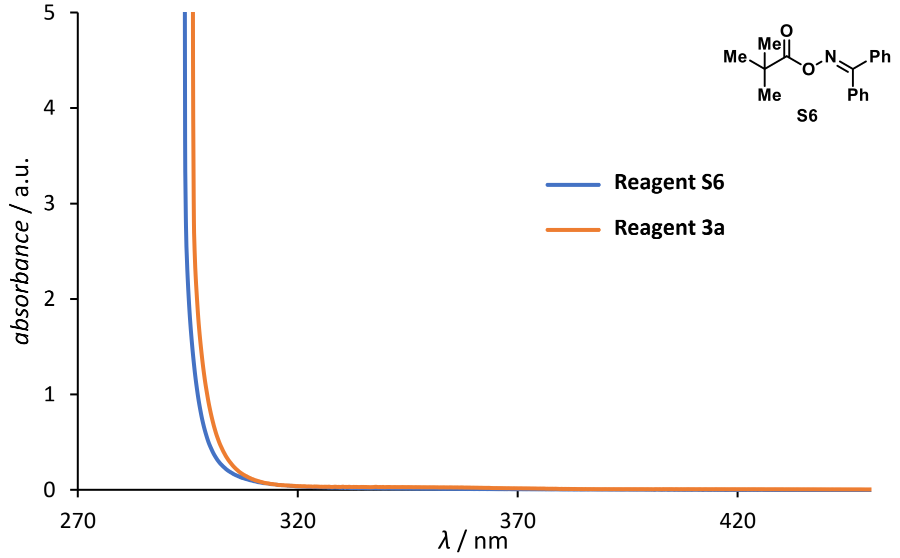

Supplementary Figure 22. UV/vis absorption spectra of **3a** (5 mM in EtOAc, orange) and **S6** (5 mM in EtOAc, blue).

Both compounds show overall similar UV/vis spectra with a slight red-shift of the absorption of **3a** compared to **S6**.

5.10.2 Computational studies

All computations were done using the ORCA 6.0.1 software package.[26,27] First, a conformational search was carried out using the GOAT implementation at the GFN2-xTB level.[28] The lowest energy conformer obtained was then considered for further geometry optimization. Geometry optimization of all relevant stationary points was performed using the CAM-B3LYP range-separated hybrid functional[29] on a recontracted Ahlrichs double-ζ-basis (def2-SVP)[30] applying the CPCM continuum solvation model[31,32] for ethyl acetate and a Grimme D3 atom-pairwise

dispersion correction.[33,34] All optimized geometries were confirmed to be local minima on the respective potential energy surface by the absence of imaginary frequencies, as obtained from the vibrational frequency calculations at the same level of theory.

The electronic energy of the previously optimized geometries was then refined through an additional single point calculation on the CAM-B3LYP / def2-TZVPP level,[29,30] with the CPCM continuum solvation model for ethyl acetate and a Grimme D3 atom-pairwise dispersion correction. The reported Gibbs free energies at 298 K were obtained as the sum of the electronic energies and the corresponding free energy corrections (ZPVE, thermal corrections, enthalpy correction, entropic corrections), as obtained from the frequency calculation. The triplet energies were determined by calculating the difference between the Gibbs free energies of the triplet and the singlet states:

$$E_T = \Delta G = G_{Triplet} - G_{Singlet}$$

Supplementary Table 3. Summary of electronic energies and free energy corrections.

Name	Electronic energy / E_h	ZPVE / E_h	Thermal correction / E_h	Enthalpy correction / E_h	Entropy correction / E_h
**^{1}3a**	-1076.026236	0.38077023	0.02169143	0.00094421	-0.07009364
**^{3}3a**	-1075.942187	0.37783699	0.02183821	0.00094421	-0.0711712
**^{1}3c**	-1076.008679	0.37940175	0.02154746	0.00094421	-0.06957393
**^{3}3c**	-1075.920352	0.37629568	0.02166239	0.00094421	-0.07044095
**^{1}S6**	-902.4448332	0.3320884	0.0194559	0.00094421	-0.06621964
**^{3}S6**	-902.3584857	0.32914384	0.019561	0.00094421	-0.06714717

Supplementary Table 4. Summary of triplet energies.

Name	Triplet energy / E_h	Triplet energy / (kcal/mol)
**^{3}3a**	0.080185618	50.3172373
**^{3}3c**	0.084468901	53.0050377
**^{3}S6**	0.082580507	51.8200528

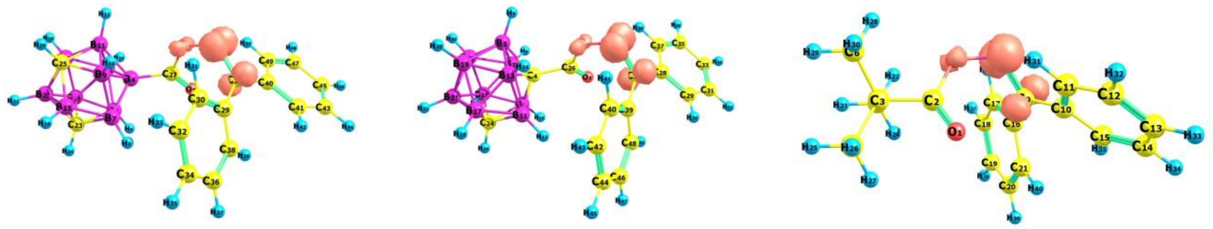

^{3}**3a** ^{3}**3c** ^{3}**S6**

Supplementary Figure 23. Triplet state spin density plots for **3a**, **3c**, and **S6**.

The triplet energies of compounds **3a**, **3c**, and **S6** are in a similar range, not indicating a major influence of the carboranyl moiety compared to a *tert*-butyl group. In the triplet state, the spin density is mainly located on the oxime fragment without relevant involvement of the carboranyl or alkyl group.

Cartesian coordinates

^{1}**3a**

50

N	4.42744505	9.381481748	6.070927708
O	5.41566341	8.567676865	6.657623517
O	5.97925537	10.27641649	8.003229947
B	7.238793	8.125882063	8.222575671
B	6.93944223	7.302674954	9.754323109
H	5.98659148	7.449840352	10.4574987
B	8.15514073	8.592292499	9.665642853
H	8.02797538	9.592760548	10.30068386
B	8.97169799	8.457571545	8.104375089
H	9.48966403	9.352273982	7.508968324
B	8.26047007	7.078012312	7.235091491
H	8.30894625	7.05973805	6.04267685
B	7.0007124	6.362561379	8.250487034
H	6.028389	5.843385227	7.78901838
B	9.73347079	7.830171467	9.558790973
H	10.7328434	8.255697996	10.04456816
B	8.60026921	5.606451518	8.156993152
H	8.88729146	4.588307246	7.604082692
B	7.77887314	5.745746342	9.716235998
H	7.41419121	4.835921438	10.39747406
B	9.50247594	6.074081571	9.590636105
H	10.3621624	5.426253433	10.09787991
C	8.48437698	7.117180262	10.43677726

H	8.57600208	7.125857185	11.52215612
C	9.68660503	6.91699802	8.141605094
H	10.6270155	6.786219849	7.607589446
C	6.16364112	9.144967689	7.631666168
C	3.34012559	9.407722126	6.746773768
C	3.12737503	8.654833915	8.015040897
C	3.1414896	7.258095045	8.025954627
H	3.30521238	6.711525681	7.095877561
C	2.950003	6.568744166	9.21922206
H	2.95848298	5.476941235	9.220577671
C	2.75674433	7.269155636	10.40824273
H	2.61276803	6.726518248	11.34497258
C	2.74762646	8.662415133	10.40100588
H	2.59952776	9.215243341	11.33082656
C	2.92599154	9.354769685	9.206838623
H	2.9199713	10.44644816	9.198369362
C	2.24610509	10.25695154	6.213227018
C	2.52893502	11.28863063	5.308055726
H	3.56457319	11.47171611	5.020867467
C	1.50257307	12.07117893	4.795287645
H	1.73325612	12.87748201	4.095983829
C	0.18186481	11.83355345	5.177770926
H	-0.6236248	12.4516891	4.775364205
C	-0.1064927	10.81135896	6.077877809
H	-1.1382152	10.62080664	6.380171029
C	0.92062937	10.02829555	6.597584932
H	0.68869341	9.22773268	7.30122946

^{3}3a

50

N	4.02324279	6.996098737	9.997684832
O	4.36640681	8.29702523	9.787803729
O	3.5907974	8.479566769	7.673878773
B	5.36455876	10.08949414	8.410287408
B	5.56582171	10.92702378	6.867789646
H	5.00964051	10.66165134	5.846612672
B	6.83600969	9.836502333	7.462764773
H	7.1327342	8.853885794	6.855223439
B	6.93800592	10.03055582	9.221051317
H	7.31801661	9.201817821	9.988073944
B	5.72631752	11.2302194	9.706641262
H	5.29037529	11.20184451	10.81684494

B	4.86607416	11.78407596	8.252339457
H	3.7168521	12.115071	8.281204722
B	8.09607608	10.83268241	8.174667108
H	9.2607222	10.59359258	8.134392052
B	6.14990149	12.77891139	8.965106706
H	6.01834763	13.80109872	9.566658253
B	6.052874	12.59103493	7.208434228
H	5.84408511	13.46346537	6.421507668
B	7.61246145	12.53171751	8.017693179
H	8.48284652	13.33146247	7.882090911
C	7.19809615	11.38260771	6.855804159
H	7.71188494	11.43859109	5.896839425
C	7.3455176	11.66326282	9.436124521
H	7.96254974	11.91882529	10.2966811
C	4.32420606	8.88918152	8.525429417
C	4.0829206	6.145359942	8.871601027
C	5.41614398	5.877063279	8.360487001
C	6.54918277	6.117608981	9.168108062
H	6.41130069	6.431617223	10.20334876
C	7.83266721	5.949731132	8.66678885
H	8.69150612	6.134138552	9.315698419
C	8.02772606	5.550768819	7.344606684
H	9.03793441	5.424283184	6.950139233
C	6.91940037	5.326523528	6.526680004
H	7.06000278	5.035789895	5.483362657
C	5.63233656	5.4874761	7.02123944
H	4.78180677	5.352077066	6.353968245
C	2.83986584	5.56128236	8.424184807
C	2.79178865	4.326661768	7.741857478
H	3.71332614	3.780237612	7.543076656
C	1.57828211	3.776551718	7.34982044
H	1.56812644	2.817990021	6.826369035
C	0.37921047	4.431005808	7.632055829
H	-0.5728066	3.995426188	7.321901201
C	0.40721083	5.642339309	8.327030133
H	-0.5258104	6.159823026	8.560725606
C	1.614265	6.197128382	8.723319249
H	1.62221076	7.146079615	9.260701533

[1]3c

50

N	4.19652454	8.856443721	6.12756409
O	5.22807037	8.121695061	6.759606302

O	6.20116424	10.05956625	7.349361519
C	7.24555711	7.98378338	7.940995022
B	6.91660736	6.342293259	8.299519888
H	5.85103351	5.887491935	8.024940763
B	7.02827271	7.565676519	9.583092439
H	6.05051252	7.946603962	10.14887247
B	8.23191441	8.755792049	9.085088601
H	8.17737713	9.917929104	9.321939904
B	8.84742657	8.285490427	7.488172364
H	9.16058566	9.162707541	6.74883015
B	8.03105721	6.797143167	6.997887129
H	7.72546178	6.667266041	5.852223646
B	8.66998435	7.506486308	10.23497329
H	8.99397579	7.833950876	11.33603803
B	9.67099069	6.743200289	7.653214709
H	10.6669152	6.558250977	7.02187578
B	8.47801497	5.530430292	8.155700078
H	8.57089426	4.386715888	7.814736523
B	9.55983503	6.255491977	9.352988614
H	10.4985137	5.729725017	9.871631149
B	7.8542273	6.006240565	9.756444294
C	9.66121485	7.891670162	8.907676654
H	10.5872137	8.42916162	9.108190294
C	6.18514433	8.86224055	7.315879506
C	3.24082873	9.132348384	6.935552007
C	3.21619752	8.721794755	8.367273373
C	3.12199962	7.373022323	8.719257767
H	3.06776517	6.611597507	7.939392653
C	3.09484302	7.00603623	10.0606559
H	3.0160501	5.951317193	10.33127037
C	3.1777169	7.979773911	11.05411032
H	3.1638621	7.688380918	12.10637708
C	3.28033614	9.324238236	10.70471791
H	3.34947015	10.08945687	11.48034949
C	3.29173226	9.697748446	9.363730121
H	3.3668174	10.75118554	9.086776118
C	2.11449086	9.921928525	6.383642986
C	2.28289742	10.6752768	5.213820893
H	3.25805188	10.69032554	4.726673452
C	1.22103888	11.40295361	4.693333913
H	1.36173518	11.99352459	3.785866768
C	-0.0196466	11.38601618	5.331747166
H	-0.8526664	11.96117335	4.921832752
C	-0.1931998	10.64080608	6.494924645

H	-1.1622713	10.62463375	6.997406738
C	0.87009336	9.914319816	7.023035844
H	0.72952713	9.330815582	7.933678737
H	7.49613092	5.204578951	10.57027978

³3c

50

N	4.80820182	6.725274587	10.03180339
O	5.91327566	7.396711803	9.559855321
O	5.00527907	9.456972523	9.370231722
C	6.93717008	8.908866574	8.06052697
B	8.54464976	8.453837511	8.406601403
H	8.77955446	7.923551903	9.449067351
B	8.04474847	10.16454861	8.403410074
H	7.93326813	10.76515547	9.427919185
B	6.79494737	10.35099959	7.176692658
H	5.85935347	11.07758583	7.257681875
B	6.50614669	8.758017092	6.429111536
H	5.39557322	8.524779212	6.073354519
B	7.59141219	7.58891962	7.185572251
H	7.18493653	6.489047356	7.390223009
B	8.44155873	10.83608059	6.818453387
H	8.6591005	11.98582134	6.584767396
B	7.98087251	8.271009007	5.608837456
H	7.89048993	7.70193465	4.563809807
B	9.25407229	8.076799185	6.830049283
H	10.1223201	7.263220599	6.702100183
B	9.18119184	9.549966173	5.854097065
H	9.91241549	9.843870301	4.956942987
B	9.53563397	9.667555993	7.585380549
C	7.52280888	9.904851475	5.73121531
H	7.15971112	10.39674698	4.829480922
C	5.83554439	8.661341865	9.059073344
C	3.56568136	7.187003874	9.545052867
C	2.66213999	7.777295756	10.4998065
C	1.26737557	7.821570114	10.28240979
H	0.84576992	7.395109443	9.37273823
C	0.41798402	8.371801501	11.23260449
H	-0.6574361	8.391289656	11.04349047
C	0.92665722	8.884024876	12.42660121
H	0.25472779	9.315470533	13.17119055
C	2.30146873	8.826801903	12.6678595

H	2.70891869	9.215655488	13.60351798
C	3.1567535	8.277329769	11.72605948
H	4.22809608	8.238215488	11.9281286
C	3.32290132	6.929578014	8.132218621
C	4.01834823	5.897440106	7.467826951
H	4.68080248	5.243928839	8.037202172
C	3.86055788	5.696513355	6.103441051
H	4.4024662	4.885391821	5.612787617
C	3.01857848	6.524832724	5.361835847
H	2.90044941	6.369391801	4.28760825
C	2.3377133	7.561446994	6.000588603
H	1.6933917	8.229179208	5.424807233
C	2.48577373	7.766398826	7.365923901
H	1.98198322	8.608454016	7.839879279
H	10.6069506	9.999110042	8.003311835

[1]S6

40

O	2.45098207	2.239122556	-0.250218219
C	2.31691635	1.06886844	-0.034315988
C	3.42740317	0.066558931	0.256778657
C	3.42140739	-1.004624775	-0.844759928
C	4.76086776	0.811243305	0.26649541
C	3.17006563	-0.585936799	1.623288228
O	1.11336192	0.440095506	-0.019355508
N	0.02859753	1.245419378	-0.328480602
C	-1.0774611	0.606922001	-0.242634016
C	-1.1912155	-0.808826938	0.206135418
C	-1.7079326	-1.774657874	-0.660836341
C	-1.8068104	-3.101961418	-0.251259232
C	-1.407067	-3.4683786	1.031653943
C	-0.9036969	-2.504746007	1.904410194
C	-0.7898828	-1.181026688	1.492459657
C	-2.3008271	1.355732636	-0.627786182
C	-2.2057928	2.511215549	-1.415950557
C	-3.3476586	3.223384177	-1.758505397
C	-4.6007896	2.794176988	-1.319385219
C	-4.7037768	1.648017488	-0.536363854
C	-3.5608153	0.930003684	-0.19369949
H	3.57743755	-0.554215943	-1.836649335
H	2.47147569	-1.556015314	-0.859663374
H	4.23605119	-1.721417377	-0.663614907

H	4.77403905	1.591044747	1.040996889
H	4.95321218	1.293768559	-0.701994274
H	5.57770883	0.10410135	0.471270254
H	3.98744171	-1.285237565	1.853713162
H	3.13195025	0.168954504	2.423211373
H	2.22506427	-1.145613457	1.628892074
H	-2.0303631	-1.485832434	-1.663454143
H	-2.2025698	-3.852814136	-0.938176856
H	-1.4905076	-4.508228736	1.354739695
H	-0.5938552	-2.786518128	2.912875377
H	-0.3884357	-0.427397267	2.17190751
H	-1.2240175	2.838740085	-1.758769683
H	-3.2619015	4.11881274	-2.377650164
H	-5.4978057	3.3543693	-1.591955697
H	-5.6806942	1.307444747	-0.187195492
H	-3.6501459	0.035432799	0.423856613

[3]S6

40

O	0.35499828	1.487180057	0.107389132
C	1.38121217	0.888207902	-0.008892107
C	2.52726741	1.197194549	-0.954252529
C	2.61827223	0.043033749	-1.966677749
C	2.21781525	2.510752994	-1.670179539
C	3.83642656	1.311650582	-0.160319396
O	1.67157712	-0.235569919	0.746718618
N	0.72018297	-0.964532462	1.395602014
C	-0.6169426	-0.810158224	0.976666236
C	-1.5217857	-0.161808297	1.898712306
C	-1.0139273	0.712761156	2.885076569
C	-1.8575703	1.326714438	3.79800048
C	-3.2321865	1.081538534	3.76657743
C	-3.748362	0.201577314	2.815257006
C	-2.9097162	-0.416844915	1.896903733
C	-0.955401	-1.45115986	-0.283836142
C	-0.1386892	-2.480498461	-0.799388252
C	-0.4109004	-3.057067192	-2.032436996
C	-1.4943461	-2.617477845	-2.79341345
C	-2.2995078	-1.586728854	-2.307573315
C	-2.0368171	-1.007468591	-1.073226616
H	2.84799175	-0.908674628	-1.468486888
H	3.41835184	0.258684168	-2.689802727

H	1.67536631	-0.074790911	-2.521230719
H	3.03040318	2.745857731	-2.372279106
H	2.12574627	3.340839379	-0.955314457
H	1.27817594	2.442776454	-2.235694667
H	4.07940692	0.371044607	0.350913276
H	4.65688386	1.552946722	-0.851691125
H	3.77584759	2.112726206	0.591415503
H	0.05739952	0.914871133	2.920091972
H	-1.4406746	2.00653894	4.544289108
H	-3.8948988	1.565936609	4.486590693
H	-4.818641	-0.015298793	2.794873256
H	-3.330123	-1.127786198	1.186386639
H	0.70978807	-2.833981236	-0.211856237
H	0.22923501	-3.859987248	-2.404331015
H	-1.7051612	-3.069527768	-3.764712449
H	-3.1365328	-1.219012719	-2.905099796
H	-2.6501948	-0.174499098	-0.730738694

6. Product diversifications

6.1 Synthesis of carboranyl analogs of known biologically active β-arylethylamines

Amino acid hydrochloride 10

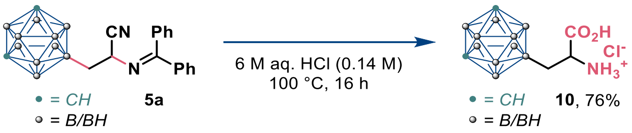

To a 10 mL Schlenk tube was added a magnetic stirring bar, compound **5a** (18.8 mg, 49.9 µmol, 1.0 equiv), and aq. HCl (6.0 M; 0.35 mL, 0.14 M). The mixture was stirred under air at 100 °C for 16 h. The reaction mixture was washed with *n*-pentane (3 x 5 mL), and the aqueous layer was subsequently evaporated and dried under vacuum to obtain the desired product as a white solid (10.1 mg, 37.7 µmol, 76%).

^{1}H NMR (400 MHz, D_2O) δ 3.95 (t, J = 6.7 Hz, 1H), 3.43 (s, 2H), 1.69 (dd, J = 15.6, 6.4 Hz, 1H), 1.48 (dd, J = 15.7, 7.0 Hz, 1H), 3.17 – 1.34 (m, 9H).

^{13}C{^{1}H} NMR (101 MHz, D_2O) δ 173.9, 55.7, 54.3, 17.2.

^{11}B{^{1}H} NMR (128 MHz, D_2O) δ -3.0, -6.9, -10.6, -13.1, -13.7, -17.1, -19.3.

HRMS (ESI): *m/z* calculated for $[C_5H_{17}B_{10}NO_2Na]^+$ $[M\text{-}HCl+Na^+]^+$: 254.2157; found: 254.2155.

Amino phosphonic acid hydrochloride 11

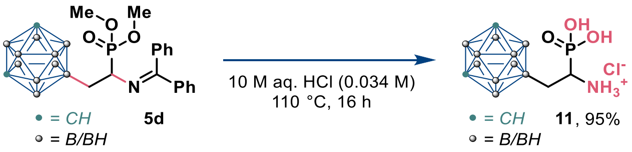

To a 10 mL Schlenk tube was added a magnetic stirring bar, compound **5d** (18.5 mg, 40.3 µmol, 1.0 equiv), and aq. HCl (10 M; 1.2 mL, 0.034 M). The mixture was stirred under air at 110 °C for 16 h. The reaction mixture was washed with *n*-pentane (3 x 5 mL), and the aqueous layer was subsequently evaporated and dried under vacuum to obtain the desired product as a white solid (11.6 mg, 38.2 µmol, 95%).

^{1}H NMR (400 MHz, MeOH-d_4) δ 3.60 (s, 2H), 3.32 – 3.23 (m, 1H), 1.77 (td, J = 15.1, 4.2 Hz, 1H), 1.30 – 1.17 (m, 1H), 4.01 – 0.49 (m, 9H).

^{13}C{^{1}H} NMR (151 MHz, MeOH-d_4) δ 57.0, 56.9, 50.3 (d, J = 153.0 Hz), 16.0.

^{11}B{^{1}H} NMR (128 MHz, MeOH-d_4) δ -1.7, -6.6, -10.1, -12.9, -13.6, -17.1, -19.5.

^{31}**P NMR** (162 MHz, MeOH-d$_4$) δ 17.1.

HRMS (ESI): *m/z* calculated for $[C_4H_{18}NO_3PB_{10}Na]^+$ $[M\text{-}HCl+Na^+]^+$: 290.1922; found: 290.1918.

Amine 5n'

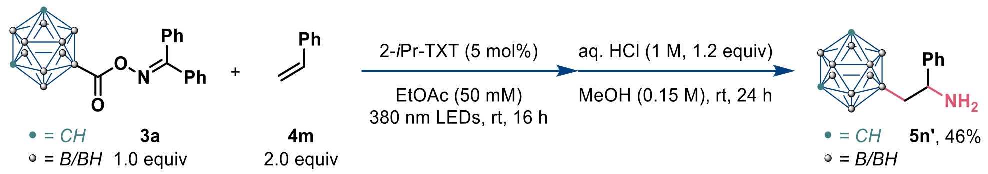

Two oven-dried 10 mL Schlenk tubes were each charged with a Teflon-coated stirring bar, 2-*i*Pr-TXT (3.3 mg, 13 µmol, 5 mol%), and bifunctional reagent **3a** (92.0 mg, 0.250 mmol, 1.0 equiv). The tubes were evacuated and backfilled with argon three times. Dry ethyl acetate (5.0 mL, 50 mM) followed by alkene **4m** (52.2 mg, 0.501 mmol, 2.0 equiv) was added under argon counter flow to each tube. The mixtures were then irradiated at 380 nm for 16 h. After that, the mixtures were combined, the solvent was removed, and the product was preliminary purified by column chromatography on deactivated silica (*n*-pentane/ethyl acetate = 100:1).

After evaporation and drying, a 20 mL vial was charged with a magnetic stirring bar, the obtained crude product, methanol (3.3 mL, 0.15 M), and aq. HCl (1.0 M; 580 µL, 0.580 mmol, 1.2 equiv). The mixture was stirred at room temperature for 24 h, and was then basified using aq. NaOH (1.0 M). The mixture was then extracted with CH_2Cl_2 (3 x 5 mL), the combined organic layers were dried using Na_2SO_4, and the solvent was removed. Column chromatography (CH_2Cl_2 +1% MeOH +1% NEt_3) on deactivated silica gave the title compound as a grey oil (60.8 mg, 0.231 mmol, 46%).

^{1}**H NMR** (400 MHz, $CDCl_3$) δ 7.39 – 7.34 (m, 2H), 7.34 – 7.27 (m, 2H), 7.25 – 7.18 (m, 1H), 4.01 (dd, *J* = 7.9, 6.1 Hz, 1H), 2.85 (s, 2H), 2.42 (s, 2H), 1.50 (dd, *J* = 15.0, 6.1 Hz, 1H), 1.39 (dd, *J* = 14.9, 7.9 Hz, 1H), 3.39 – 1.23 (m, 9H).

^{13}**C{^{1}H} NMR** (101 MHz, $CDCl_3$) δ 146.2, 128.5, 127.2, 127.1, 56.8, 54.5, 25.8.

^{11}**B{^{1}H} NMR** (128 MHz, $CDCl_3$) δ -0.7, -6.4, -9.9, -13.2, -13.9, -17.6, -19.9.

HRMS (ESI): *m/z* calculated for $[C_{10}H_{21}NB_{10}H]^+$ $[M+H]^+$: 264.2755; found: 264.2749.

Amine 12

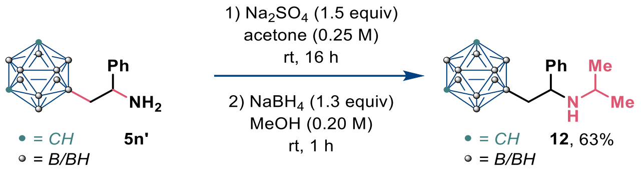

To a 10 mL Schlenk tube was added a magnetic stirring bar and compound **5n'** (26.3 mg, 99.9 μmol, 1.0 equiv). The tube was evacuated and backfilled with argon three times. Then, dry acetone (0.40 mL, 0.25 M) and Na_2SO_4 (21.3 mg, 0.150 mmol, 1.5 equiv) were added and the mixture was stirred at rt for 16 h. Filtration through Celite, washing of the pad with acetone (0.5 mL), and concentrating gave the crude imine, which was then transferred to another Schlenk tube. The tube was evacuated and backfilled with argon three times and then, dry MeOH (0.50 mL, 0.20 M) was added, followed by $NaBH_4$ (4.9 mg, 0.130 mmol, 1.3 equiv). After stirring at rt for 1 h, 3 drops of water were added, the mixture was filtered through silica, the pad was washed with ethyl acetate (3 mL), and the solution was concentrated. Column chromatography on deactivated silica (CH_2Cl_2 + 0.5% MeOH to CH_2Cl_2 + 1% MeOH) gave the title compound as a colorless oil (19.1 mg, 62.5 μmol, 63%).

^{1}H NMR (400 MHz, $CDCl_3$) δ 7.38 – 7.33 (m, 2H), 7.33 – 7.26 (m, 2H), 7.24 – 7.17 (m, 1H), 3.81 (t, J = 7.0 Hz, 1H), 2.82 (s, 2H), 2.58 (hept, J = 6.3 Hz, 1H), 1.46 (s, 2H), 1.07 (d, J = 6.2 Hz, 3H), 0.97 (d, J = 6.3 Hz, 3H), 3.40 – 1.04 (m, 9H).

^{13}C{^{1}H} NMR (126 MHz, $CDCl_3$) δ 146.6, 128.3, 127.7, 126.8, 60.9, 54.4, 46.3, 25.8, 24.2, 21.9.

^{11}B NMR (128 MHz, $CDCl_3$) δ -0.6, -6.5 (d, J = 162.5 Hz), -10.0 (d, J = 149.9 Hz), -13.2 (d, J = 147.9 Hz), -14.0 (d, J = 166.1 Hz), -17.7 (d, J = 181.0 Hz), -20.1 (d, J = 191.5 Hz).

HRMS (ESI): *m/z* calculated for $[C_{13}H_{27}B_{10}NH]^+$ $[M+H]^+$: 306.3219; found: 306.3219.

Amine 13

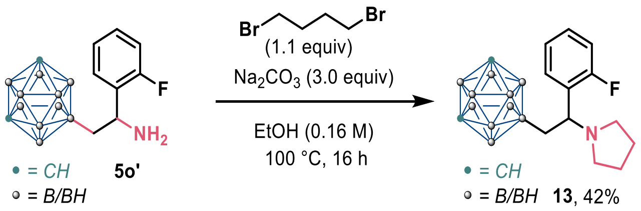

Free amine **5o'** was obtained from amine hydrochloride **5o** by adding aq. NaOH (1.0 M) and CH_2Cl_2 to **5o**, shaking well, separating the layers, extracting of the aqueous layer with CH_2Cl_2, drying the organic layers over Na_2SO_4, evaporating, and drying. **5o'** obtained this way was used without further purification for the next reaction step.

A Schlenk tube was charged with a magnetic stirring bar, **5o'** (22.0 mg, 78.2 µmol, 1.0 equiv), and Na_2CO_3 (24.9 mg, 0.235 mmol, 3.0 equiv). The tube was evacuated and backfilled with argon three times and was then charged with dry EtOH (0.50 mL, 0.16 M) and 1,4-dibromobutane (18.6 mg, 86.1 µmol, 1.1 equiv). The mixture was stirred at 100 °C for 16 h and was then filtered through Celite. The pad was washed with EtOH (2 mL), the obtained solution was concentrated, and the residue was purified by column chromatography on deactivated silica (*n*-pentane/ethyl acetate = 15:1 to 5:1) to give the title compound as a colorless oil (11.0 mg, 32.8 µmol, 42%).

^{1}H NMR (400 MHz, $CDCl_3$) δ 7.51 – 7.40 (m, 1H), 7.25 – 7.18 (m, 1H), 7.12 (t, *J* = 7.5 Hz, 1H), 7.03 – 6.96 (m, 1H), 4.02 (dd, *J* = 10.4, 5.3 Hz, 1H), 2.75 (s, 2H), 2.70 – 2.58 (m, 2H), 2.48 – 2.35 (m, 2H), 1.78 – 1.64 (m, 6H), 3.25 – 1.10 (m, 9H).

^{13}C{^{1}H} NMR (101 MHz, $CDCl_3$) δ 161.6 (d, *J* = 244.6 Hz), 130.2 (d, *J* = 4.6 Hz), 128.7, 127.4, 123.9, 115.1 (d, *J* = 23.8 Hz), 59.5, 54.3, 50.6, 23.1, 20.1.

^{11}B{^{1}H} NMR (128 MHz, $CDCl_3$) δ -0.9, -6.6, -10.0, -13.4, -14.1, -17.8, -20.0.

^{19}F{^{1}H} NMR (376 MHz, $CDCl_3$) δ -116.3.

HRMS (ESI): *m/z* calculated for $[C_{14}H_{26}NB_{10}FH]^+$ $[M+H]^+$: 336.3132; found: 336.3122.

Amine 5q'

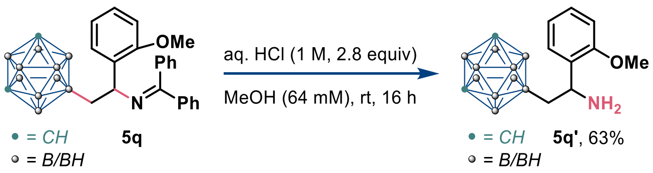

To a 20 mL vial was added a magnetic stirring bar, **5q** (58.0 mg, 0.127 mmol, 1.0 equiv), methanol (2.0 ml, 64 mM), and aq. HCl (1.0 M; 350 µL, 0.350 mmol, 2.8 equiv). The mixture was stirred at room temperature for 16 h, and then, the solvent was removed under reduced pressure. Aq. HCl (1.0 M; 2 mL) was added, and the aqueous layer was washed with *n*-pentane (3 x 5 mL). The aqueous layer was then basified using aq. sat. $NaHCO_3$ and was then extracted with CH_2Cl_2 (3 x 5 mL). The combined organic layers were dried over Na_2SO_4 and were then evaporated. Column chromatography on deactivated silica (CH_2Cl_2 + 1% MeOH to CH_2Cl_2 + 2% MeOH) gave the title compound as a colorless oil (23.4 mg, 79.8 µmol, 63%).

^{1}H NMR (400 MHz, $CDCl_3$) δ 7.41 – 7.32 (m, 1H), 7.23 – 7.16 (m, 1H), 6.91 (tt, *J* = 7.4, 1.5 Hz, 1H), 6.85 – 6.81 (m, 1H), 4.35 (t, *J* = 7.4 Hz, 1H), 3.85 – 3.81 (m, 3H), 3.39 (s, 2H), 2.83 (s, 2H), 1.63 (dd, *J* = 14.9, 6.8 Hz, 1H), 1.39 (dd, *J* = 14.8, 7.4 Hz, 1H), 3.82 – 1.13 (m, 9H).

^{13}C{^{1}H} NMR (101 MHz, $CDCl_3$) δ 157.1, 134.0, 127.9, 127.4, 120.6, 110.6, 55.4, 54.4, 51.0, 31.1,

24.0.

^{11}B{^{1}H} NMR (128 MHz, $CDCl_3$) δ -0.4, -6.4, -9.9, -13.2, -14.0, -17.7, -20.0.

HRMS (ESI): *m/z* calculated for $[C_{11}H_{23}NOB_{10}Na]^+$ $[M+Na]^+$: 316.2681; found: 316.2676.

Amine 14

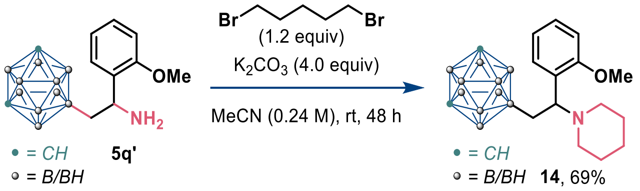

A Schlenk tube was charged with a magnetic stirring bar, **5q'** (21.0 mg, 71.6 µmol, 1.0 equiv), and K_2CO_3 (39.5 mg, 0.286 mmol, 4.0 equiv). The tube was evacuated and backfilled with argon three times and was then charged with dry MeCN (0.30 mL, 0.24 M). After stirring at rt for 10 mins, 1,5-dibromopentane (19.8 mg, 86.1 µmol, 1.2 equiv) was added and the mixture was stirred at rt for 48 h. The mixture was then filtered through Celite, the pad was washed with CH_2Cl_2 (5 mL), and the resulting solution was evaporated. Column chromatography on deactivated silica (CH_2Cl_2 + 0.5% MeOH to CH_2Cl_2 + 2% MeOH) gave the title compound as a light-yellow solid (17.8 mg, 49.2 µmol, 69%).

^{1}H NMR (500 MHz, $CDCl_3$) δ 7.30 – 7.22 (m, 2H), 6.94 (t, *J* = 7.4 Hz, 1H), 6.90 – 6.85 (m, 1H), 4.50 (s, 1H), 3.80 (s, 3H), 2.73 (s, 2H), 2.58 – 2.36 (m, 4H), 1.84 – 1.52 (m, 6H), 1.41 – 1.26 (m, 2H), 3.09 – 1.11 (m, 9H).

^{13}C{^{1}H} NMR (126 MHz, $CDCl_3$) δ 158.7, 129.8, 128.4, 119.9, 110.8, 60.8, 55.6, 54.2, 49.9, 25.7, 24.3, 17.9.

^{11}B{^{1}H} NMR (160 MHz, $CDCl_3$) δ -0.6, -6.6, -10.1, -13.4, -14.3, -17.9, -20.3.

HRMS (ESI): *m/z* calculated for $[C_{16}H_{32}B_{10}NO]^+$ $[M+H]^+$: 362.3490; found: 362.3457.

6.2 Further hydrolytic downstream transformations

Amine hydrochloride 15

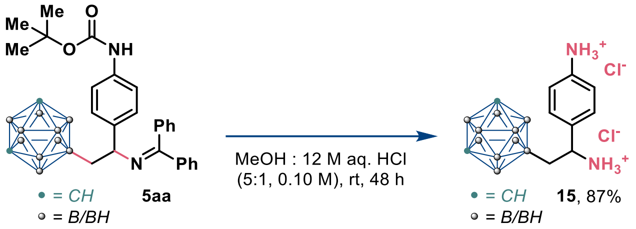

To a 4 mL vial was added a magnetic stirring bar, compound **5aa** (32.4 mg, 59.7 µmol, 1.0 equiv), methanol (0.50 mL), and aq. HCl (12 M; 0.10 mL). The mixture was stirred at room temperature for 48 h, and then, aq. HCl (1.0 M; 1 mL) was added, and the aqueous layer was washed with *n*-pentane (3 x 3 mL). The aqueous layer was subsequently filtered through Celite, the Celite pad was washed with water (1.0 mL), and the volatiles were evaporated. Drying under vacuum delivered the desired product as an orange solid (18.2 mg, 51.8 µmol, 87%).

^{1}H NMR (400 MHz, D_2O) δ 7.72 – 7.63 (m, 2H), 7.51 – 7.40 (m, 2H), 4.49 (dd, J = 12.7, 3.0 Hz, 1H), 3.24 (s, 2H), 1.89 (t, J = 13.6 Hz, 1H), 1.67 (dd, J = 14.4, 2.4 Hz, 1H), 3.31 – 1.00 (m, 9H).

^{13}C{^{1}H} NMR (101 MHz, D_2O) δ 137.5, 131.0, 130.4, 123.5, 55.5, 55.3, 55.2, 19.6.

^{11}B{^{1}H} NMR (128 MHz, D_2O) δ -3.1, -7.0, -10.7, -13.4, -14.1, -17.4, -19.4.

HRMS (ESI): *m/z* calculated for $[C_{20}H_{44}B_{20}N_4H]^+$ $[2xM\text{-}4Cl^-\text{-}3H^+]^+$: 557.5657; found: 557.5651.

Amine hydrochloride 16

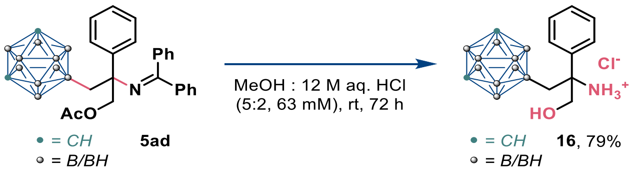

To a 4 mL vial was added a magnetic stirring bar, compound **5ad** (22.0 mg, 44.0 µmol, 1.0 equiv), methanol (0.50 mL), and aq. HCl (12 M; 0.20 mL). The mixture was stirred at room temperature for 72 h, and then, aq. HCl (1.0 M; 1 mL) was added, and the aqueous layer was washed with *n*-pentane (3 x 3 mL). The aqueous layer was subsequently filtered through Celite, the Celite pad was washed with water (1.0 mL), and the volatiles were evaporated. Drying under vacuum delivered the desired product as a white solid (11.5 mg, 34.9 µmol, 79%).

^{1}H NMR (400 MHz, D_2O) δ 7.51 – 7.36 (m, 5H), 4.09 (d, J = 12.1 Hz, 1H), 3.98 (d, J = 12.0 Hz, 1H), 3.22 (s, 2H), 1.95 (d, J = 15.5 Hz, 1H), 1.83 (d, J = 15.5 Hz, 1H), 3.10 – 0.98 (m, 9H).

$^{13}C\{^1H\}$ **NMR** (101 MHz, D_2O) δ 136.7, 128.7, 128.6, 126.2, 66.5, 64.0, 55.4, 22.4.

$^{11}B\{^1H\}$ **NMR** (128 MHz, D_2O) δ -3.9, -7.0, -10.6, -13.3, -14.1, -17.5, -19.1.

HRMS (ESI): *m/z* calculated for $[C_{11}H_{24}NOB_{10}]^+$ $[M-Cl^-]^+$: 294.2861; found: 294.2854.

Amine hydrochloride 17

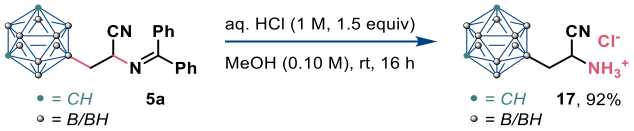

To a 20 mL vial was added a magnetic stirring bar, compound **5a** (18.8 mg, 49.9 µmol, 1.0 equiv), methanol (0.50 mL, 0.10 M), and aq. HCl (1.0 M; 75 µL, 75 µmol, 1.5 equiv). The mixture was stirred at room temperature for 16 h, and then, the solvent was removed under reduced pressure. Aq. HCl (1.0 M; 1 mL) was added, and the aqueous layer was washed with *n*-pentane (3 x 5 mL). The aqueous layer was subsequently evaporated and dried under vacuum to obtain the desired product as a white solid (11.4 mg, 45.8 µmol, 92%).

^1H **NMR** (400 MHz, D_2O) δ 4.46 (dd, *J* = 12.8, 3.8 Hz, 1H), 3.48 (s, 2H), 1.73 (t, *J* = 13.6 Hz, 1H), 1.61 (dd, *J* = 14.4, 3.7 Hz, 1H), 3.52 – 1.41 (m, 9H).

$^{13}C\{^1H\}$ **NMR** (101 MHz, D_2O) δ 117.1, 55.8, 42.3, 17.4.

$^{11}B\{^1H\}$ **NMR** (128 MHz, D_2O) δ -4.1, -6.9, -10.5, -13.1, -13.6, -17.1, -18.9.

HRMS (ESI): *m/z* calculated for $[C_5H_{16}B_{10}N_2Na]^+$ $[M-HCl+Na^+]^+$: 235.2211; found: 235.2209.

Amine 18

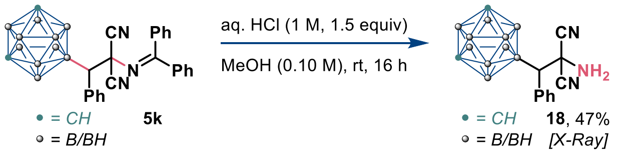

To a 20 mL vial was added a magnetic stirring bar, compound **5k** (28.8 mg, 60.3 µmol, 1.0 equiv), methanol (0.60 mL, 0.10 M), and aq. HCl (1.0 M; 90 µL, 90 µmol, 1.5 equiv). The mixture was stirred at room temperature for 16 h, and then, the solvent was removed under reduced pressure. Sat. aq. $NaHCO_3$ (1 mL) was added, and the aqueous layer was extracted with CH_2Cl_2 (3 x 5 mL). The organic layer was subsequently dried over Na_2SO_4 and evaporated. Column chromatography on deactivated silica (*n*-pentane/ethyl acetate = 10:1 to 5:1) gave the title compound as a white solid (8.9 mg, 28 µmol, 47%).

^{1}**H NMR** (400 MHz, CD_2Cl_2) δ 7.78 – 7.71 (m, 1H), 7.45 – 7.38 (m, 1H), 7.37 – 7.27 (m, 2H), 7.18 – 7.08 (m, 1H), 3.06 (s, 3H), 2.29 (s, 2H), 3.46 – 1.12 (m, 9H).

^{13}**C{^{1}H} NMR** (101 MHz, CD_2Cl_2) δ 139.4, 131.7, 129.5, 129.3, 128.0, 127.9, 117.4, 117.1, 55.4, 51.6, 44.9.

^{11}**B{^{1}H} NMR** (128 MHz, CD_2Cl_2) δ -2.0, -6.3, -9.8, -12.9, -13.5, -17.2, -18.2.

HRMS (ESI): *m/z* calculated for $[C_{12}H_{19}N_3B_{10}Na]^+$ $[M+Na]^+$: 336.2480; found: 336.2471.

Note: This compound's structure was further confirmed by X-Ray crystal structure analysis.

6.3 Clickable carborane-containing building blocks via diazotransfer

The procedures were executed similar to a reported procedure.[35] FSO_2N_3 was prepared according to this reference.

Azide 19

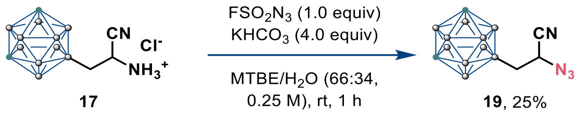

To a Schlenk tube under air was added a magnetic stirring bar and amine hydrochloride **17** (16.3 mg, 65.5 µmol, 1.0 equiv). A solution of FSO_2N_3 in MTBE (0.39 M; 170 µL, 66 µmol, 1.0 equiv) and an aqueous solution of $KHCO_3$ (3.0 M; 88 µL, 0.26 mmol, 4.0 equiv) were added. The reaction mixture was stirred for 1 h at room temperature. Then, EtOAc (30 mL) was added and the organic layer was washed sequentially with brine (3 x 30 mL), water (2 x 30 mL), and brine (30 mL), dried over Na_2SO_4, concentrated, and dried in vacuo to afford the title compound as a colorless oil (3.9 mg, 0.016 mmol, 25%) without further purification.

^{1}**H NMR** (400 MHz, $CDCl_3$) δ 4.05 (t, J = 7.8 Hz, 1H), 2.98 (s, 2H), 1.66 – 1.48 (m, 2H), 3.57 – 0.93 (m, 9H).

^{13}**C{^{1}H} NMR** (101 MHz, $CDCl_3$) δ 117.7, 55.0, 51.7, 19.4.

^{11}**B{^{1}H} NMR** (128 MHz, $CDCl_3$) δ -3.0, -6.4, -9.9, -13.1, -13.6, -17.3, -19.1.

HRMS (ESI): *m/z* calculated for $[C_5H_{14}N_4B_{10}Na]^+$ $[M+Na]^+$: 261.2117; found: 261.2113.

Azide 20

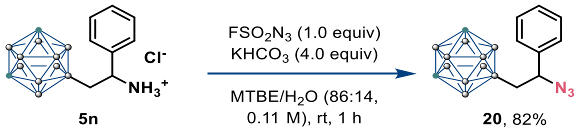

To a Schlenk tube under air was added a magnetic stirring bar and amine hydrochloride **5n** (30.1 mg, 100 µmol, 1.0 equiv). A solution of FSO_2N_3 in MTBE (0.125 M; 800 µL, 100 µmol, 1.0 equiv) and an aqueous solution of $KHCO_3$ (3.0 M; 134 µL, 0.402 mmol, 4.0 equiv) were added. The reaction mixture was stirred for 1 h at room temperature. Then, EtOAc (30 mL) was added and the organic layer was washed sequentially with brine (3 x 30 mL), water (2 x 30 mL), and brine (30 mL), dried over Na_2SO_4, concentrated, and dried in vacuo to afford the title compound as a colorless oil (23.7 mg, 81.9 µmol, 82%) without further purification.

^{1}H NMR (400 MHz, $CDCl_3$) δ 7.40 – 7.28 (m, 5H), 4.40 (t, J = 7.5 Hz, 1H), 2.87 (s, 2H), 1.68 – 1.43 (m, 2H), 3.43 – 1.03 (m, 9H).

^{13}C{^{1}H} NMR (101 MHz, $CDCl_3$) δ 141.4, 128.7, 128.2, 127.3, 66.9, 54.6, 22.8.

^{11}B{^{1}H} NMR (128 MHz, $CDCl_3$) δ -1.2, -6.4, -9.9, -13.2, -13.9, -17.5, -19.7.

HRMS (ESI): m/z calculated for $[B_{20}N_6H_{38}C_{20}Ag]^+$ $[2M+Ag]^+$: 686.4215; found: 686.4217.

7. X-Ray analysis

X-Ray diffraction: Data sets for compounds **3a**, **5a**, **5o**, and **18** were collected with a Bruker D8 Venture Photon III Diffractometer. Programs used: data collection: *APEX4* Version 2021.4-0[36] (Bruker AXS Inc., **2021**); cell refinement: *SAINT* Version 8.40B (Bruker AXS Inc., **2021**); data reduction: *SAINT* Version 8.40B (Bruker AXS Inc., **2021**). A Multi-Scan absorption correction using SADABS Version 2016/2 (Bruker AXS Inc., **2021**) was applied. The structure was solved by Intrinsic Phasing methods with *SHELXT*-Version 2018-3[37] (Sheldrick, G. M. *Acta Cryst.,* **2015**, *A71*, 3-8) and refined by full-matrix least-squares methods against F^2 using *SHELXL*- Version 2018-3[38] (Sheldrick, G. M. *Acta Cryst.,* **2015**, *C71* (1), 3-8). All non-hydrogen atoms were refined with anisotropic displacement parameters. All hydrogen atoms at carbon were refined isotropic on calculated positions using a riding model with their U_{iso} values constrained to 1.5 times the U_{eq} of their pivot atoms for terminal sp^3 carbon atoms and 1.2 times for all other carbon atoms. *R*-values are given for observed reflections, and wR^2 values are given for all reflections.

Exceptions and special features: For compounds **3a**, **5a** and **18** the hydrogen atoms of the *closo*-Carborane groups (carbon and boron atoms) were found in the difference map and refined freely. For compound **5o** three independent molecules were found in the asymmetric unit. For molecules named with suffix "A" and "B" the hydrogen atoms of the *closo*-Carborane groups (carbon and boron atoms) were found in the difference map and refined freely. For the independent molecule with suffix "C" the hydrogen atoms of the *closo*-Carborane group were found in the difference map and refined freely, but with restraints on the B-H and C-H distances (U_{iso} values constrained to 1.5 times the U_{eq} of their pivot atom). Moreover, for compound **5o** a half badly disordered water molecule was found in the asymmetric unit and could not be satisfactorily refined. The program SQUEEZE (Spek, A.L. (**2015**). Acta Cryst. C71, 9-18) was therefore used to remove mathematically the effect of the solvent. The quoted formula and derived parameters include the squeezed solvent molecule. For compound **18** the hydrogen atoms at nitrogen (NH_2 groups) were found in the difference map and refined freely.

X-ray crystal structure analysis of 3a (glo10626): A colorless, plate-like specimen of $C_{16}H_{21}B_{10}NO_2$, approximate dimensions 0.031 mm x 0.102 mm x 0.142 mm, was used for the X-ray crystallographic analysis. The X-ray intensity data were measured on a single crystal diffractometer Bruker D8 Venture Photon III system equipped with a micro focus tube Cu ImS (CuKα, λ = 1.54178 Å) and a MX mirror monochromator. A total of 1817 frames were collected. The total exposure time was 14.57 hours. The frames were integrated with the Bruker SAINT software package using a wide-frame algorithm. The integration of the data using a monoclinic unit cell yielded a total of 22472 reflections to a maximum θ angle of 66.65° (0.84 Å resolution), of which 3481 were independent (average redundancy 6.456, completeness = 99.8%, R_{int} = 6.92%, R_{sig} = 3.77%) and 2790 (80.15%) were greater than 2σ(F^2). The final cell constants

of a = 12.4058(2) Å, b = 12.2488(3) Å, c = 13.3959(3) Å, β = 104.3400(10)°, volume = 1972.17(7) Å^3, are based upon the refinement of the XYZ-centroids of 5922 reflections above 20 σ(I) with 8.701° < 2θ < 133.1°. Data were corrected for absorption effects using the multi-scan method (SADABS). The ratio of minimum to maximum apparent transmission was 0.880. The calculated minimum and maximum transmission coefficients (based on crystal size) are 0.9280 and 0.9840. The structure was solved and refined using the Bruker SHELXTL Software Package, using the space group $P2_1/n$, with Z = 4 for the formula unit, $C_{16}H_{21}B_{10}NO_2$. The final anisotropic full-matrix least-squares refinement on F^2 with 306 variables converged at R1 = 3.84%, for the observed data and wR2 = 9.68% for all data. The goodness-of-fit was 1.026. The largest peak in the final difference electron density synthesis was 0.201 e$^-$/Å^3 and the largest hole was -0.221 e$^-$/Å^3 with an RMS deviation of 0.041 e$^-$/Å^3. On the basis of the final model, the calculated density was 1.238 g/cm^3 and F(000), 760 e$^-$. CCDC Nr.: 2423424.

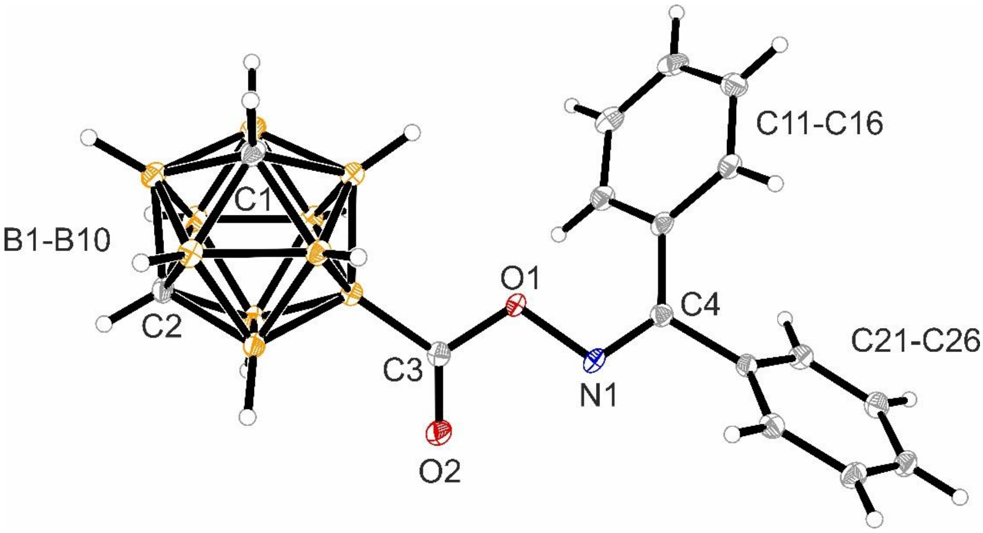

Supplementary Figure 24. Crystal structure of compound **3a**. Thermal ellipsoids are shown at 50% probability.

X-ray crystal structure analysis of 5a (glo10676): A colorless, prism-like specimen of $C_{18}H_{24}B_{10}N_2$, approximate dimensions 0.078 mm x 0.104 mm x 0.107 mm, was used for the X-ray crystallographic analysis. The X-ray intensity data were measured on a single crystal diffractometer Bruker D8 Venture Photon III system equipped with a micro focus tube Cu ImS (CuKα, λ = 1.54178 Å) and a MX mirror monochromator. A total of 763 frames were collected. The total exposure time was 4.61 hours. The frames were integrated with the Bruker SAINT software package using a wide-frame algorithm. The integration of the data using an orthorhombic unit cell yielded a total of 22969 reflections to a maximum θ angle of 68.35° (0.83 Å resolution), of which 3840 were independent (average redundancy 5.982, completeness = 99.3%, R_{int} = 8.39%, R_{sig} = 5.05%) and 3446 (89.74%) were greater than 2σ(F^2). The final cell constants

of a = 10.5732(3) Å, b = 13.9729(4) Å, c = 14.2704(4) Å, volume = 2108.28(10) Å^3, are based upon the refinement of the XYZ-centroids of 5088 reflections above 20 σ(I) with 8.857° < 2θ < 136.6°. Data were corrected for absorption effects using the multi-scan method (SADABS). The ratio of minimum to maximum apparent transmission was 0.791. The calculated minimum and maximum transmission coefficients (based on crystal size) are 0.9530 and 0.9650. The structure was solved and refined using the Bruker SHELXTL Software Package, using the space group $P2_12_12_1$, with Z = 4 for the formula unit, $C_{18}H_{24}B_{10}N_2$. The final anisotropic full-matrix least-squares refinement on F^2 with 315 variables converged at R1 = 4.03%, for the observed data and wR2 = 9.91% for all data. The goodness-of-fit was 1.097. The largest peak in the final difference electron density synthesis was 0.174 e$^-$/Å^3 and the largest hole was -0.208 e$^-$/Å^3 with an RMS deviation of 0.046 e$^-$/Å^3. On the basis of the final model, the calculated density was 1.186 g/cm^3 and F(000), 784 e$^-$. CCDC Nr.: 2423425.

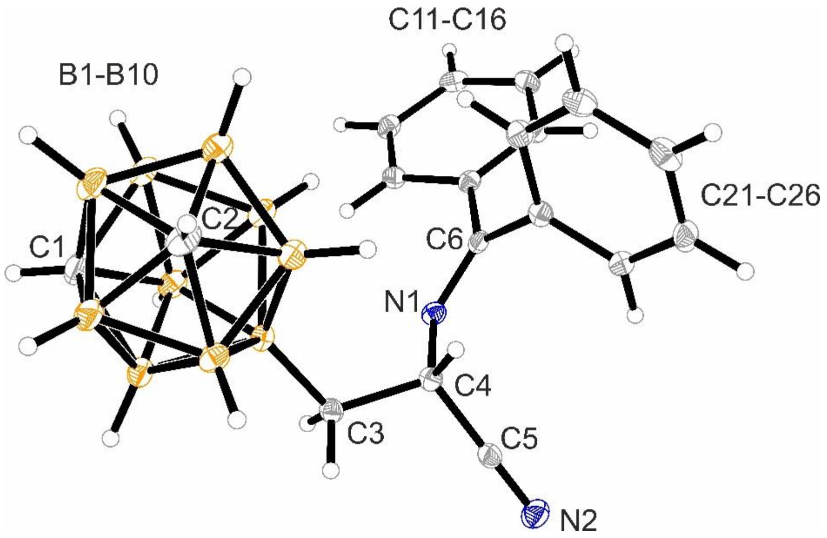

Supplementary Figure 25. Crystal structure of compound **5a**. Thermal ellipsoids are shown at 50% probability.

X-ray crystal structure analysis of 5o (glo10781): A colourless, prism shaped specimen of $C_{10}H_{21}B_{10}ClFN$ x 1/2 H_2O, approximate dimensions 0.086×0.151×0.188 mm^3, was used for the X-ray crystallographic analysis. The crystals were crystallised from deuterated water. The X-ray intensity data of glo10781 were measured on a Bruker D8 VENTURE KAPPA diffractometer system equipped with a microfocus sealed tube (λ = 0.71073 Å) and a multilayer mirror monochromator. A total of 2088 frames were collected. The total exposure time was 15.94 hours. The frames were integrated with the SAINT V8.41 package using a narrow-frame algorithm. The integration of the data using a triclinic unit cell yielded a total of 103002 reflections to a maximum θ angle of 26.78° (0.79 Å resolution), of which 11436 were independent (average redundancy 9.01, completeness = 99.9%, Rint = 5.85%, Rsig = 3.04%) and 9850 (86.1%) were greater than

$2\sigma(F^2)$. The final cell constants of a = 13.5868(4) Å, b = 15.1370(4) Å, c = 15.3091(5) Å, volume = 2684.71(14) Å^3, are based upon the refinement of the XYZ-centroids of 9699 reflections above 20 $\sigma(I)$ with 2.22° < 2θ < 26.74°. Data were corrected for absorption effects using the Multi-Scan method in SADABS 2016/2. The calculated minimum and maximum transmission coefficients (based on crystal size) are 0.961 and 0.982. The structure was solved by SHELXT 2018/2 and refined using the SHELXL-2019/2 Software, in the space group *P*-1 (2), with Z = 6 for the formula unit $C_{10}H_{21}B_{10}ClFN$ x 1/2 H_2O. The final anisotropic full-matrix least-squares refinement on F^2 with 779 variables against 11436 data points 396 and 396 restraints converged at R_1 = 5.34%, for the observed data and wR_2 = 14.96% for all data. The goodness-of-fit on F^2 was 1.06. The largest peak in the final difference electron density synthesis was 0.97 e$^-$/Å^3 and the deepest hole was −0.48 e$^-$/Å^3 with an RMS deviation of 0.062 e$^-$/Å^3. On the basis of the final model, the calculated density was 1.21 g/cm^3 and *F*(000), 1014 e$^-$. CCDC Nr.: 2423426.

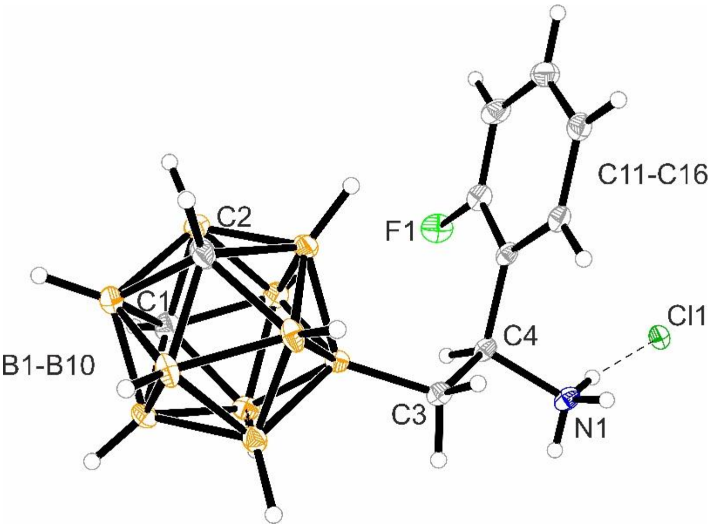

Supplementary Figure 26. Crystal structure of compound **5o**. Thermal ellipsoids are shown at 30% probability.

X-ray crystal structure analysis of 18 (glo10778): A colourless, prism shaped specimen of $C_{12}H_{19}B_{10}N_3$, approximate dimensions 0.047×0.137×0.144 mm^3, was used for the X-ray crystallographic analysis. The crystals were crystallised from DCM and heptane. The X-ray intensity data of glo10778 were measured on a Bruker D8 VENTURE KAPPA diffractometer system equipped with a microfocus sealed tube (λ = 1.54178 Å) and a multilayer mirror monochromator. The specimen was held at 100(2) K during the measurement with an Oxford Cryostream 700 low temperature device. A total of 2738 frames were collected. The total exposure time was 21.59 hours. The frames were integrated with the SAINT V8.41 package using a narrow-frame algorithm. The integration of the data using a triclinic unit cell yielded a total of 24218 reflections to a maximum θ angle of 68.19° (0.83 Å resolution), of which 3110 were independent

(average redundancy 7.79, completeness = 99.9%, Rint = 4.87%, Rsig = 3.33%) and 2797 (89.9%) were greater than 2σ(F^2). The final cell constants of a = 6.5139(2) Å, b = 9.6676(3) Å, c = 14.5377(4) Å, volume = 851.68(4) Å^3, are based upon the refinement of the XYZ-centroids of 9957 reflections above 20 σ(I) with 3.17° < 2θ < 68.15°. Data were corrected for absorption effects using the Multi-Scan method in SADABS 2016/2. The calculated minimum and maximum transmission coefficients (based on crystal size) are 0.935 and 0.978. The structure was solved by SHELXT 2018/2 and refined using the SHELXL-2019/2 Software, in the space group P-1 (2), with Z = 2 for the formula unit $C_{12}H_{19}B_{10}N_3$. The final anisotropic full-matrix least-squares refinement on F^2 with 278 variables against 3110 data points converged at R_1 = 3.63%, for the observed data and wR_2 = 9.89% for all data. The goodness-of-fit on F^2 was 1.03. The largest peak in the final difference electron density synthesis was 0.23 e⁻/Å^3 and the deepest hole was −0.22 e⁻/Å^3 with an RMS deviation of 0.038 e⁻/Å^3. On the basis of the final model, the calculated density was 1.22 g/cm^3 and F(000), 324 e⁻. CCDC Nr.: 2423427.

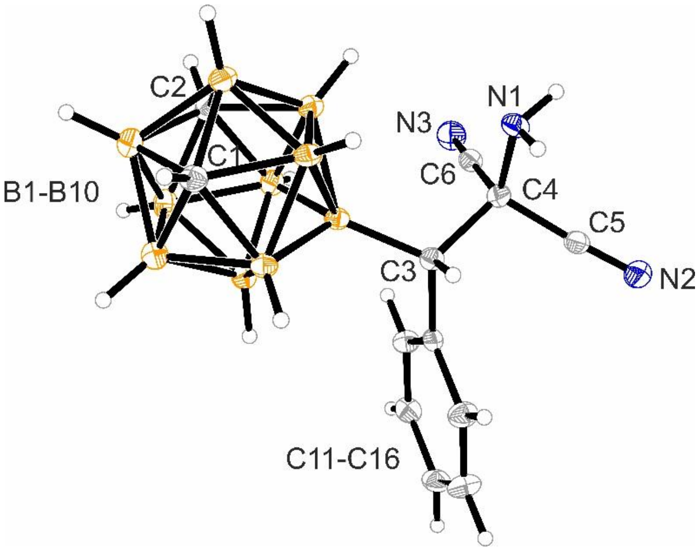

Supplementary Figure 27. Crystal structure of compound **18**. Thermal ellipsoids are shown at 50% probability.

8. NMR spectra

8.1 Starting materials

^{1}H NMR (acetone-d$_6$, 400 MHz) for **1a**

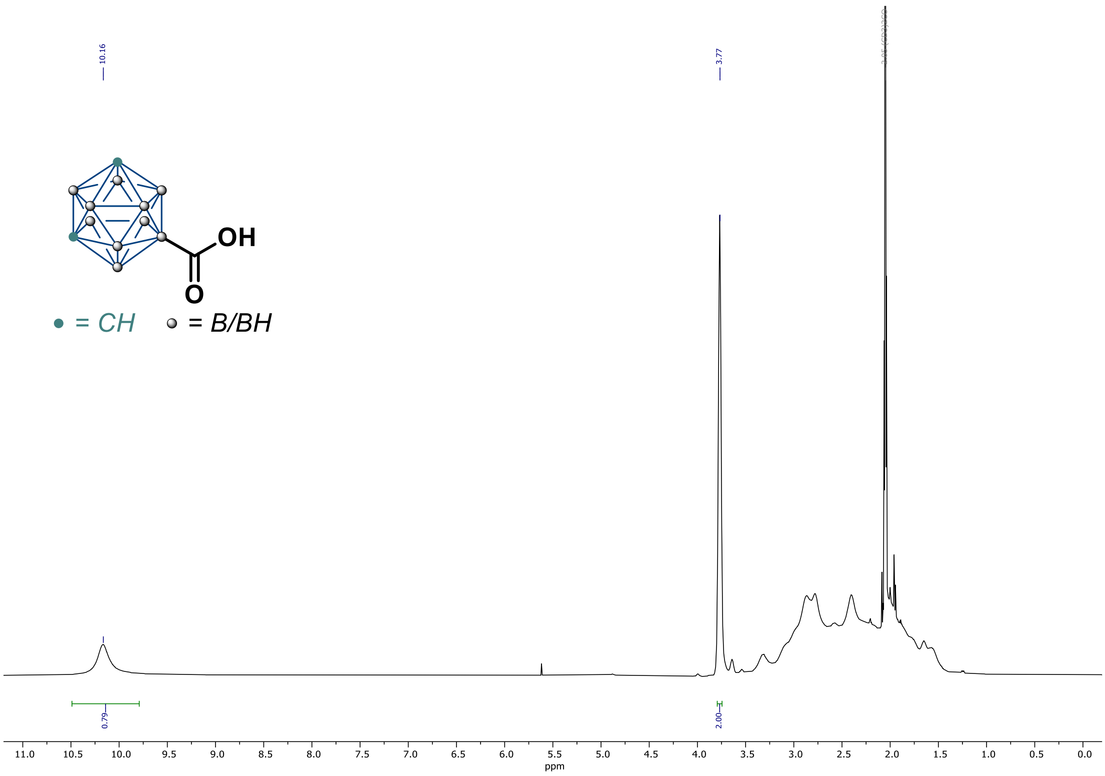

^{13}C{^{1}H} NMR (acetone-d$_6$, 101 MHz) for **1a**

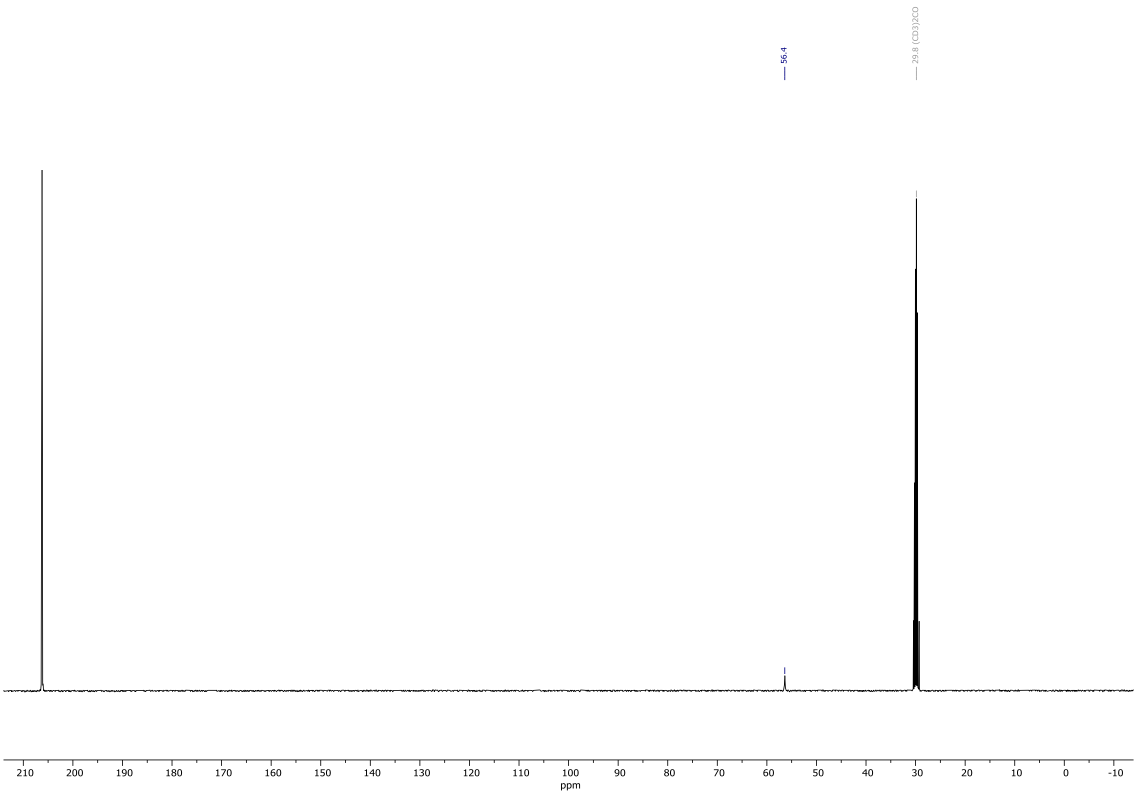

$^{11}B\{^1H\}$ NMR (acetone-d$_6$, 128 MHz) for **1a**

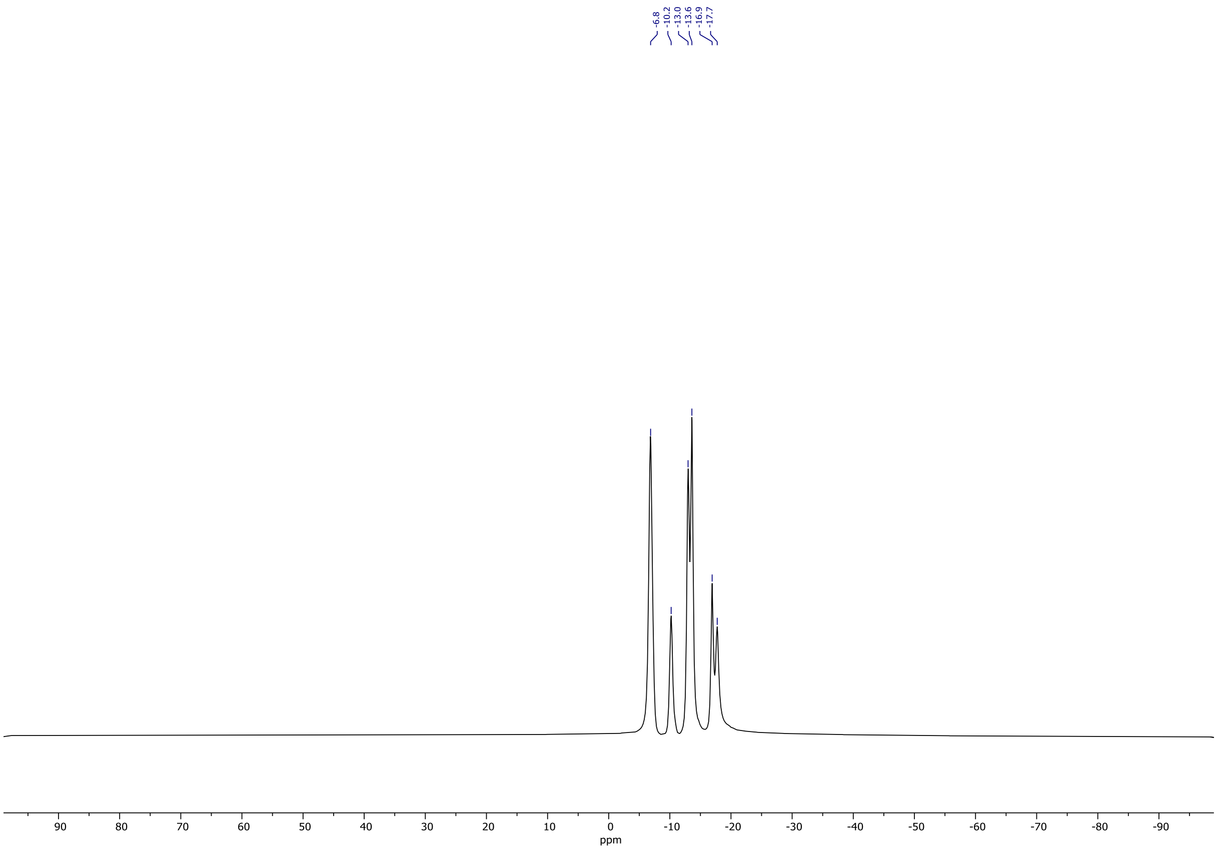

^{1}H NMR (CDCl$_3$, 400 MHz) for **1b**

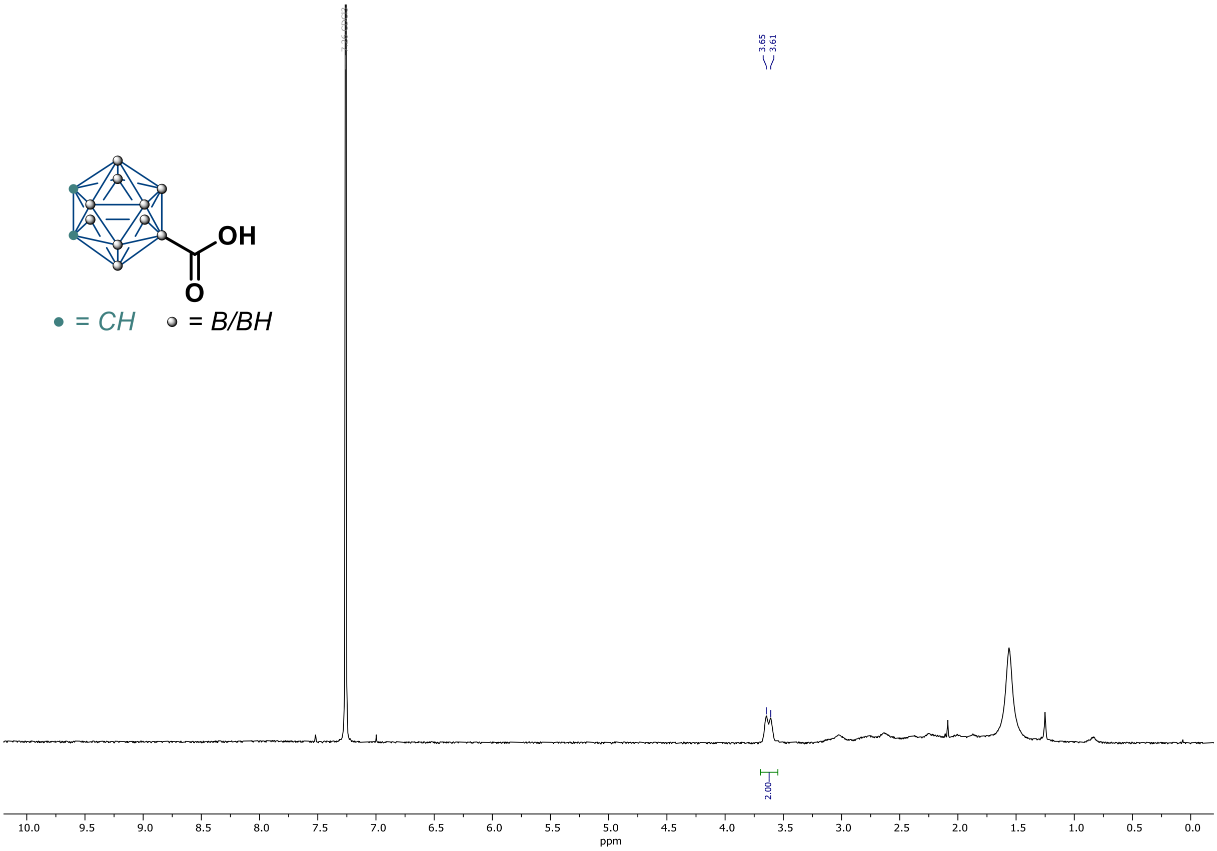

^{13}C{^{1}H} NMR (CDCl$_3$, 101 MHz) for **1b**

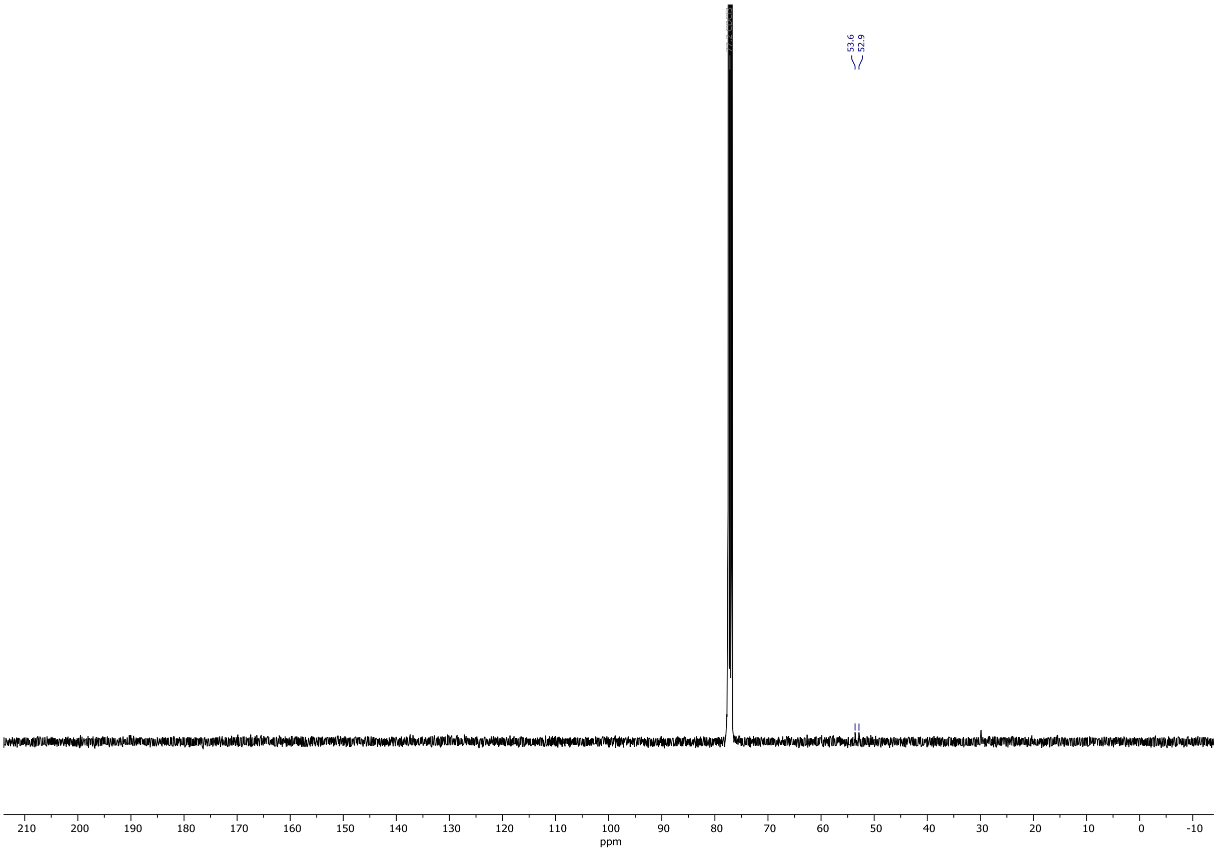

^{11}B{^{1}H} NMR (CDCl$_3$, 128 MHz) for **1b**

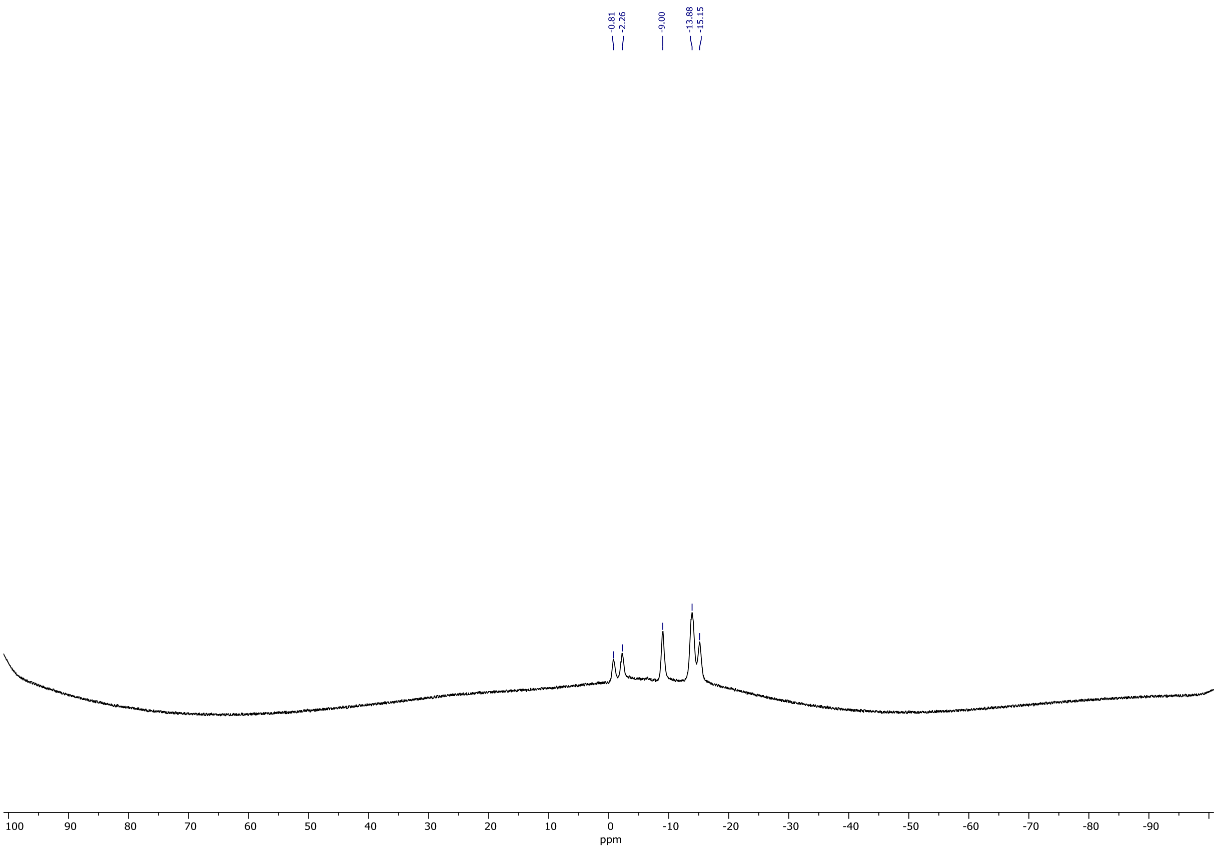

^{1}H NMR ($CDCl_3$, 400 MHz) for **1c**

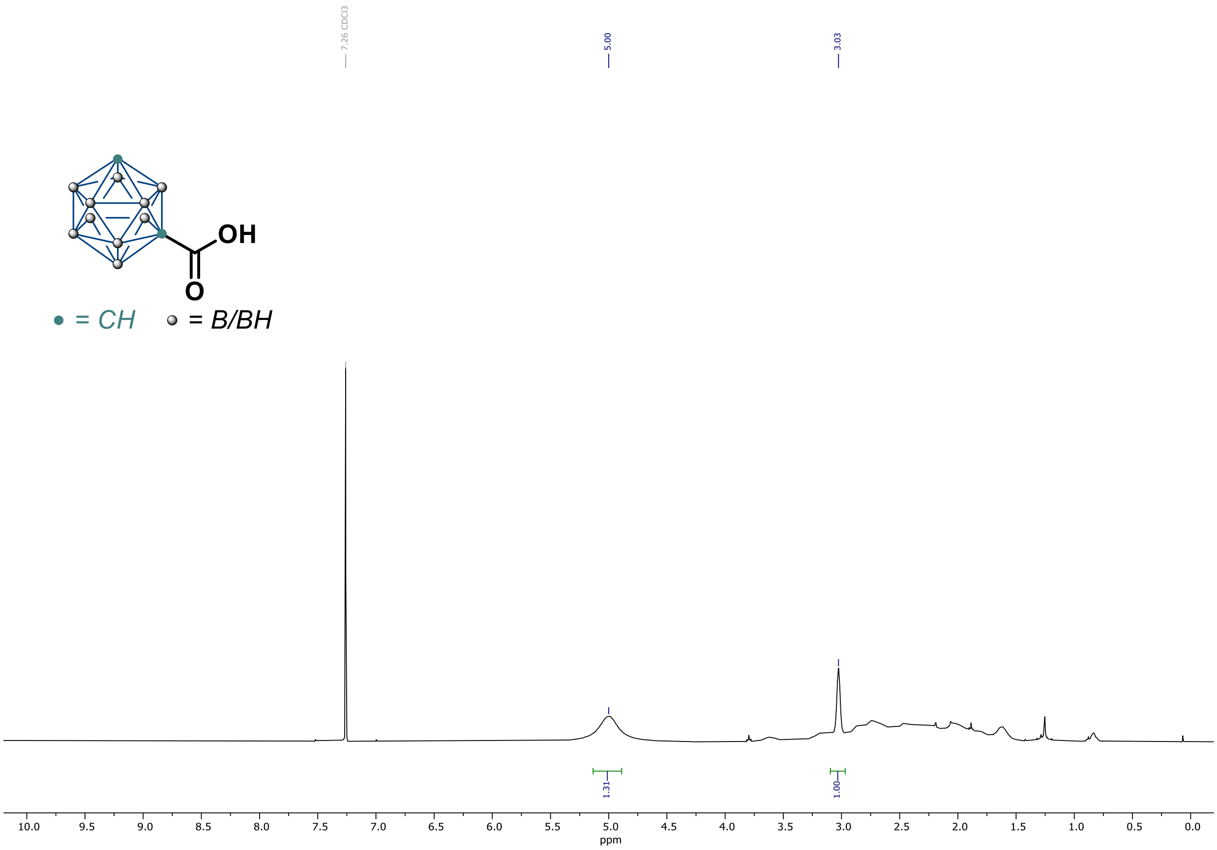

^{13}C{^{1}H} NMR ($CDCl_3$, 101 MHz) for **1c**

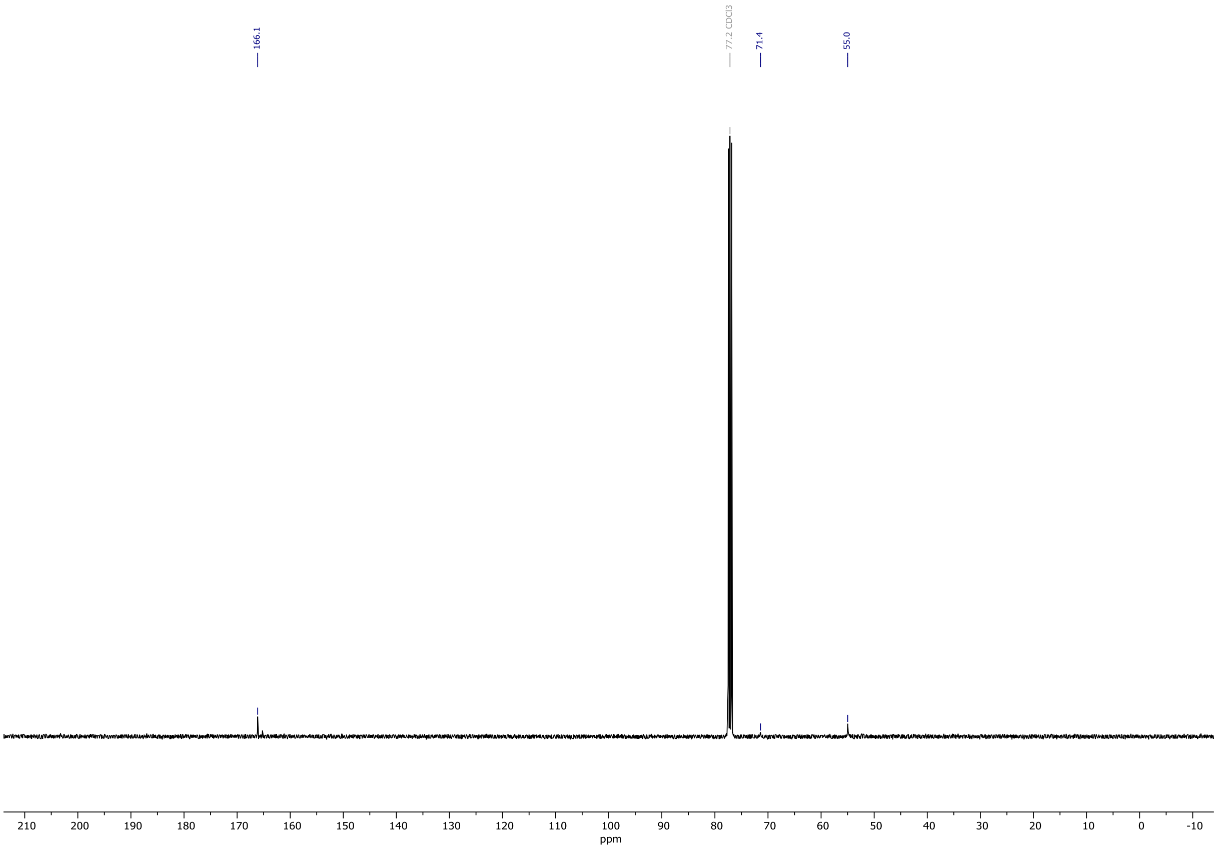

^{11}B{^{1}H} NMR (CDCl$_3$, 128 MHz) for **1c**

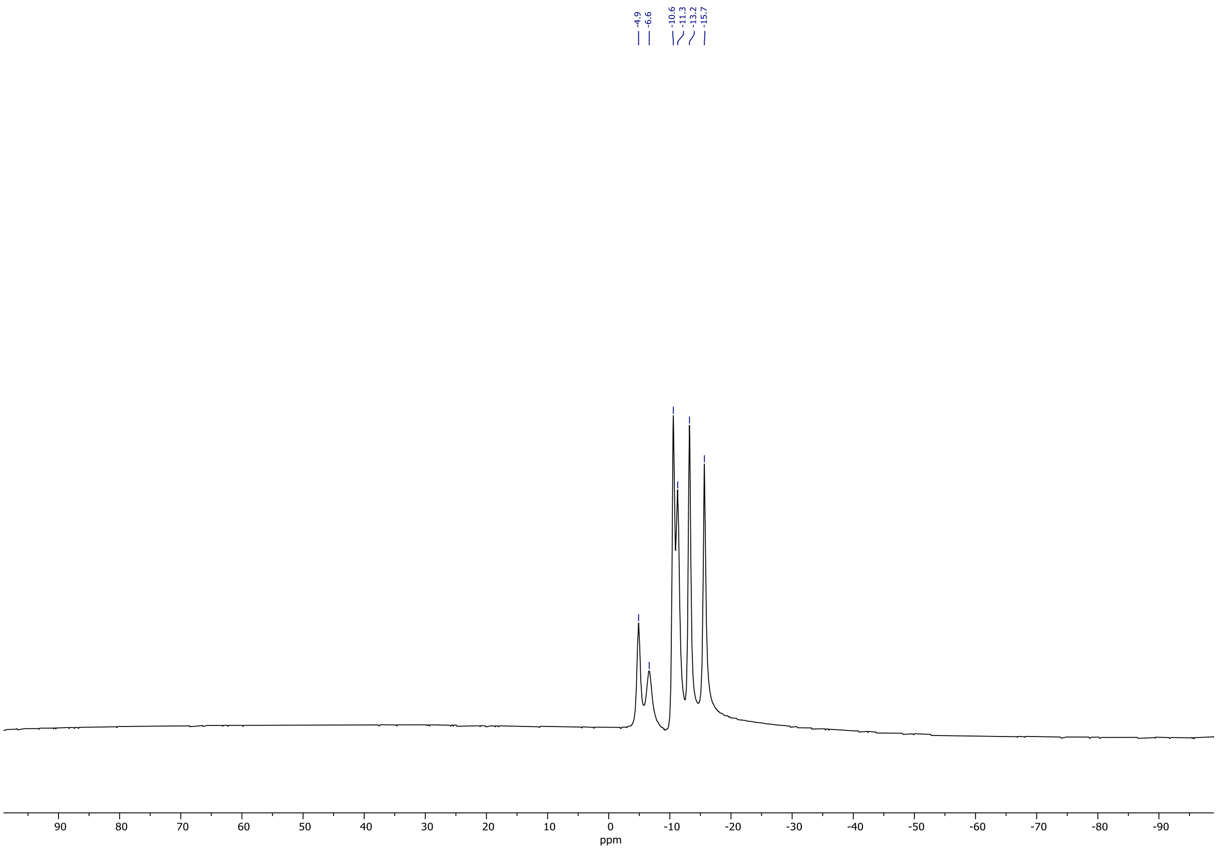

^{1}H NMR ($CDCl_3$, 400 MHz) for **1d**

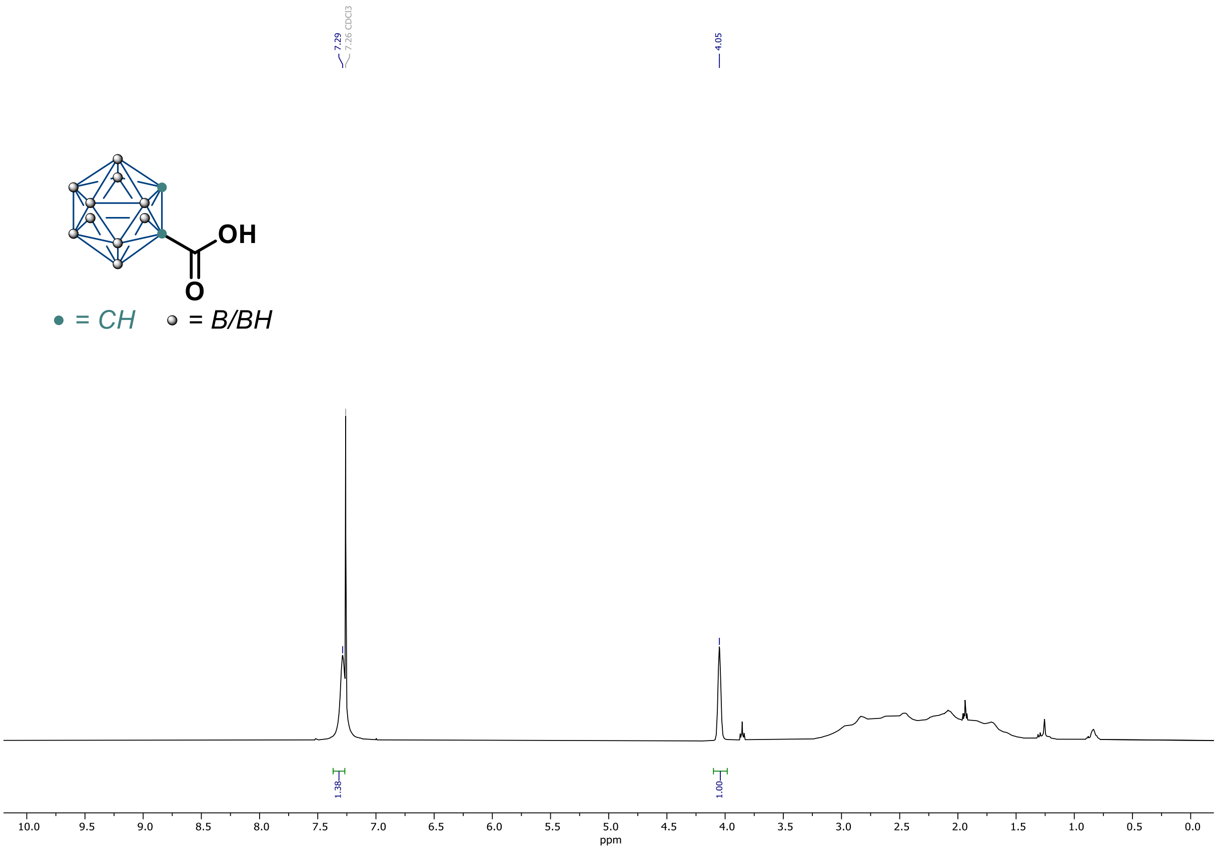

^{13}C{^{1}H} NMR ($CDCl_3$, 101 MHz) for **1d**

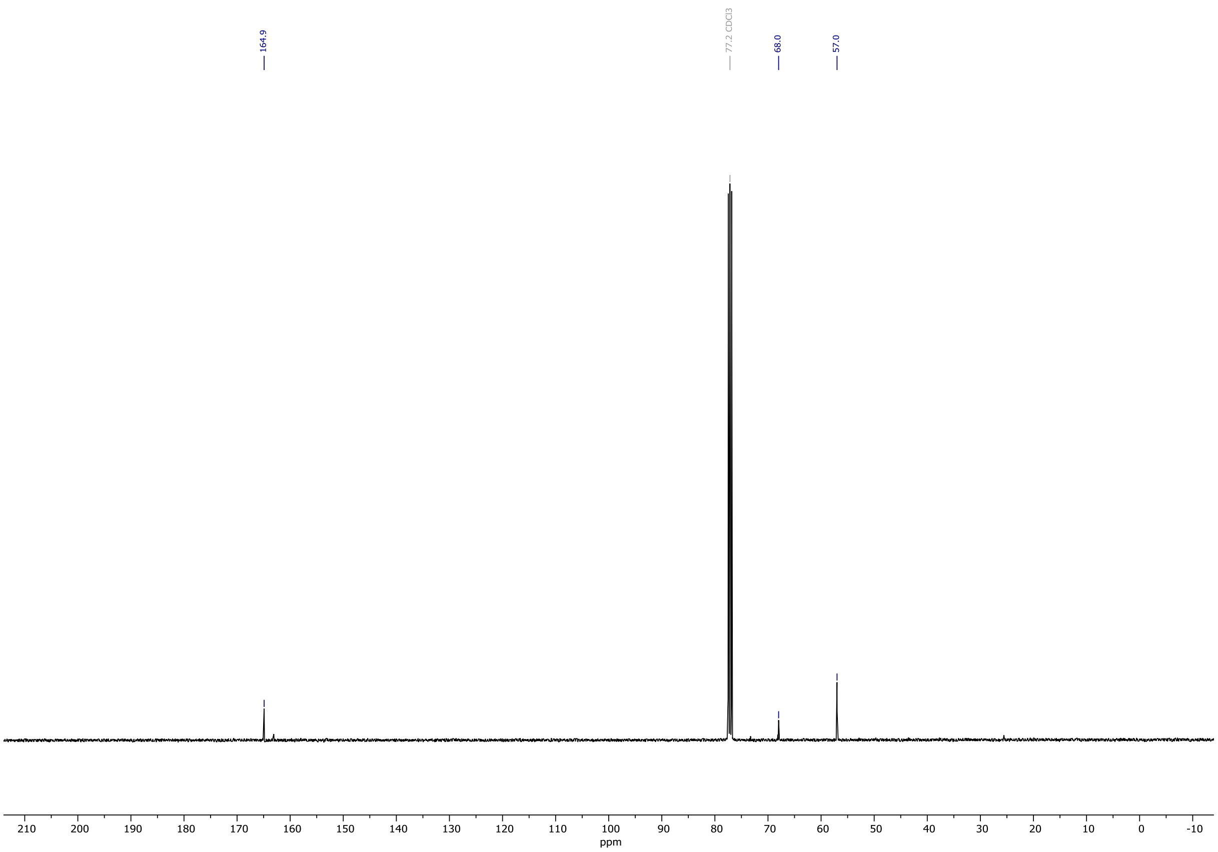

^{11}B{^{1}H} NMR (CDCl$_3$, 128 MHz) for **1d**

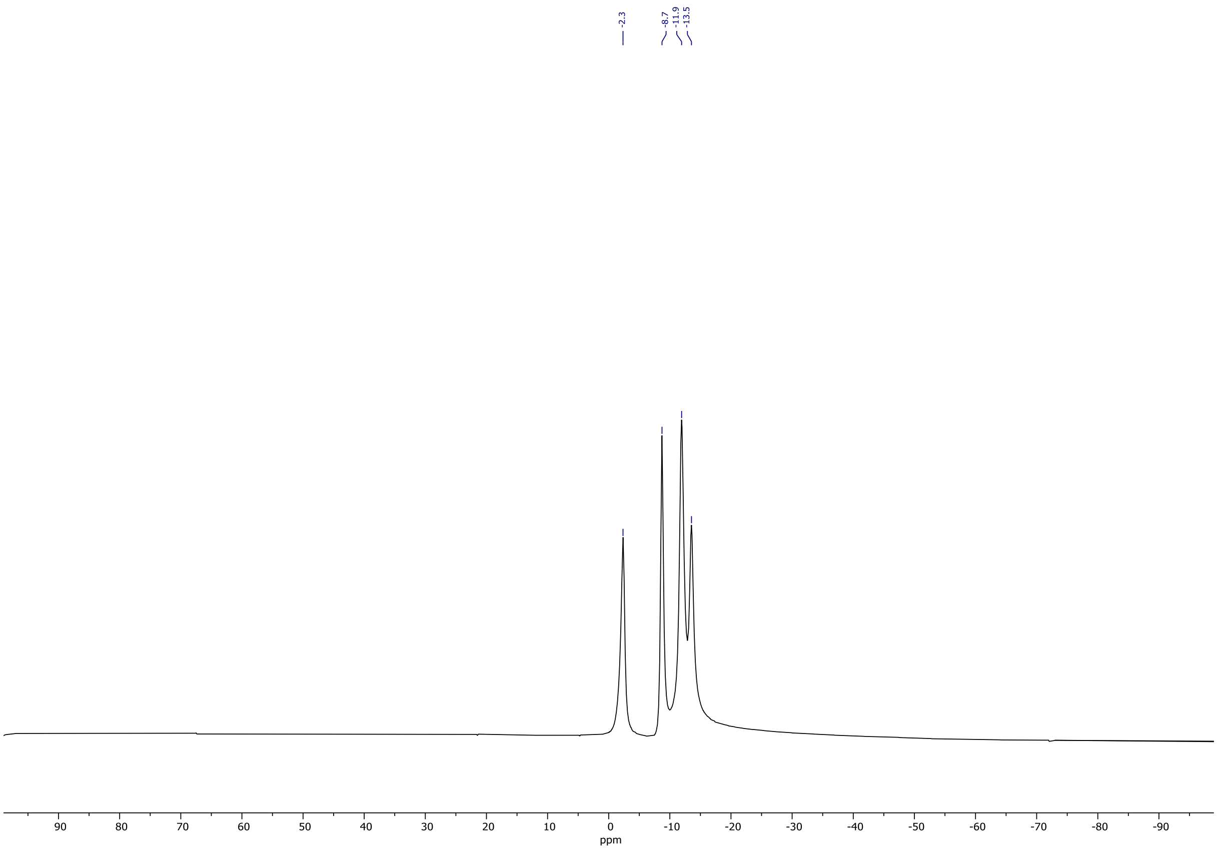

¹H NMR ($CDCl_3$, 400 MHz) for **3a**

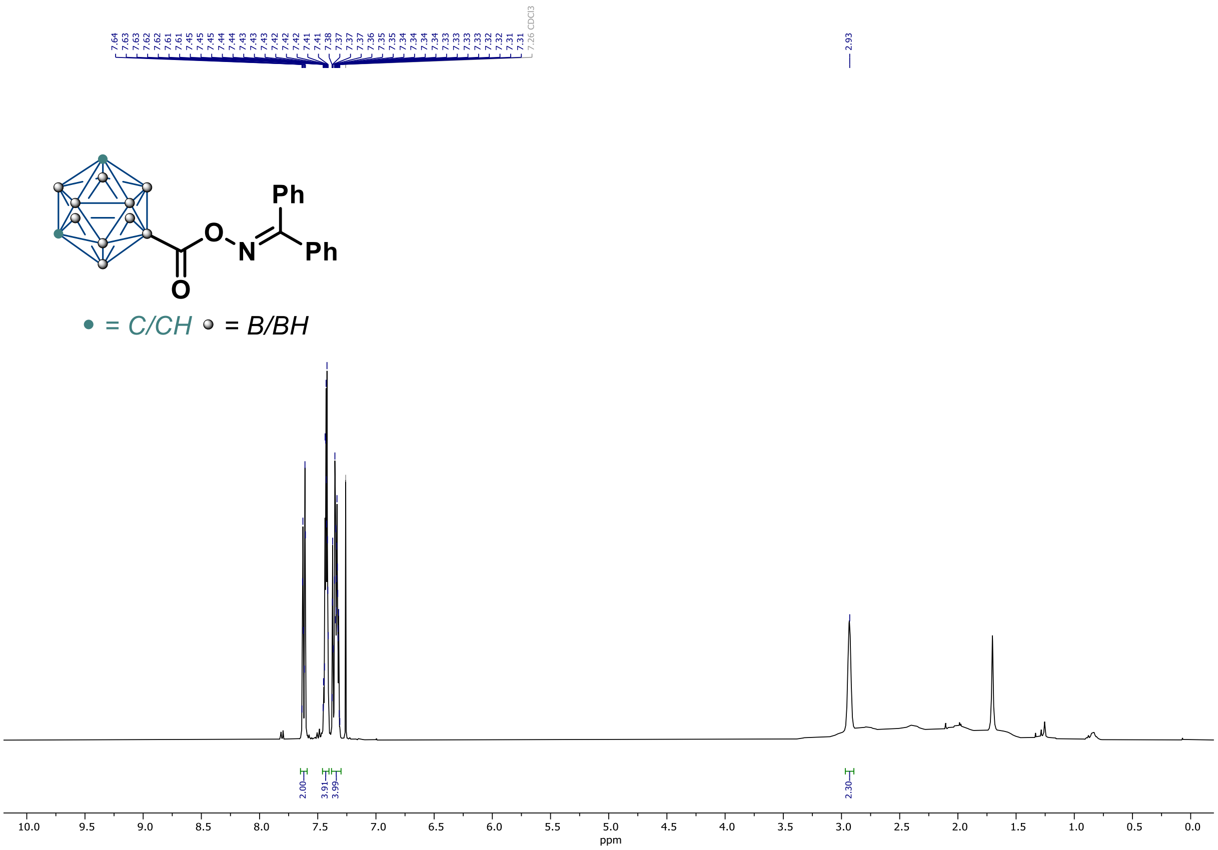

¹³C{¹H} NMR ($CDCl_3$, 101 MHz) for **3a**

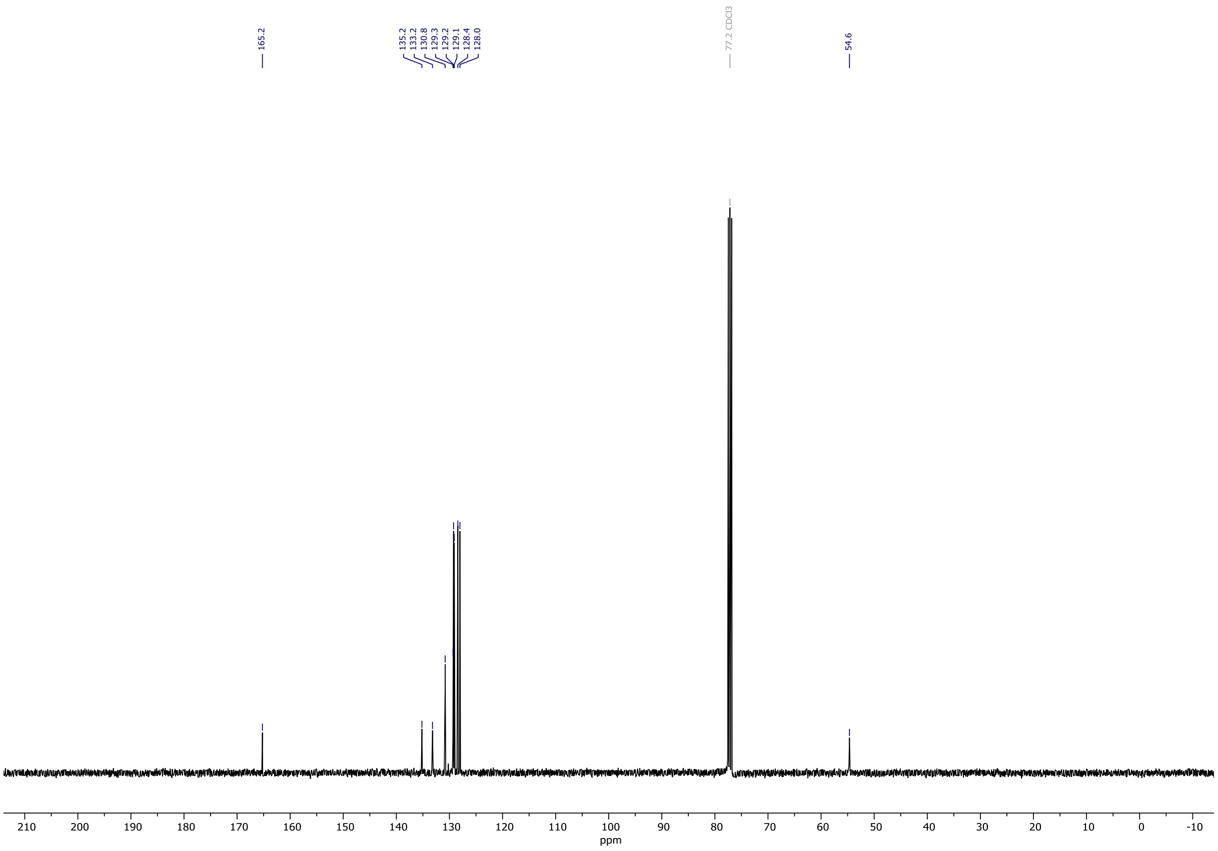

^{11}B{^{1}H} NMR (CDCl$_3$, 128 MHz) for **3a**

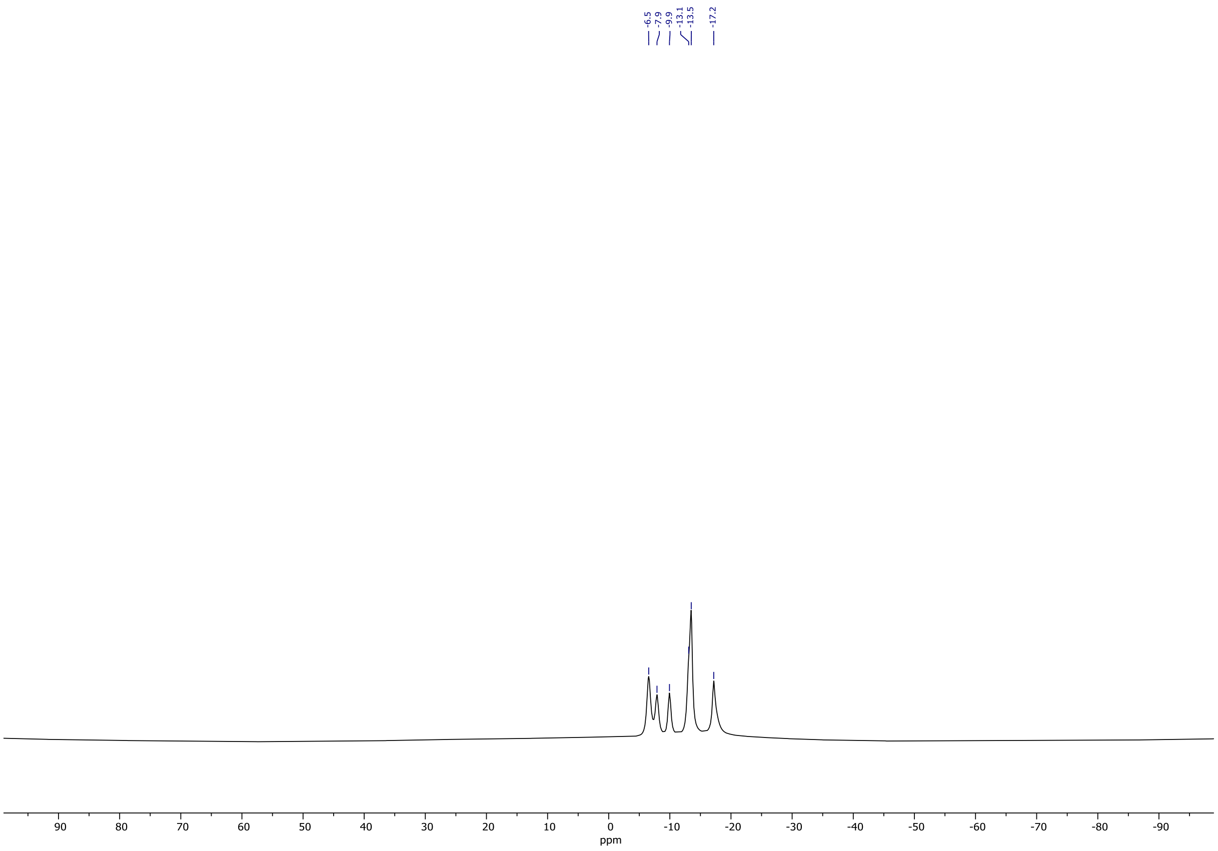

^{1}**H NMR** (CDCl$_3$, 400 MHz) for **3b**

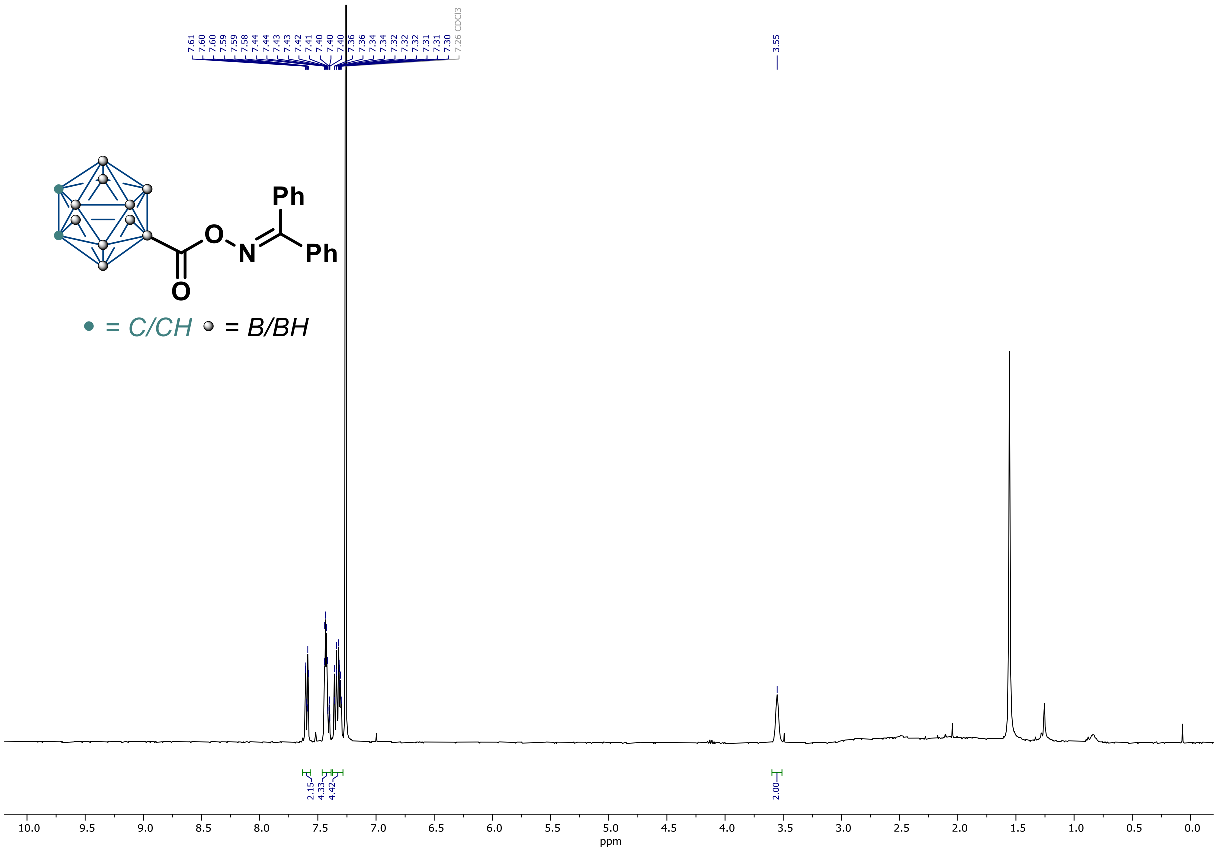

^{13}**C{**^{1}**H} NMR** (CDCl$_3$, 101 MHz) for **3b**

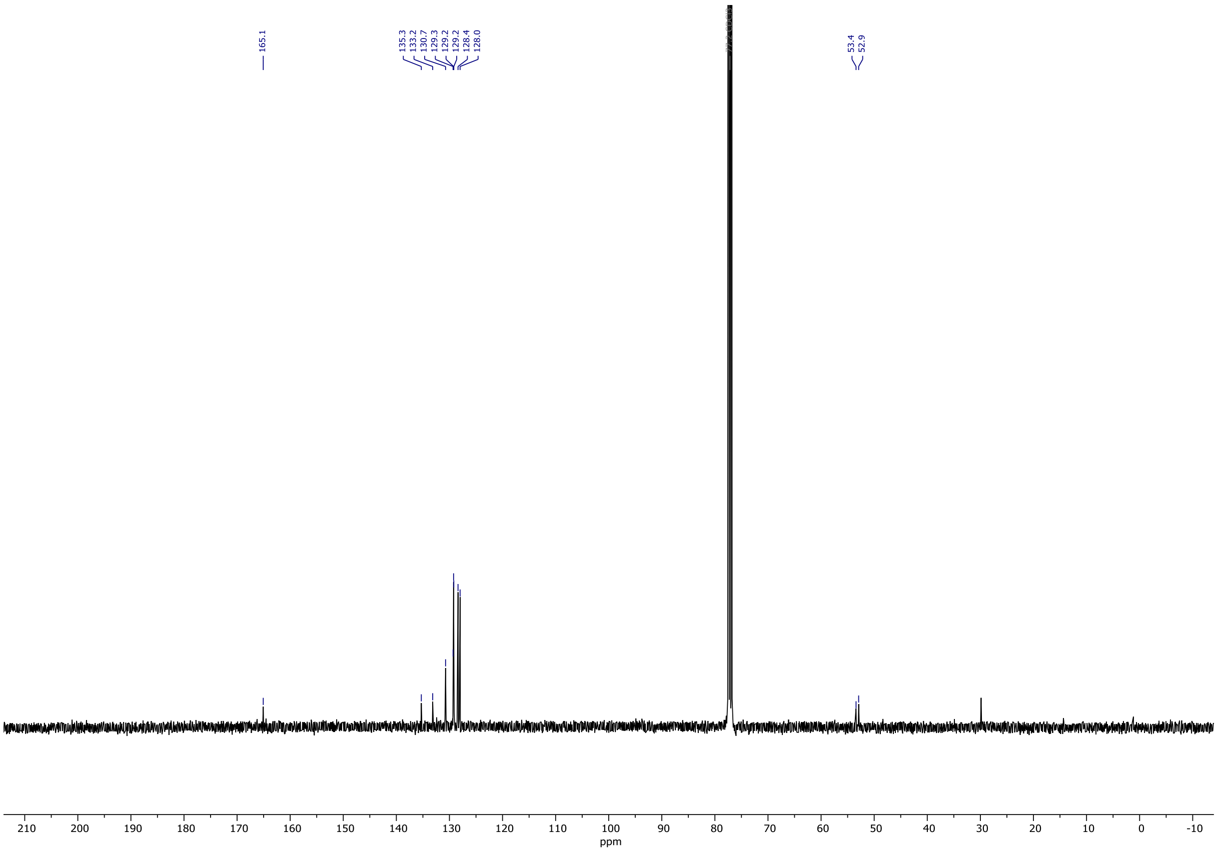

$^{11}B\{^{1}H\}$ NMR ($CDCl_3$, 128 MHz) for **3b**

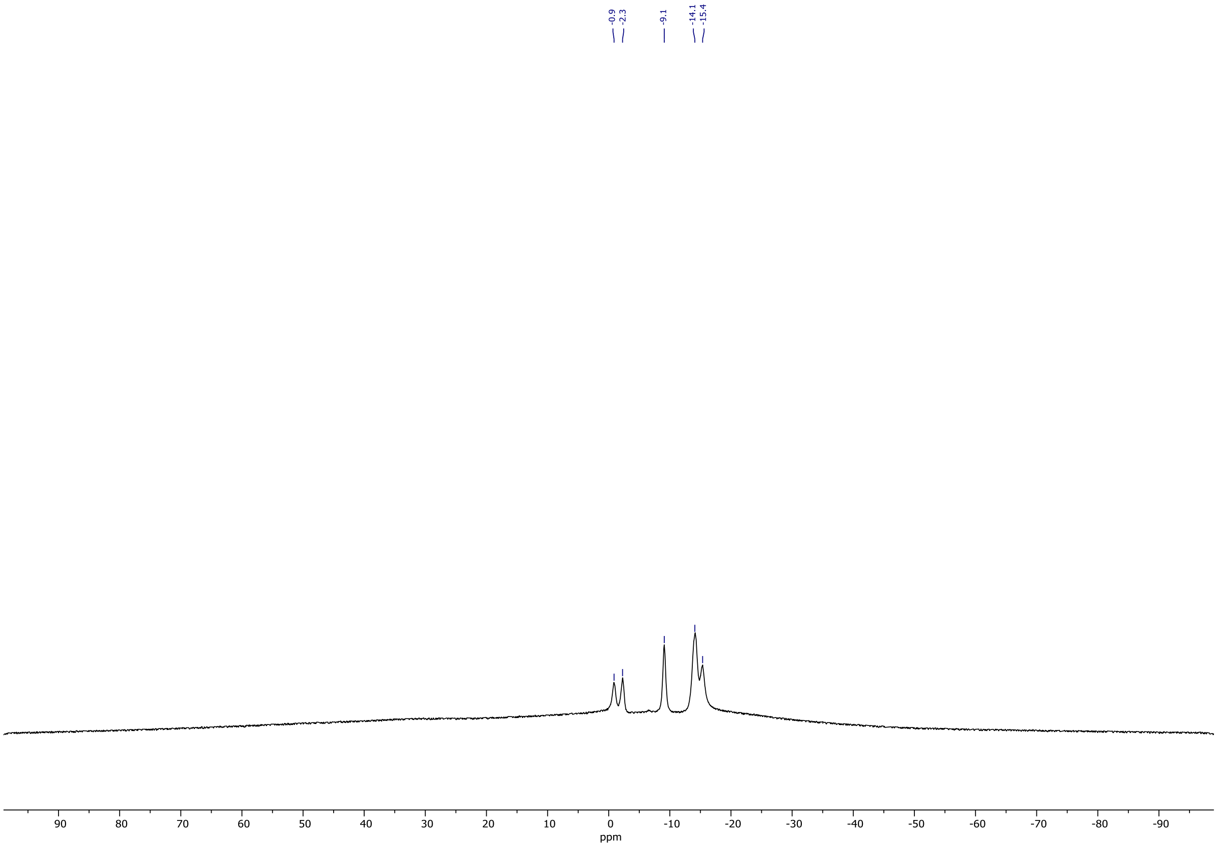

^{1}H NMR (CDCl$_3$, 400 MHz) for **3c**

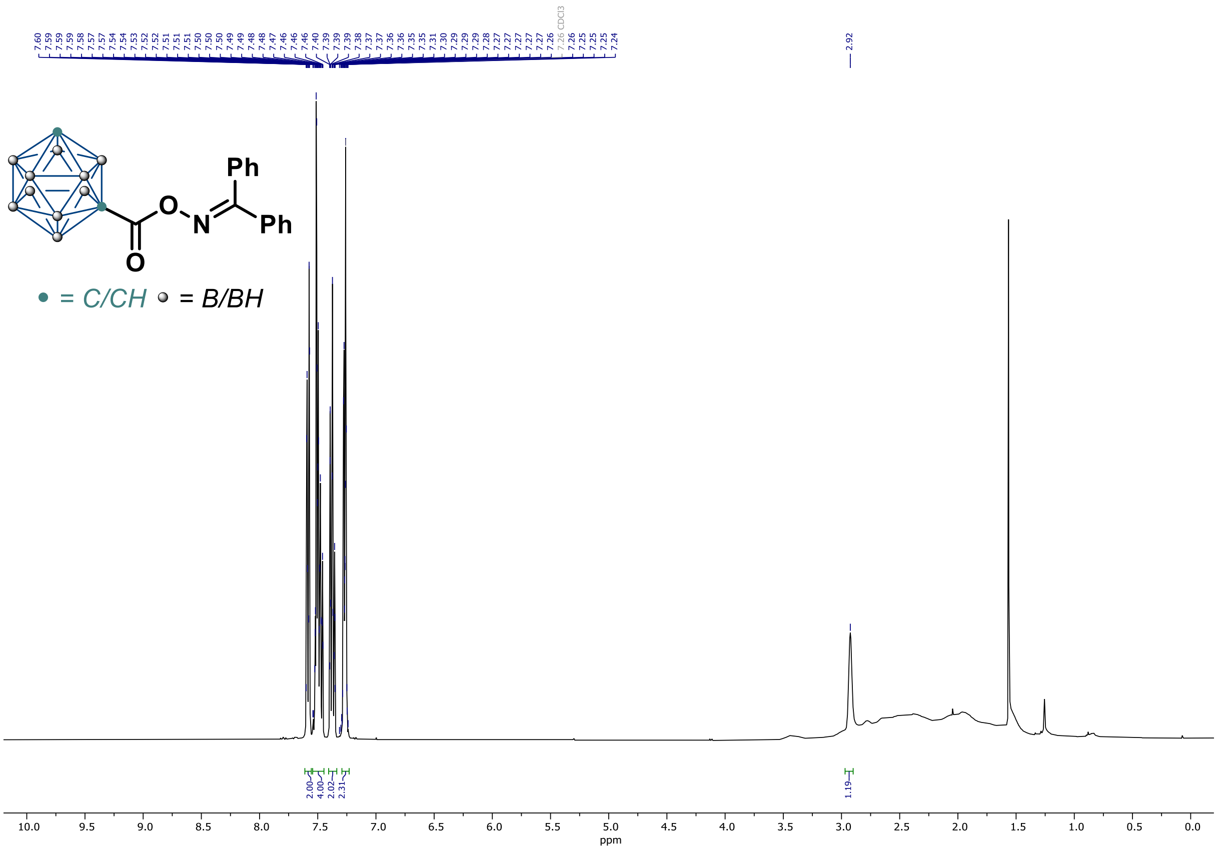

^{13}C{^{1}H} NMR (CDCl$_3$, 101 MHz) for **3c**

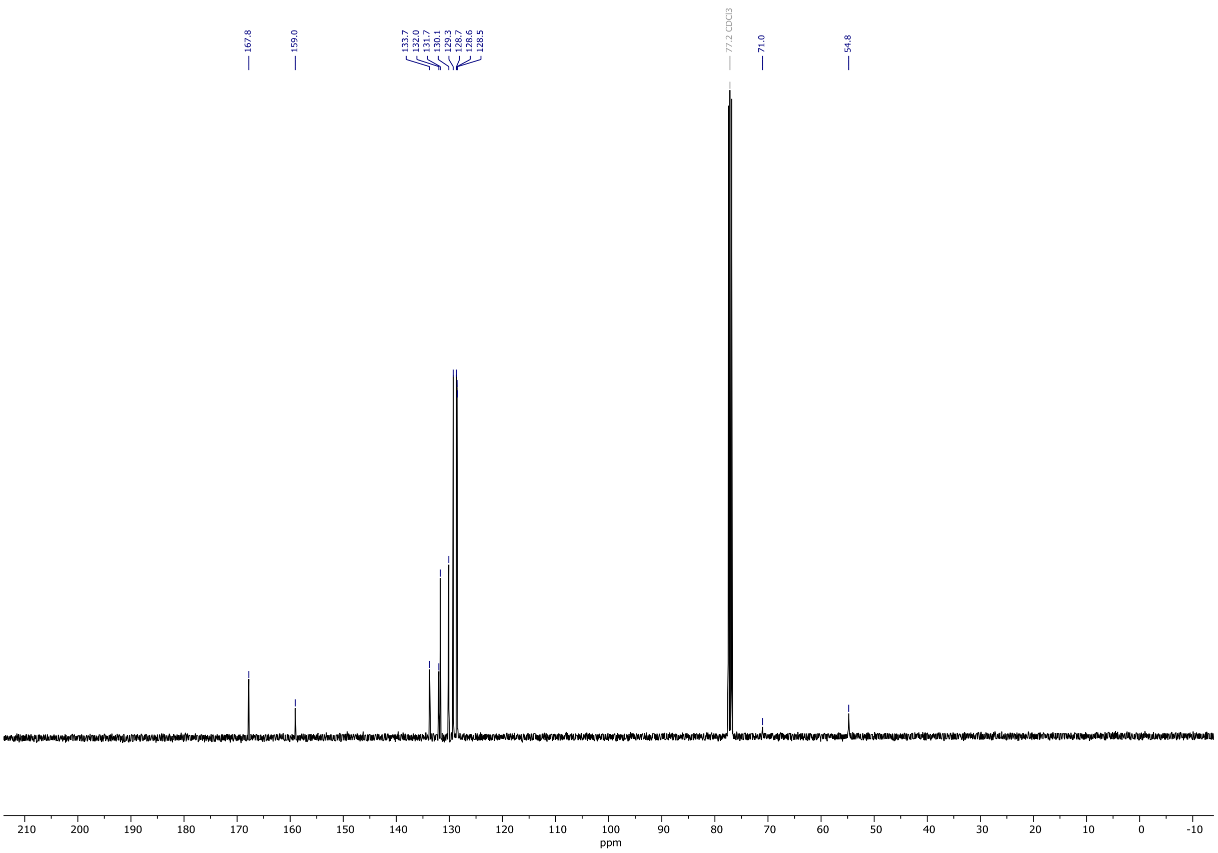

$^{11}B\{^{1}H\}$ NMR (CDCl$_3$, 128 MHz) for **3c**

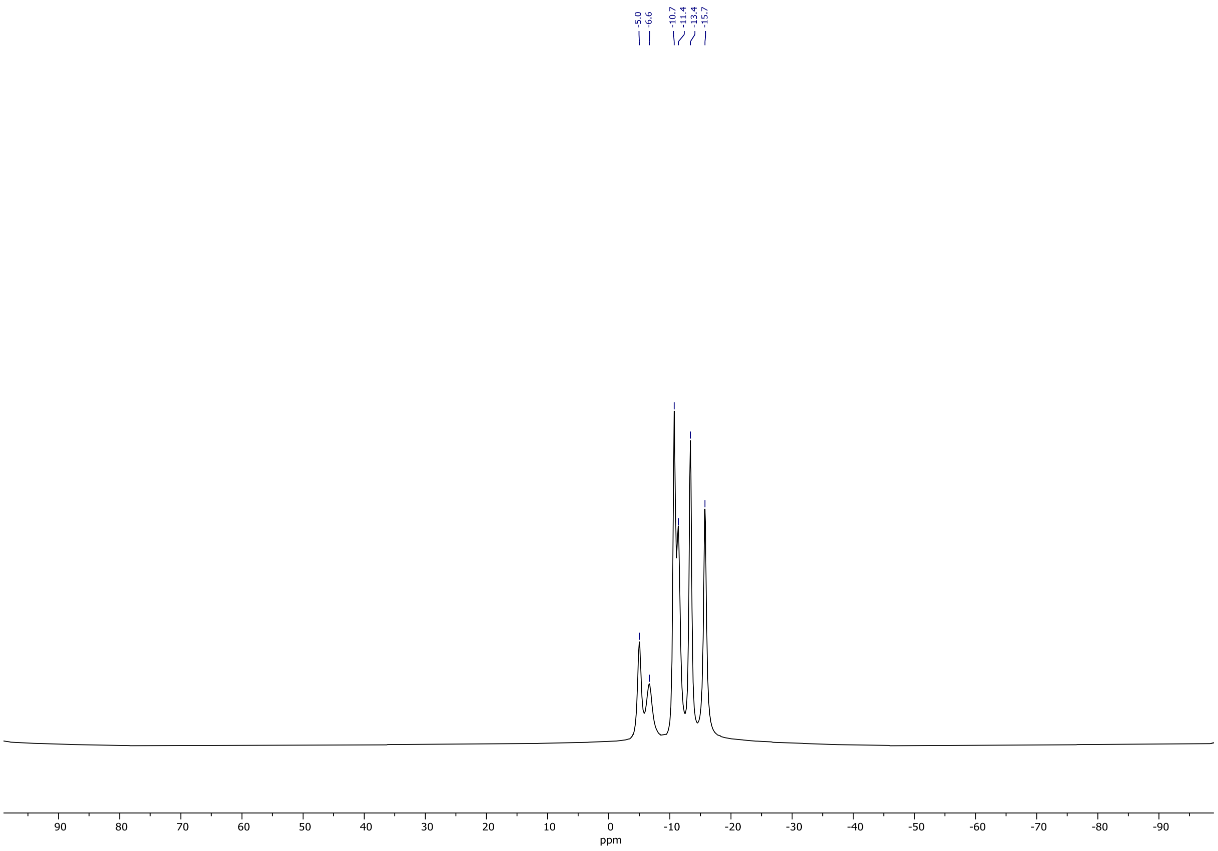

^{1}H NMR (CDCl$_3$, 400 MHz) for **3d**

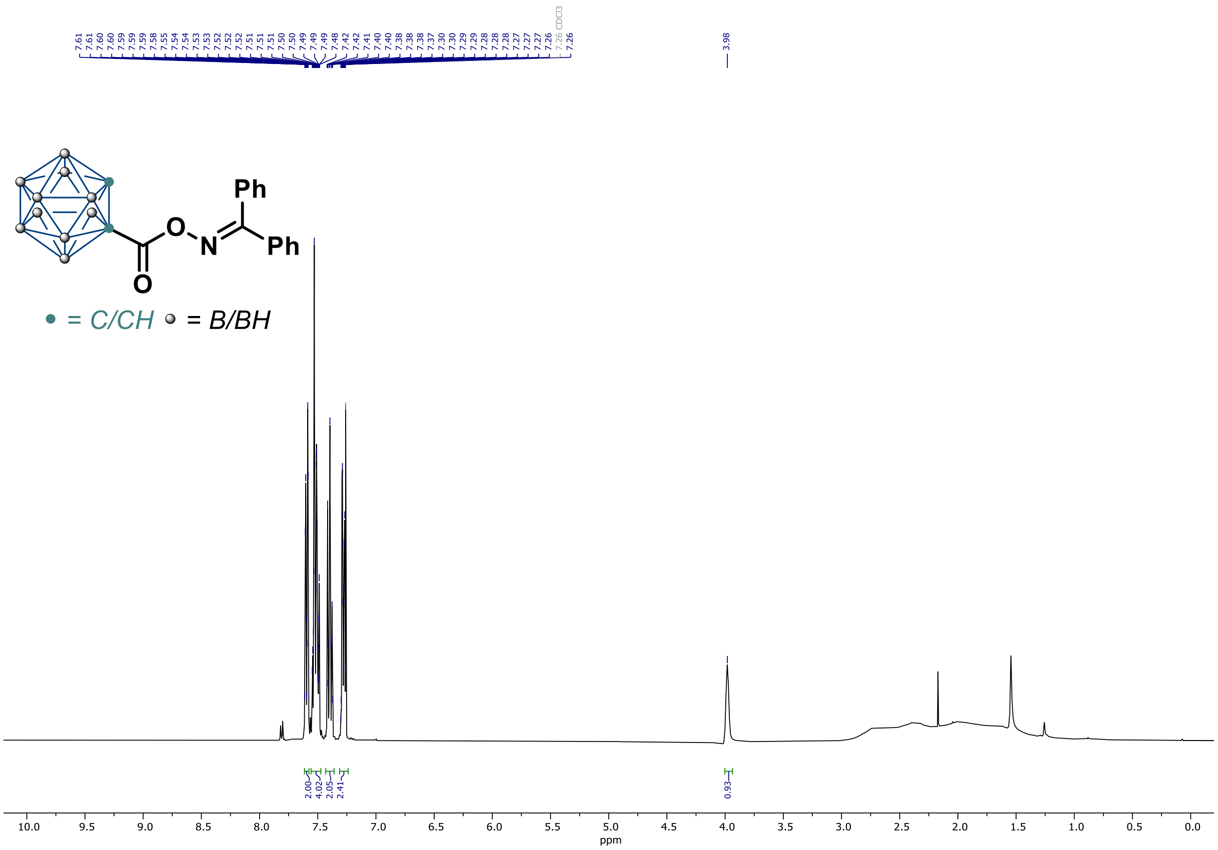

^{13}C{^{1}H} NMR (CDCl$_3$, 101 MHz) for **3d**

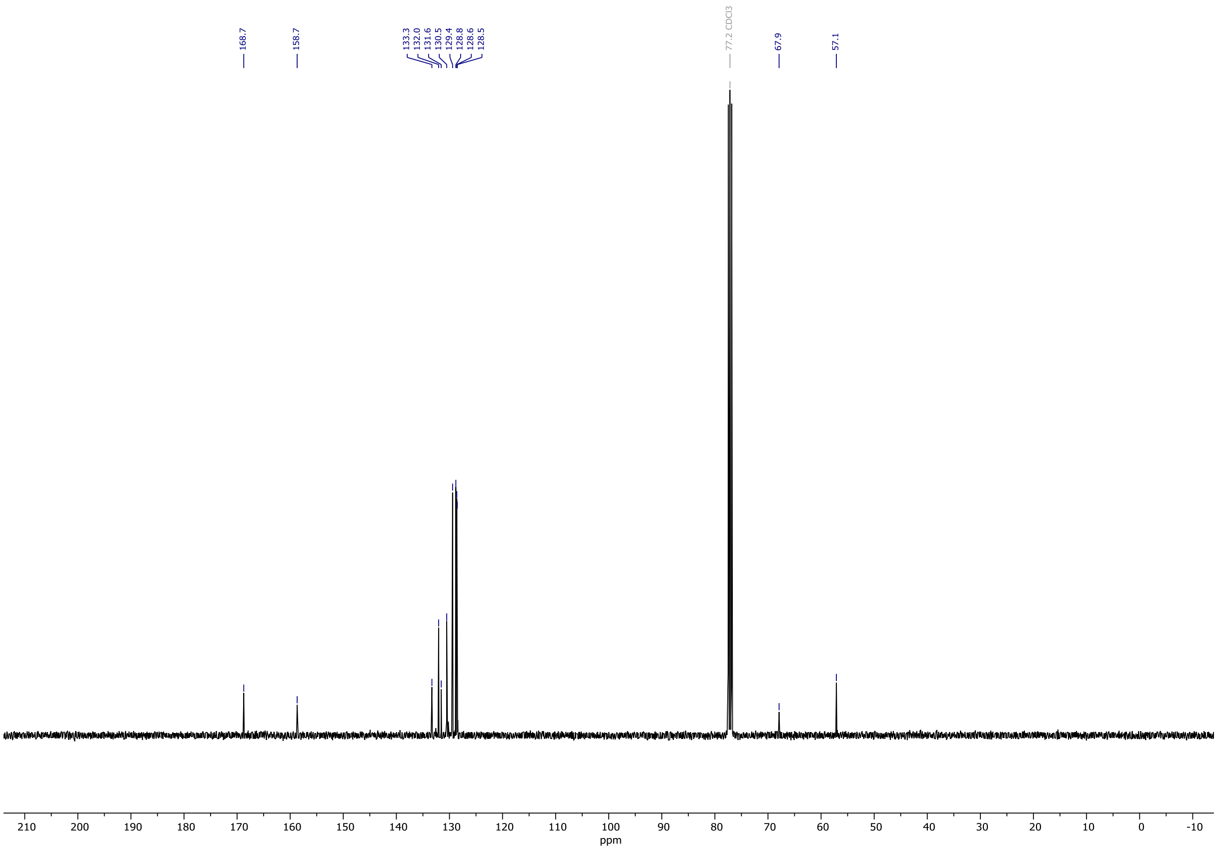

$^{11}B\{^{1}H\}$ NMR ($CDCl_3$, 128 MHz) for **3d**

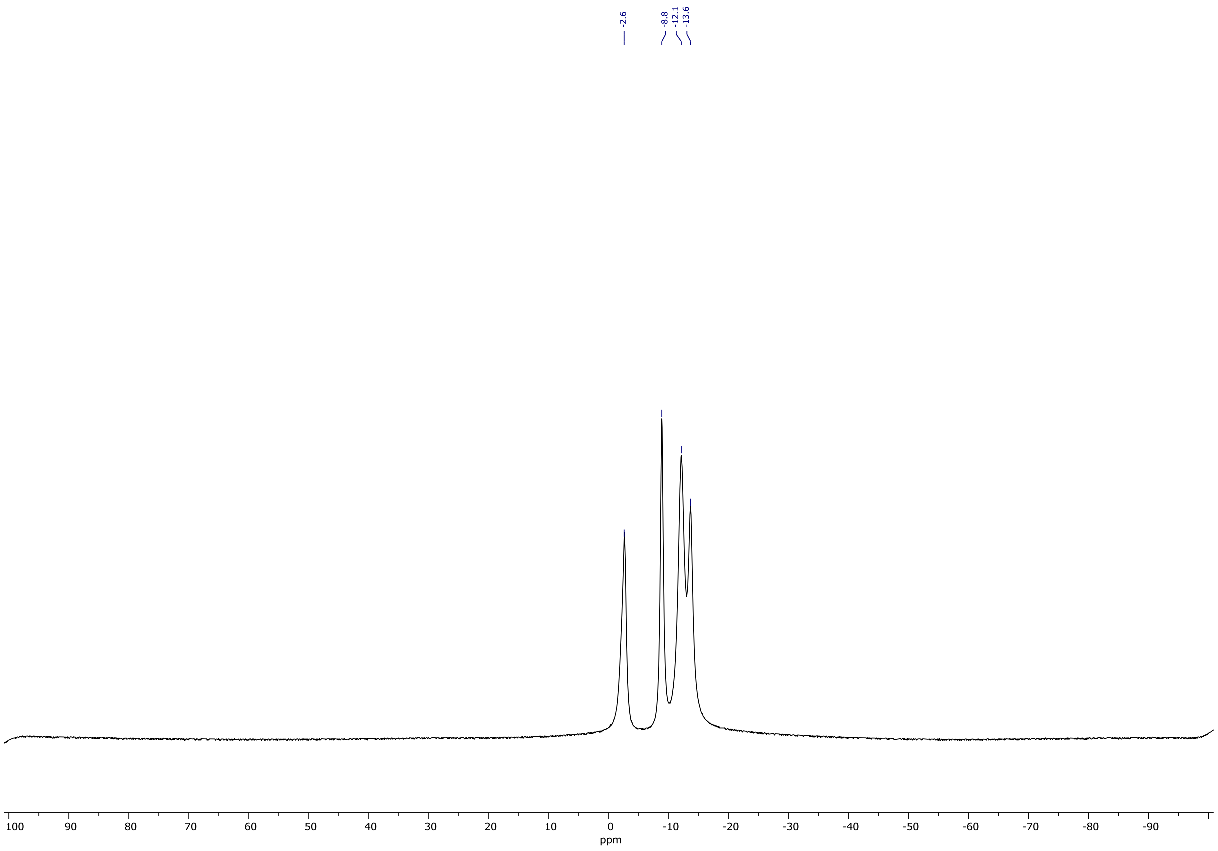

^{1}H NMR ($CDCl_3$, 500 MHz) for **3e**

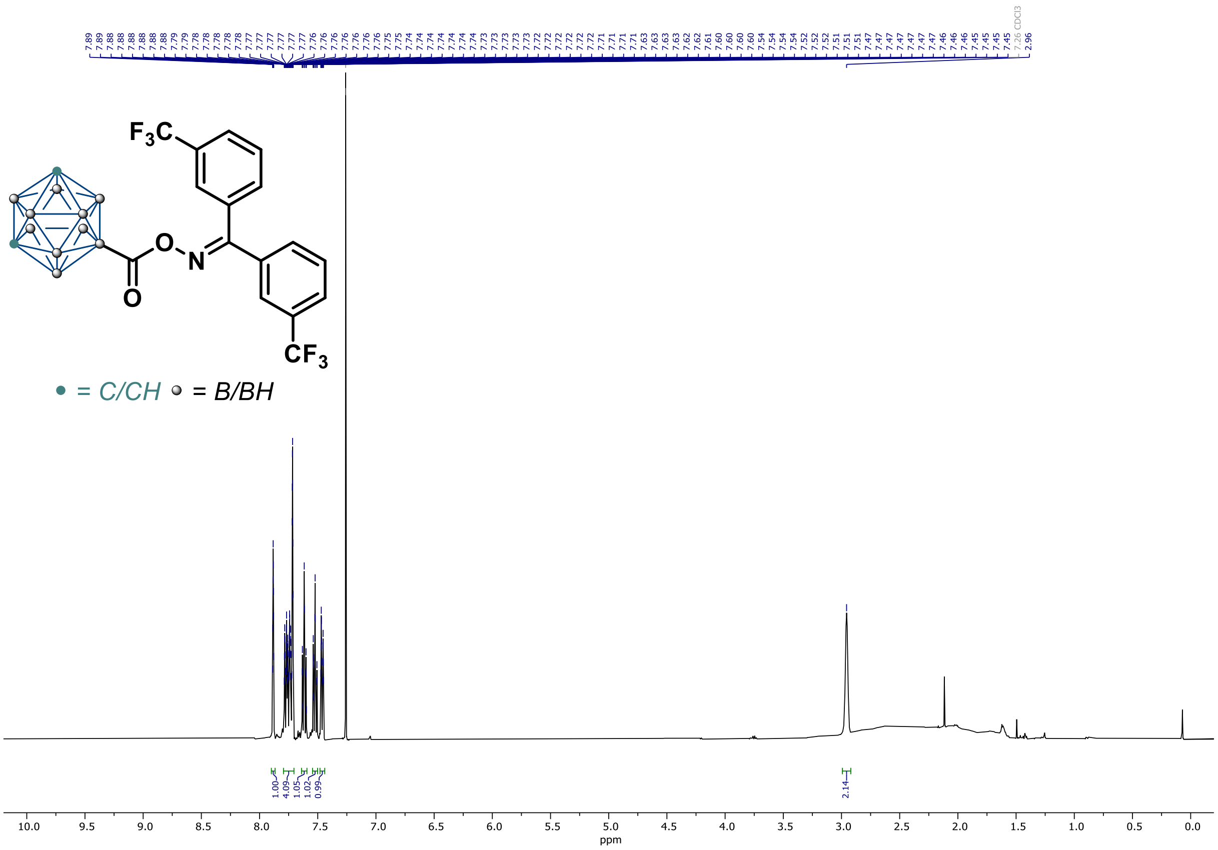

^{13}C{^{1}H} NMR ($CDCl_3$, 126 MHz) for **3e**

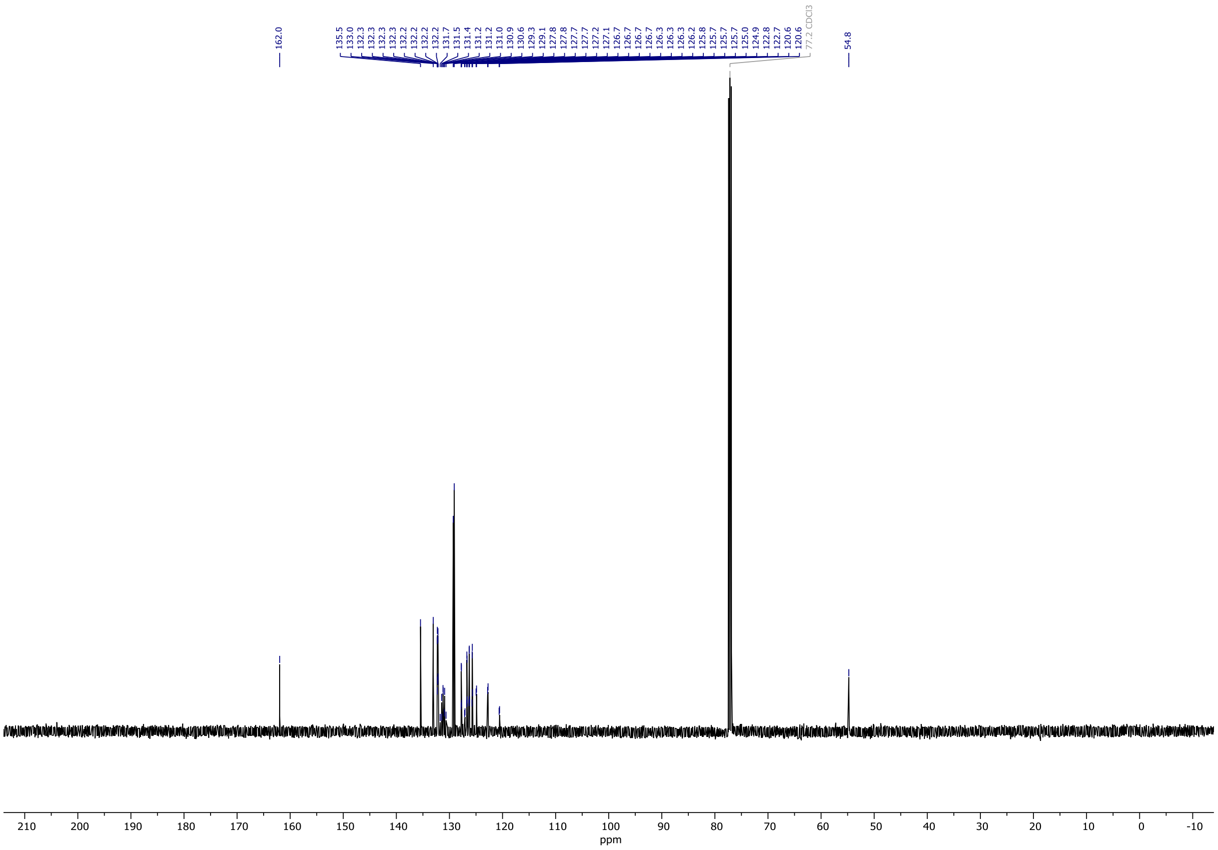

^{11}B{^{1}H} NMR (CDCl$_3$, 160 MHz) for **3e**

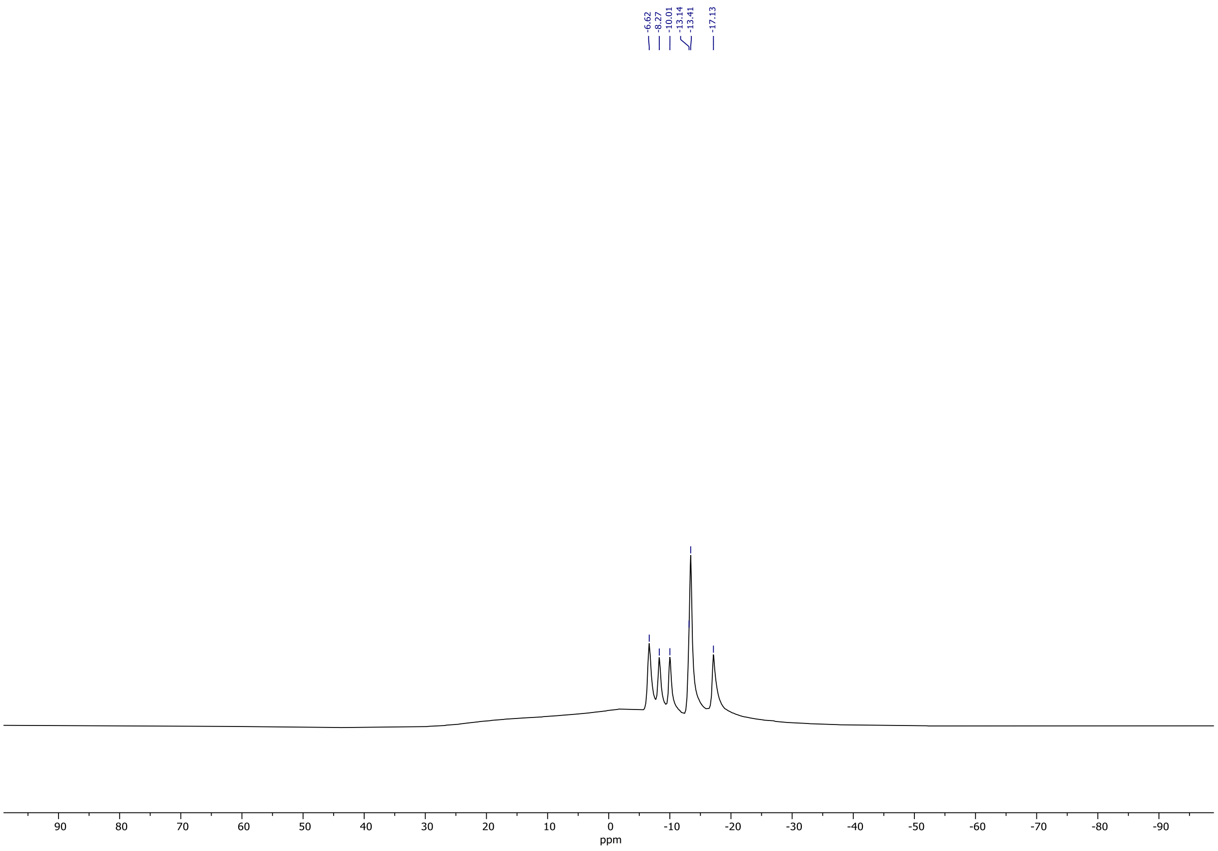

^{19}F{^{1}H} NMR (CDCl$_3$, 470 MHz) for **3e**

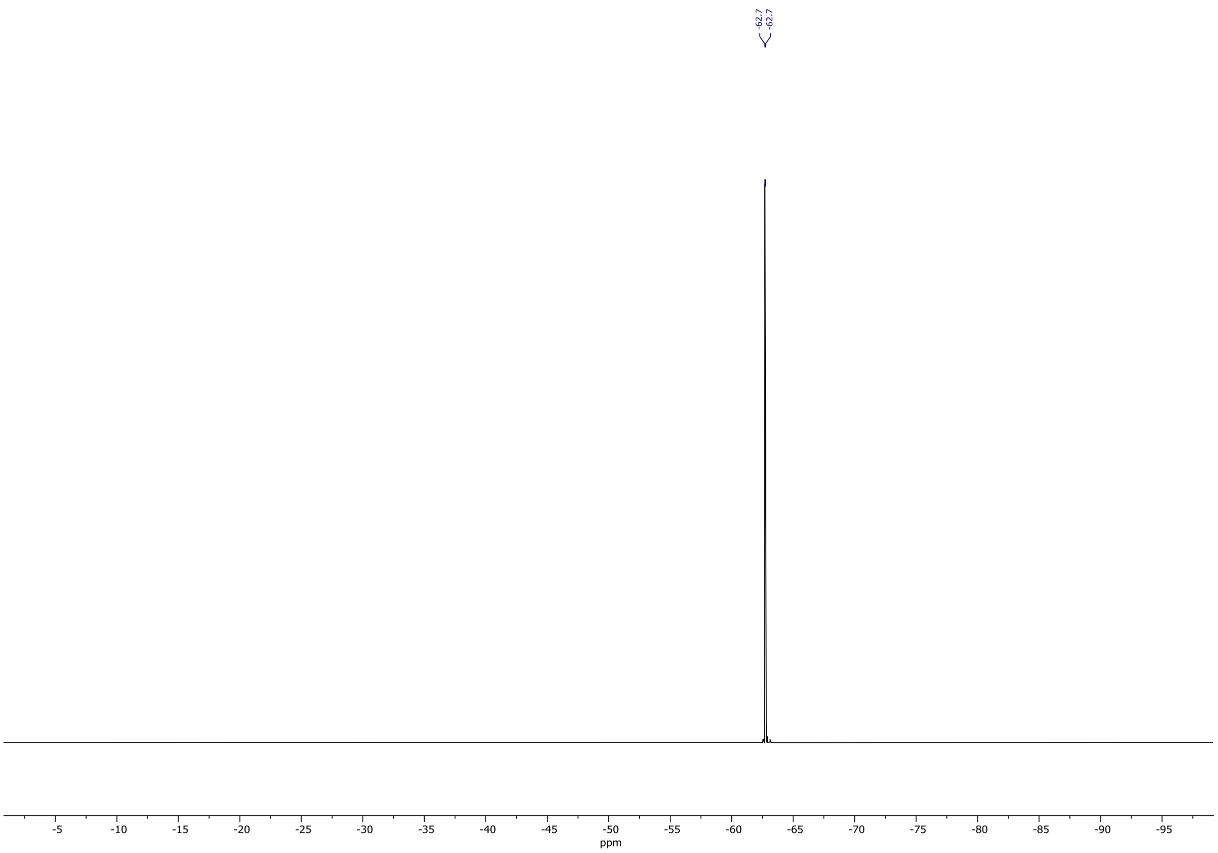

^{1}H NMR (CDCl$_3$, 400 MHz) for **3f**

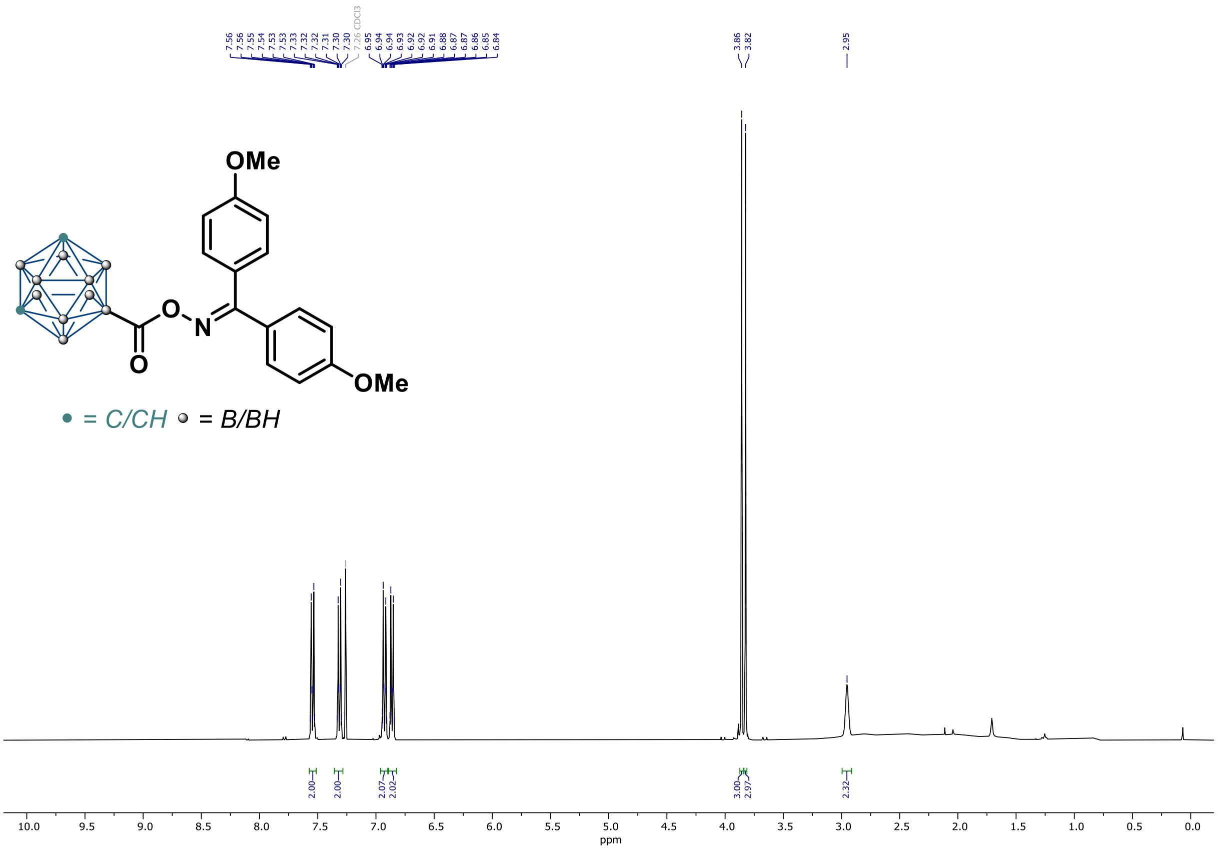

^{13}C{^{1}H} NMR (CDCl$_3$, 101 MHz) for **3f**

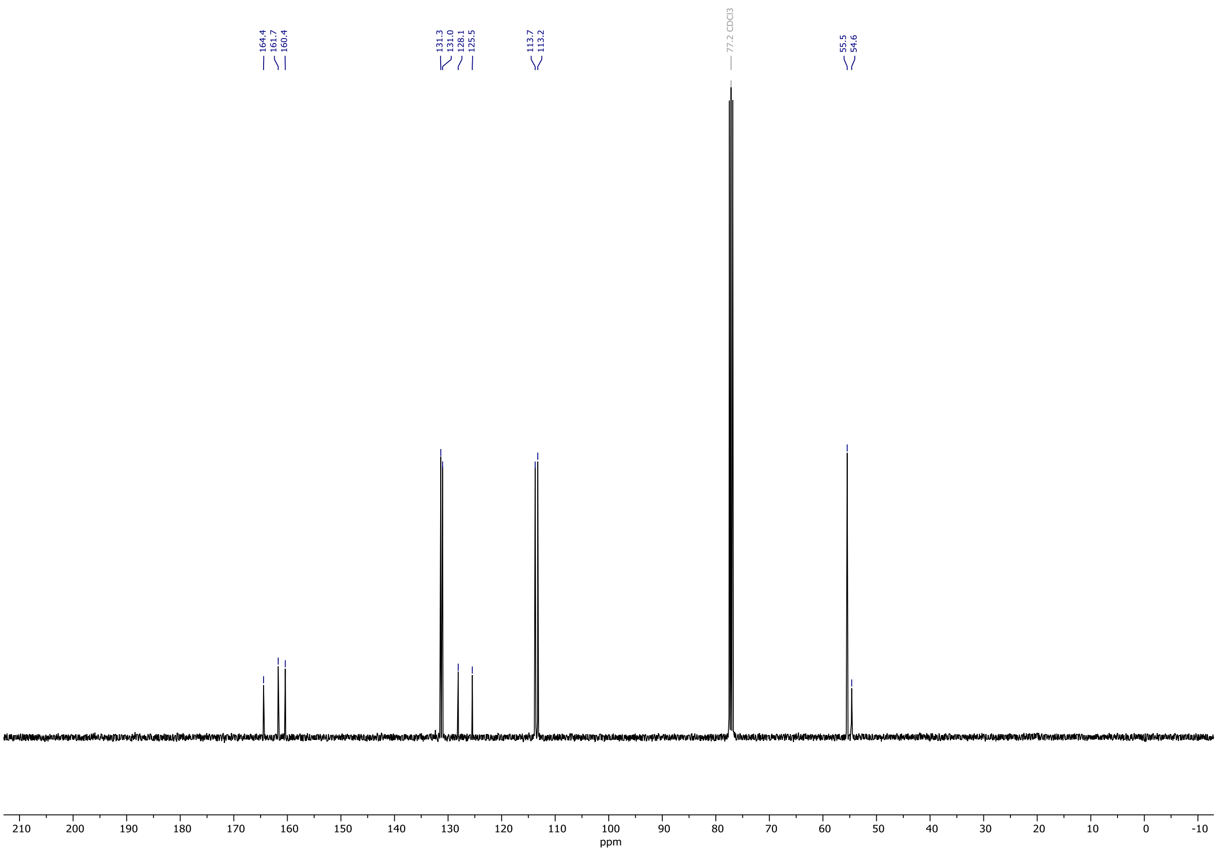

[11]**B{^{1}H} NMR** (CDCl$_3$, 128 MHz) for **3f**

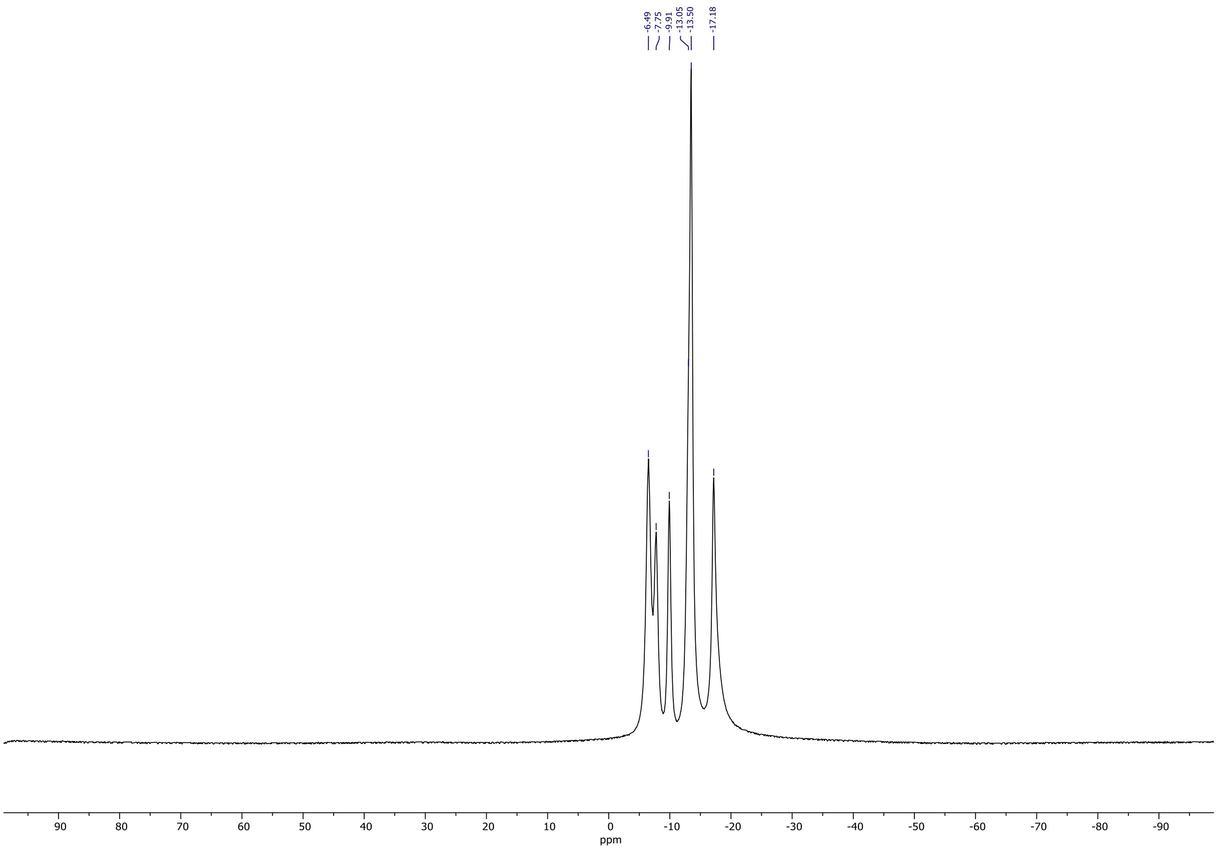

¹H NMR (CDCl$_3$, 400 MHz) for **3g**

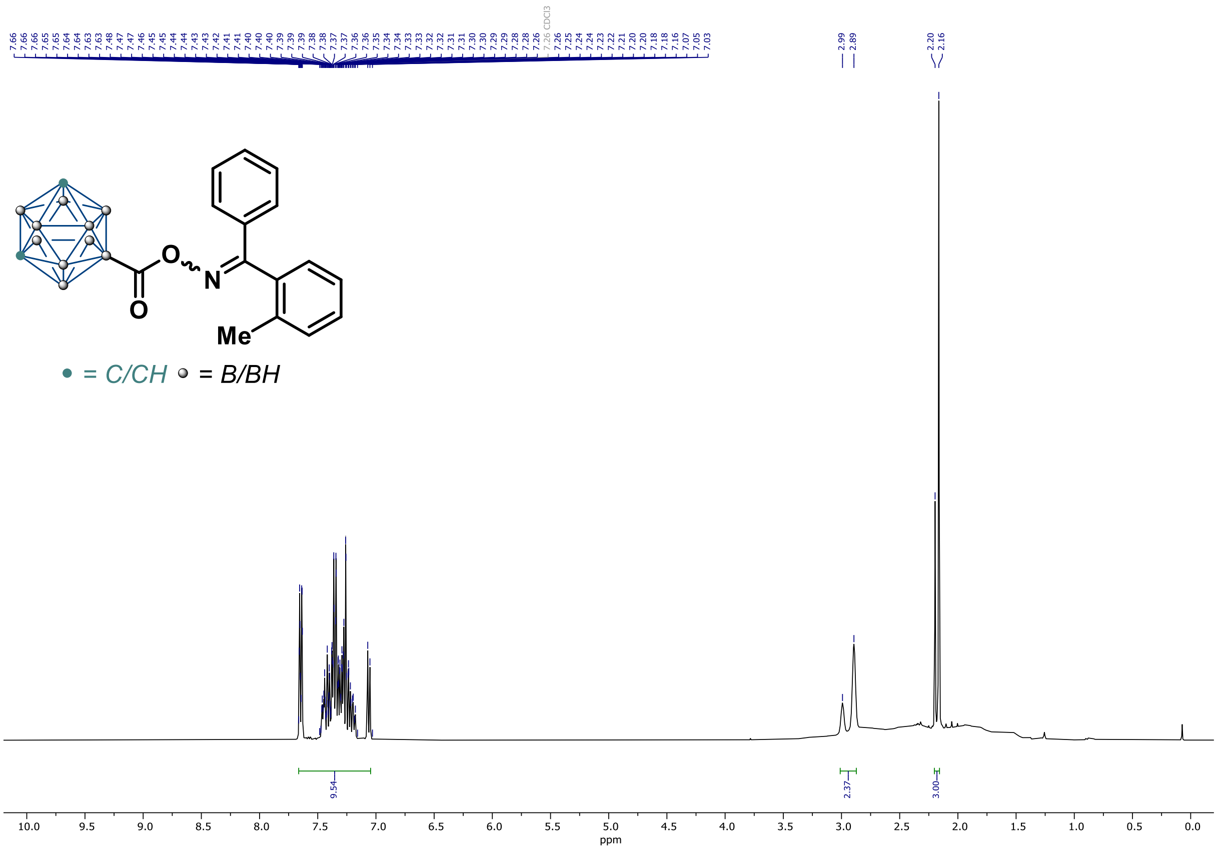

¹³C{¹H} NMR (CDCl$_3$, 101 MHz) for **3g**

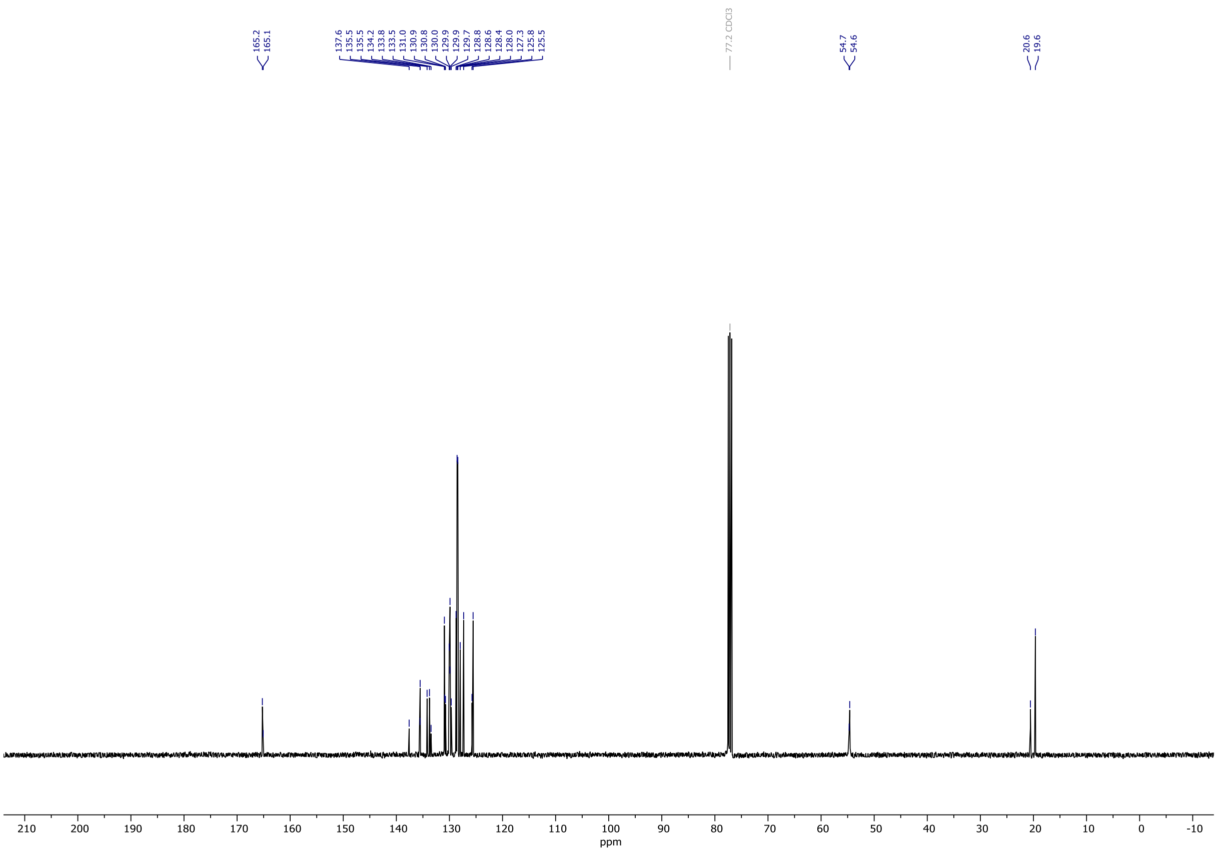

^{11}B{^{1}H} NMR (CDCl$_3$, 128 MHz) for **3g**

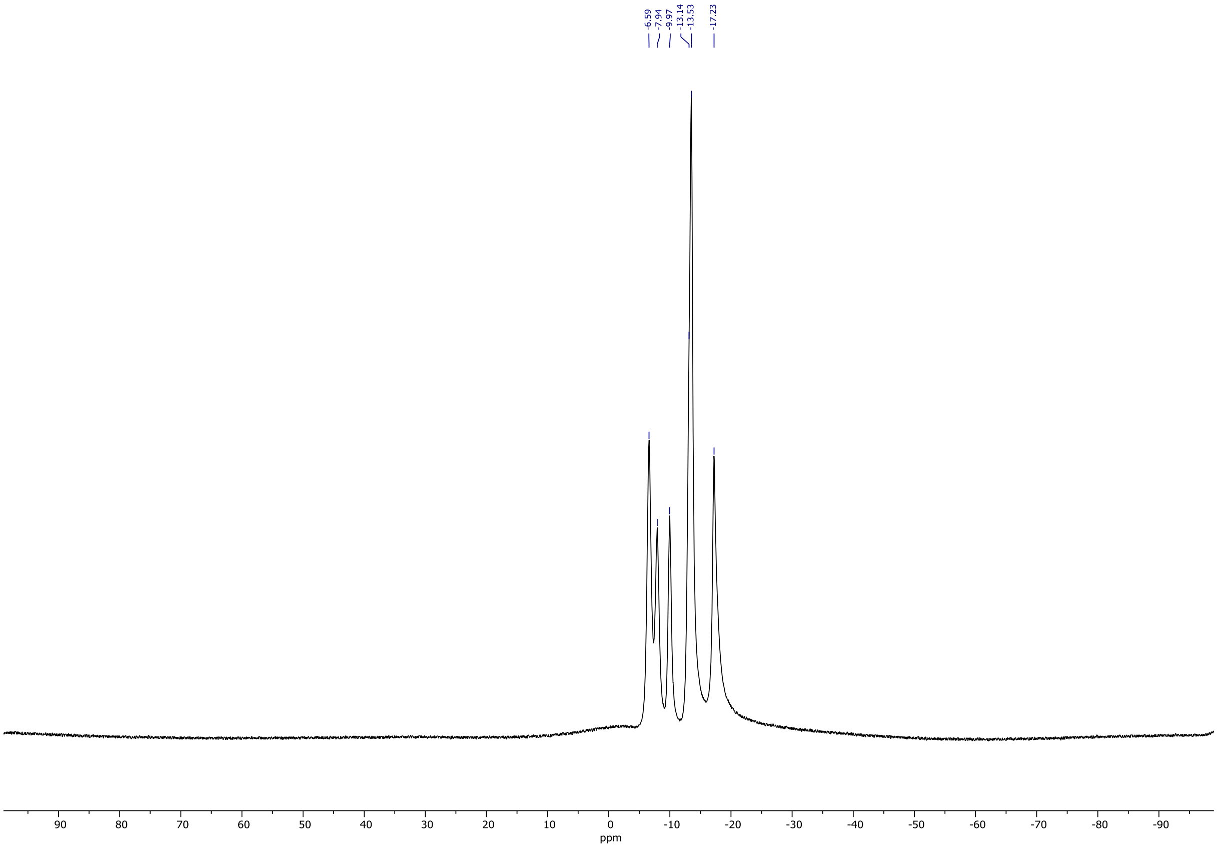

^{1}H NMR ($CDCl_3$, 400 MHz) for **4ai**

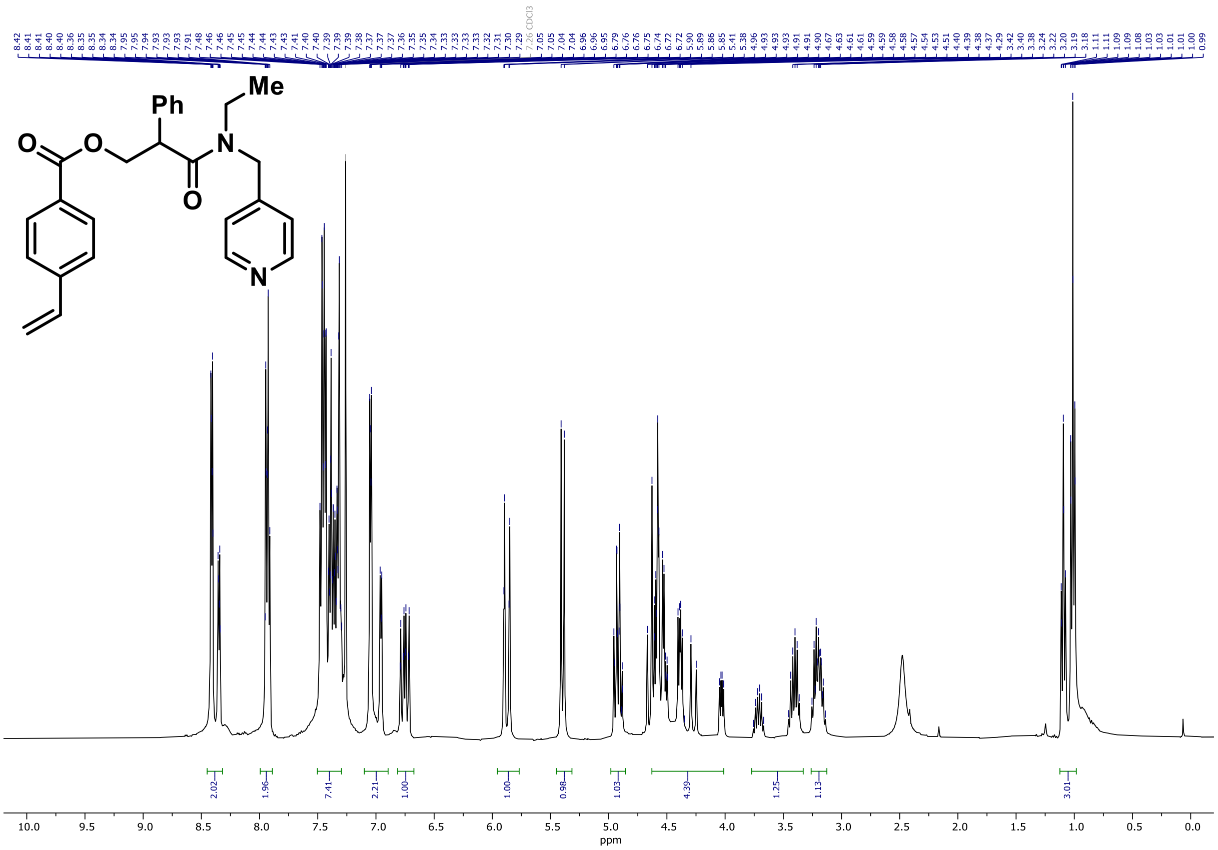

^{13}C{^{1}H} NMR ($CDCl_3$, 101 MHz) for **4ai**

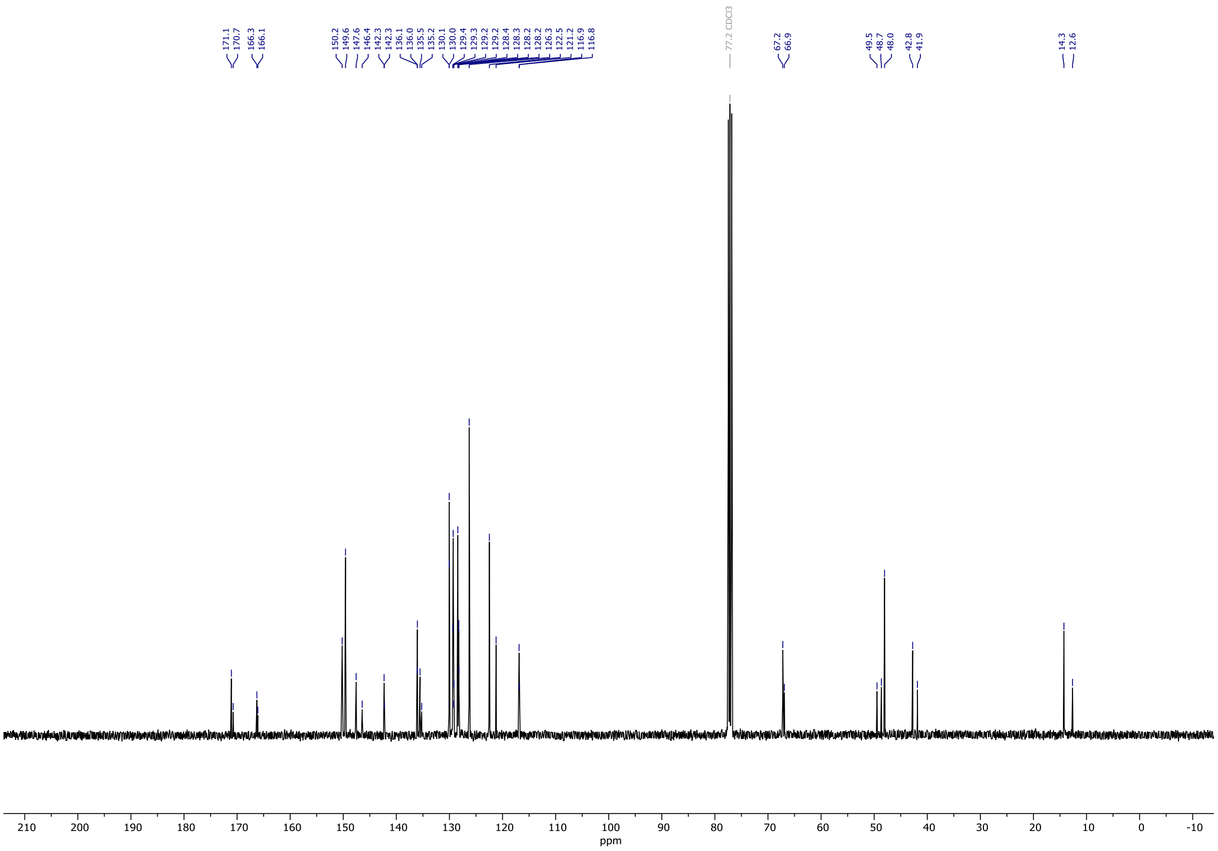

8.2 Substrate scope

¹H NMR ($CDCl_3$, 400 MHz) for **5a**

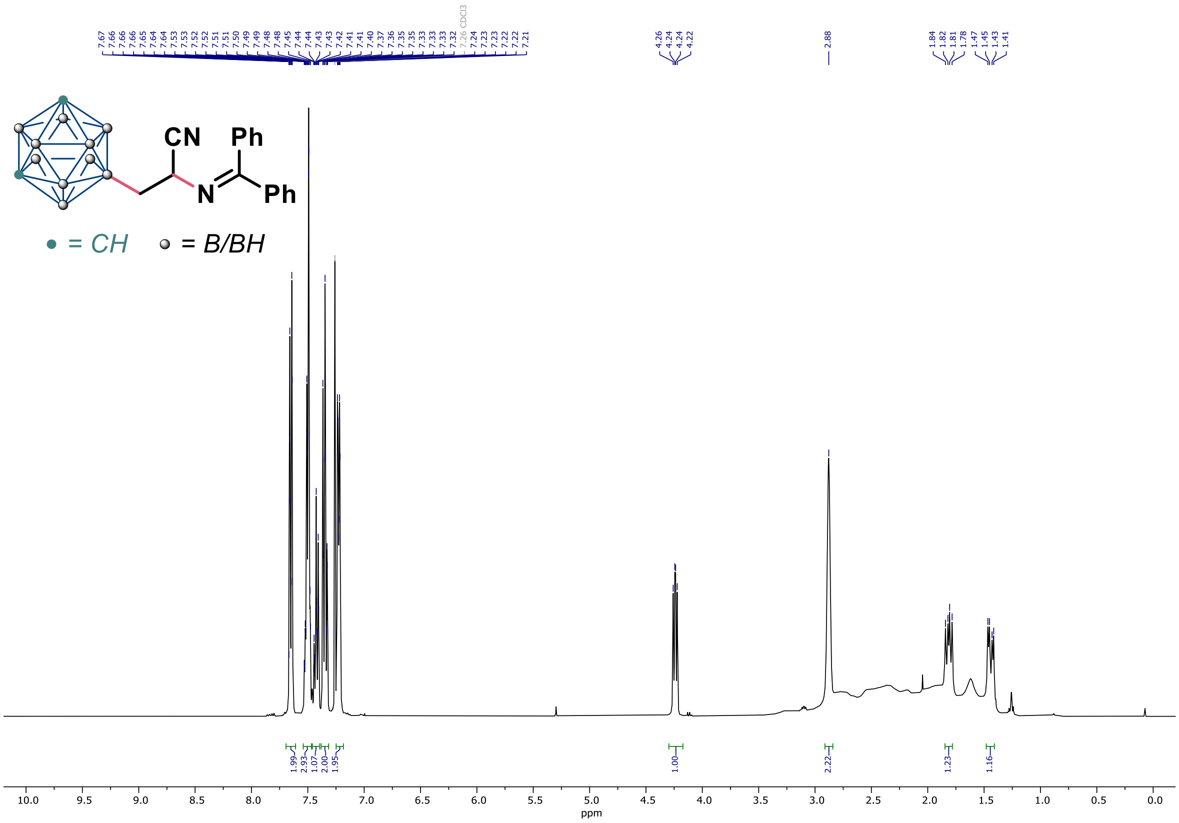

¹³C{¹H} NMR ($CDCl_3$, 101 MHz) for **5a**

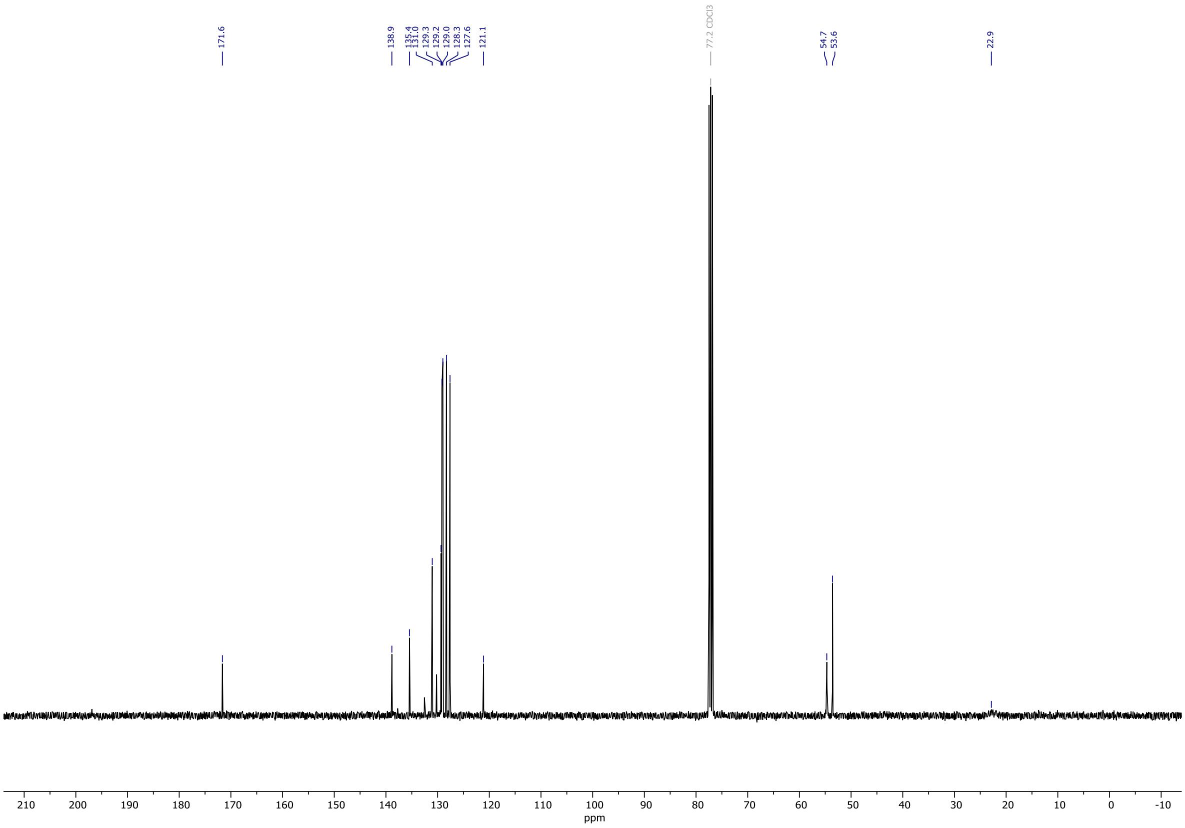

^{11}B{^{1}H} NMR (CDCl$_3$, 128 MHz) for **5a**

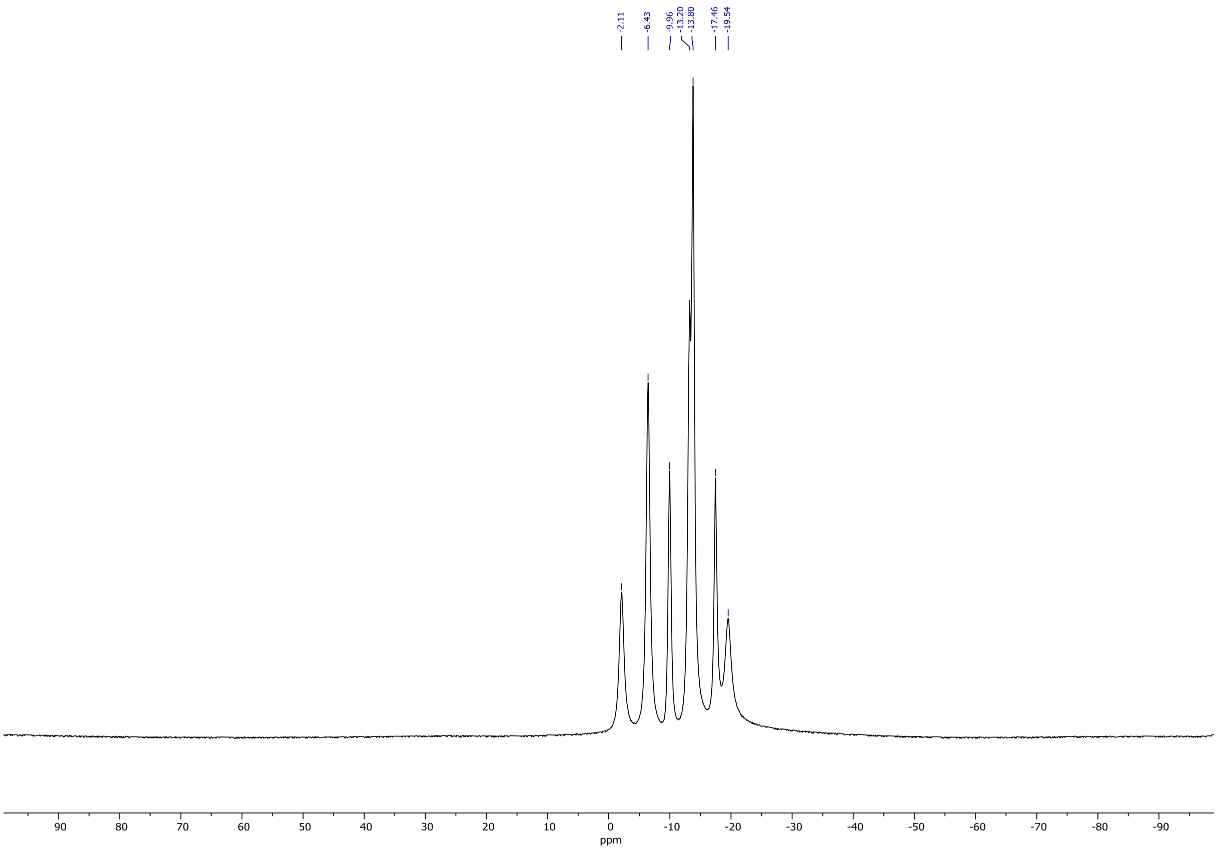

^{1}H NMR (CDCl$_3$, 400 MHz) for **5b**

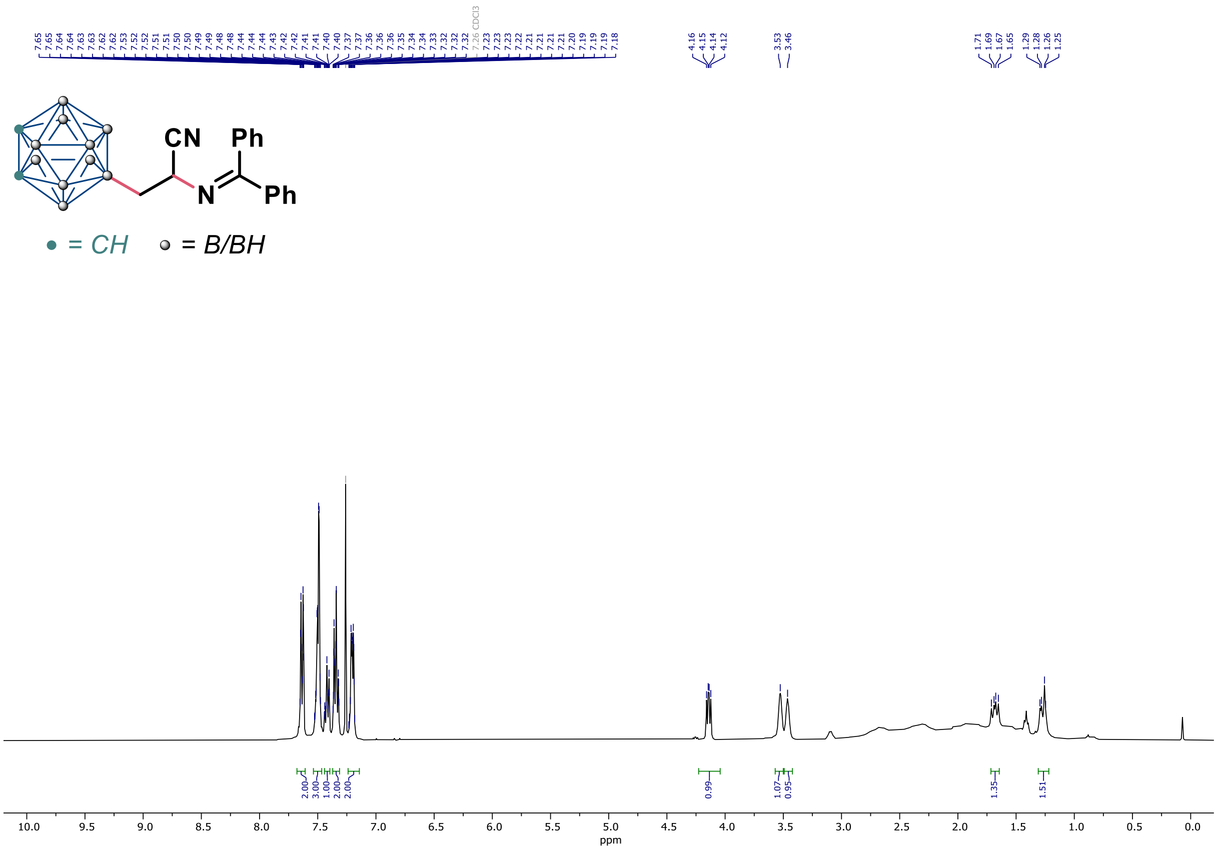

^{13}C{^{1}H} NMR (CDCl$_3$, 101 MHz) for **5b**

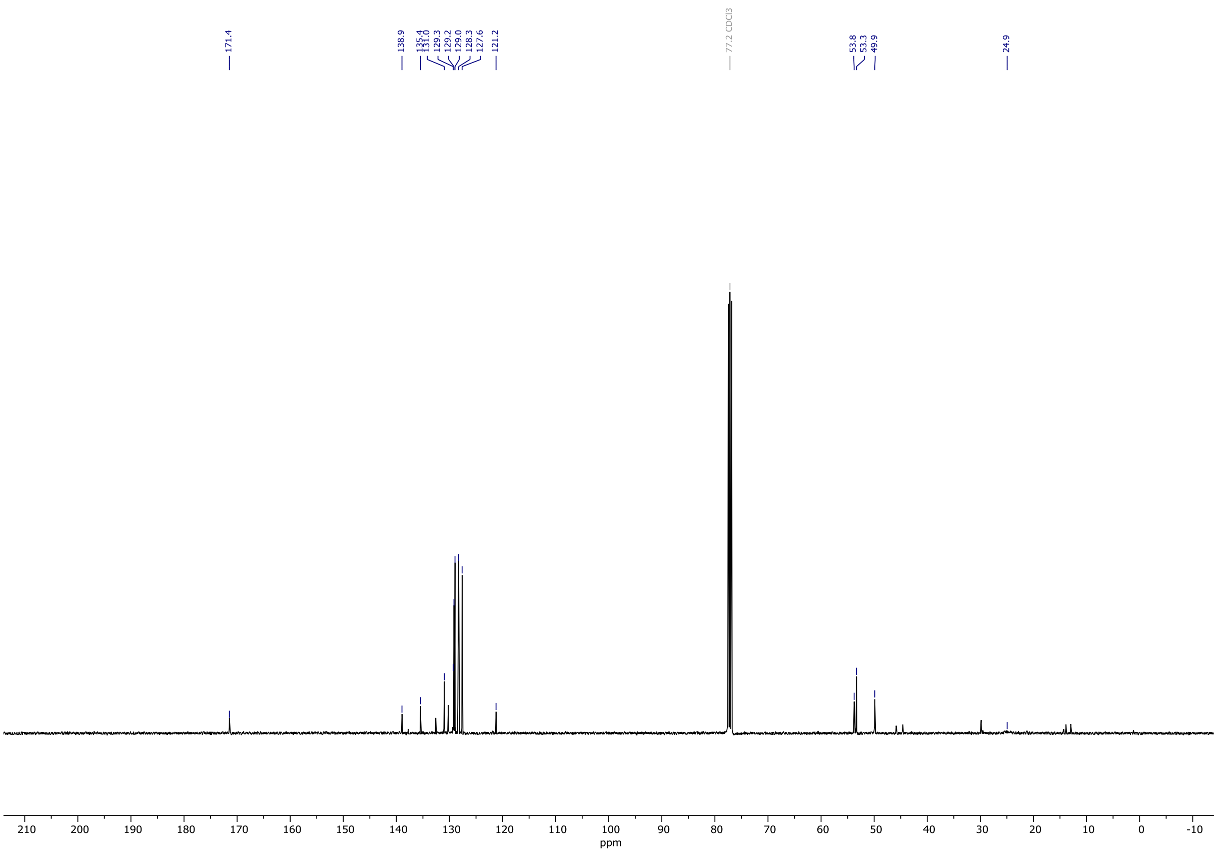

¹¹B{¹H} NMR (CDCl$_3$, 128 MHz) for **5b**

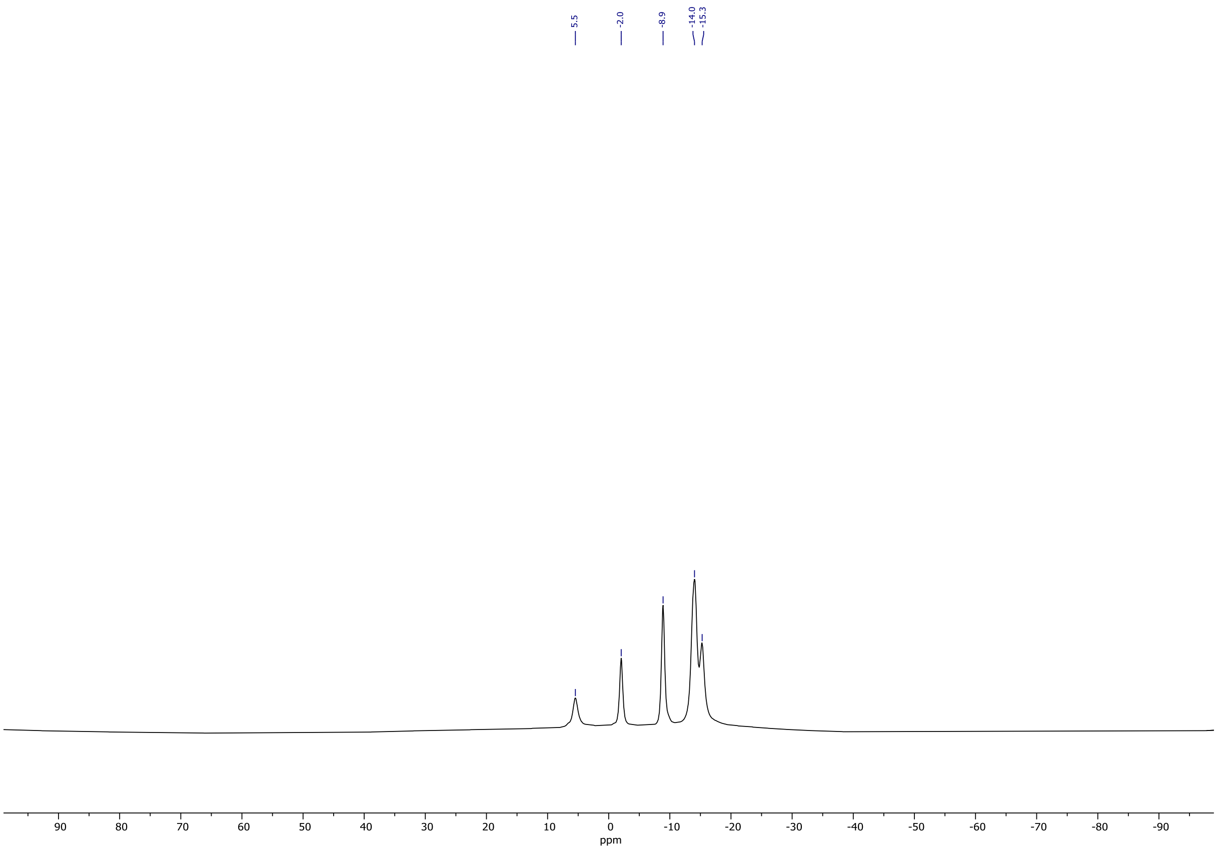

¹H NMR (CDCl₃, 400 MHz) for **5c**

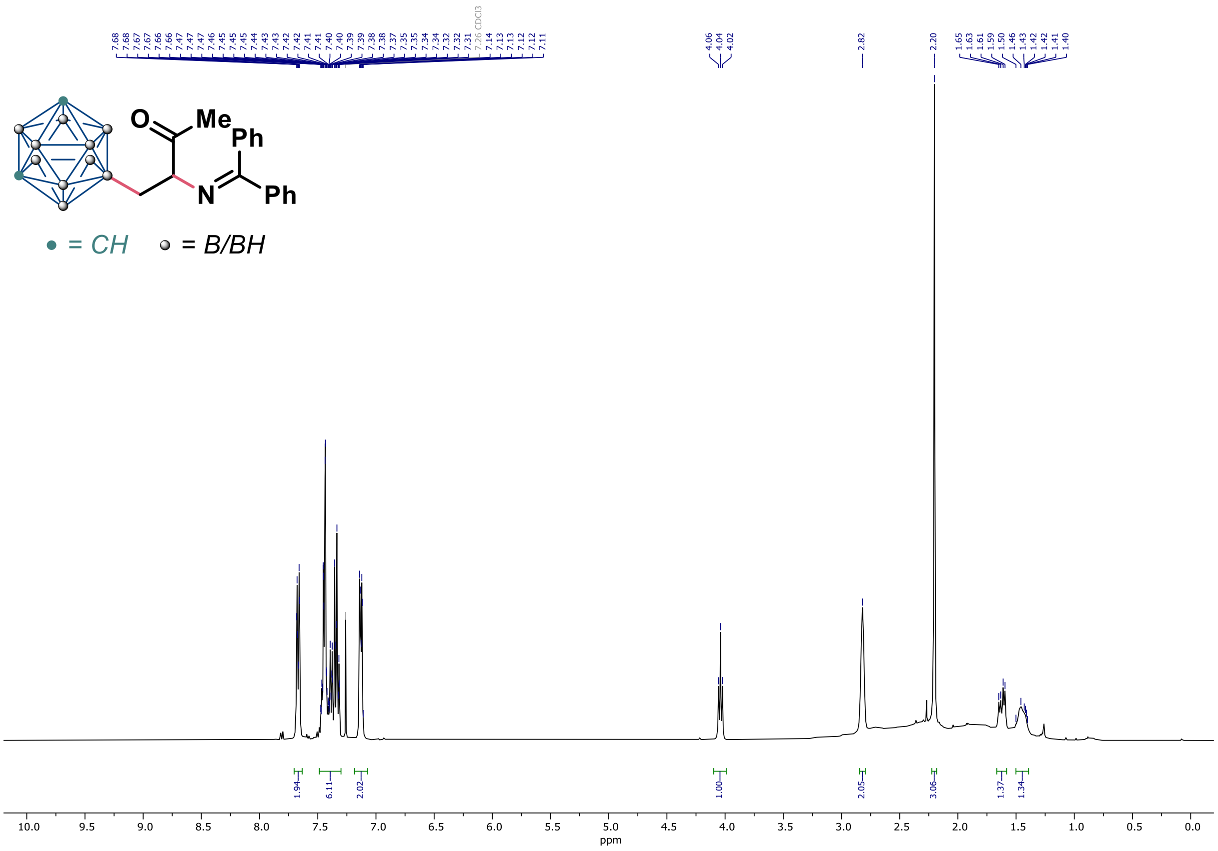

¹³C{¹H} NMR (CDCl₃, 101 MHz) for **5c**

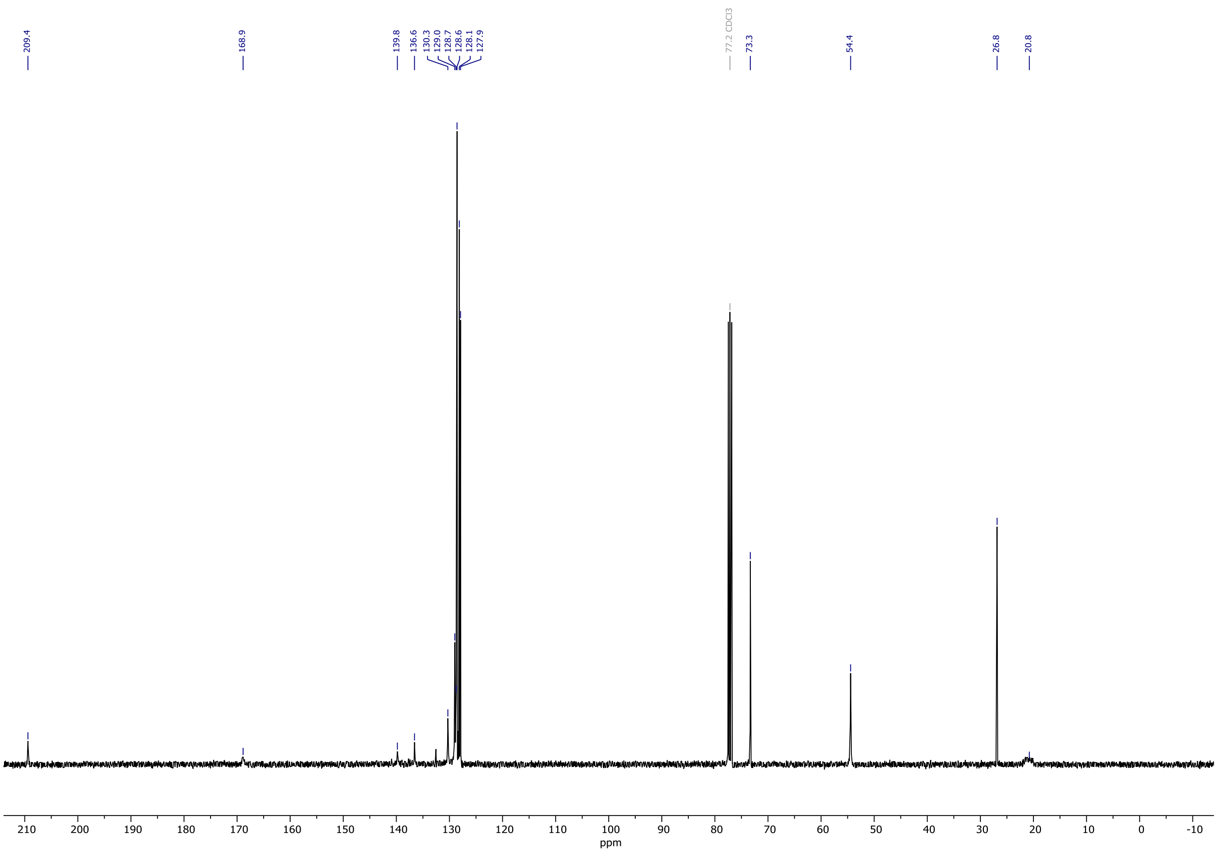

$^{11}B\{^1H\}$ NMR ($CDCl_3$, 128 MHz) for **5c**

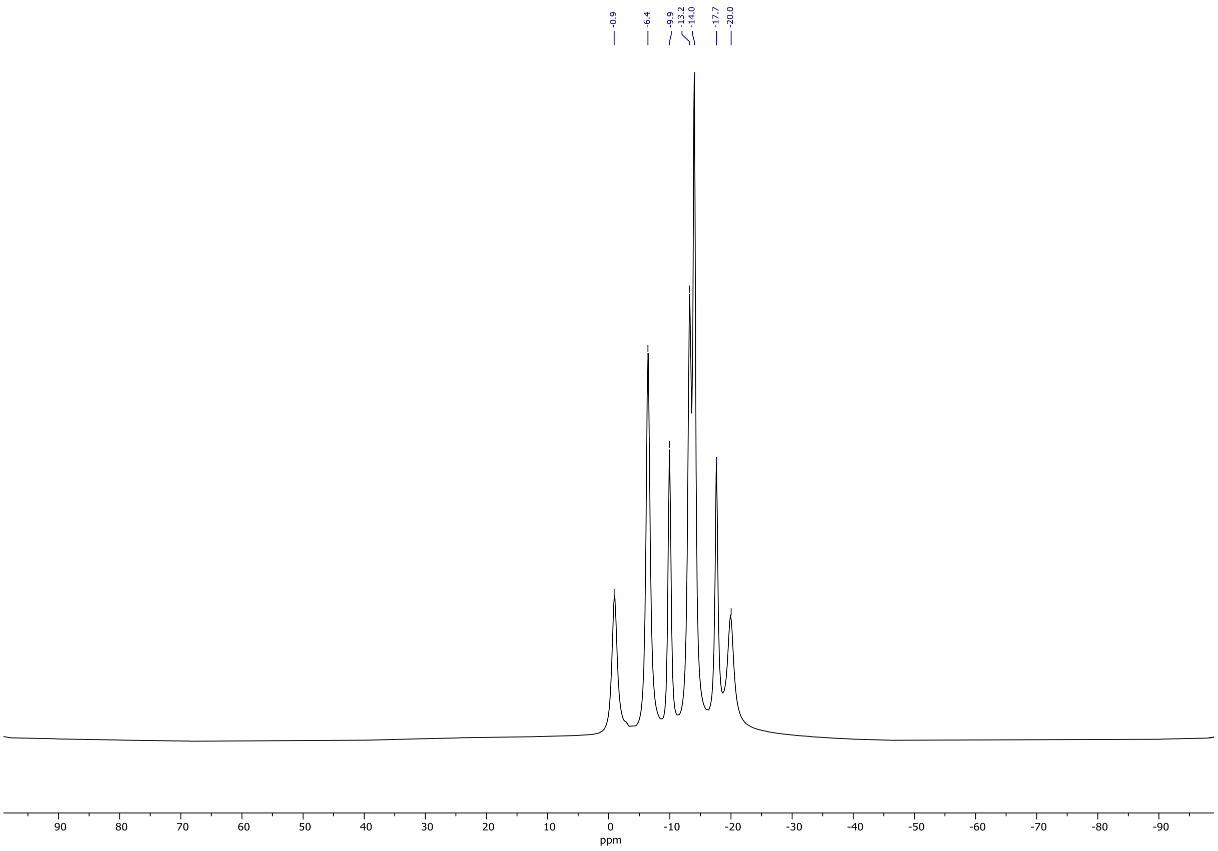

^{1}H NMR ($CDCl_3$, 400 MHz) for **5d**

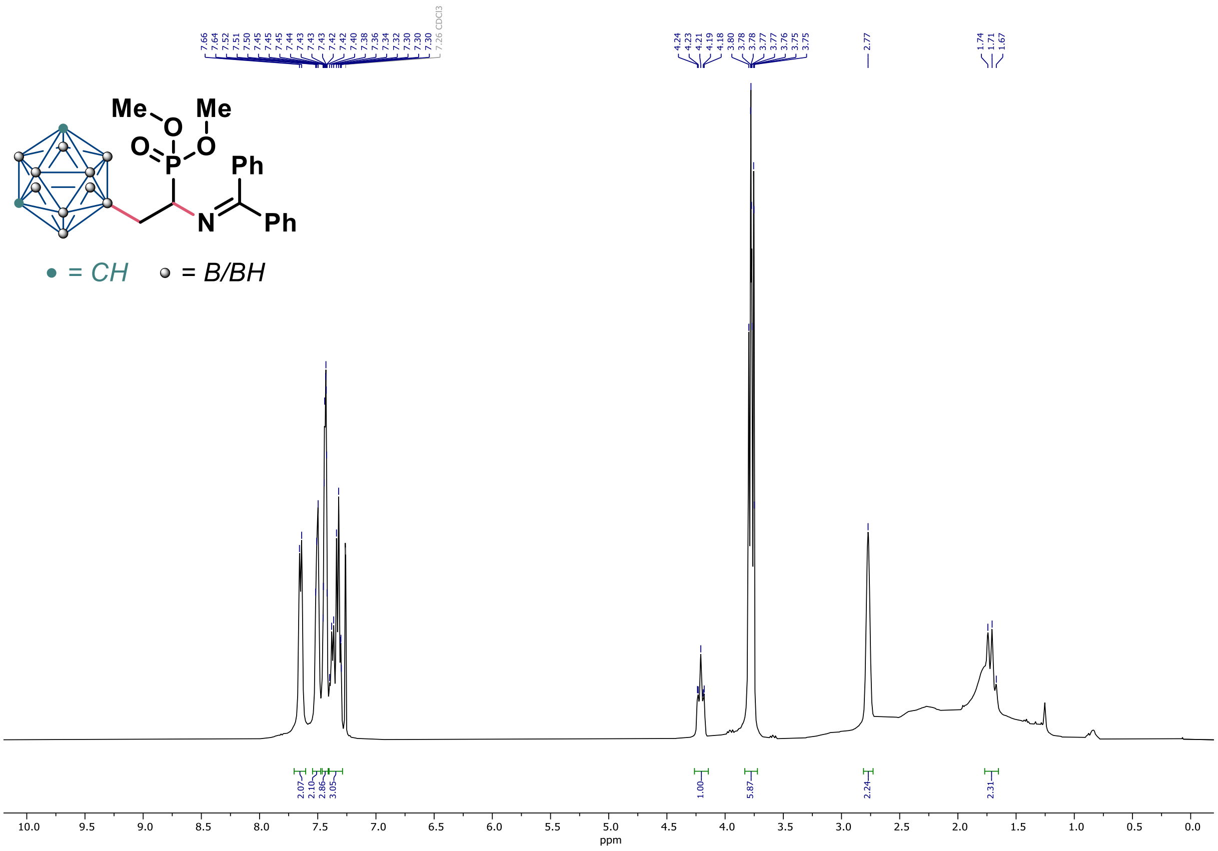

^{13}C{^{1}H} NMR ($CDCl_3$, 101 MHz) for **5d**

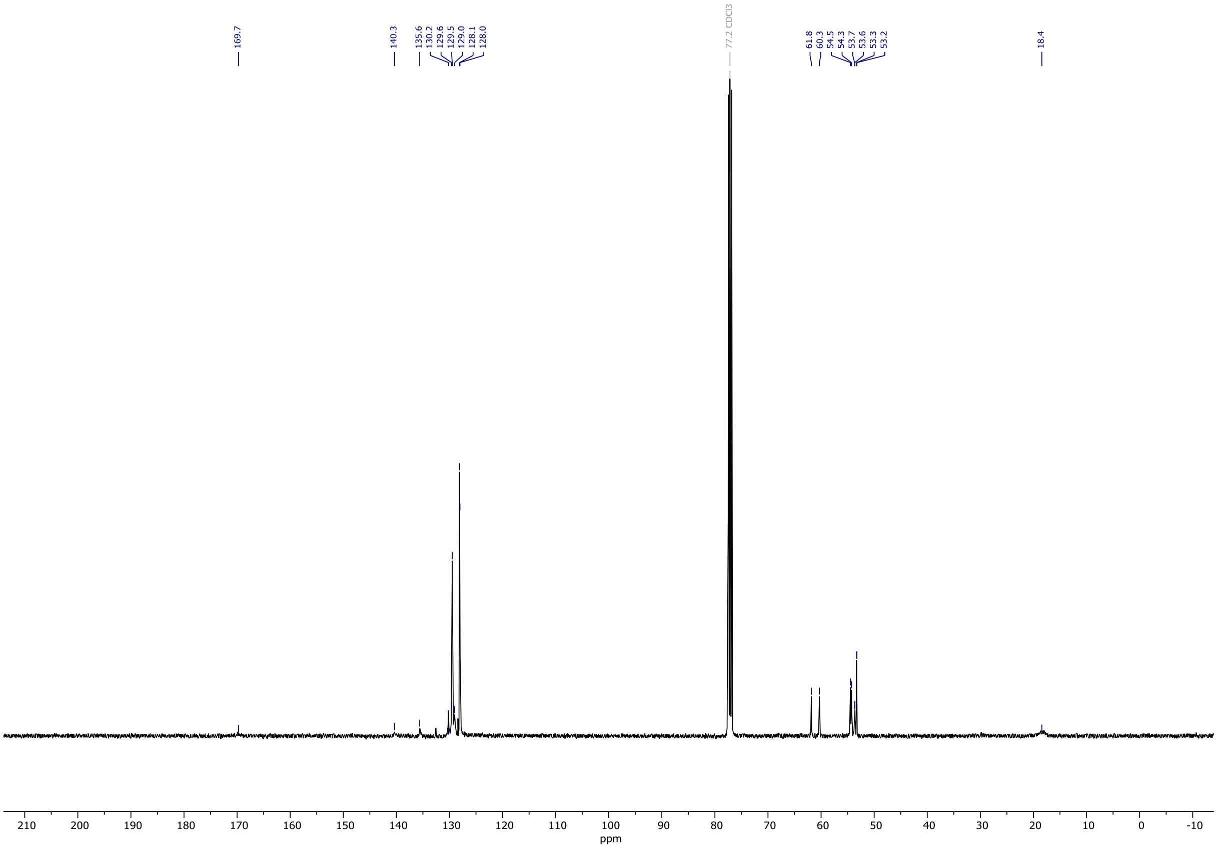

$^{11}B\{^{1}H\}$ NMR (CDCl$_3$, 128 MHz) for **5d**

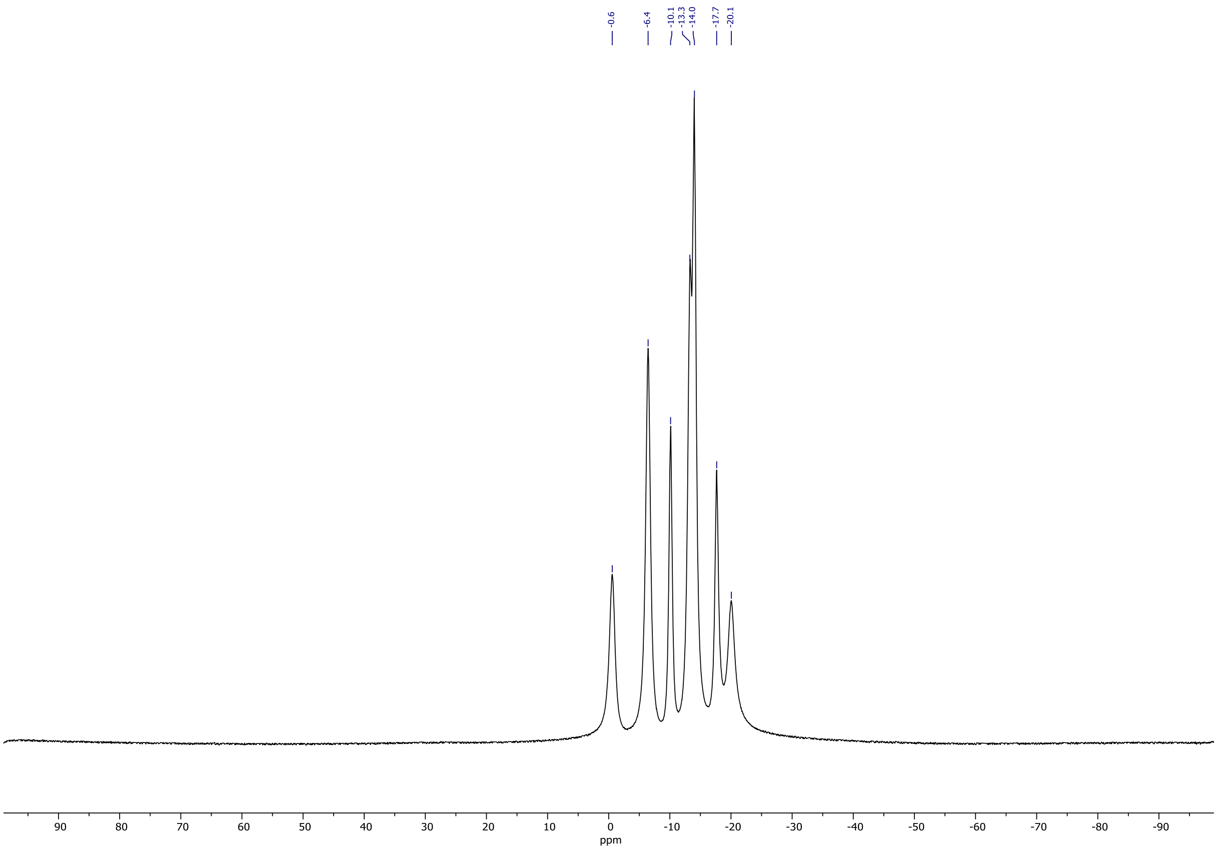

^{31}P NMR (CDCl$_3$, 162 MHz) for **5d**

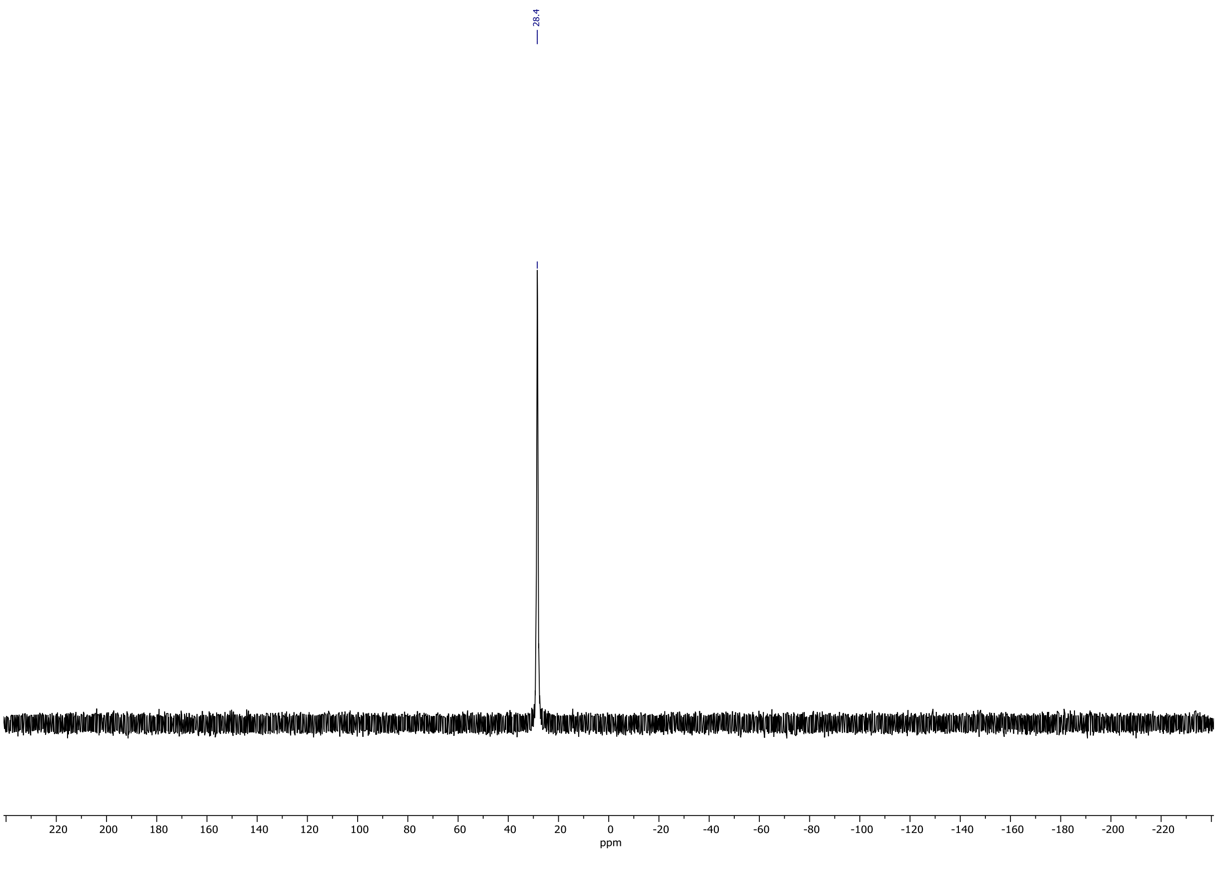

^{1}H NMR (D_2O, 400 MHz) for **5e**

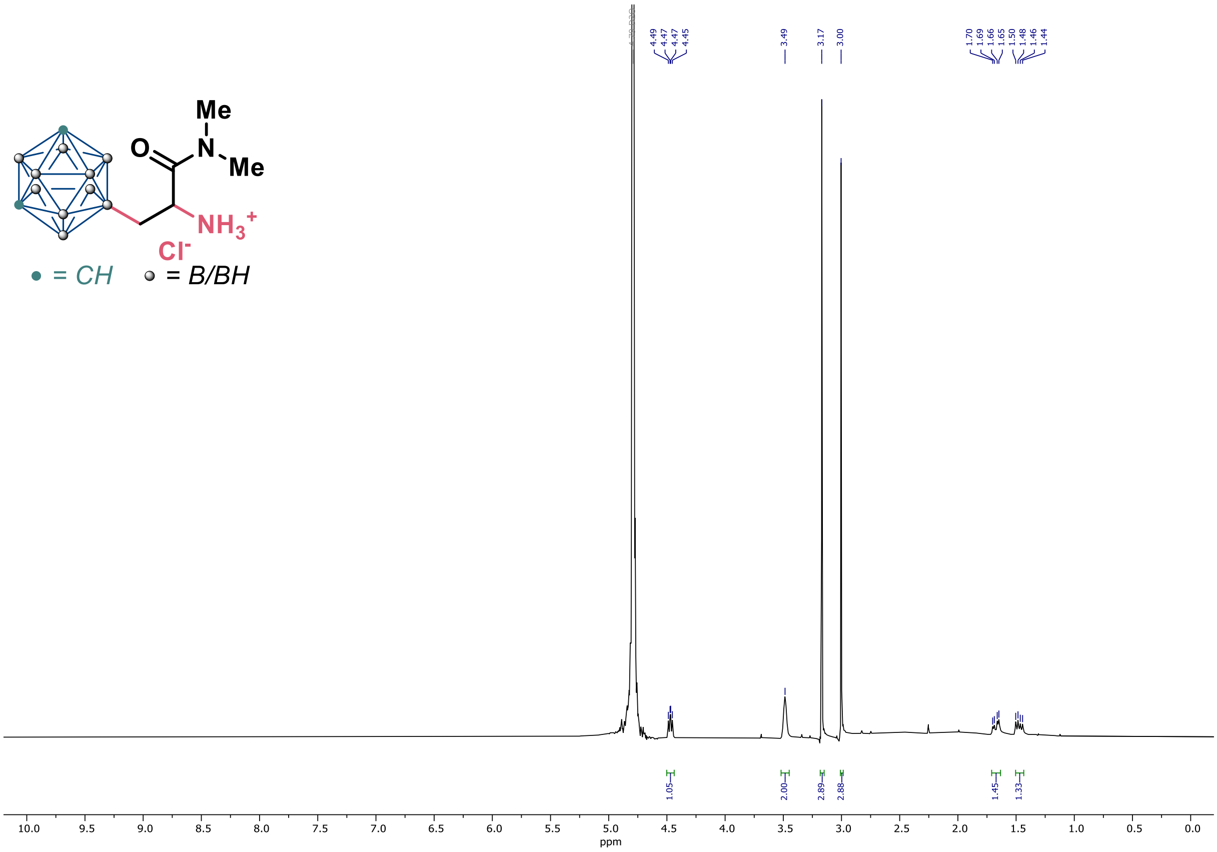

^{13}C{^{1}H} NMR (D_2O, 101 MHz) for **5e**

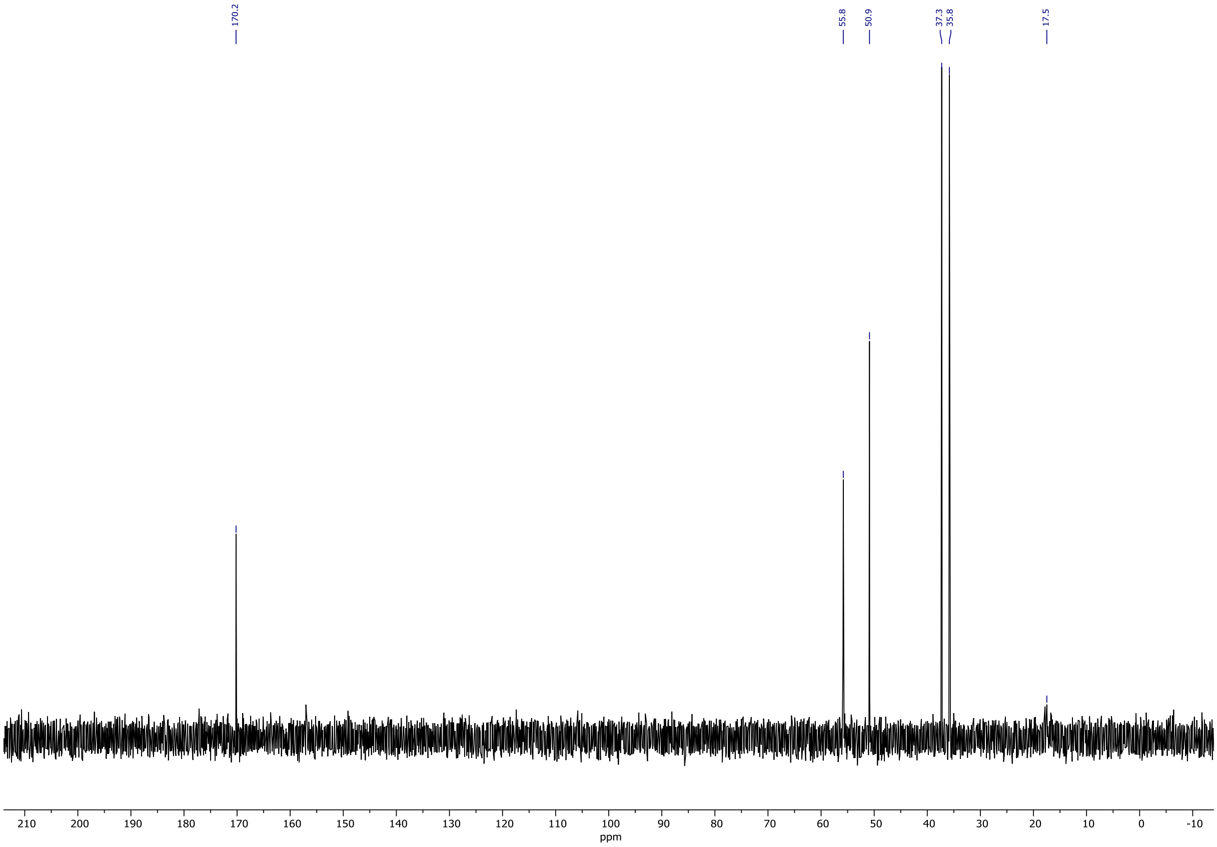

$^{11}B\{^{1}H\}$ NMR (D_2O, 128 MHz) for **5e**

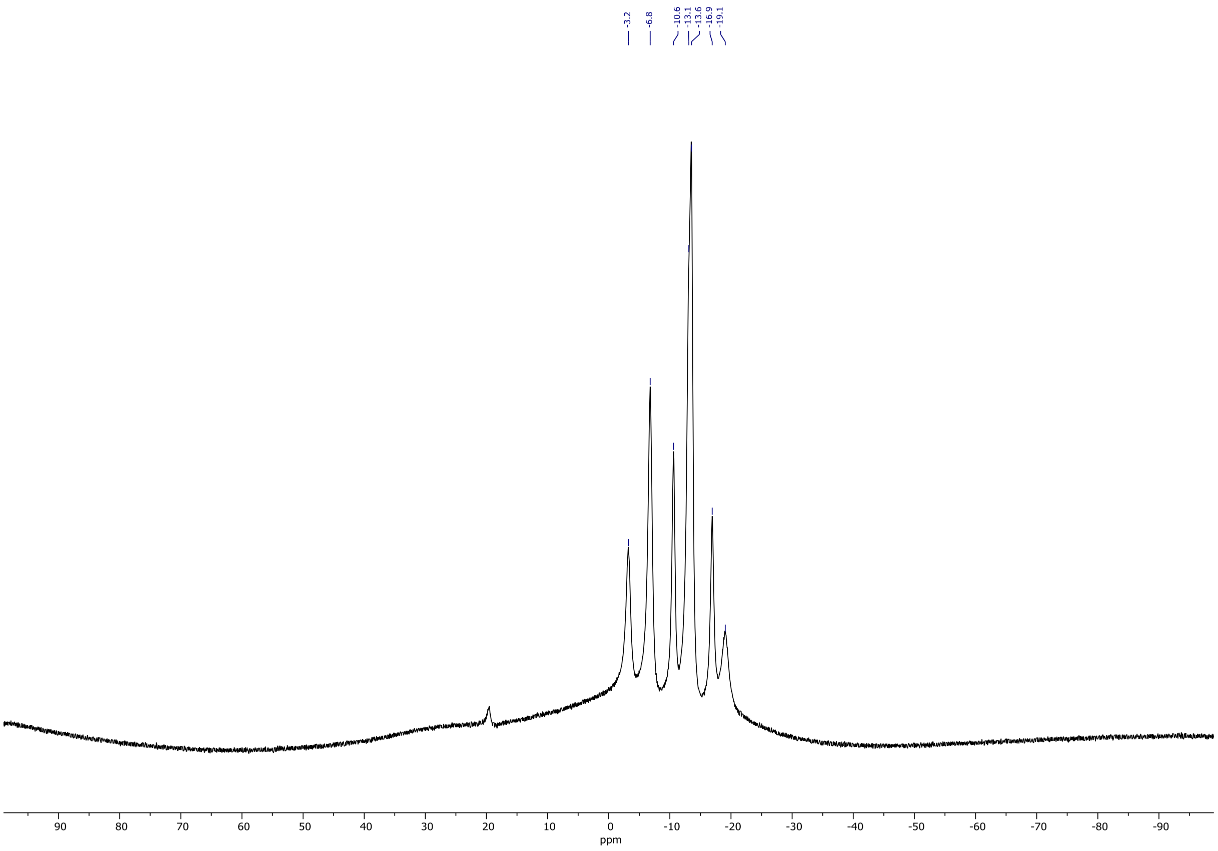

¹H NMR (CDCl$_3$, 400 MHz) for **5f**

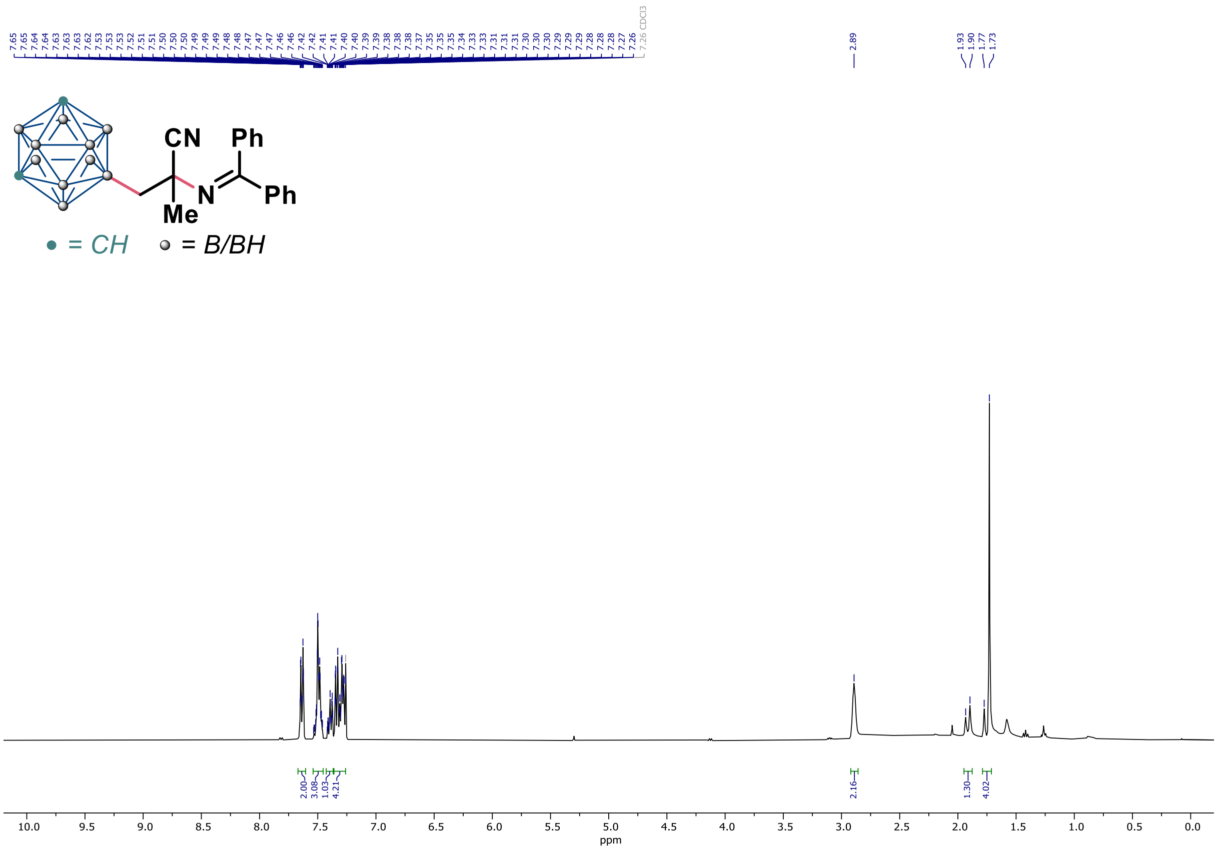

¹³C{¹H} NMR (CDCl$_3$, 101 MHz) for **5f**

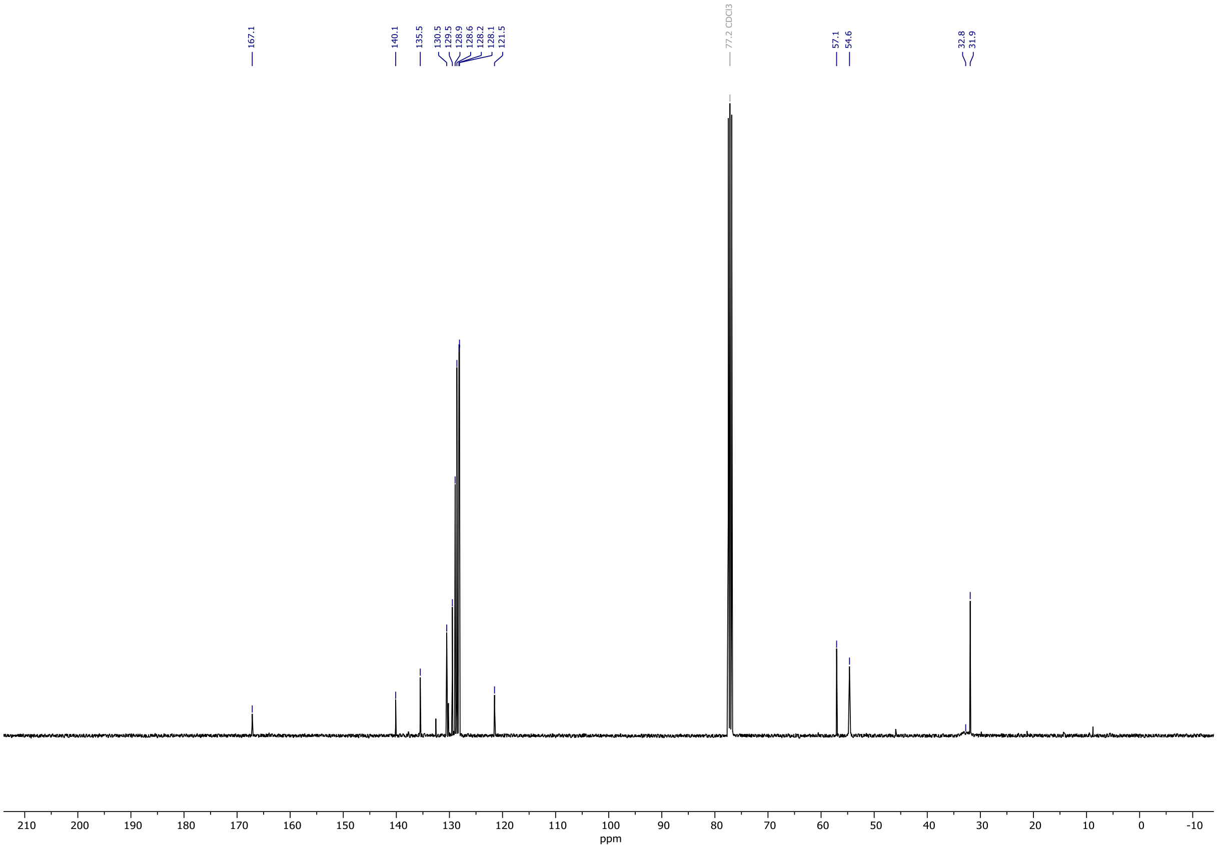

$^{11}B\{^{1}H\}$ NMR ($CDCl_3$, 128 MHz) for **5f**

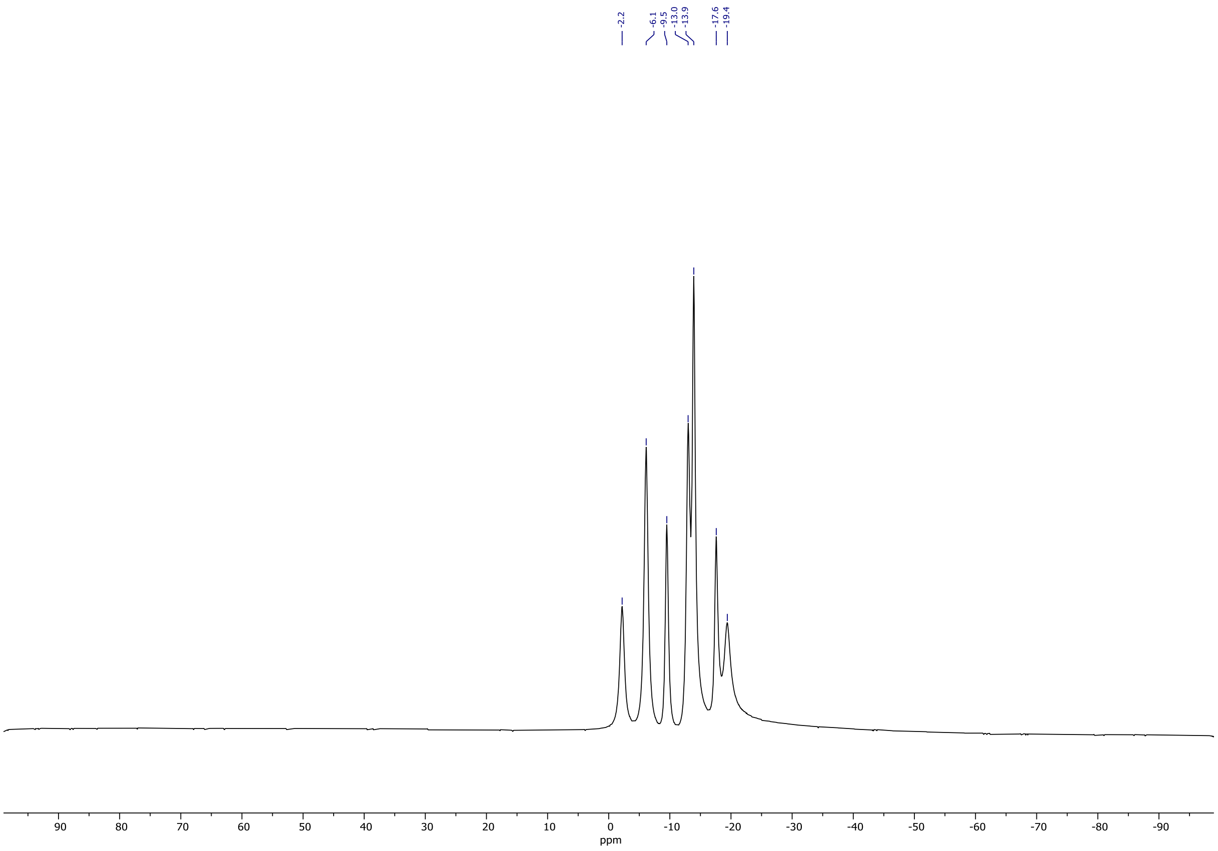

^{1}**H NMR** ($CDCl_3$, 400 MHz) for **5g**

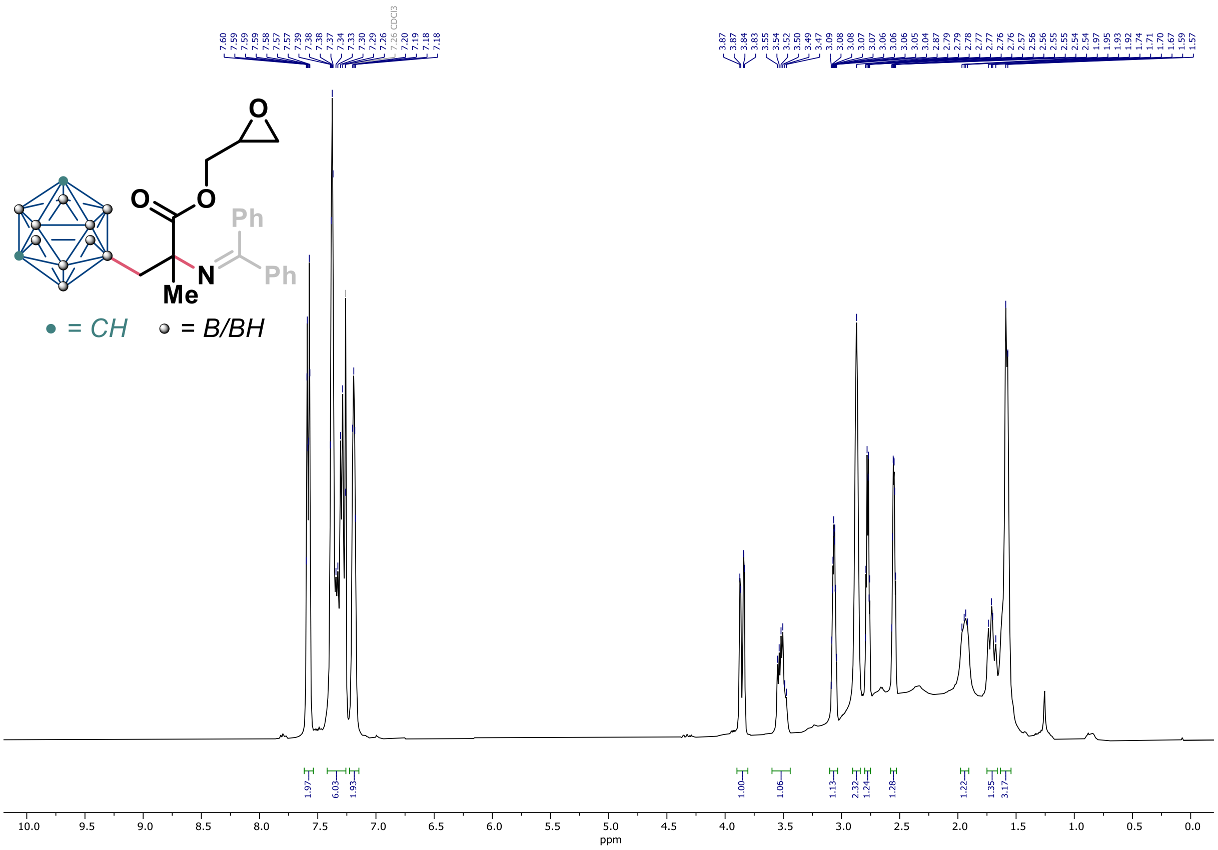

^{13}**C{^{1}H} NMR** ($CDCl_3$, 101 MHz) for **5g**

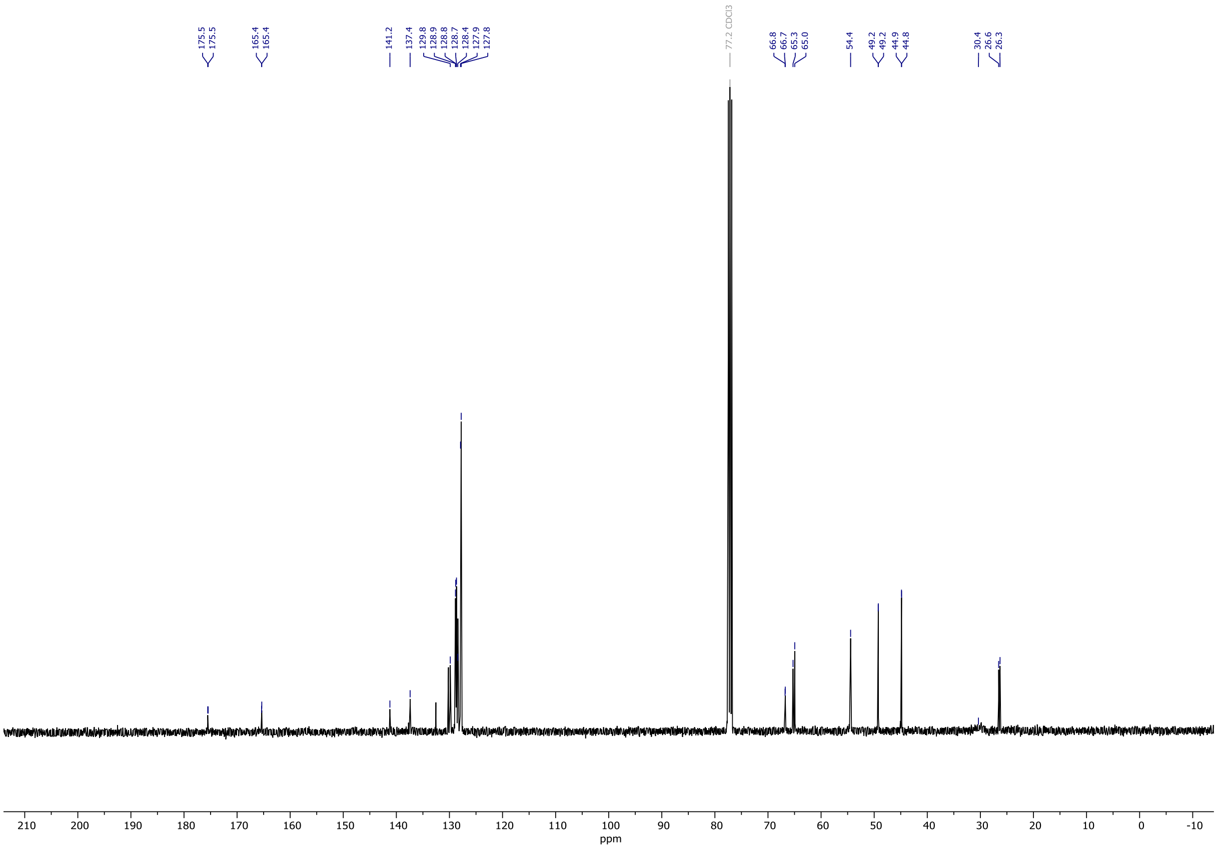

$^{11}B\{^{1}H\}$ NMR ($CDCl_3$, 128 MHz) for **5g**

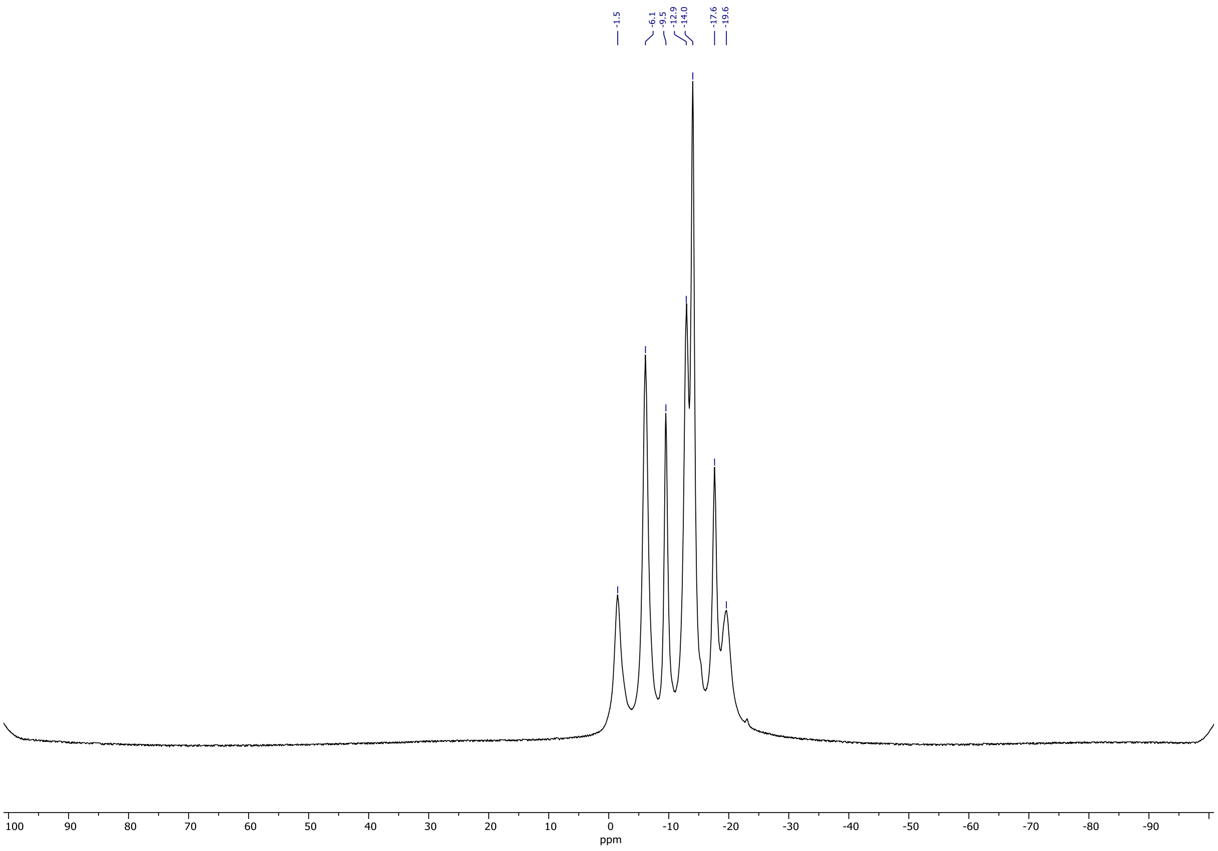

^{1}H NMR ($CDCl_3$, 400 MHz) for **5h**

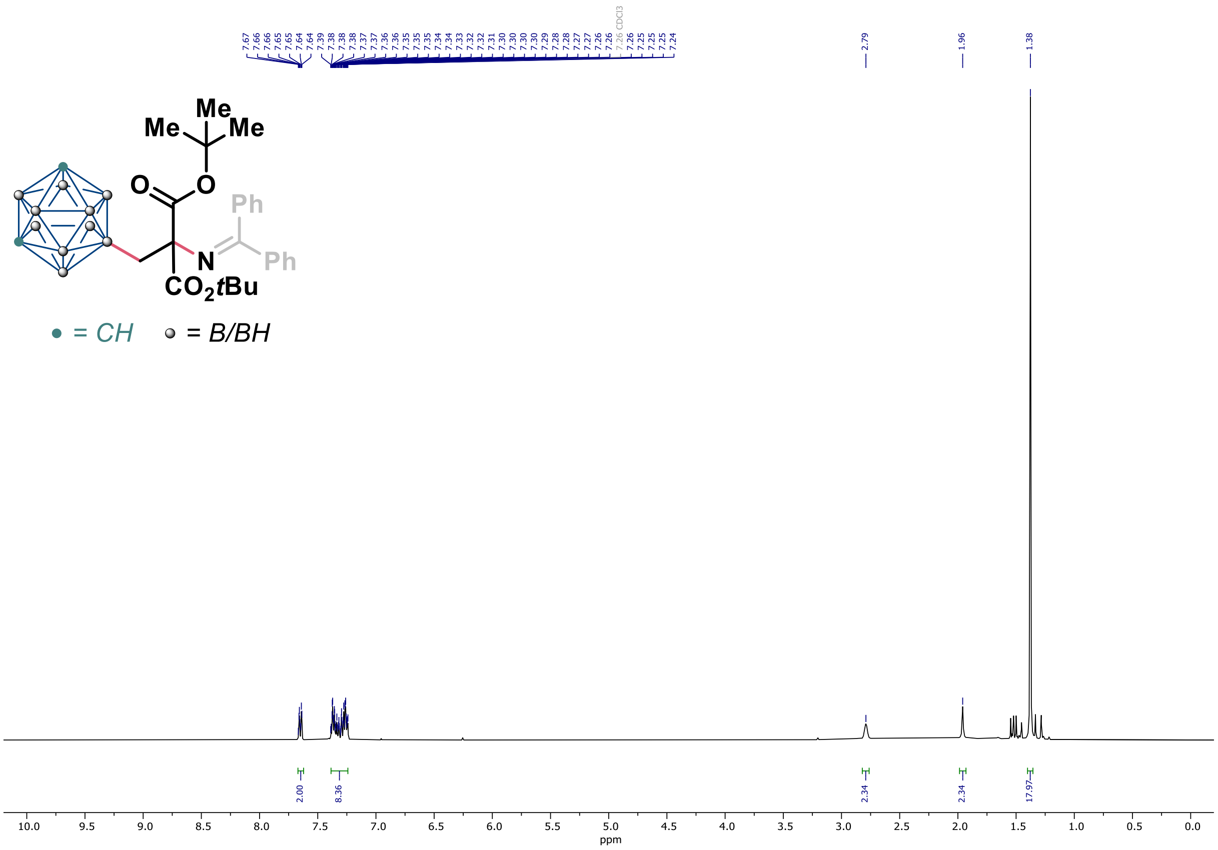

^{13}C{^{1}H} NMR ($CDCl_3$, 101 MHz) for **5h**

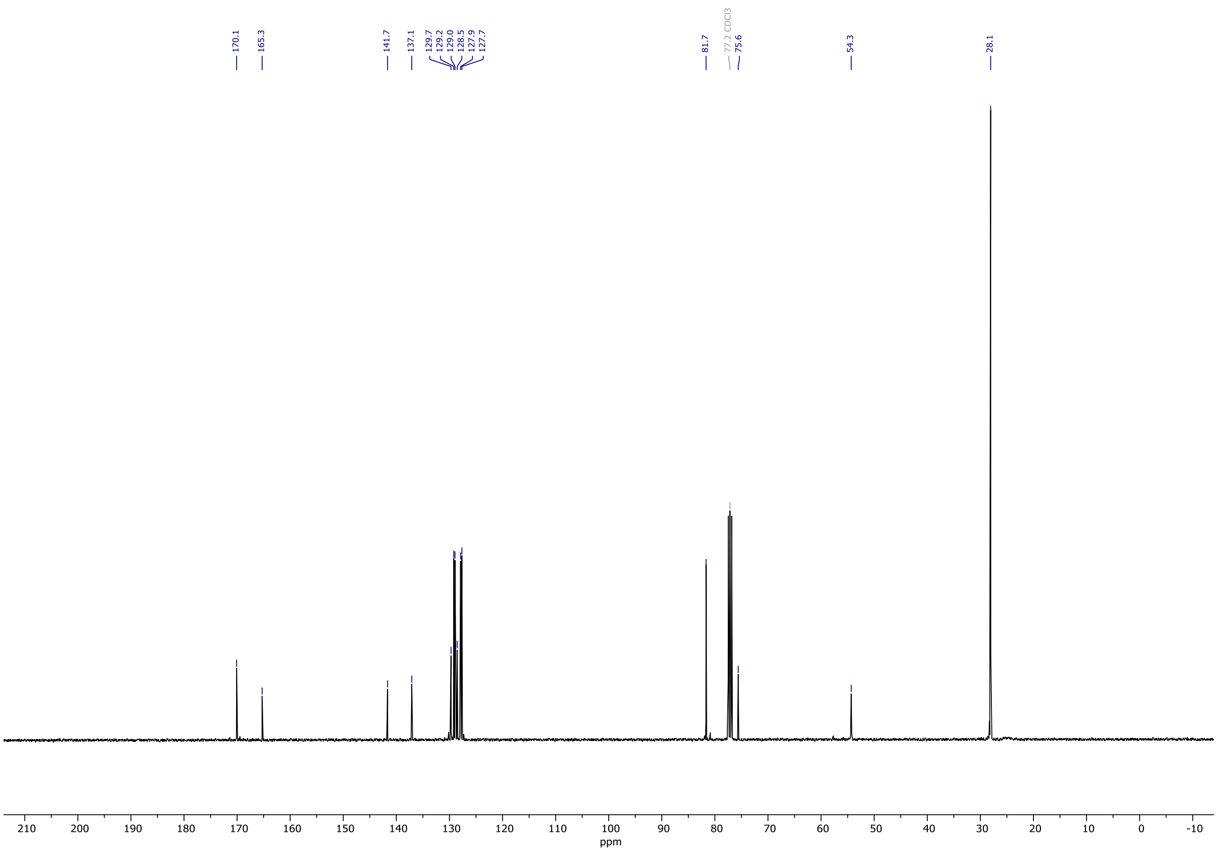

^{11}B{^{1}H} NMR (CDCl$_3$, 128 MHz) for **5h**

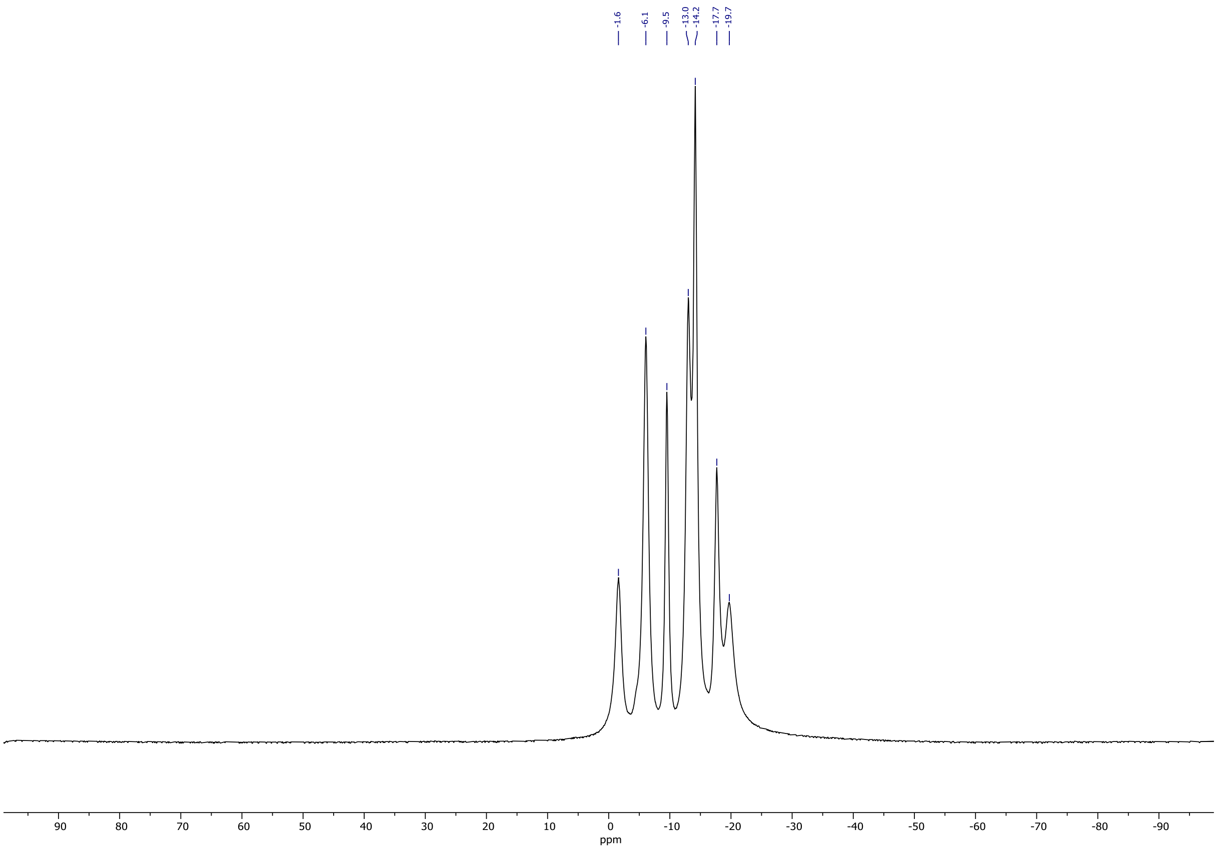

^{1}H NMR ($CDCl_3$, 400 MHz) for **5i (major regioisomer)**

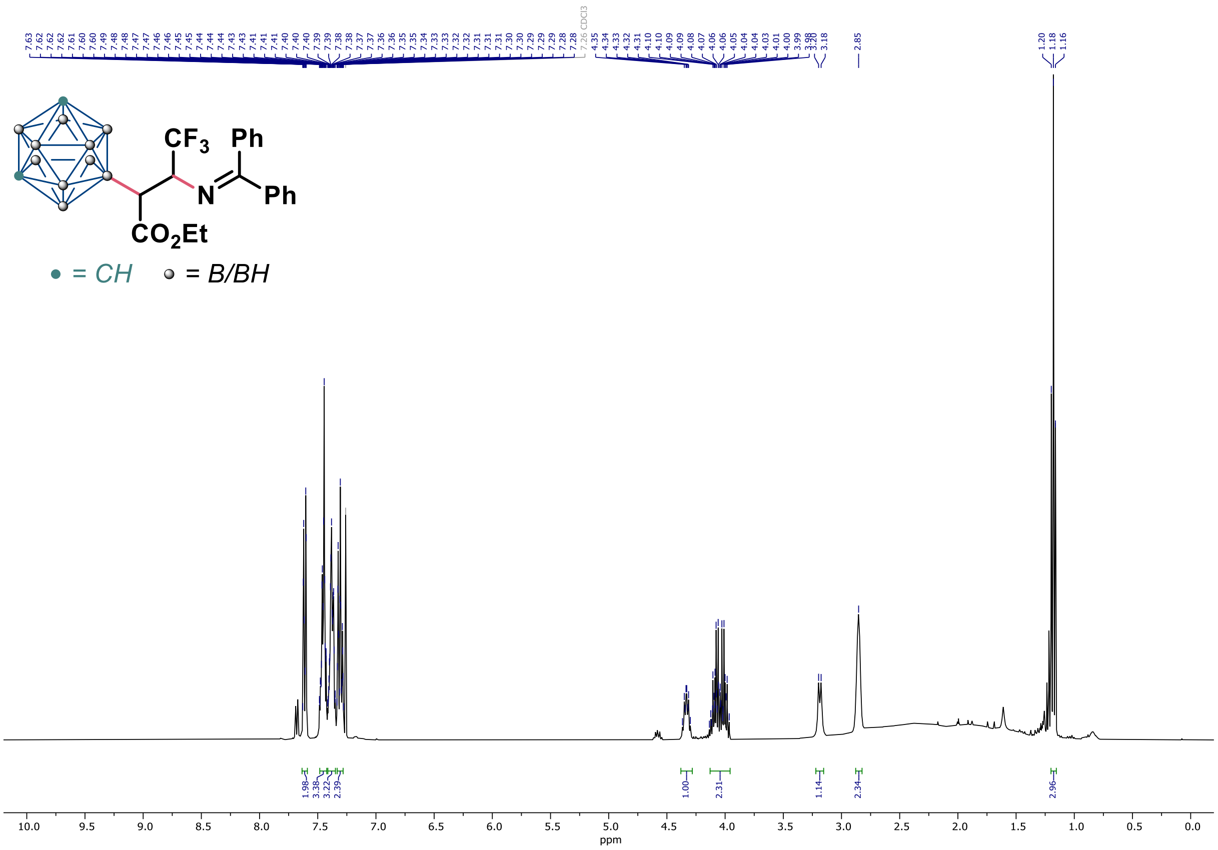

^{13}C{^{1}H} NMR ($CDCl_3$, 101 MHz) for **5i (major regioisomer)**

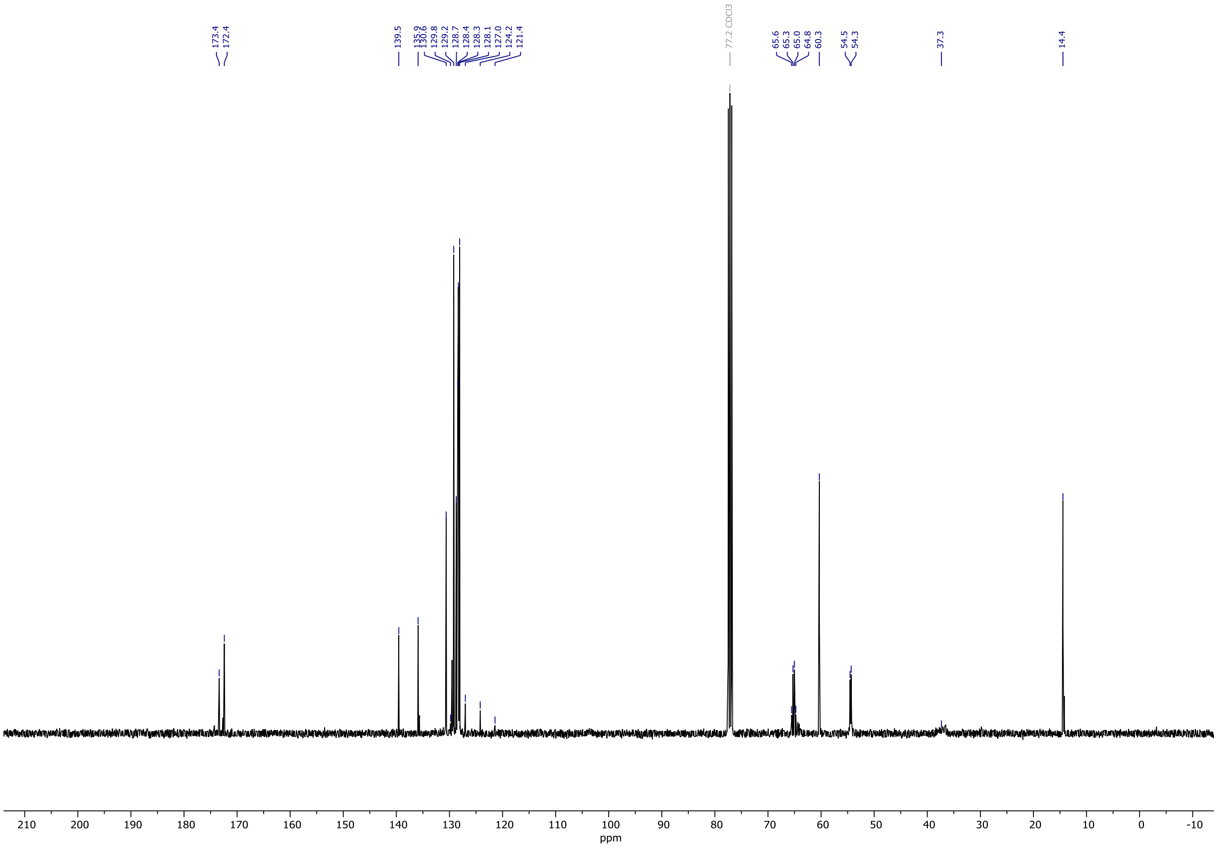

$^{11}B\{^1H\}$ NMR ($CDCl_3$, 128 MHz) for **5i (major regioisomer)**

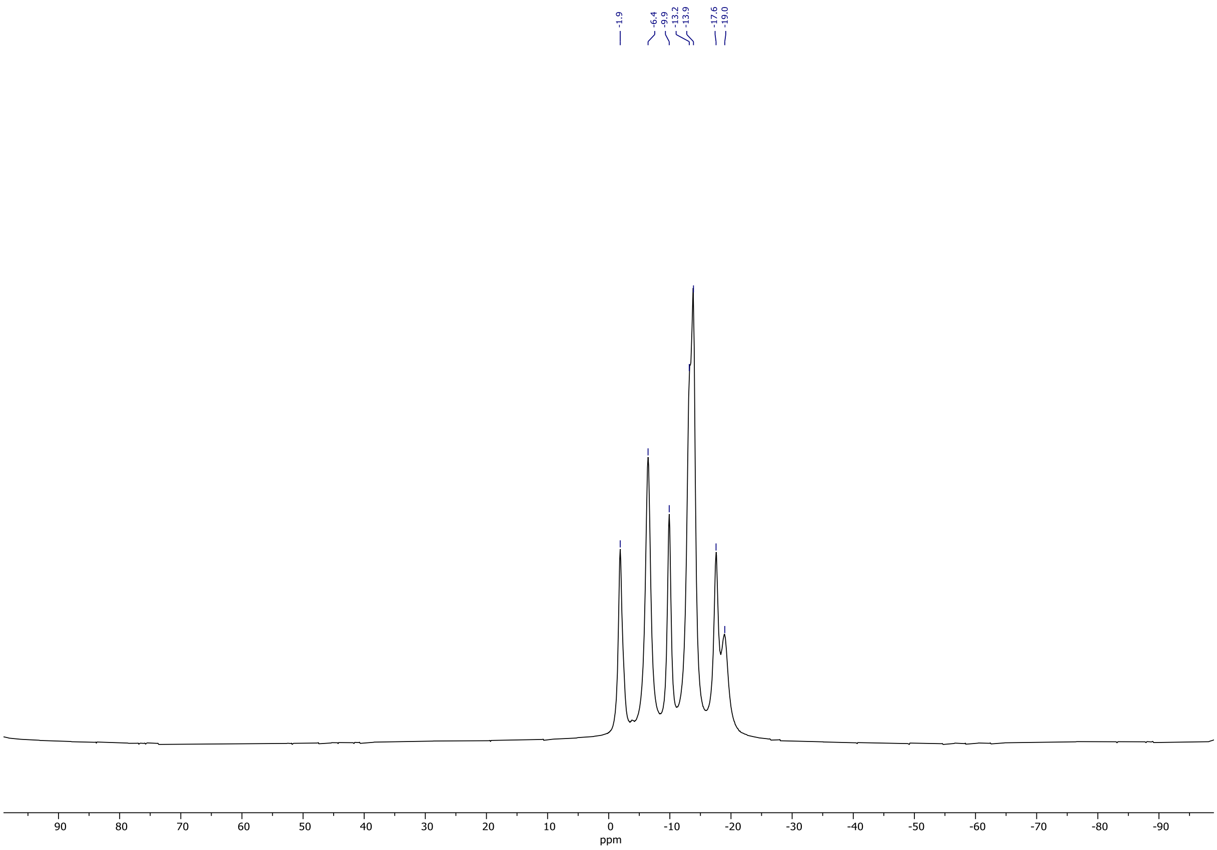

$^{19}F\{^1H\}$ NMR ($CDCl_3$, 376 MHz) for **5i (major regioisomer)**

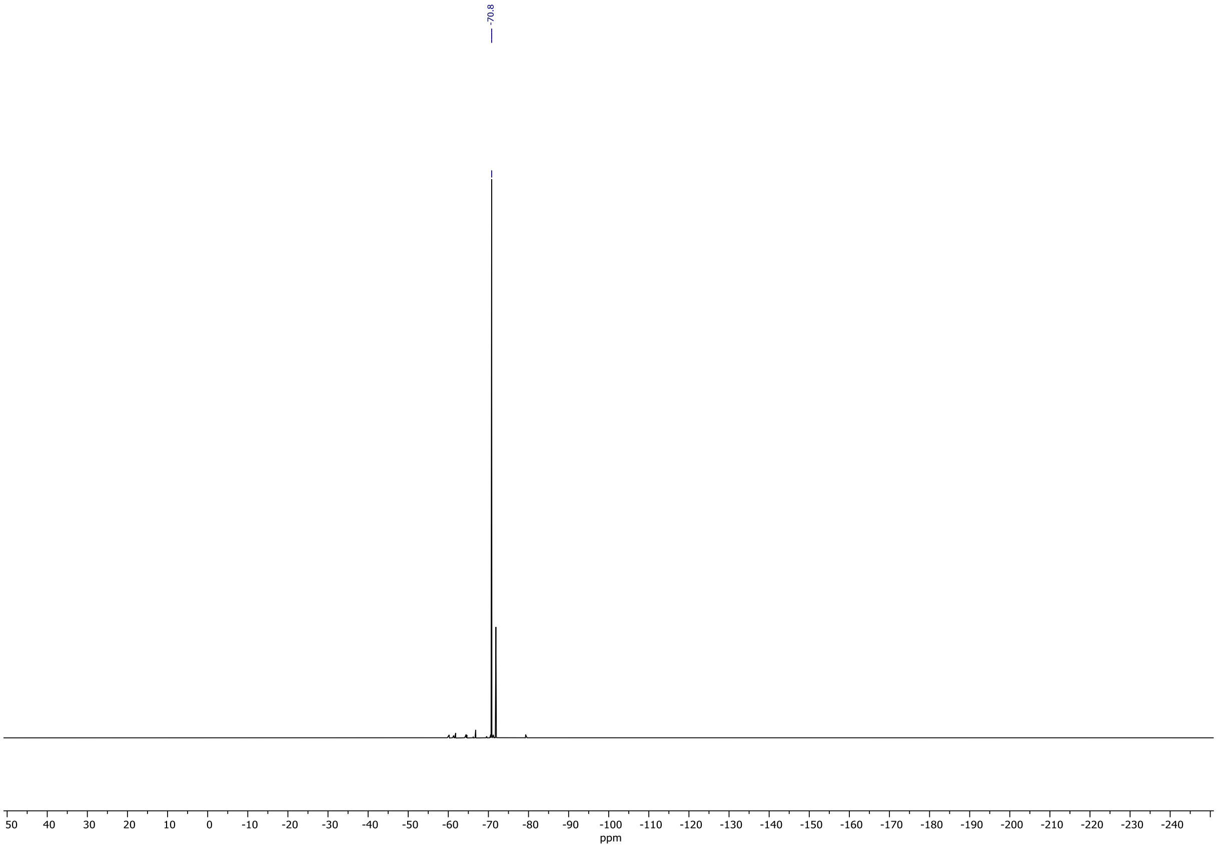

^{1}H NMR ($CDCl_3$, 400 MHz) for **5i (minor regioisomer)**

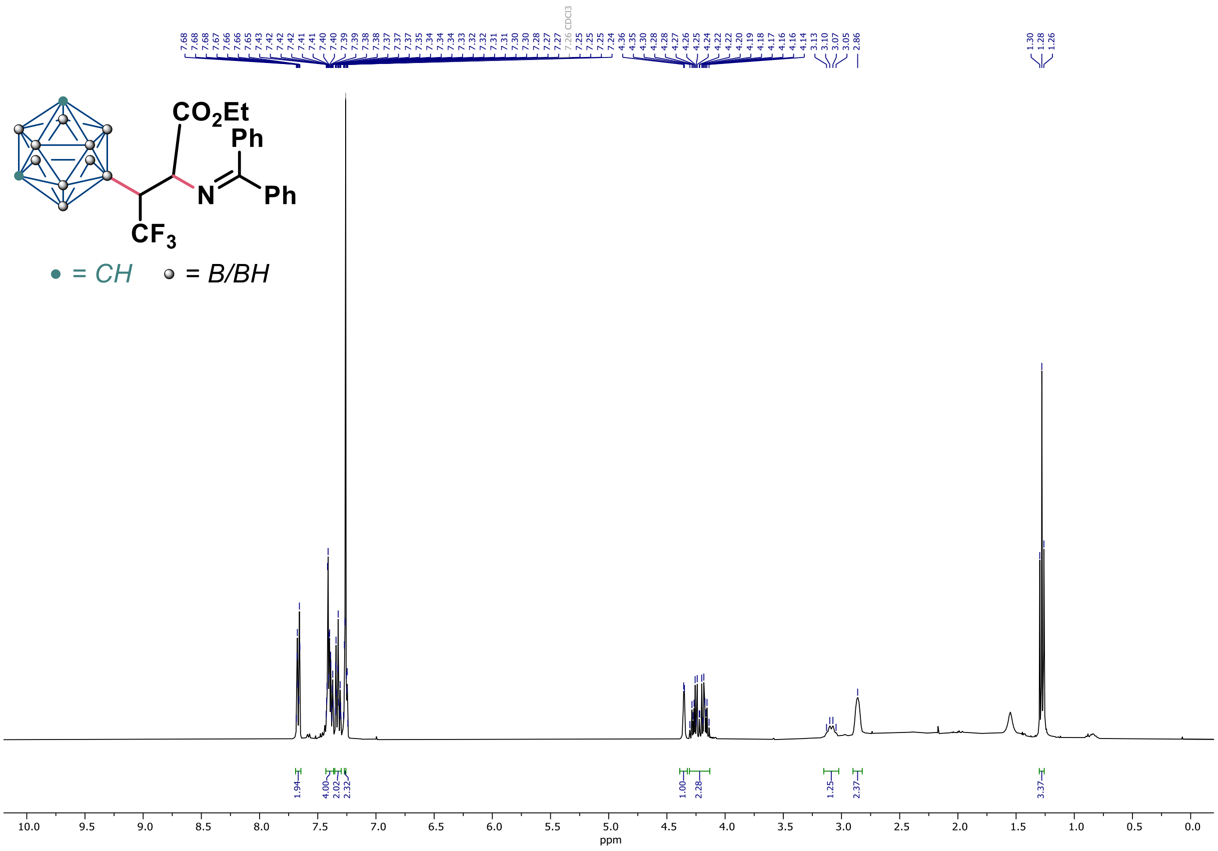

^{13}C{^{1}H} NMR ($CDCl_3$, 101 MHz) for **5i (minor regioisomer)**

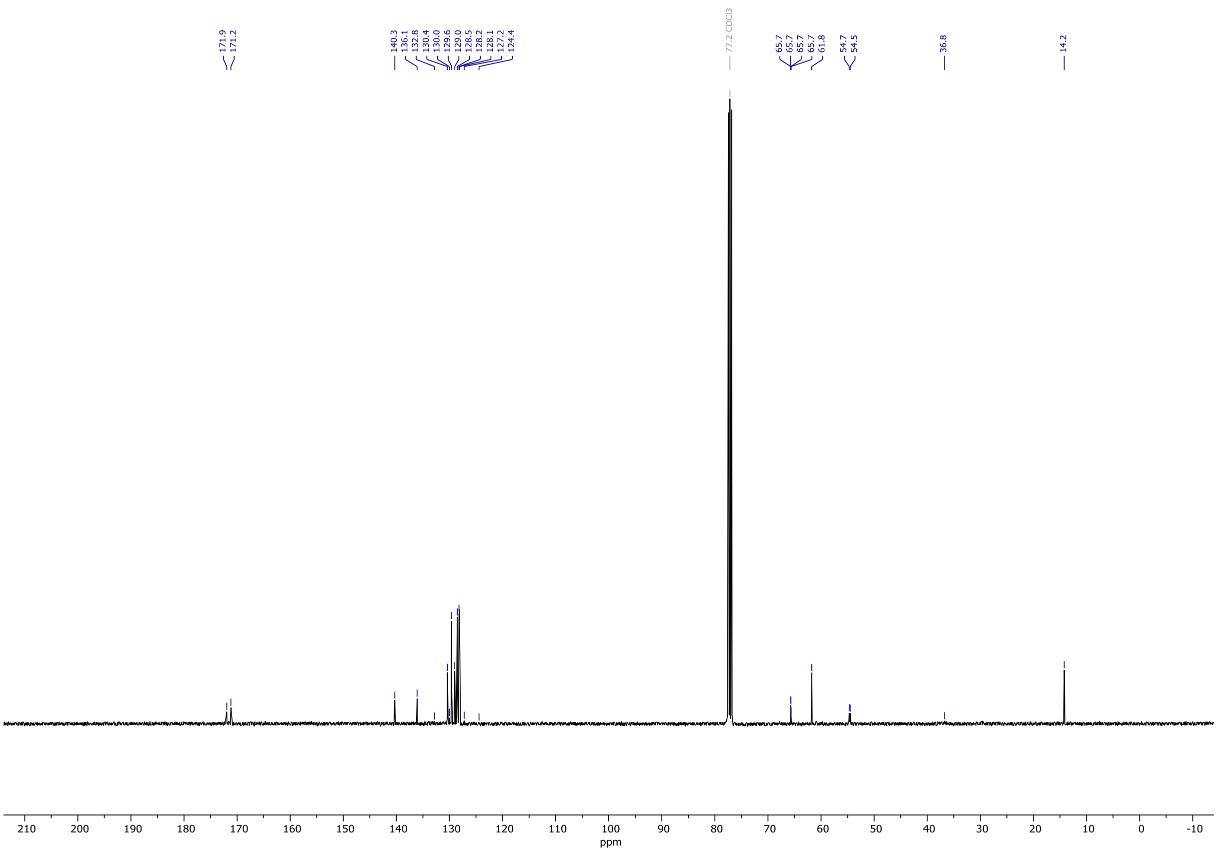

$^{11}B\{^{1}H\}$ NMR (CDCl$_3$, 128 MHz) for **5i (minor regioisomer)**

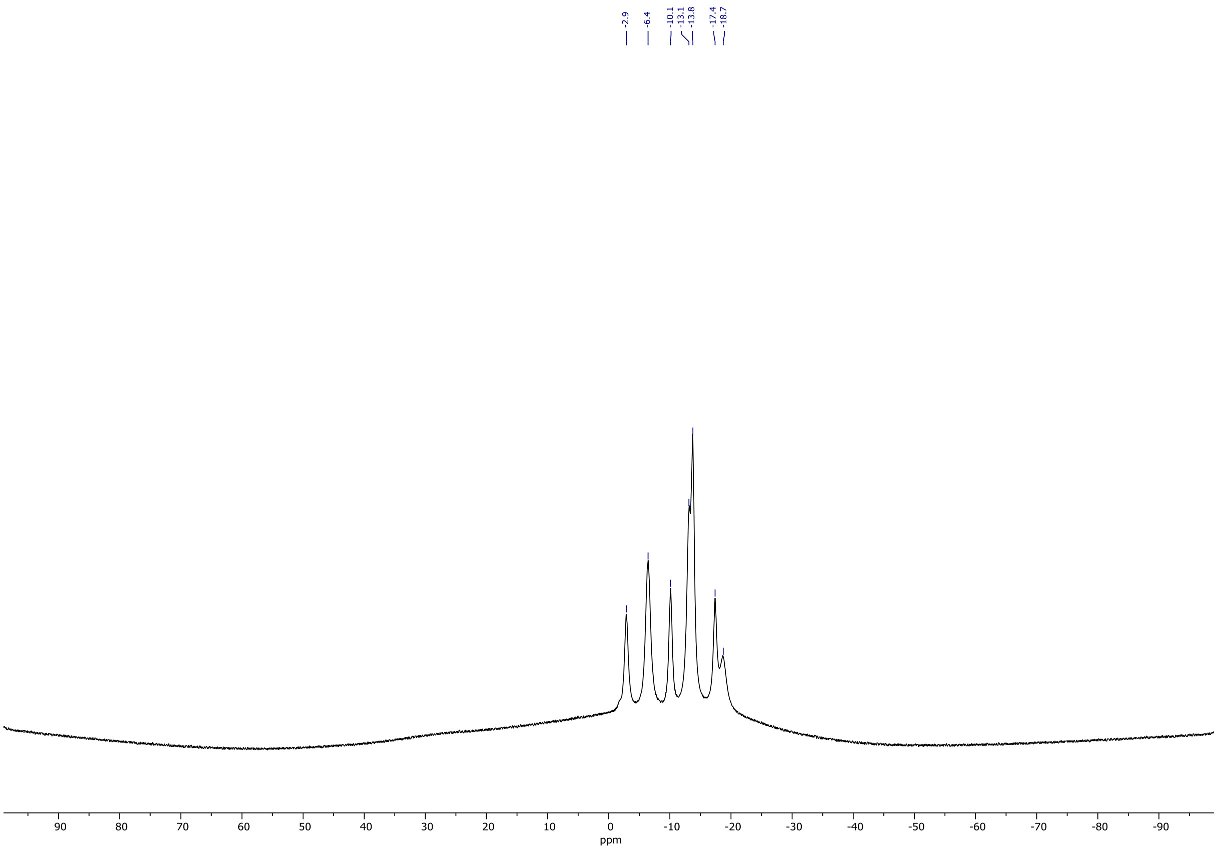

$^{19}F\{^{1}H\}$ NMR (CDCl$_3$, 376 MHz) for **5i (minor regioisomer)**

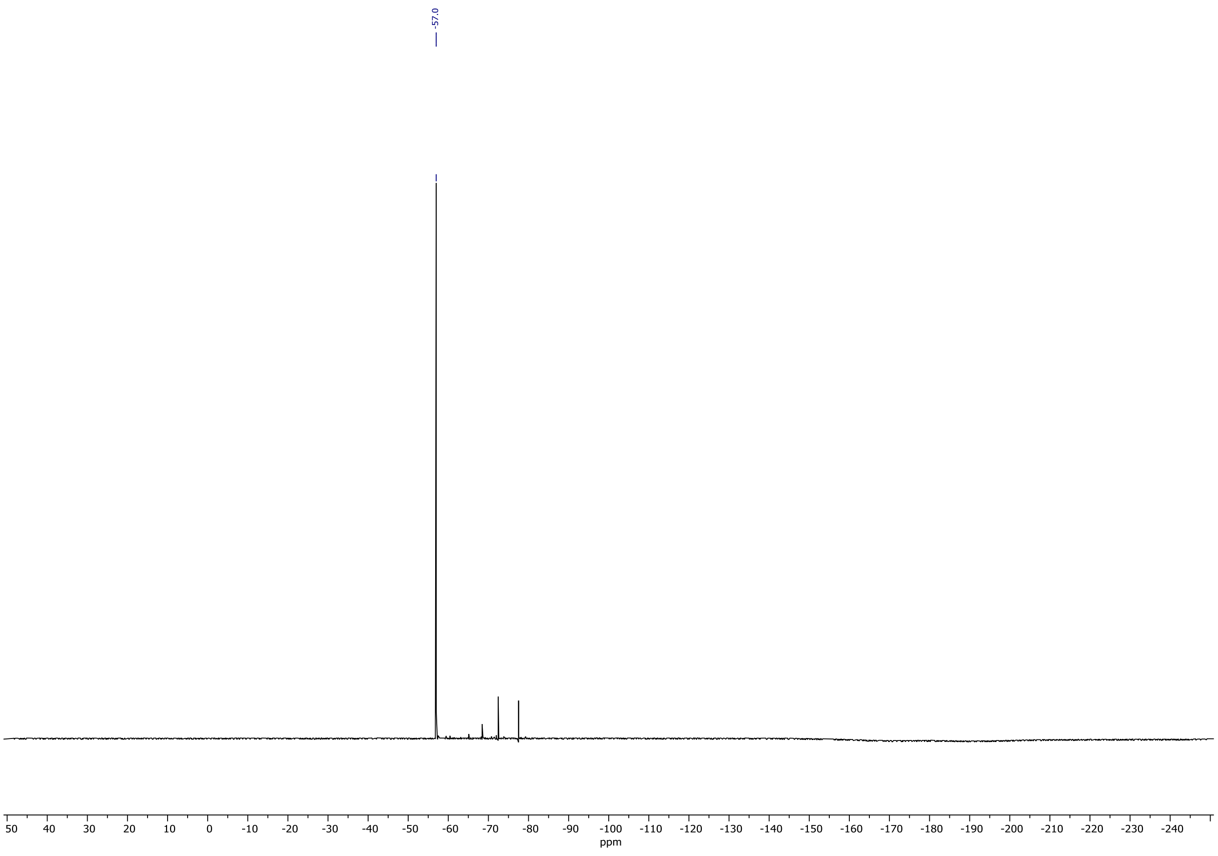

¹H NMR ($CDCl_3$, 400 MHz) for **5j**

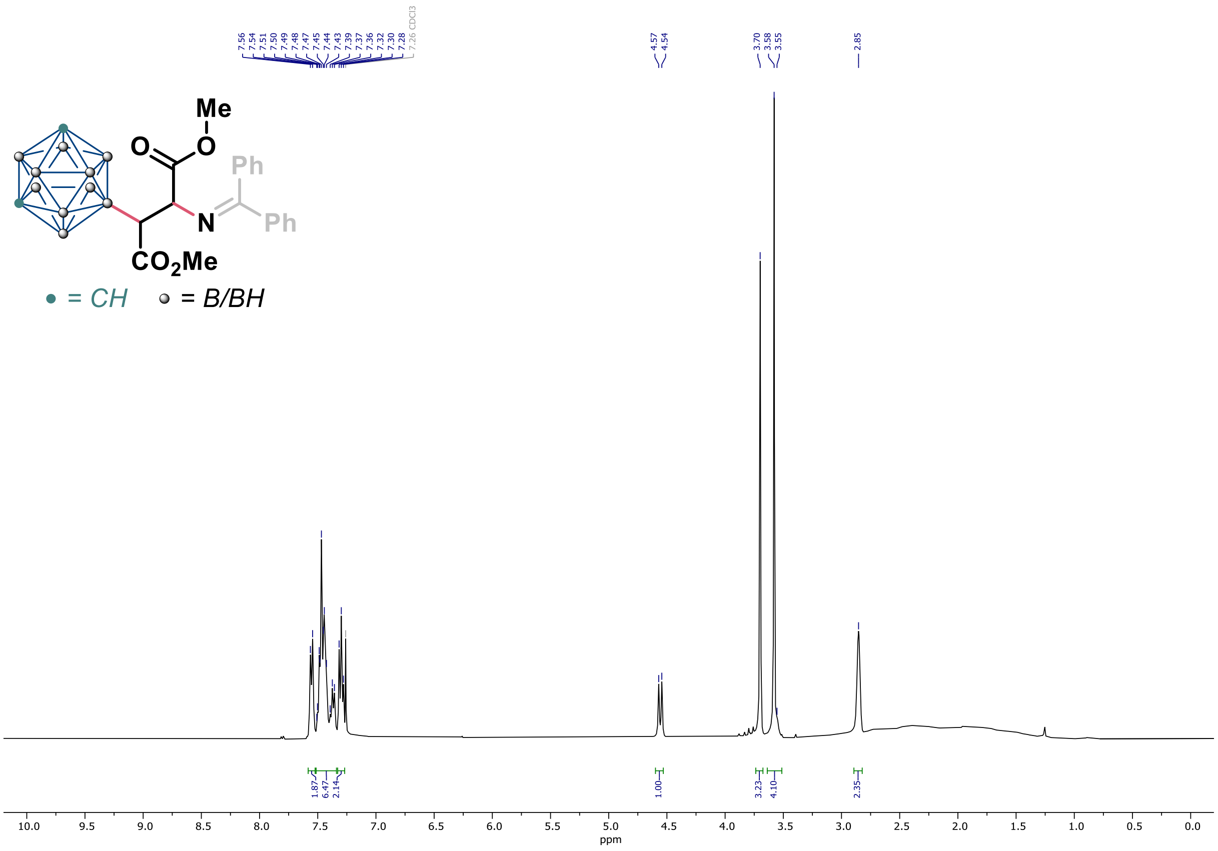

¹³C{¹H} NMR ($CDCl_3$, 101 MHz) for **5j**

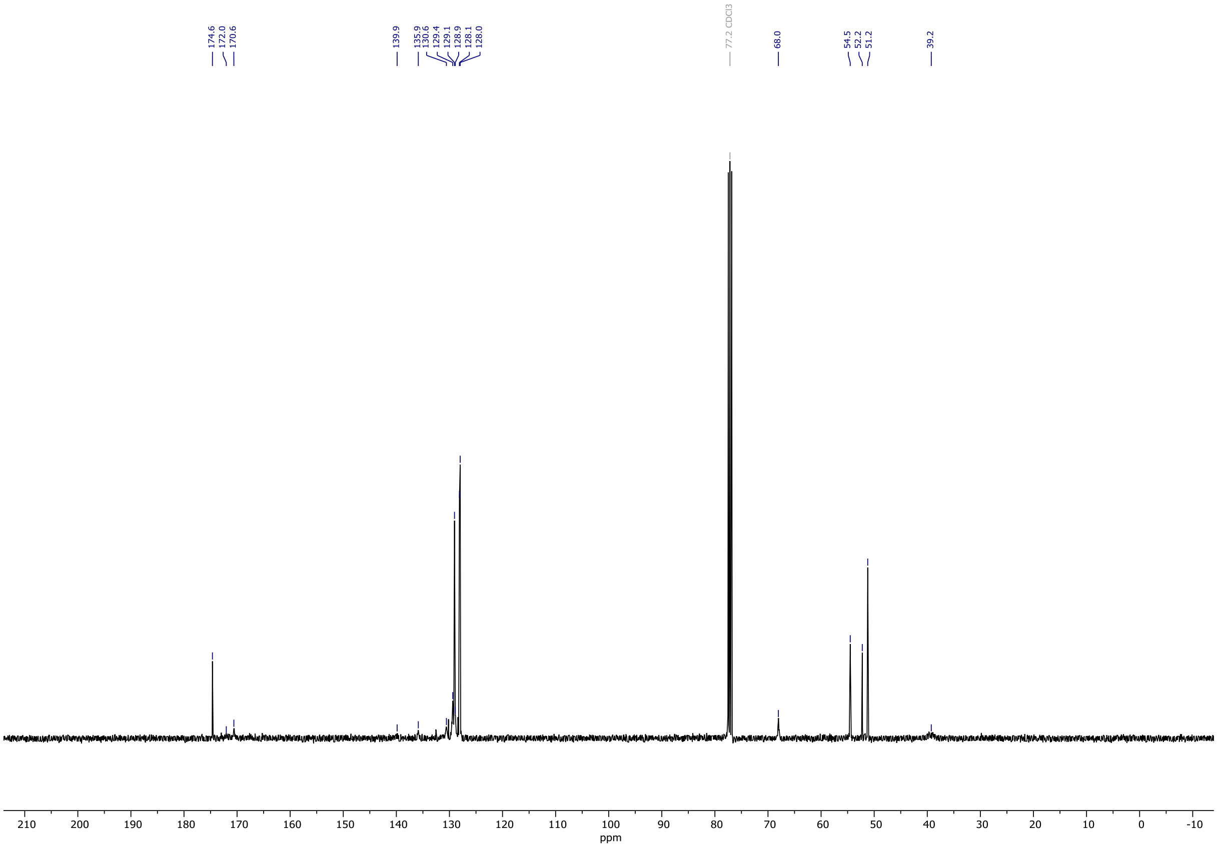

^{11}B{^{1}H} NMR (CDCl$_3$, 128 MHz) for **5j**

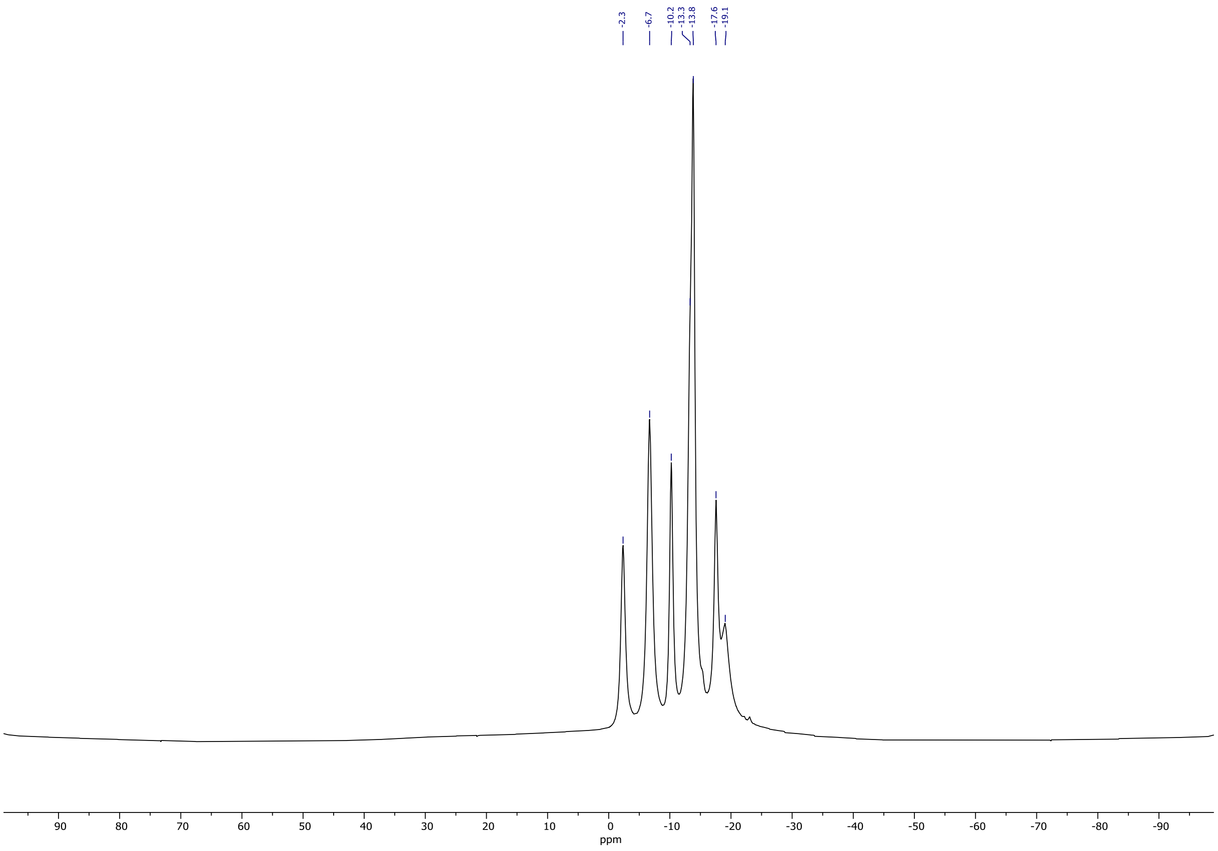

^{1}H NMR ($CDCl_3$, 400 MHz) for **5k**

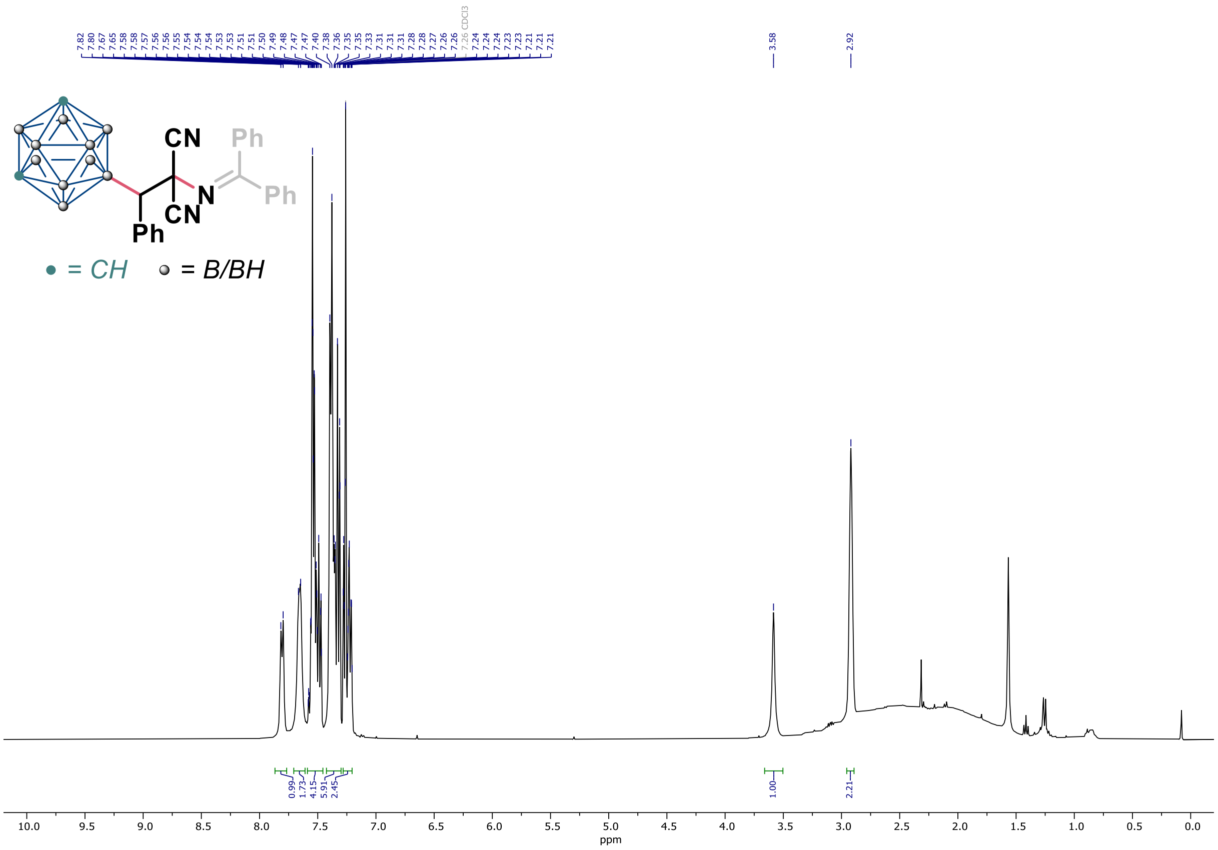

^{13}C{^{1}H} NMR ($CDCl_3$, 101 MHz) for **5k**

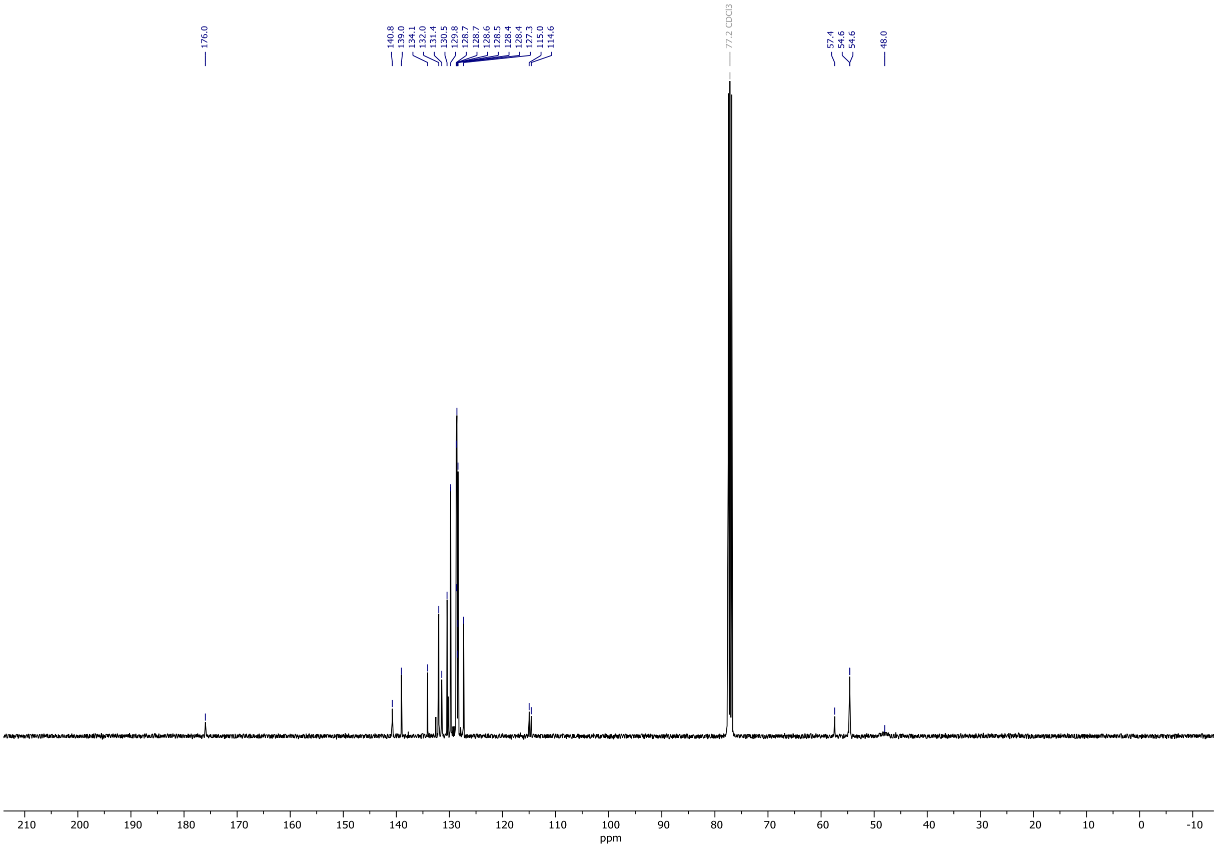

^{11}B{^{1}H} NMR (CDCl$_3$, 128 MHz) for **5k**

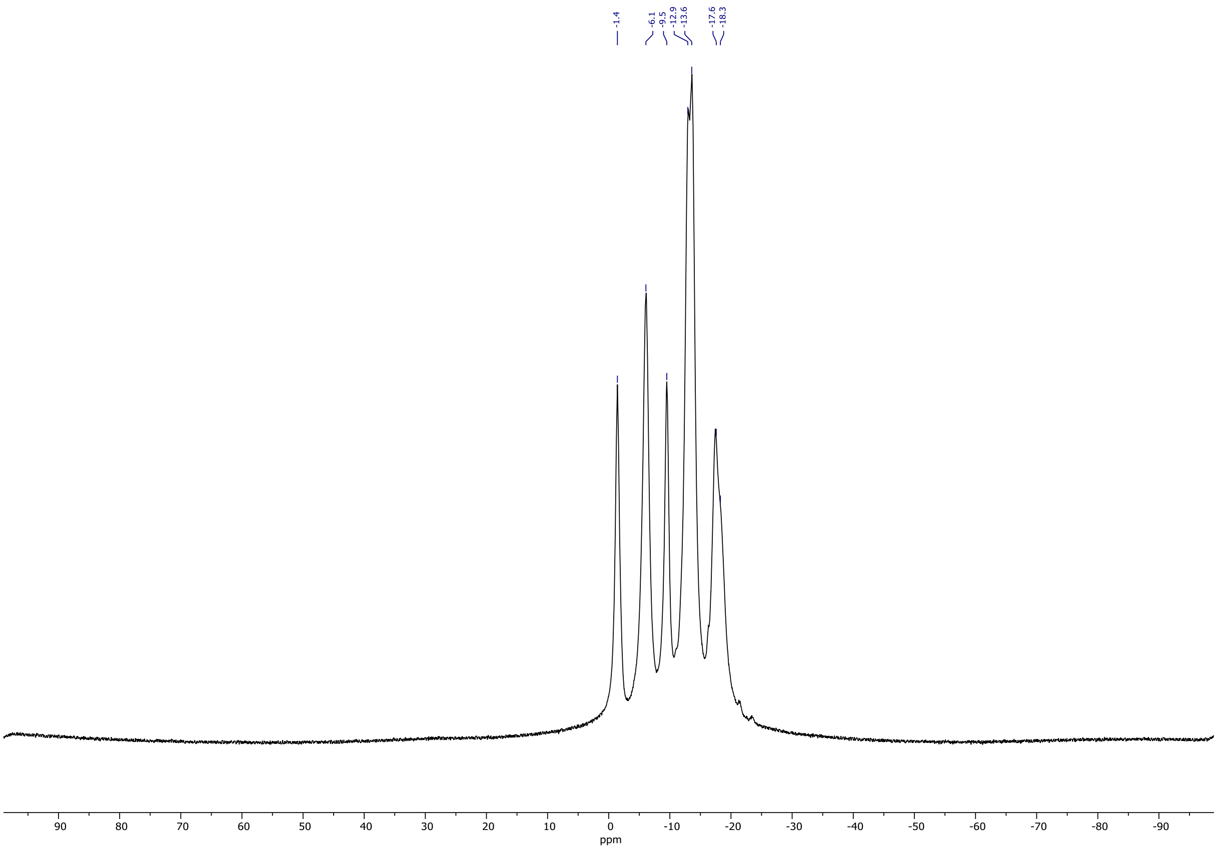

¹H NMR (CDCl$_3$, 400 MHz) for **5l**

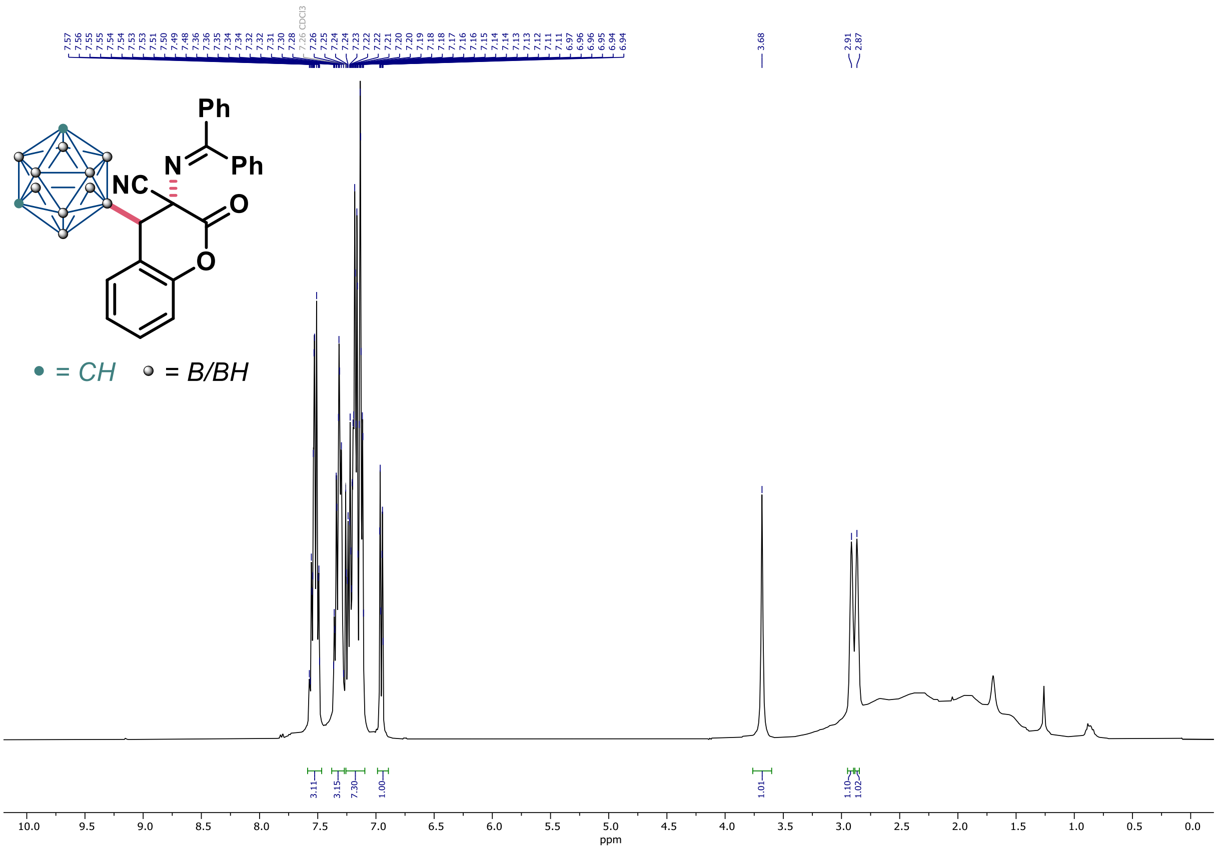

¹³C{¹H} NMR (CDCl$_3$, 101 MHz) for **5l**

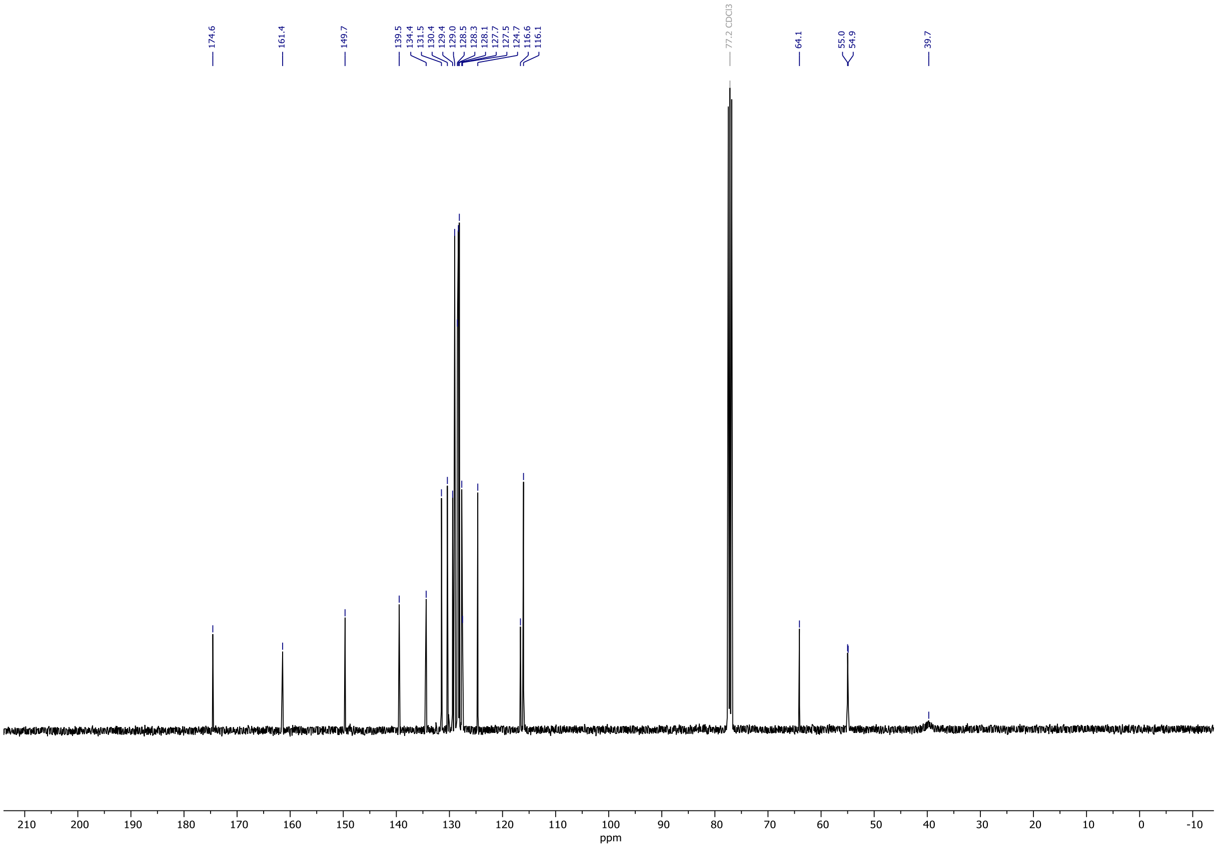

^{11}B{^{1}H} NMR (CDCl$_3$, 128 MHz) for **5l**

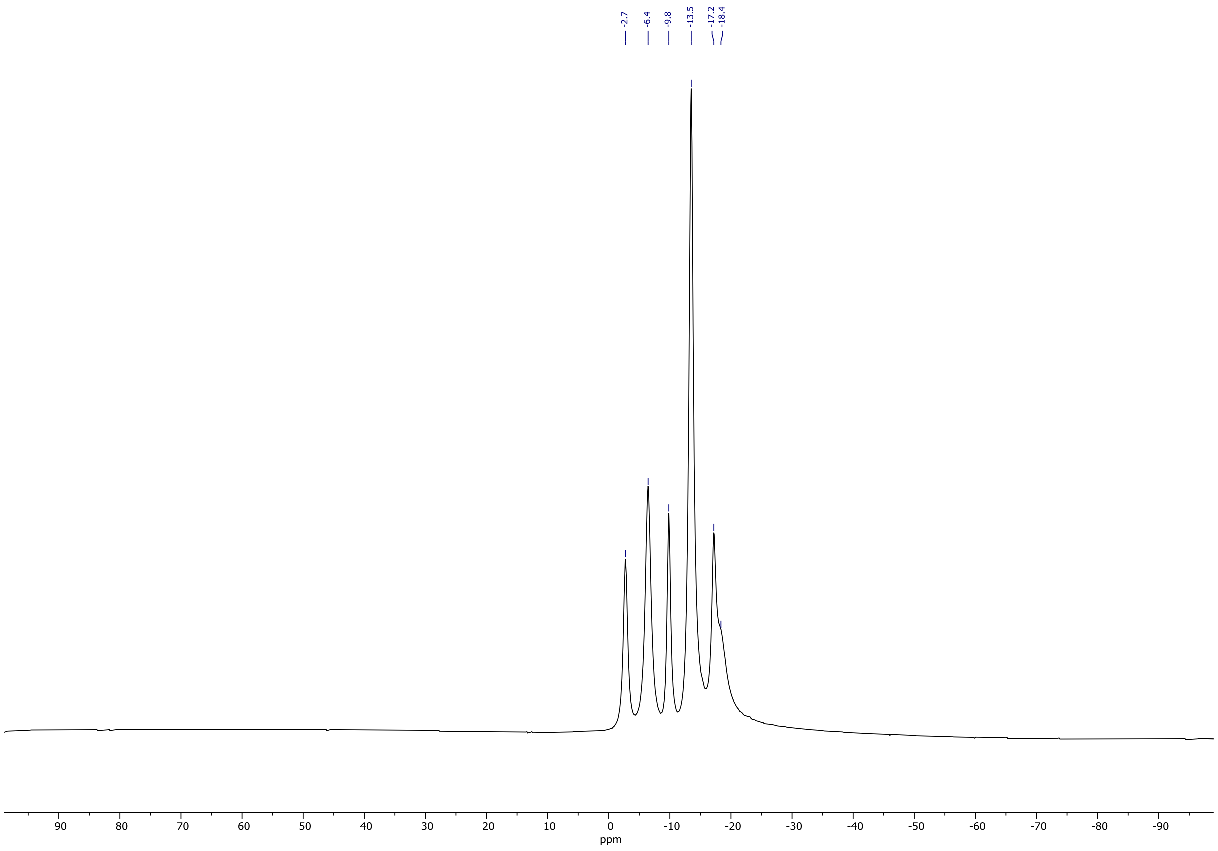

^{1}H NMR ($CDCl_3$, 400 MHz) for **5m**

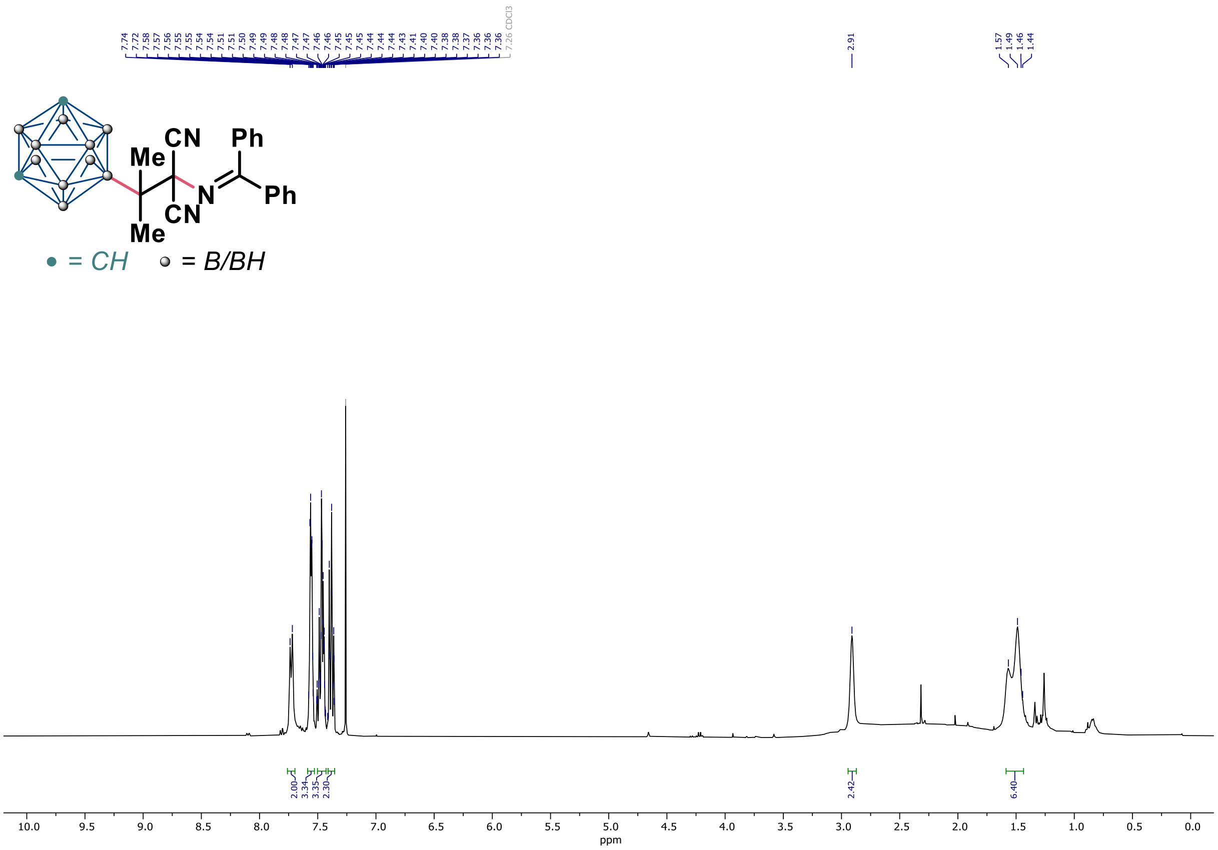

^{13}C{^{1}H} NMR ($CDCl_3$, 101 MHz) for **5m**

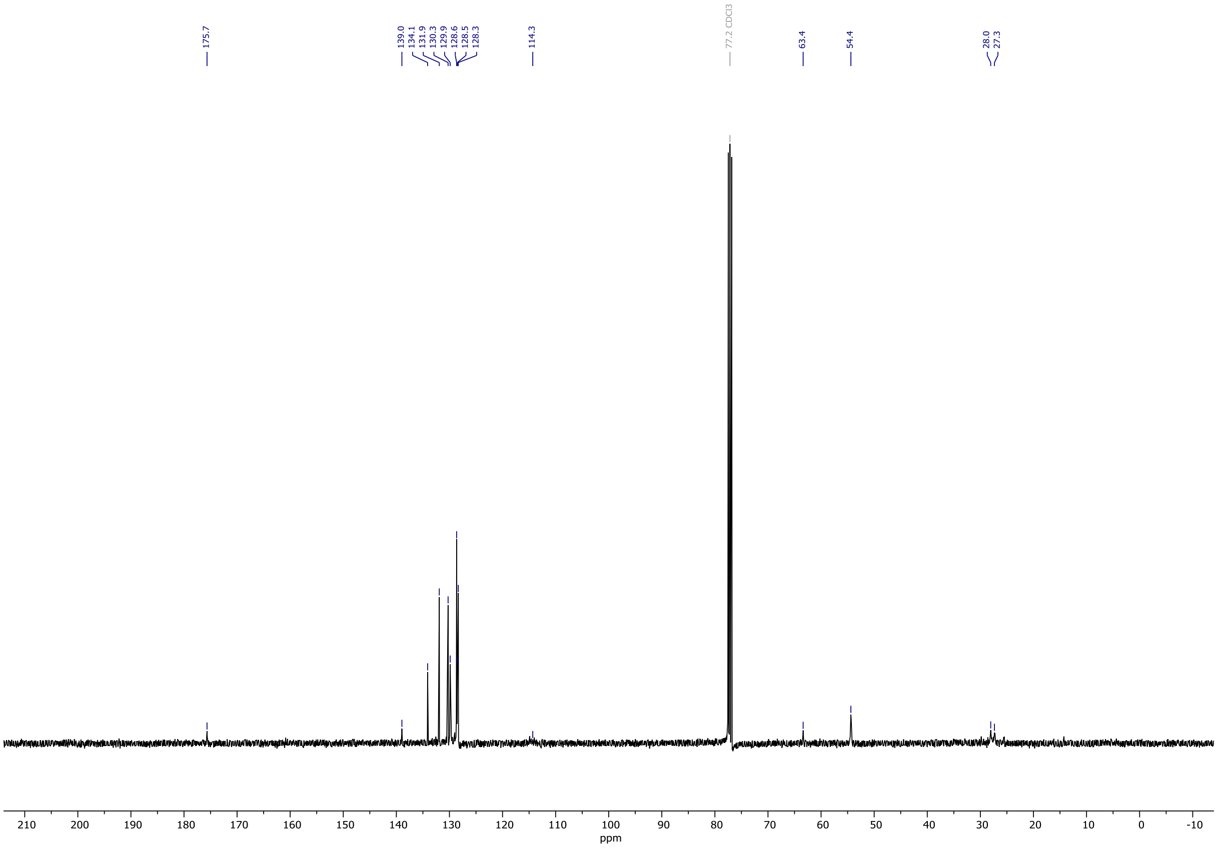

$^{11}B\{^1H\}$ NMR ($CDCl_3$, 128 MHz) for **5m**

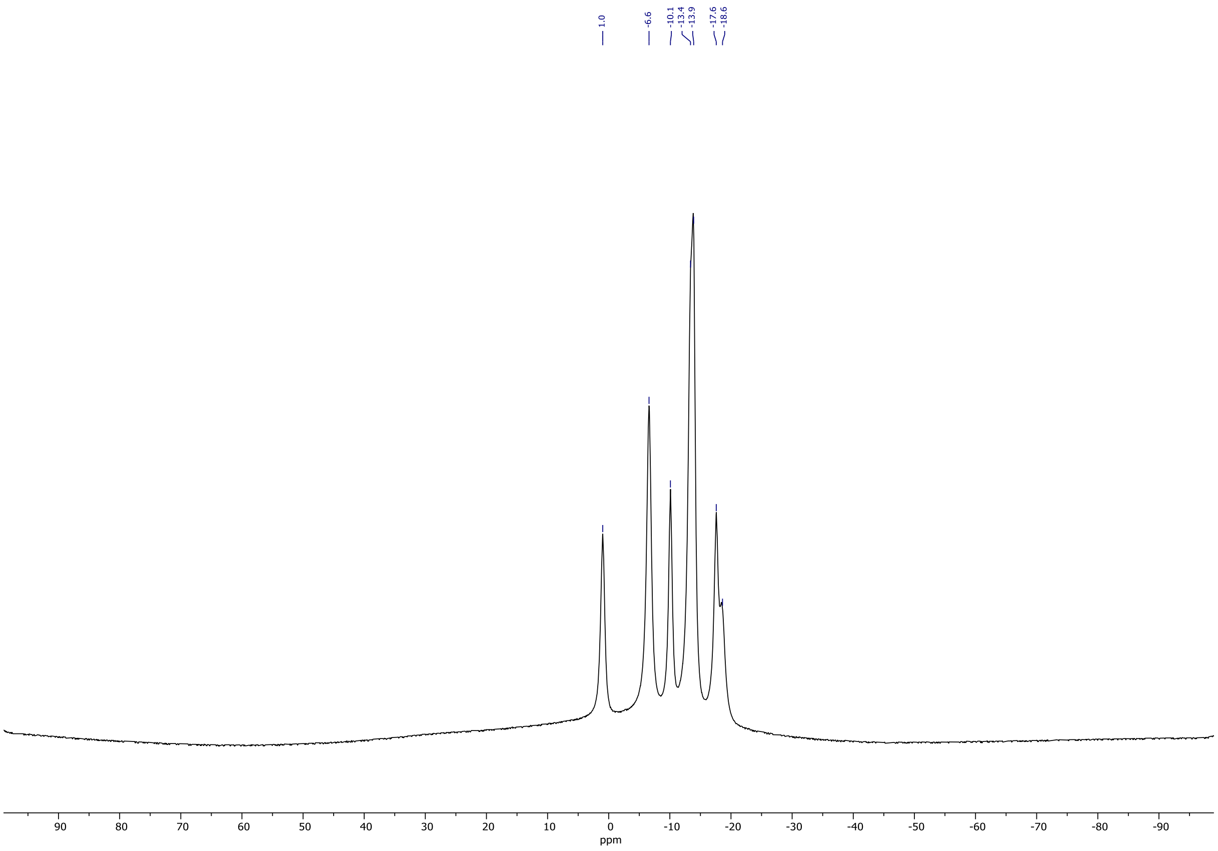

¹H NMR (CDCl$_3$, 400 MHz) for **5n**

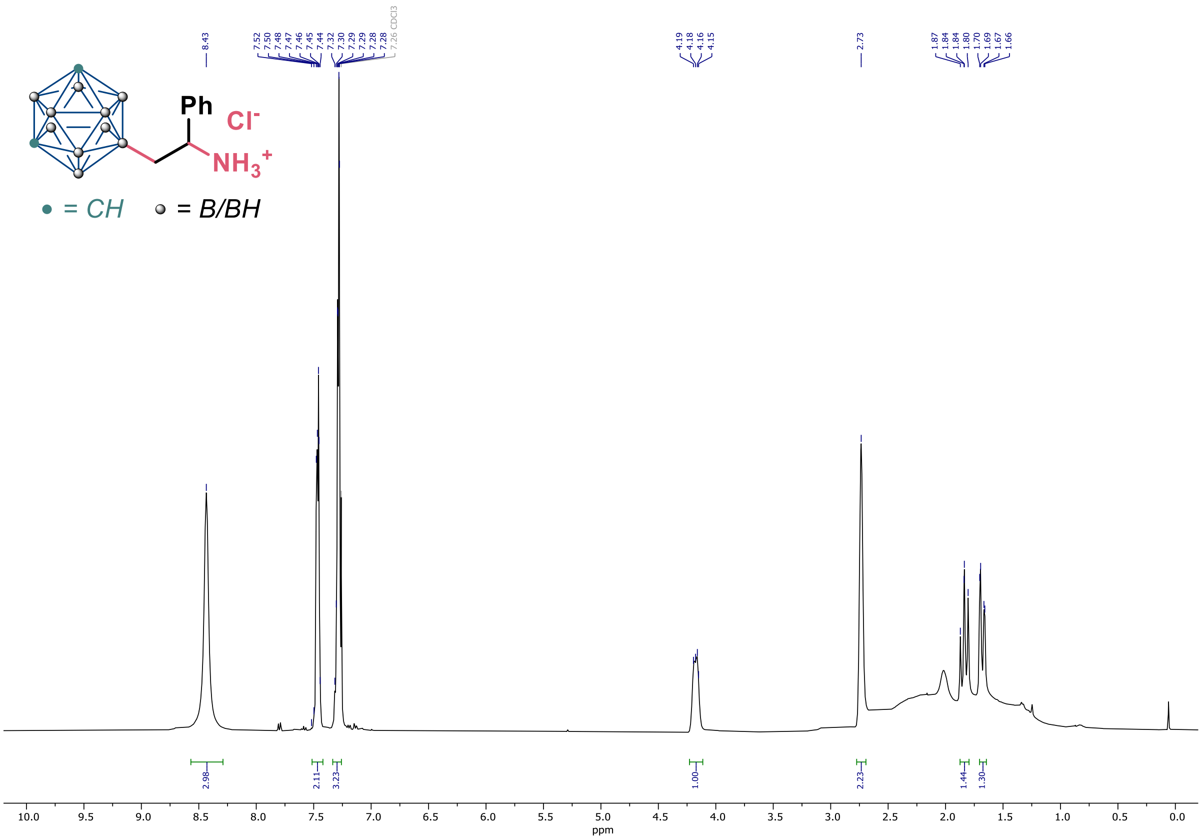

¹³C{¹H} NMR (CDCl$_3$, 101 MHz) for **5n**

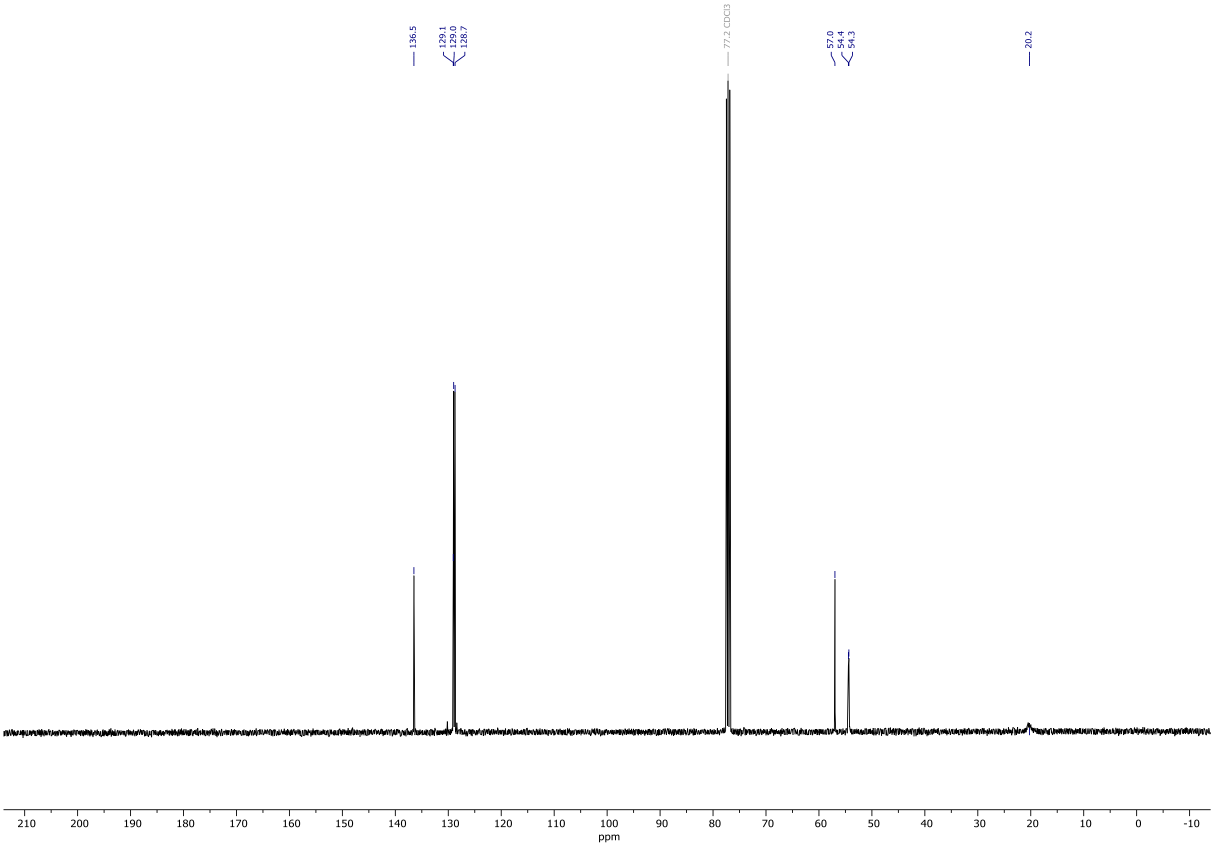

^{11}B{^{1}H} NMR (CDCl$_3$, 128 MHz) for **5n**

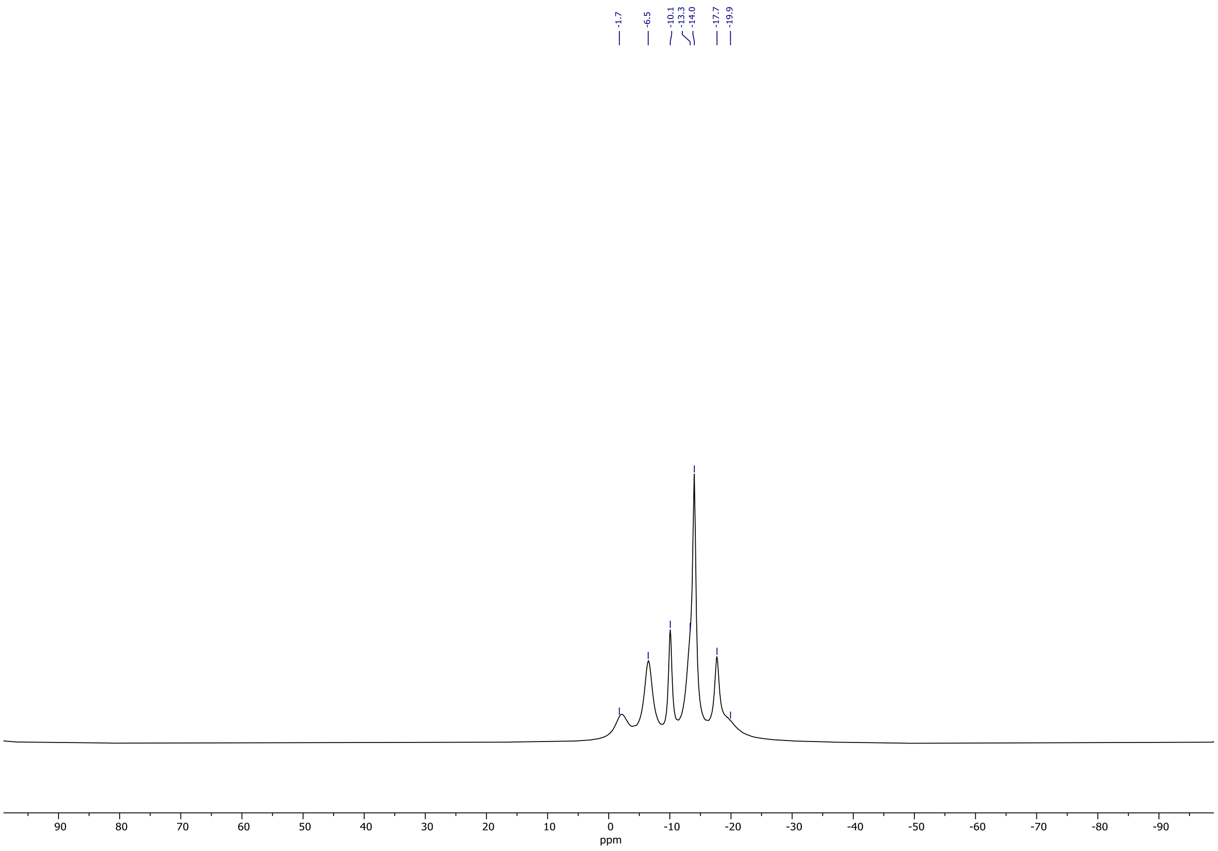

¹H NMR (D_2O, 400 MHz) for **5o**

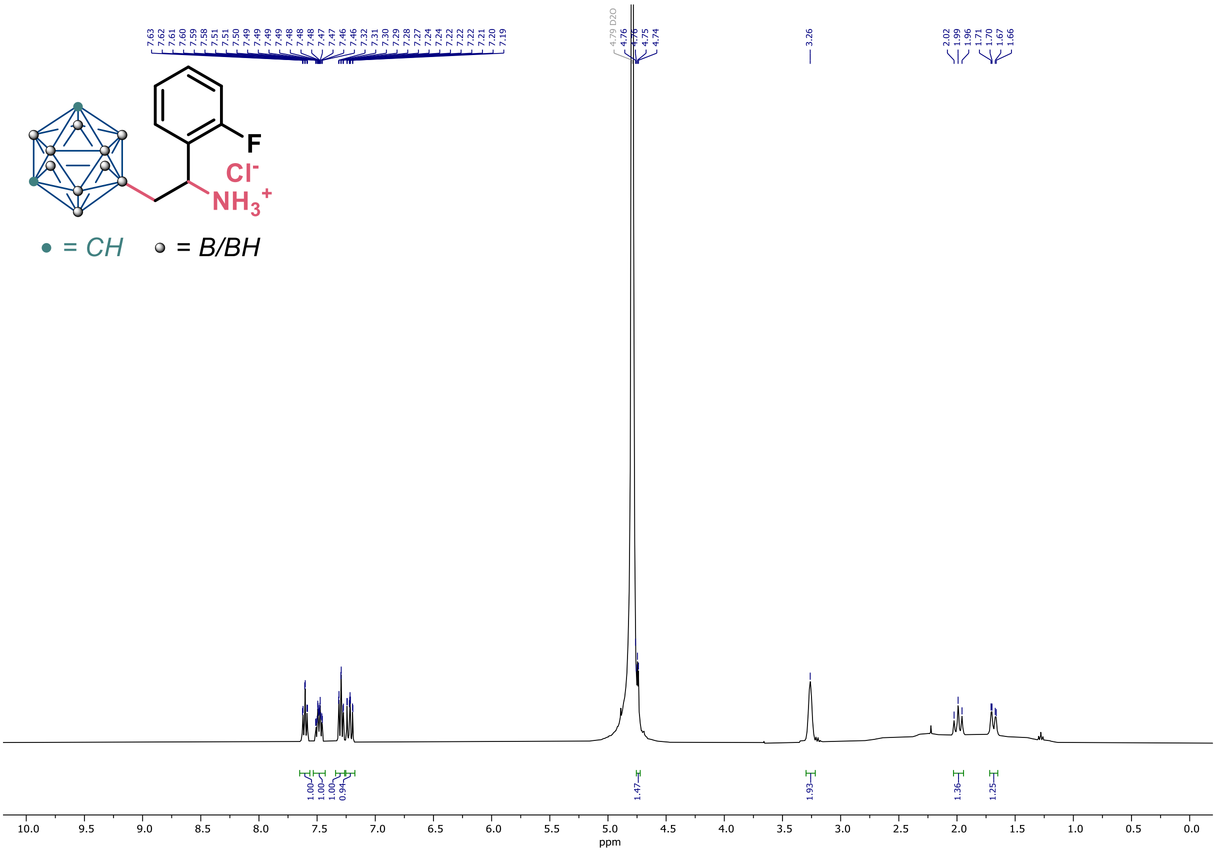

¹³C{¹H} NMR (D_2O, 101 MHz) for **5o**

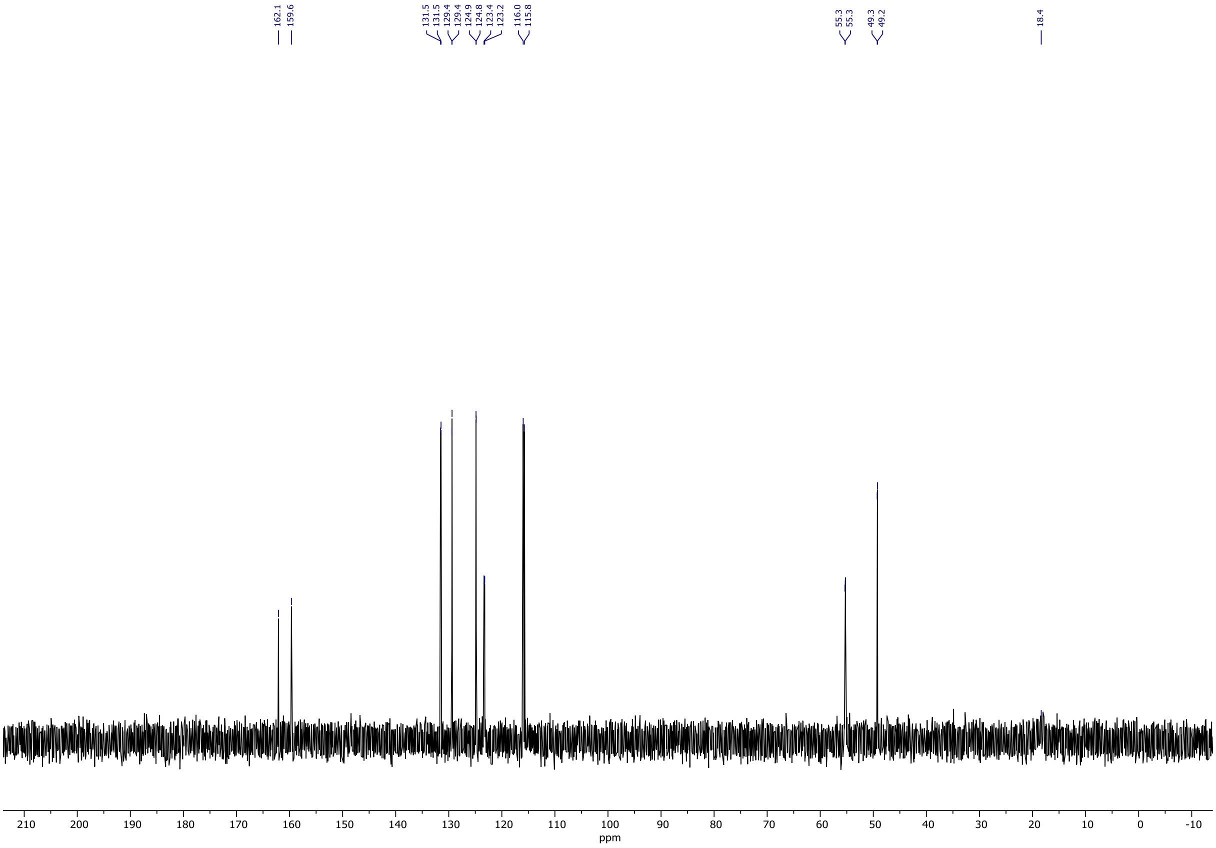

$^{11}B\{^1H\}$ NMR (D_2O, 128 MHz) for **5o**

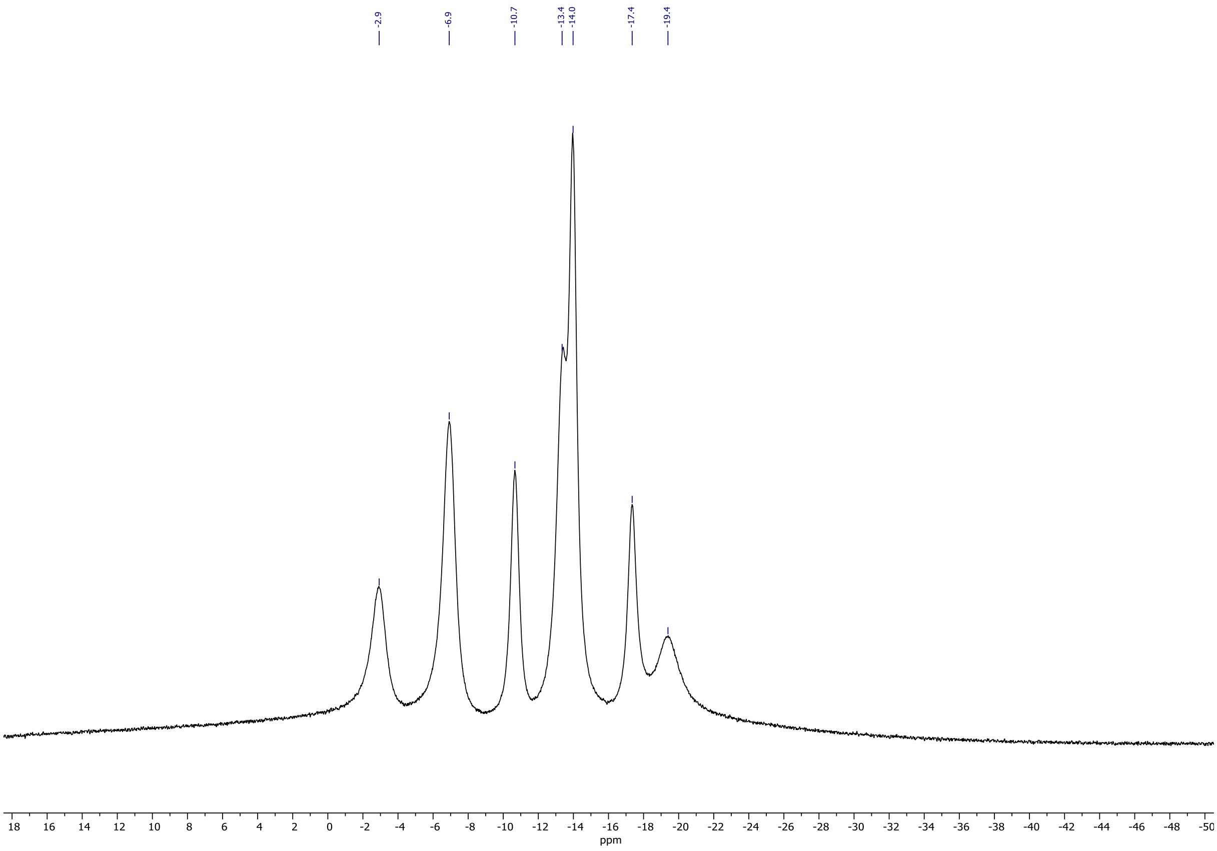

^{19}F NMR (D_2O, 376 MHz) for **5o**

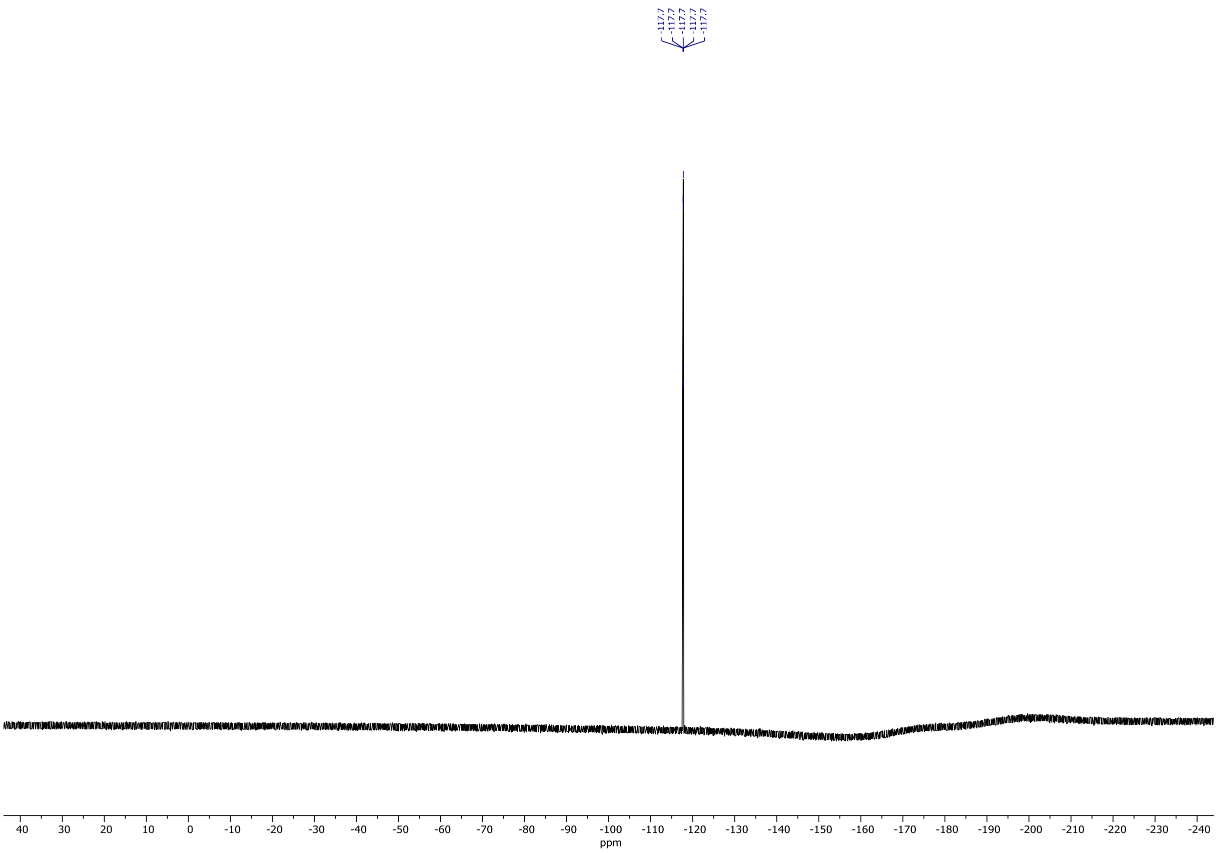

^{1}H NMR (D_2O, 400 MHz) for **5p**

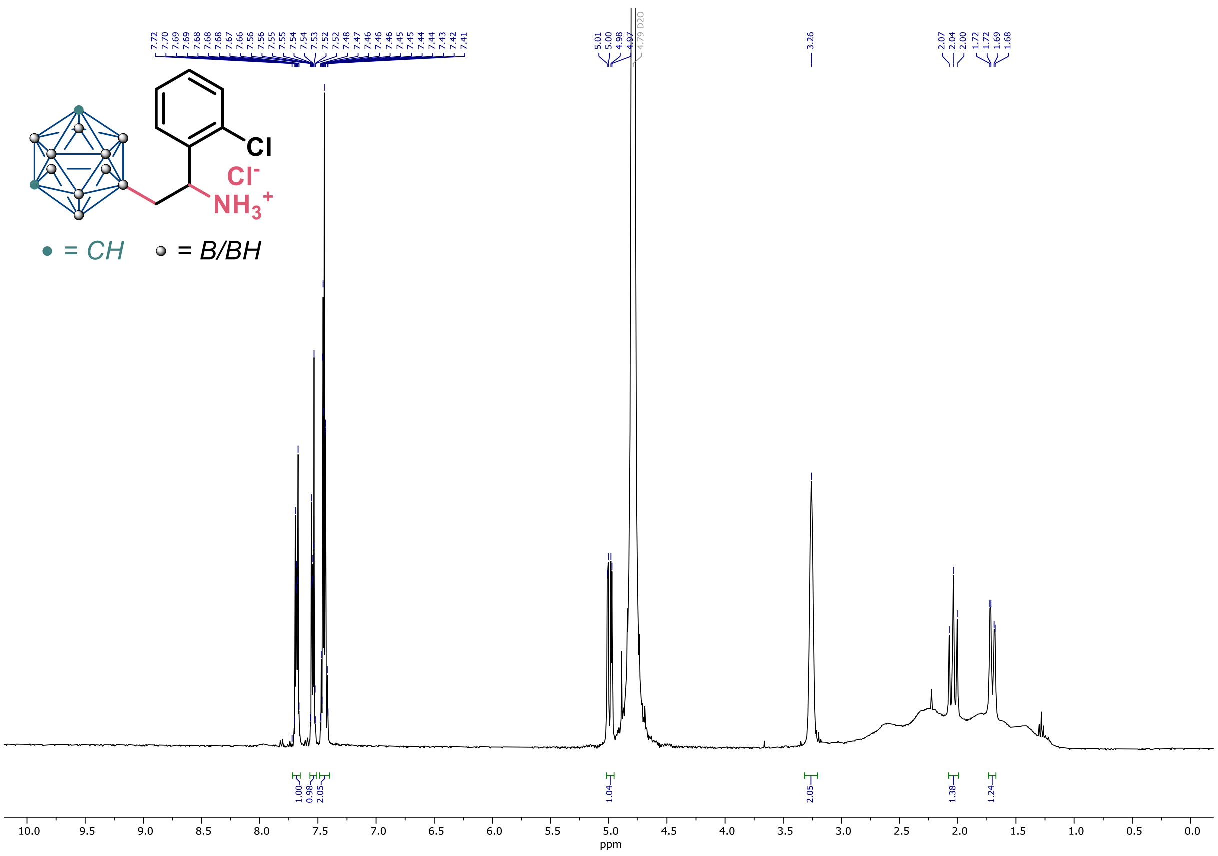

^{13}C{^{1}H} NMR (D_2O, 101 MHz) for **5p**

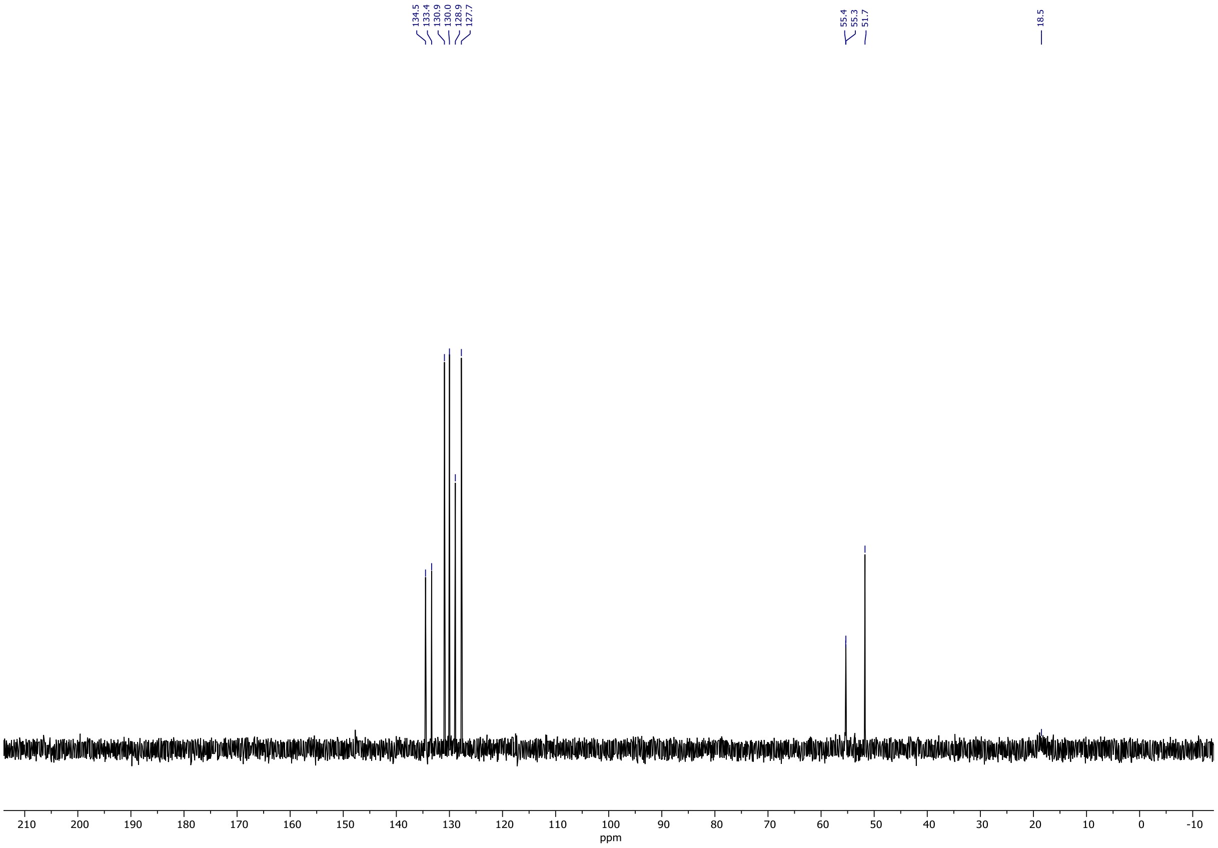

^{11}B{^{1}H} NMR (D_2O, 128 MHz) for **5p**

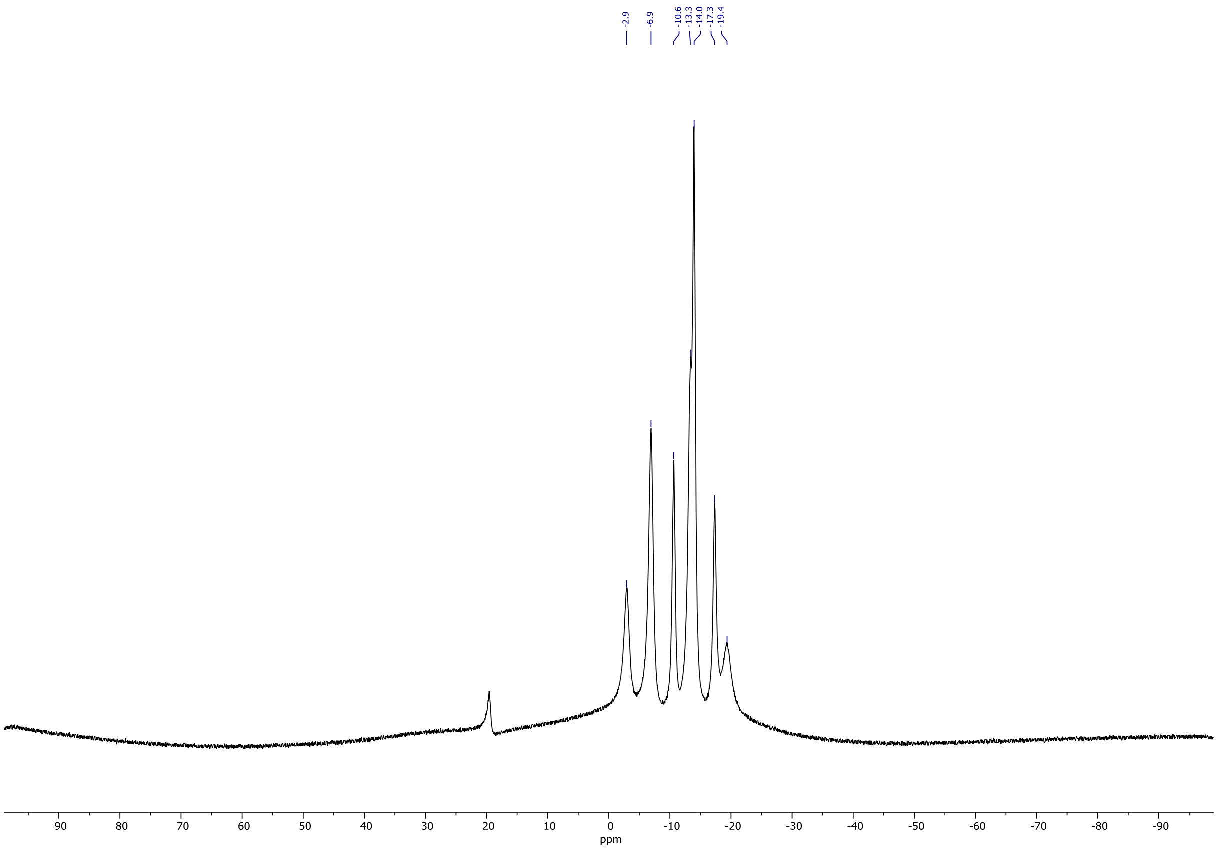

^{1}H NMR (CDCl$_3$, 400 MHz) for **5q**

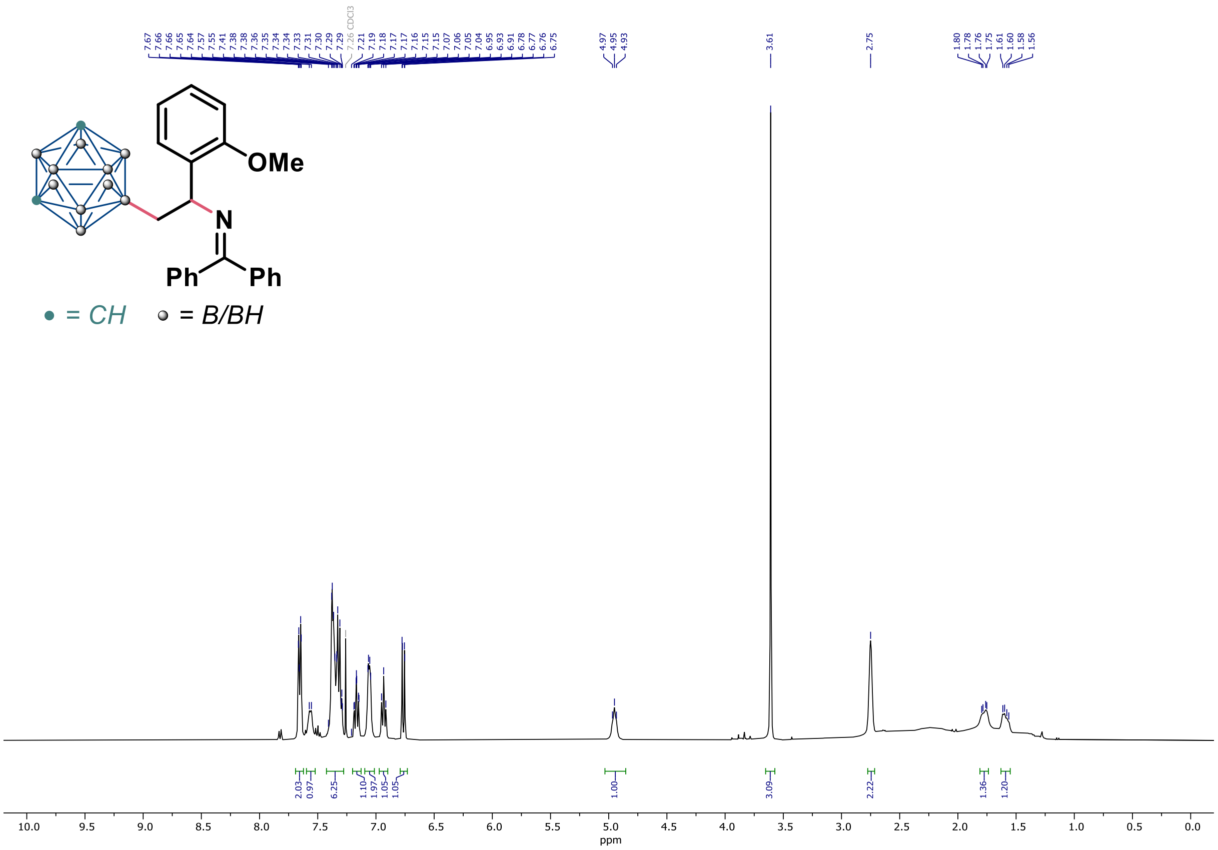

^{13}C{^{1}H} NMR (CDCl$_3$, 101 MHz) for **5q**

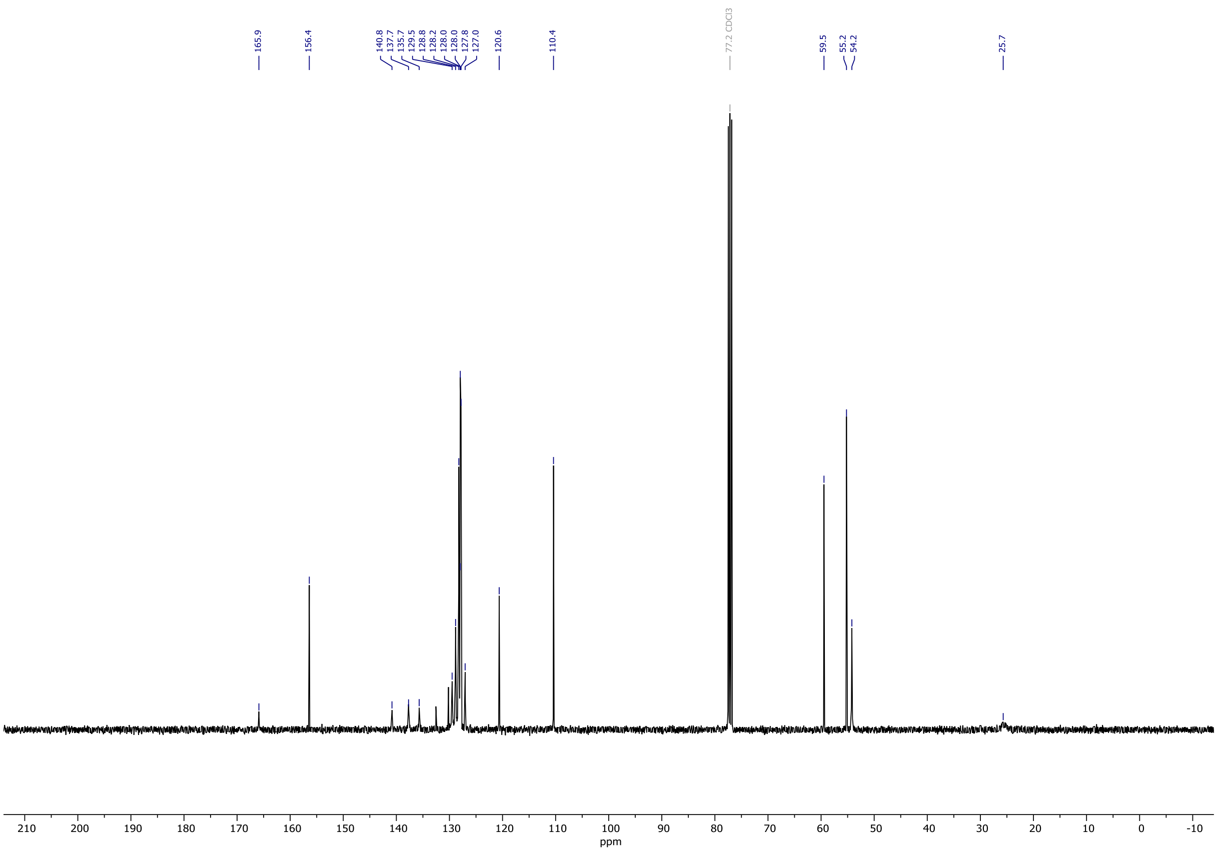

^{11}B{^{1}H} NMR (CDCl$_3$, 128 MHz) for **5q**

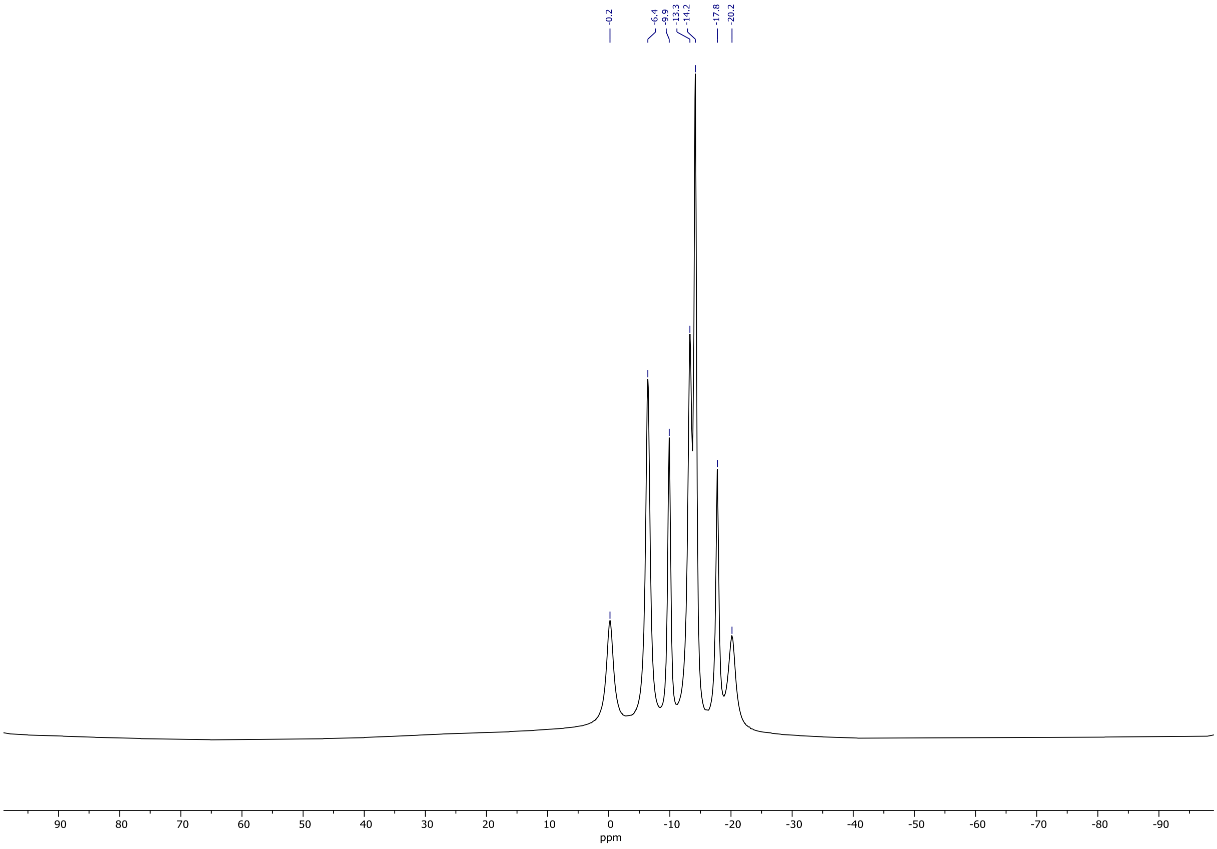

^{1}H NMR (CDCl$_3$, 400 MHz) for **5r**

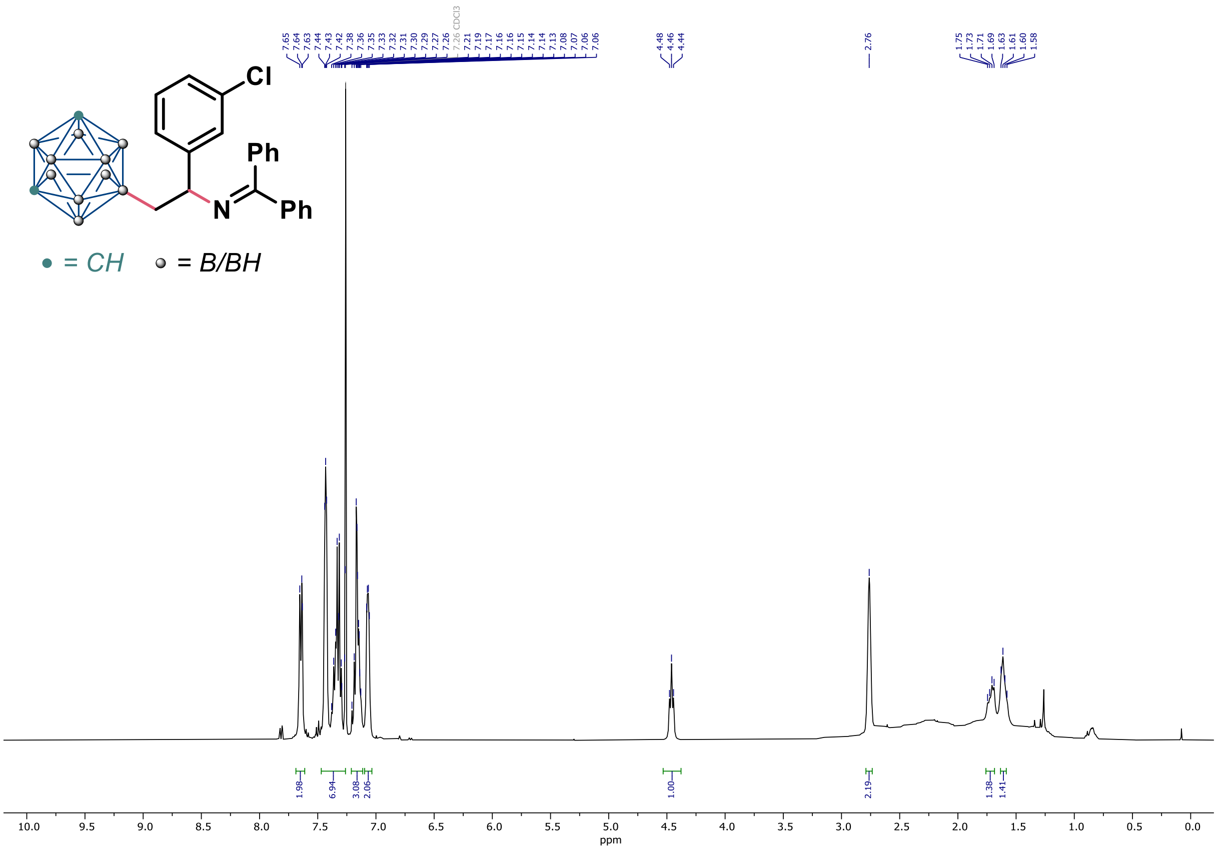

^{13}C{^{1}H} NMR (CDCl$_3$, 101 MHz) for **5r**

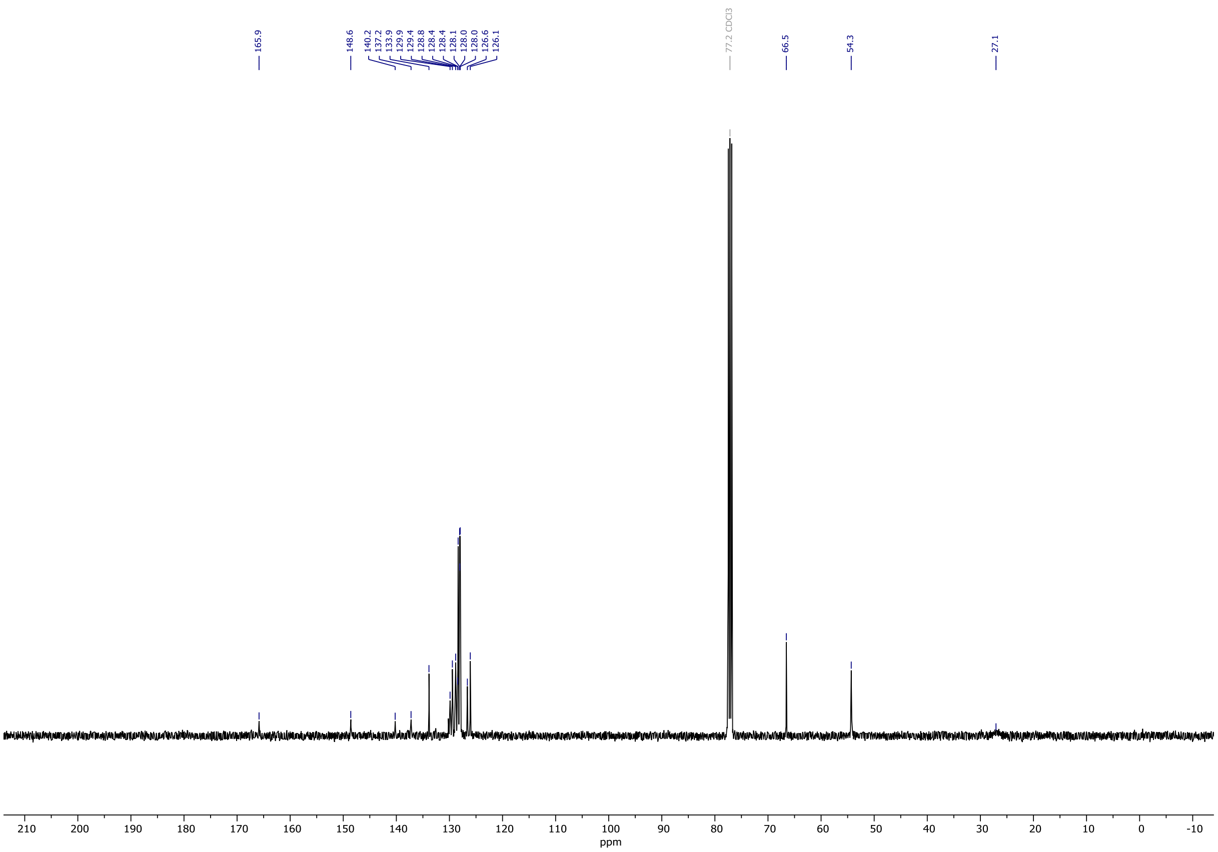

$^{11}B\{^1H\}$ NMR ($CDCl_3$, 128 MHz) for **5r**

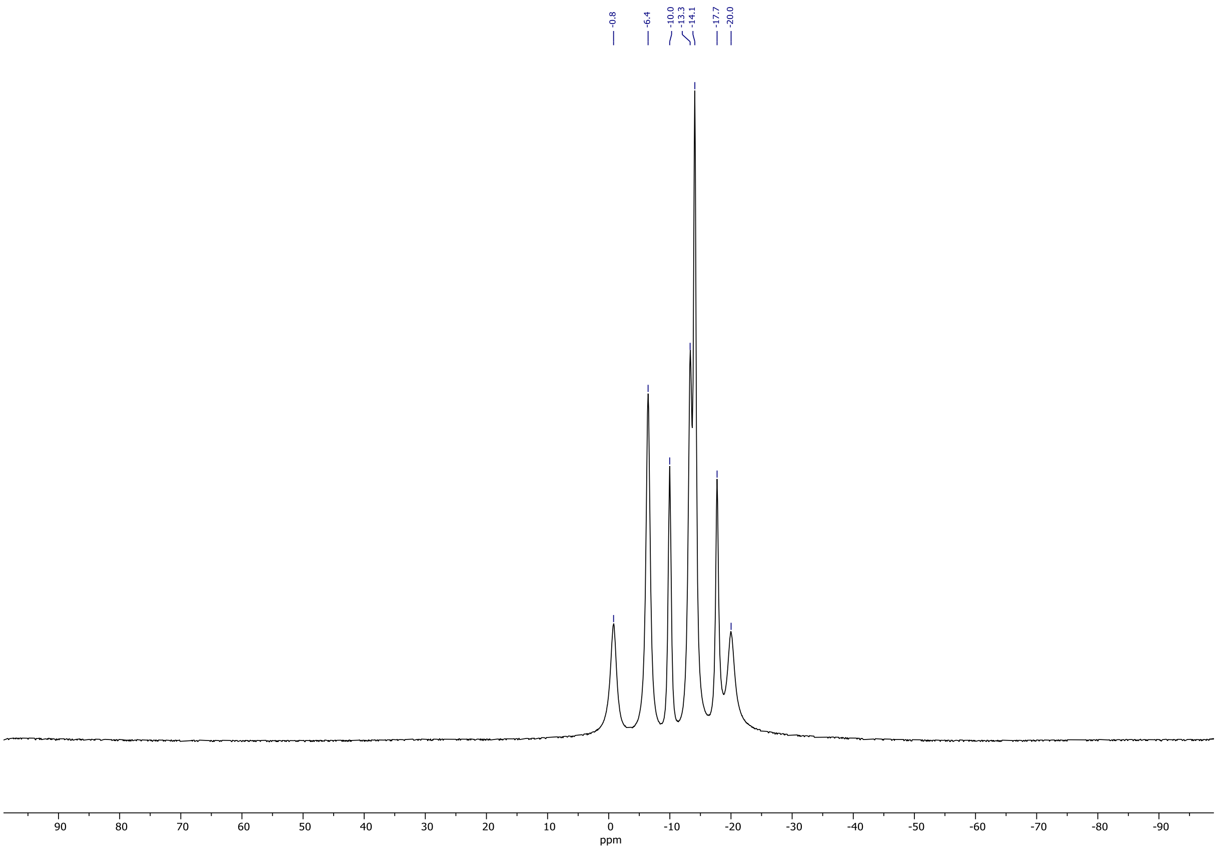

^{1}H NMR (CDCl$_3$, 400 MHz) for **5s**

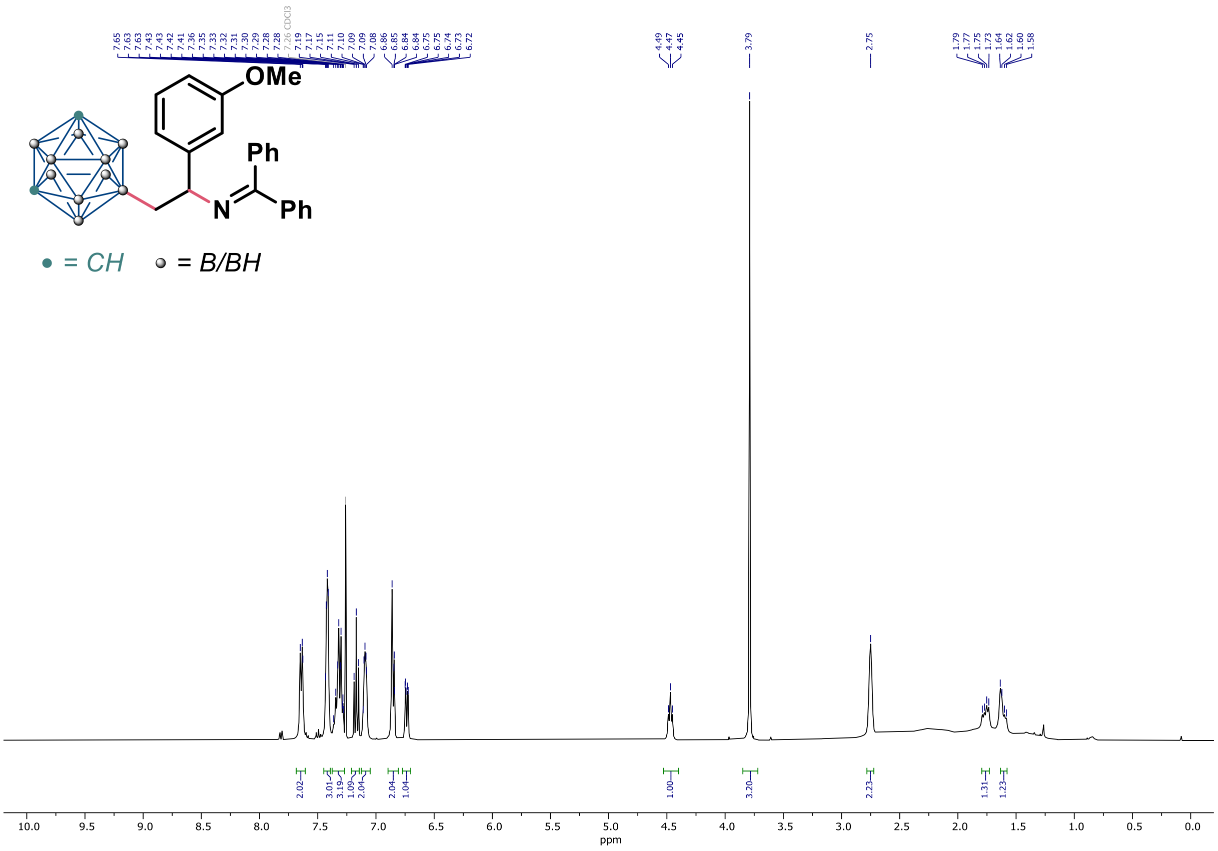

^{13}C{^{1}H} NMR (CDCl$_3$, 101 MHz) for **5s**

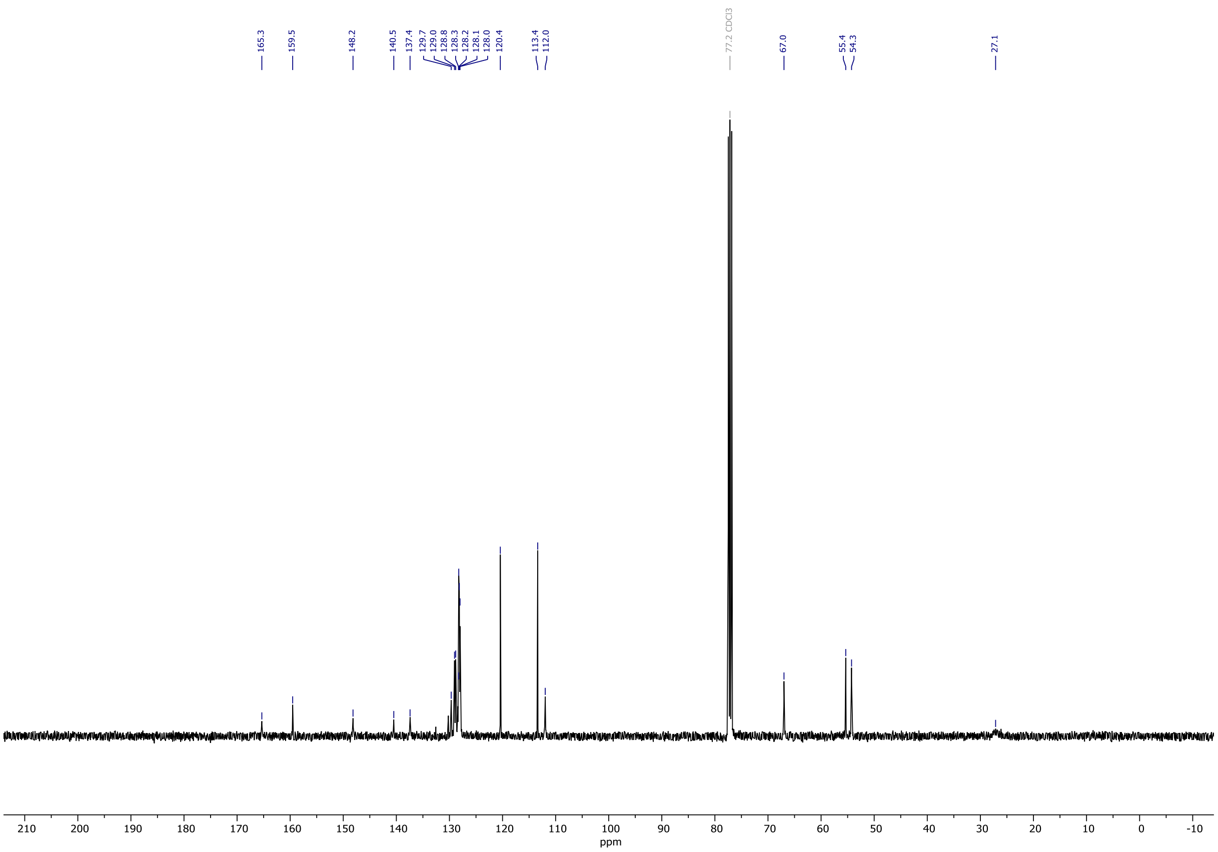

$^{11}B\{^1H\}$ NMR (CDCl$_3$, 128 MHz) for **5s**

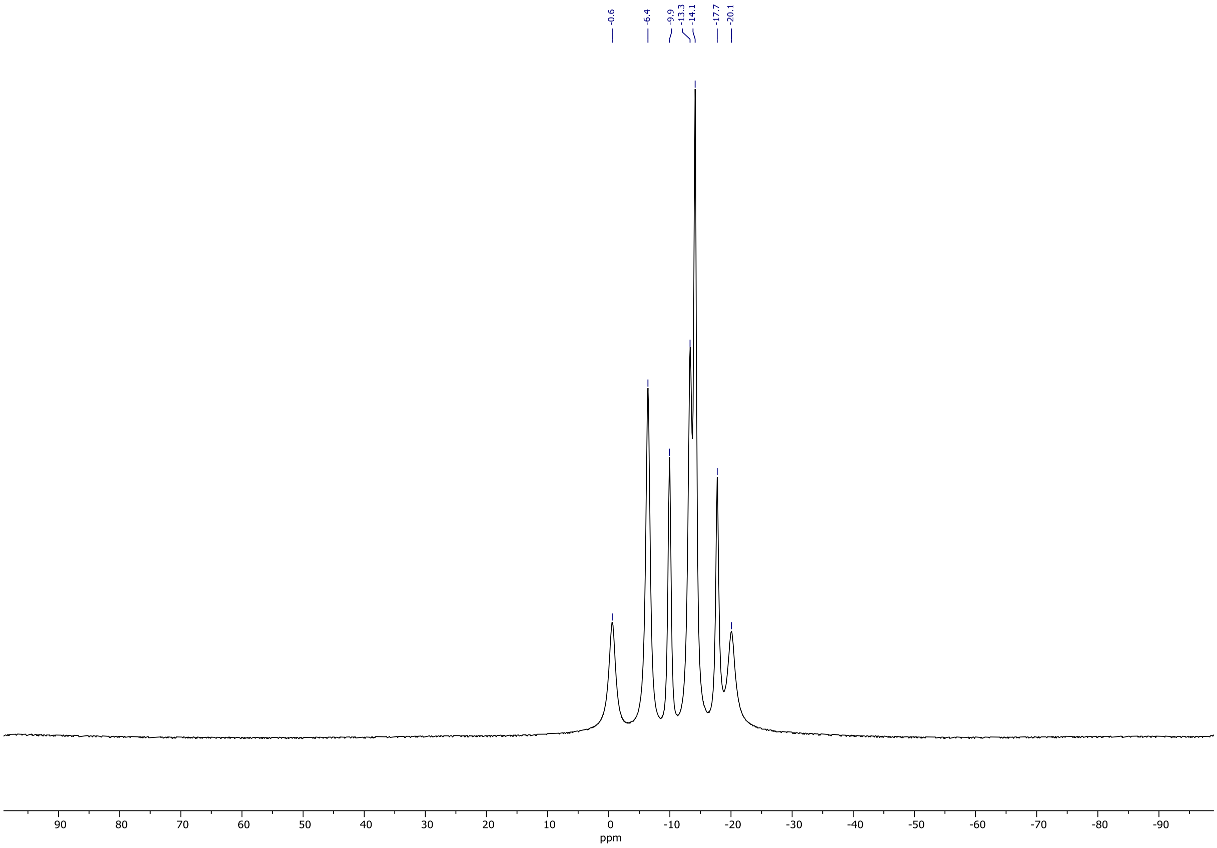

^{1}H NMR ($CDCl_3$, 400 MHz) for **5t**

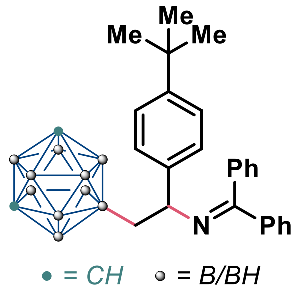

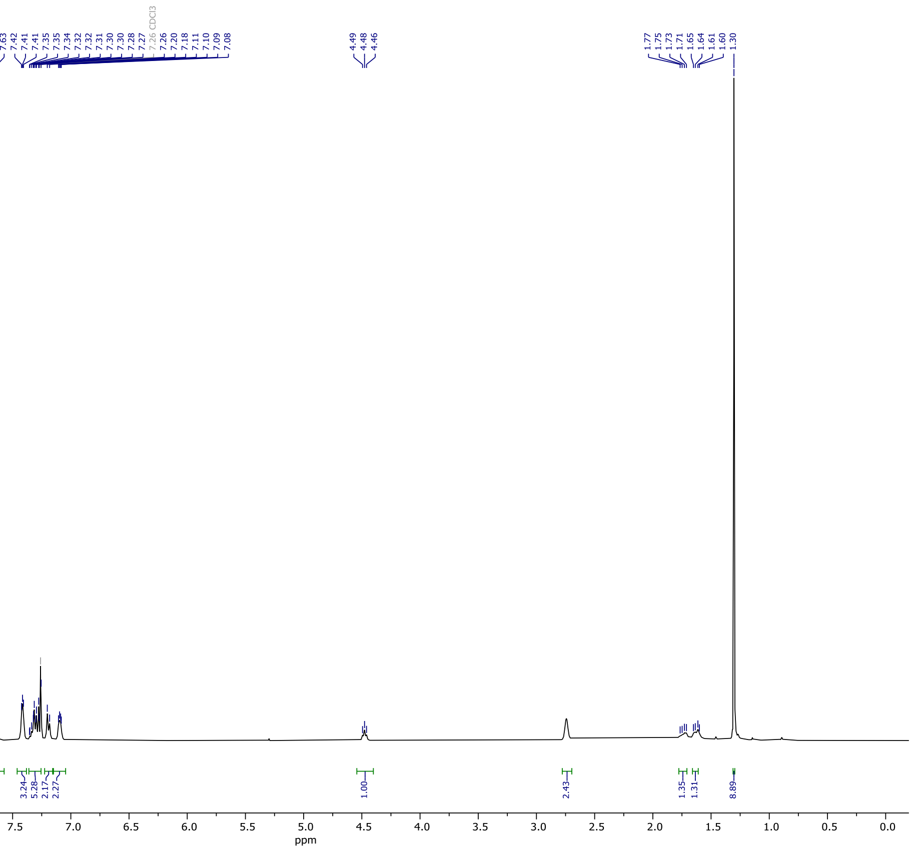

^{13}C{^{1}H} NMR ($CDCl_3$, 101 MHz) for **5t**

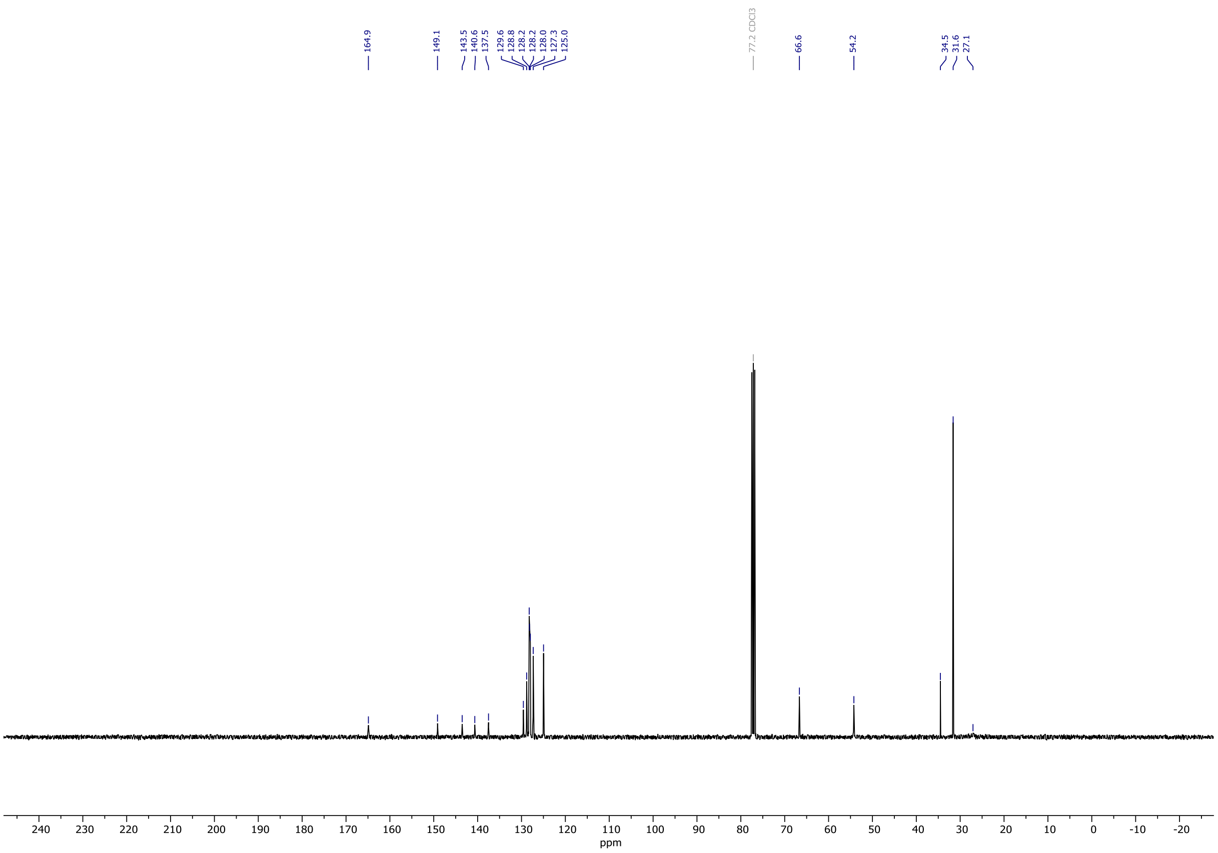

^{11}B{^{1}H} NMR (CDCl$_3$, 128 MHz) for **5t**

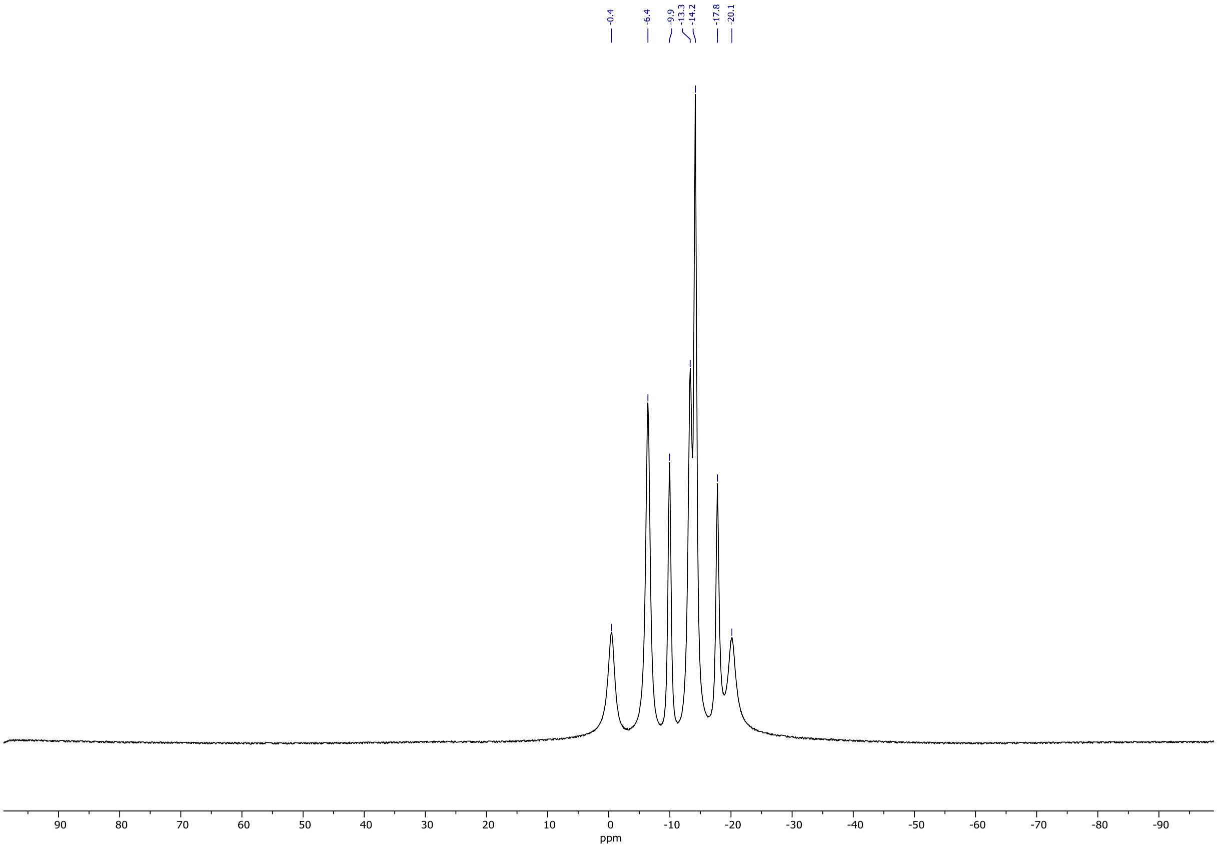

^{1}H NMR (CDCl$_3$, 400 MHz) for **5u**

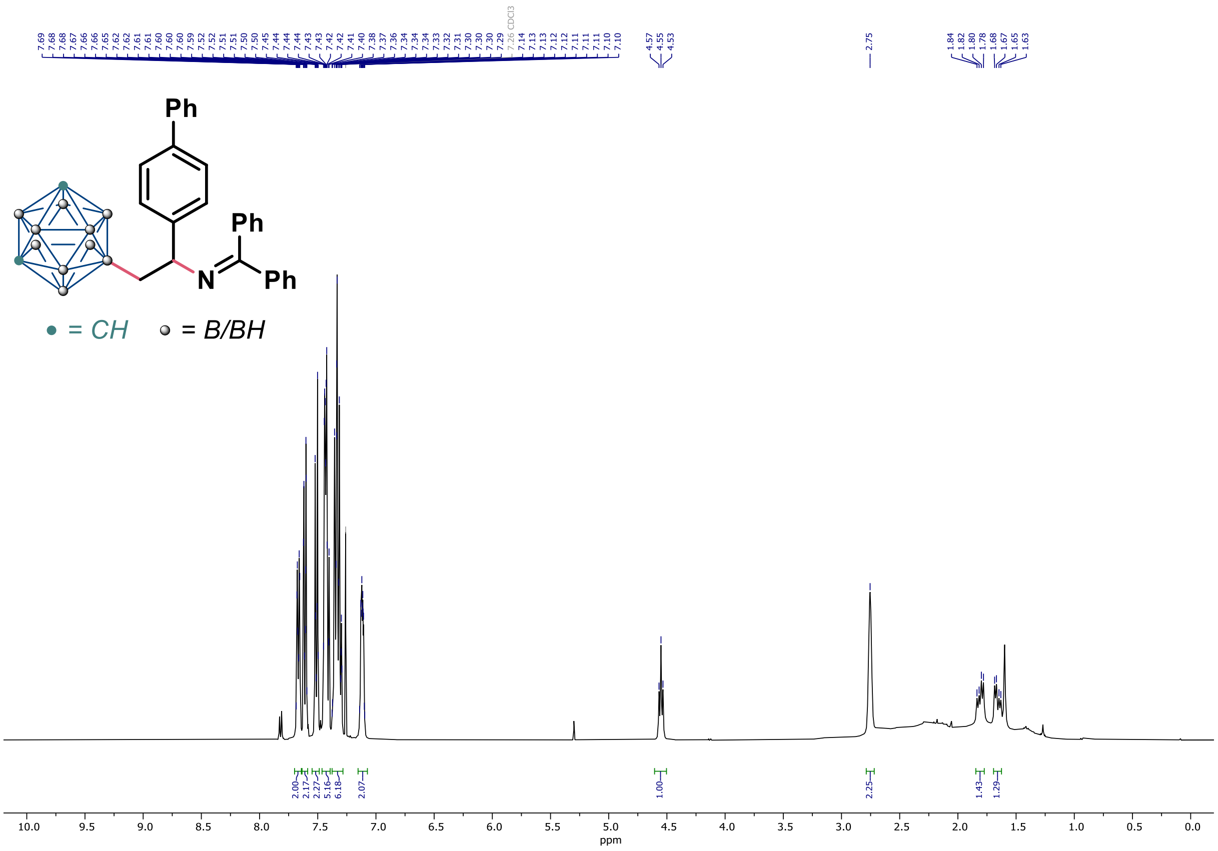

^{13}C{^{1}H} NMR (CDCl$_3$, 101 MHz) for **5u**

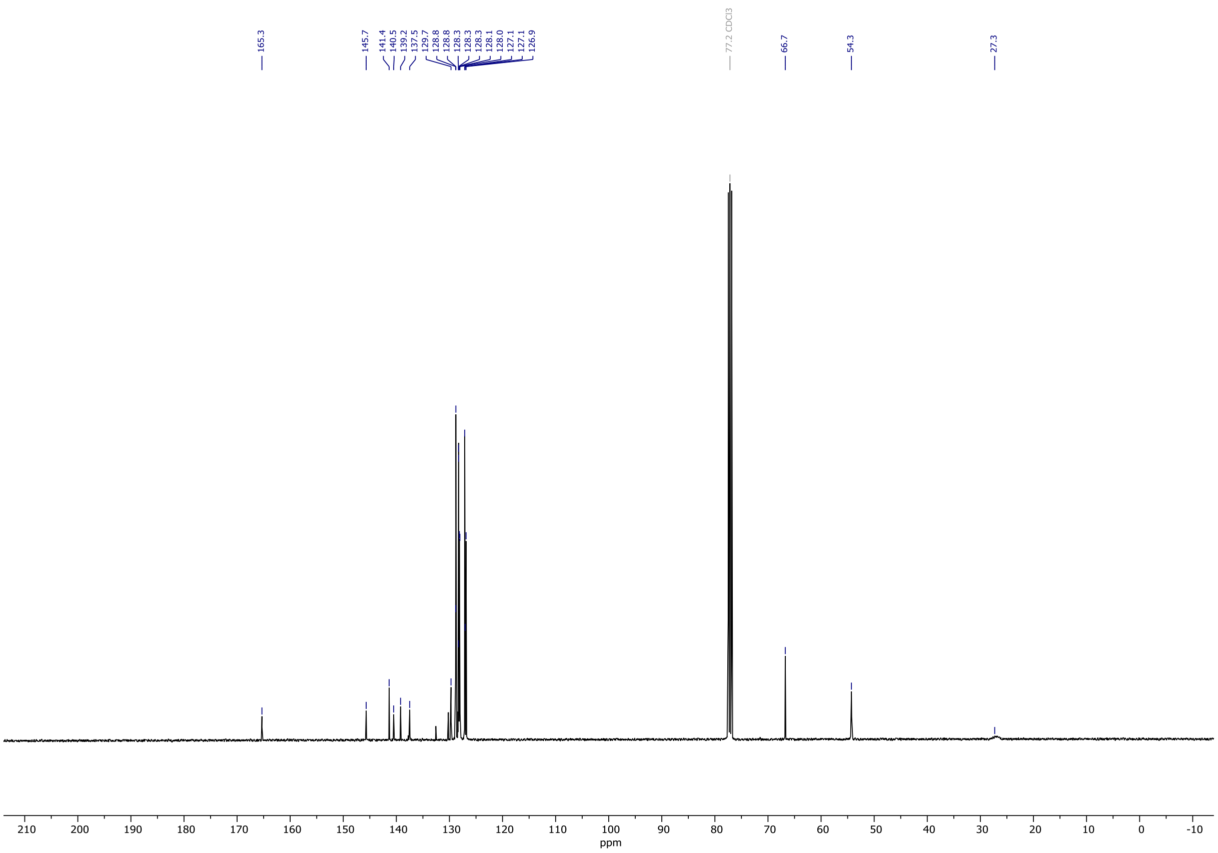

^{11}B{^{1}H} NMR (CDCl$_3$, 128 MHz) for **5u**

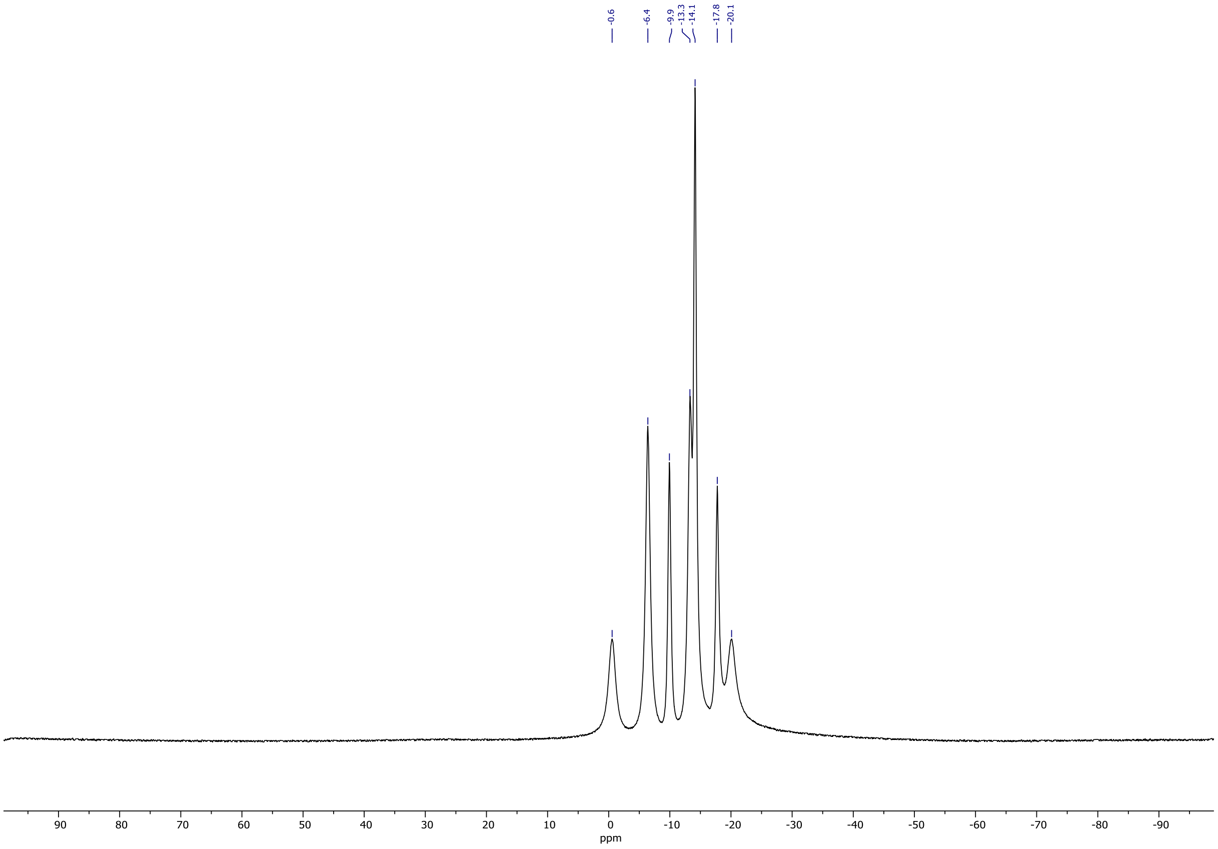

¹H NMR (CDCl$_3$, 400 MHz) for **5v**

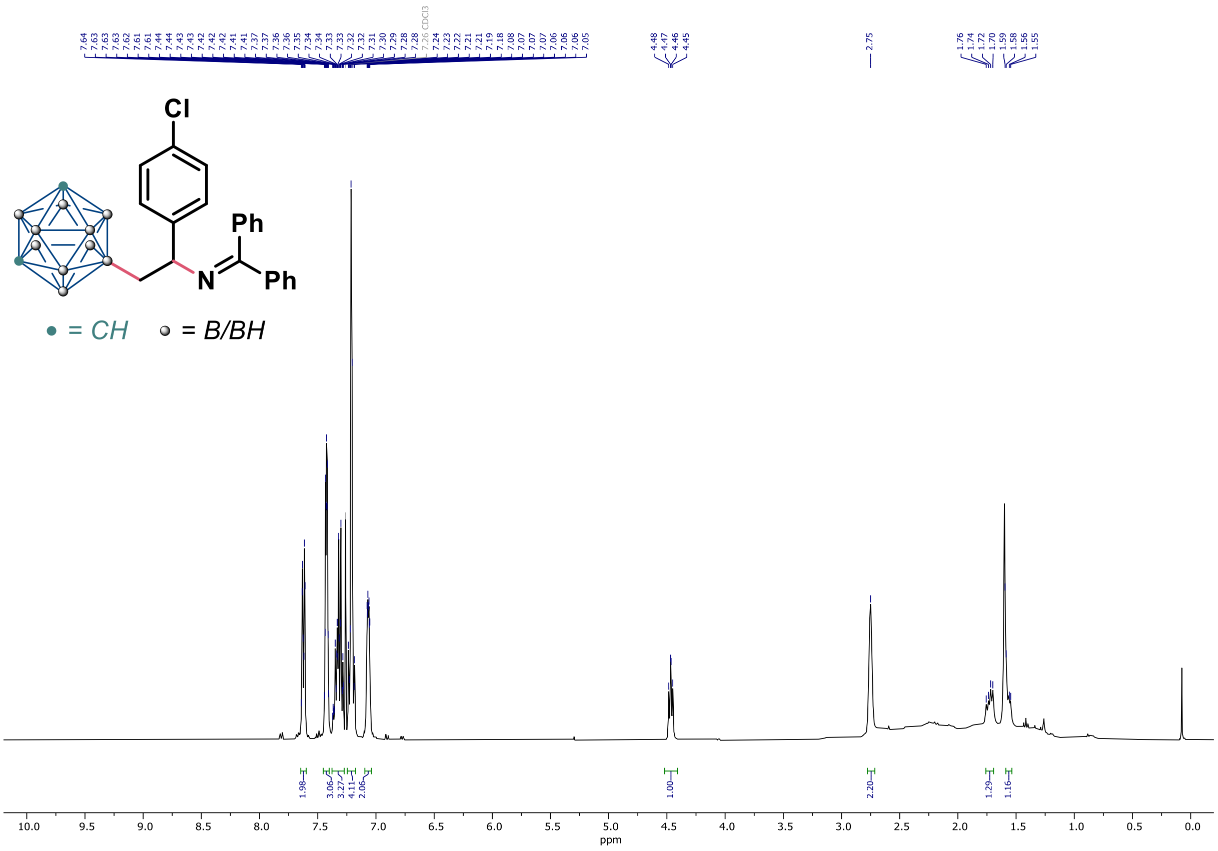

¹³C{¹H} NMR (CDCl$_3$, 101 MHz) for **5v**

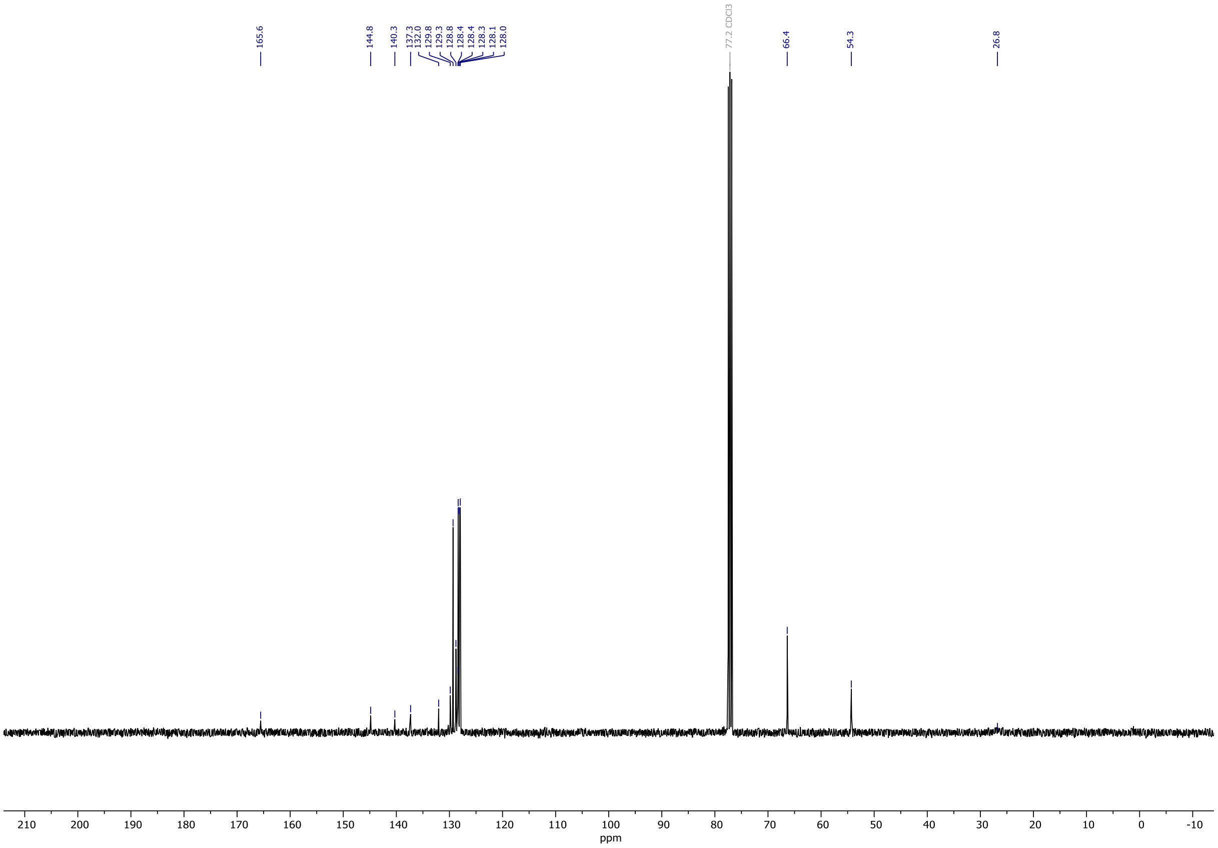

^{11}B{^{1}H} NMR (CDCl$_3$, 128 MHz) for **5v**

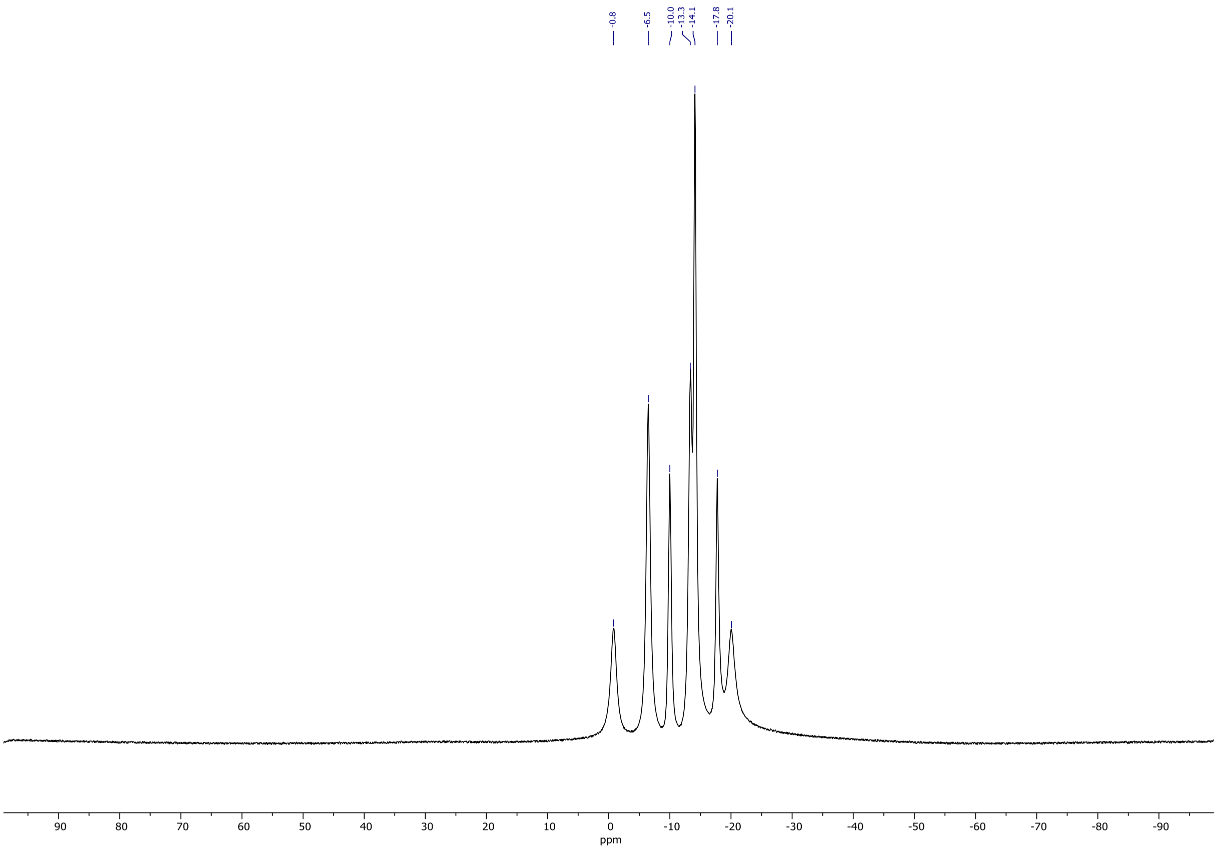

^{1}H NMR (CDCl$_3$, 400 MHz) for **5w**

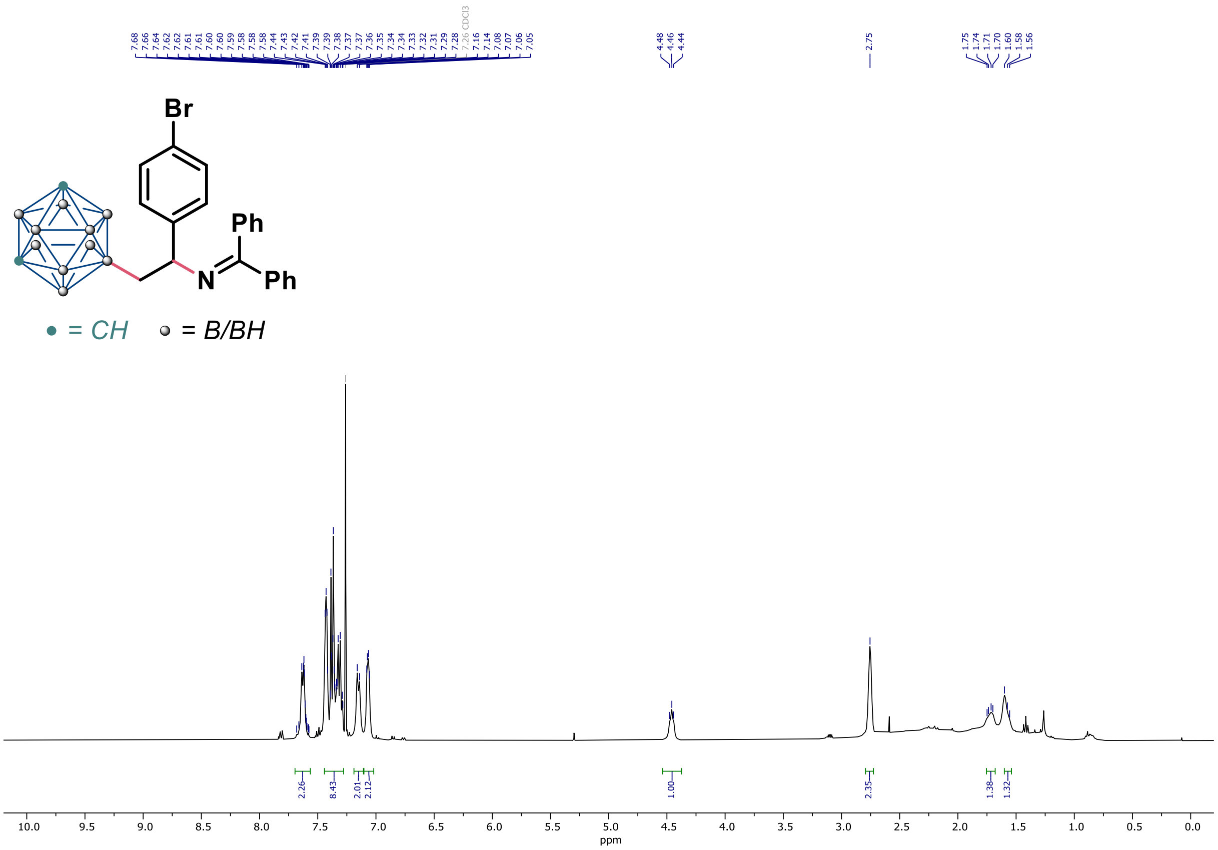

^{13}C{^{1}H} NMR (CDCl$_3$, 101 MHz) for **5w**

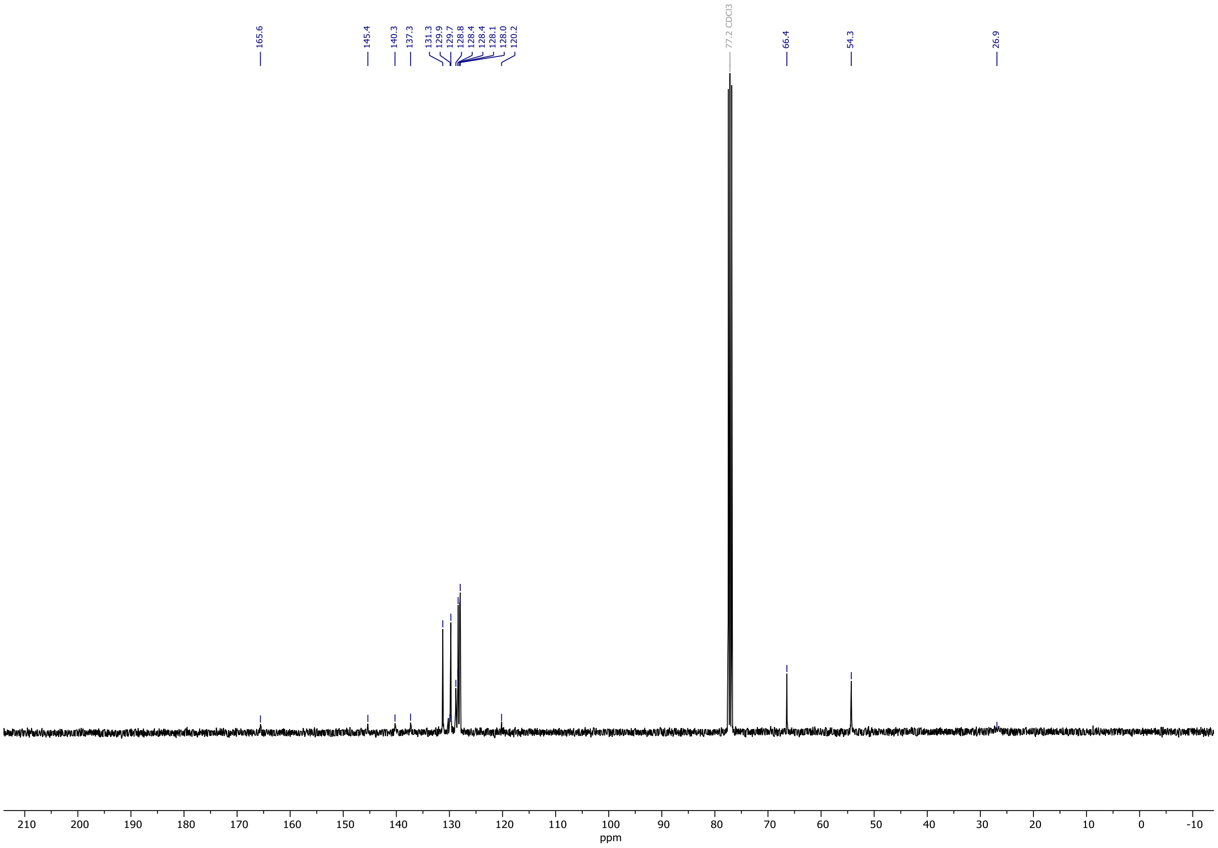

$^{11}B\{^1H\}$ NMR ($CDCl_3$, 128 MHz) for **5w**

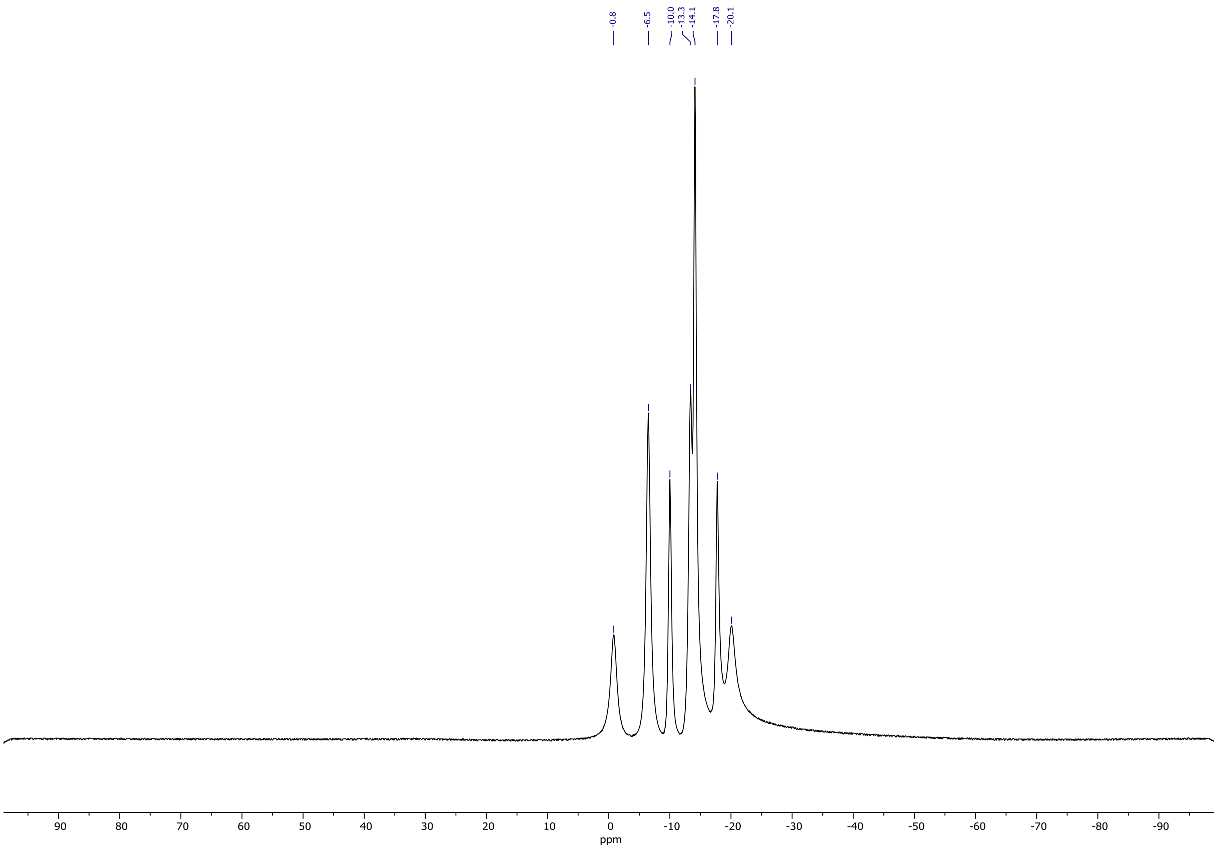

^{1}H NMR ($CDCl_3$, 400 MHz) for **5x**

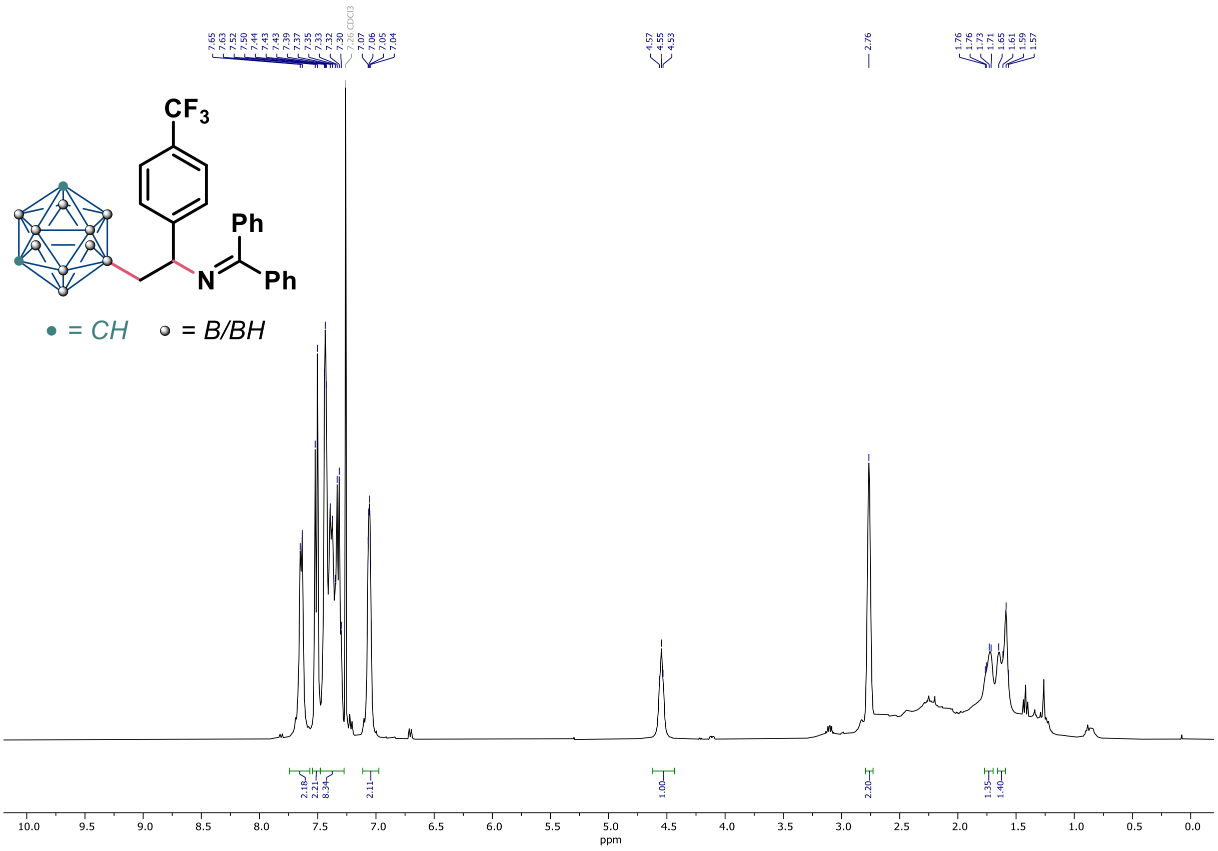

^{13}C{^{1}H} NMR ($CDCl_3$, 101 MHz) for **5x**

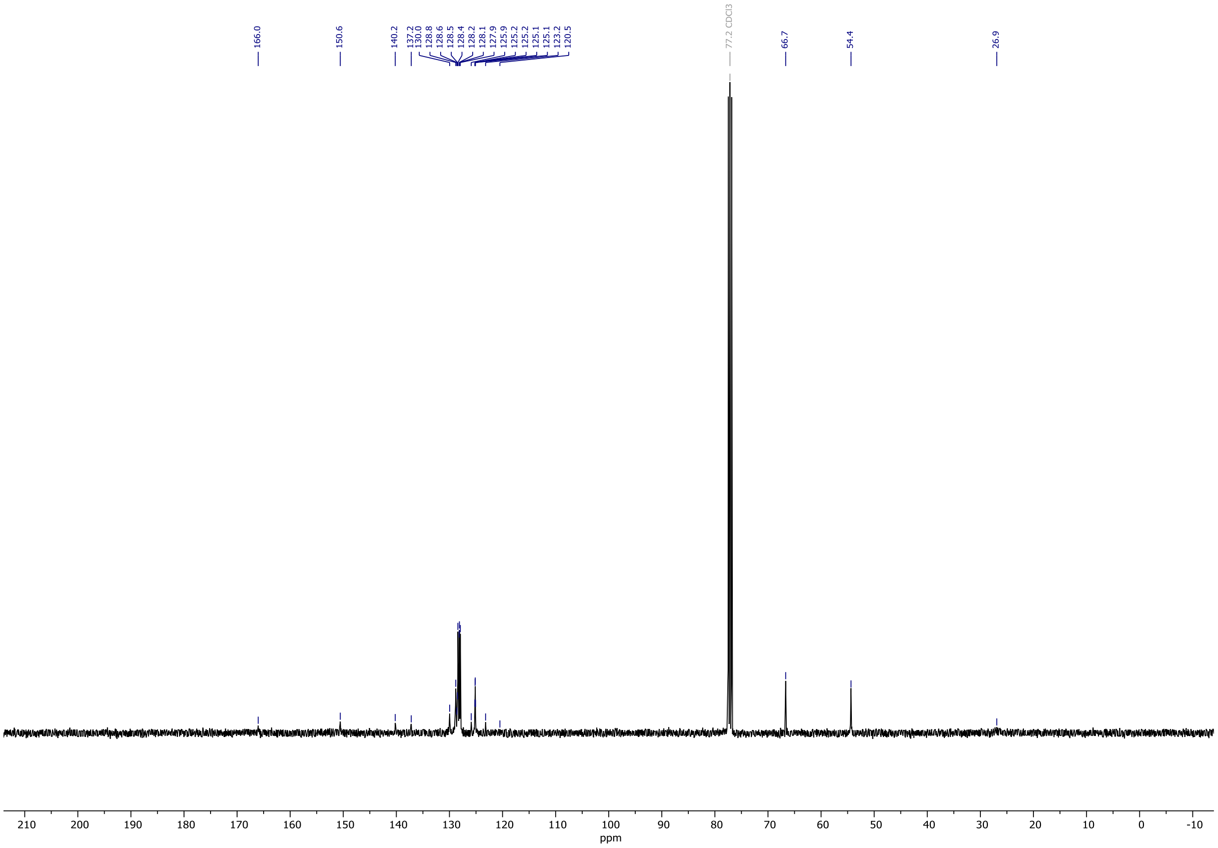

$^{11}B\{^1H\}$ NMR ($CDCl_3$, 128 MHz) for **5x**

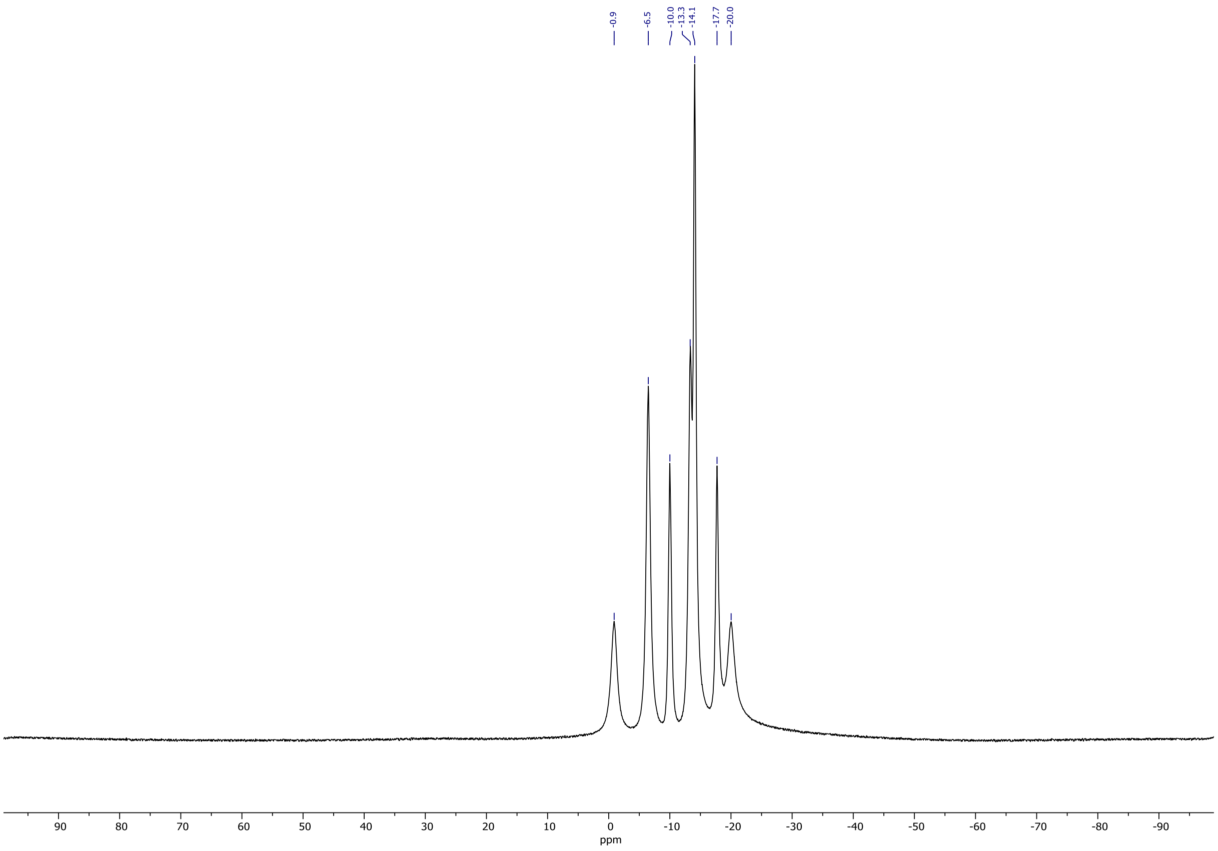

$^{19}F\{^1H\}$ NMR ($CDCl_3$, 377 MHz) for **5x**

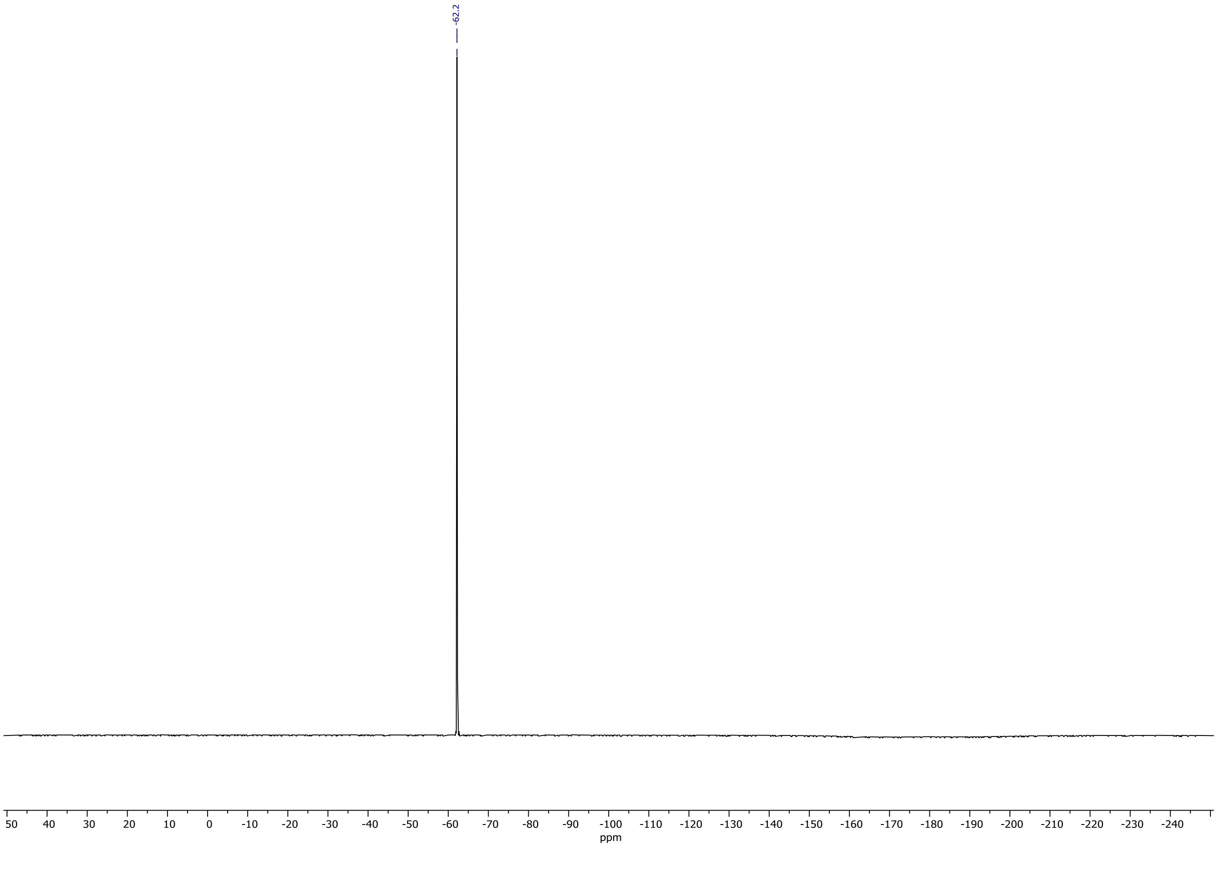

^{1}H NMR ($CDCl_3$, 400 MHz) for **5y**

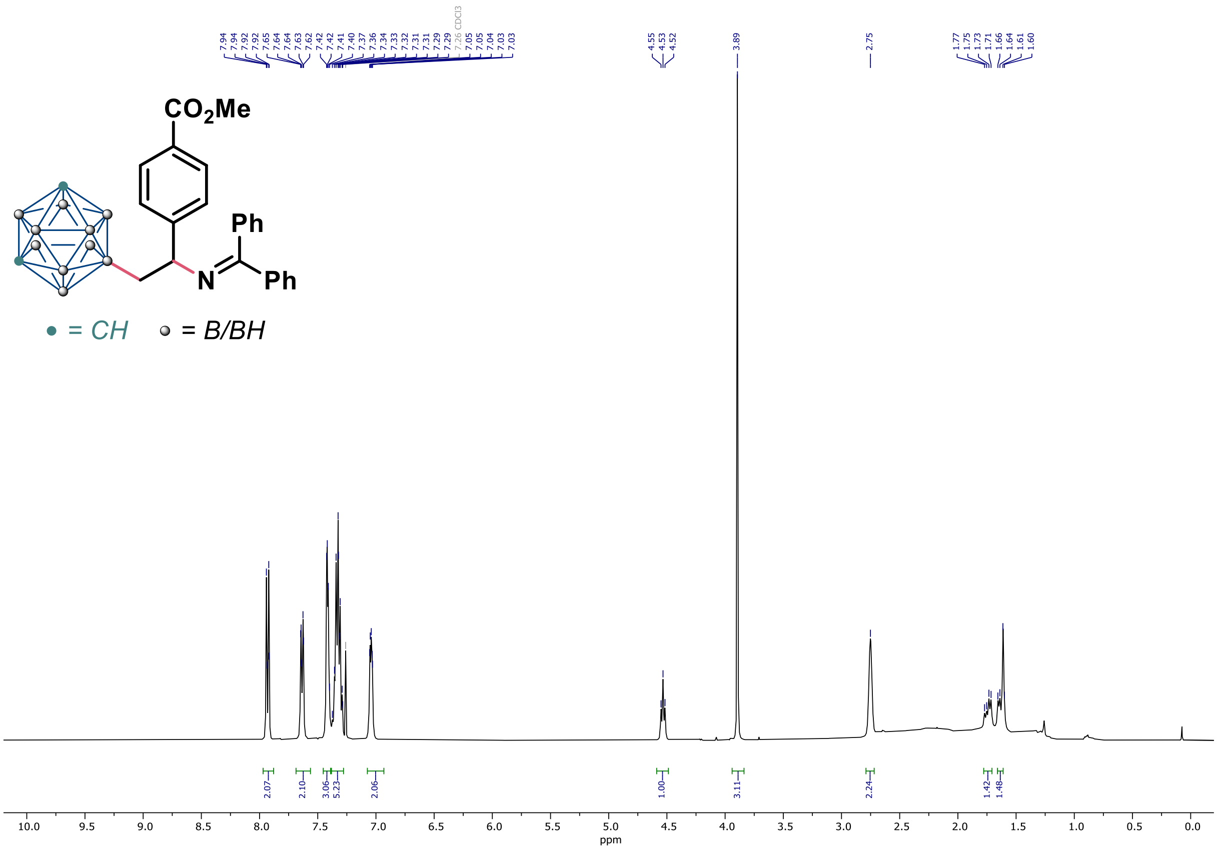

^{13}C{^{1}H} NMR ($CDCl_3$, 101 MHz) for **5y**

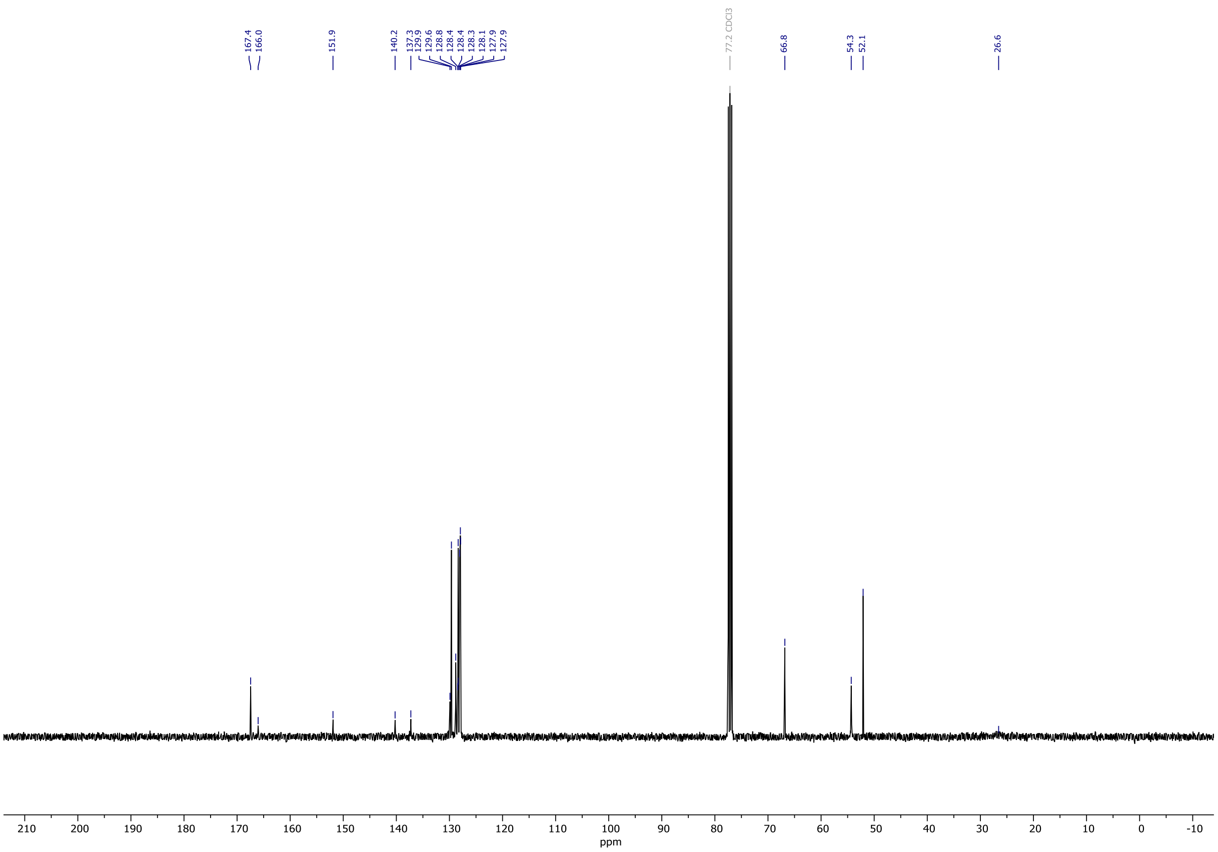

^{11}B{^{1}H} NMR (CDCl$_3$, 128 MHz) for **5y**

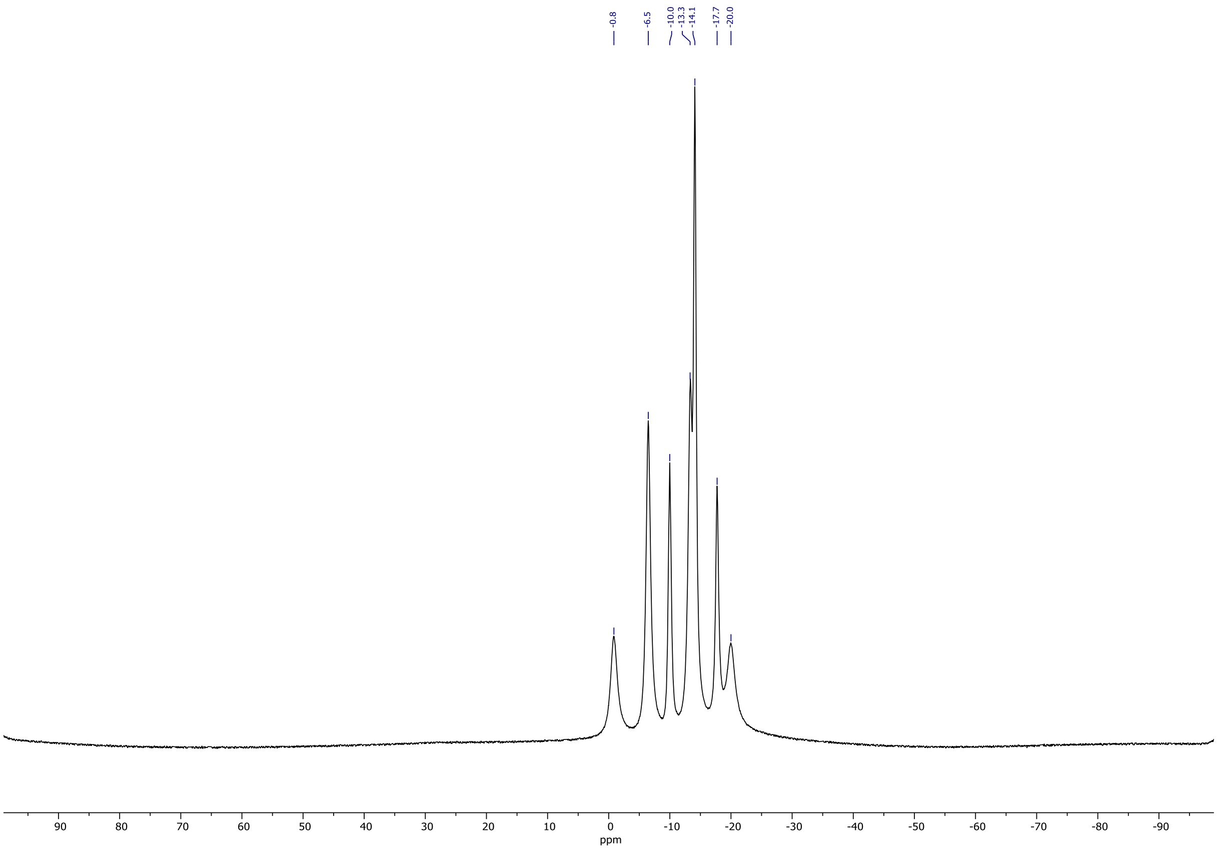

¹H NMR (CDCl$_3$, 400 MHz) for **5z**

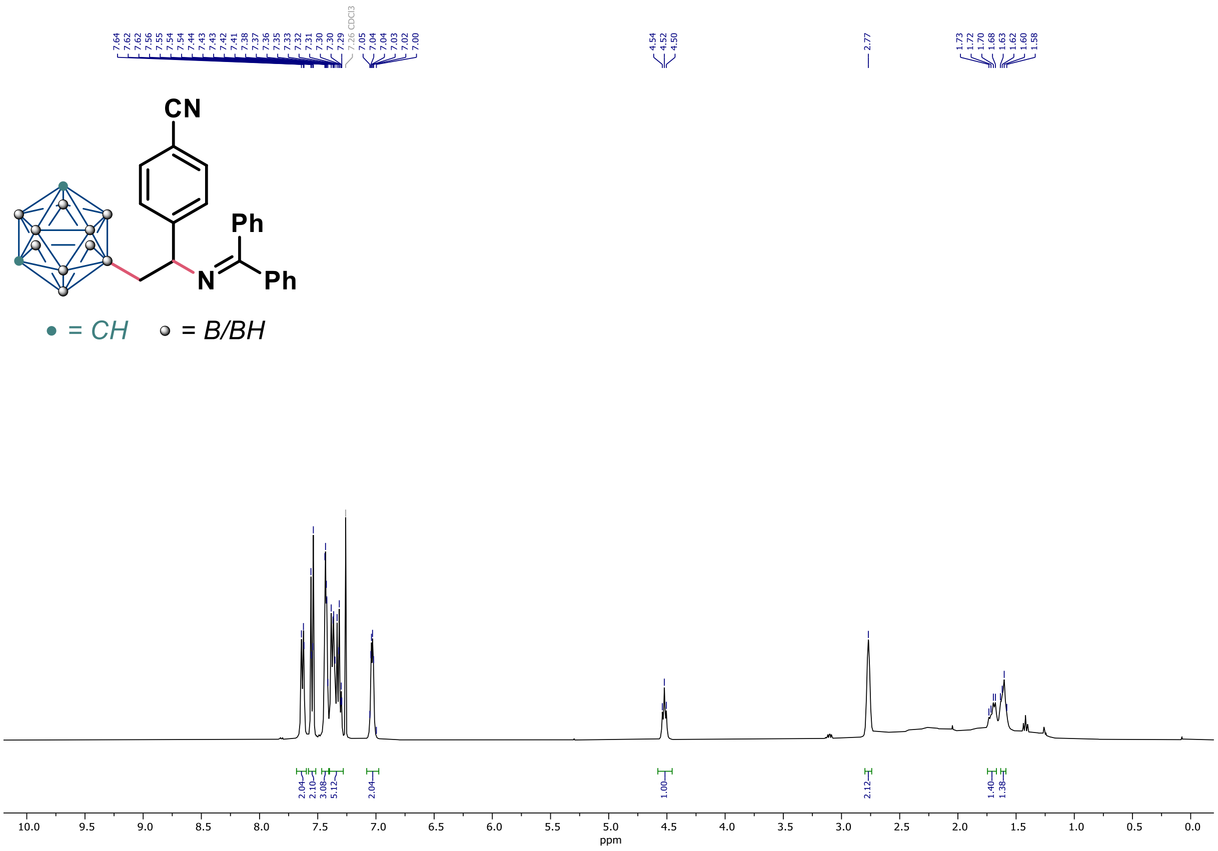

¹³C{¹H} NMR (CDCl$_3$, 101 MHz) for **5z**

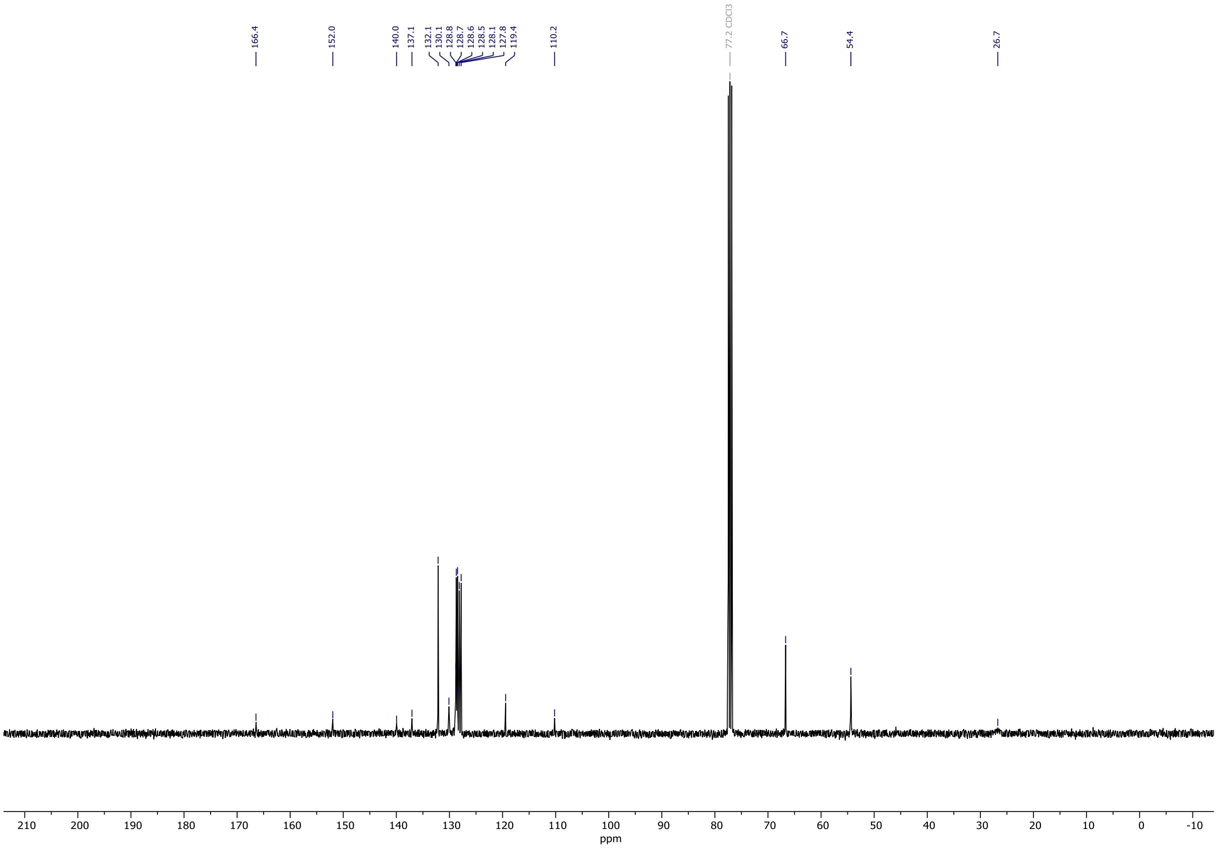

^{11}B{^{1}H} NMR (CDCl$_3$, 128 MHz) for **5z**

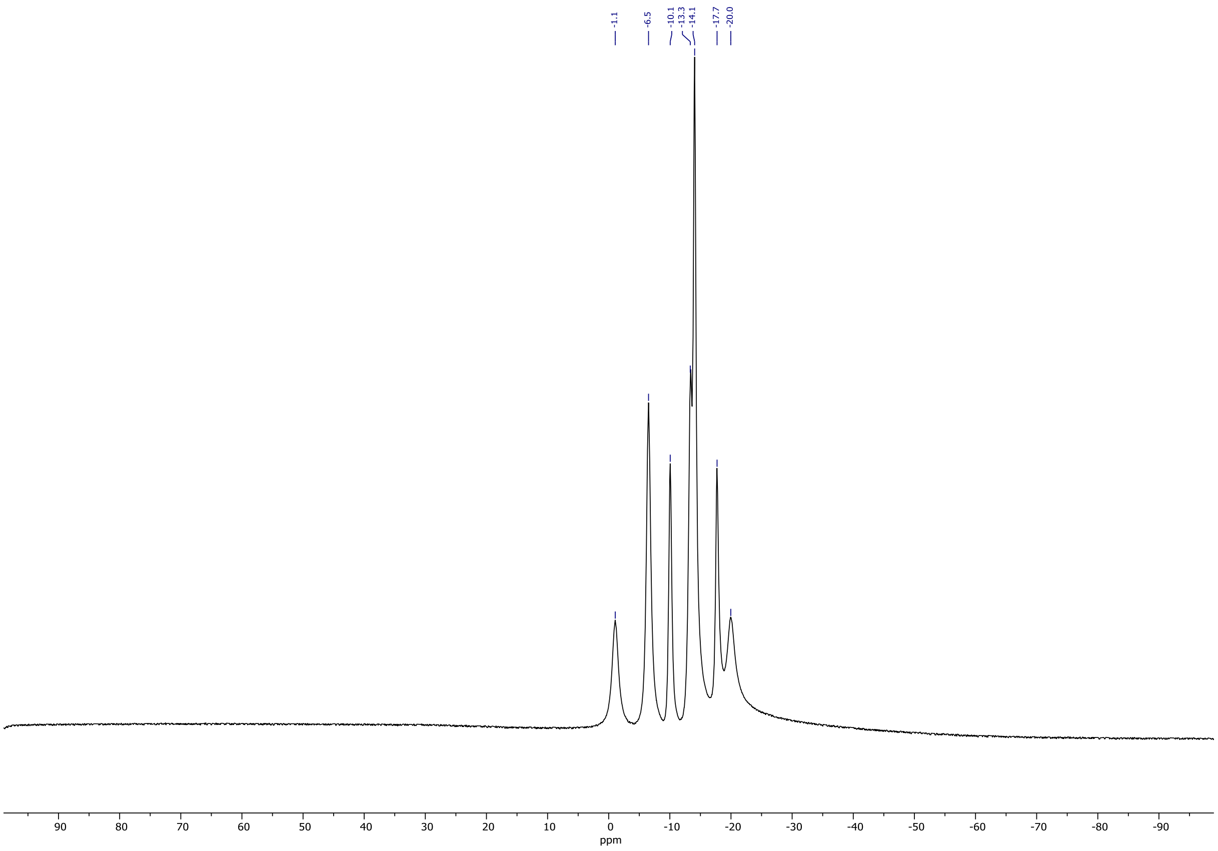

^{1}H NMR (CDCl$_3$, 400 MHz) for **5aa**

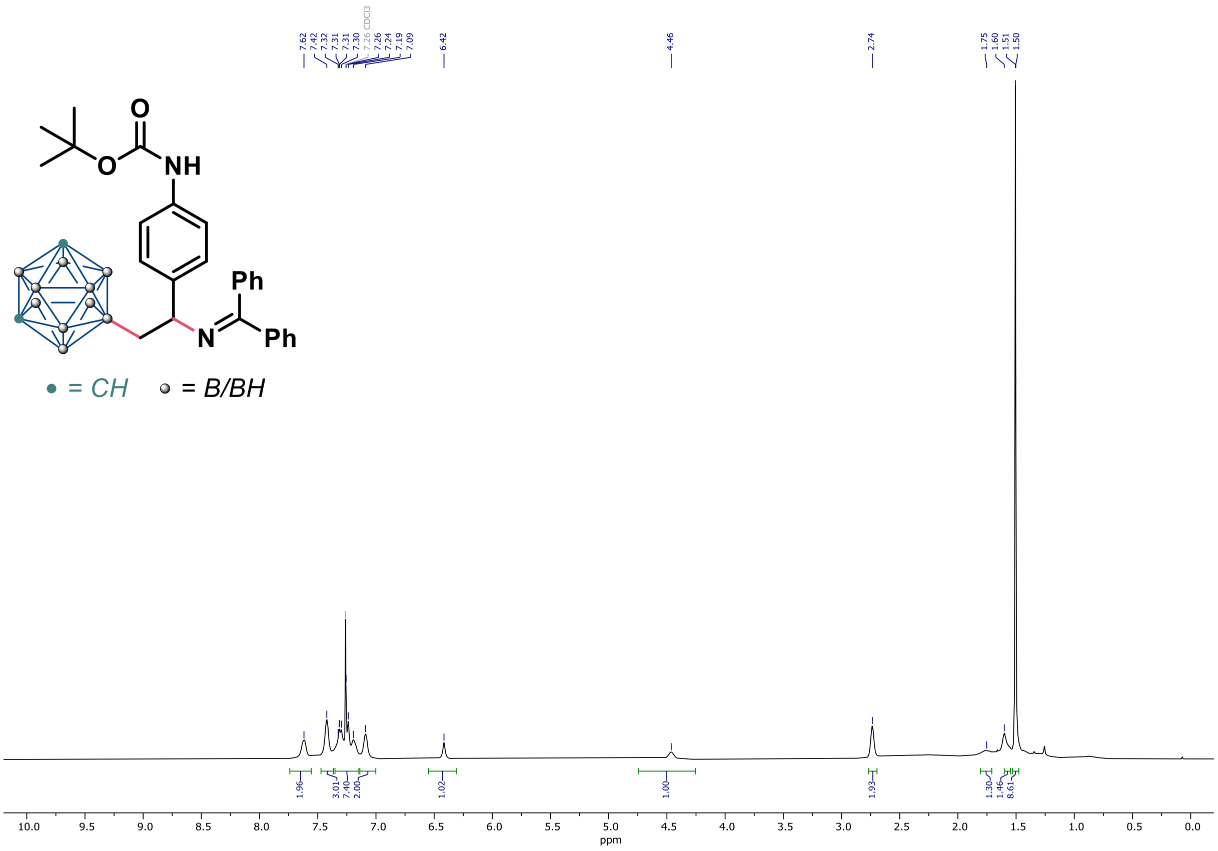

^{13}C{^{1}H} NMR (CDCl$_3$, 101 MHz) for **5aa**

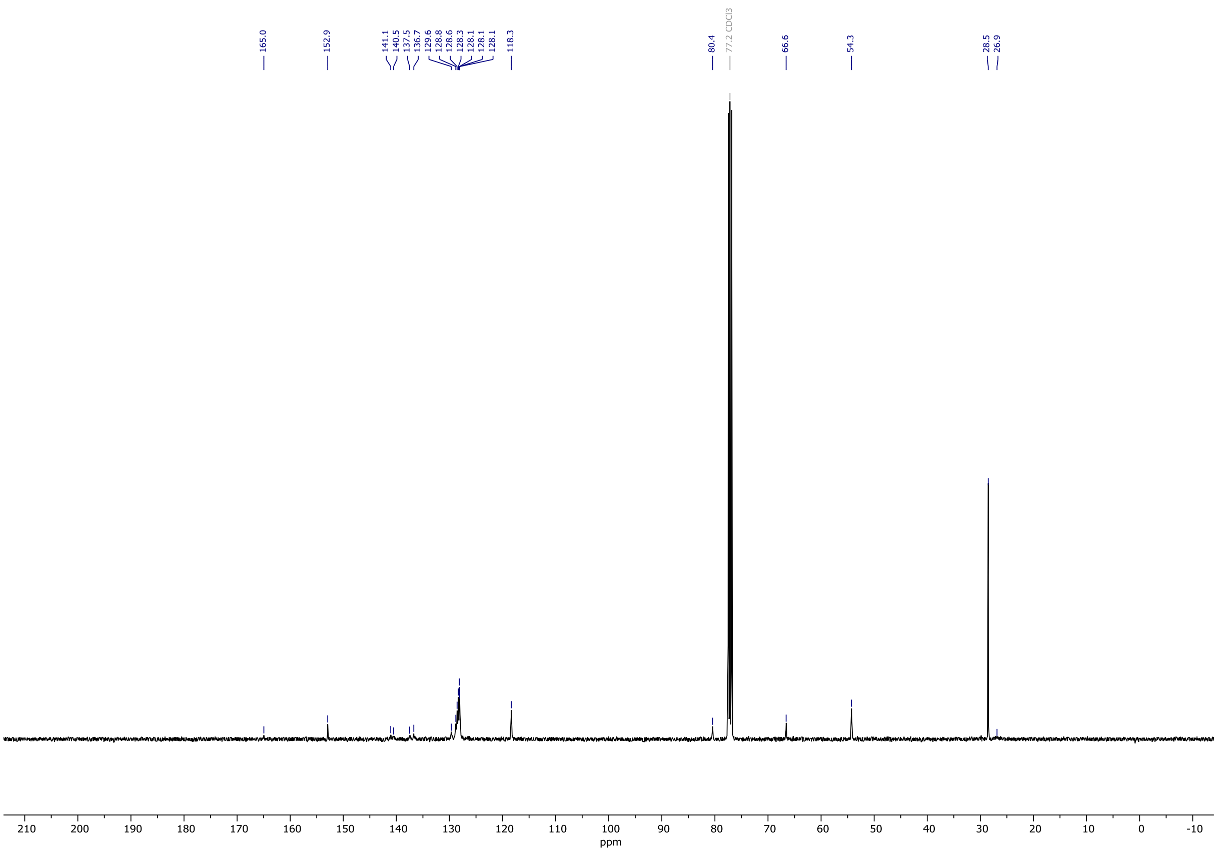

^{11}B{^{1}H} NMR (CDCl$_3$, 128 MHz) for **5aa**

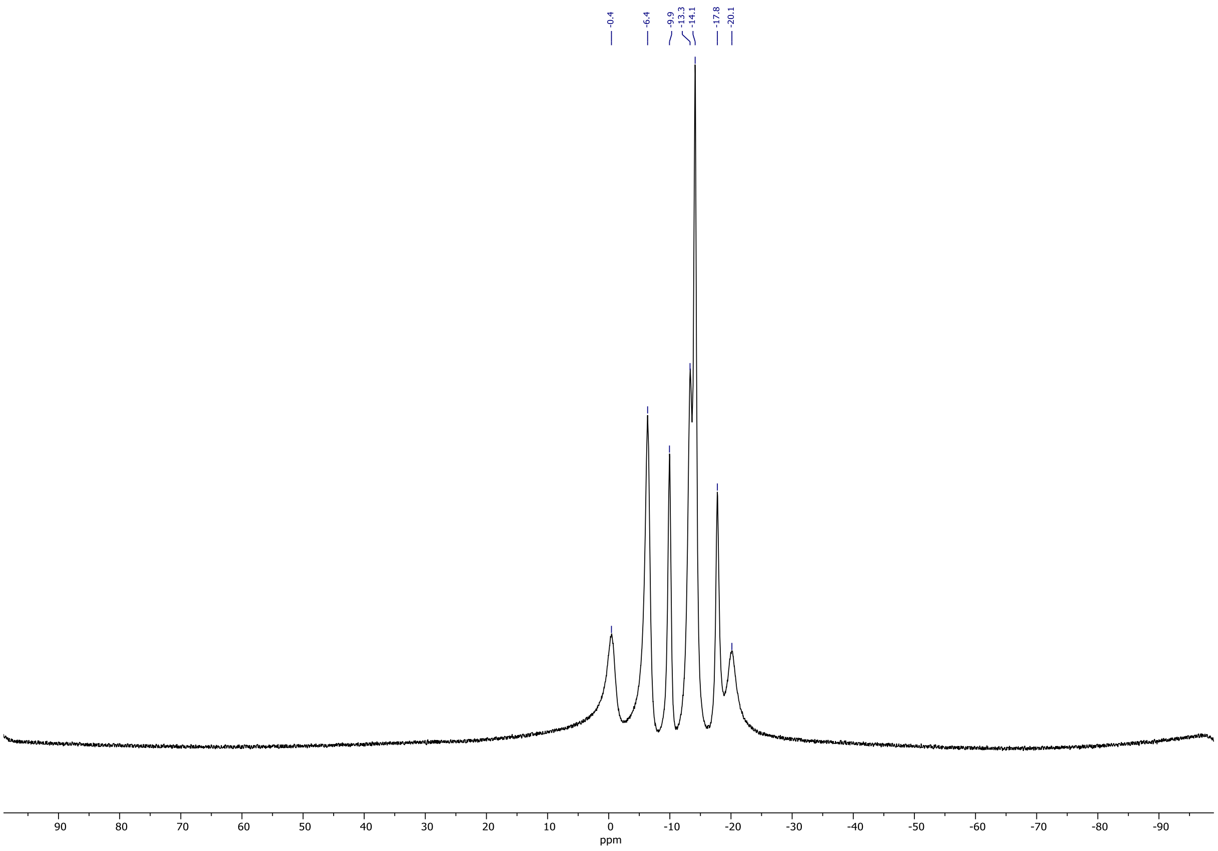

^{1}H NMR (CDCl$_3$, 400 MHz) for **5ab**

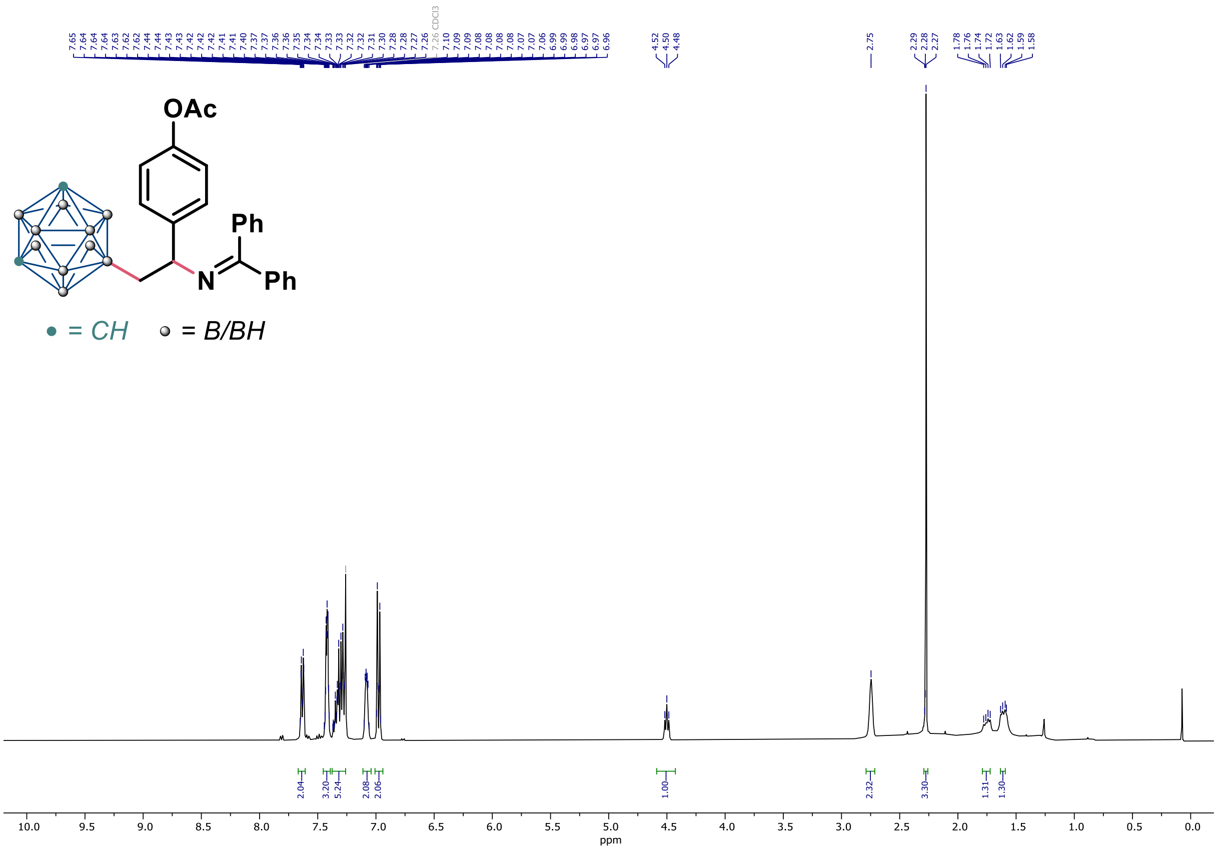

^{13}C{^{1}H} NMR (CDCl$_3$, 101 MHz) for **5ab**

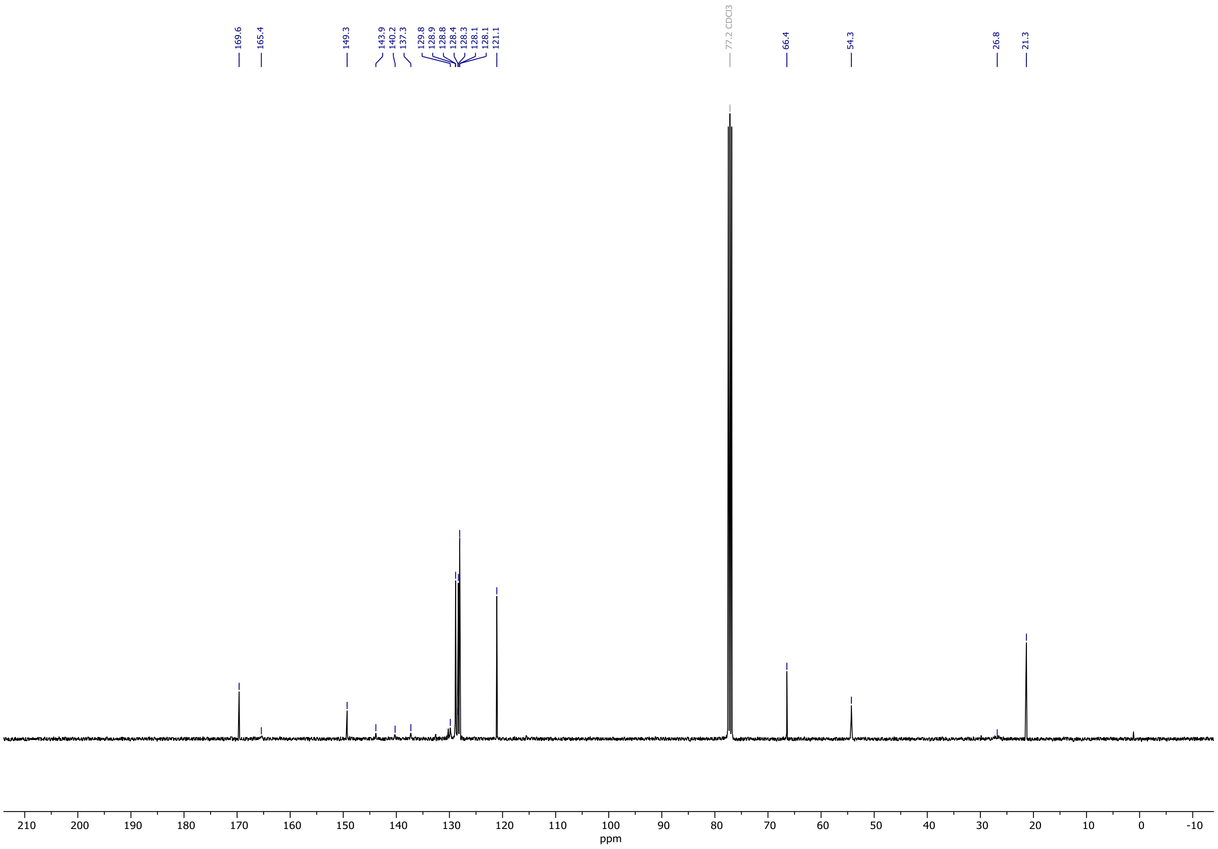

$^{11}B\{^{1}H\}$ NMR ($CDCl_3$, 128 MHz) for **5ab**

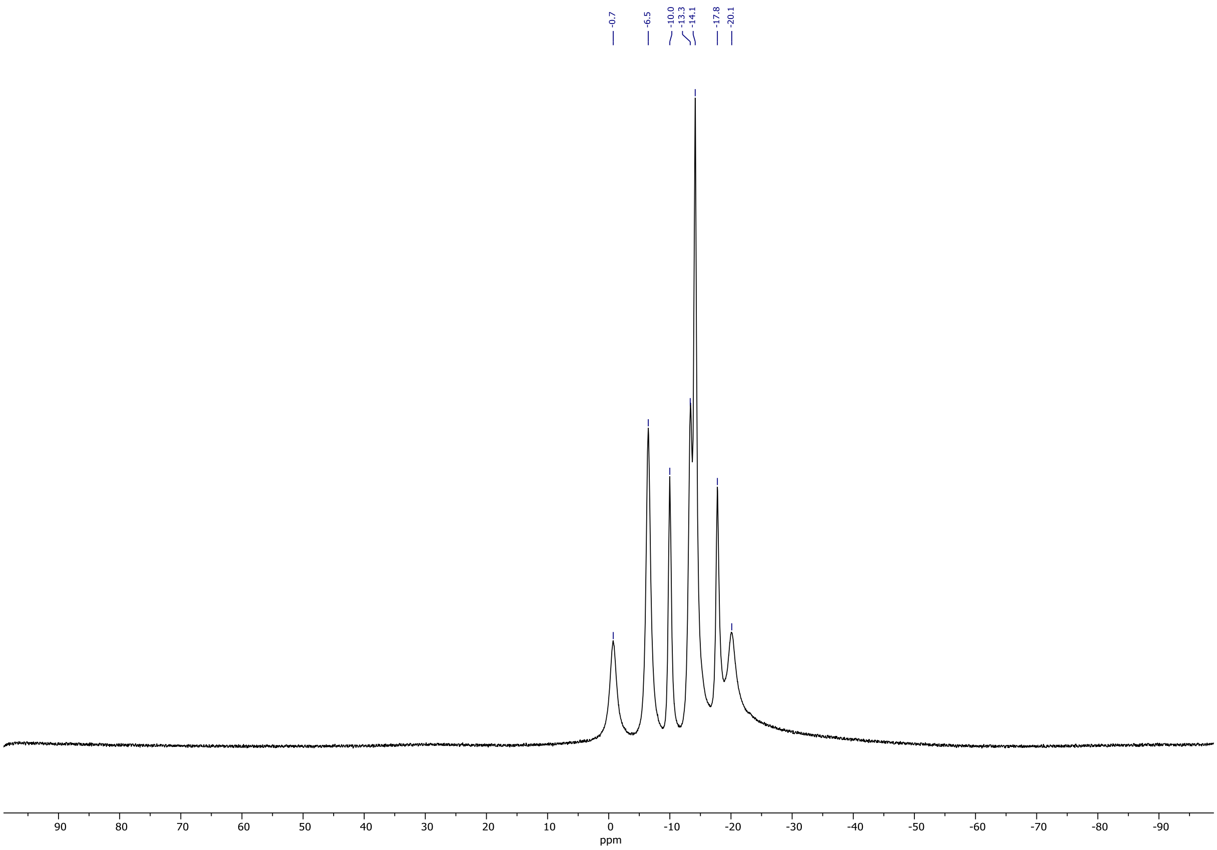

^{1}H NMR ($CDCl_3$, 400 MHz) for **5ac**

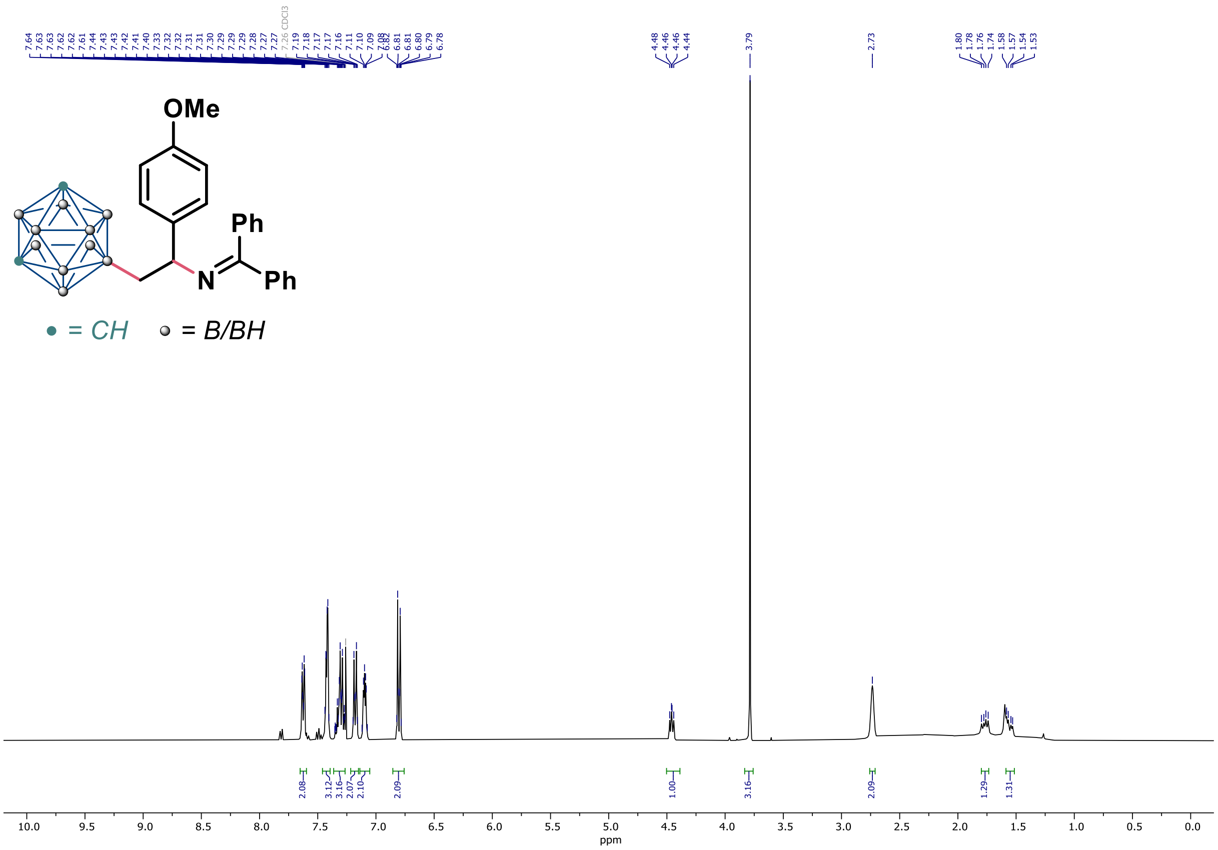

^{13}C{^{1}H} NMR ($CDCl_3$, 101 MHz) for **5ac**

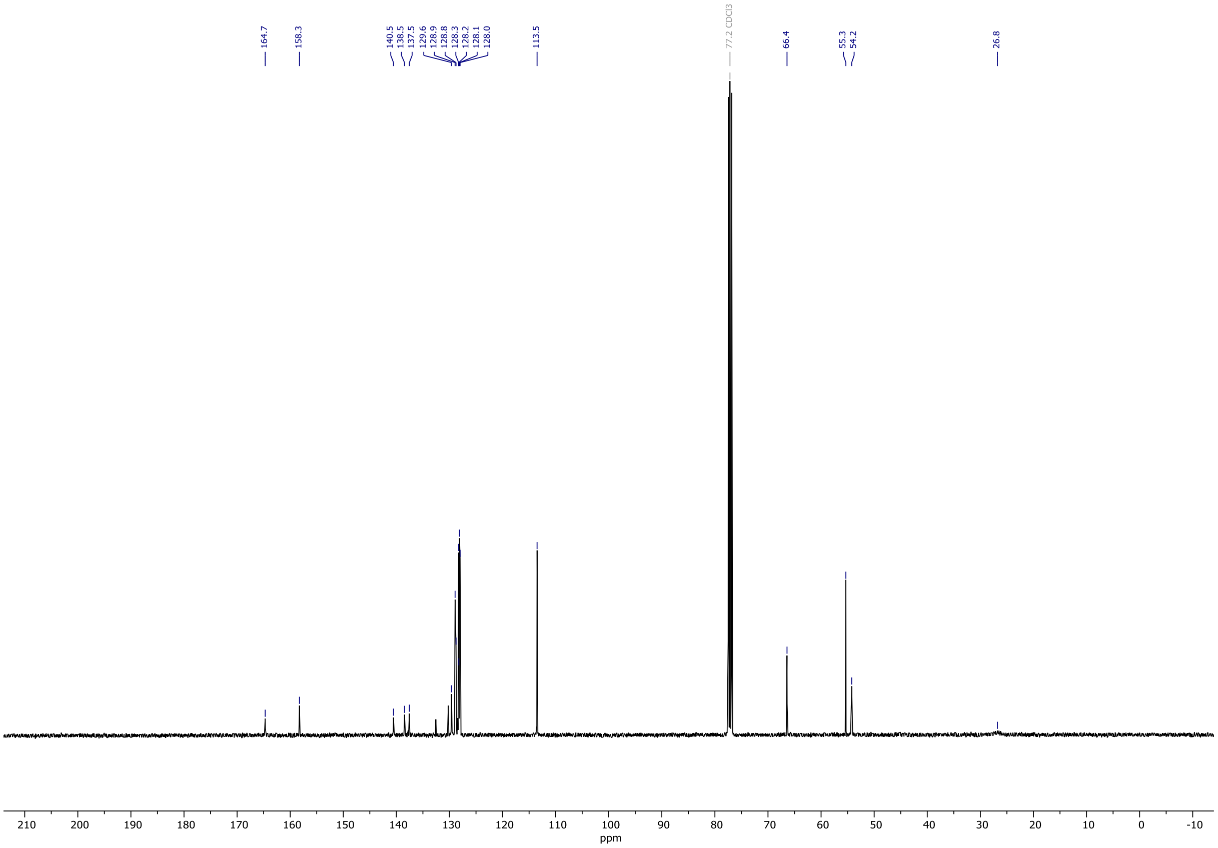

^{11}B{^{1}H} NMR (CDCl$_3$, 128 MHz) for **5ac**

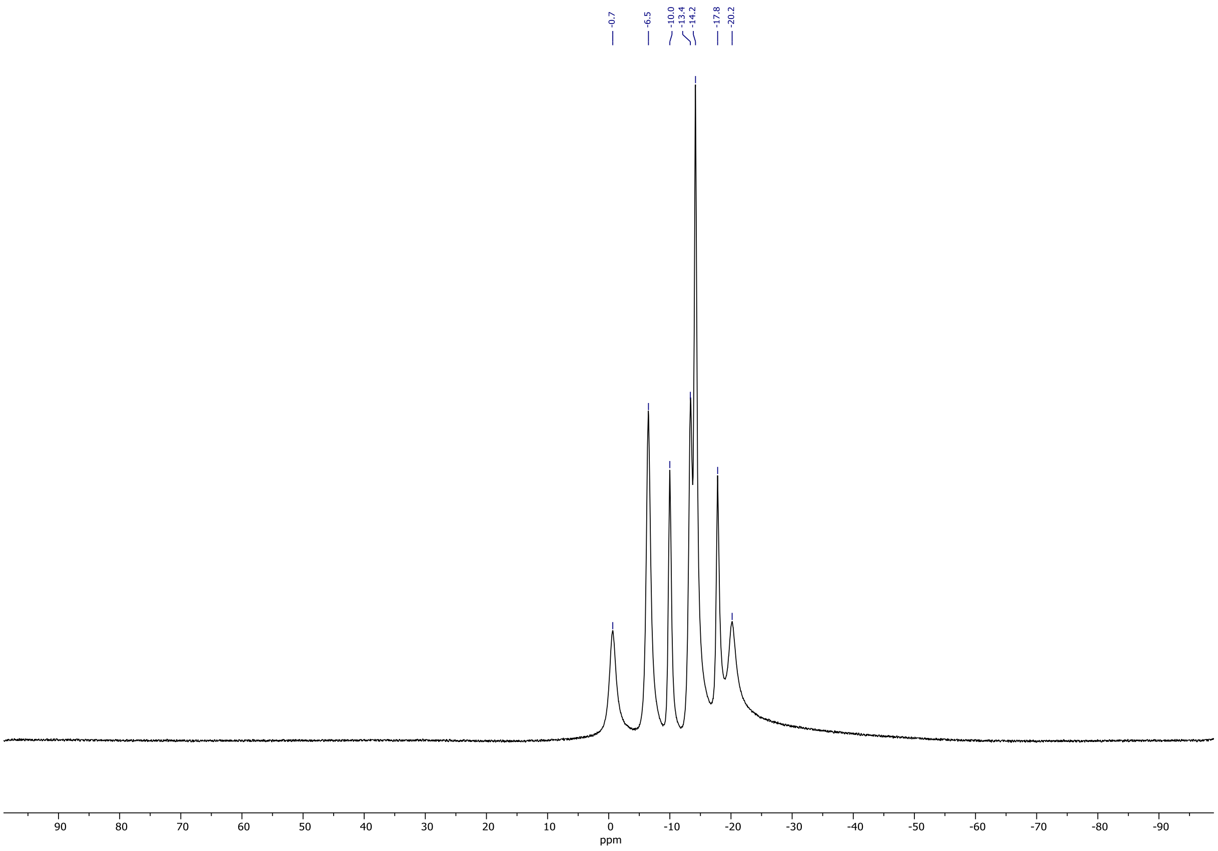

^{1}H NMR (CDCl$_3$, 400 MHz) for **5ac'**

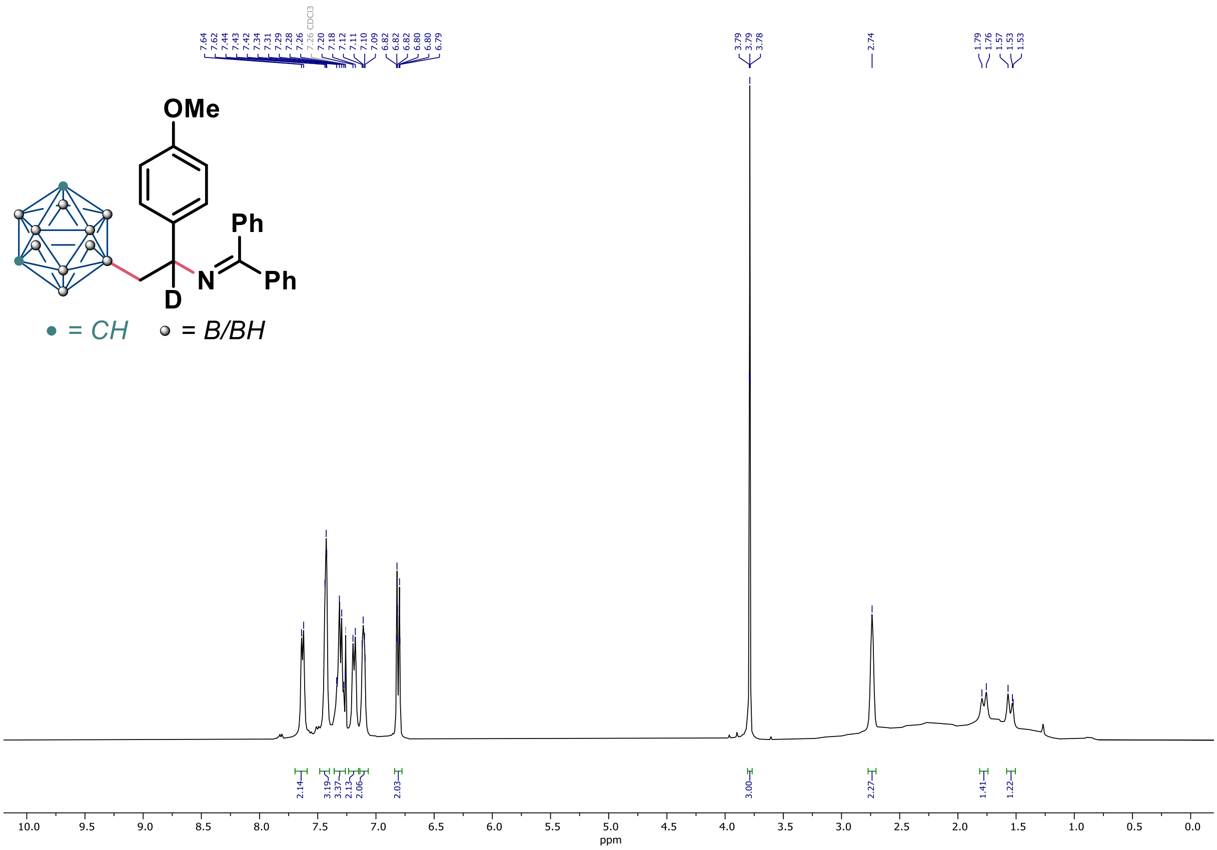

^{13}C{^{1}H} NMR (CDCl$_3$, 101 MHz) for **5ac'**

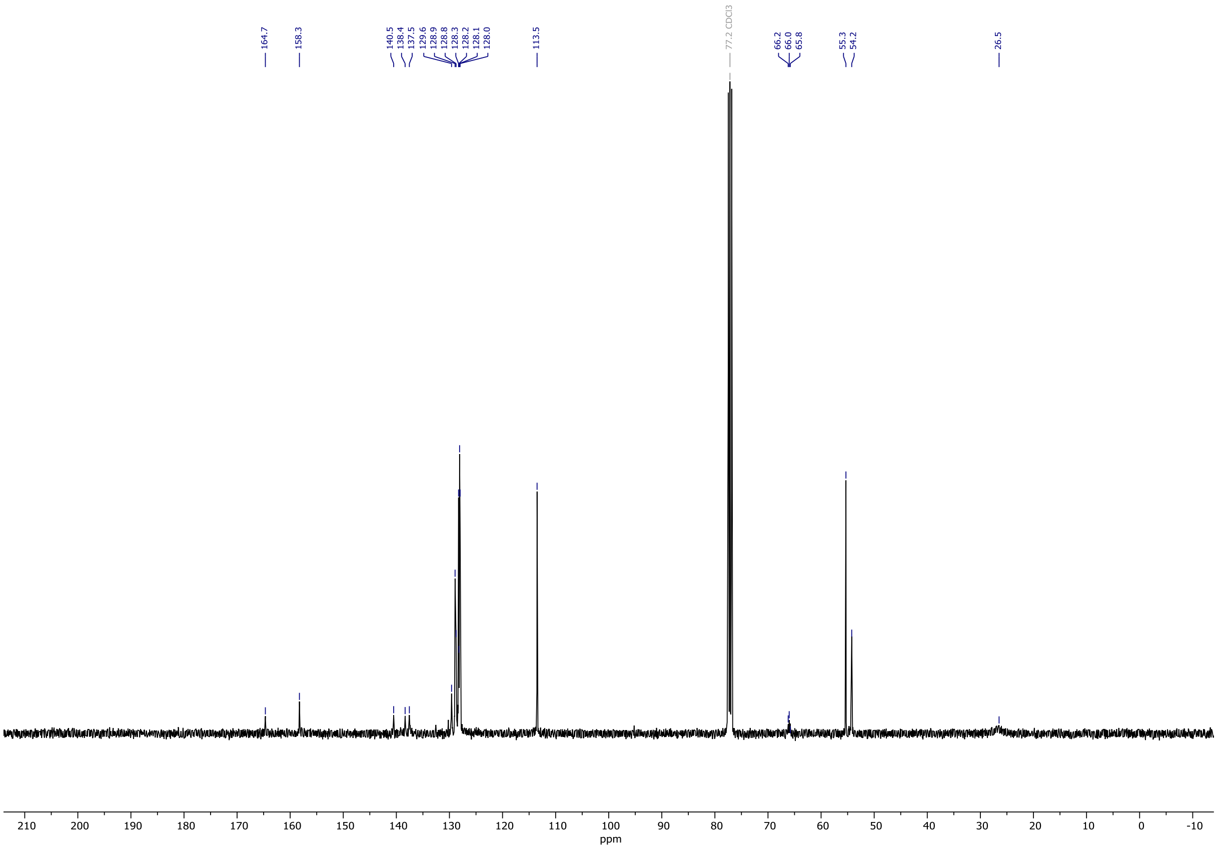

^{11}B{^{1}H} NMR (CDCl$_3$, 128 MHz) for **5ac'**

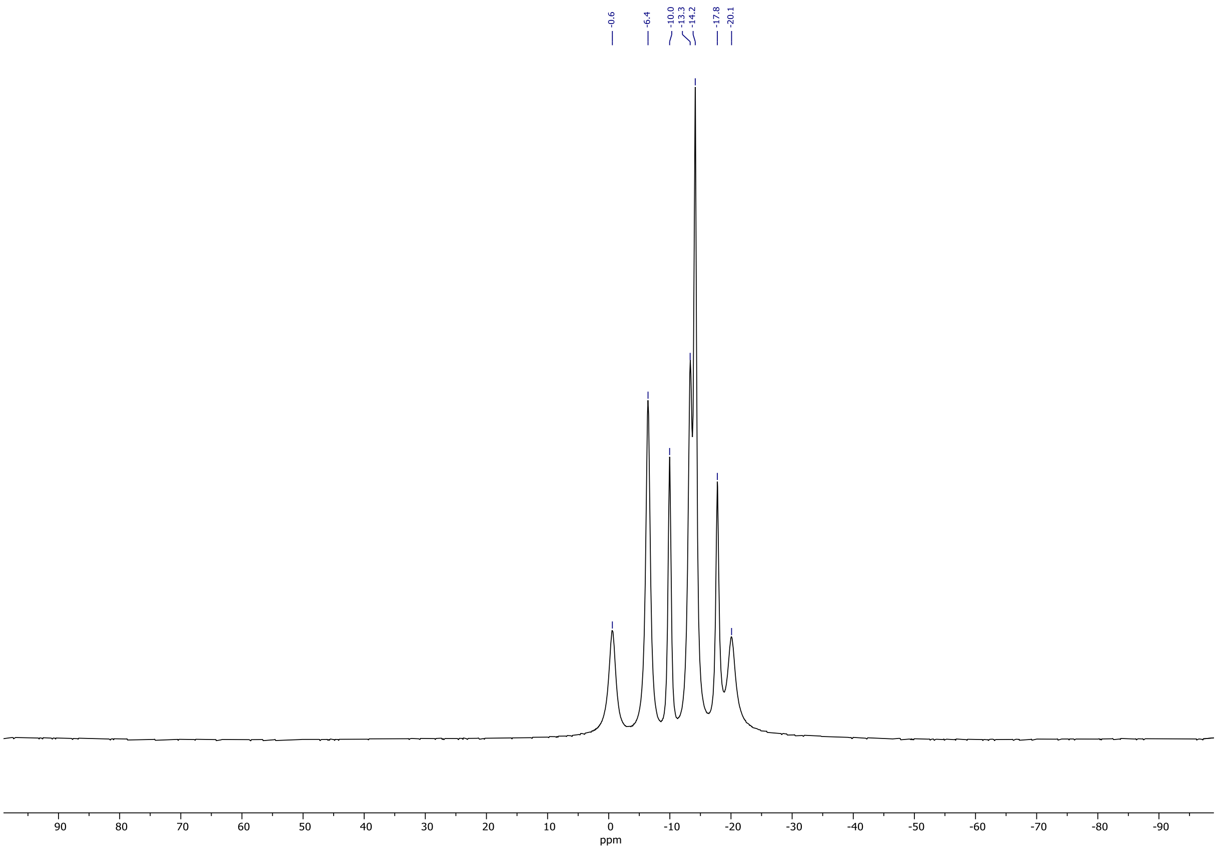

^{1}H NMR ($CDCl_3$, 400 MHz) for **5ac''**

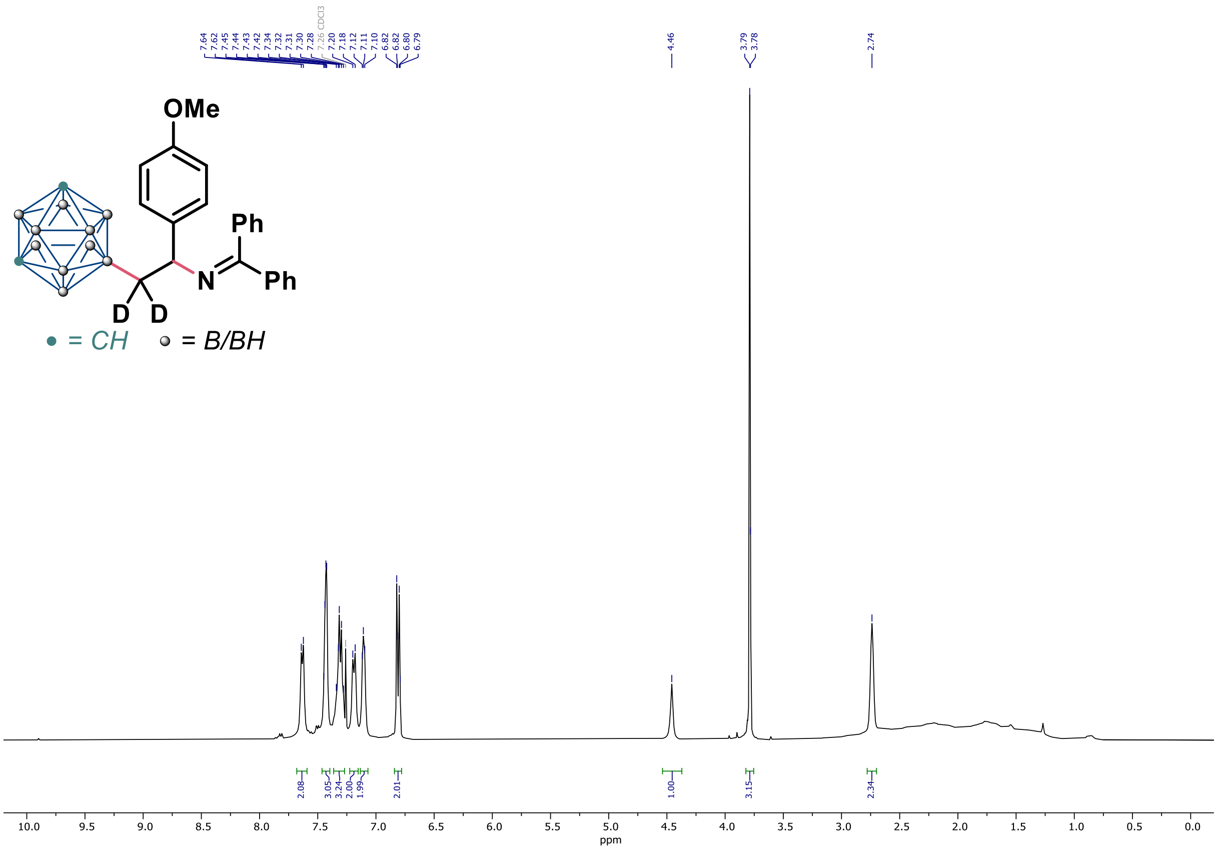

^{13}C{^{1}H} NMR ($CDCl_3$, 101 MHz) for **5ac''**

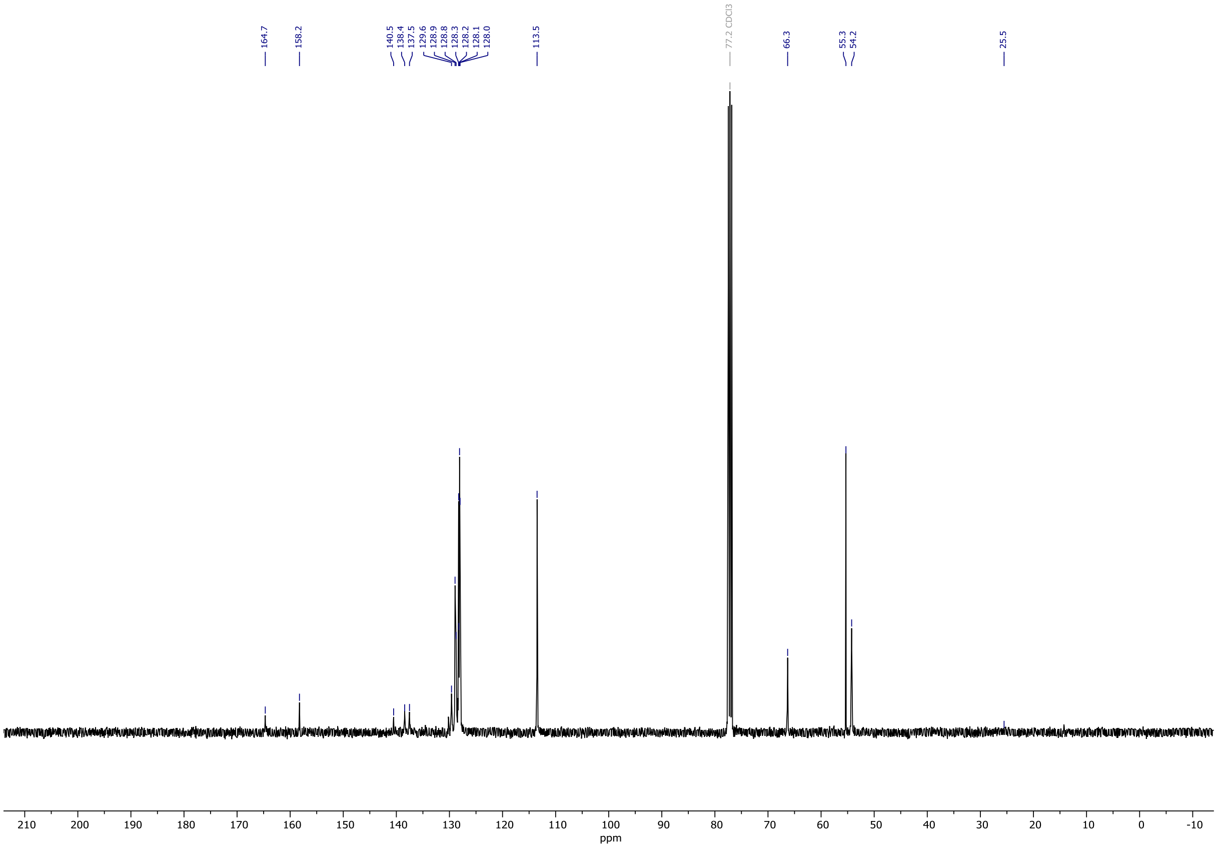

$^{11}B\{^1H\}$ NMR (CDCl$_3$, 128 MHz) for **5ac''**

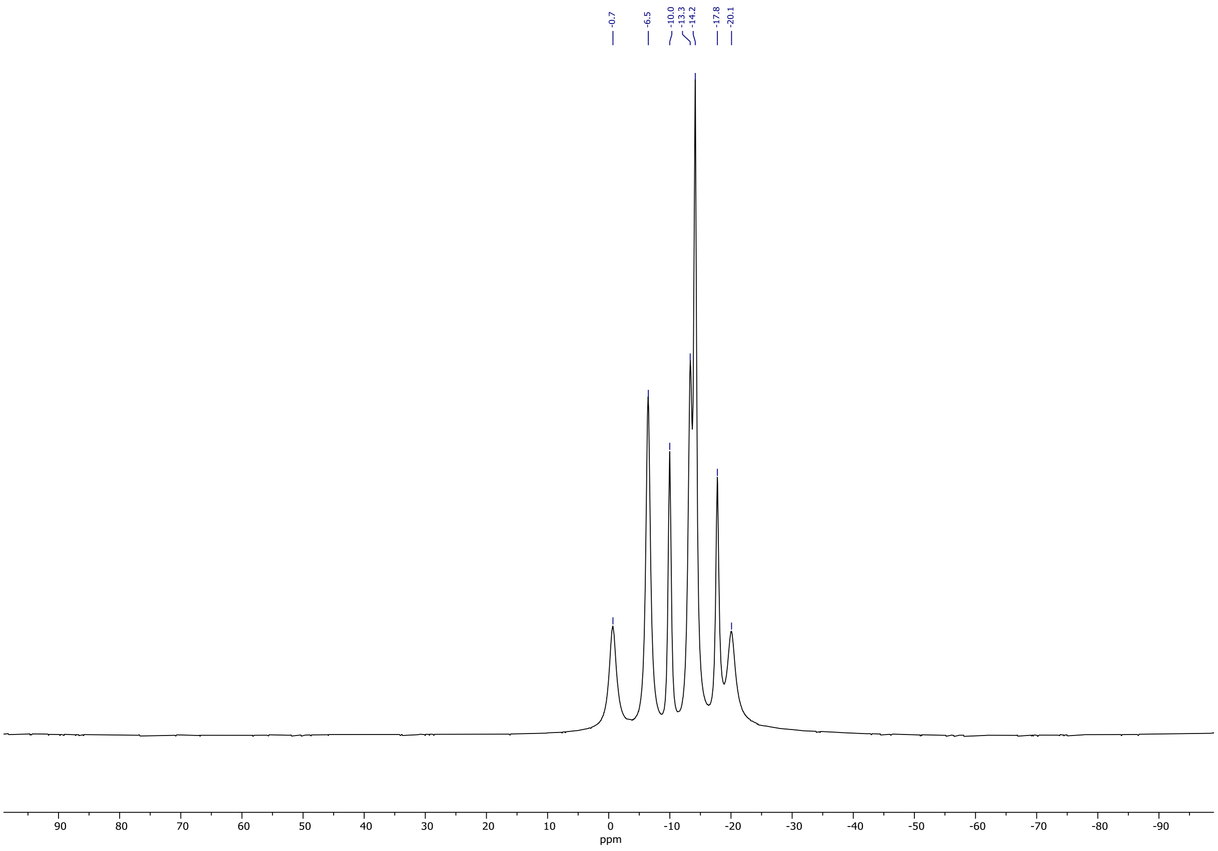

^{1}H NMR ($CDCl_3$, 400 MHz) for **5ad**

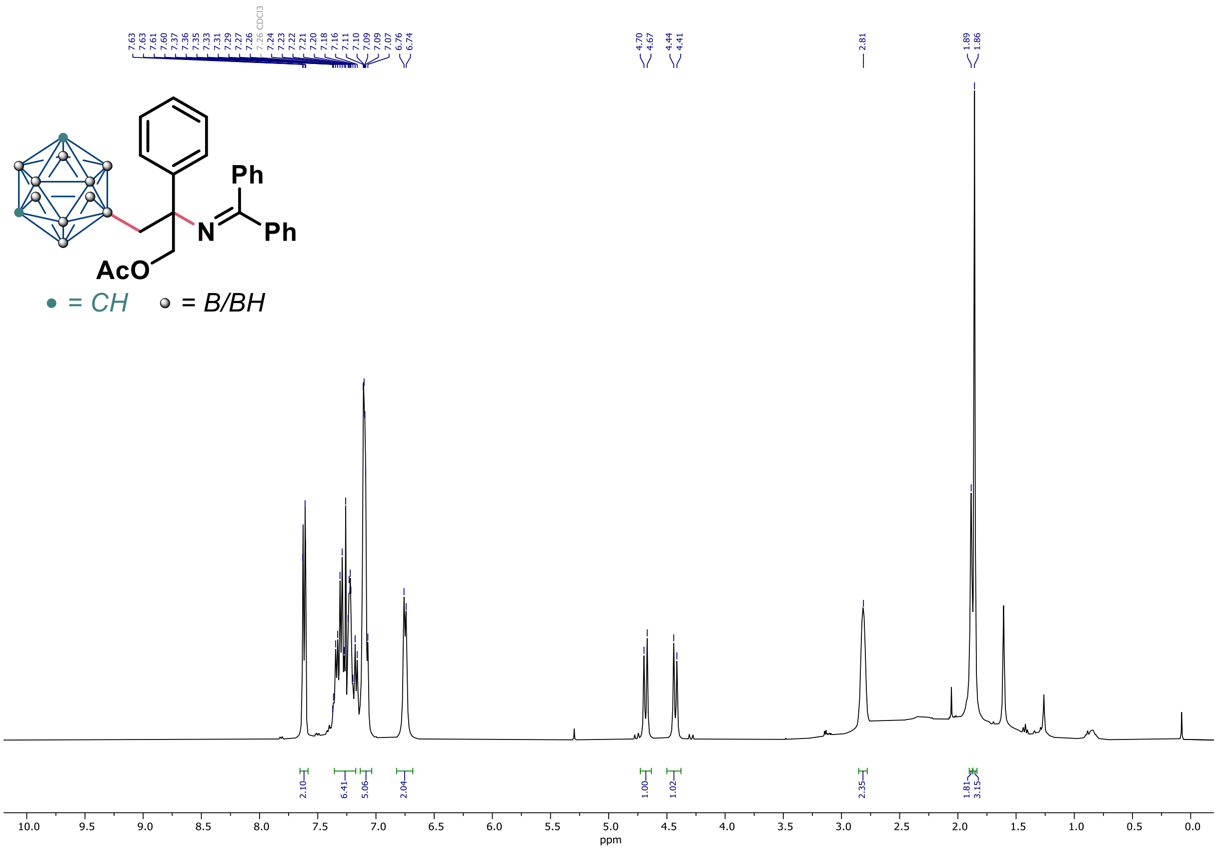

^{13}C{^{1}H} NMR ($CDCl_3$, 101 MHz) for **5ad**

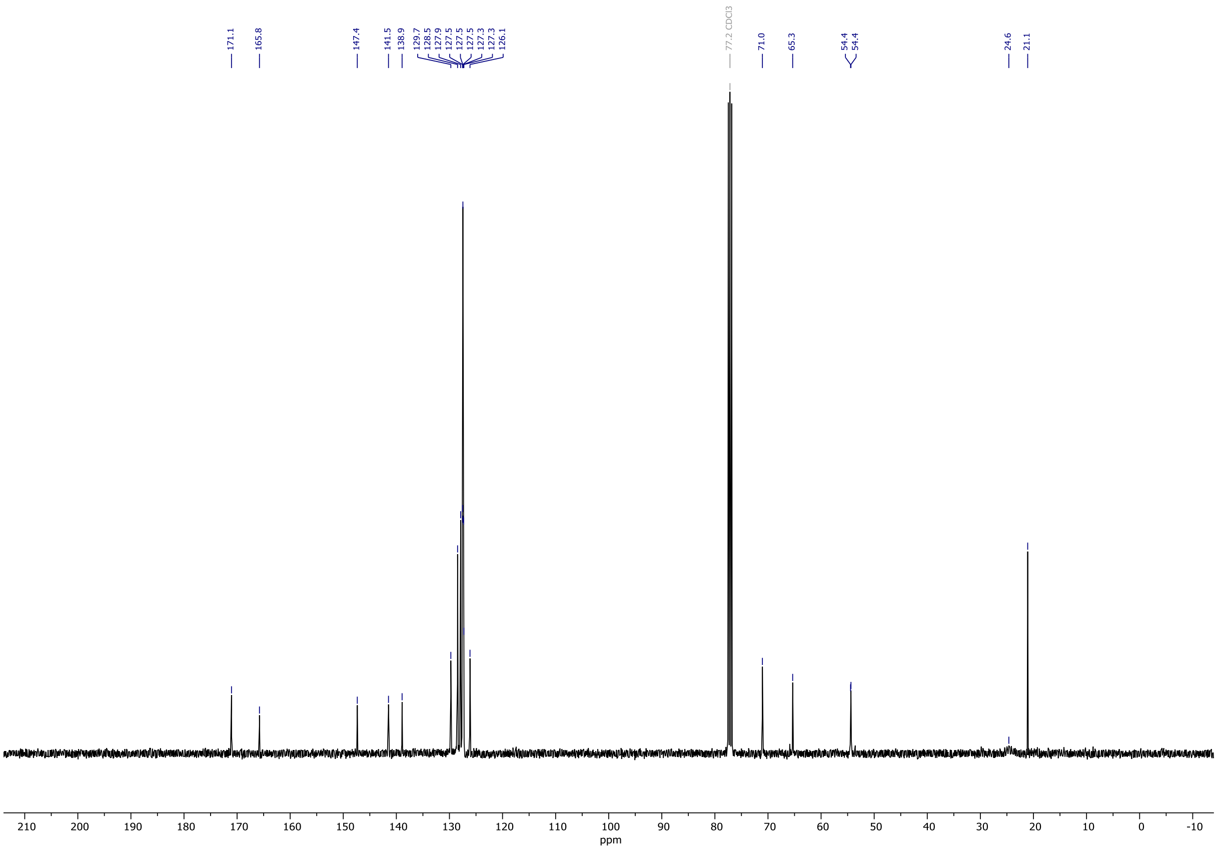

$^{11}B\{^{1}H\}$ NMR (CDCl$_3$, 128 MHz) for **5ad**

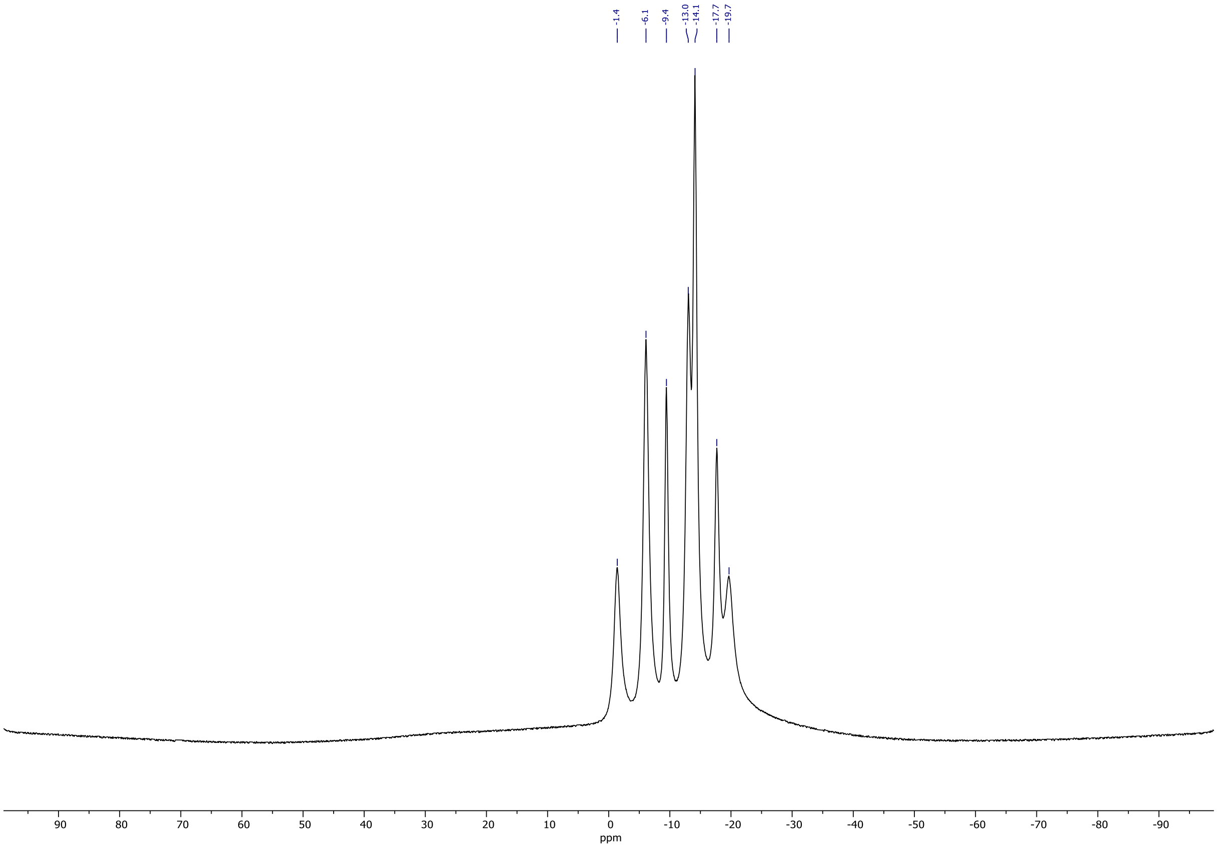

^{1}**H NMR** ($CDCl_3$, 400 MHz) for **5ae**

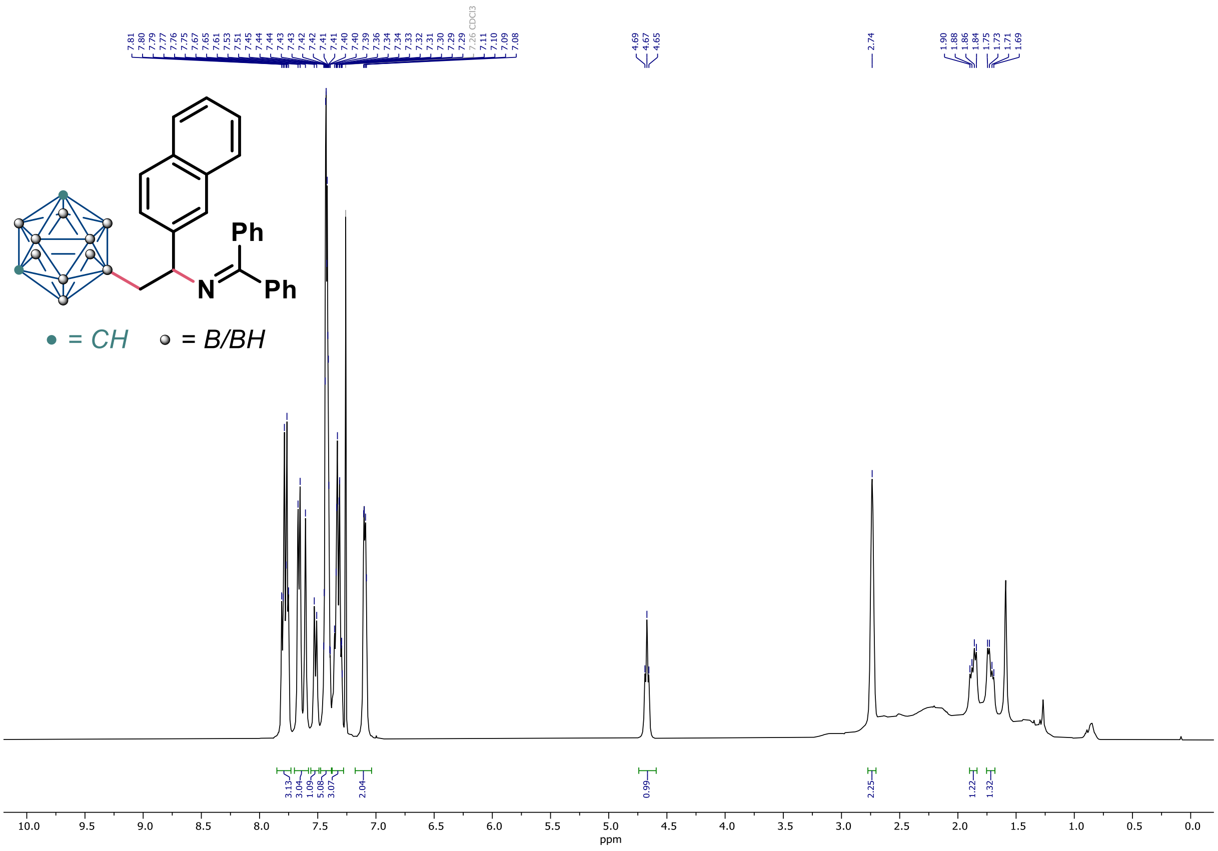

^{13}**C{^{1}H} NMR** ($CDCl_3$, 101 MHz) for **5ae**

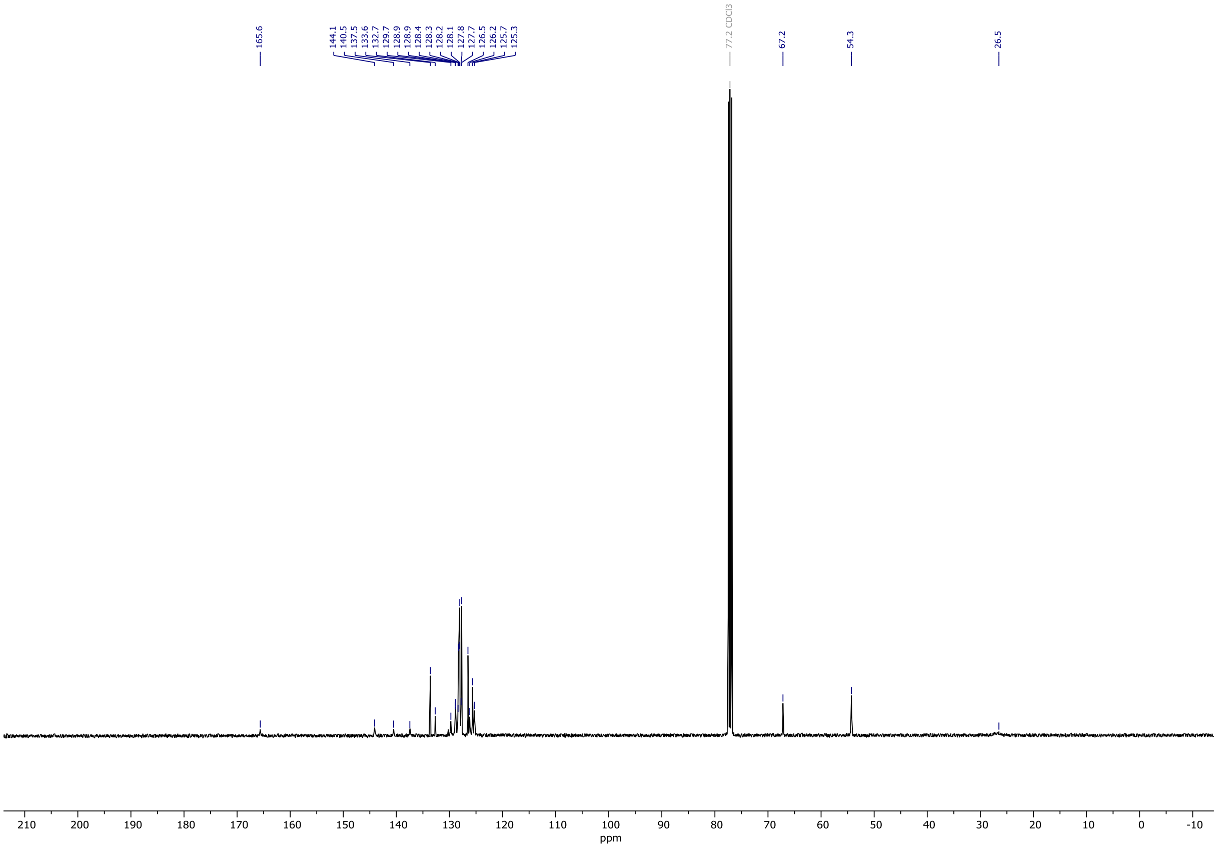

^{11}B{^{1}H} NMR (CDCl$_3$, 128 MHz) for **5ae**

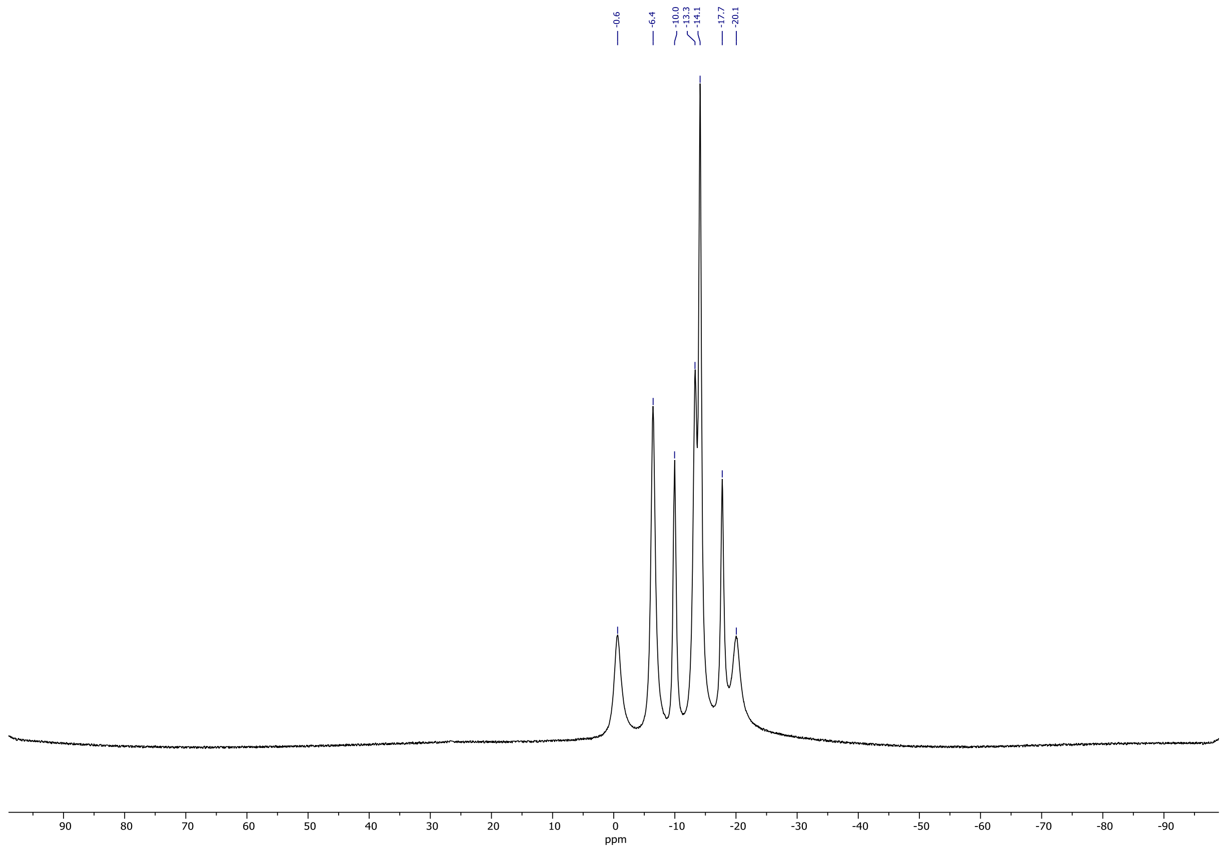

¹H NMR (CDCl$_3$, 400 MHz) for **5af**

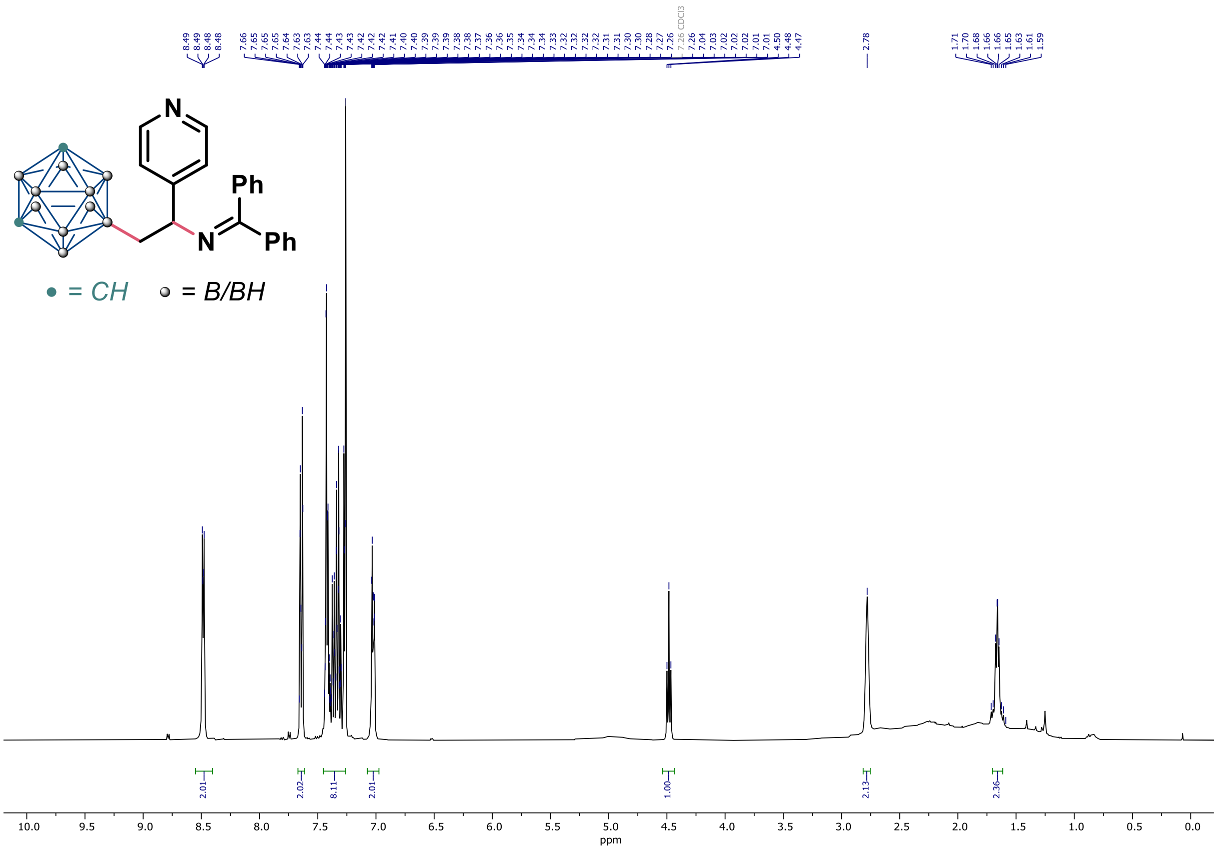

¹³C{¹H} NMR (CDCl$_3$, 101 MHz) for **5af**

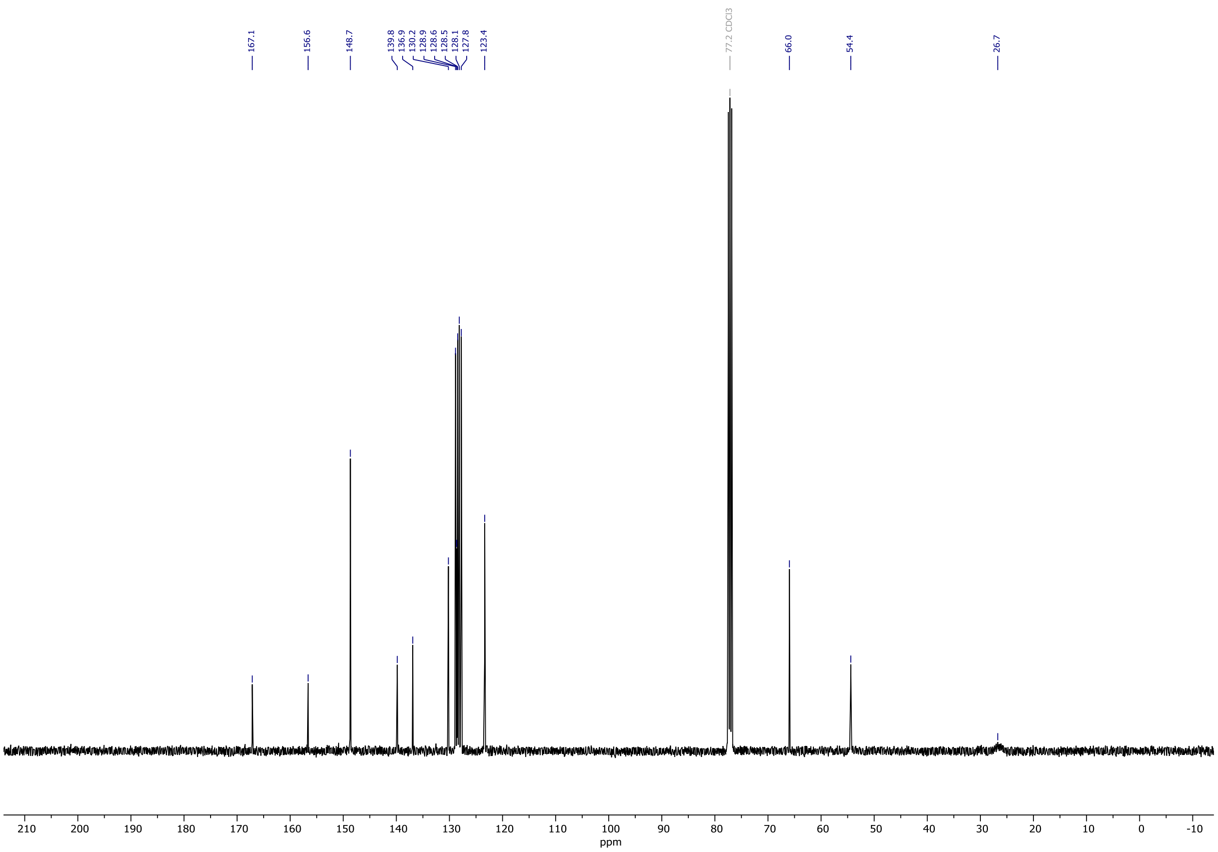

^{11}B{^{1}H} NMR (CDCl$_3$, 128 MHz) for **5af**

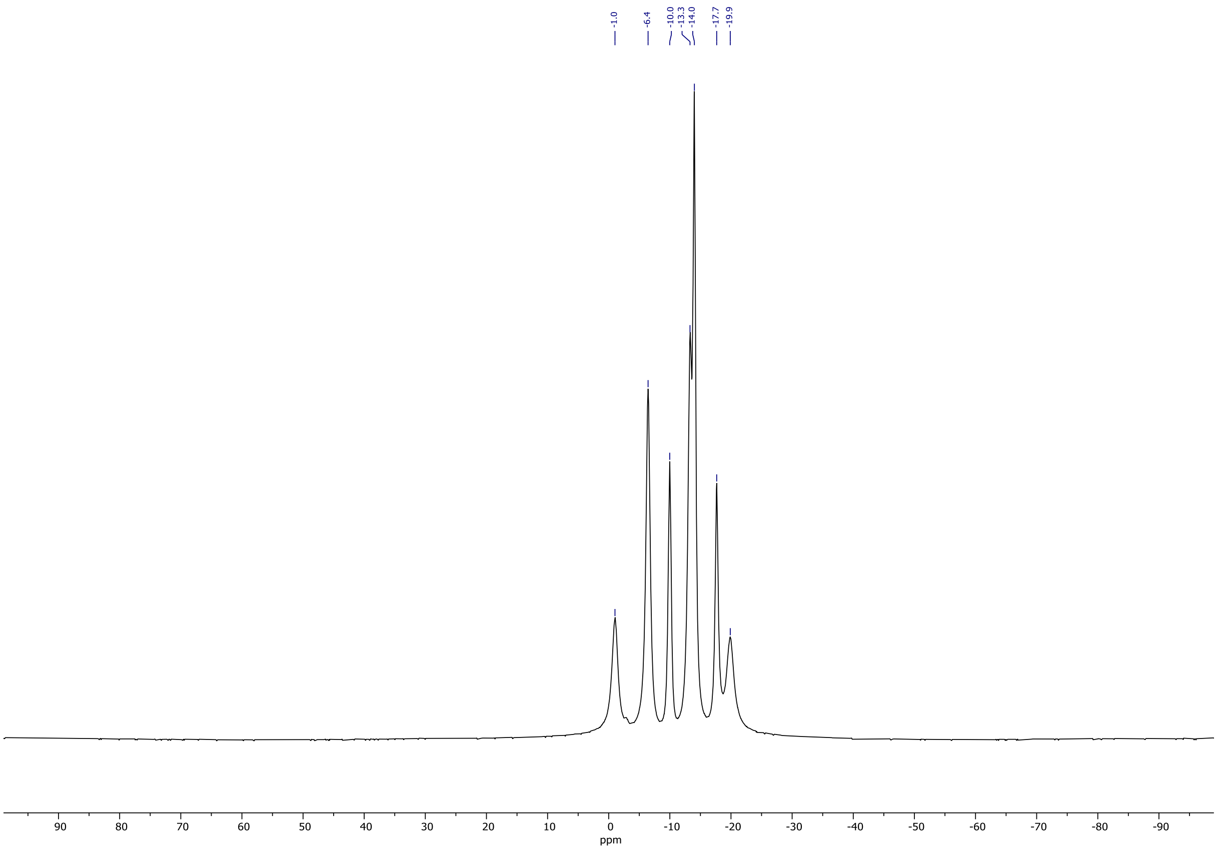

^{1}H NMR ($CDCl_3$, 400 MHz) for **5ag**

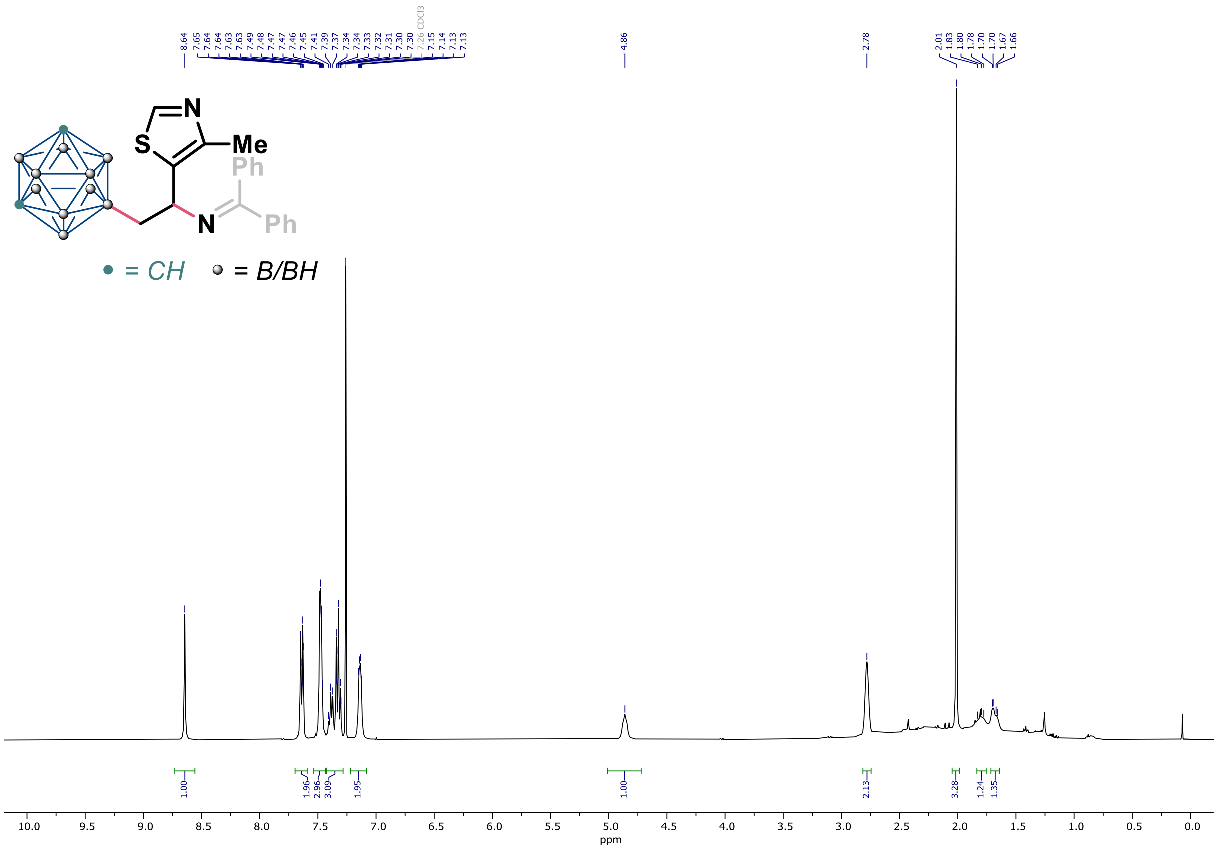

^{13}C{^{1}H} NMR ($CDCl_3$, 101 MHz) for **5ag**

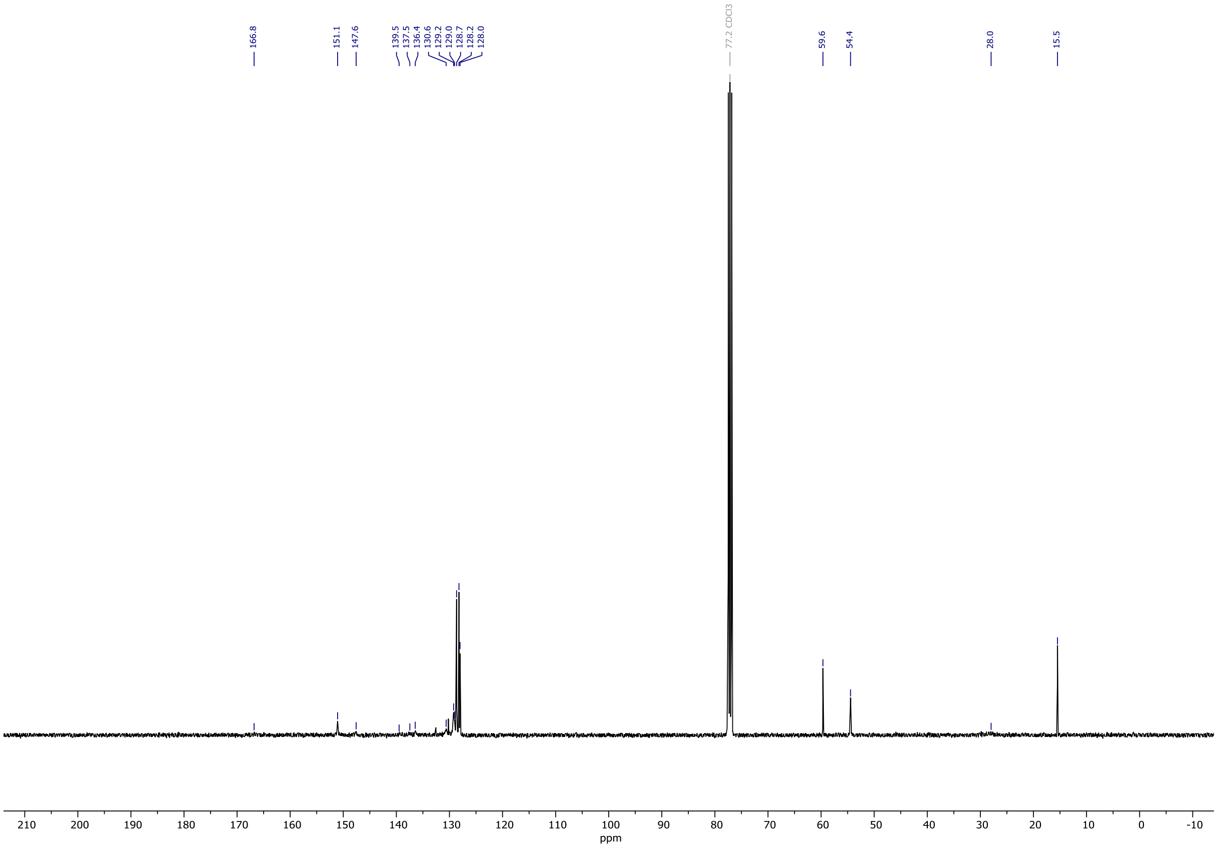

$^{11}B\{^{1}H\}$ NMR ($CDCl_3$, 192 MHz) for **5ag**

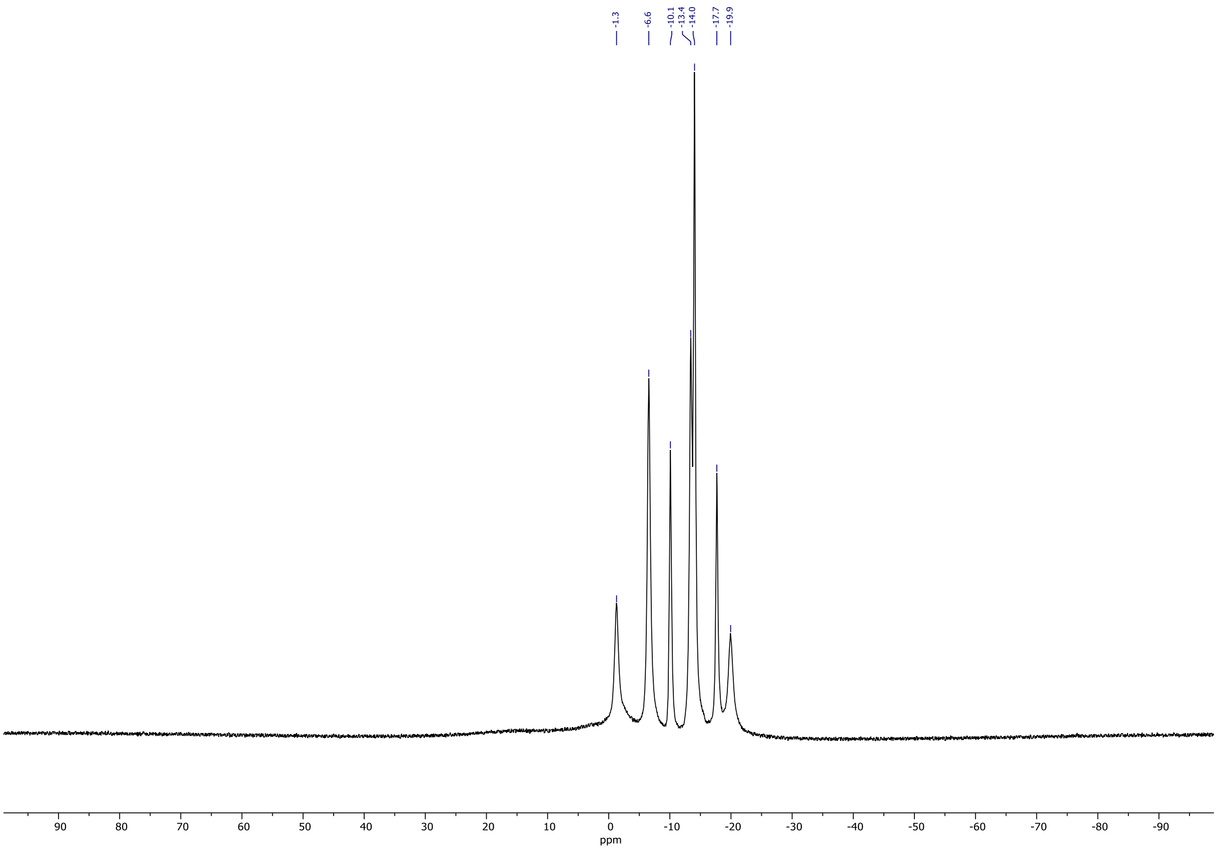

¹H NMR (CDCl$_3$, 400 MHz) for **5ah**

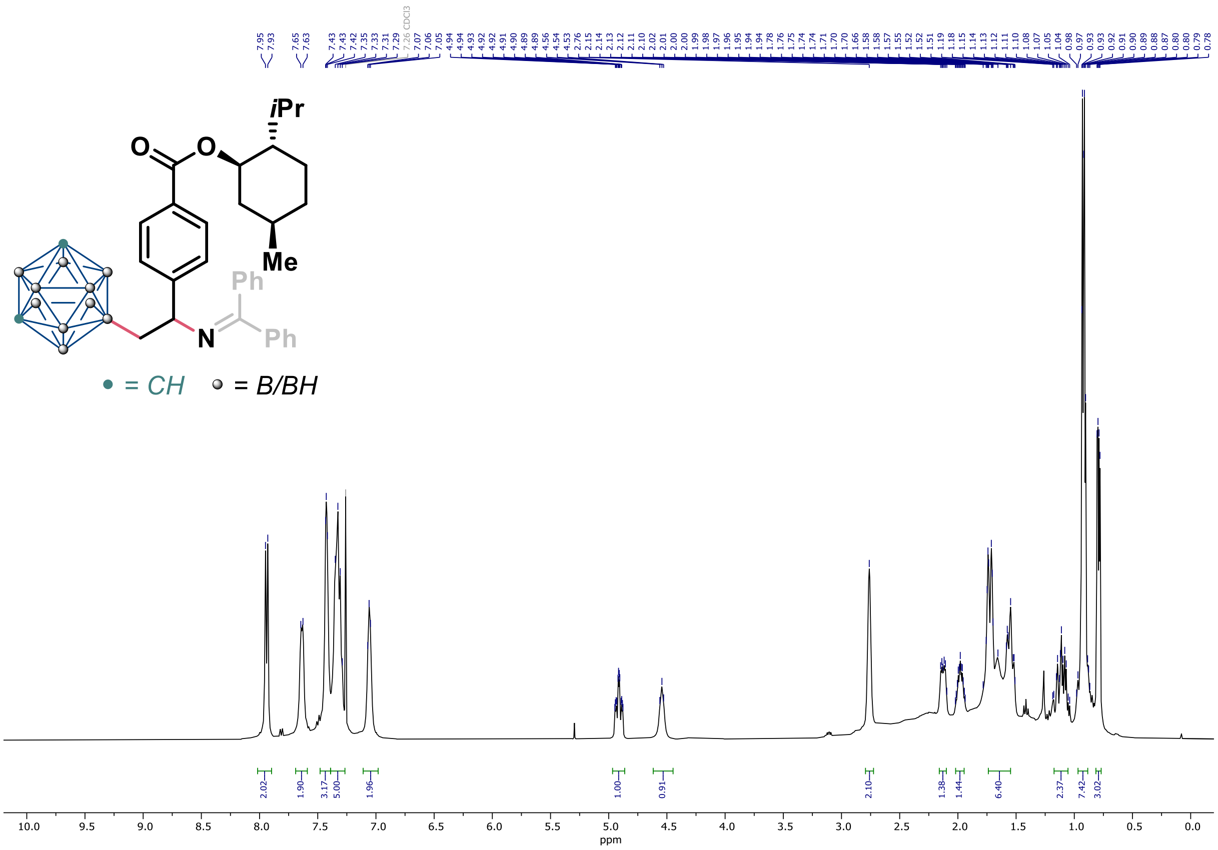

¹³C{¹H} NMR (CDCl$_3$, 101 MHz) for **5ah**

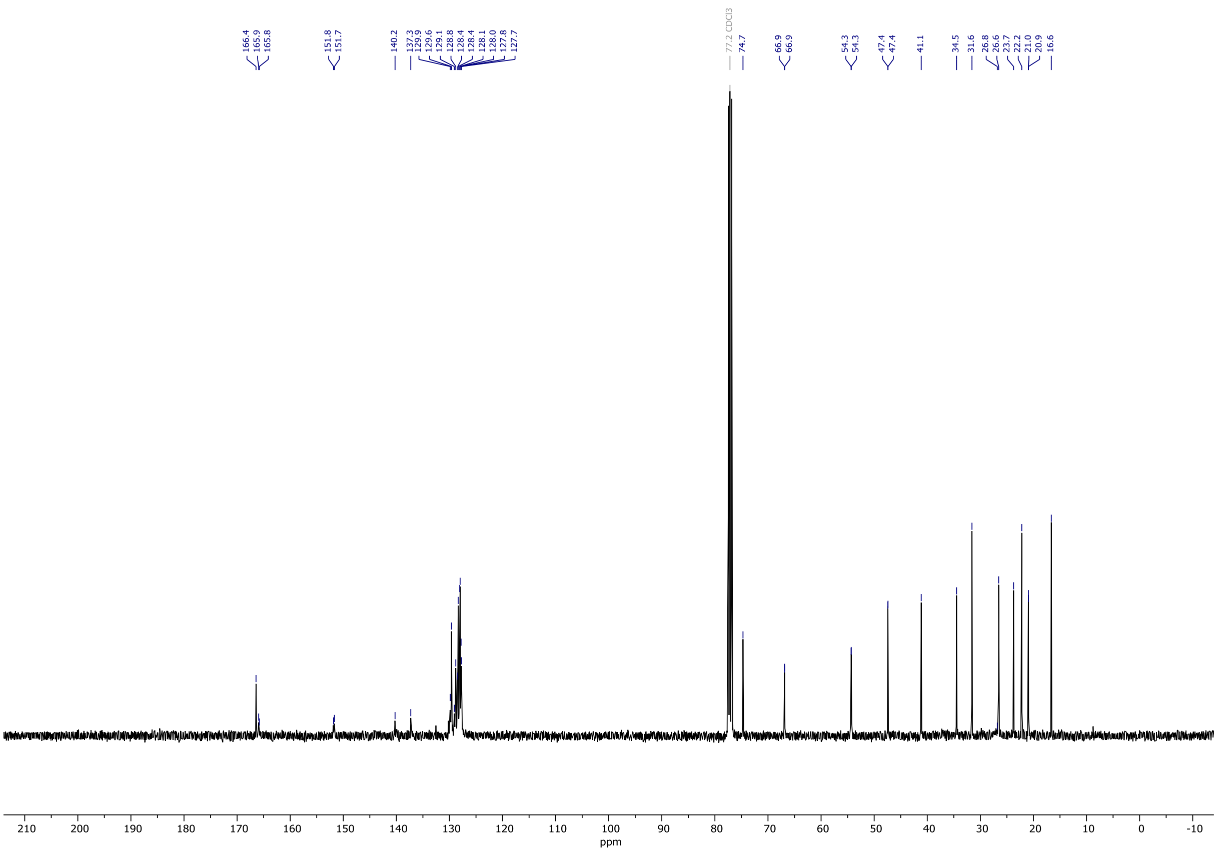

^{11}B{^{1}H} NMR (CDCl$_3$, 128 MHz) for **5ah**

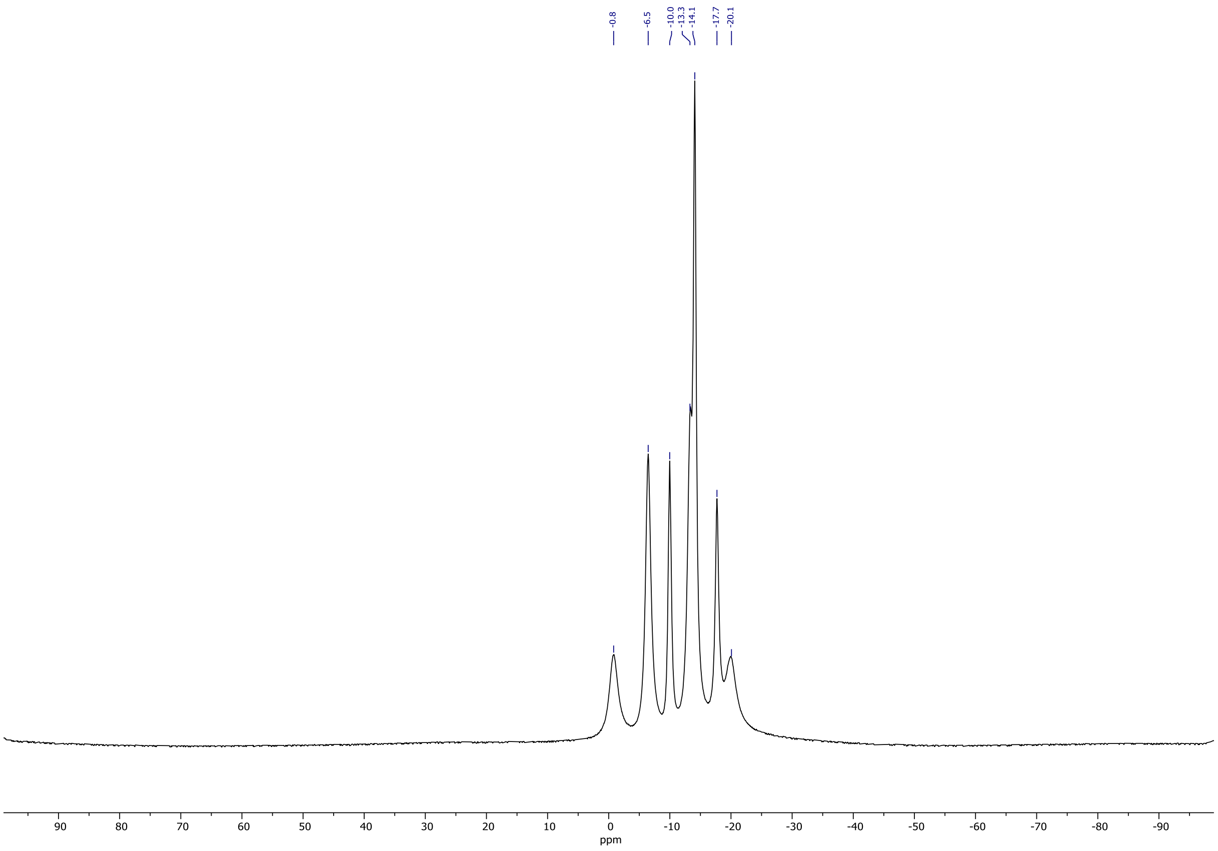

¹H NMR (CDCl$_3$, 400 MHz) for **5ai**

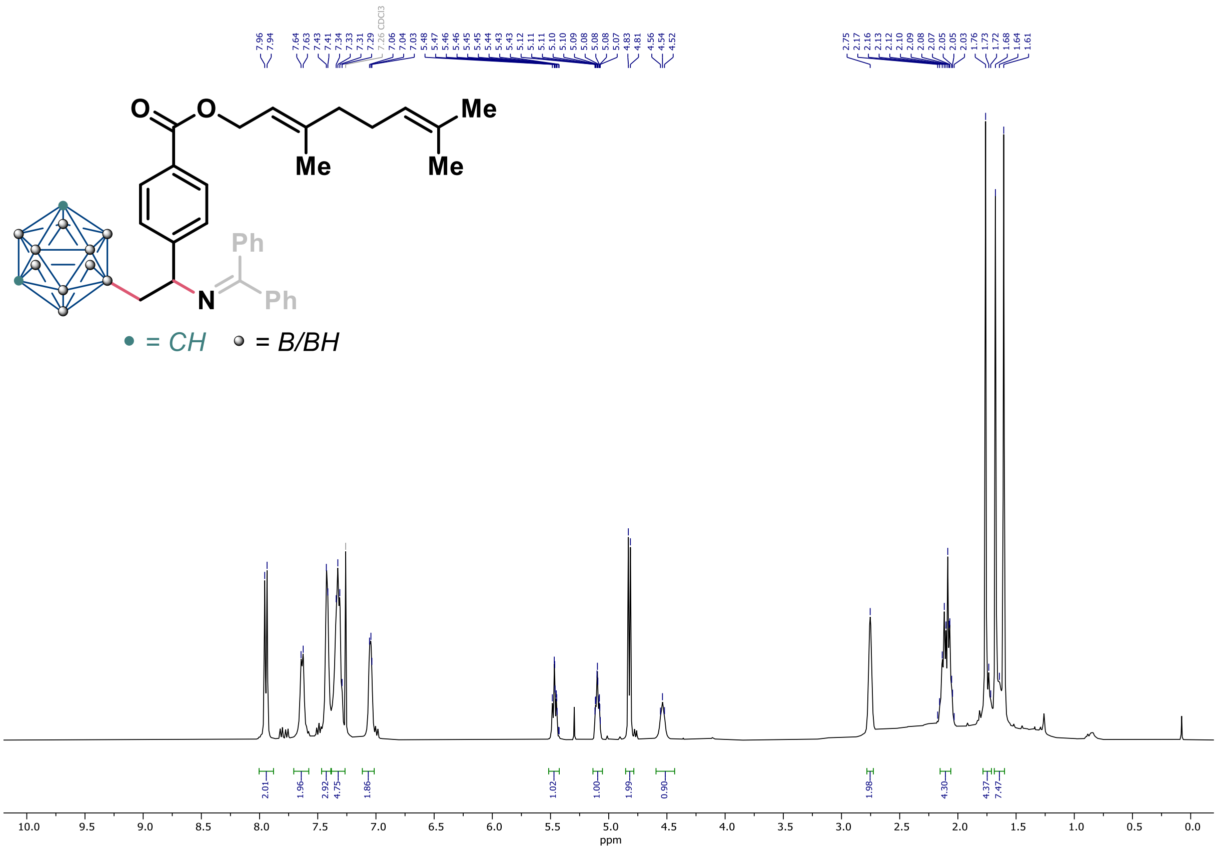

^{13}C{^{1}H} NMR (CDCl$_3$, 101 MHz) for **5ai**

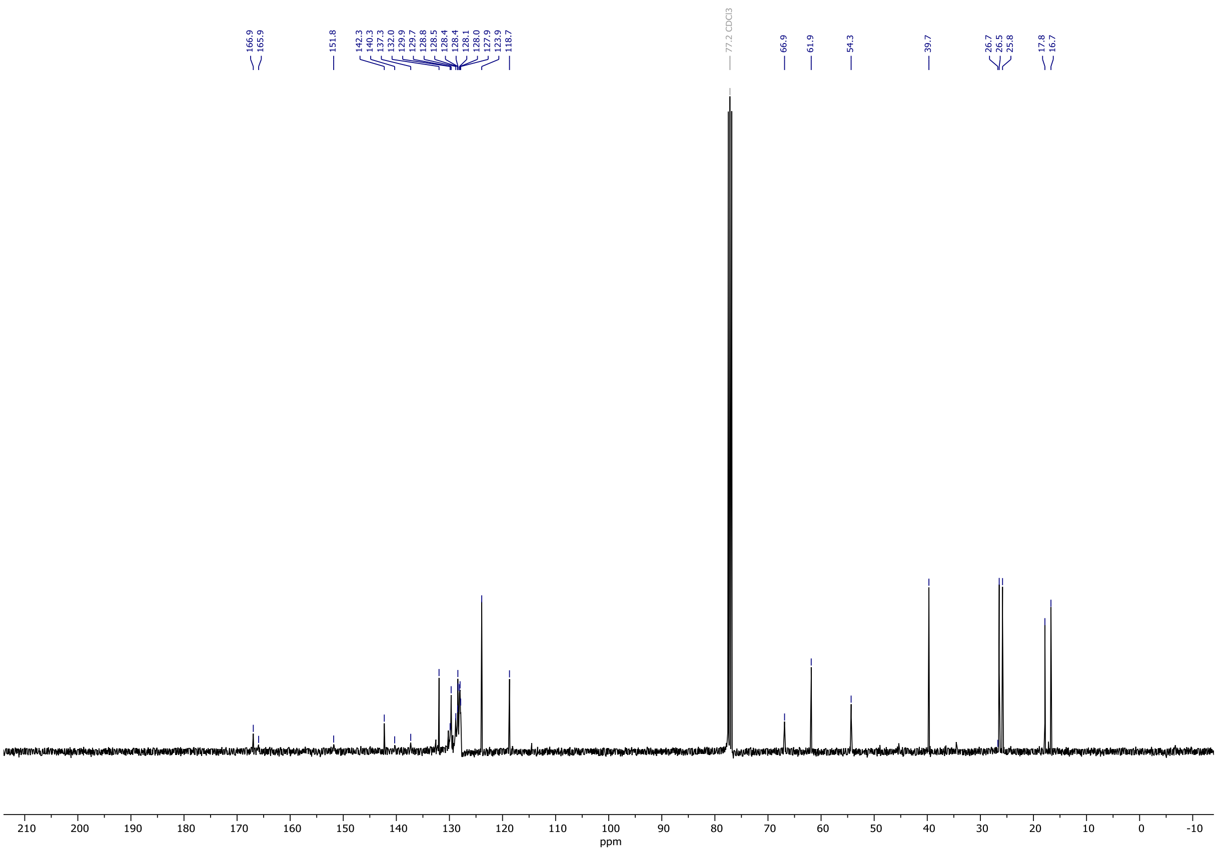

^{11}B{^{1}H} NMR (CDCl$_3$, 128 MHz) for **5ai**

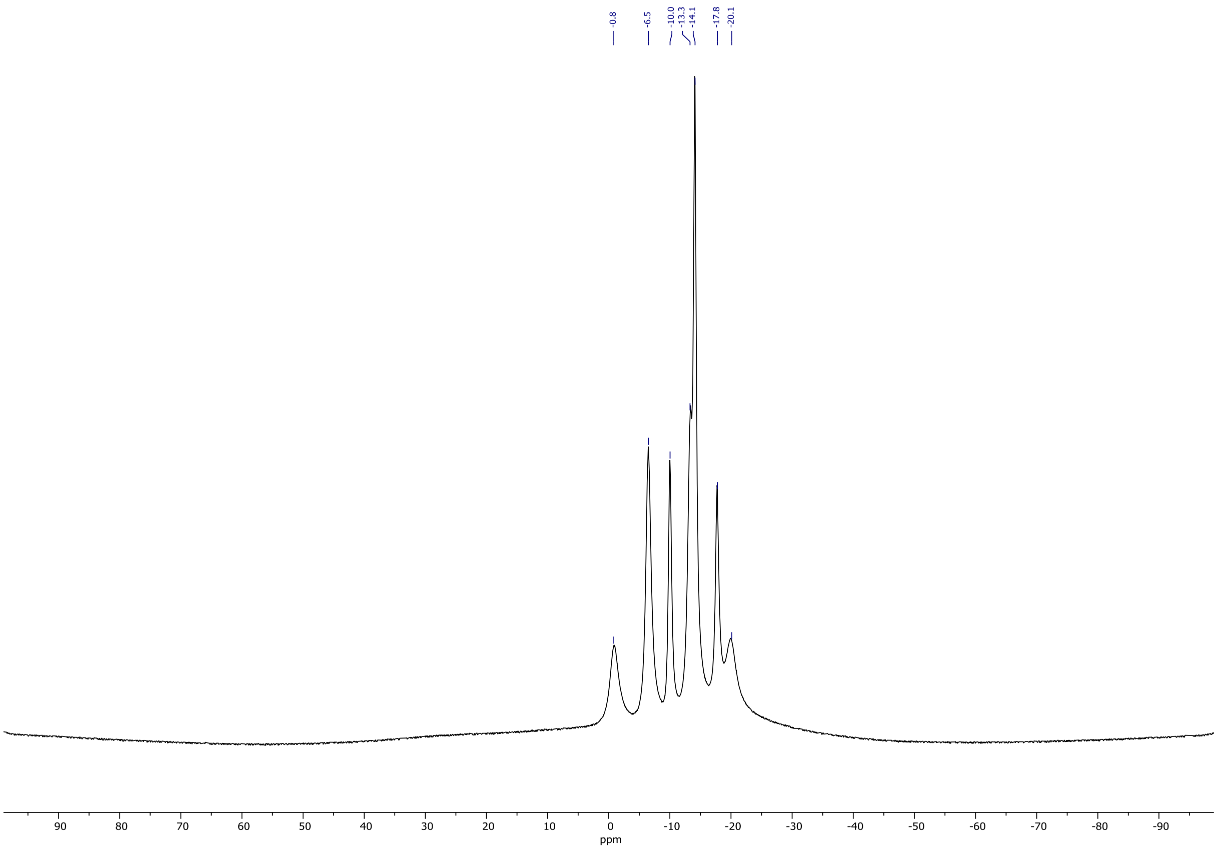

^{1}H NMR ($CDCl_3$, 400 MHz) for **5aj**

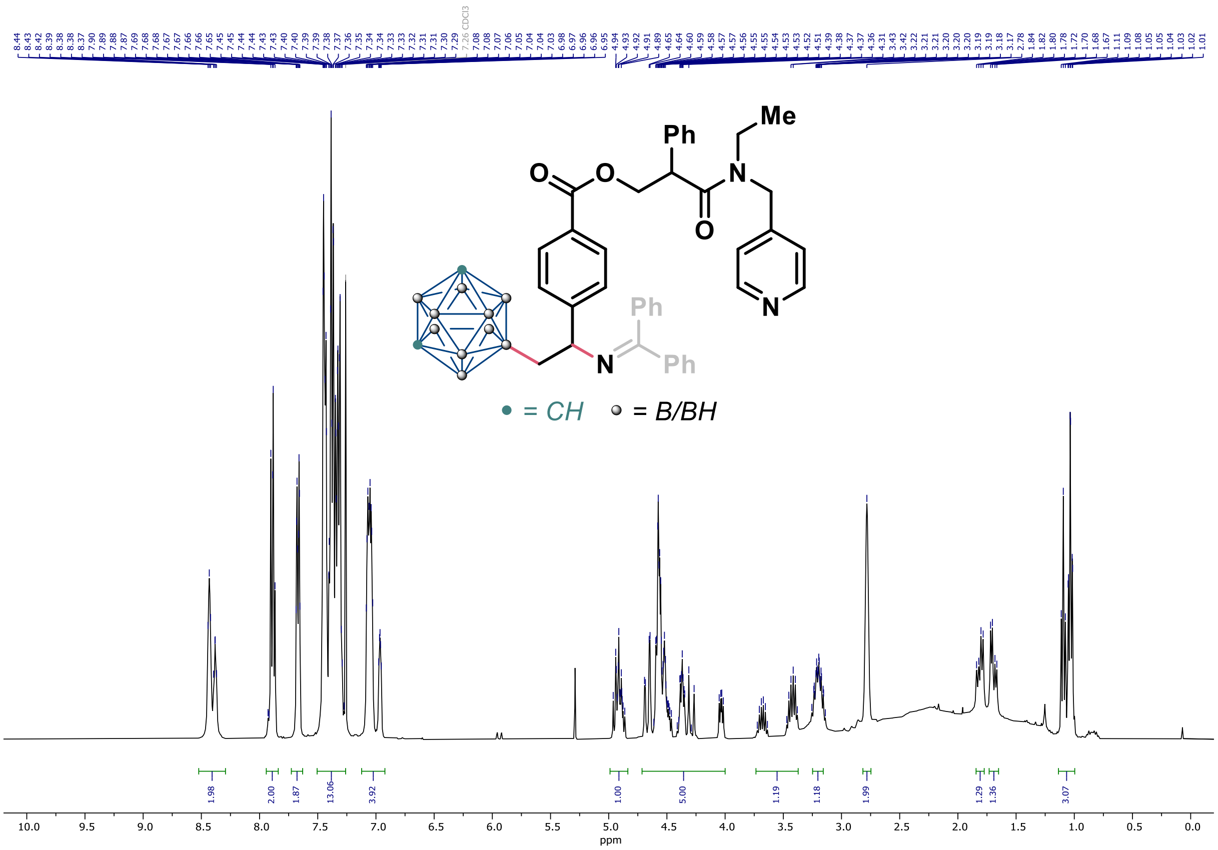

^{13}C{^{1}H} NMR ($CDCl_3$, 101 MHz) for **5aj**

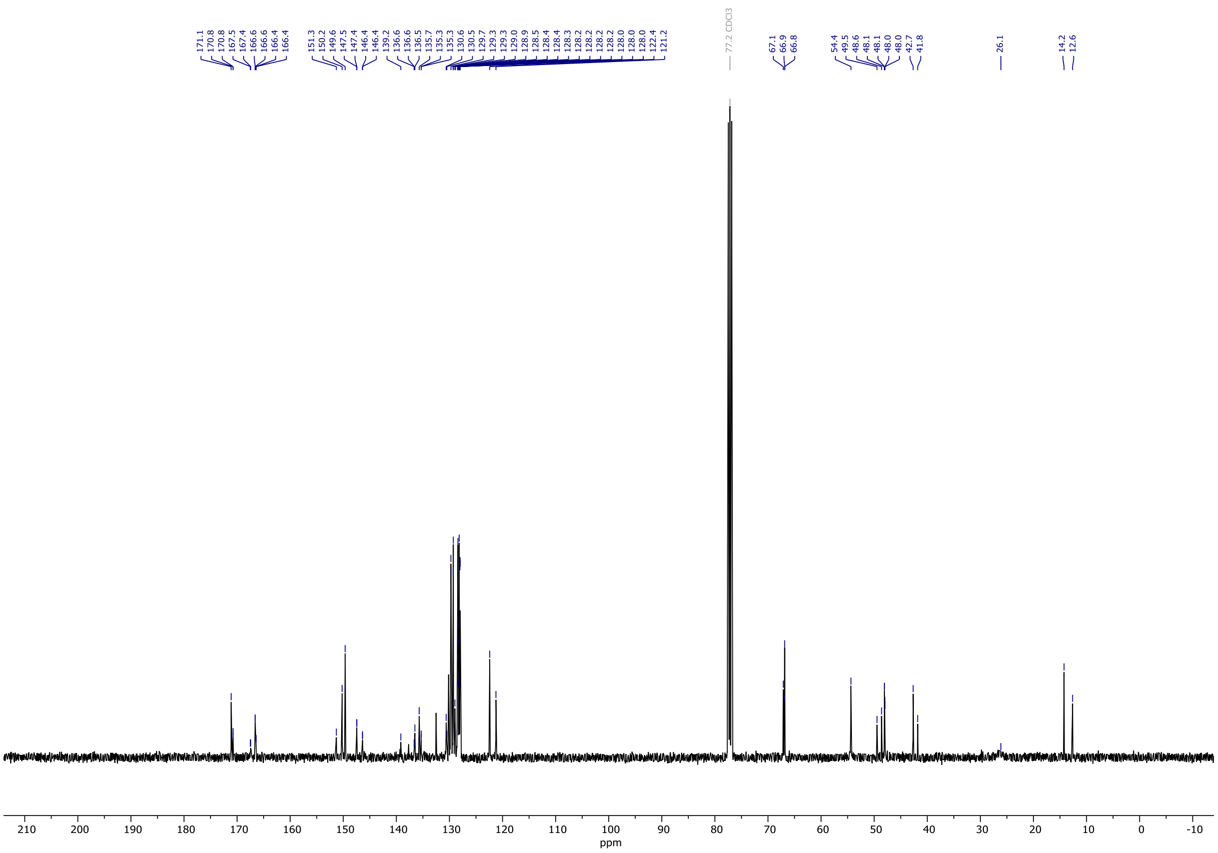

$^{11}B\{^1H\}$ NMR ($CDCl_3$, 128 MHz) for **5aj**

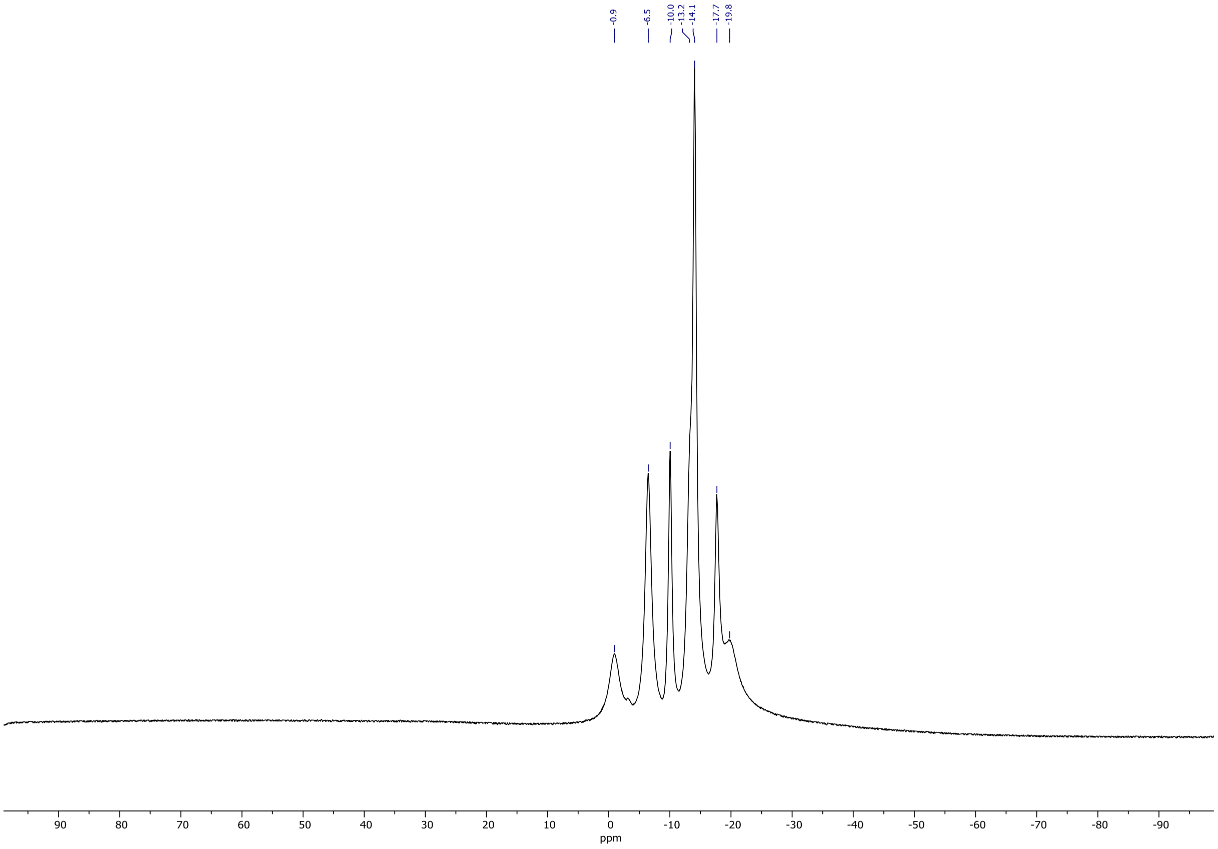

^{1}H NMR ($CDCl_3$, 400 MHz) for **5ak**

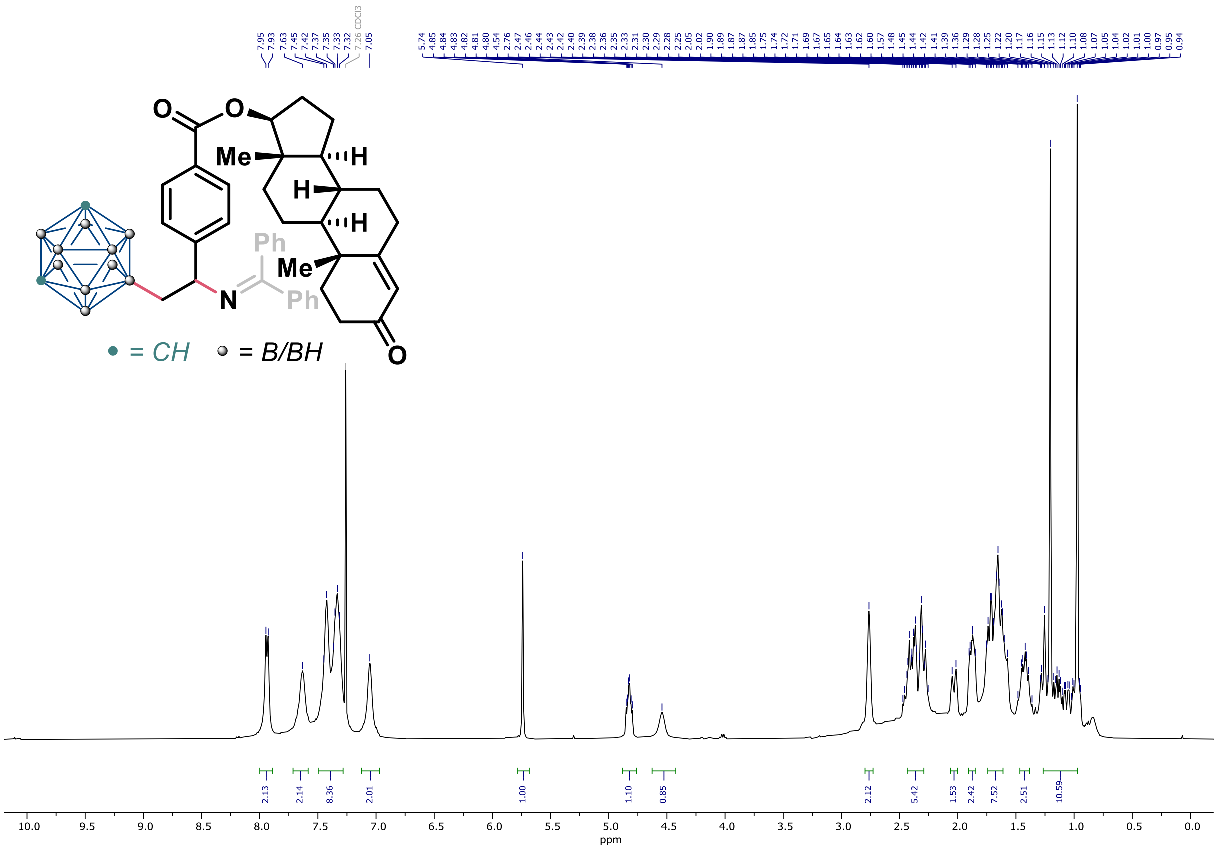

^{13}C{^{1}H} NMR ($CDCl_3$, 101 MHz) for **5ak**

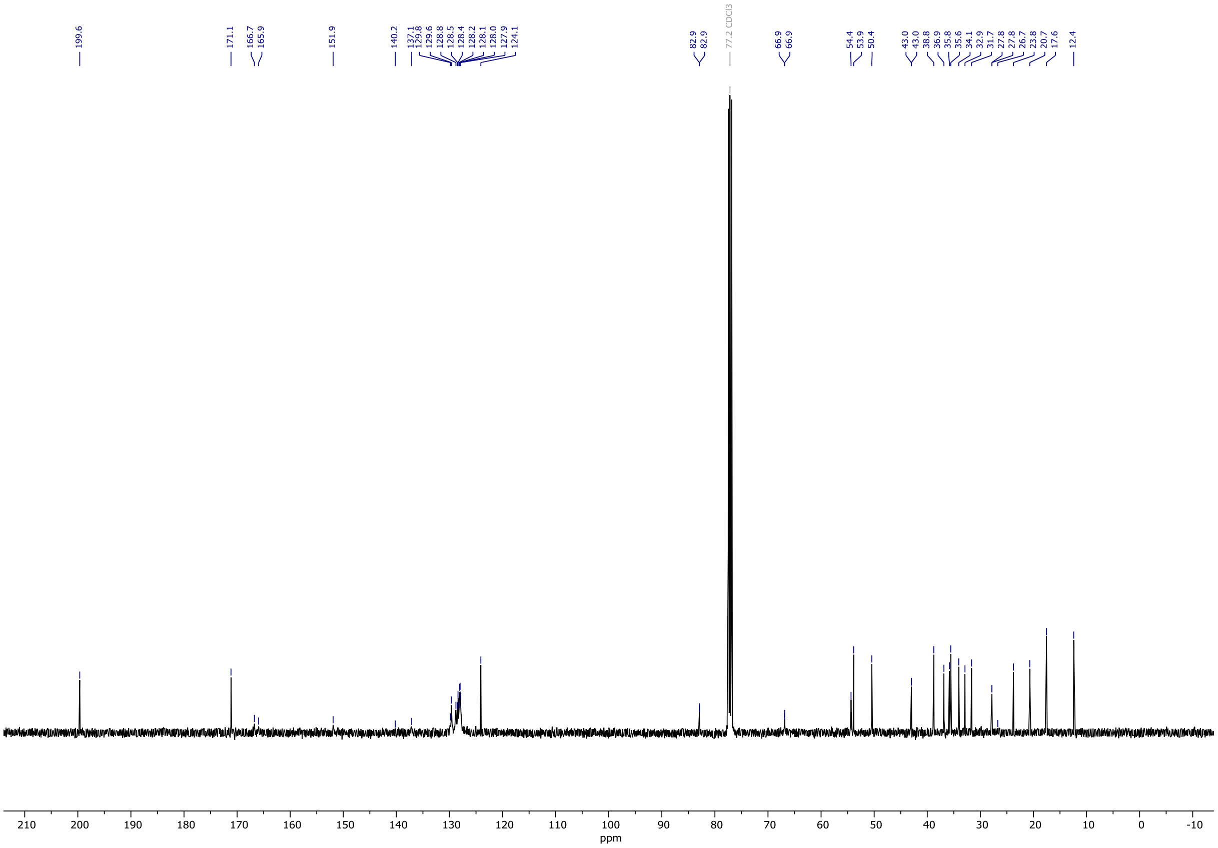

$^{11}B\{^1H\}$ NMR ($CDCl_3$, 128 MHz) for **5ak**

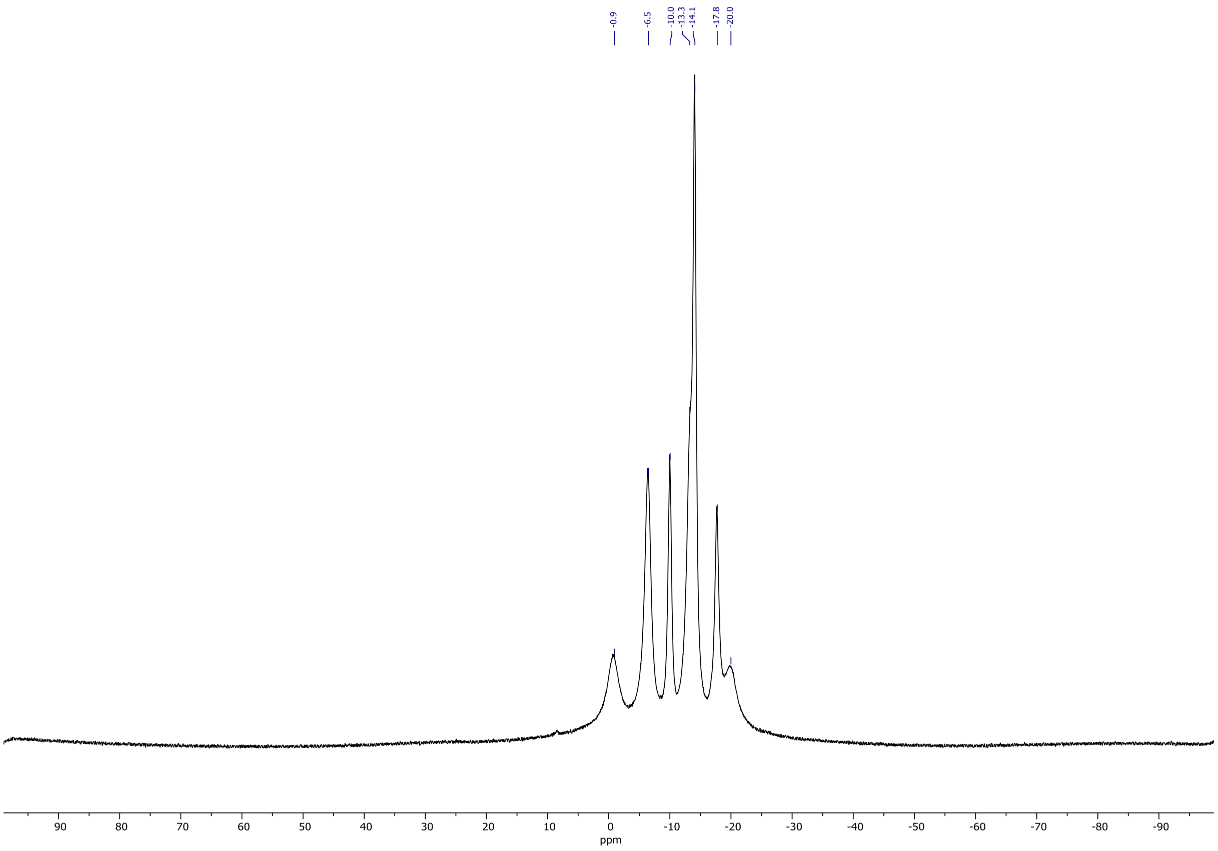

^{1}H NMR ($CDCl_3$, 400 MHz) for **5al**

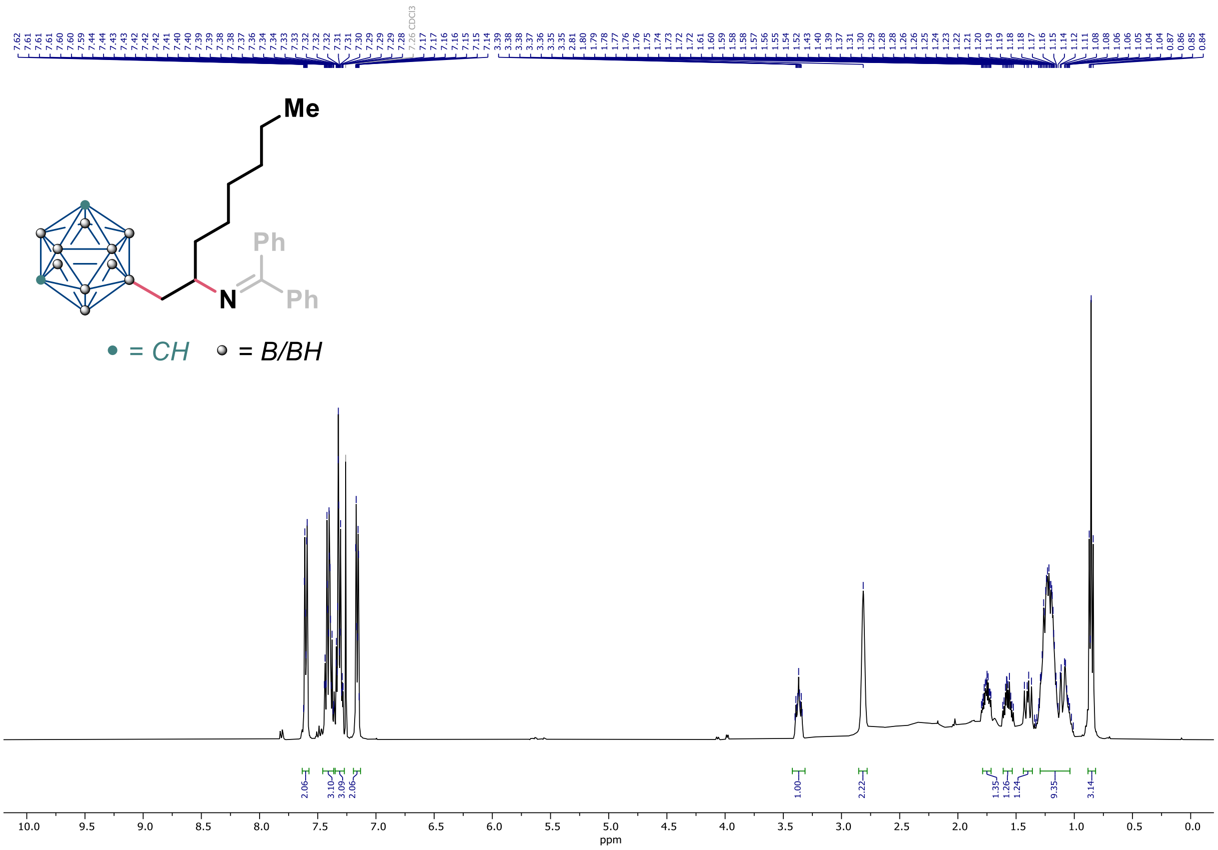

^{13}C{^{1}H} NMR ($CDCl_3$, 101 MHz) for **5al**

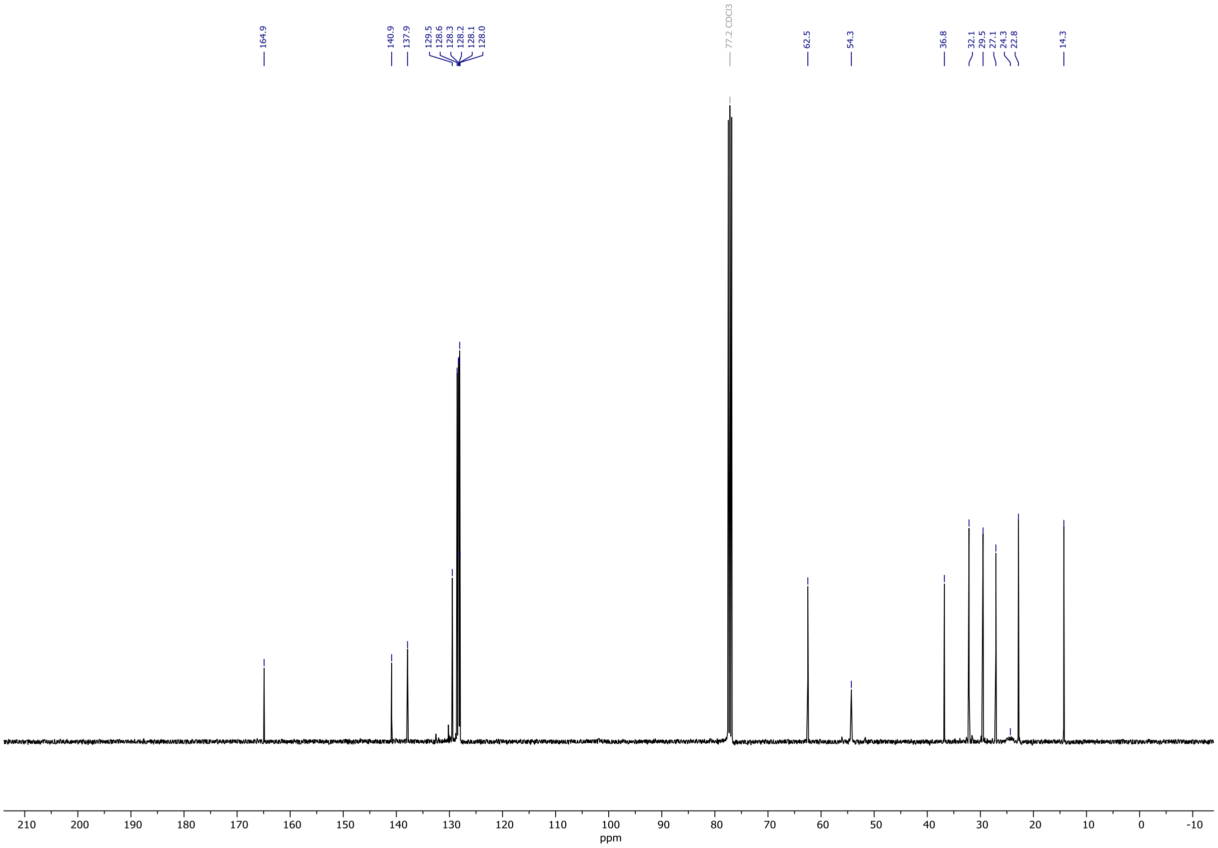

^{11}B{^{1}H} NMR (CDCl$_3$, 128 MHz) for **5al**

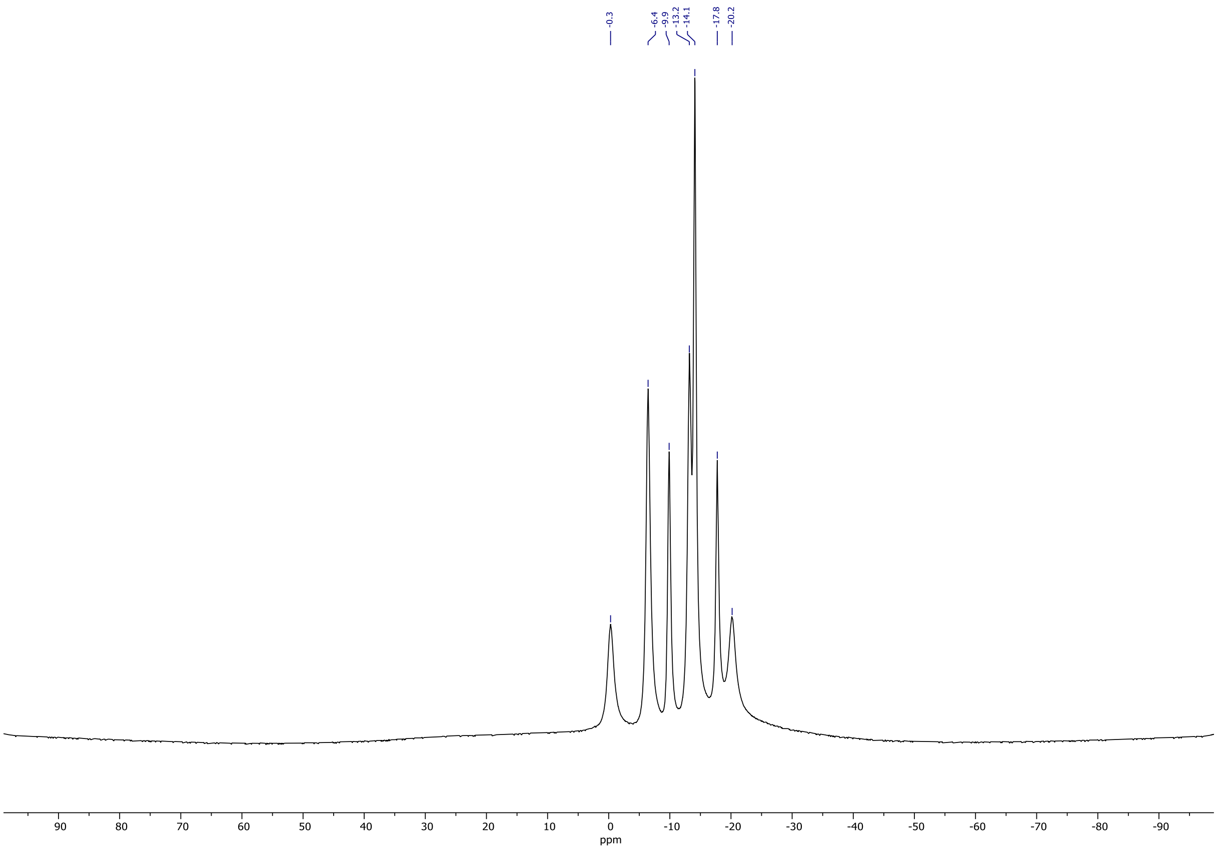

^{1}H NMR ($CDCl_3$, 400 MHz) for **5am**

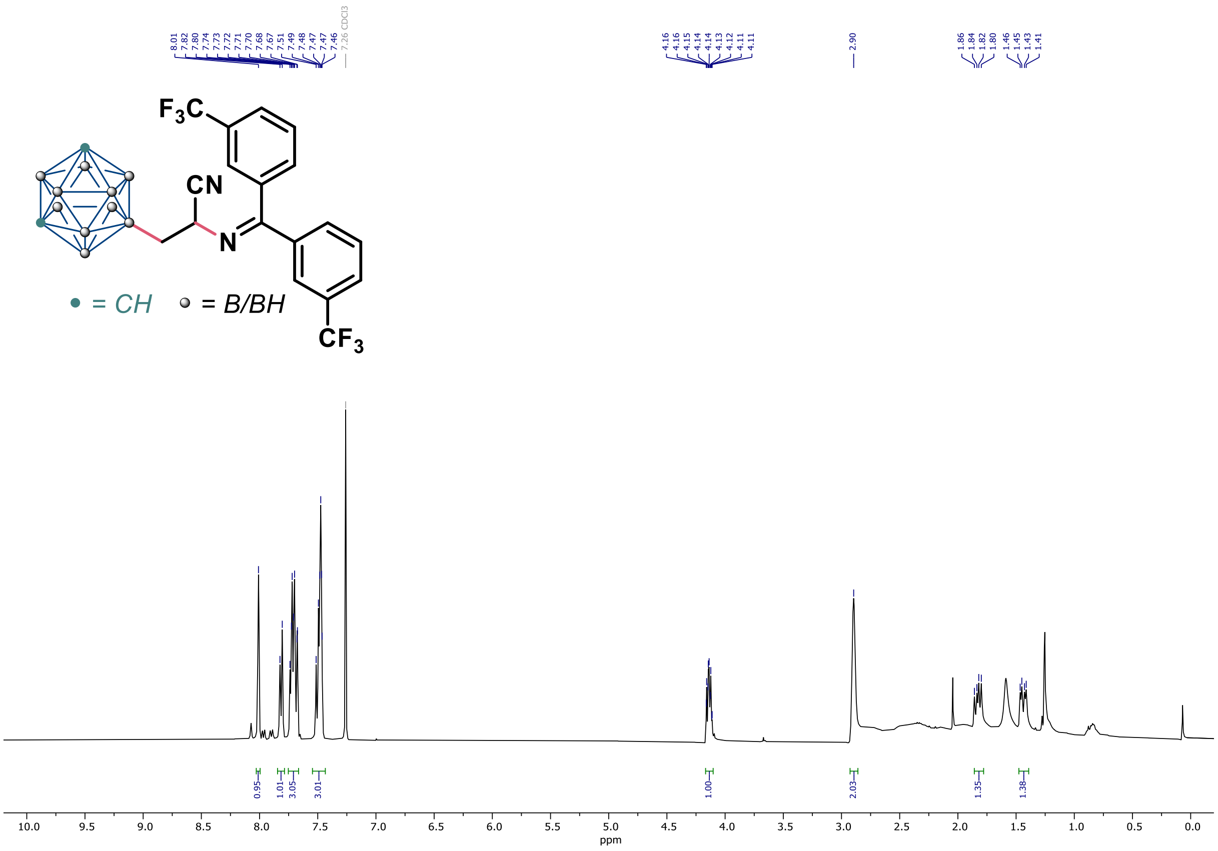

^{13}C{^{1}H} NMR ($CDCl_3$, 126 MHz) for **5am**

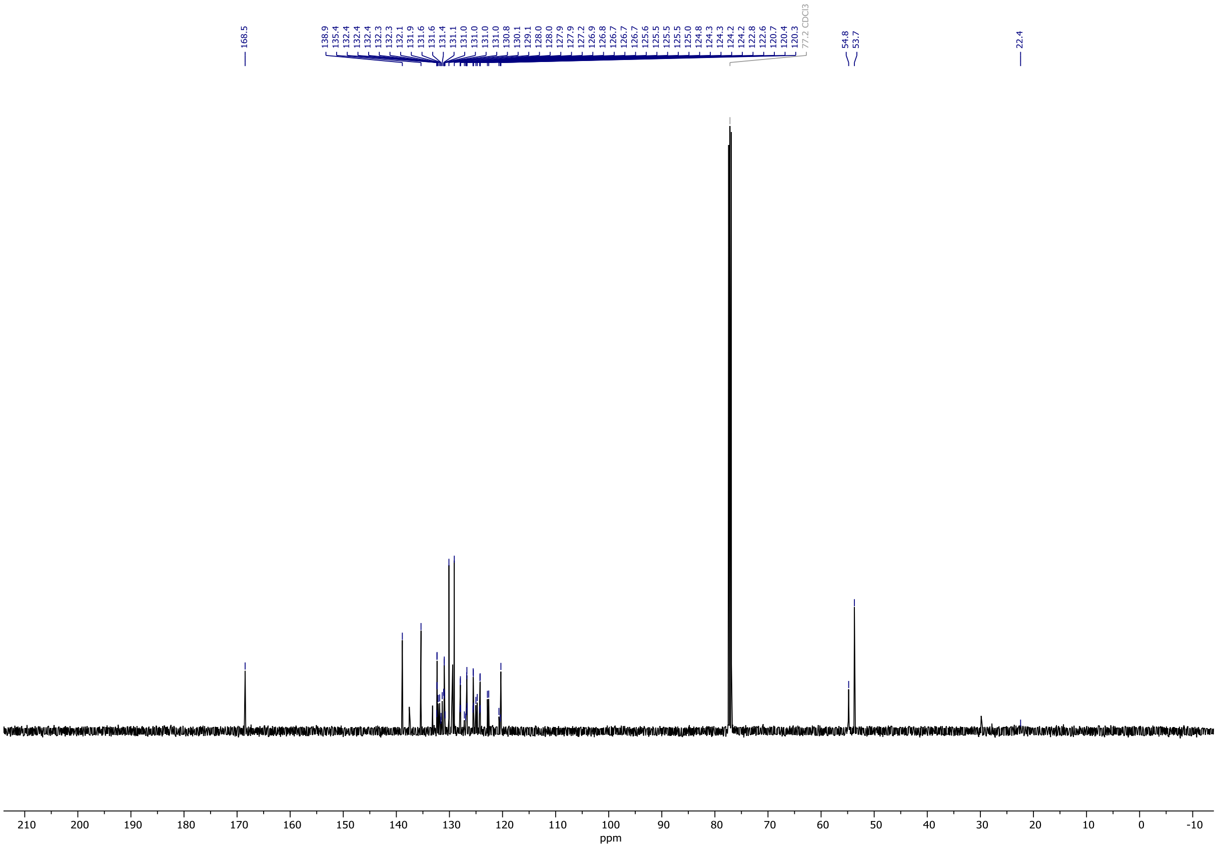

$^{11}B\{^{1}H\}$ NMR ($CDCl_3$, 128 MHz) for **5am**

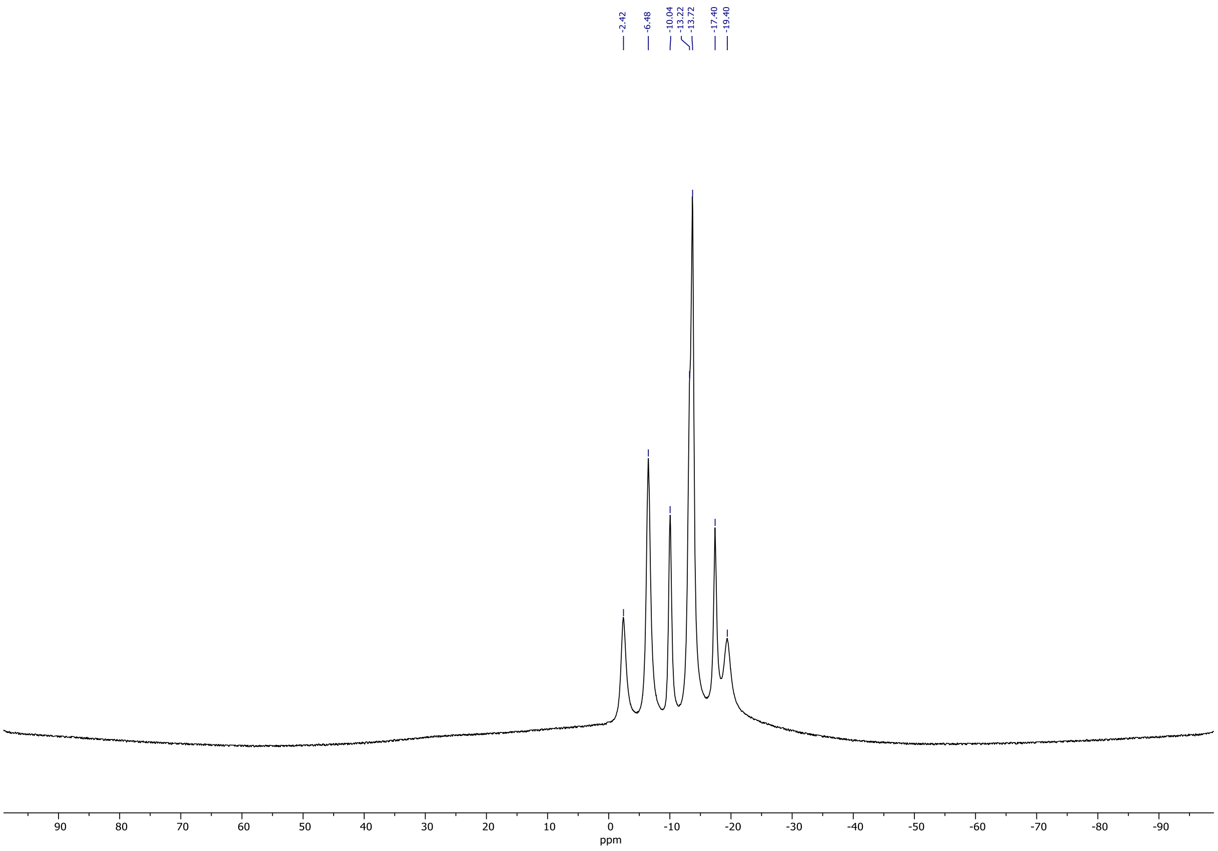

^{19}F NMR ($CDCl_3$, 376 MHz) for **5am**

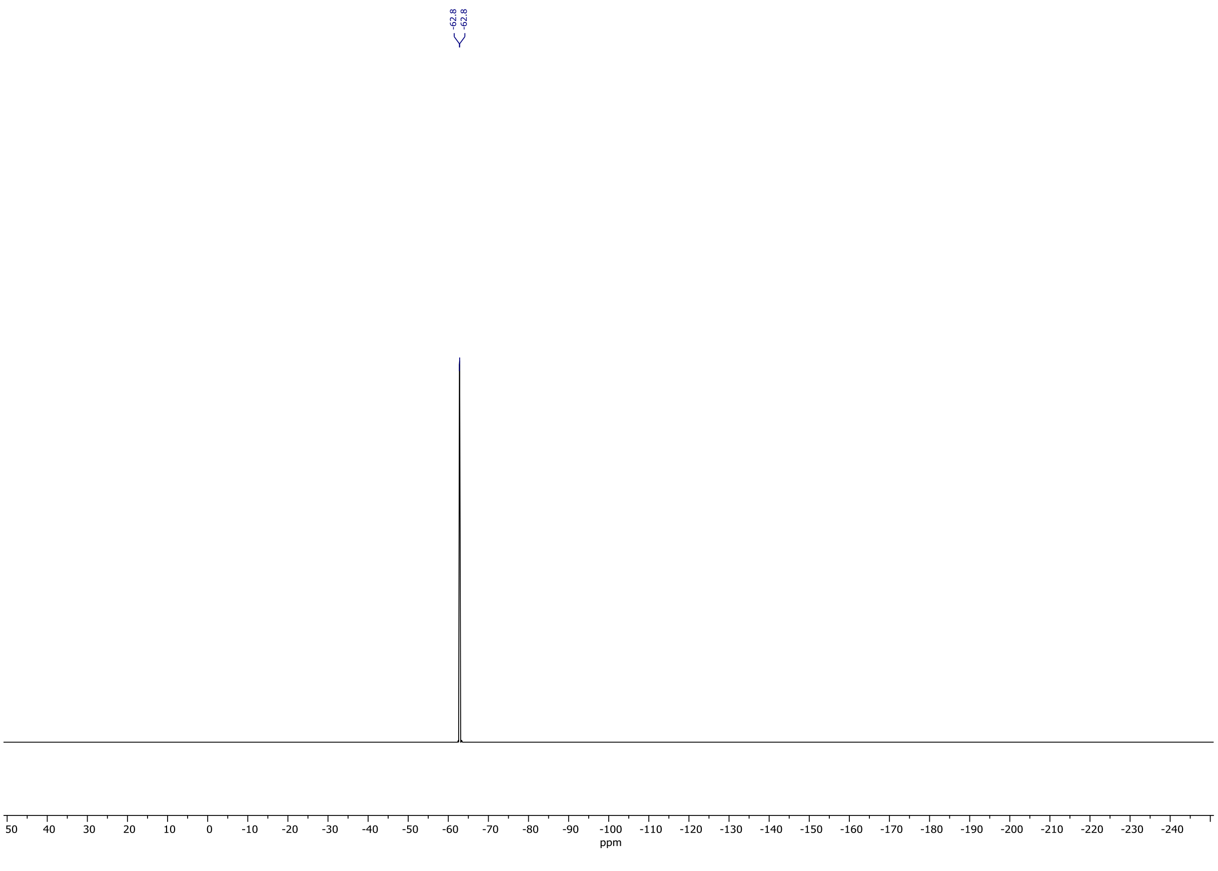

¹H NMR (CDCl$_3$, 400 MHz) for **5an**

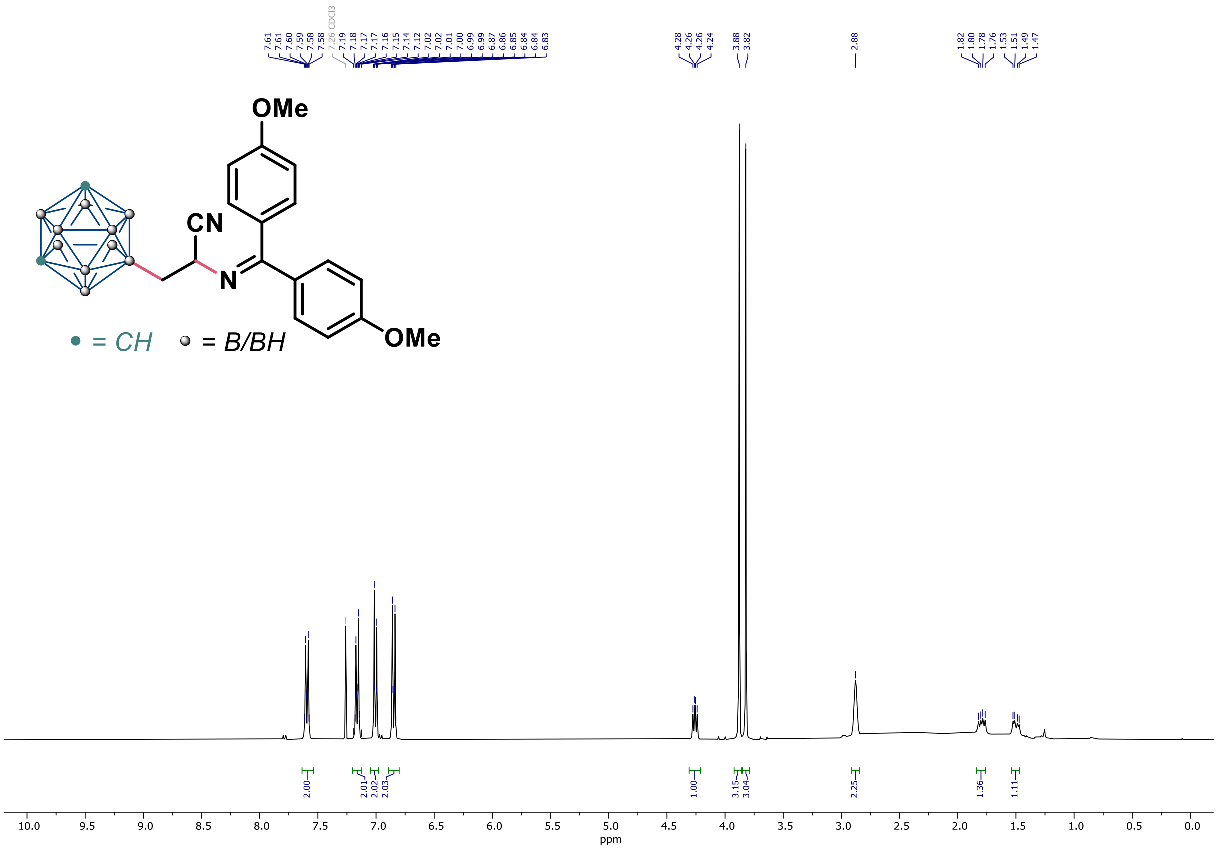

^{13}C{¹H} NMR (CDCl$_3$, 101 MHz) for **5an**

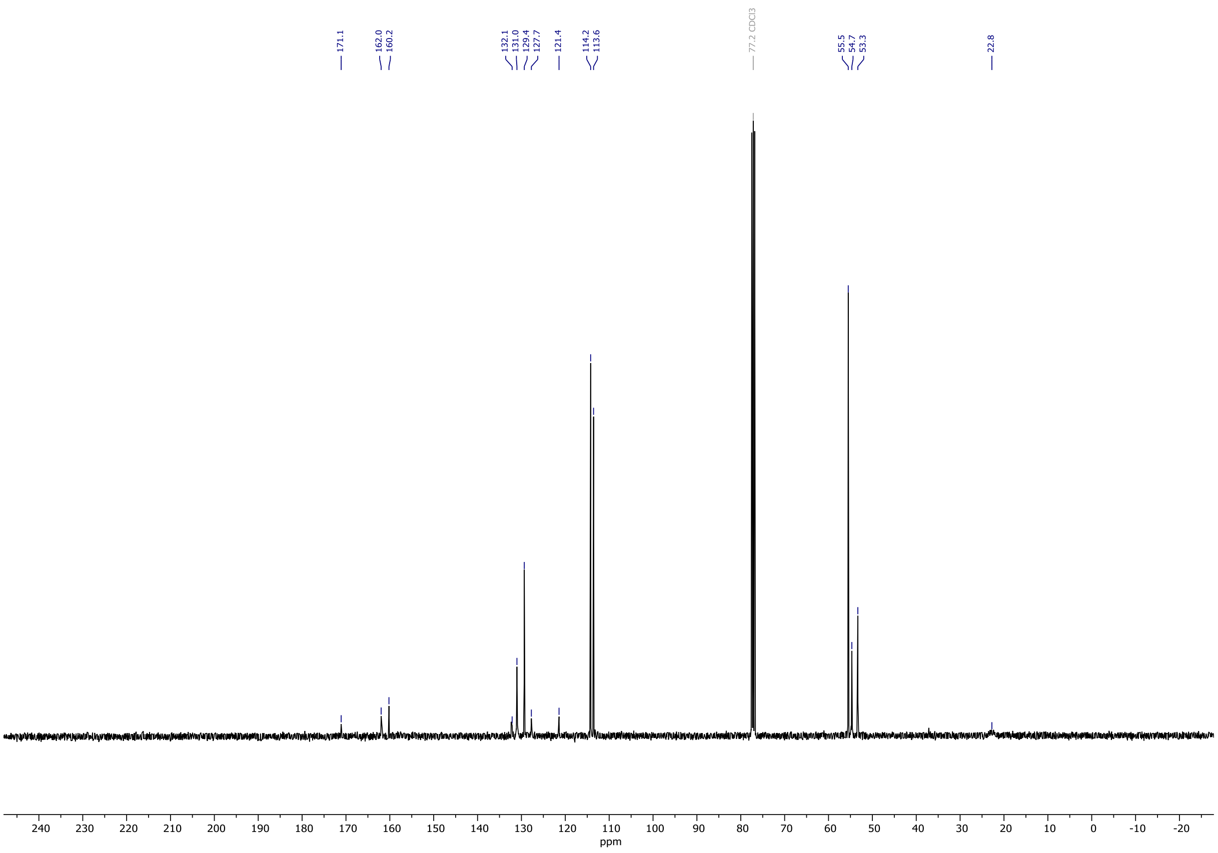

^{11}B{^{1}H} NMR ($CDCl_3$, 128 MHz) for **5an**

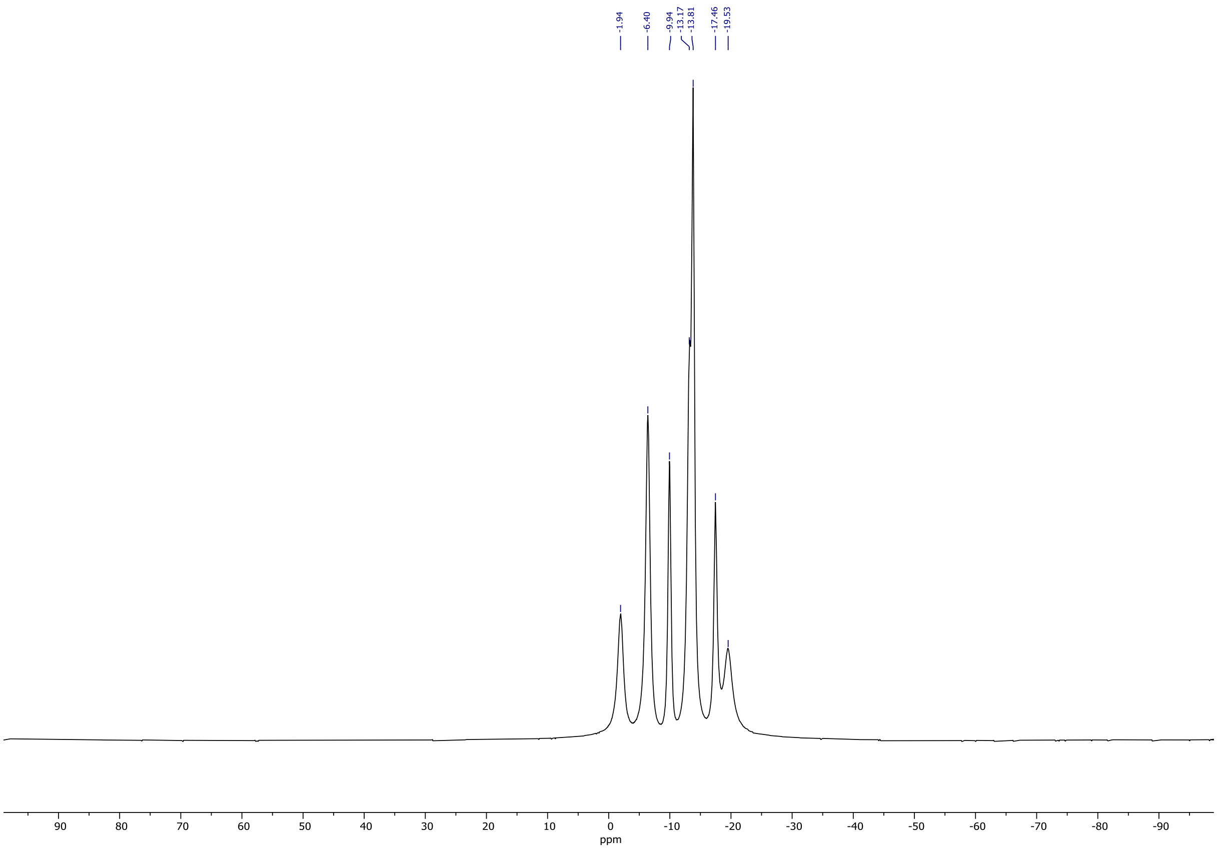

^{1}H NMR (CDCl$_3$, 400 MHz) for **5ao**

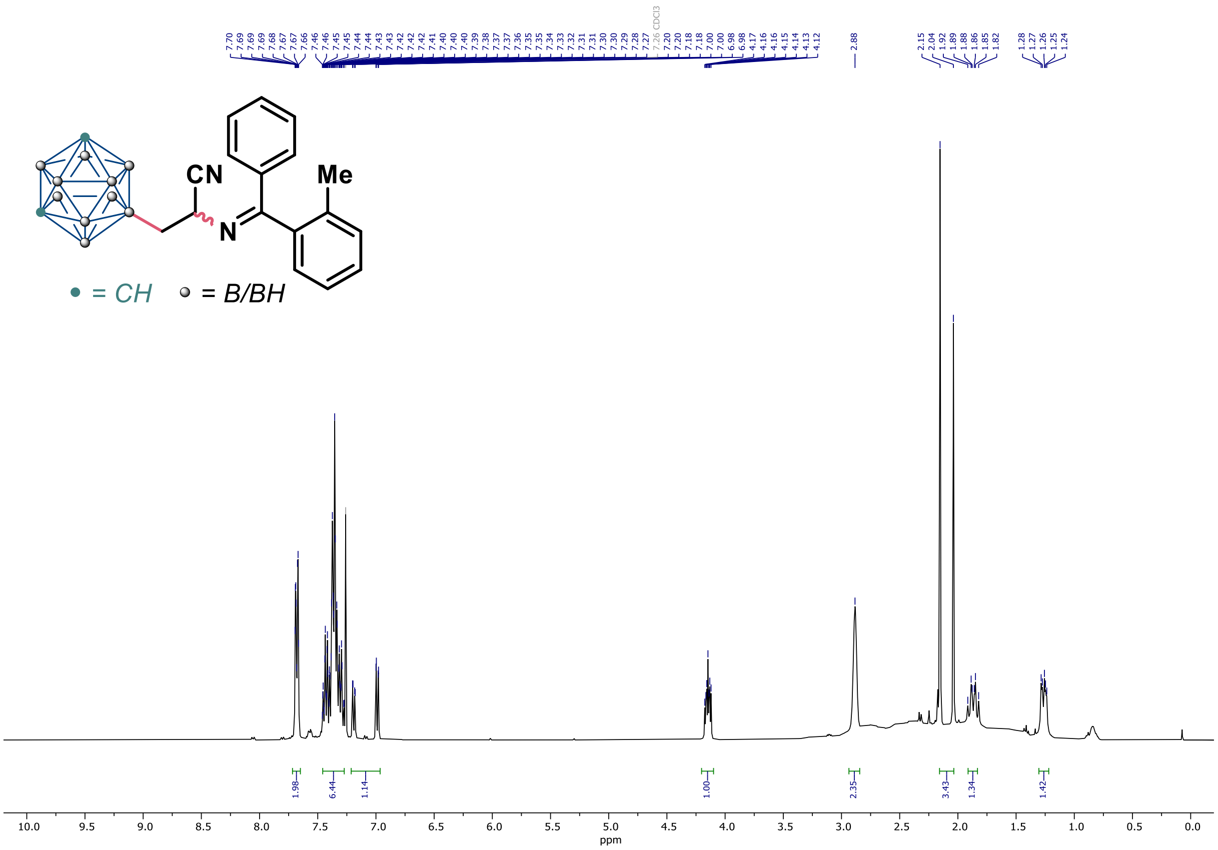

^{13}C{^{1}H} NMR (CDCl$_3$, 101 MHz) for **5ao**

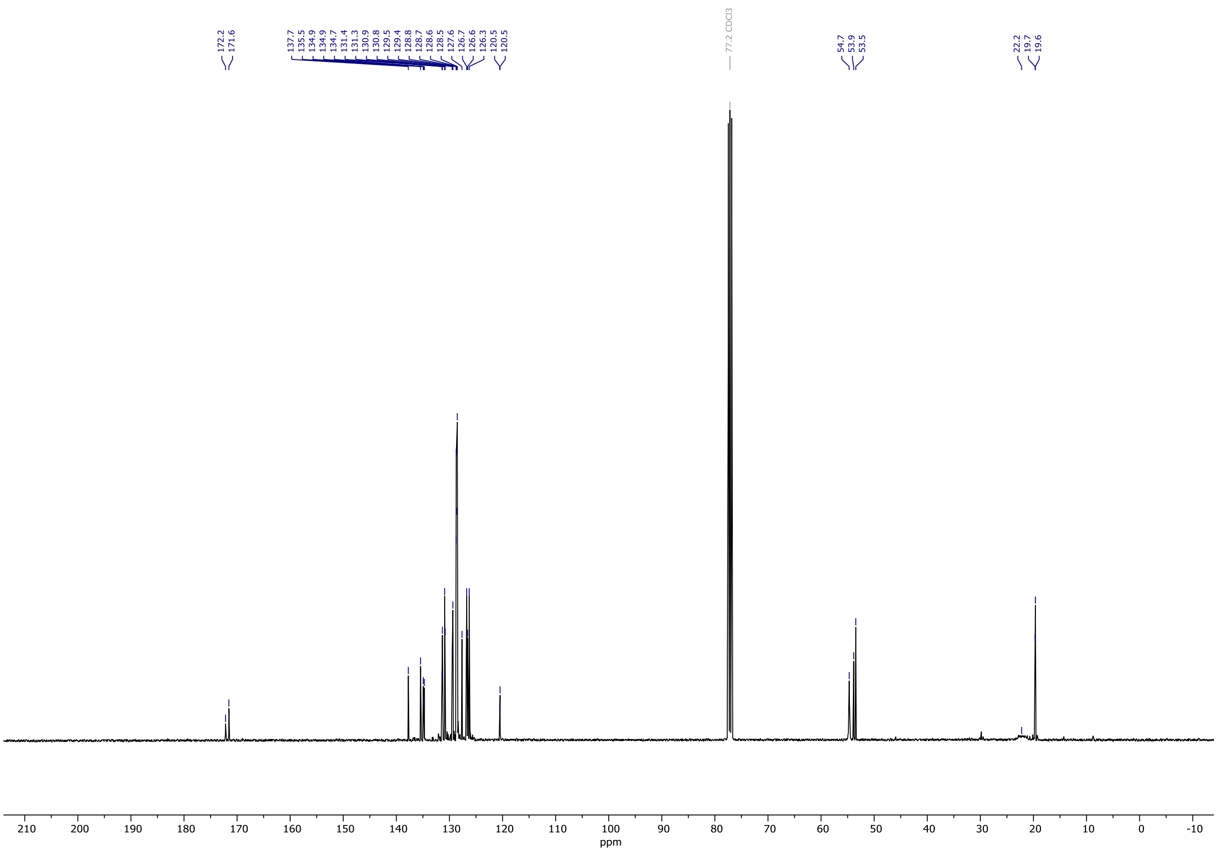

^{11}B{^{1}H} NMR (CDCl$_3$, 128 MHz) for **5ao**

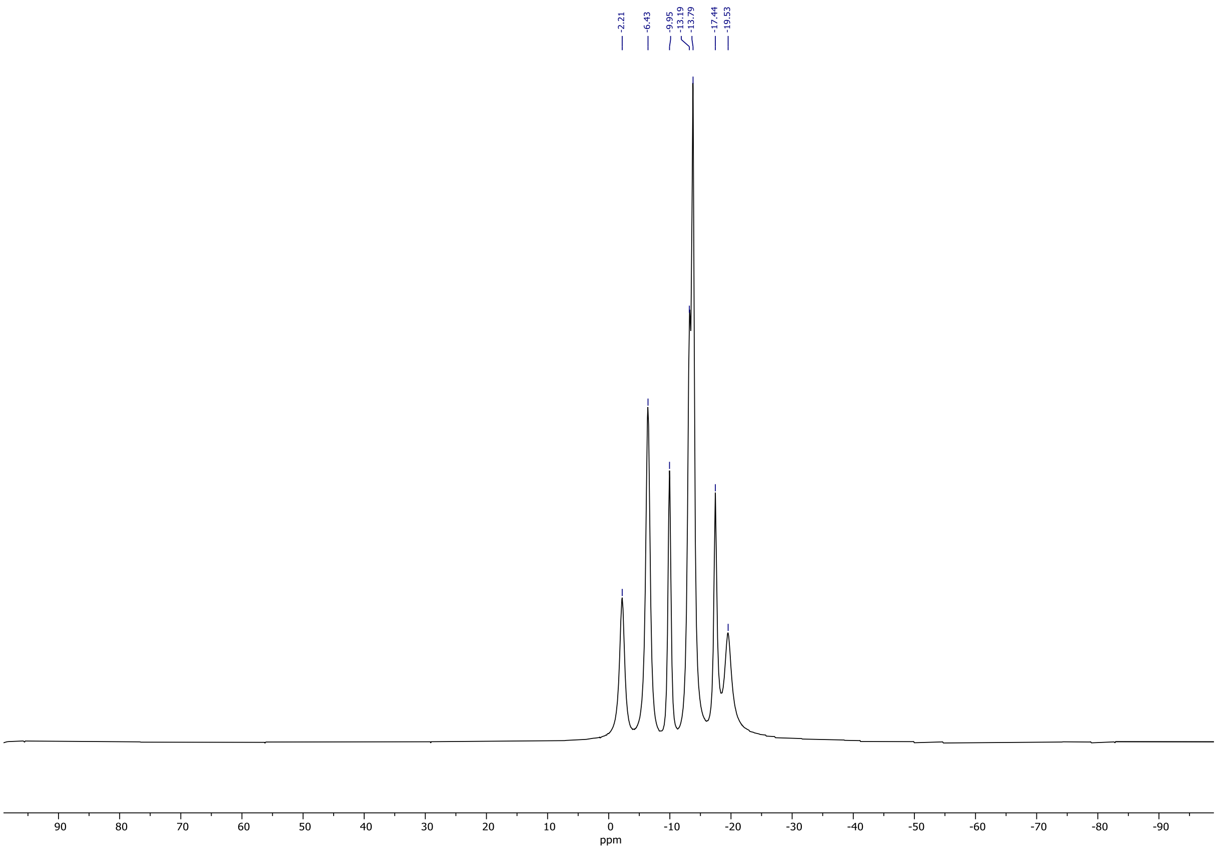

8.3 Mechanistic investigations

^{1}H NMR ($CDCl_3$, 400 MHz) for **6**

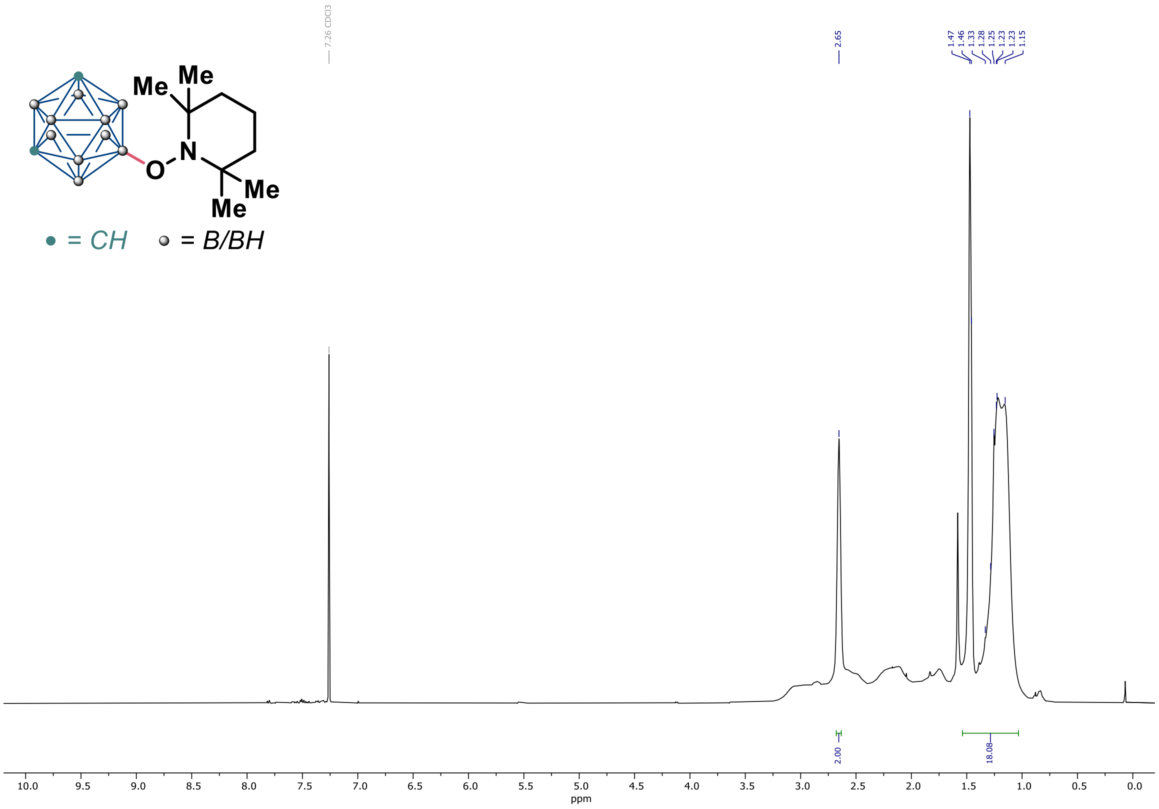

^{13}C{^{1}H} NMR ($CDCl_3$, 101 MHz) for **6**

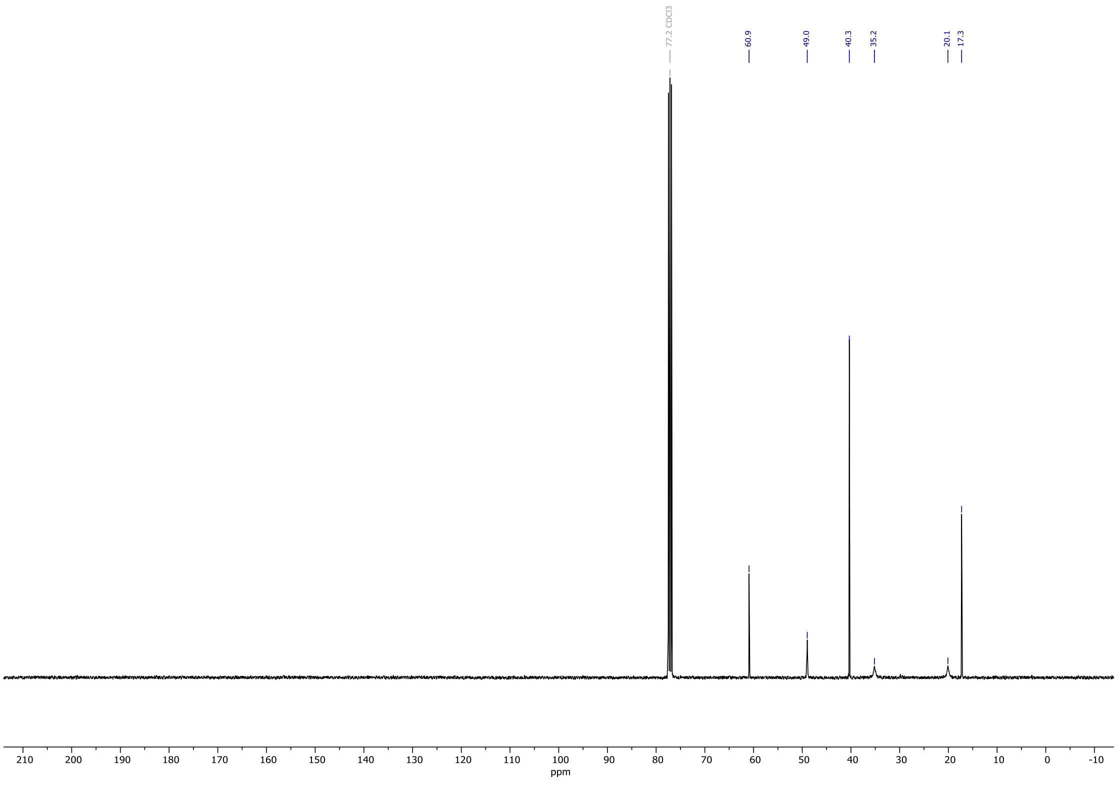

$^{11}B\{^1H\}$ NMR (CDCl$_3$, 128 MHz) for **6**

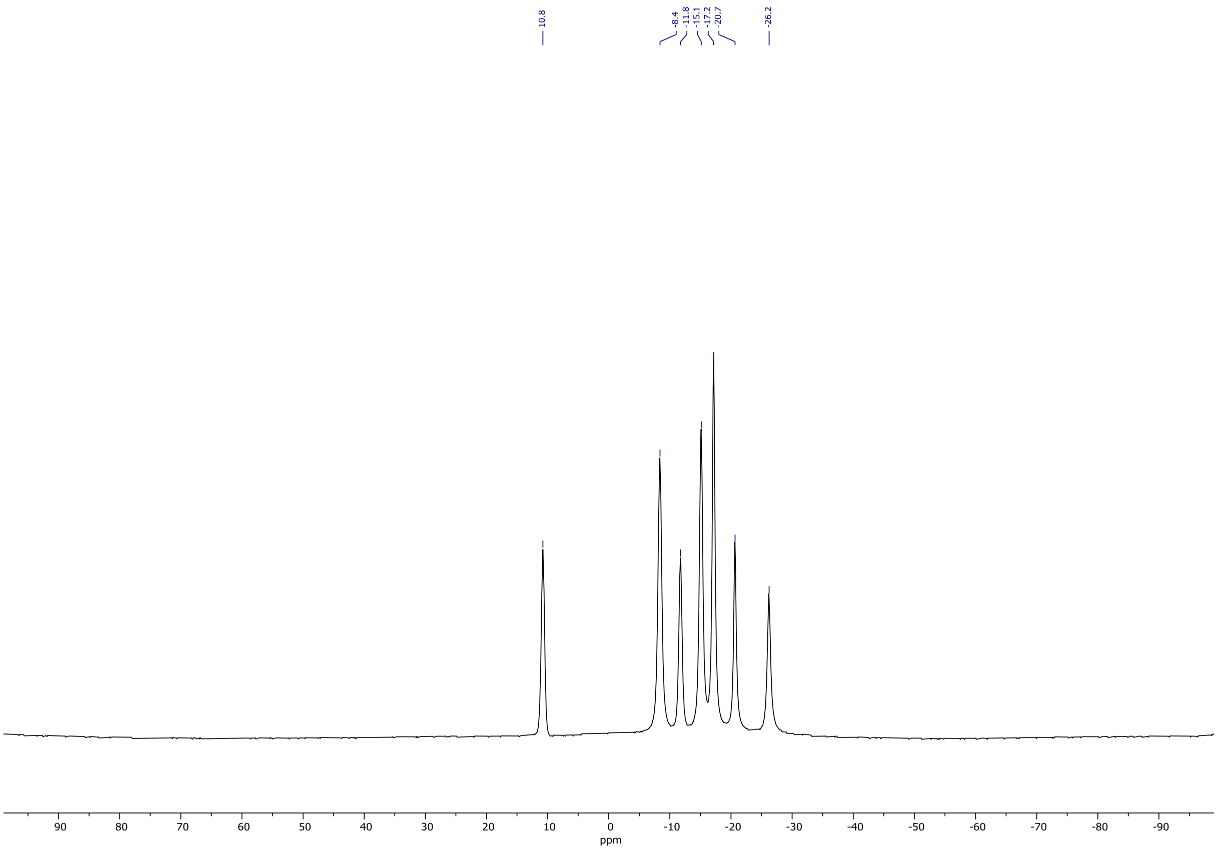

^{1}H NMR ($CDCl_3$, 400 MHz) for **7a**

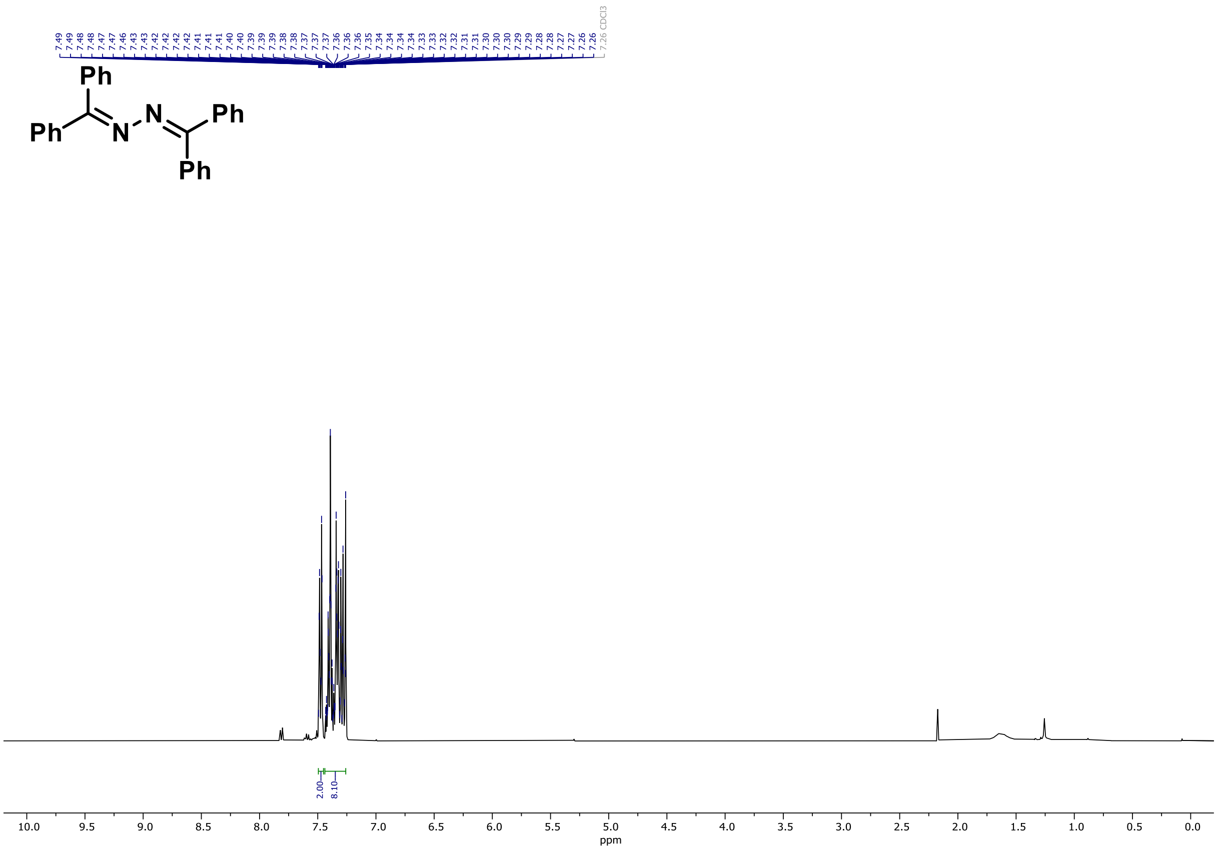

^{13}C{^{1}H} NMR ($CDCl_3$, 101 MHz) for **7a**

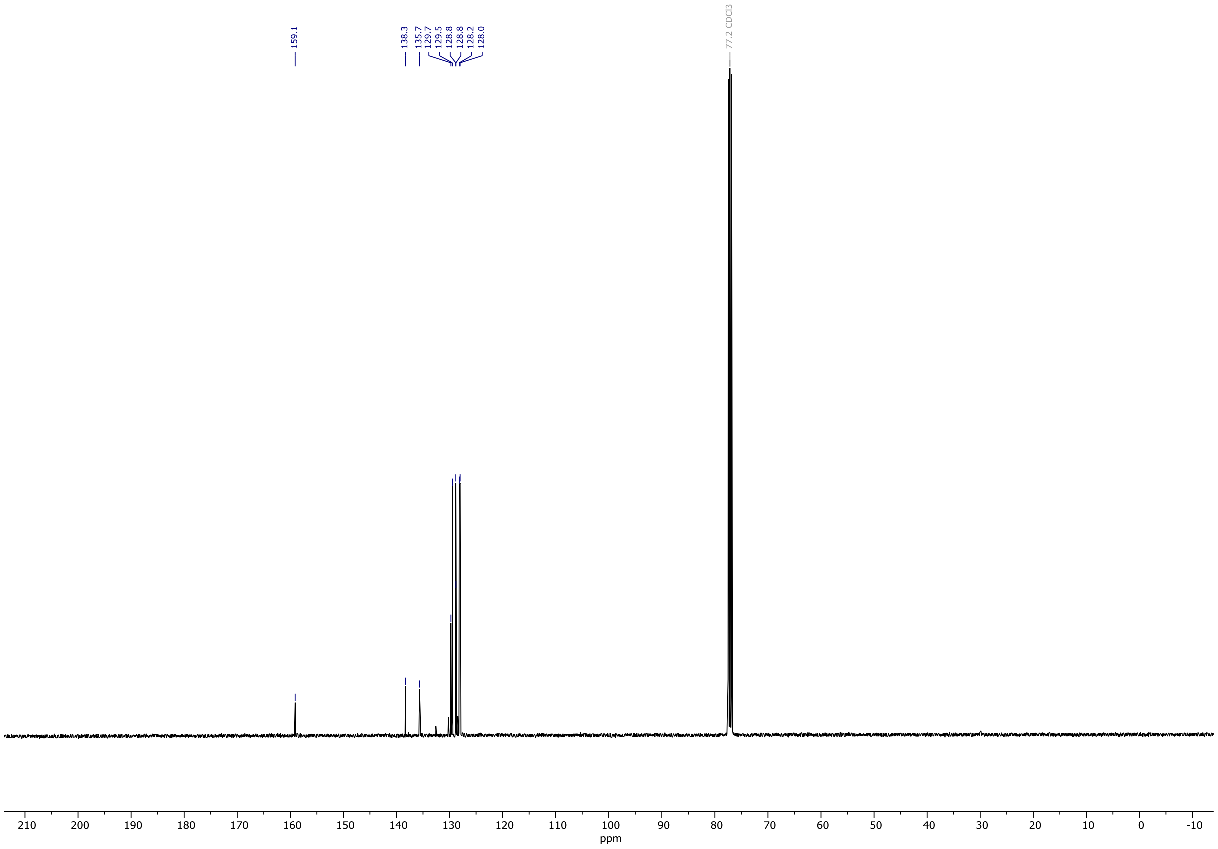

^{1}H NMR (CDCl$_3$, 400 MHz) for **S5**

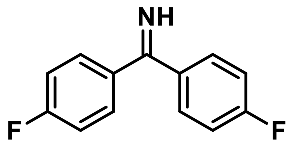

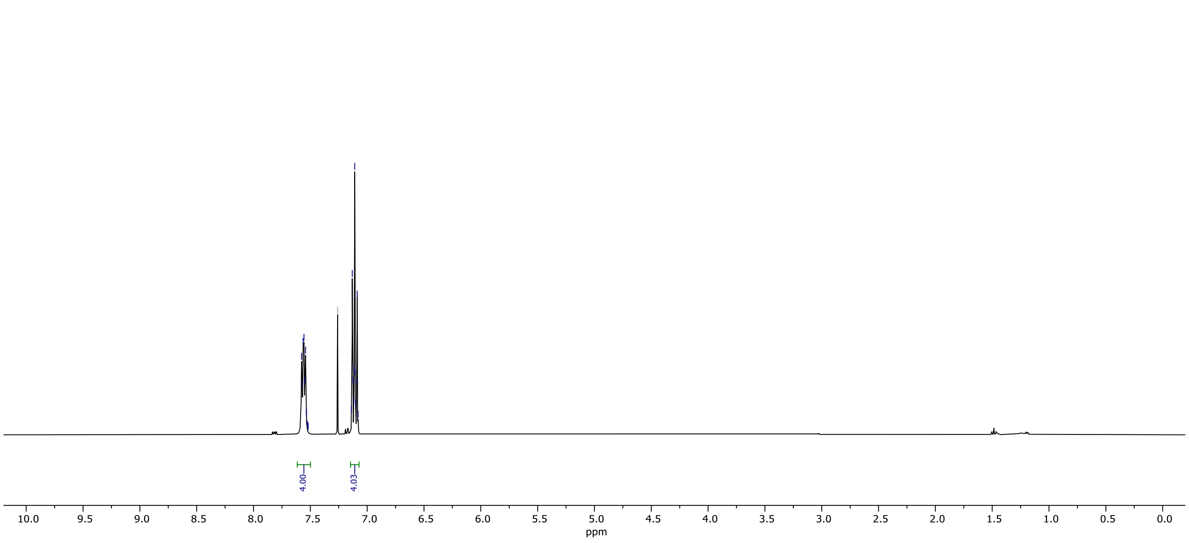

^{13}C{^{1}H} NMR (CDCl$_3$, 101 MHz) for **S5**

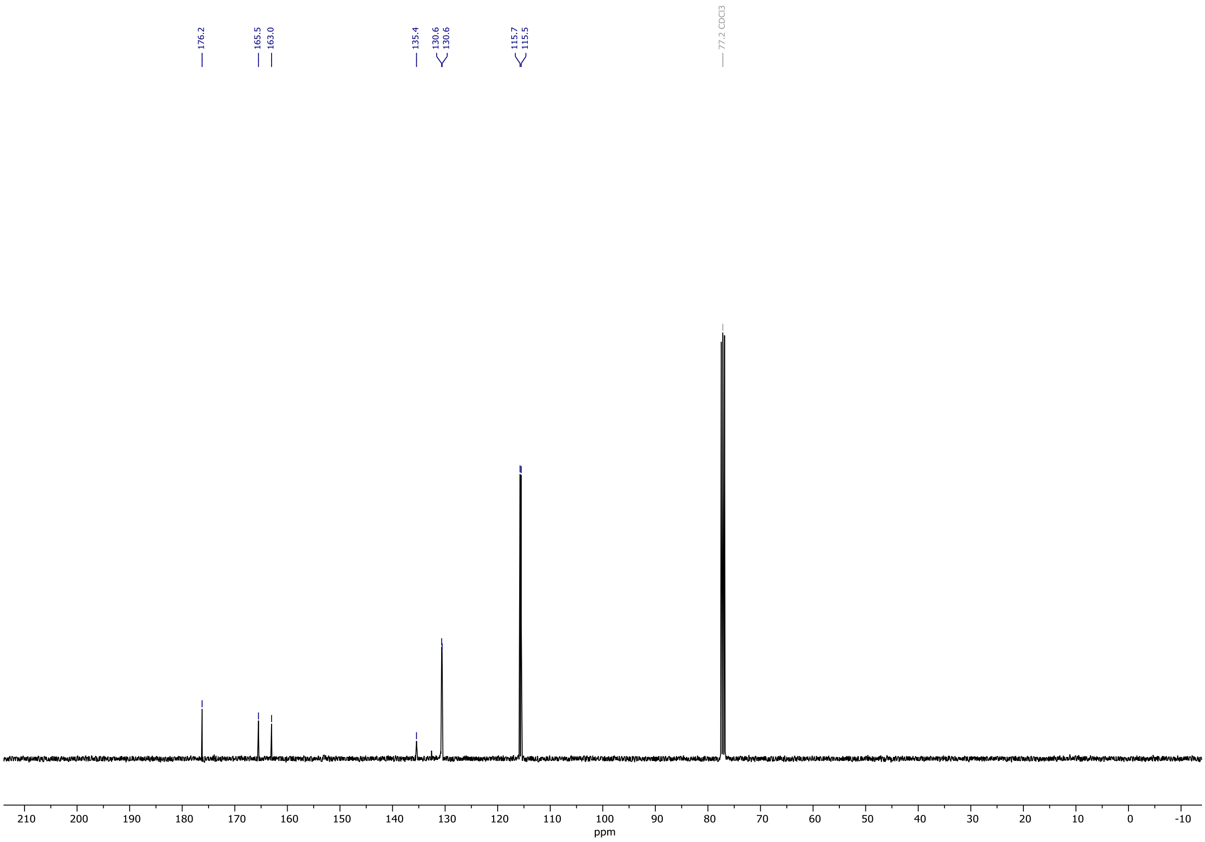

^{19}**F NMR** ($CDCl_3$, 376 MHz) for **S5**

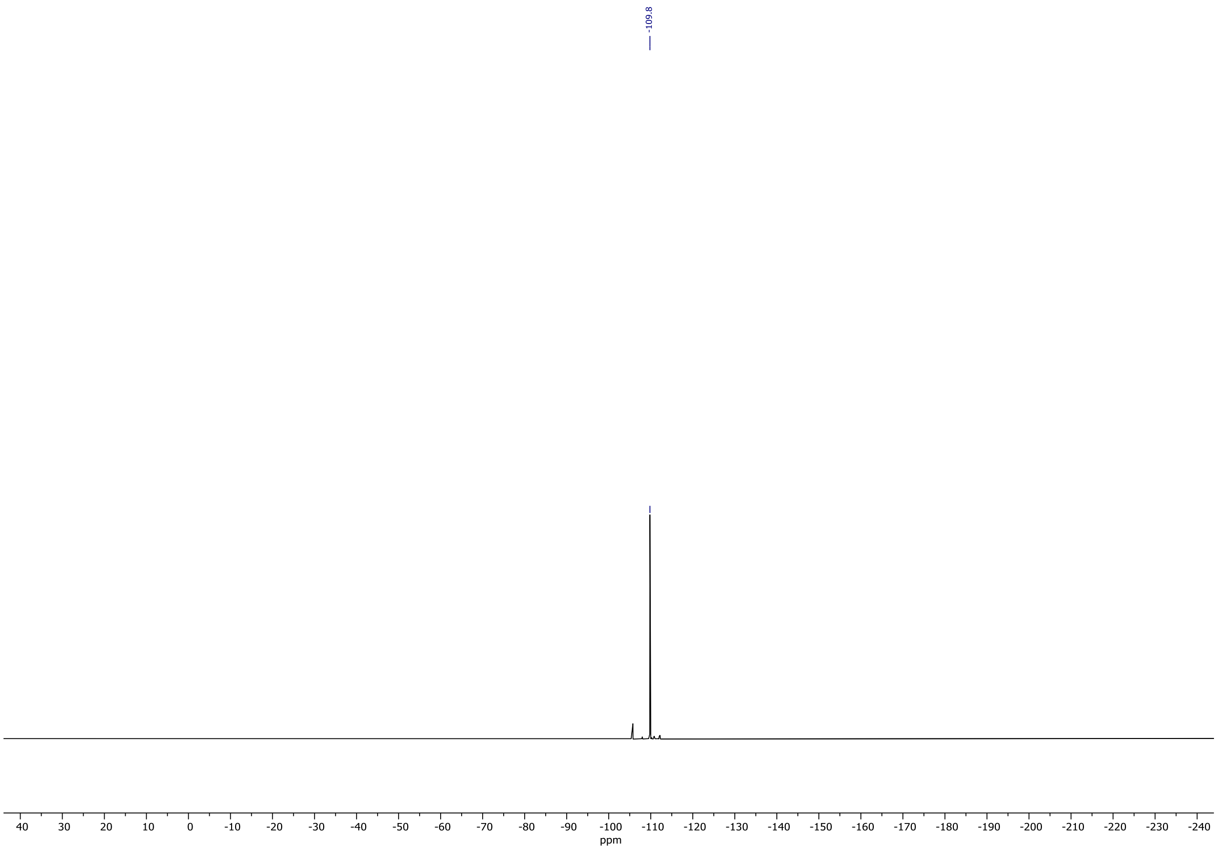

^{1}H NMR (CDCl$_3$, 400 MHz) for **7b**

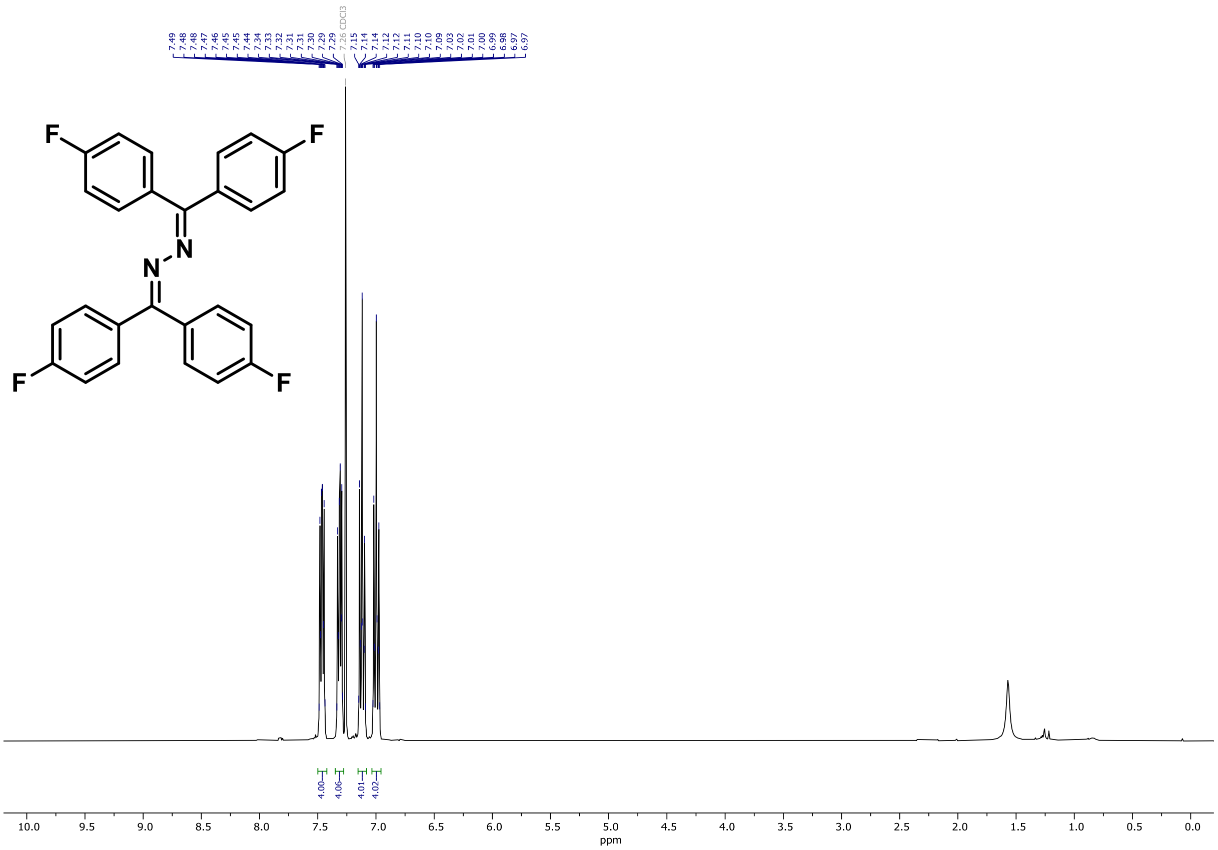

^{13}C{^{1}H} NMR (CDCl$_3$, 101 MHz) for **7b**

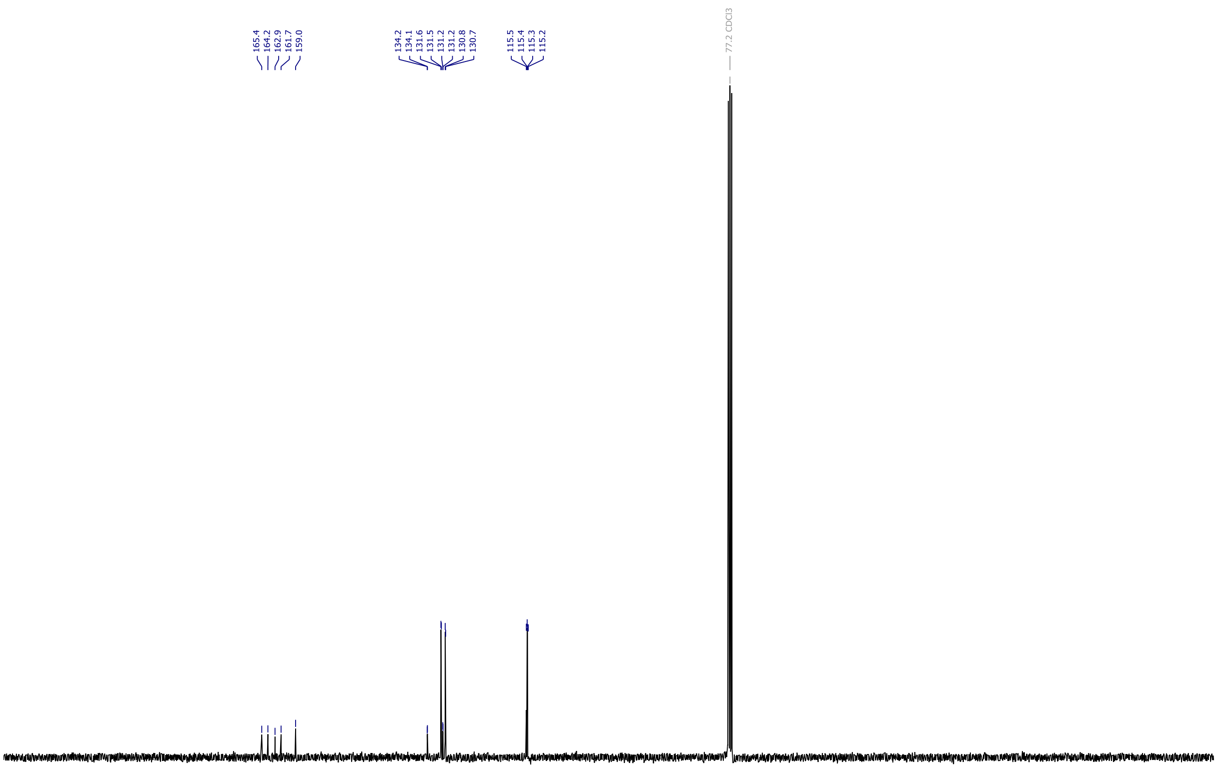

$^{19}F\{^{1}H\}$ NMR ($CDCl_3$, 376 MHz) for **7b**

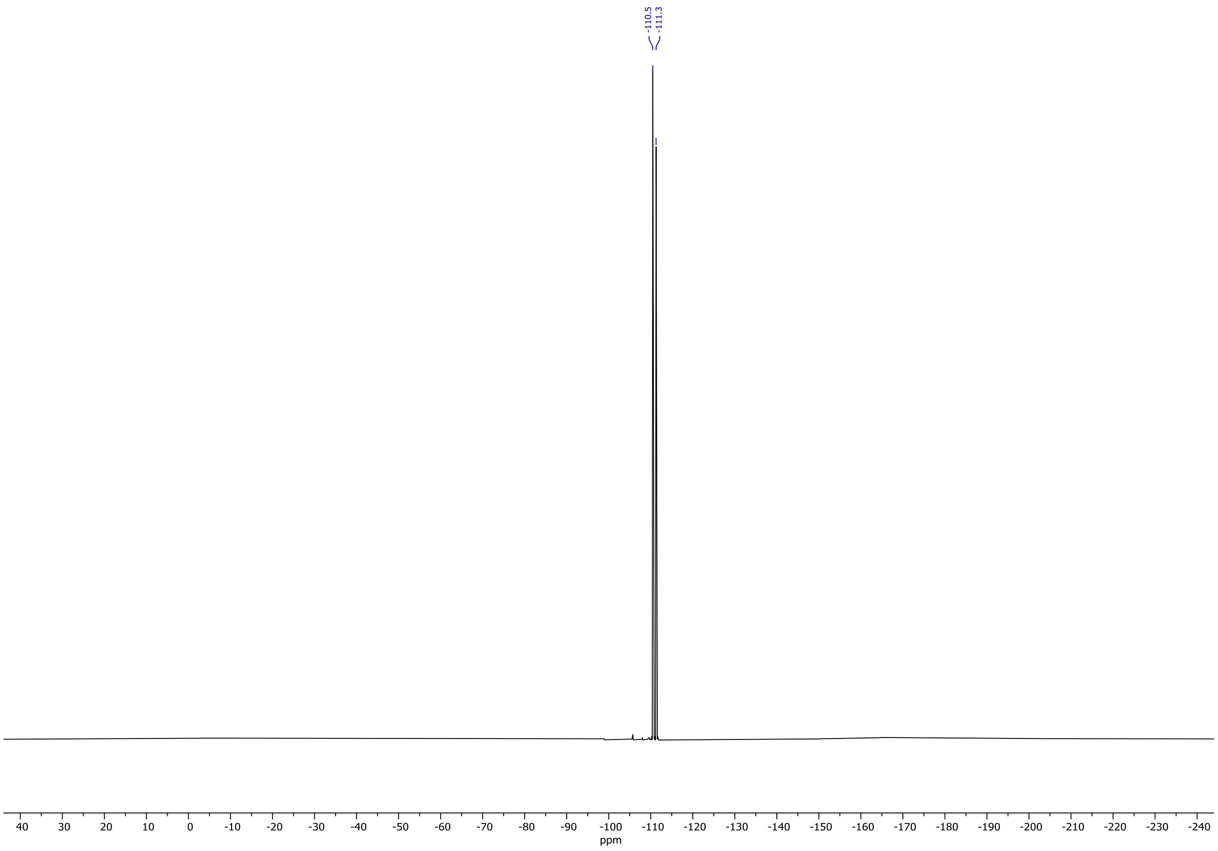

¹H NMR (CDCl$_3$, 599 MHz) for **8**

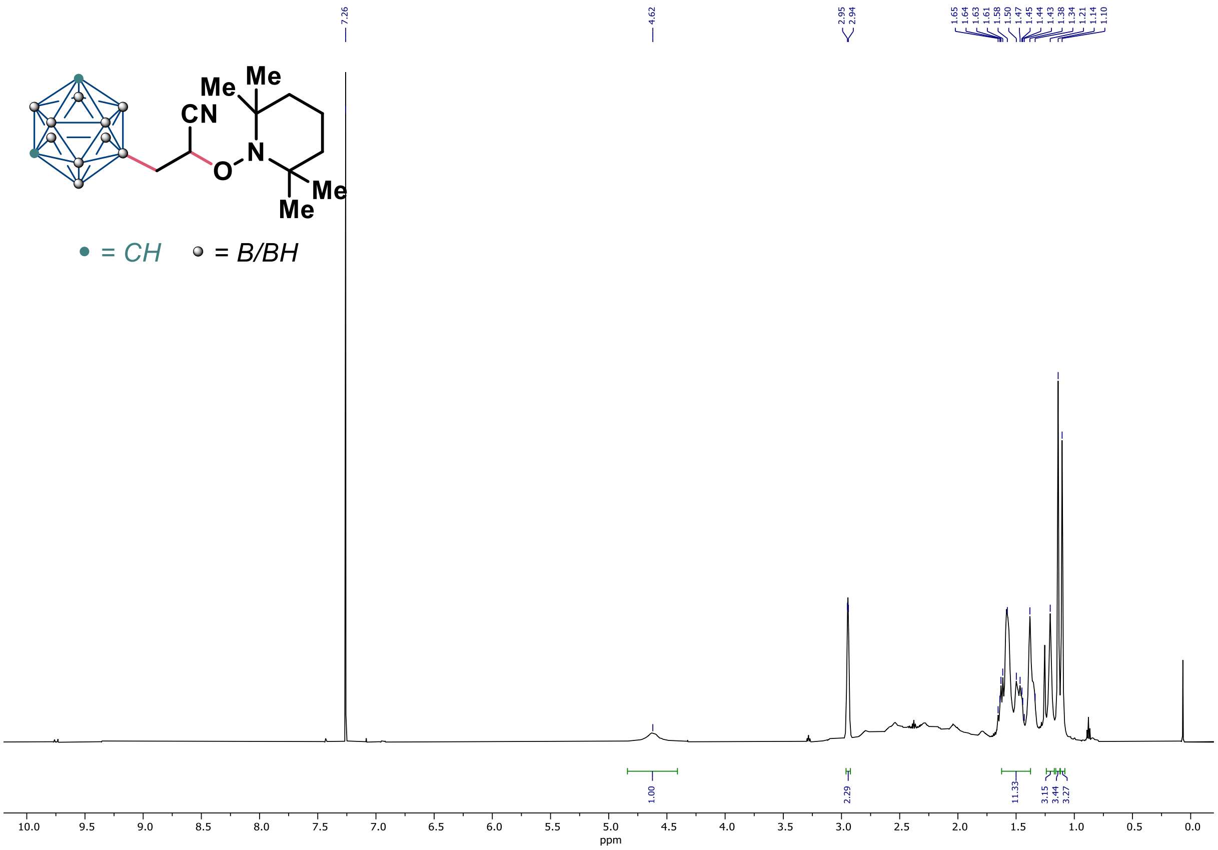

¹³C{¹H} NMR (CDCl$_3$, 151 MHz) for **8**

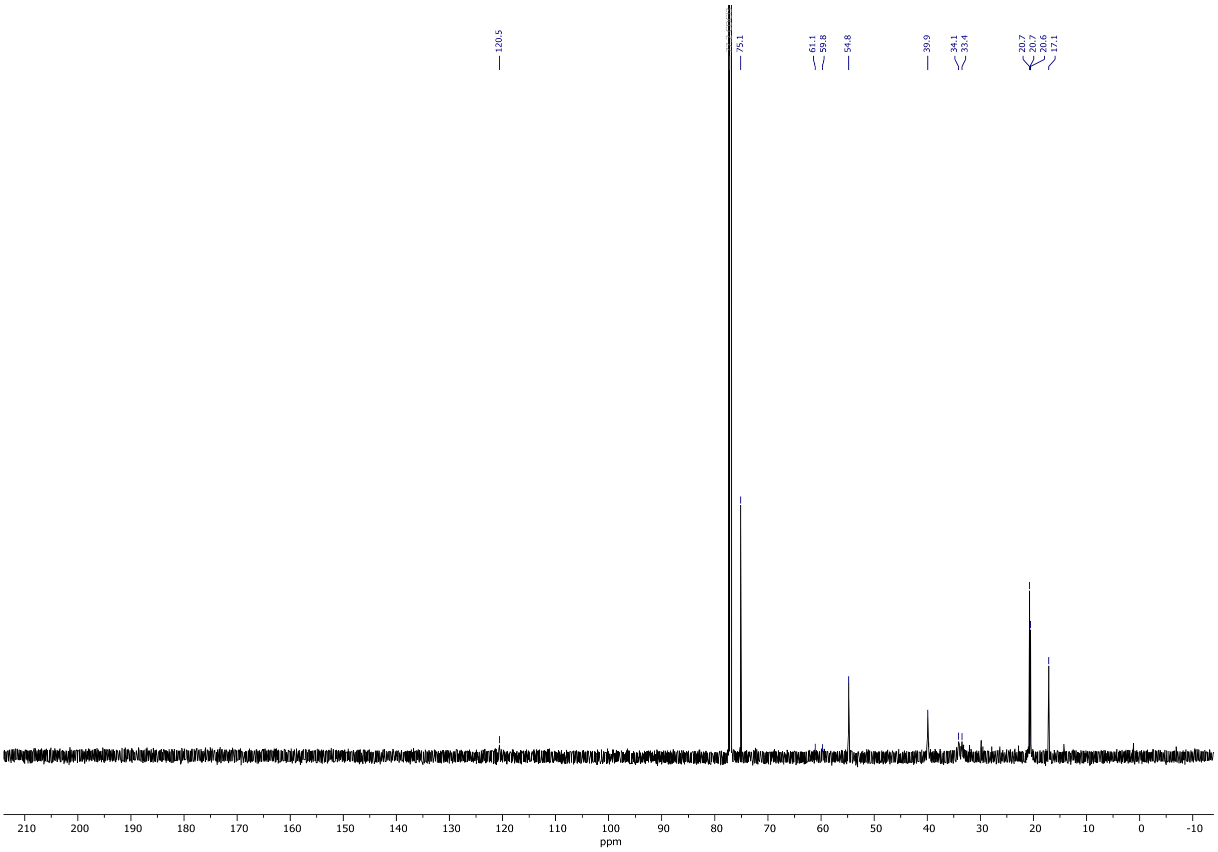

$^{11}B\{^1H\}$ NMR ($CDCl_3$, 128 MHz) for **8**

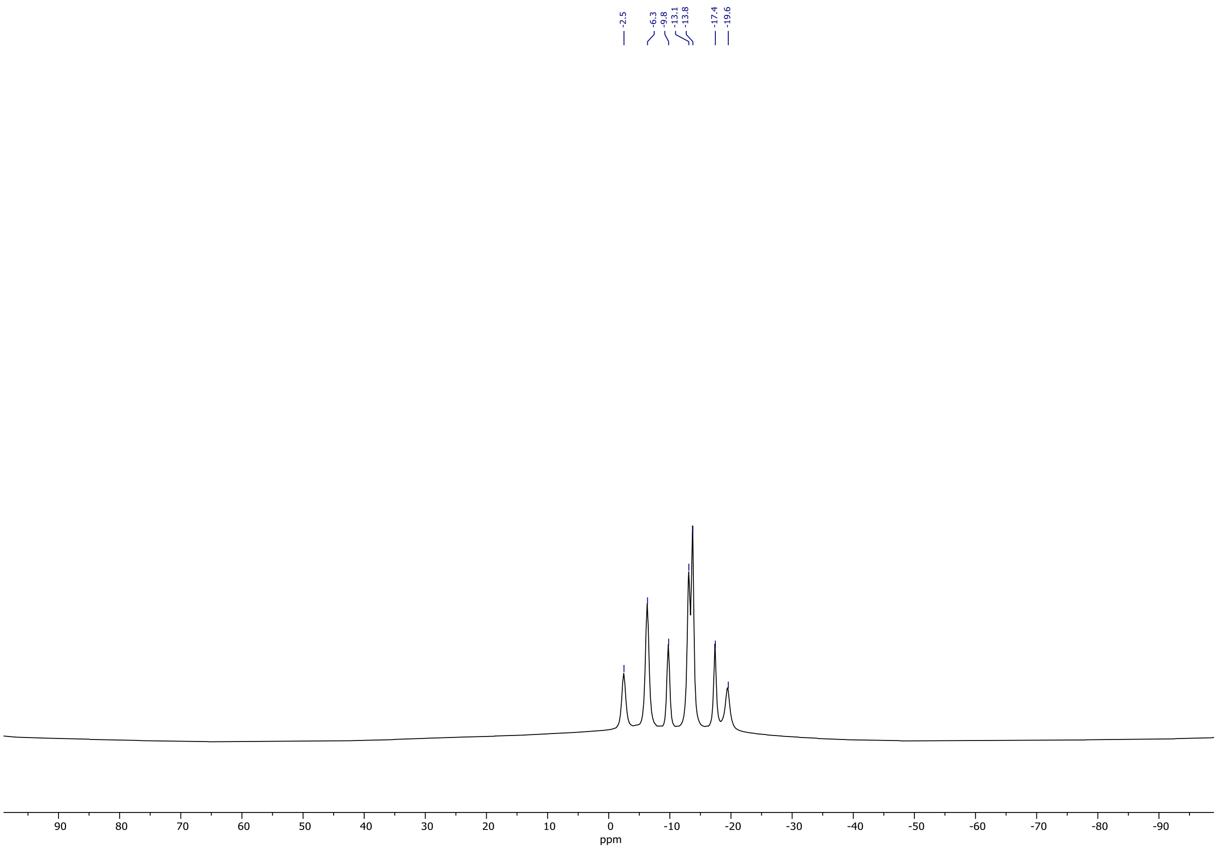

8.4 Product diversifications

^{1}H NMR (D_2O, 400 MHz) for **10**

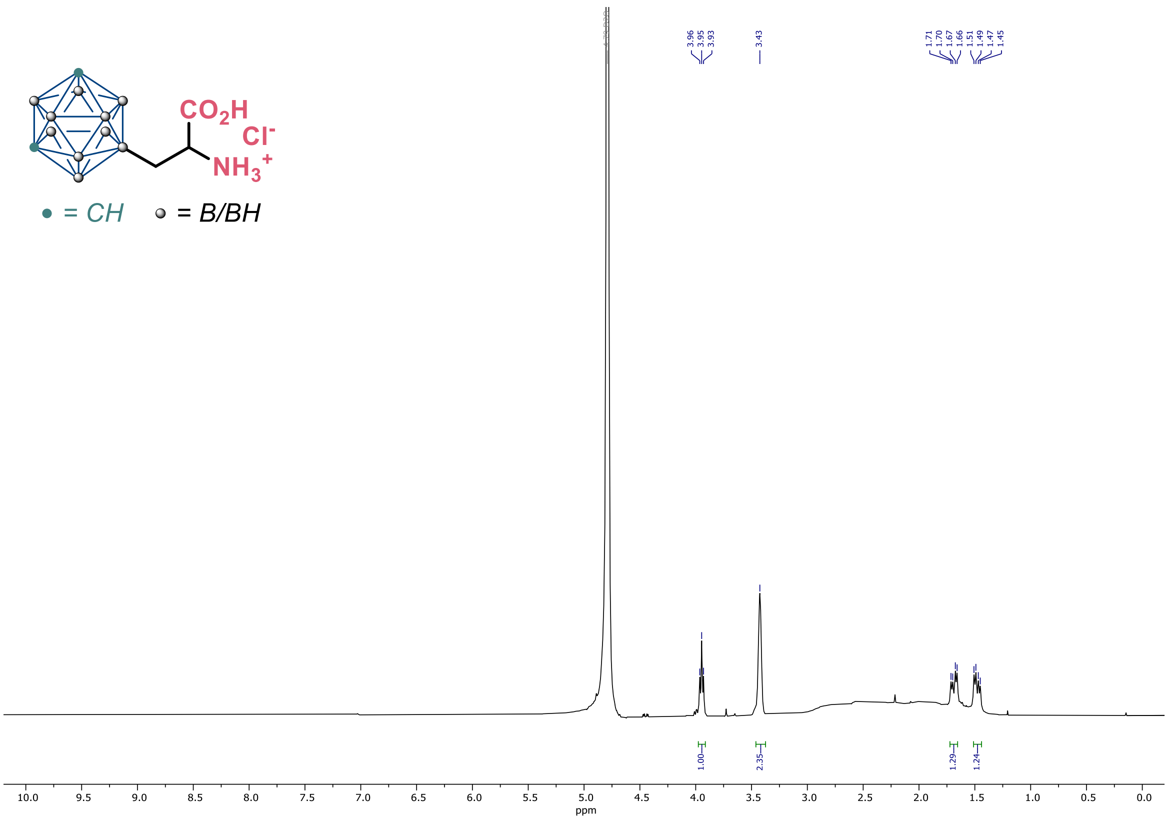

^{13}C{^{1}H} NMR (D_2O, 101 MHz) for **10**

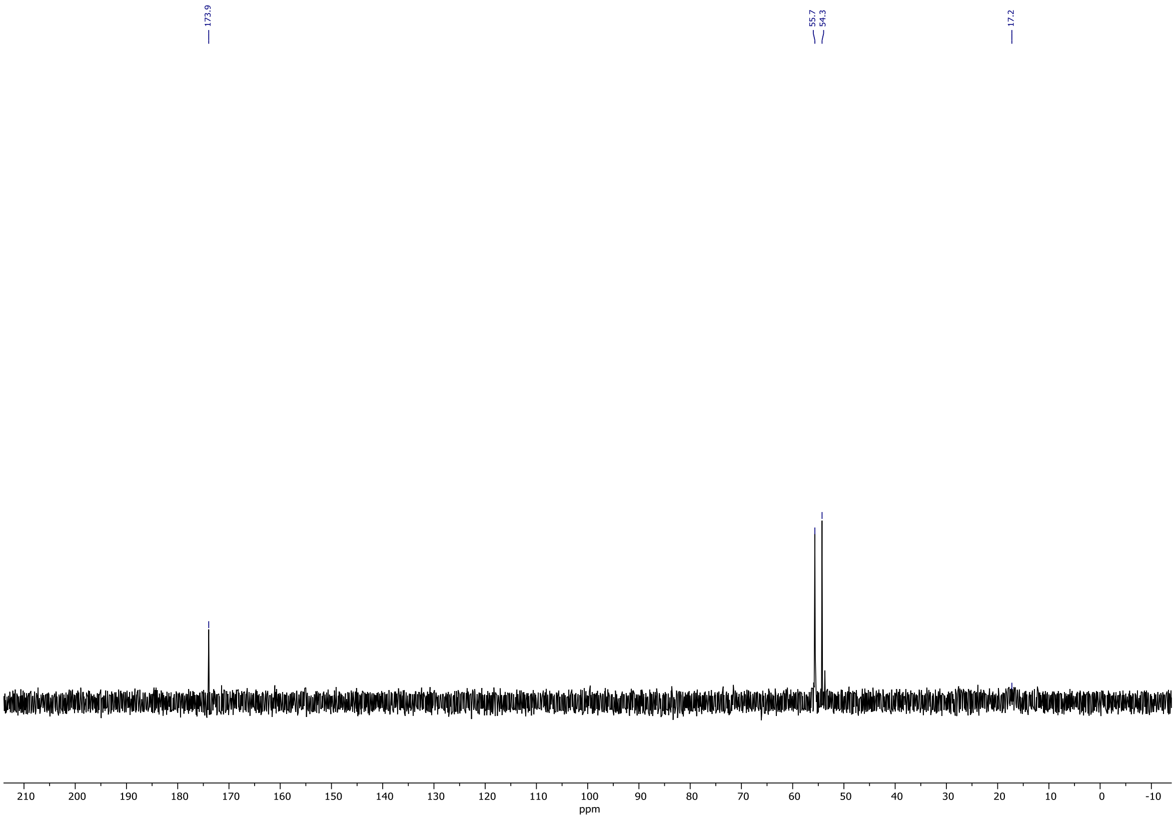

^{11}B{^{1}H} NMR (D_2O, 128 MHz) for **10**

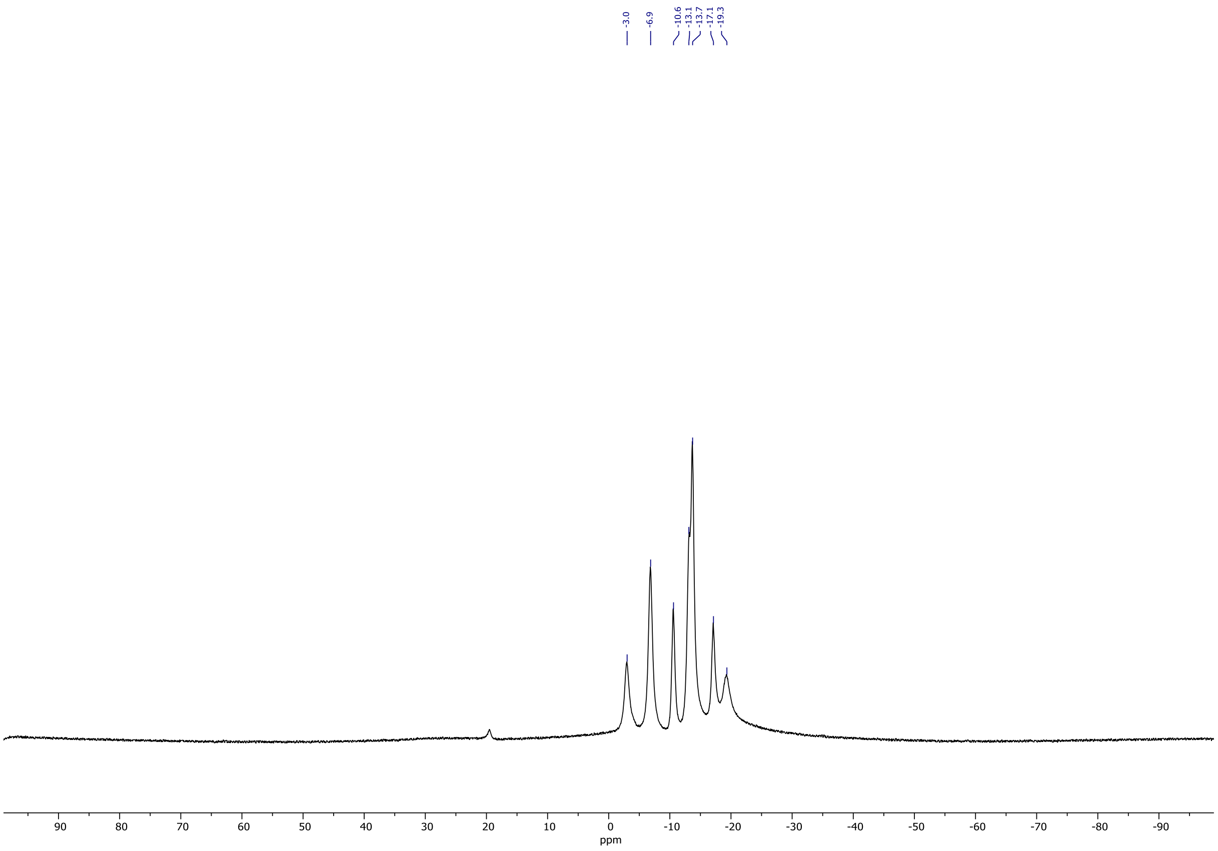

^{1}H NMR (MeOH-d$_4$, 400 MHz) for **11**

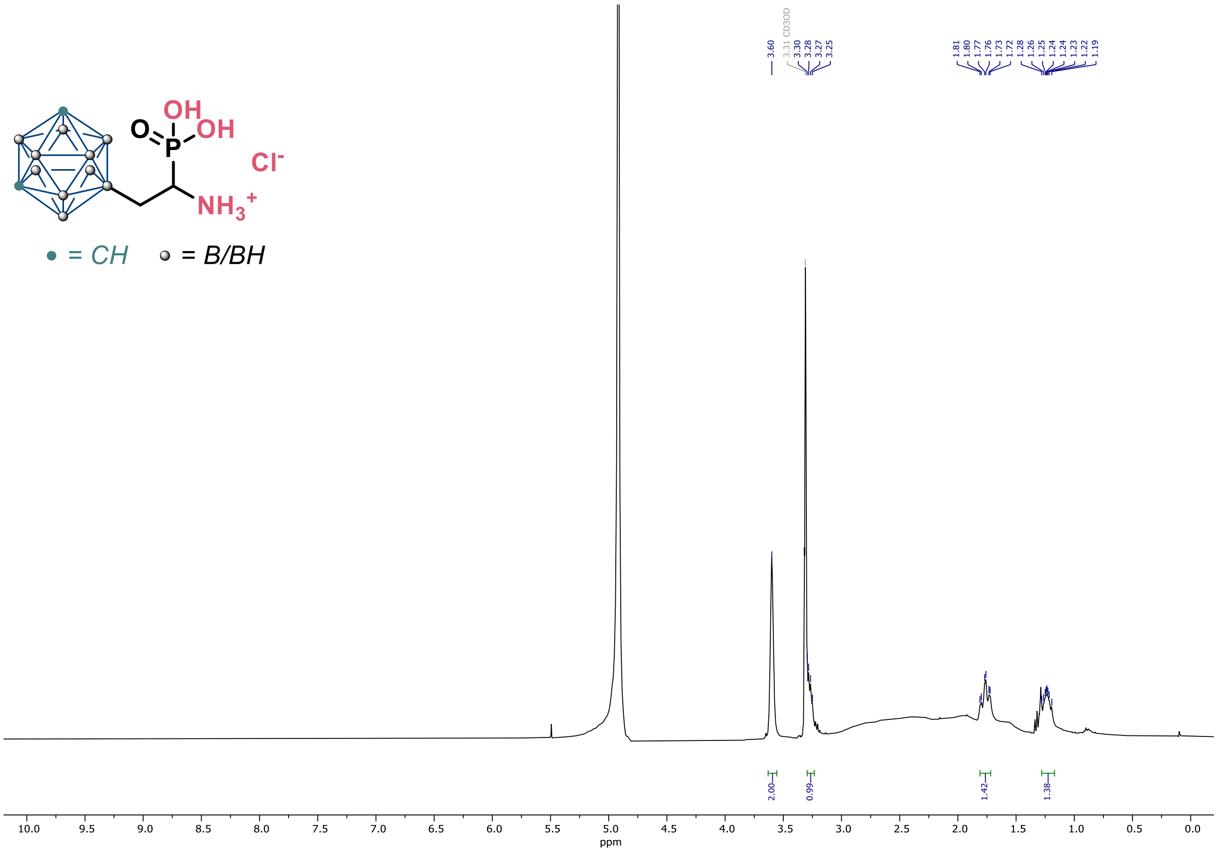

^{13}C{^{1}H} NMR (MeOH-d$_4$, 151 MHz) for **11**

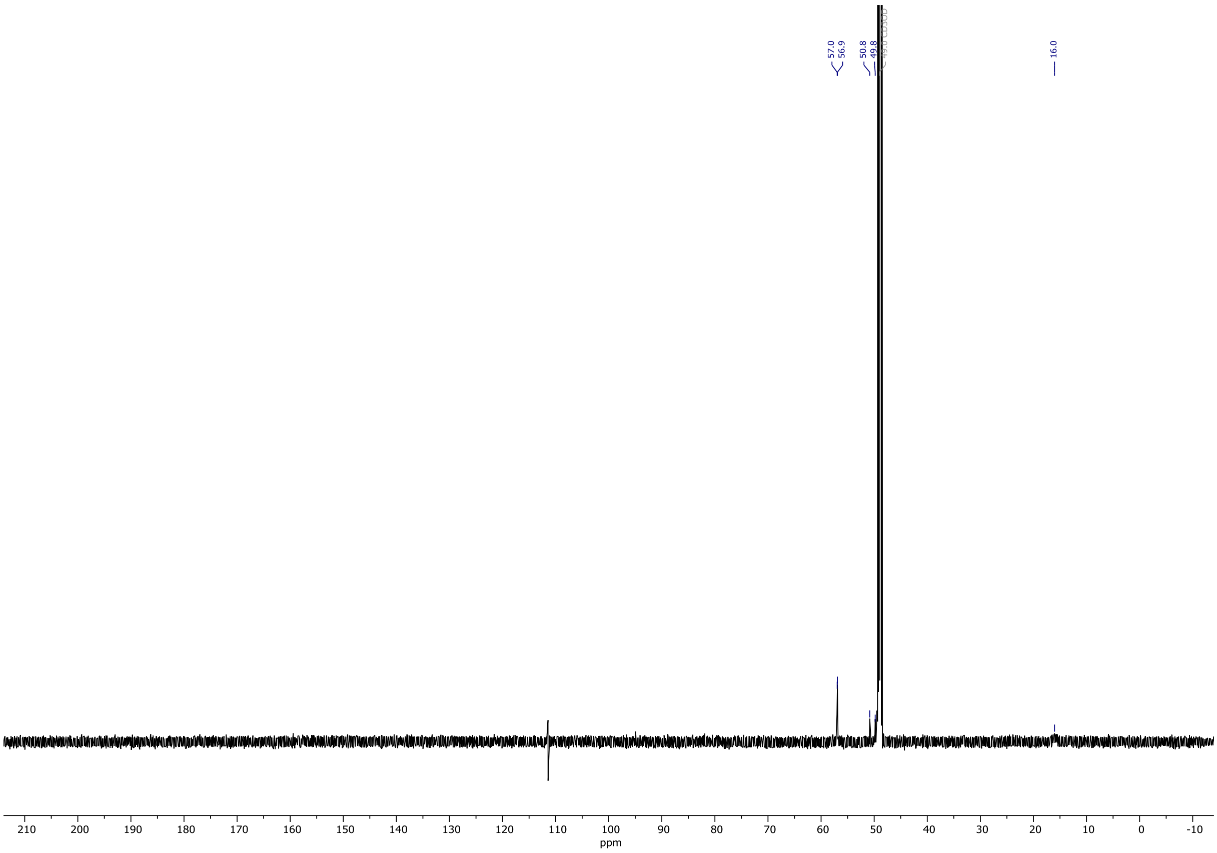

$^{11}B\{^{1}H\}$ NMR (MeOH-d$_4$, 128 MHz) for **11**

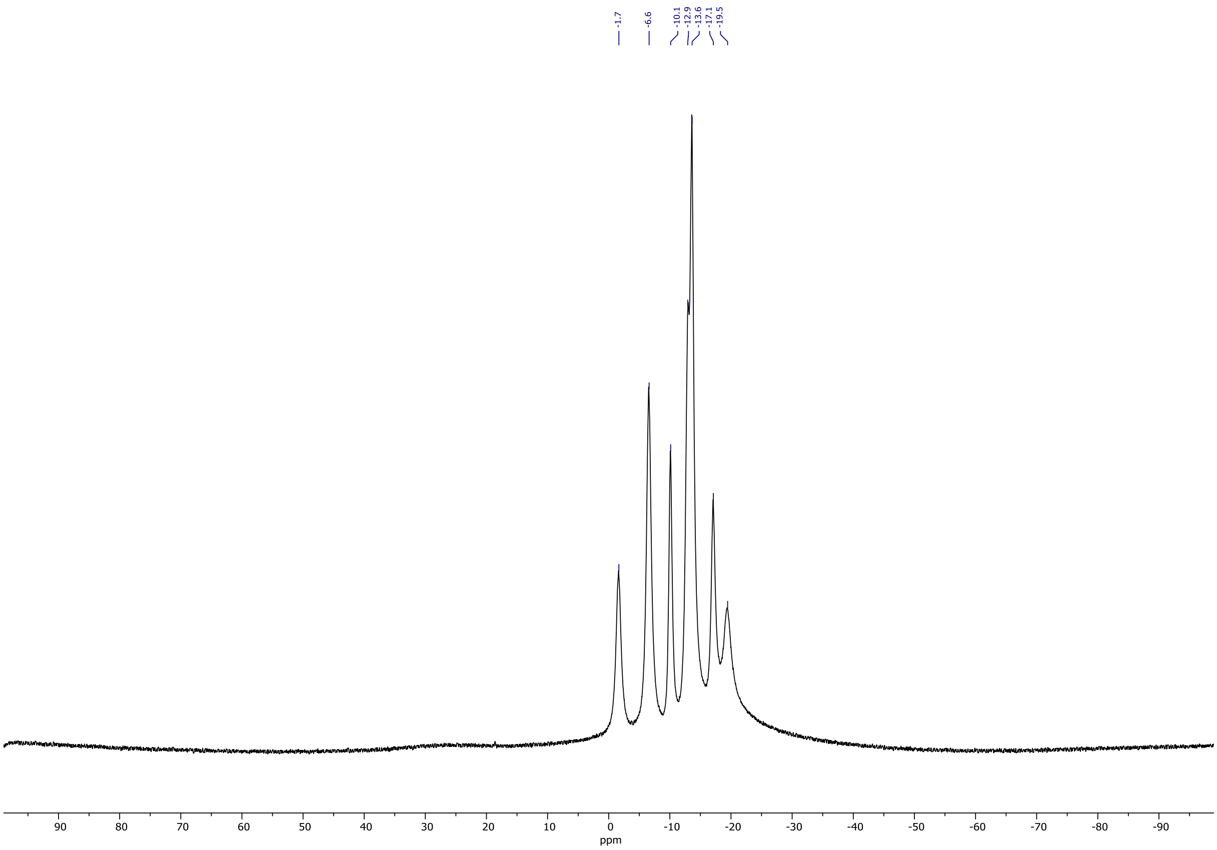

^{31}P NMR (MeOH-d$_4$, 162 MHz) for **11**

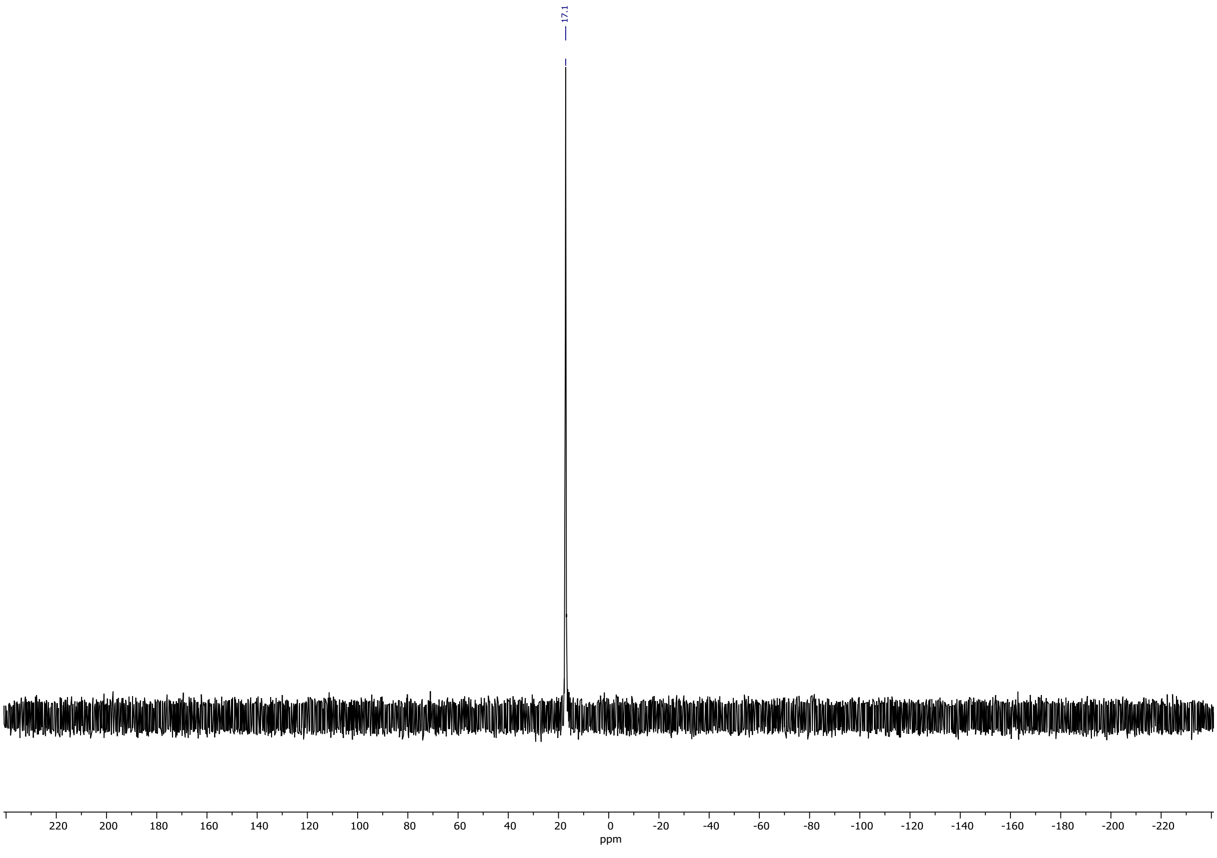

^{1}H NMR ($CDCl_3$, 400 MHz) for **5n’**

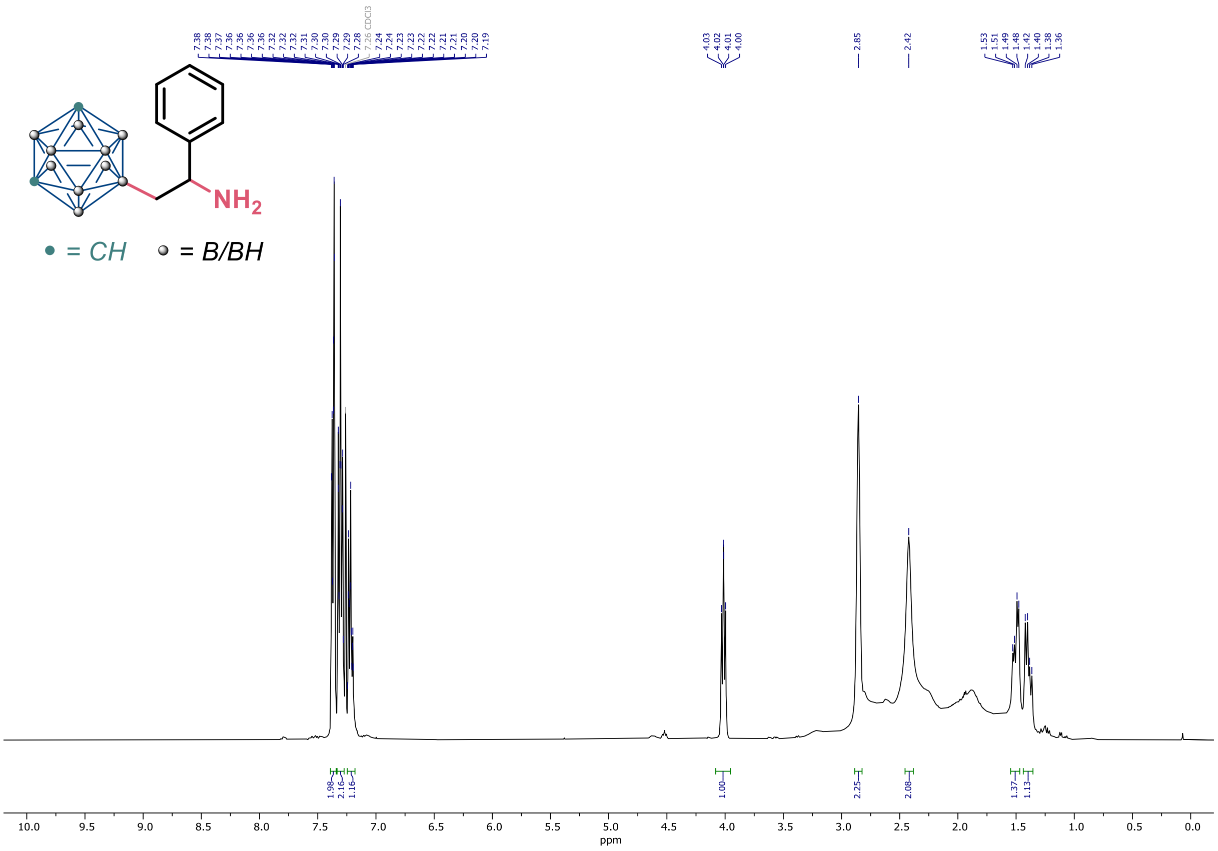

^{13}C{^{1}H} NMR ($CDCl_3$, 101 MHz) for **5n’**

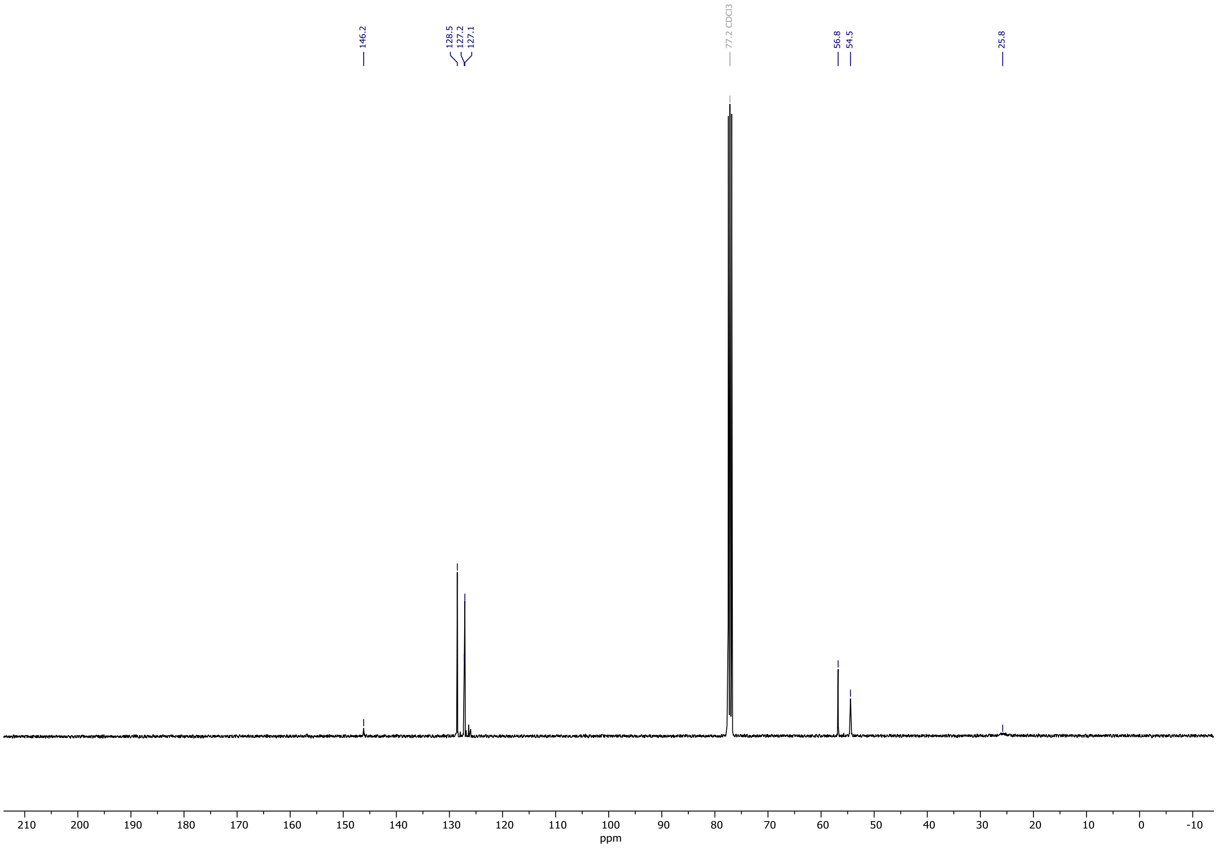

$^{11}B\{^1H\}$ NMR ($CDCl_3$, 128 MHz) for **5n'**

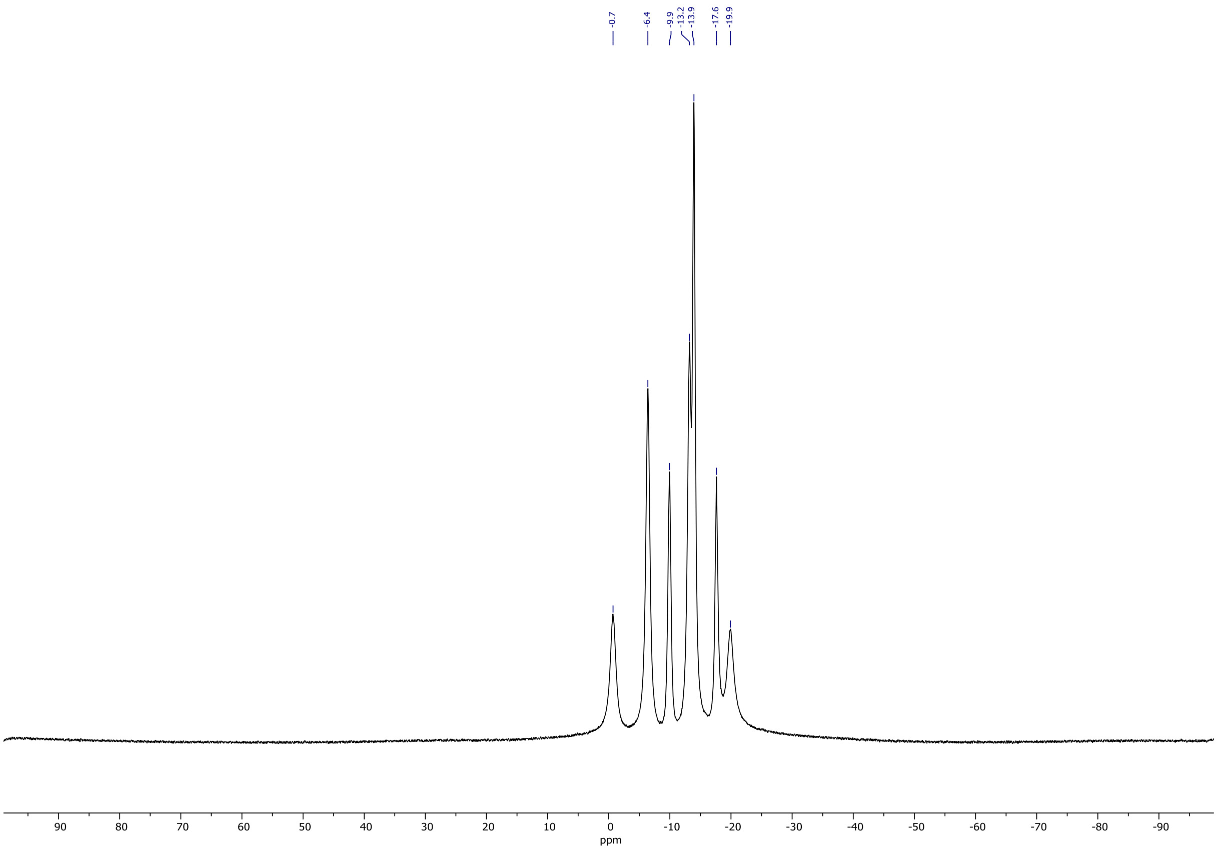

^{1}H NMR ($CDCl_3$, 400 MHz) for **12**

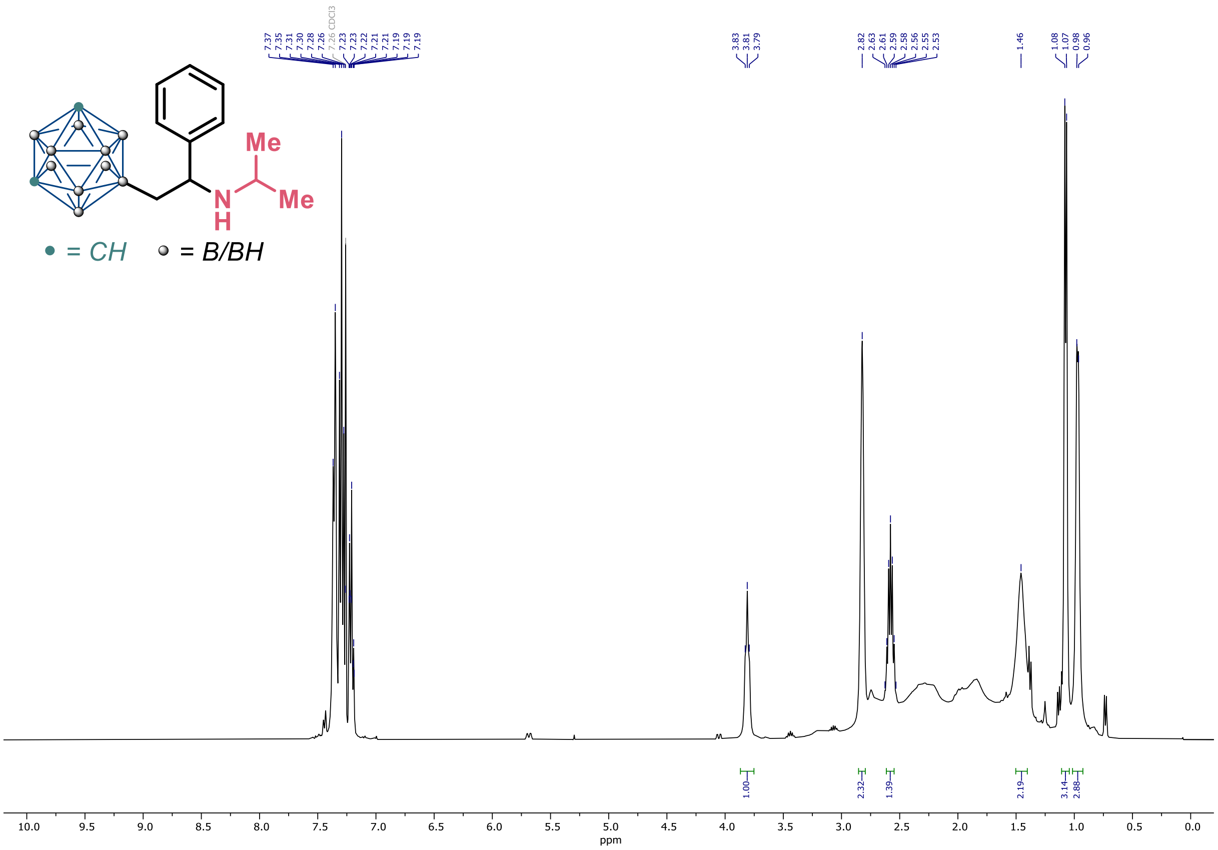

^{13}C{^{1}H} NMR ($CDCl_3$, 126 MHz) for **12**

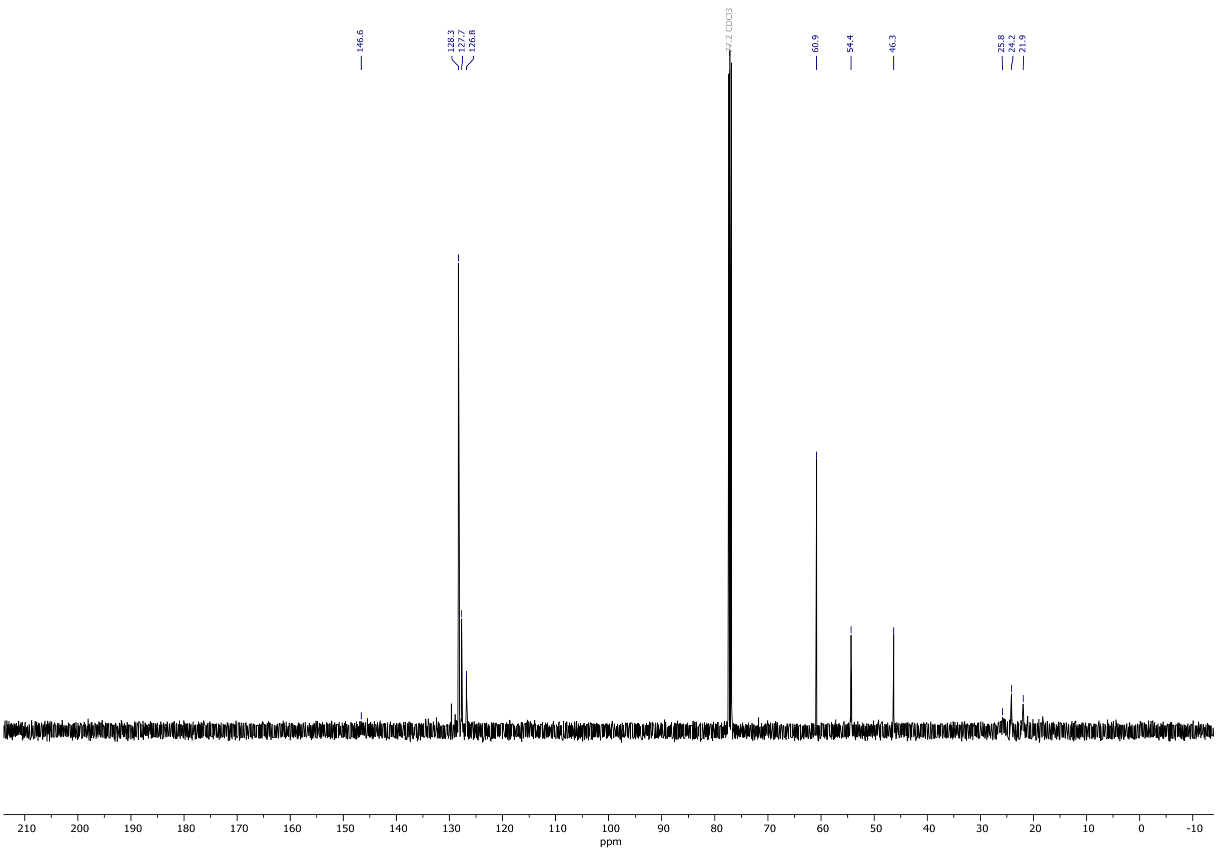

^{11}B NMR (CDCl$_3$, 128 MHz) for **12**

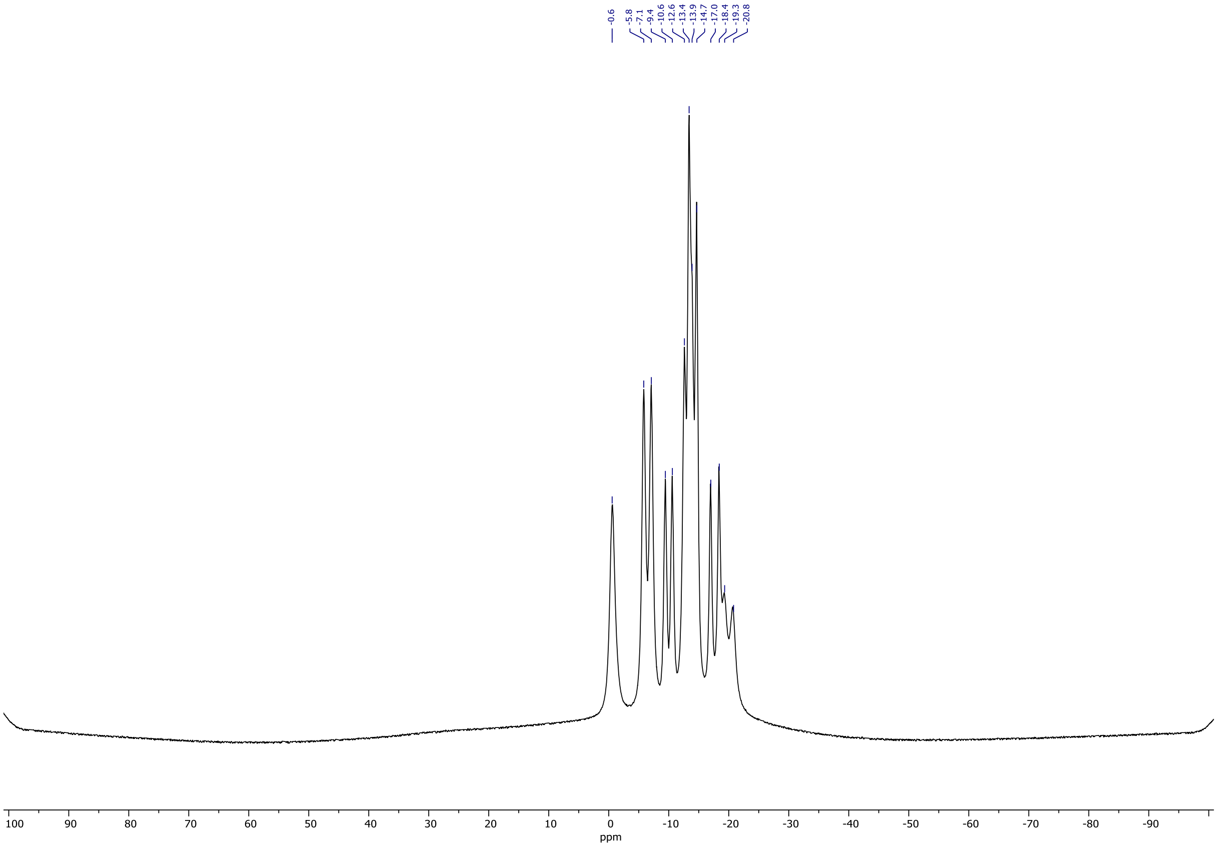

^{1}H NMR (CDCl$_3$, 400 MHz) for **13**

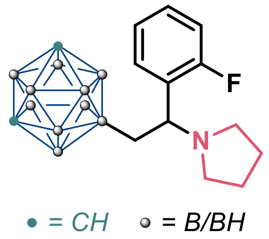

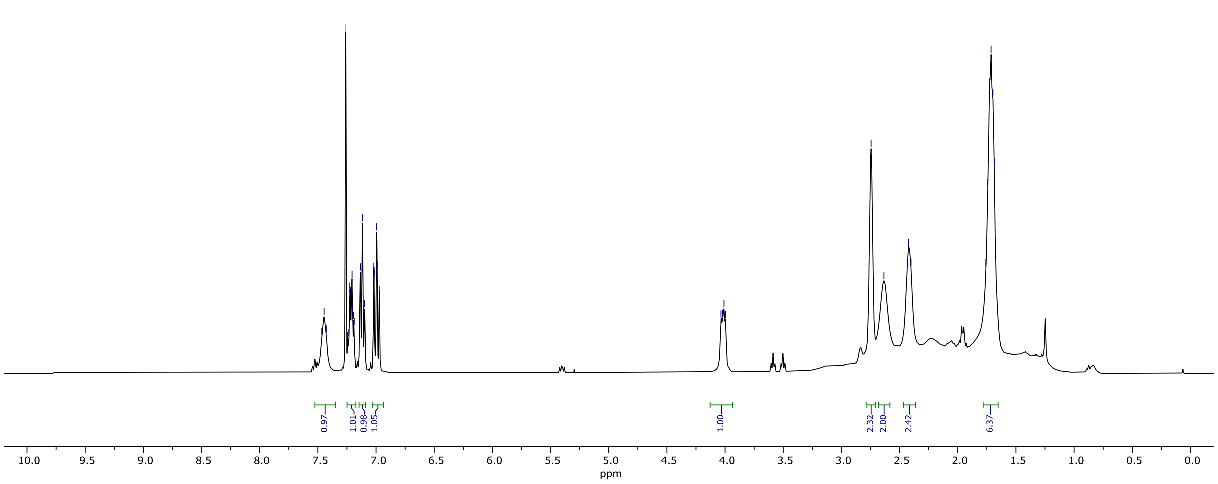

^{13}C{^{1}H} NMR (CDCl$_3$, 101 MHz) for **13**

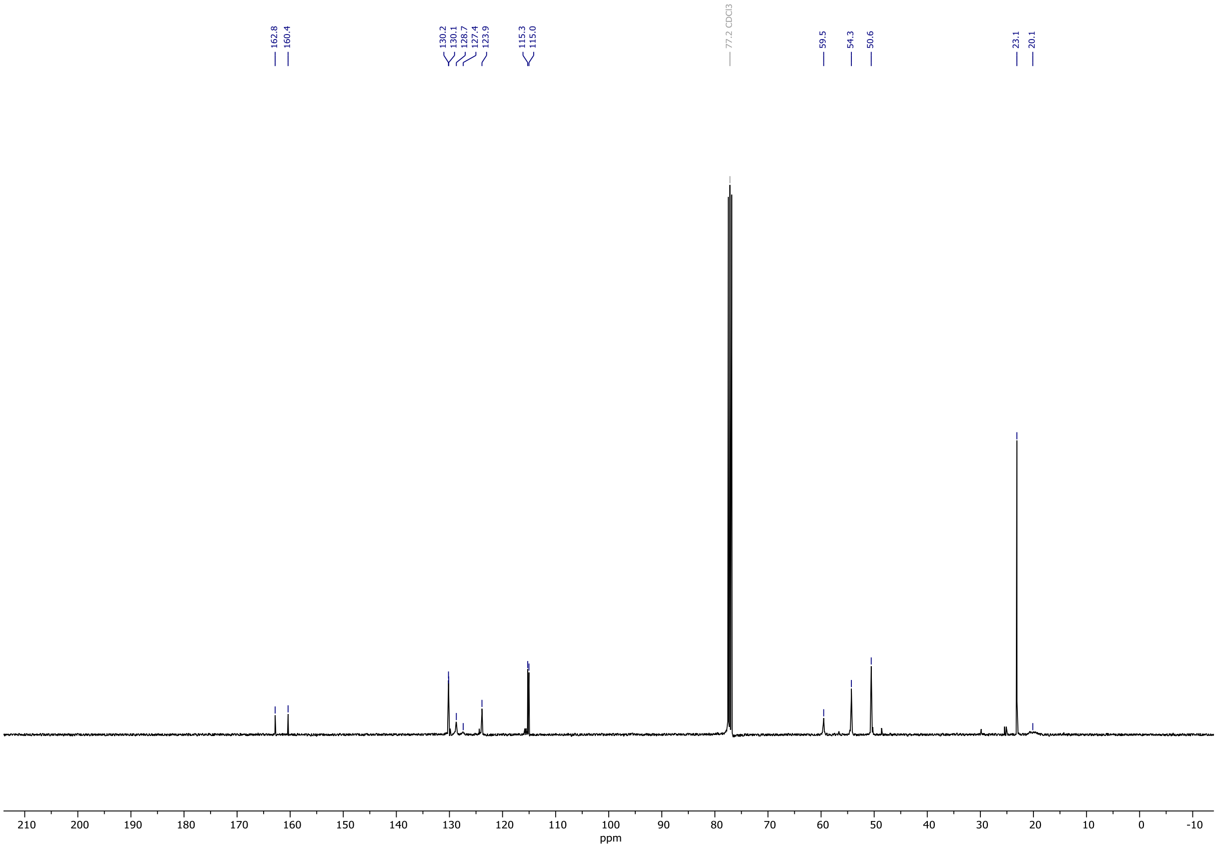

^{11}B{^{1}H} NMR (CDCl$_3$, 128 MHz) for **13**

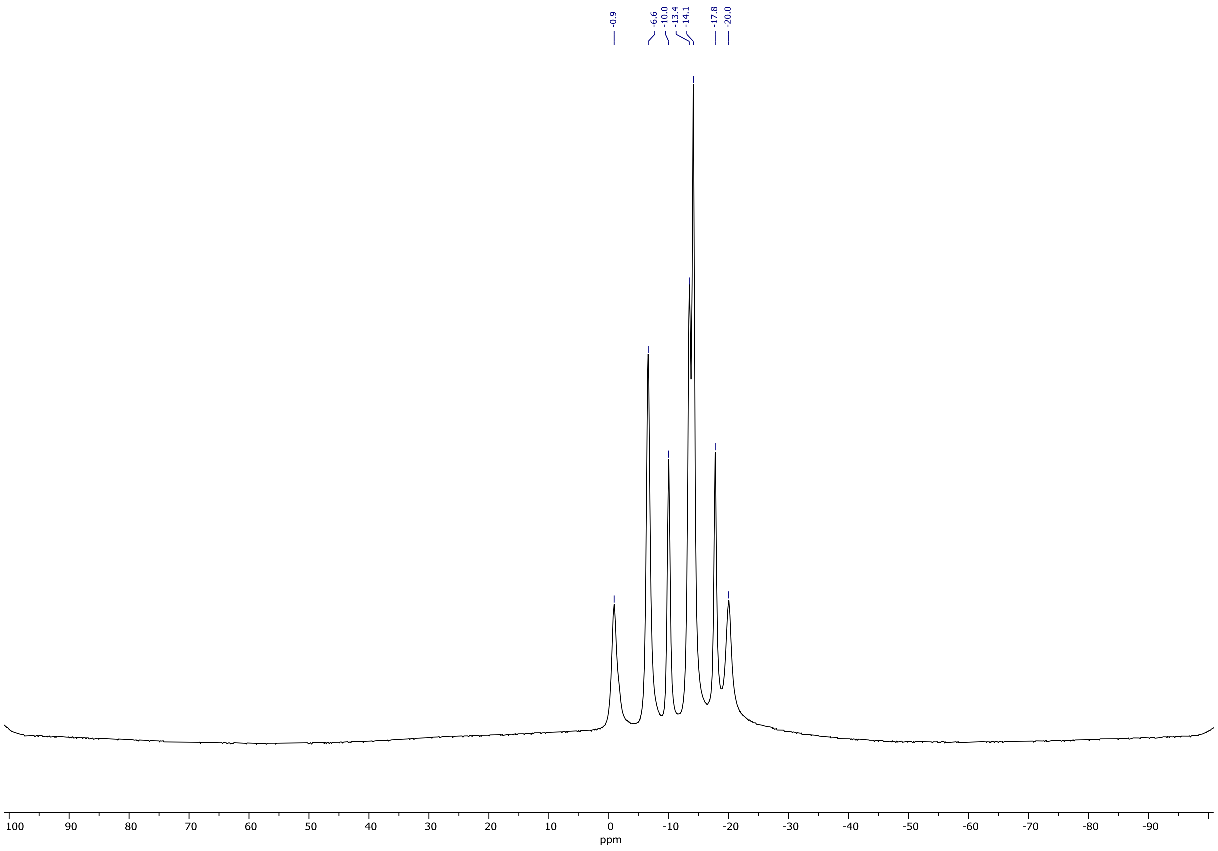

^{19}F{^{1}H} NMR (CDCl$_3$, 376 MHz) for **13**

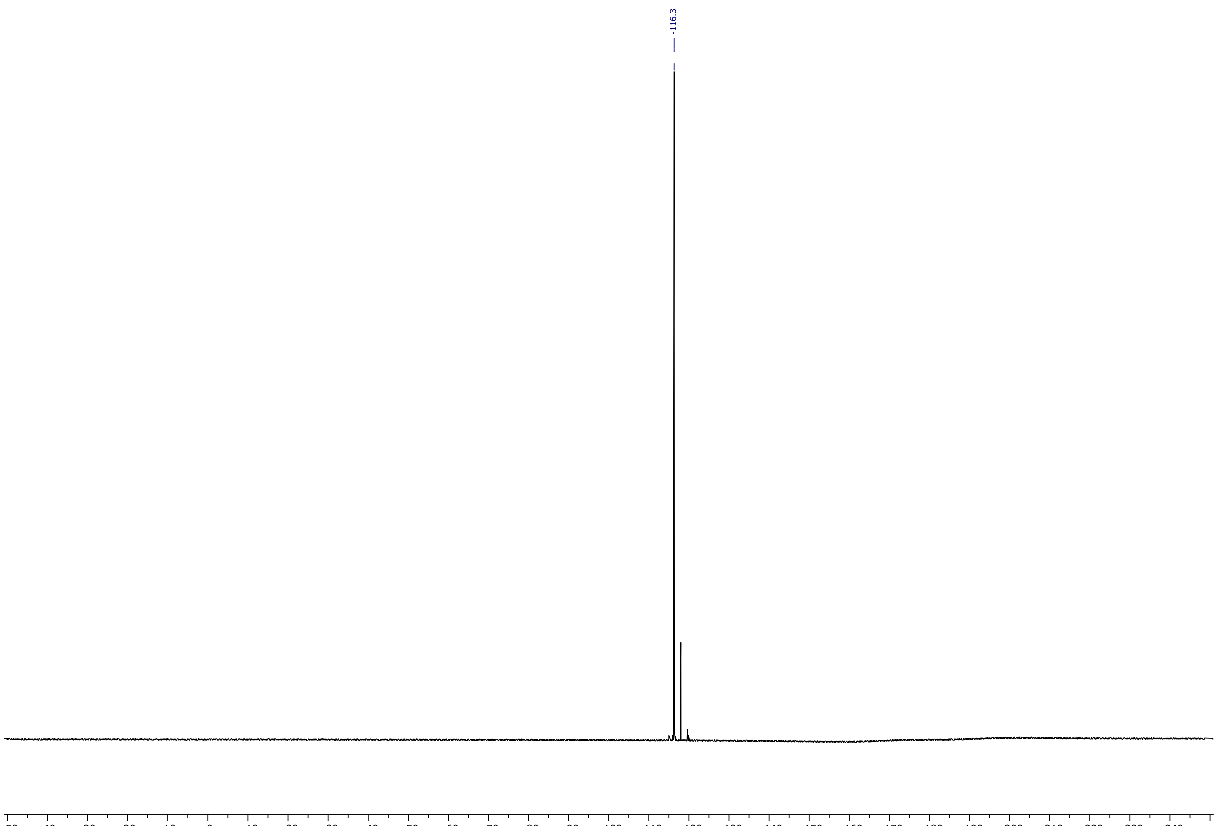

^{1}H NMR ($CDCl_3$, 400 MHz) for **5q’**

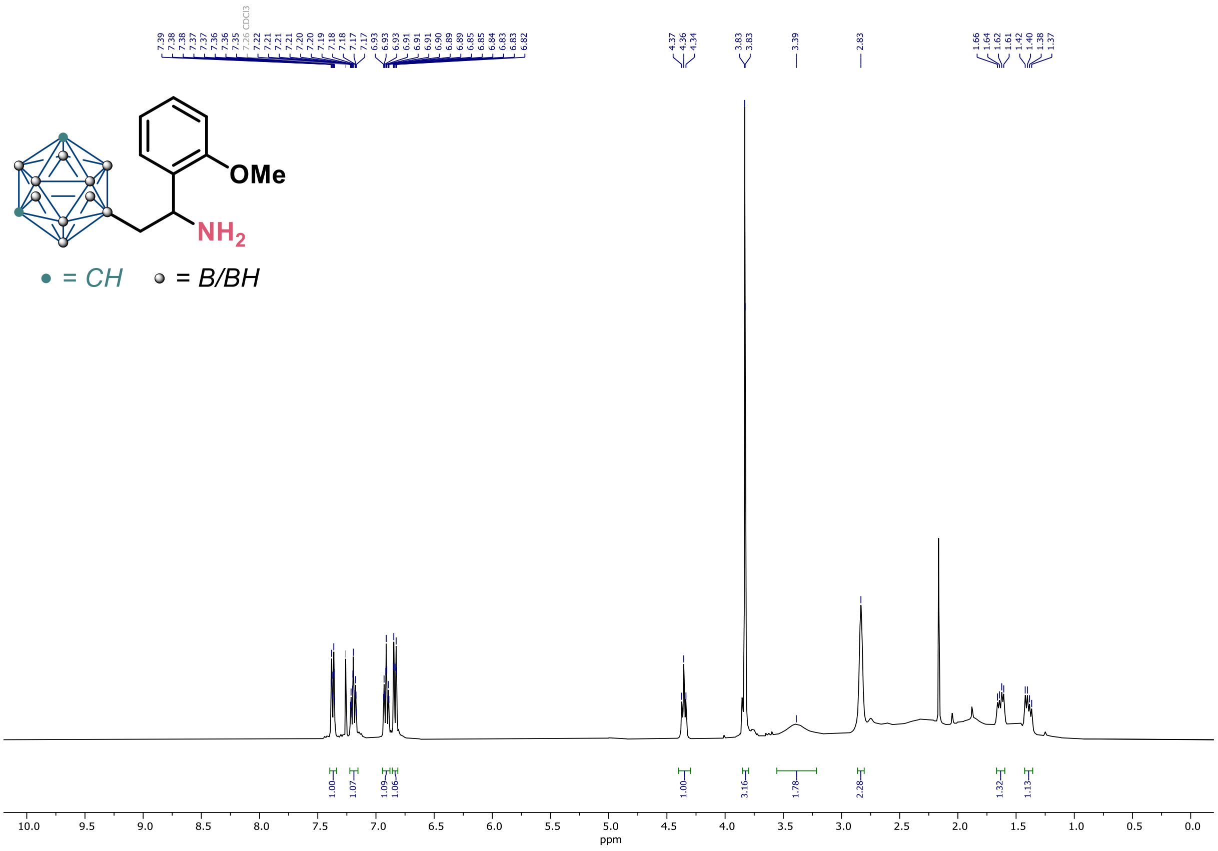

^{13}C{^{1}H} NMR ($CDCl_3$, 101 MHz) for **5q’**

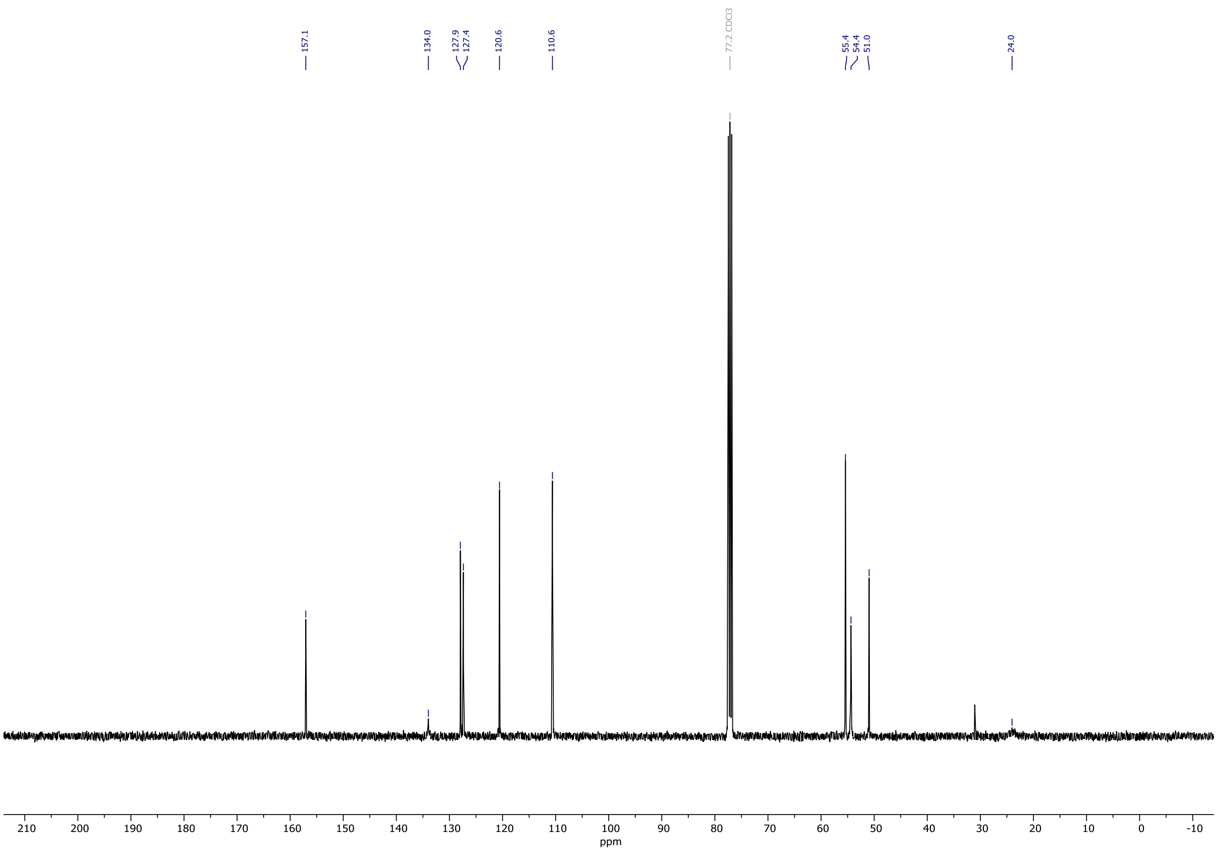

^{11}B{^{1}H} NMR (CDCl$_3$, 128 MHz) for **5q'**

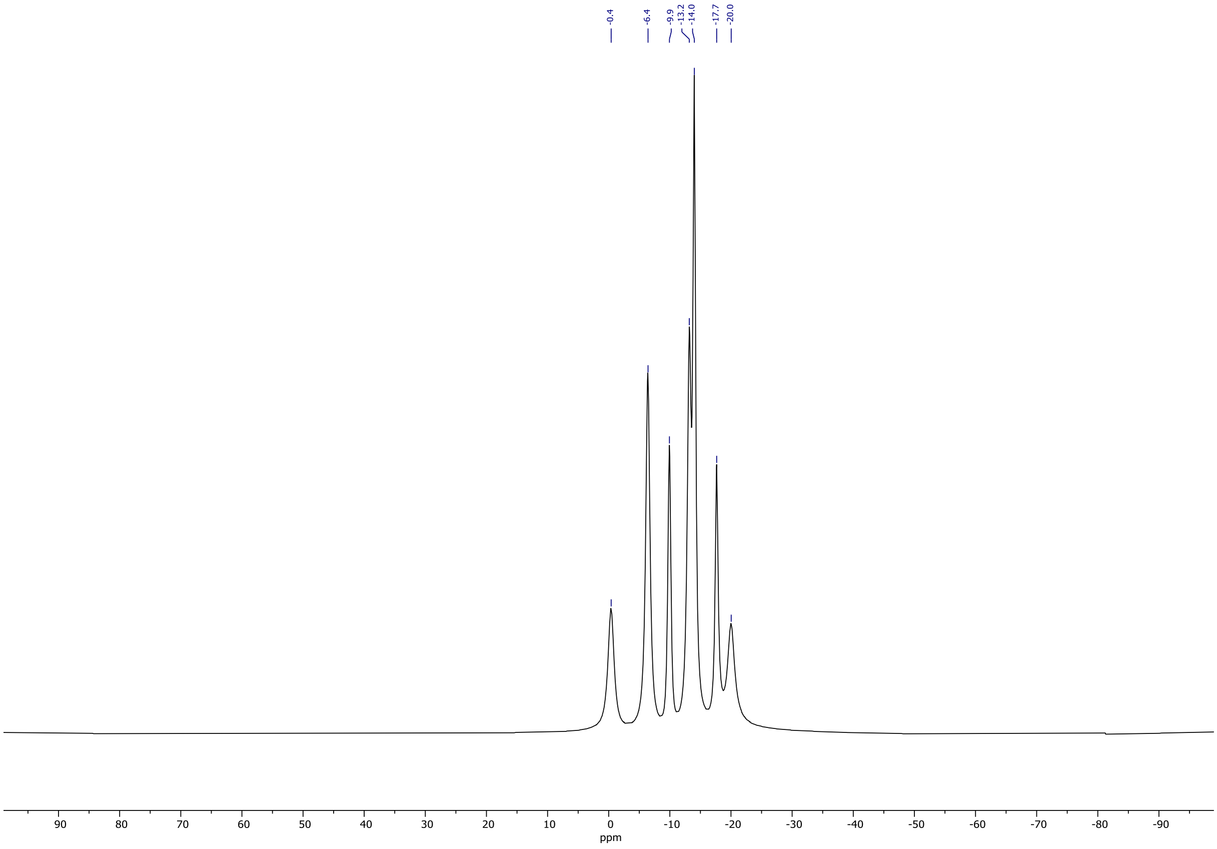

¹H NMR (CDCl$_3$, 500 MHz) for **14**

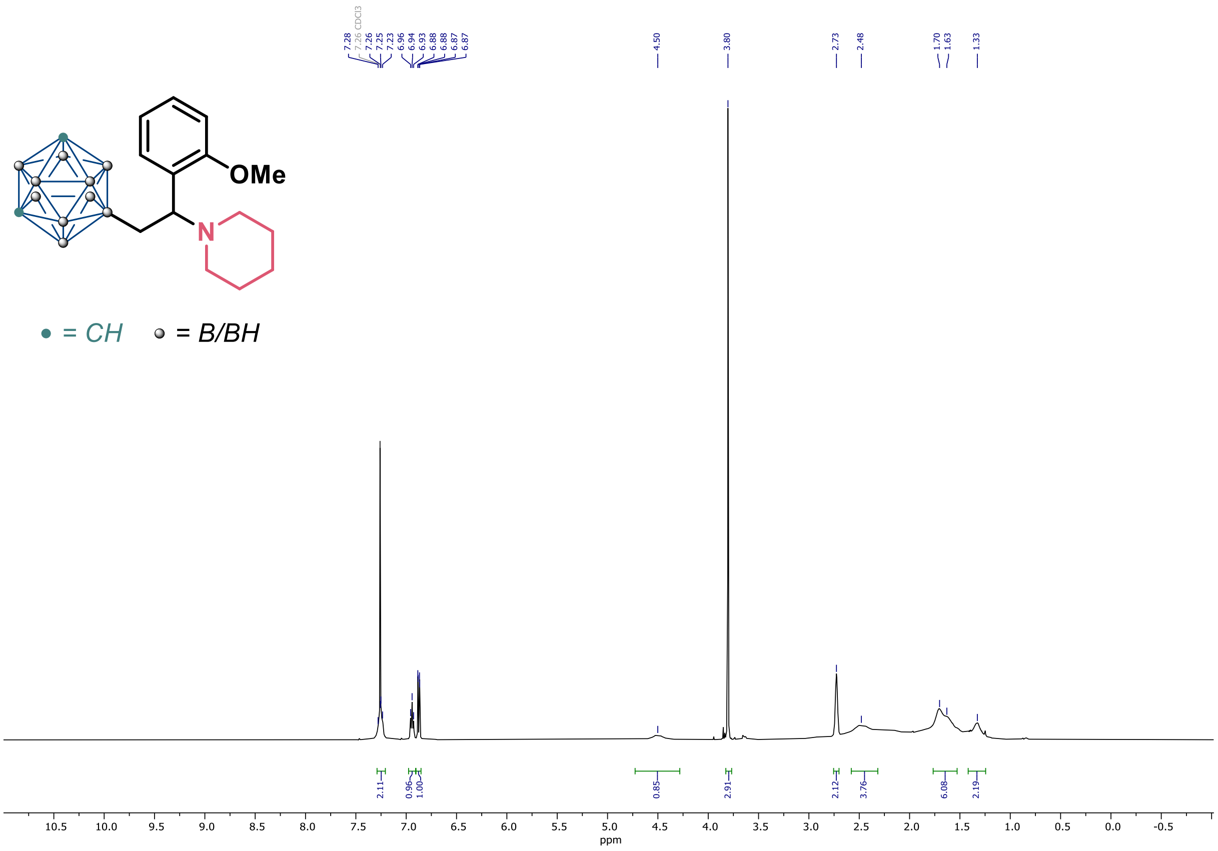

¹³C{¹H} NMR (CDCl$_3$, 126 MHz) for **14**

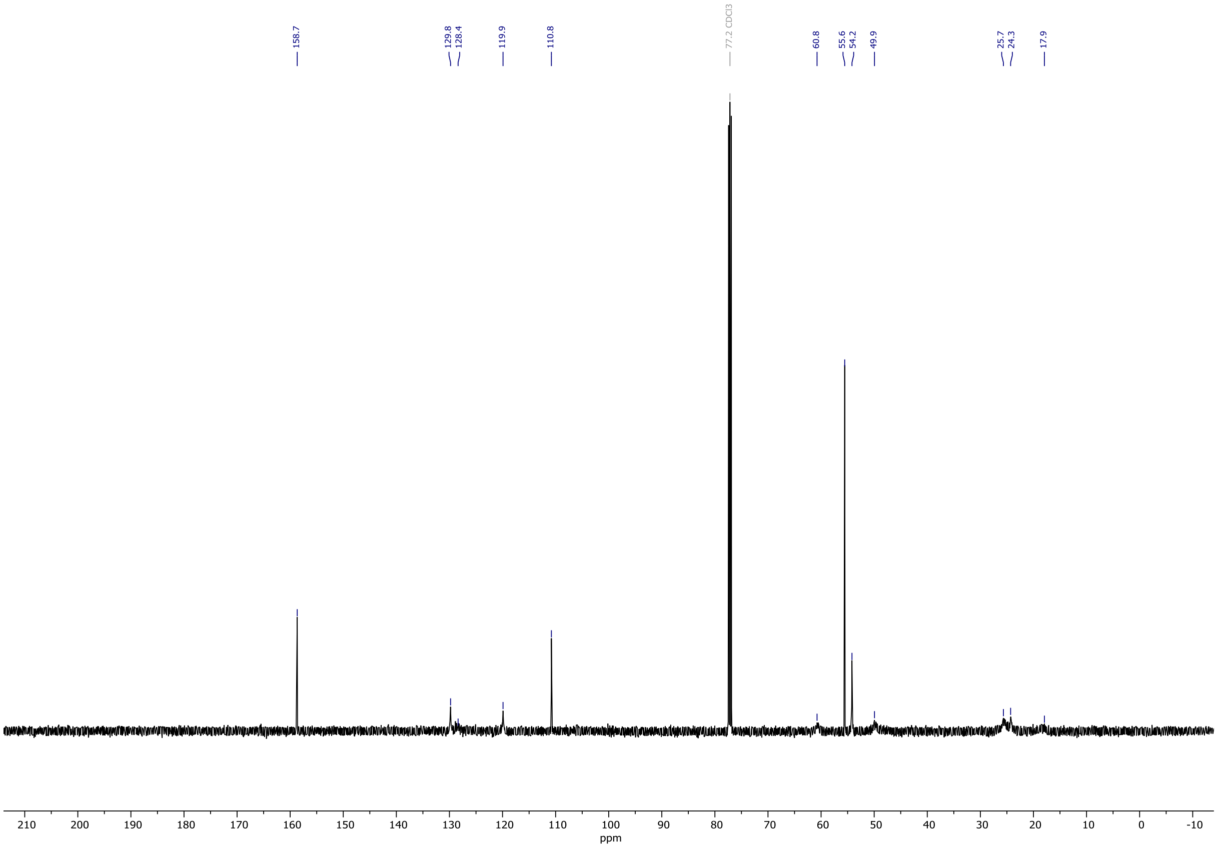

^{11}B{^{1}H} NMR (CDCl$_3$, 160 MHz) for **14**

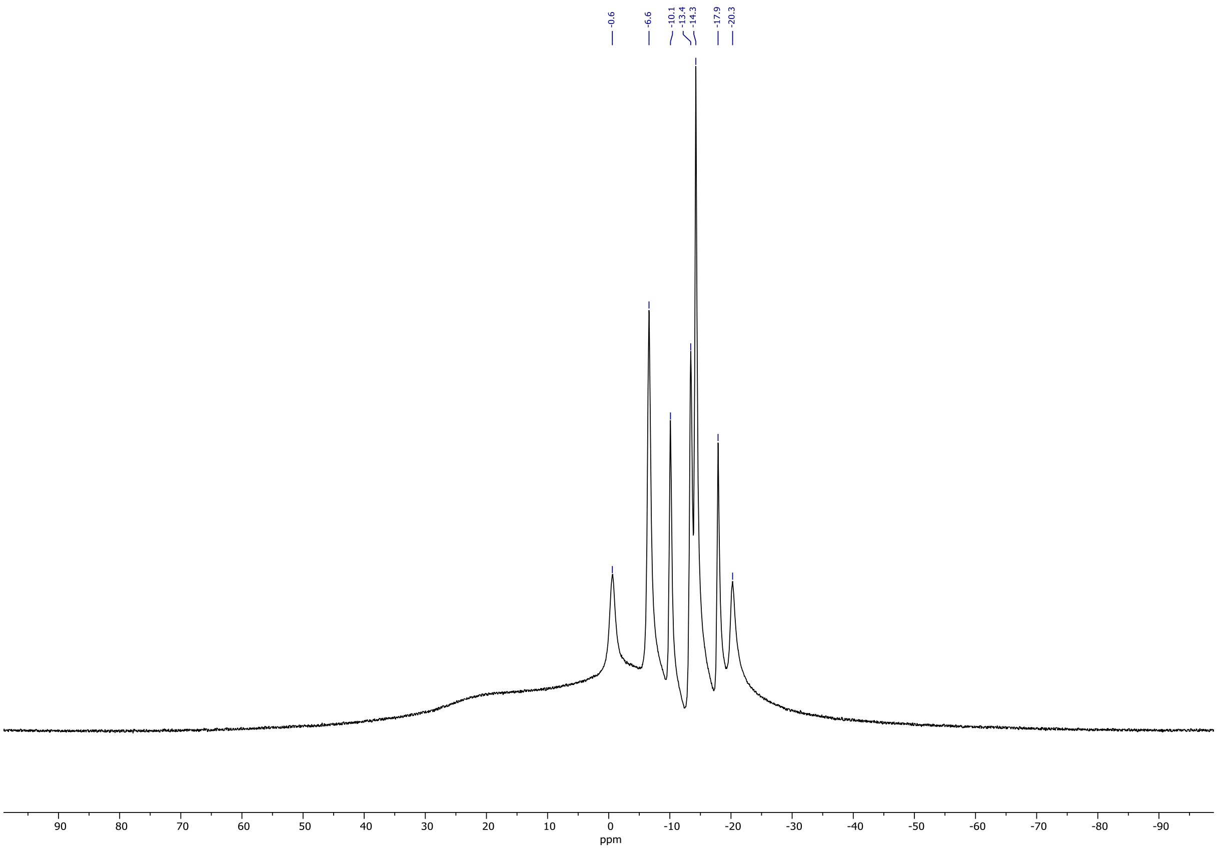

¹H NMR (D_2O, 400 MHz) for **15**

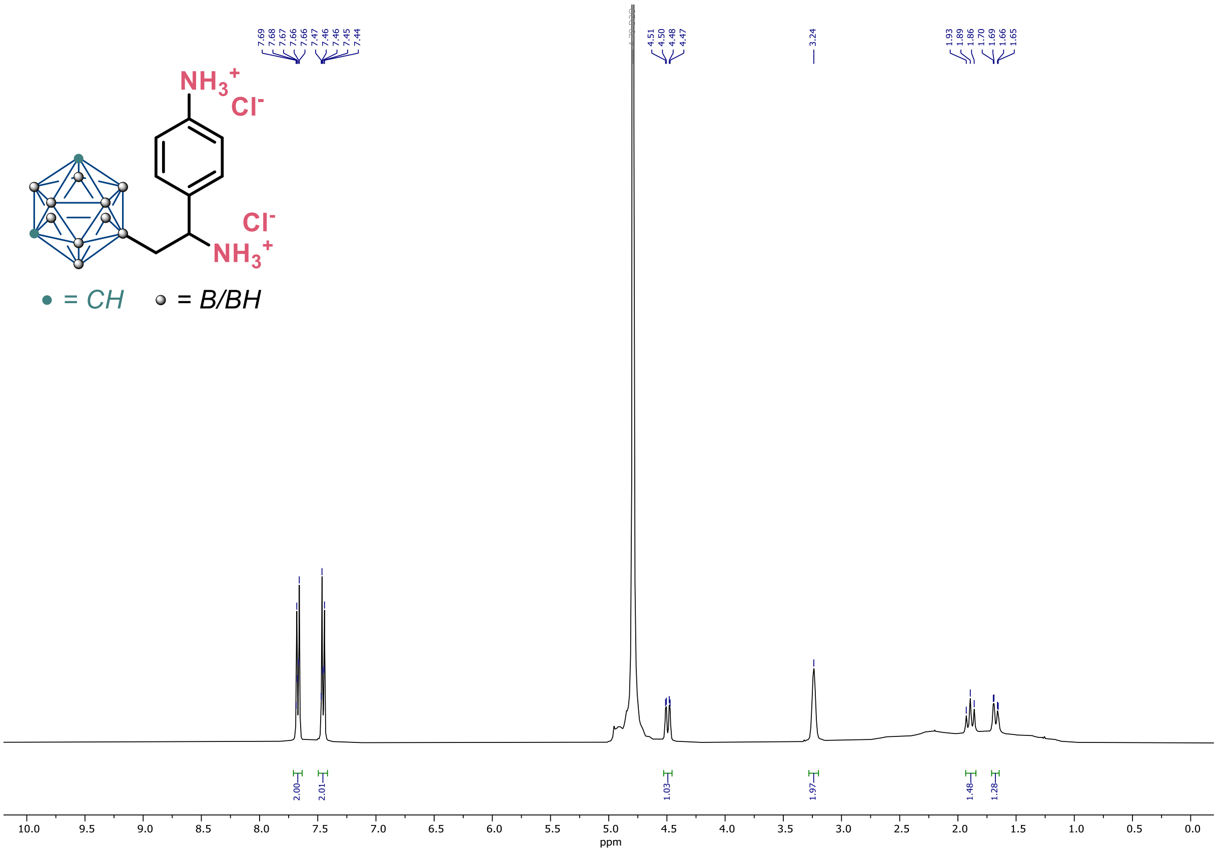

¹³C{¹H} NMR (D_2O, 101 MHz) for **15**

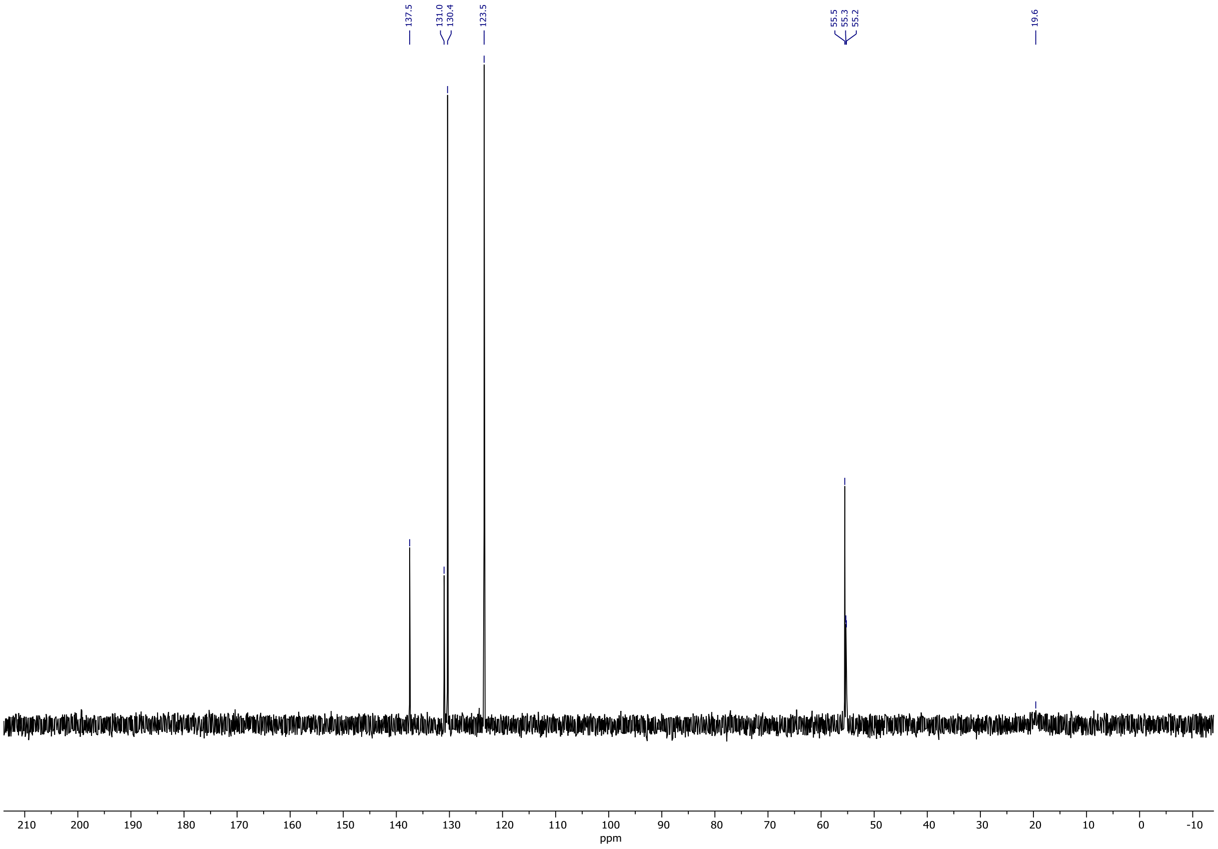

$^{11}B\{^1H\}$ **NMR** (D_2O, 128 MHz) for **15**

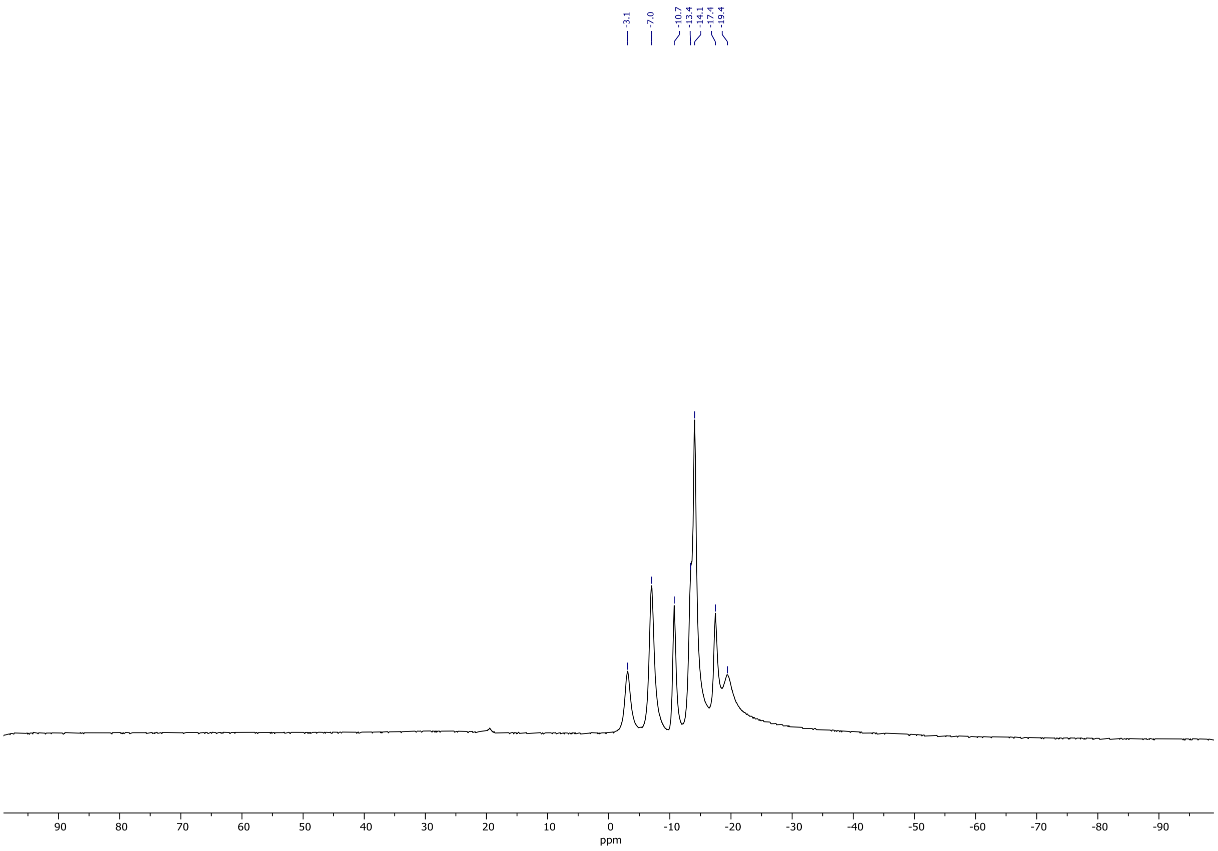

^{1}H NMR (D_2O, 400 MHz) for **16**

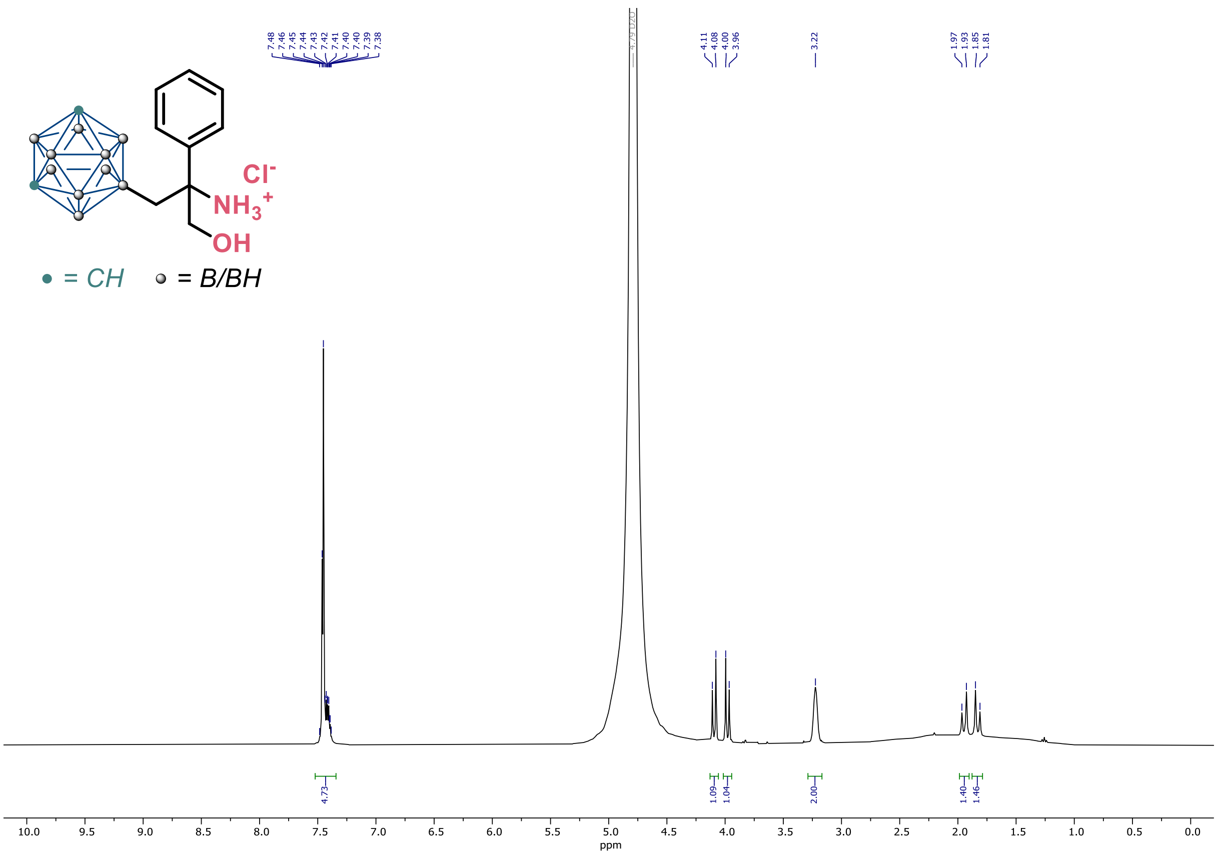

^{13}C{^{1}H} NMR (D_2O, 101 MHz) for **16**

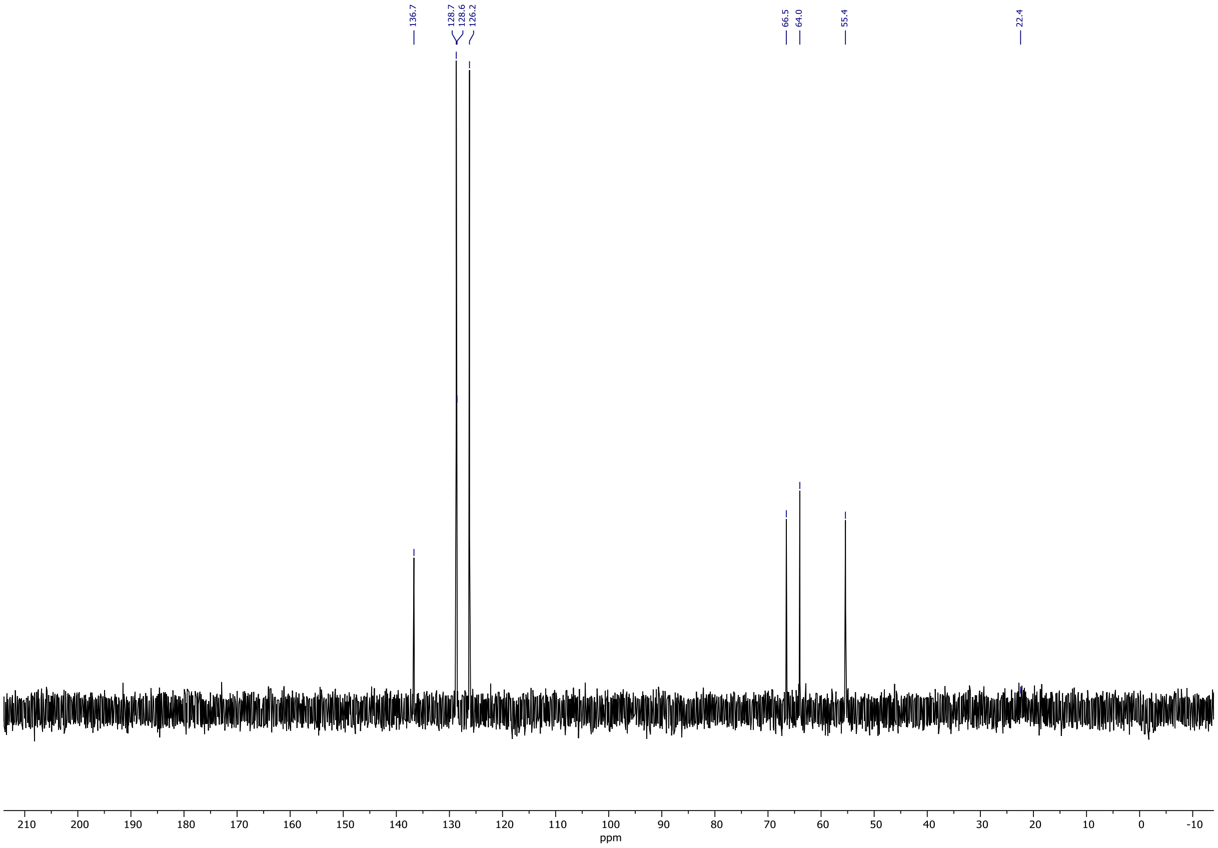

$^{11}B\{^{1}H\}$ NMR (D_2O, 128 MHz) for **16**

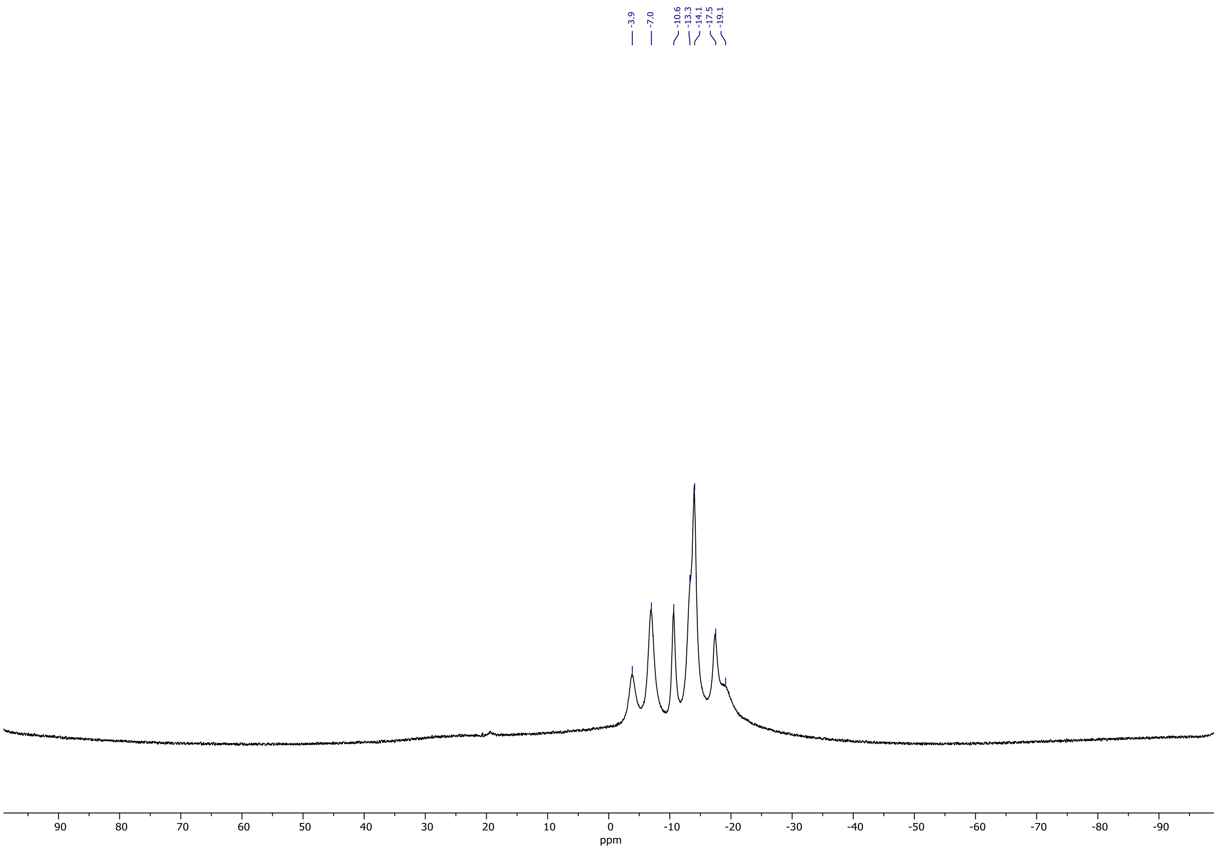

^{1}H NMR (D_2O, 400 MHz) for **17**

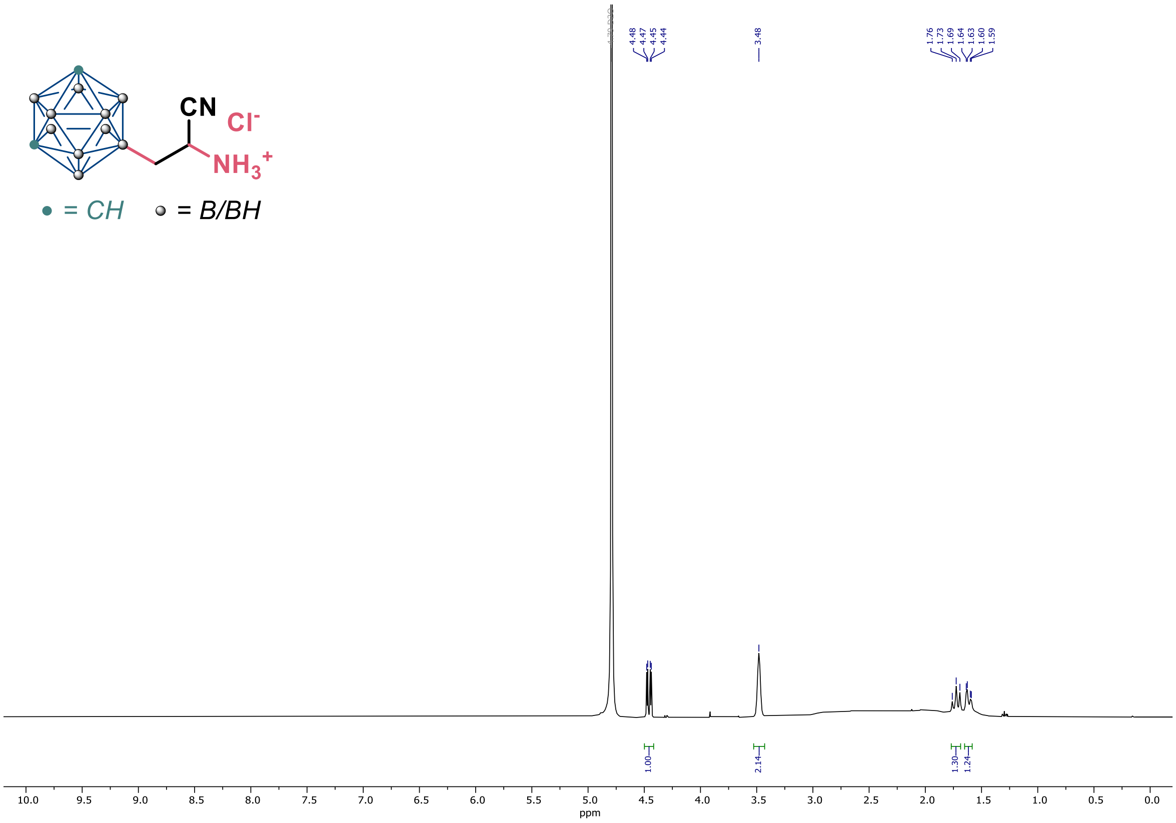

^{13}C{^{1}H} NMR (D_2O, 101 MHz) for **17**

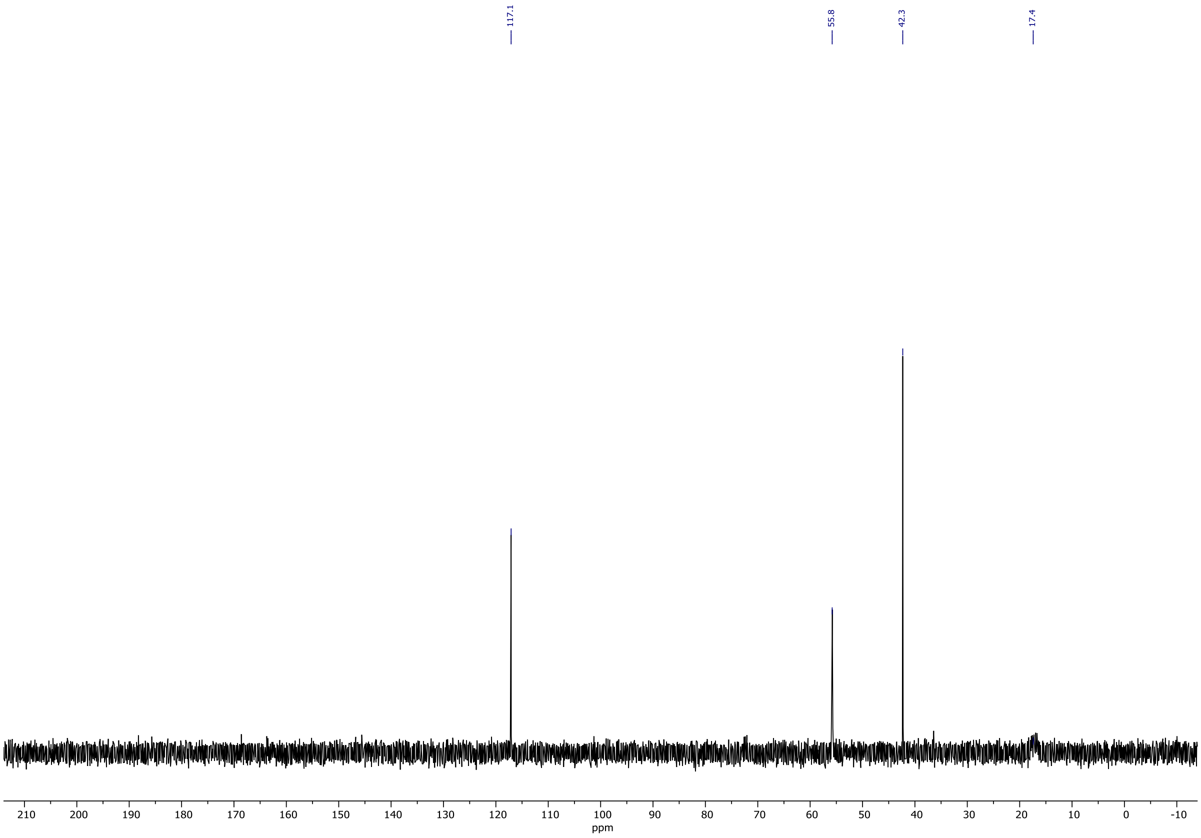

$^{11}B\{^{1}H\}$ NMR (D_2O, 128 MHz) for **17**

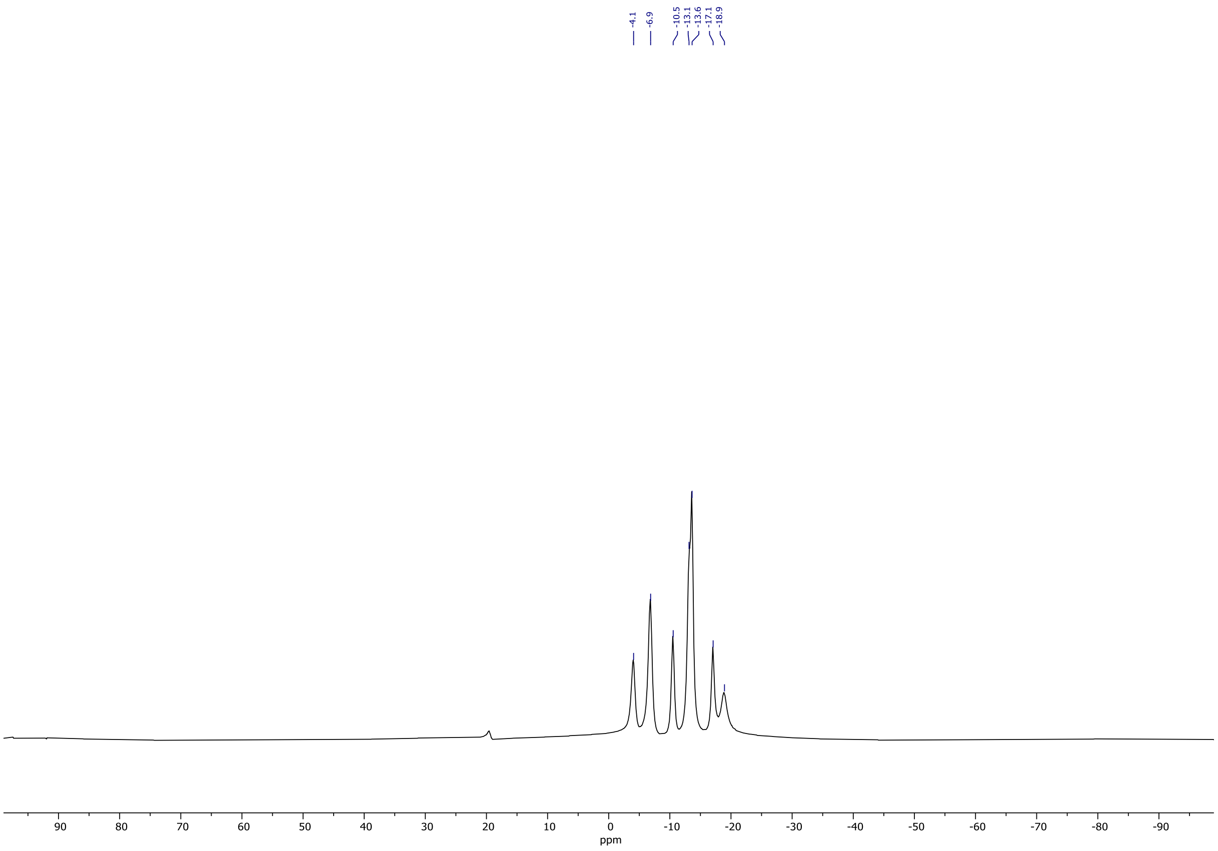

^{1}H NMR (CD_2Cl_2, 400 MHz) for **18**

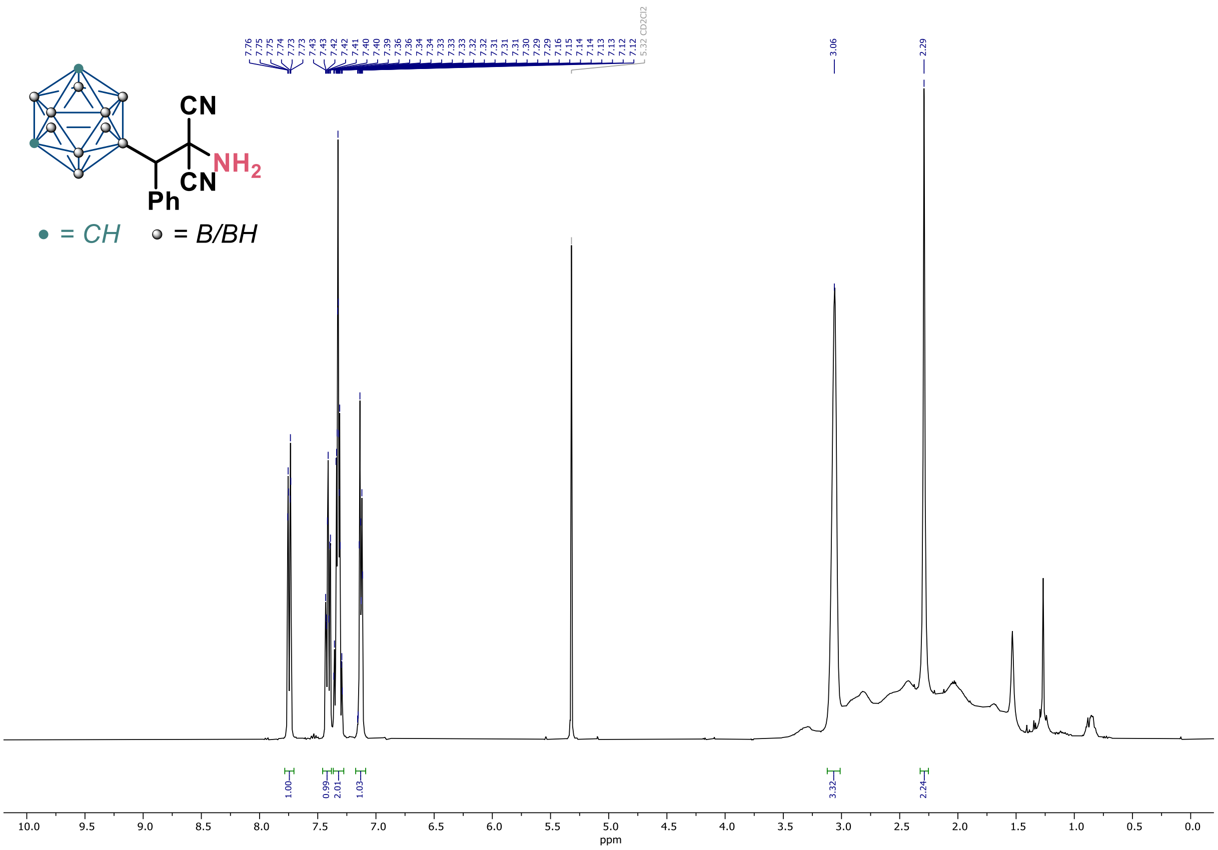

^{13}C{^{1}H} NMR (CD_2Cl_2, 101 MHz) for **18**

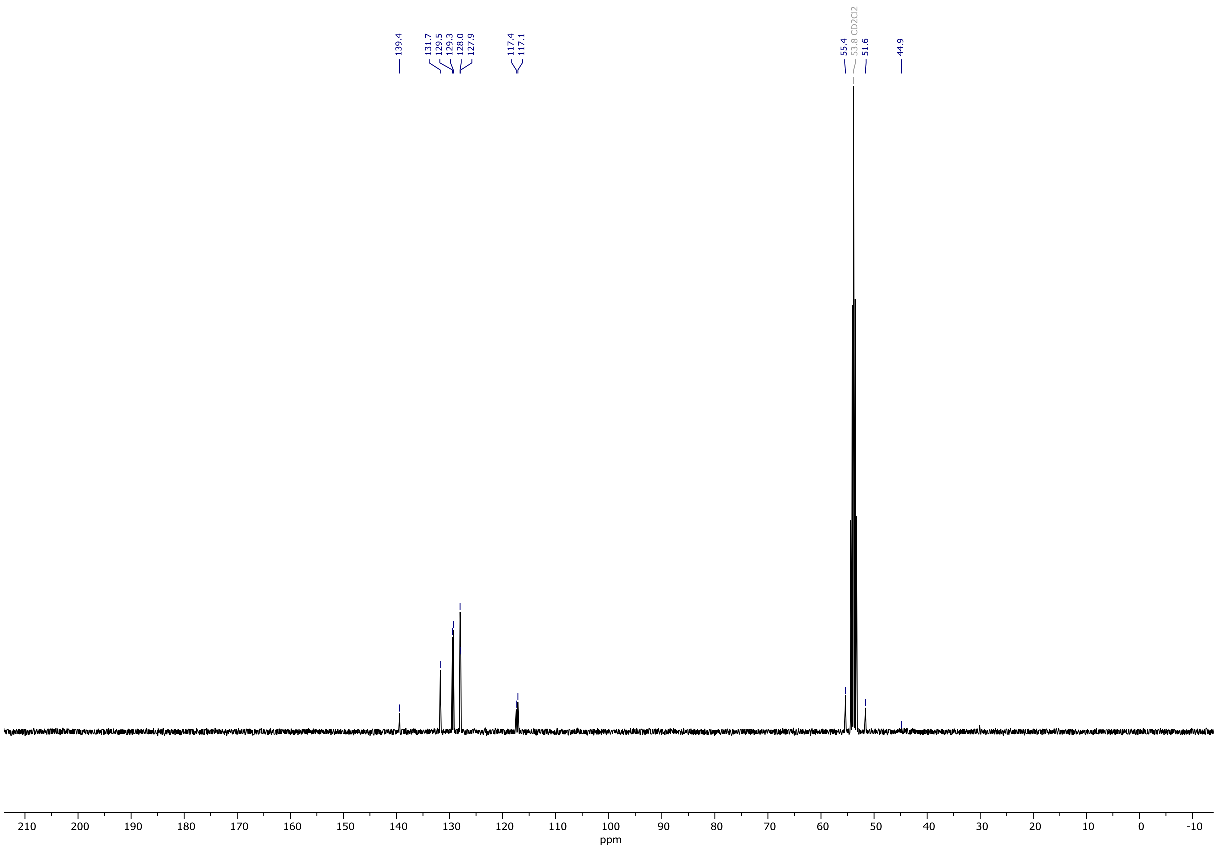

$^{11}B\{^1H\}$ NMR (CD_2Cl_2, 128 MHz) for **18**

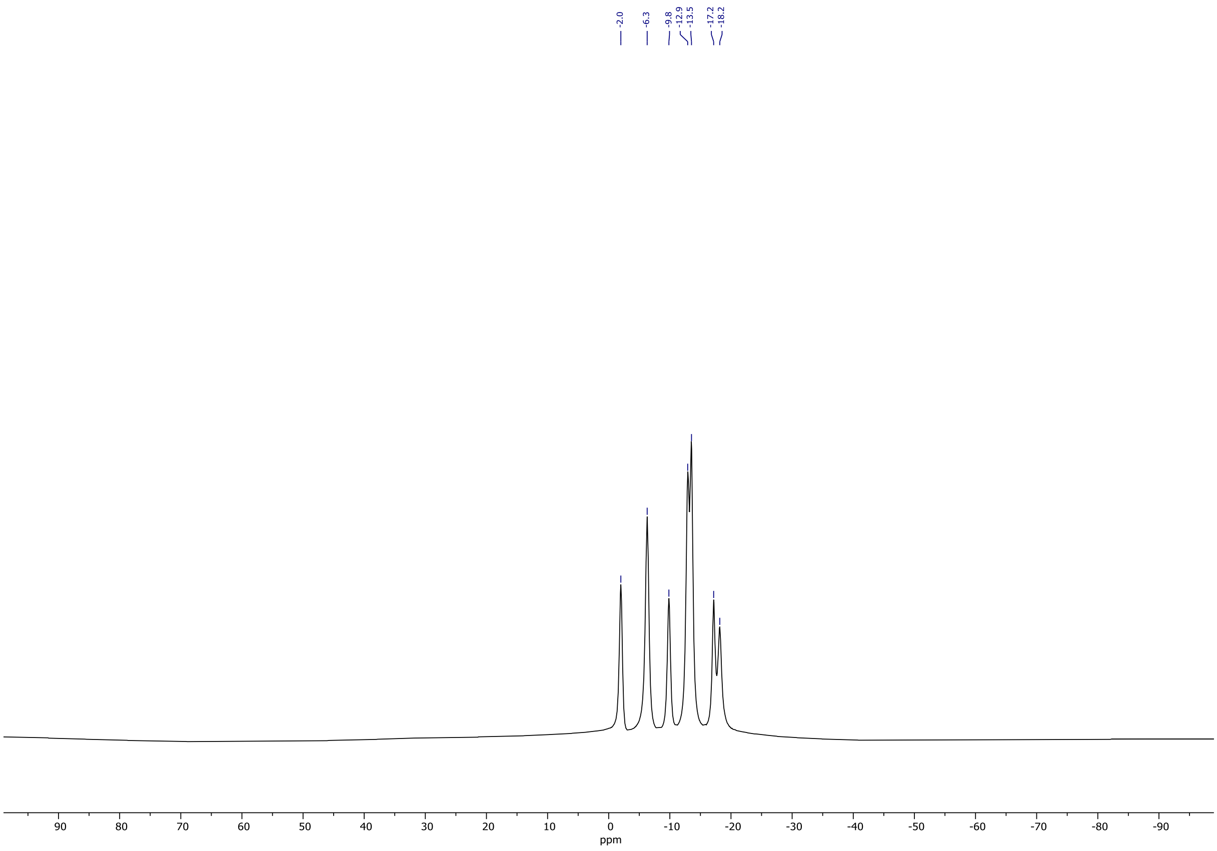

^{1}H NMR (CDCl$_3$, 400 MHz) for **19**

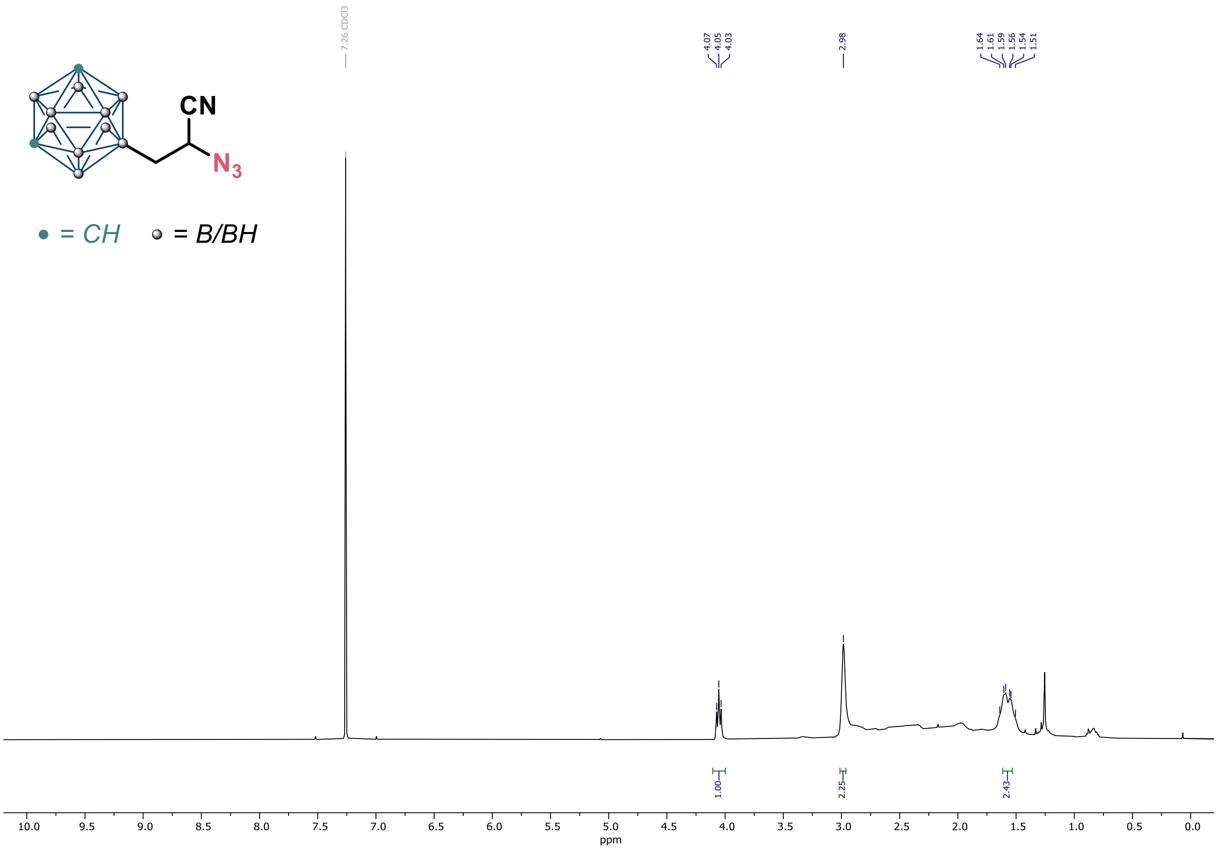

^{13}C{^{1}H} NMR (CDCl$_3$, 101 MHz) for **19**

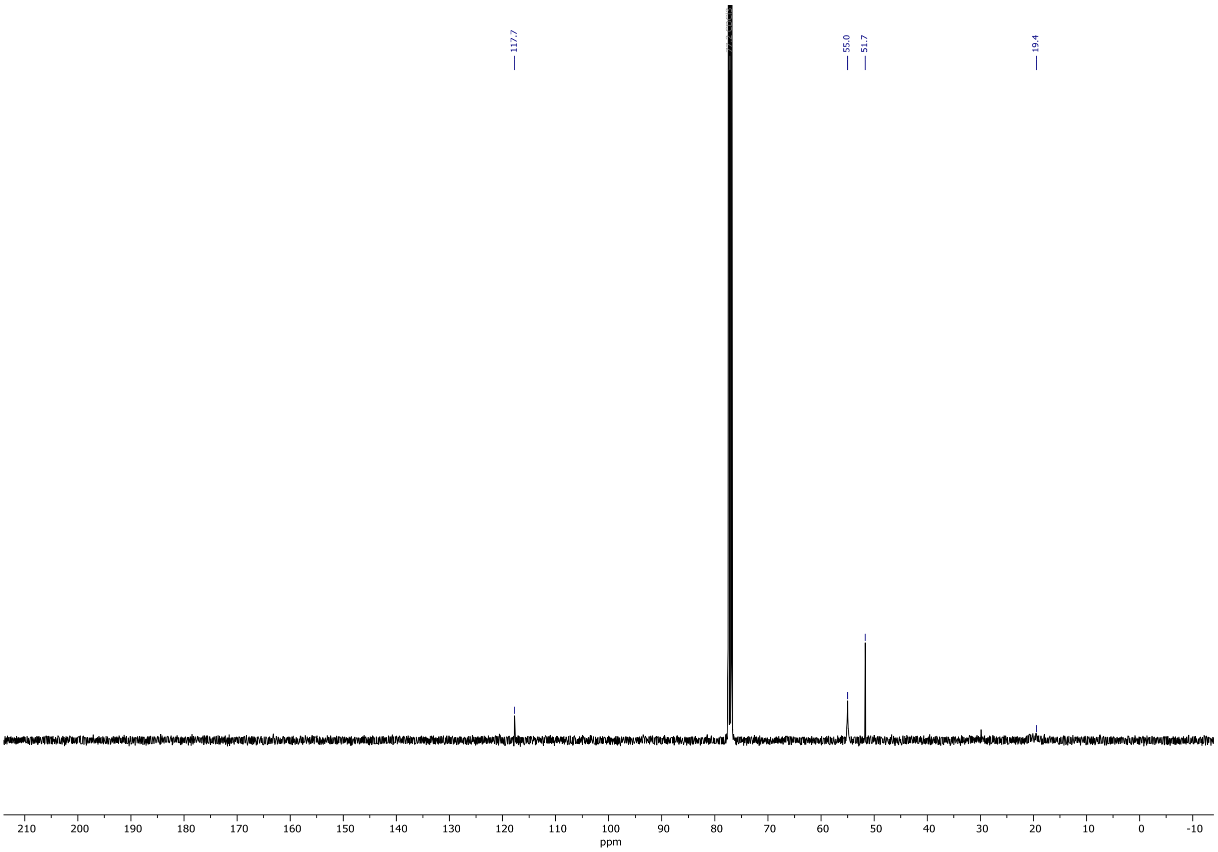

$^{11}B\{^{1}H\}$ NMR ($CDCl_3$, 128 MHz) for **19**

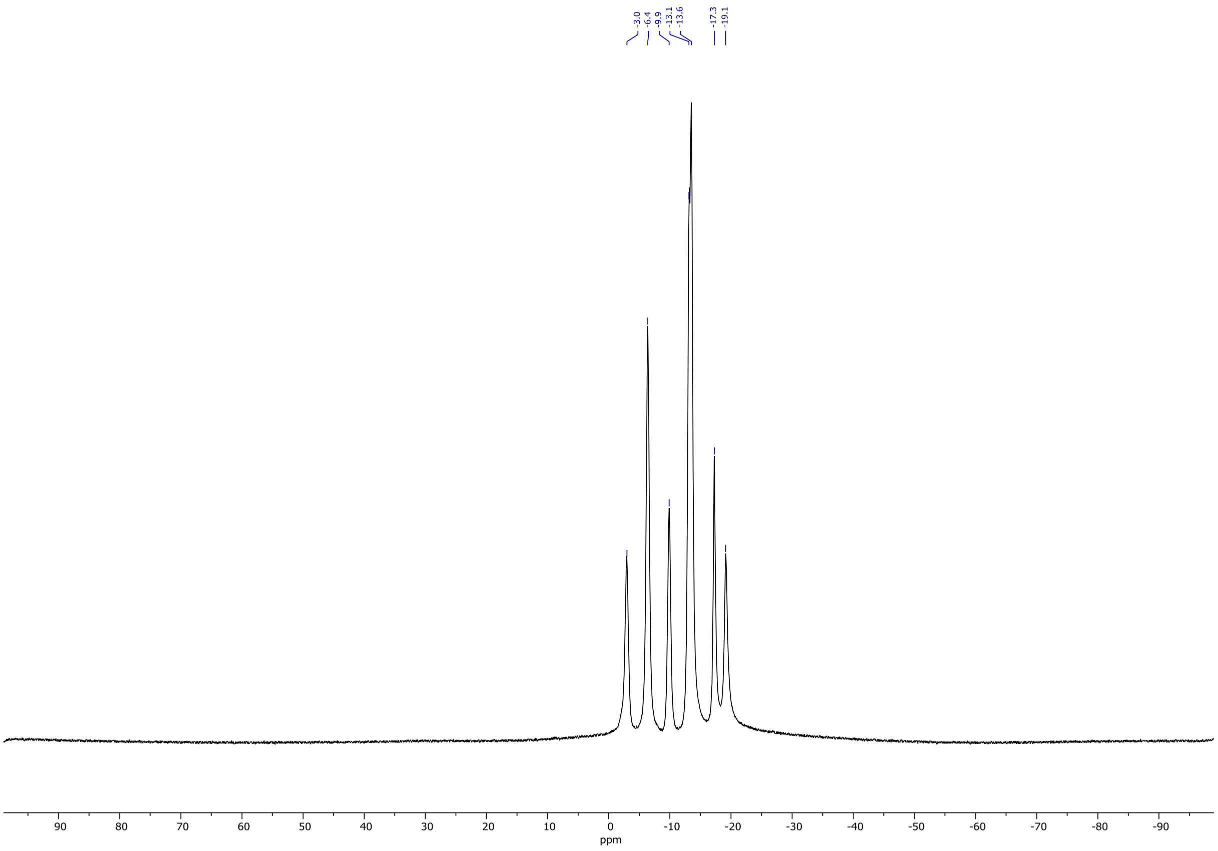

¹H NMR (CDCl$_3$, 400 MHz) for **20**

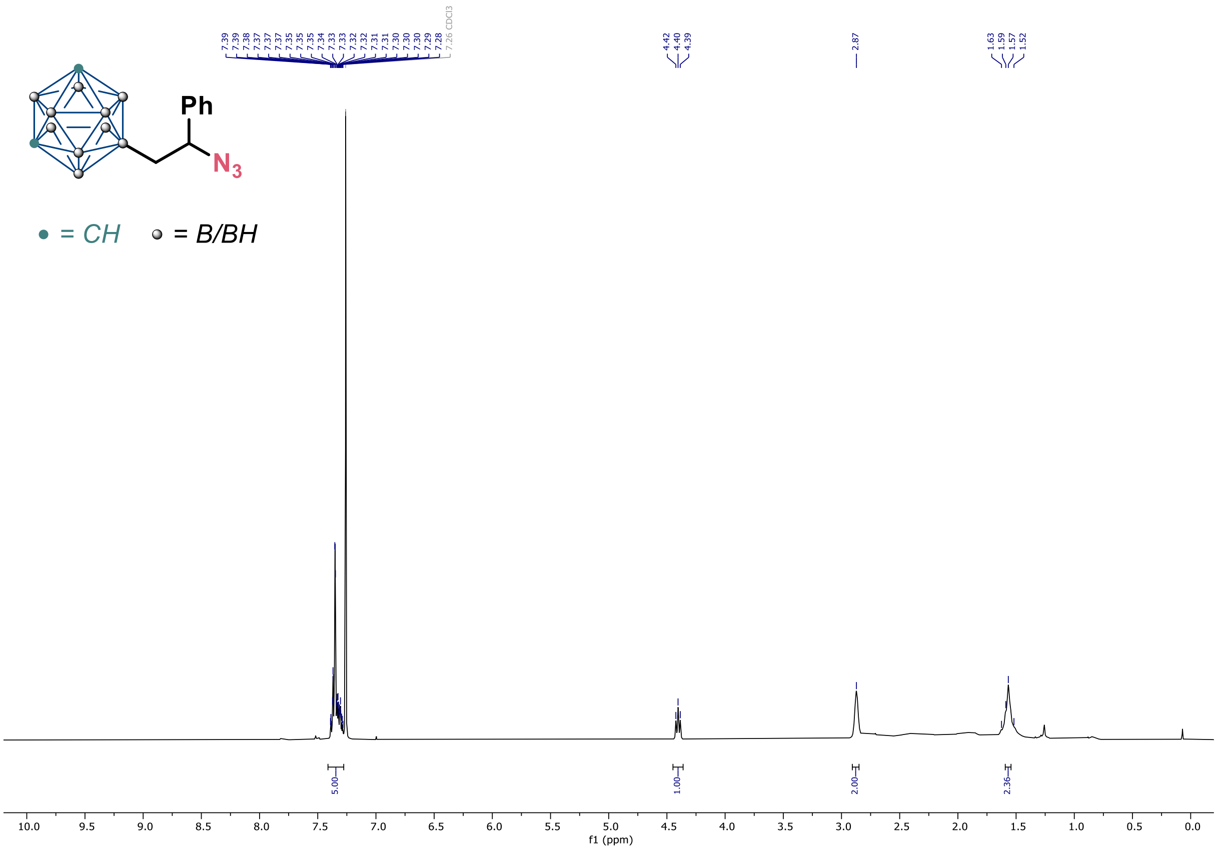

¹³C{¹H} NMR (CDCl$_3$, 101 MHz) for **20**

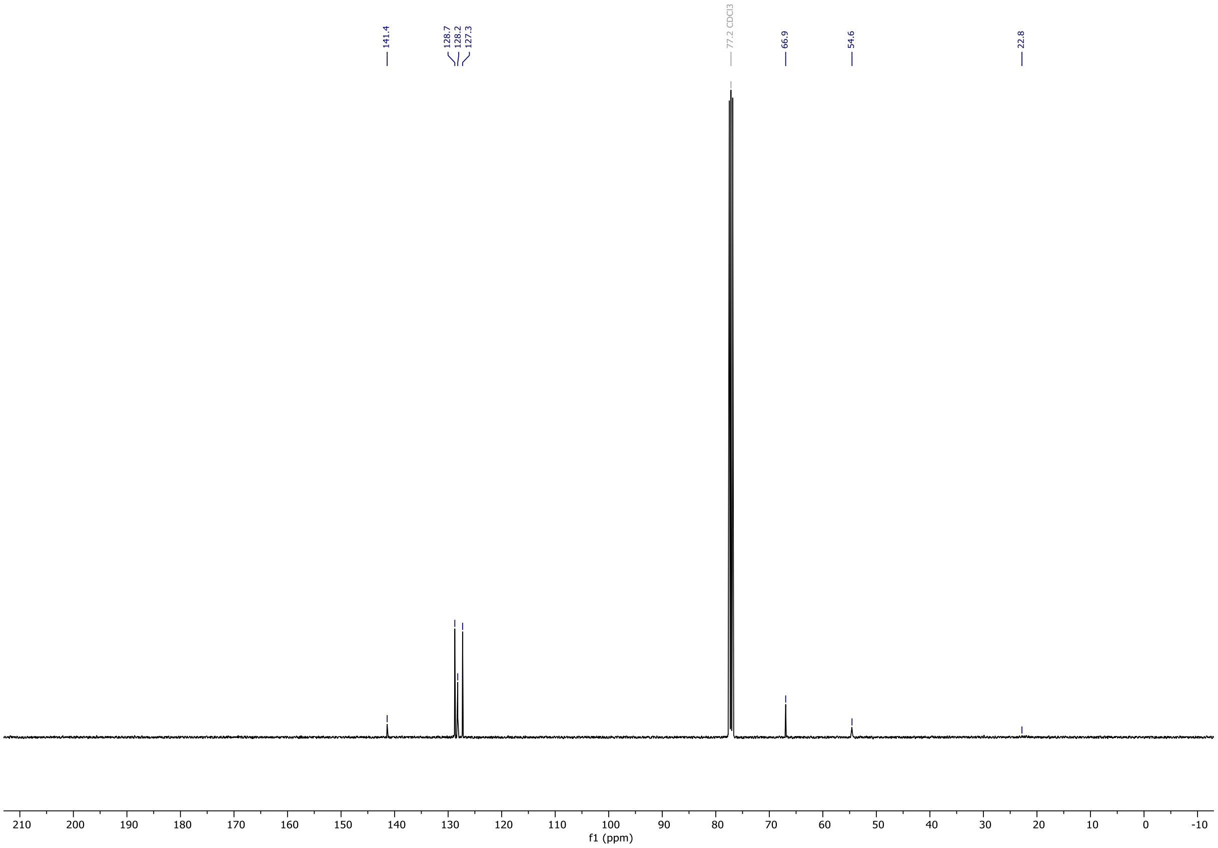

^{11}B{^{1}H} NMR (CDCl$_3$, 128 MHz) for **20**

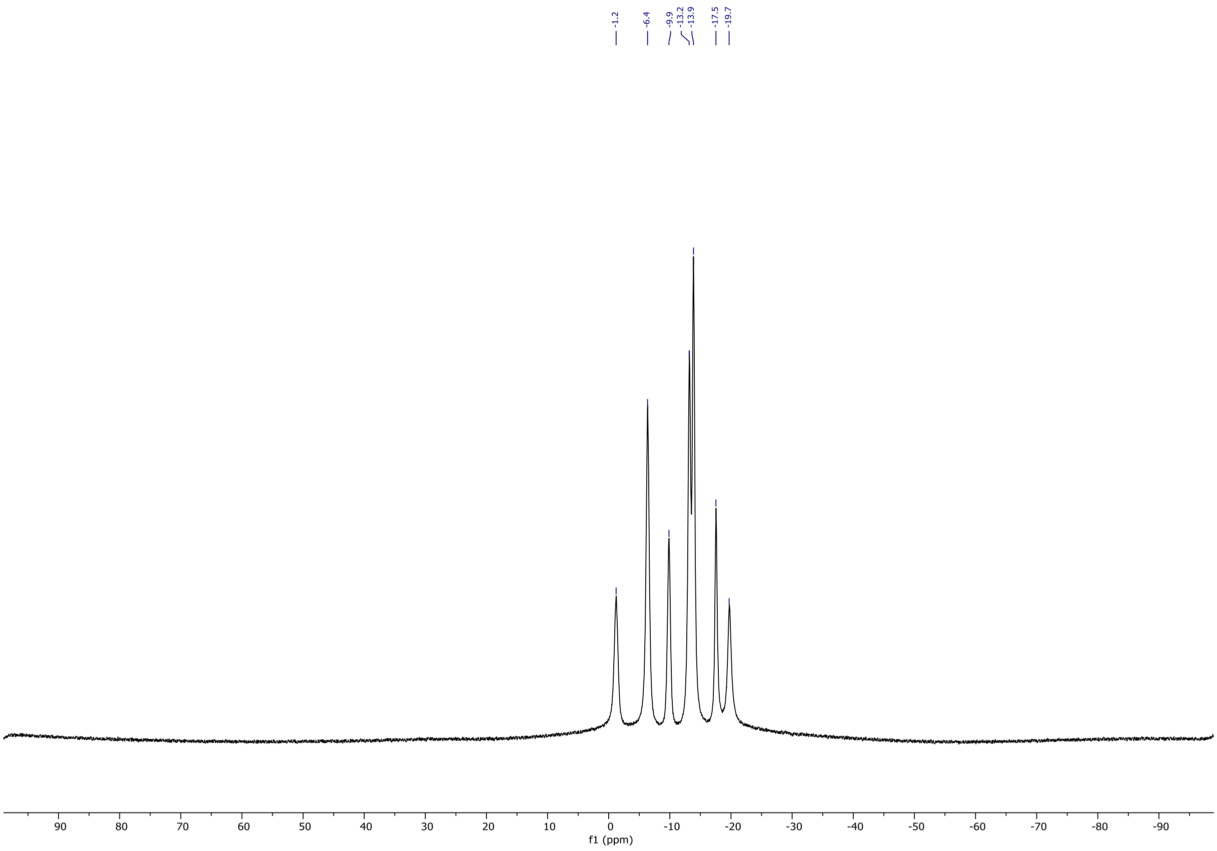

9. References

(1) Rossolini, T.; Leitch, J. A.; Grainger, R.; Dixon, D. J. Photocatalytic Three-Component Umpolung Synthesis of 1,3-Diamines. *Org. Lett.* **2018**, *20*, 6794–6798.

(2) Chen, M.; Xu, J.; Zhao, D.; Sun, F.; Tian, S.; Tu, D.; Lu, C.; Yan, H. Site-Selective Functionalization of Carboranes at the Electron-Rich Boron Vertex: Photocatalytic B-C Coupling via a Carboranyl Cage Radical. *Angew. Chem. Int. Ed.* **2022**, *61*, e202205672.

(3) Asawa, Y.; Nishida, K.; Kawai, K.; Domae, K.; Ban, H. S.; Kitazaki, A.; Asami, H.; Kohno, J.-Y.; Okada, S.; Tokuma, H.; Sakano, D.; Kume, S.; Tanaka, M.; Nakamura, H. Carborane as an Alternative Efficient Hydrophobic Tag for Protein Degradation. *Bioconjugate Chem.* **2021**, *32*, 2377–2385.

(4) Patra, T.; Mukherjee, S.; Ma, J.; Strieth-Kalthoff, F.; Glorius, F. Visible-Light-Photosensitized Aryl and Alkyl Decarboxylative Functionalization Reactions. *Angew. Chem. Int. Ed.* **2019**, *58*, 10514–10520.

(5) Paulus, F.; Stein, C.; Heusel, C.; Stoffels, T. J.; Daniliuc, C. G.; Glorius, F. Three-Component Photochemical 1,2,5-Trifunctionalizations of Alkenes toward Densely Functionalized Lynchpins. *J. Am. Chem. Soc.* **2023**, *145*, 23814–23823.

(6) Zhang, M.; Chen, M.; Ding, X.; Kang, J.; Gao, Y.; He, X.; Wang, Z.; Lu, A.; Wang, Q. The photoredox-catalyzed hydrosulfamoylation of styrenes and its application in the novel synthesis of naratriptan. *Chem. Commun.* **2021**, *57*, 9140–9143.

(7) Vassilikogiannakis, G.; Hatzimarinaki, M.; Orfanopoulos, M. Mechanism of the [2 + 2] Photocycloaddition of Fullerene C60 with Styrenes. *J. Org. Chem.* **2000**, *65*, 8180–8187.

(8) Min, G. K.; Bjerglund, K.; Kramer, S.; Gøgsig, T. M.; Lindhardt, A. T.; Skrydstrup, T. Generation of stoichiometric ethylene and isotopic derivatives and application in transition-metal-catalyzed vinylation and enyne metathesis. *Chem. Eur. J.* **2013**, *19*, 17603–17607.

(9) Bellido, M.; Garçon, M.; Verdaguer, X.; Riera, A. Three-Component Palladium-Catalyzed Tandem Suzuki-Miyaura/Allylic Substitution: A Regioselective Synthesis of (2-Arylallyl) Aryl Sulfones. *Adv. Synth. Catal.* **2024**, *366*, 2791–2800.

(10) Xu, J.; Zhou, Y.; Liu, B. Dicarbofunctionalization of Vinylarenes with Pyridine and Aldehydes via Photocatalytic Hydrogen Atom Transfer. *J. Org. Chem.* **2024**, *89*, 15877–15883.

(11) Wang, G.-Z.; Shang, R.; Fu, Y. Irradiation-Induced Palladium-Catalyzed Decarboxylative Heck Reaction of Aliphatic N-(Acyloxy)phthalimides at Room Temperature. *Org. Lett.* **2018**, *20*, 888–891.

(12) Pitzer, L.; Schäfers, F.; Glorius, F. Rapid Assessment of the Reaction-Condition-Based Sensitivity of Chemical Transformations. *Angew. Chem. Int. Ed.* **2019**, *58*, 8572–8576.

(13) Miura, K.; Fujisawa, N.; Saito, H.; Wang, D.; Hosomi, A. Synthetic utility of stannyl enolates as radical alkylating agents. *Org. Lett.* **2001**, *3*, 2591–2594.

(14) Giese, B.; Damm, W.; Wetterich, F.; Zeitz, H.-G. 1,2-Stereoinduction in acyclic radicals: allylic strain effects. *Tetrahedron Lett.* **1992**, *33*, 1863–1866.

(15) Jiang, Y.-S.; Liang, F.; Chen, A.-M.; Li, S.-S.; Luo, X.-L.; Xia, P.-J. Photocatalytic Regio- and Site-Selective Alkylamination of Coumarins: Access to 3-Amino- and 4-Amino Dihydrocoumarins. *Adv. Synth. Catal.* **2023**, *365*, 997–1001.

(16) Tan, G.; Das, M.; Keum, H.; Bellotti, P.; Daniliuc, C.; Glorius, F. Photochemical single-step synthesis of β-amino acid derivatives from alkenes and (hetero)arenes. *Nat. Chem.* **2022**, *14*, 1174–1184.

(17) Stoll, S.; Schweiger, A. EasySpin, a comprehensive software package for spectral simulation and analysis in EPR. *J. Magn. Reson.* **2006**, *178*, 42–55.

(18) The MathWorks Inc., MATLAB 24.2.0.2712019 (R2024b), Natick, Massachusetts, United States, **2024**.

(19) Hunter, J. D. Matplotlib: A 2D Graphics Environment. *Comput. Sci. Eng.* **2007**, *9*, 90–95.

(20) Cismesia, M. A.; Yoon, T. P. Characterizing Chain Processes in Visible Light Photoredox Catalysis. *Chem. Sci.* **2015**, *6*, 5426–5434.

(21) Hatchard, C. G.; Parker C. A. A new sensitive chemical actinometer - II. Potassium ferrioxalate as a standard chemical actinometer. *Proc. R. Soc. London. Ser. A. Math. Phys. Sci.* **1956**, *235*, 518–536.

(22) Chen, J.; Dvornikov, A. S.; Rentzepis, P. M. Comment on "New insight into photochemistry of ferrioxalate". *J. Phys. Chem. A* **2009**, *113*, 8818-8819.

(23) Wegner, E. E.; Adamson, A. W. Photochemistry of Complex Ions. III. Absolute Quantum Yields for the Photolysis of Some Aqueous Chromium(III) Complexes. Chemical Actinometry in the Long Wavelength Visible Region. *J. Am. Chem. Soc.* **1966**, *88*, 394–404.

(24) Tan, G.; Paulus, F.; Petti, A.; Wiethoff, M.-A.; Lauer, A.; Daniliuc, C.; Glorius, F. Metal-free photosensitized radical relay 1,4-carboimination across two distinct olefins. *Chem. Sci.* **2023**, *14*, 2447–2454.

(25) Balakrishna, B.; Mossin, S.; Kramer, S. Photo-induced metal-free dehydrogenative N-N homo-coupling. *Chem. Commun.* **2022**, *58*, 10977–10980.

(26) Neese, F. The ORCA program system. *WIREs Comput. Mol. Sci.* **2012**, *2*, 73–78.

(27) Neese, F. Software update: The ORCA program system—Version 5.0. *WIREs Comput. Mol. Sci.* **2022**, *12*, e1606.

(28) Bannwarth, C.; Ehlert, S.; Grimme, S. GFN2-xTB-An Accurate and Broadly Parametrized Self-Consistent Tight-Binding Quantum Chemical Method with Multipole Electrostatics and Density-Dependent Dispersion Contributions. *J. Chem. Theory Comput.* **2019**, *15*, 1652–1671.

(29) Yanai, T.; Tew, D. P.; Handy, N. C. A new hybrid exchange–correlation functional using the Coulomb-attenuating method (CAM-B3LYP). *Chem. Phys. Lett.* **2004**, *393*, 51–57.

(30) Weigend, F.; Ahlrichs, R. Balanced basis sets of split valence, triple zeta valence and quadruple zeta valence quality for H to Rn: Design and assessment of accuracy. *Phys. Chem. Chem. Phys.* **2005**, *7*, 3297–3305.

(31) Barone, V.; Cossi, M. Quantum Calculation of Molecular Energies and Energy Gradients in Solution by a Conductor Solvent Model. *J. Phys. Chem. A* **1998**, *102*, 1995–2001.

(32) Cossi, M.; Rega, N.; Scalmani, G.; Barone, V. Energies, structures, and electronic properties of molecules in solution with the C-PCM solvation model. *J. Comput. Chem.* **2003**, *24*, 669–681.

(33) Grimme, S.; Antony, J.; Ehrlich, S.; Krieg, H. A consistent and accurate ab initio parametrization of density functional dispersion correction (DFT-D) for the 94 elements H-Pu. *J. Chem. Phys.* **2010**, *132*, 154104.

(34) Grimme, S.; Ehrlich, S.; Goerigk, L. Effect of the damping function in dispersion corrected density functional theory. *J. Comput. Chem.* **2011**, *32*, 1456–1465.

(35) Meng, G.; Guo, T.; Ma, T.; Zhang, J.; Shen, Y.; Sharpless, K. B.; Dong, J. Modular click chemistry libraries for functional screens using a diazotizing reagent. *Nature* **2019**, *574*, 86–89.

(36) Bruker AXS. *APEX4 Version 2021.4-0, SAINT Version 8.40B and SADABS Bruker AXS area detector scaling and absorption correction Version 2016/2*; Bruker AXS Inc., **2021**.

(37) Sheldrick, G. M. SHELXT - Integrated space-group and crystal-structure determination. *Acta Crystallogr. A* **2015**, *71*, 3–8.

(38) Sheldrick, G. M. Crystal structure refinement with SHELXL. *Acta Crystallogr. C* **2015**, *71*, 3–8.
